# Open Source Antibiotics: Simple Diarylimidazoles Are Potent against Methicillin-Resistant *Staphylococcus aureus*

**DOI:** 10.1021/acsinfecdis.3c00286

**Published:** 2023-11-22

**Authors:** Dana M. Klug, Edwin G. Tse, Daniel G. Silva, Yafeng Cao, Susan A. Charman, Jyoti Chauhan, Elly Crighton, Maria Dichiara, Chris Drake, David Drewry, Flavio da Silva Emery, Lori Ferrins, Lee Graves, Emily Hopkins, Thomas A. C. Kresina, Álvaro Lorente-Macías, Benjamin Perry, Richard Phipps, Bruno Quiroga, Antonio Quotadamo, Giada N. Sabatino, Anthony Sama, Andreas Schätzlein, Quillon J. Simpson, Jonathan Steele, Julia Shanu-Wilson, Peter Sjö, Paul Stapleton, Christopher J. Swain, Alexandra Vaideanu, Huanxu Xie, William Zuercher, Matthew H. Todd

**Affiliations:** † School of Pharmacy, University College London, 29-39 Brunswick Square, London WC1N 1AX, United Kingdom; ‡ School of Pharmaceutical Sciences of Ribeirão Preto, University of São Paulo, Ribeirão Preto, São Paulo 14040-903. Brazil; § WuXi AppTec (Wuhan) Co., Ltd., 666 Gaoxin Road, East Lake High-Tech Development Zone, Wuhan 430075, People’s Republic of China; ∥ Centre for Drug Candidate Optimization, Monash Institute of Pharmaceutical Sciences, Monash University, Parkville, VIC 3052, Australia; ⊥ Department of Chemistry and Chemical Biology, Northeastern University, Boston, Massachusetts 02115, United States; # Hypha Discovery, 154b Brook Dr, Milton, Abingdon OX14 4SD, United Kingdom; 7 UNC Lineberger Comprehensive Cancer Center, School of Medicine, University of North Carolina at Chapel Hill, Chapel Hill, North Carolina 27599, United States; 8 Structural Genomics Consortium, UNC Eshelman School of Pharmacy, University of North Carolina at Chapel Hill, Chapel Hill, North Carolina 27599, United States; 9 Department of Pharmacology, University of North Carolina at Chapel Hill, Chapel Hill, North Carolina 27599, United States; 10 Department of Medicinal & Organic Chemistry and Excellence Research Unit of ‘‘Chemistry Applied to Biomedicine and the Environment’’, Faculty of Pharmacy, University of Granada, Campus de Cartuja s/n, 18071 Granada, Spain; 11 A. L-M. Cancer Research UK Edinburgh Centre, Institute of Genetics & Cancer, University of Edinburgh, Edinburgh EH4 2XR, United Kingdom; 12 Drugs for Neglected Diseases initiative (DNDi), 15 Chemin Camille-Vidart, 1202 Geneva, Switzerland; 13 Clinical and Experimental Medicine PhD Program, University of Modena and Reggio Emilia, 41121 Modena, Italy; 14 Citizen scientist, New York, New York 11570, United States; 15 Cambridge MedChem Consulting, 8 Mangers Lane, Duxford, Cambridge CB22 4RN, United Kingdom; 16 Structural Genomics Consortium, University College London, 29-39 Brunswick Square, London WC1N 1AX, United Kingdom

**Keywords:** Drug discovery, organic synthesis, bioactive molecules, antibiotics, antibacterials, open science

## Abstract

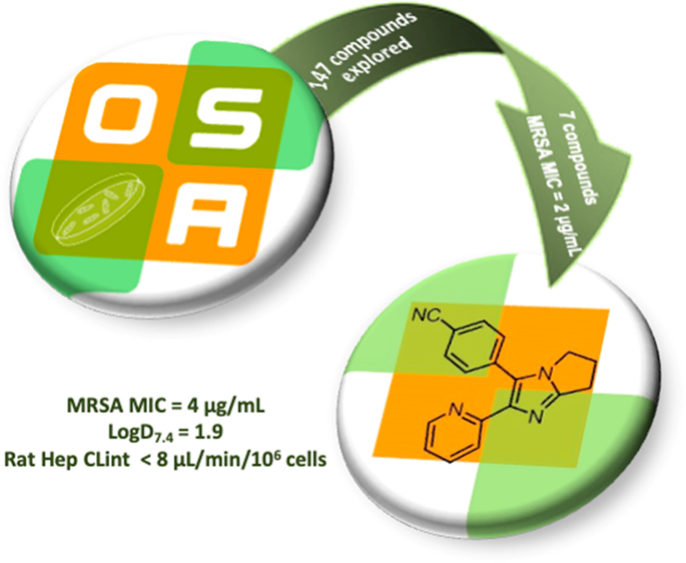

Antimicrobial resistance (AMR) is widely acknowledged as one of the most serious public health threats facing the world, yet the private sector finds it challenging to generate much-needed medicines. As an alternative discovery approach, a small array of diarylimidazoles was screened against the ESKAPE pathogens, and the results were made publicly available through the Open Source Antibiotics (OSA) consortium (https://github.com/opensourceantibiotics). Of the 18 compounds tested (at 32 μg/mL), 15 showed >90% growth inhibition activity against methicillin-resistant *Staphylococcus aureus* (MRSA) alone. In the subsequent hit-to-lead optimization of this chemotype, 147 new heterocyclic compounds containing the diarylimidazole and other core motifs were synthesized and tested against MRSA, and their structure–activity relationships were identified. While potent, these compounds have moderate to high intrinsic clearance and some associated toxicity. The best overall balance of parameters was found with OSA_975, a compound with good potency, good solubility, and reduced intrinsic clearance in rat hepatocytes. We have progressed toward the knowledge of the molecular target of these phenotypically active compounds, with proteomic techniques suggesting TGFBR1 is potentially involved in the mechanism of action. Further development of these compounds toward antimicrobial medicines is available to anyone under the licensing terms of the project.

Antimicrobial resistance (AMR) is widely acknowledged as one of the most serious public health threats facing the world.^1−4^ The problem may have been worsened by the overuse of antibiotics to treat COVID-19 patients during the pandemic.^5,6^ Unfortunately, those antibiotics rendered ineffective by resistance are not being replaced at the rate that society needs. Of the 43 antibiotics in clinical development as of December 2020, only 6 have the requisite activity against World Health Organization (WHO) “critical” or Centers for Disease Control and Prevention (CDC) “urgent” pathogens, and only 10 represent a novel class or target.^7^ This shortfall is primarily due to the high cost of antibiotic development and the challenges of creating a market structure in which novel antibiotics are both stewarded responsibly and made profitable.^8^

In this environment, an open source model for drug discovery can offer several advantages.^9^ A project adopting this approach lacks secrecy: it takes place publicly in real time, with all communication in the public domain, and is not based on a patent model.^10^ Such an approach allows for the recruitment of diverse scientific expertise (freedom to operate is clearly delineated through the use of a Creative Commons license), reduces or eliminates unproductive duplication of effort, and enables a competitive yet collaborative assessment of strategies to solve a problem. While such a model has been previously applied to other discovery projects toward, for example, antimalarials^11^ (https://github.com/OpenSourceMalaria) and antifungals^12^ (https://github.com/OpenSourceMycetoma), the project described herein is an example of an open source drug discovery framework applied to the challenge of novel antibiotic discovery.

In an effort to discover new leads against high-priority pathogens, a small array of diarylimidazoles was screened using the Community for Open Antimicrobial Drug Discovery (CO-ADD) platform,^13^ which profiled the compounds against the ESKAPE pathogens *Escherichia coli*, *Klebsiella pneumoniae*, *Acinetobacter baumannii*, *Pseudomonas aeruginosa*, methicillin-resistant *Staphylococcus aureus* (MRSA), and the fungi *Candida neoformans* and *C. albicans*. Of the 18 compounds tested (at 32 μg/mL), 15 showed >90% growth inhibition activity against MRSA but not the other pathogens (Supporting Information - Biology, Figure S1 and S2). MRSA is a Gram-positive pathogenic bacterium that was designated a high-priority pathogen by the WHO.^4^ AMR was one of the greatest public health concerns prior to the COVID-19 pandemic; MRSA infections increased by 13% from 2019 to 2020.^2^ Given the potential public health benefits (particularly of narrow-spectrum antibiotics on host health),^15^ we decided to pursue hit-to-lead optimization of this series, which is exemplified by **OSA_822** (MRSA-active) and **OSA_812** (MRSA-inactive) shown in Figure 1. [Note that in this paper the numbering of the compounds retains the numbering used in the online Open Source Antibiotics project (https://github.com/opensourceantibiotics), maintaining the connection between data in this paper and the live research. Molecules in Open Source Antibiotics are numbered according to a convention described in the Supporting Information Table (and online); in the rest of this paper, the prefix “**OSA_**” is omitted.]

**Figure 1:**
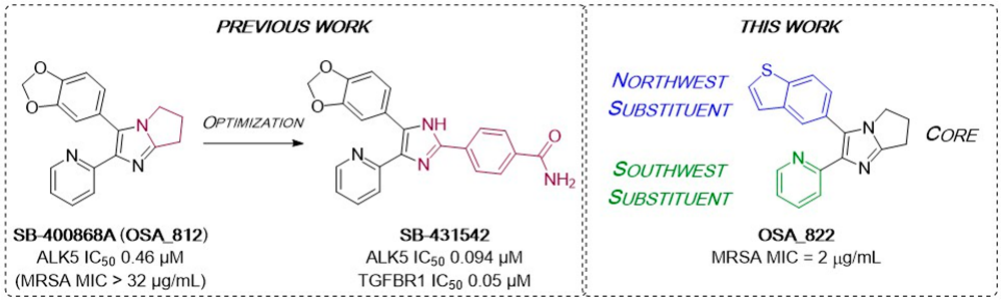
Literature optimization of diarylimidazole SB-400868A to generate ALK5 inhibitors and structure of a representative compound investigated in this work (**OSA_822**) toward compounds potent vs MRSA.

The chemotype was of previous interest because of a screen of a GlaxoSmithKline internal library collection for inhibitors of the transforming growth factor β1 (TGF-β1) type I receptor (ALK5), where compound **812** displayed an IC_50_ of 0.46 μM against ALK5 and no inhibition of p38 kinase. Optimization led to the selective inhibitor SB-431542 (IC_50_ = 94 nM against ALK5 and still no inhibition of p38 kinase, CC_50_ > 30 μM), which inhibits TGF-β1-induced fibronectin mRNA formation (IC_50_ = 0.05 μM) while displaying no measurable cytotoxicity in a 48 h XTT assay (CC_50_ > 30 μM).^16^

The promising activity against MRSA and the low cytotoxicity observed in the ALK5 research stimulated us to pursue a new hit-to-lead campaign. Three regions of the main scaffold were modified: the bicyclic imidazole core (black), the “northwest” aryl substituent (blue), and the “southwest” aryl substituent (green). Diversification of these motifs as well as their linkages formed the basis of our synthetic studies of this chemotype. The aim was to generate potent compounds with promising physicochemical properties and improved metabolic stability while maintaining an acceptable toxicity vs mammalian cells.

## Results and Discussion

### Synthesis

**Scheme 1:**
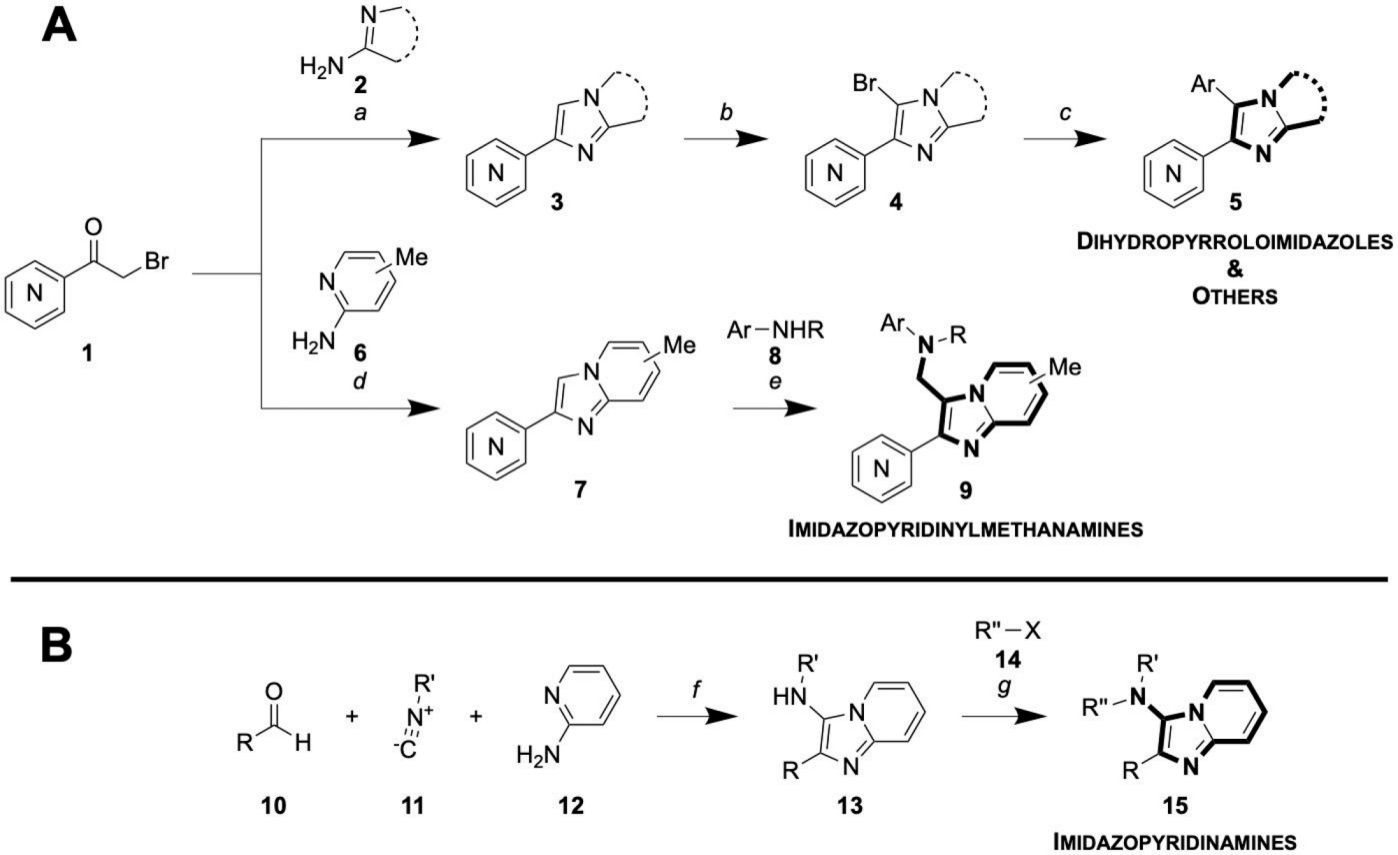
Synthetic Routes to (A) General (Aryl–Aryl Linkage) Analogues and (B) N-Linked Analogues Reagents and conditions for part A: (*a*) **2**, DMF, 100 °C, 18 h; (*b*) NBS, DCM, rt, 1 h; (*c*) ArB(OH)_2_ or ArB(pin), Na_2_CO_3_, Pd(PPh_3_)_4_ or PdCl_2_(dppf)CH_2_Cl_2_, 3:1 PhMe:EtOH, 120 °C, 18 h (conventional heating) or 30 min (μW reactor); (*d*) **6**, NaHCO_3_, MeOH, reflux, 12 h; (*e*) **8**, formalin (37% aq.), acetic acid, DCM, 18 h. Reagents and conditions for part B: (*f*) Yb(OTf)_3_, 120 °C, 30 min (μW reactor); (*g*) **14**, Cs_2_CO_3_, DMF, rt, 18 h. A phenyl ring containing a central “N” denotes a general (aza)aromatic. Yields are described in Supplementary Information - Chemistry.

Most compounds synthesized for this campaign followed a general three-step route (Scheme 1A); all chemical experiments are available in full in the relevant “live” online electronic laboratory notebooks as well as in snapshot copies archived in an electronic university repository.^17^ The starting α-bromoketones **1** were cyclized with the desired 2-amino heterocycles **2** to afford fused bicycles **3** that were brominated at the 5-position of the imidazole ring to give **4**. Final compounds **5** were synthesized using standard Suzuki coupling conditions under either conventional or microwave heating.

Several analogues were made with a methylene linker between the core and the aryl amine substituent (**9**). These were prepared via an analogous method, starting with a condensation–cyclization of the appropriate α-bromoketone **1** and aminopyridine **6** to give the imidazopyridine core **7**. A Mannich reaction allowed the aminoalkylations necessary for the imidazopyridine derivatives **9**;^18,19^ some anilines that were used in this reaction (**8**) were alkylated prior to the Mannich step (see Supporting Information - Chemistry).

Nitrogen-linked analogues were accessed via a one-pot Yb(OTf)_3_-catalyzed Groebke–Blackburn–Bienaymé reaction with aldehyde **10**, isonitrile **11**, and aminopyridine **12** to afford compounds **13**.^20^ This was followed, if desired, by nucleophilic substitution with an alkyl halide **14** using Cs_2_CO_3_ to yield *N*-alkylated compounds **15** (**B**, Scheme 1B).

### Structure–Activity Relationships

In total, 147 compounds have been evaluated in this study. The most significant molecules—those that contribute most to an understanding of the structure–activity relationships (SARs)—are reported below, but all molecules are described in the Supporting Information - Biology and may additionally be found in the online project infrastructure (https://github.com/opensourceantibiotics/Series-2-Diarylimidazoles). Twenty-four compounds were donated by the Drugs for Neglected Diseases *initiative* (DND*i*), for which the experimental data have been published.^21^ An additional 36 compounds were contributed from Northeastern University, the experimental details for which may be found in a separate publication that has been recently published;^22^ the compound identities are delineated in the Supporting Information - Biology, Tables S1 and S2.

**Figure 2:**
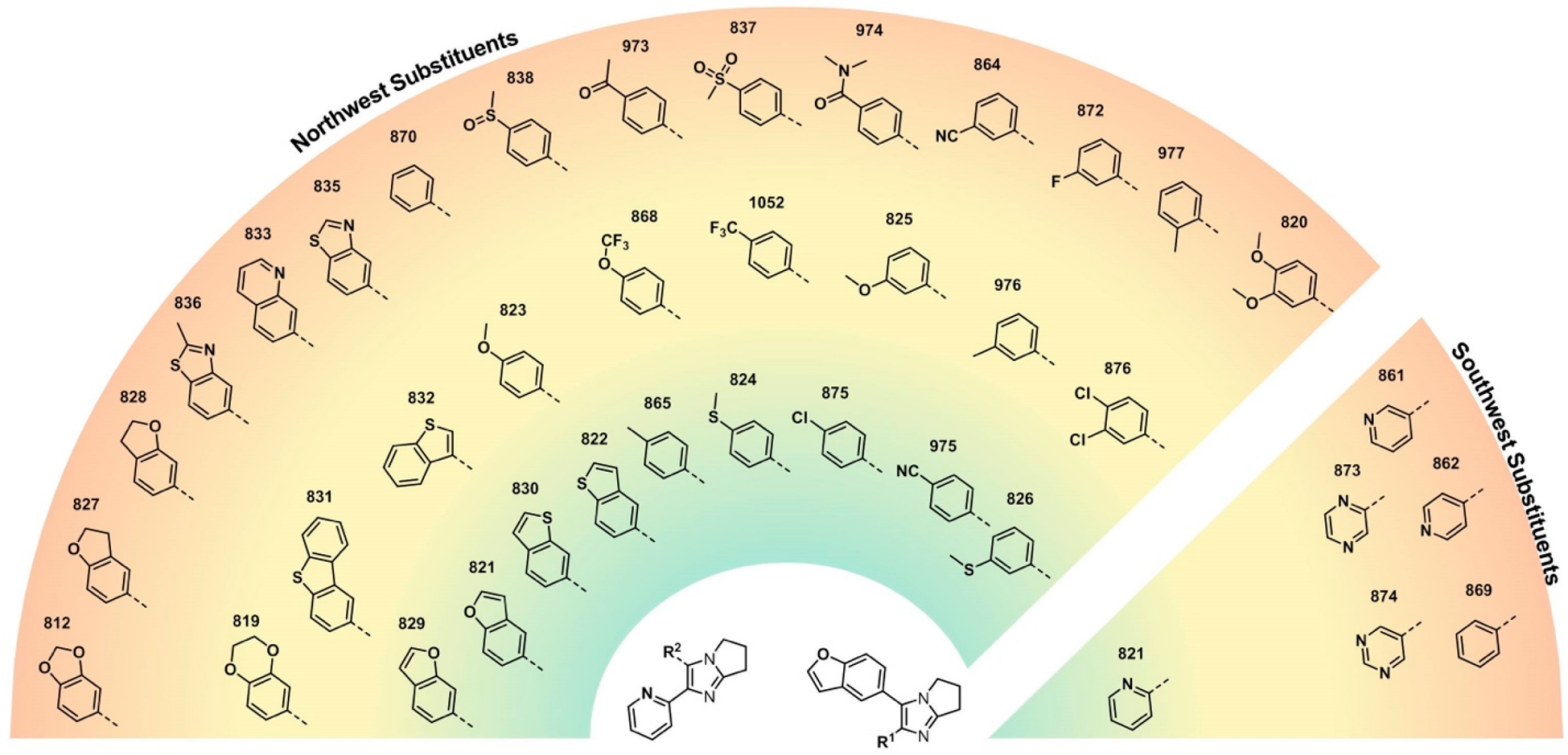
Heat map of *in vitro* potency against MRSA of analogues with variations in the northwest and southwest substituents. Color gradient: green <4 μg/mL; yellow <16 μg/mL; red = 32 μg/mL.

We began our SAR investigations by preparing a wide variety of analogues with variation in the northwest aryl substituent; the aim was to capitalize on the potency of the benzothiophene of **822** while reducing the potential metabolic liabilities of that motif.^23,24^ As shown in Figure 2, several fused bicycles were tolerated; of note is the “reverse” (6-substituted) thiophene **830** that was found to be almost equipotent to the original compound **822** (unlike the less potent 3-substituted analogue **832**) and the benzofuran pair **821** and **829** that were similarly potent. Compounds with bicyclic motifs containing saturated rings were found to be ineffective (e.g., **827**/**828**), as were aromatic nitrogen-containing fused bicycles (e.g., benzothiazoles **835** and **836**, and quinoline **833**).

We additionally explored a variety of simpler phenyl rings substituted at the northwest position. Replacement of the benzofuran moiety in **821** with *para*-tolyl (**865**, green region in Figure 2) maintained potency; other *para*-substituted phenyl rings (e.g., in **824**, **875**, and **975**) resulted in significantly improved activity against MRSA when compared to the analogue with an unsubstituted phenyl ring (**870**). Small alterations to the *para*-substituent significantly influenced potency (e.g., **824** vs **838**), though a group in this position generally performed better than the same group in the *meta* position (e.g., **865** vs **976**, **975** vs **864**). A focused library based on the promising compound **975** was synthesized and evaluated (Supporting Information - Biology, Table S2), but none of the compounds retained the potency of the parent. More oxidized substituents, such as acyl (**973**), methyl sulfone (**837**), or dimethylamide (**974**), were not tolerated (red region in Figure 2).

We rapidly established the essentiality of the southwest 2-pyridyl substituent: unlike compound **821**, pyridine isomers **861** and **862** were inactive, as were the unsubstituted phenyl compound (**869**) and the pyrazines **874** and **873**.

**Figure 3:**
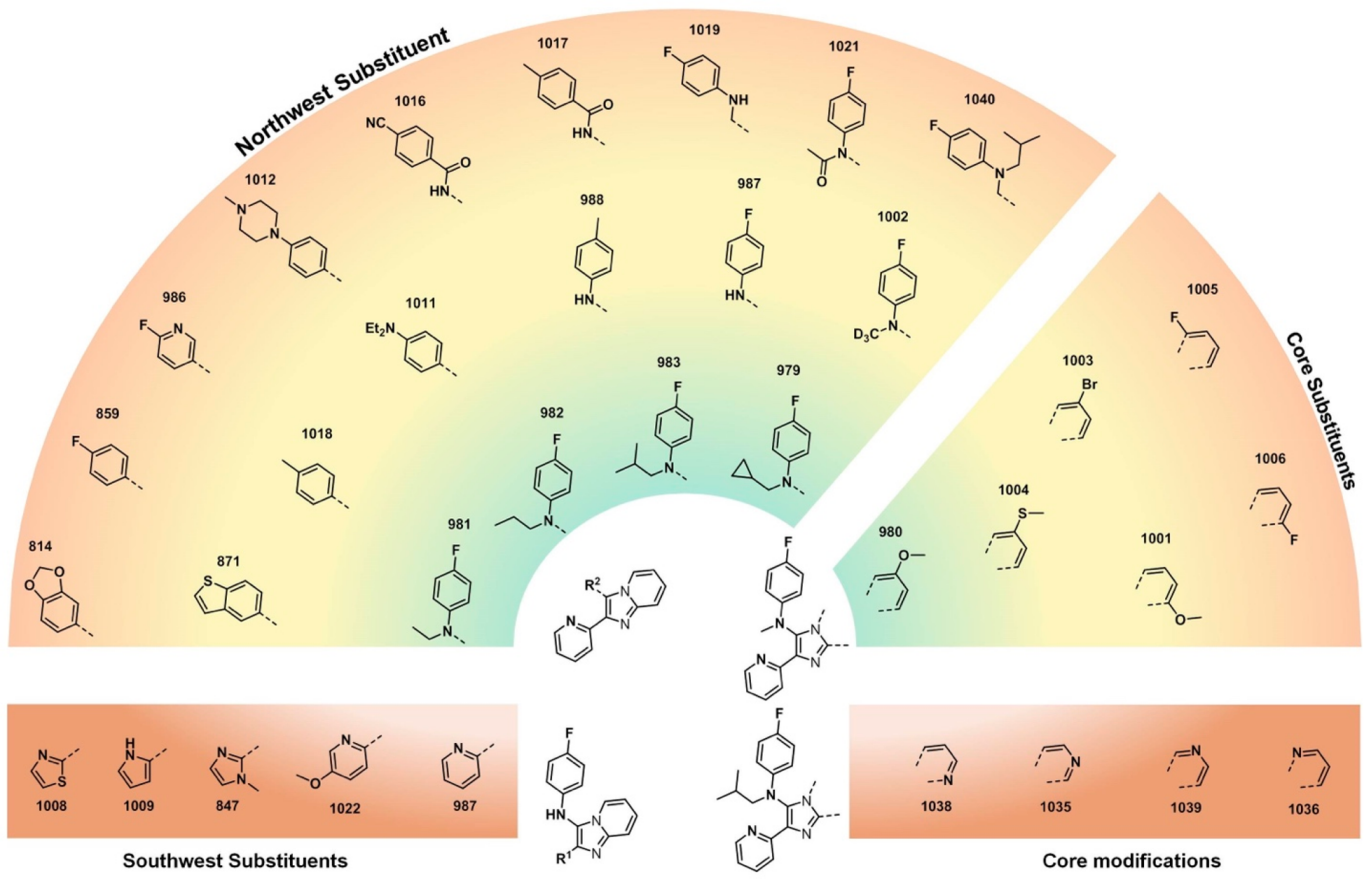
Heat map of *in vitro* potency against MRSA of analogues containing fused bicyclic cores. Color gradient: green <4 μg/mL; yellow <16 μg/mL; red = 32 μg/mL.

Our SAR investigations then focused on the evaluation of fused aromatic cores (Figure 3). Compounds **859** and **986**, containing a benzo-fused core and a northwest aromatic moiety, were inactive, but the potency could be mitigated by fine-tuning of the substituents (e.g., *p*-Me (**1018**) or *p*-(NEt_2_) (**1011**)) and, interestingly, the introduction of a nitrogen atom spacer in compound **987** that was seen as promising because of the introduction of an sp^3^ nitrogen providing an additional point of diversity and potentially better solubility. This compound was explored through variation of other coordinating rings in the southwest position (**1022**, **847**, **1008**, and **1009**) with no improvement in potency.

Acylation (**1021**) or homologation (**1019**) of the amine provided no benefit, but a significant improvement in potency was seen with alkylation of the nitrogen atom (**979**, **981**–**983**). While maintaining this motif, it was found that some substitutions were acceptable on the fused aromatic core (e.g., 6-methoxy in **980**) though with a high degree of sensitivity to such substitutions (e.g., lower potencies seen with structurally close isomers (**1001**) or analogues with other minor changes (**1003**–**1006**)). While it was hoped that the use of an aza-aromatic ring in the fused core might help with solubility, a nitrogen atom in any of the four available positions on that ring (i.e., **1035**, **1036**, **1038**, and **1039**) was deleterious to potency. An obvious final structure to explore, 1,2-disubstituted benzimidazole, was briefly explored but provided only inactive compounds (Supporting Information - Biology, Table S2).

Due to the lack of antibiotics for the treatment of multidrug-resistant enterococcal infections, we also tested selected compounds against vancomycin-resistant enterococcus (VRE; Supporting Information - Biology, Tables S1 and S2). Of these, **979** and **982** showed potent MICs (MIC = 4 μg/mL) against VRE.

From the above campaign that aimed to optimize the potency of this series against MRSA, 147 heterocyclic compounds were synthesized and tested, of which 88 were inactive (MIC > 32 μg/mL), 11 were moderately active (MIC = 16 μg/mL), and 43 displayed promising potency (MIC ≤ 8 μg/mL).

### ADME and Pharmacokinetics

**Figure 4:**
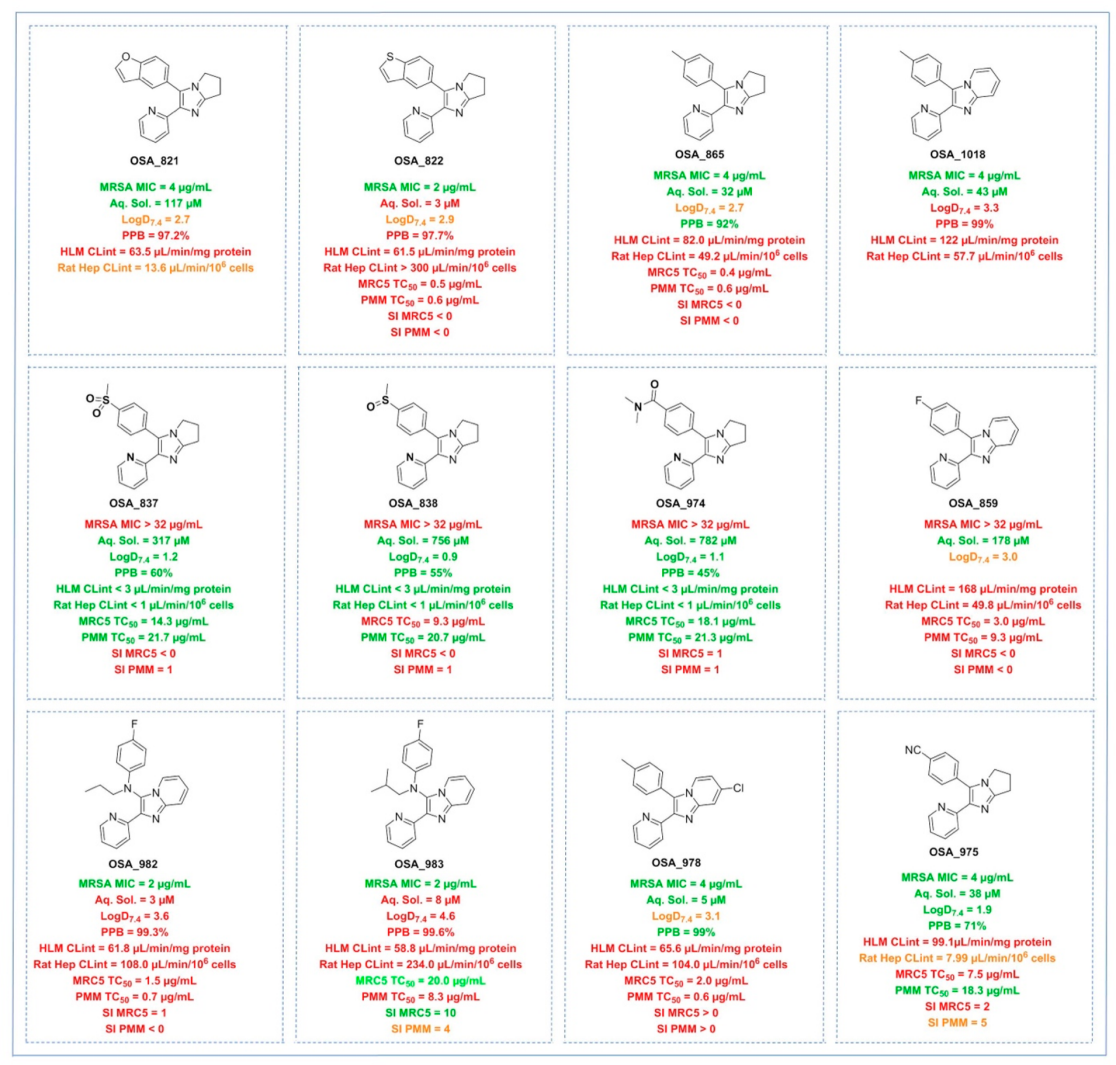
Human and rat liver microsome and hepatocyte intrinsic clearance data and mammalian cell line toxicity results. MRSA MIC data units are μg/mL. Selectivity index (SI) = ratio of cytotoxic TC_50_ to MRSA MIC.

To guide our initial SAR exploration work, we obtained liver microsomal intrinsic clearance data for 5 of the 18 compounds assayed by CO-ADD (Supporting Information - CDCO Report and Supporting Information - Biology). We then acquired intrinsic clearance (CL_int_), plasma protein binding (PPB), and solubility data on further compounds throughout the SAR campaign (Supporting Information - Biology, Tables S3 and S4). Our target was to identify compounds with promising characteristics for lead development: MIC < 4 μg/mL, thermodynamic aqueous solubility ≥ 10 μM, Chrom Log *D*_7.4_ values < 2, human liver CL_int_ < 9.0 μL/min/mg protein, rat hepatocyte CL_int_ < 5 μL/min/10^6^ cells, and human PPB ≤ 95%. The data for selected compounds (full data are available in the Supporting Information - Biology, Table S3 and S4) show that these compounds have moderate to high intrinsic clearance (Figure 4). The more polar compounds (lower LogD) had reduced PPB and were more metabolically stable. In general, potency was negatively correlated with polarity and solubility, in that the most potent compounds were poorly soluble.

Of the initially studied compounds, the potent benzofuran **821** performed better than the potent benzothiophene **822** (particularly in rat liver microsomes), though it is also known that benzofuran-containing compounds can undergo metabolic activation at the 2-position,^24^ which had made replacement of this functionality a priority for series development. We also confirmed potential sites of metabolism using SMARTCyp (https://smartcyp.sund.ku.dk/mol_to_som; see Supporting Information - Biology, Figure S3).

**Figure 5:**
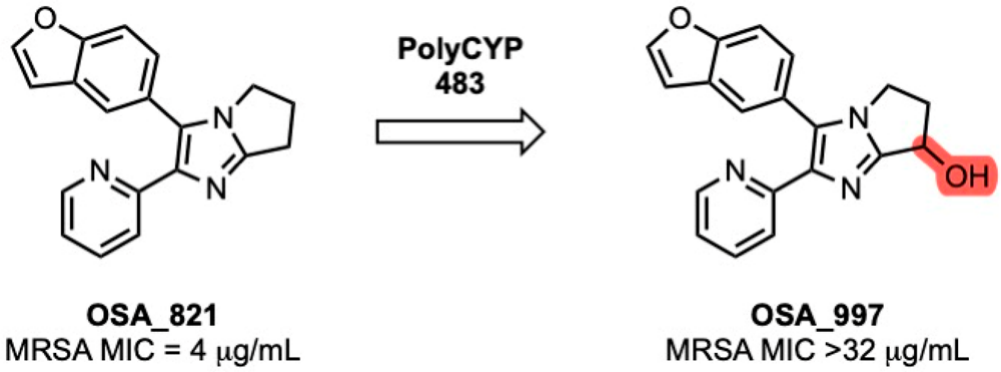
Structure and MRSA activity of a metabolite of **821**.

To probe experimentally the cause of any CYP-mediated metabolic liability in the promising benzofuran, this compound was screened with a PolyCYPs enzyme panel kit (provided by Hypha Discovery) that identified one main monohydroxylated metabolite. This matched the monohydroxylated metabolite observed in human liver S9 incubations in the presence of the NADPH cofactor-generating system; human liver S9 incubations performed in the presence of the UDP-GA cofactor resulted in samples containing a directly conjugated glucuronide metabolite, the structure of which was not determined (Supporting Information - Biology). Following a scale-up reaction with one of the PolyCYP enzymes, it was possible to identify the main hydroxylated metabolite (**997**, Figure 5) resulting from oxidation on the saturated core at the 7-position (Supporting Information - Chemistry). This metabolite was found to be inactive when evaluated vs MRSA. The metabolic liability identified for the benzofuran may not be responsible for the intrinsic clearance data obtained, since compounds with such a motif displayed intrinsic clearance similar to that of analogous compounds with a fully aromatic core (e.g., **865** vs **1018**).

Several compounds displayed low microsomal intrinsic clearance (e.g., HLM CL_int_ < 3 μg/min/mg protein for **837**, **838**, and **974**) in tandem with good solubility, but these compounds were not potent. The fully aromatic (imidazopyridine **1018**) vs partially aromatic (diarylimidazole **865**) cores performed approximately equivalently. The introduction of the nitrogen linker between rings did not have the expected impact on compound solubility (e.g., **859** vs **982**, **982** and **983**).

To judge the selectivity of this series for MRSA vs other cells, the toxicity of key compounds was evaluated against MRC-5_SV2_ (human lung fibroblast cell line) and PMM (primary mouse macrophages) and the selectivity index (SI) calculated (where SI = ratio of cytotoxic TC_50_ and MRSA MIC; additional data can be found in Supporting Information - Biology, Table S5). Most of the inactive compounds exhibited low toxicity to either cell line. The imidazopyridine **983** showed a good selectivity index (SI = 10) for the MRC5 cell line, but this was accompanied by poor ADME properties. The best overall balance of parameters in these systems was found with **975**, a potent compound with reasonable solubility and low intrinsic clearance in rat hepatocytes, with low toxicity against MRC5 cells and PMM.

**Figure 6:**
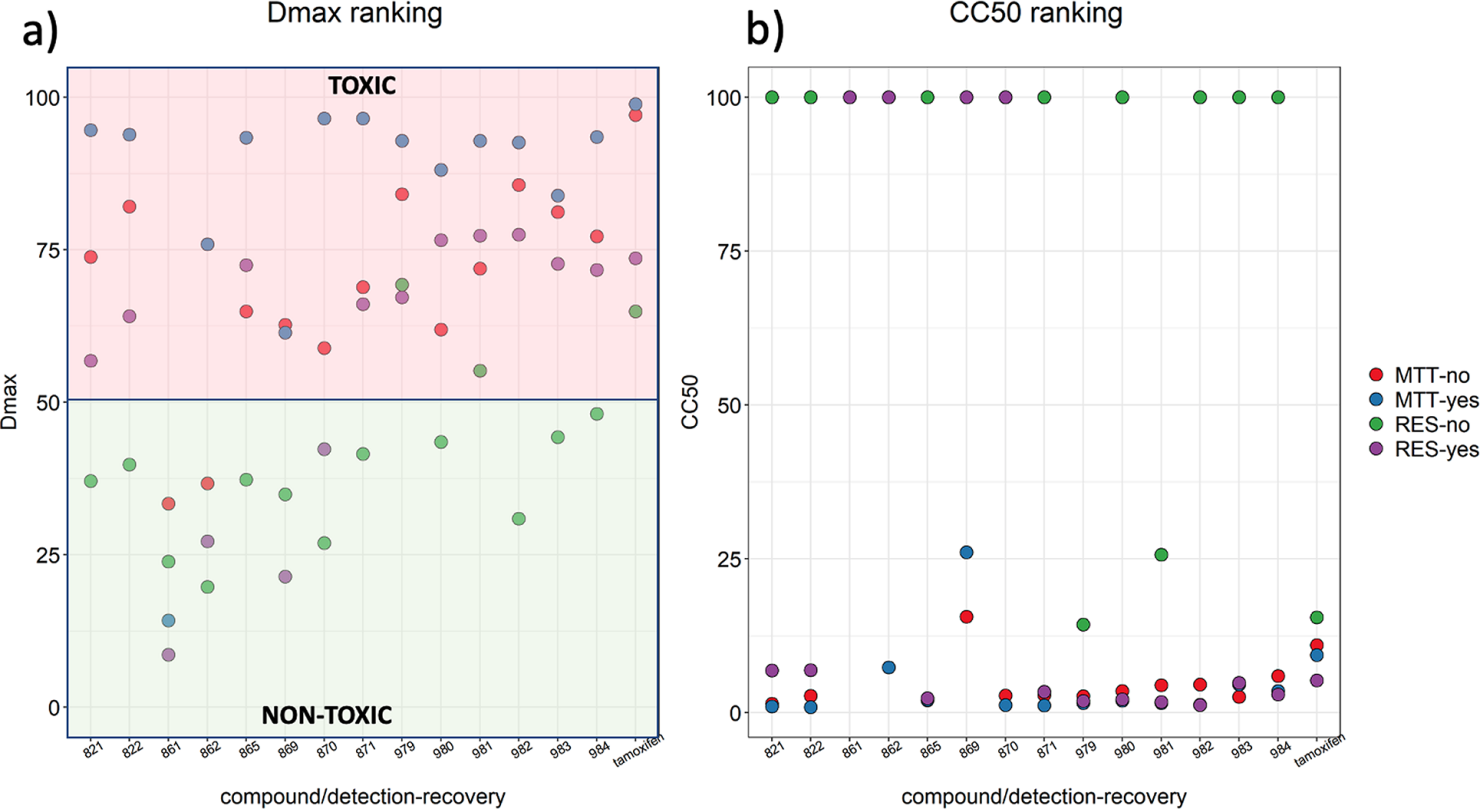
(a) *D*_max_ classification: compound toxicity at highest concentration is mapped against recovery status and detection output; compounds where *D*_max_ is lower than 50% are deemed non-toxic. (b) CC_50_ classification: concentrations resulting in 50% reduction in viability were calculated by fitting logistic curves to viability data.

The importance of balancing potency vs MRSA and cytotoxicity led us to examine a subset of compounds more thoroughly for toxicity against human embryonic kidney cell line HEK293. We measure toxicity through either CC_50_ (concentration of compound inducing 50% reduction in viability) or *D*_max_ as defined by CO-ADD^25^ and calculated following exposure of cells to compounds and recording of metabolic activity via either tetrazolium dye absorbance or resorufin fluorescence (methods are described in Supporting Information - Biology). *D*_max_ is a parameter that quantifies toxicity at the highest tested concentration (32 μg/mL for all compounds), calculated as reciprocal percentage of viable cells (100 – viability%), where cells either were allowed to recover or viability was measured immediately after treatment. While some studies suggest that 3-[4,5-dimethylthiazol-2-yl]-2,5-diphenyltetrazolium bromide (MTT) is less sensitive than resorufin,^26^ others report overestimation of viability due to accumulation of resorufin in cells or the opposite due to extensive reduction of the dye by cells with high metabolic activity.^27^ Our efforts focused on trying to replicate the original toxicity experiments (by CO-ADD) with tamoxifen using a protocol modified for use with 96-well plates. Given the variability of protocols and results in the literature,^28^ it was important to compare with a standard protocol we have reliably used in our own laboratories.^29−33^ When both CC_50_ values and *D*_max_ were mapped against the other two experimental variables (i.e., recovery status after treatment (yes/no) and the detection output (MTT/resazurin)), the majority of compounds were found to be toxic, as classified by CO-ADD,^25^ with a small spread of the data for most variations of protocol used (Figure 6A). Truly “non-toxic” compounds might be expected to show *D*_max_ < 50% for all experiment variations, e.g., **861**. The *D*_max_ spread for the control compound (tamoxifen) across all experiments was small and in agreement with previously measured values (CC_50_ = 9 ± 2 μg/mL).^25^ A small difference in *D*_max_ irrespective of whether cells were allowed to recover suggests those compounds are cytostatic or induce a partial response through a mechanism of toxicity which results in slowed cell growth or cause cell death through mechanisms the cells can resist,^28,34^ for example, compounds **821**, **822**, **869**, **979**, **982**, **983**, and **984**. A large spread of *D*_max_ values comparing recovery and non-recovery conditions larger than 20% is perhaps indicative of a mechanism of toxicity that persists beyond exposure, i.e., cytotoxic compounds that continue to induce toxicity as cells recover and restart dividing, for example, compounds **865**, **870**, **871**, **980**, and **981**. CC_50_ values reflect these classifications (Figure 6B), with **861**, **862**, and **869** being least toxic compared to tamoxifen (CC_50_ = 5.3–15.5 μg/mL), while other compounds exhibit CC_50_ values associated with higher toxicity (1–6 μg/mL). Where resorufin fluorescence was the measured output, data are more variable (e.g., **862** was found to be non-toxic by resazurin assay as well as with MTT after recovery, but toxicity more than 50% was measured in the acute exposure by MTT). This variability could be driven by interference of the compounds with the fluorescence readout or resorufin build-up as previously described.^27^ Another limitation to consider is the solubility of the compounds; while all were tested at concentrations ranging from 32 μg/mL (approximately 100 μM for a 300 Da compound) to 0.00625 μg/mL (∼0.19 μM) with a dilution factor of 2, all compounds screened against HEK293 except for **821**, **861**, and **870** have aqueous solubility less than 100 μM. Consequently, compounds precipitating during the assay or unpredictable interactions with serum in the media (and as a result interaction with the cells and their transport in the cell) may explain the spread of the data shown, in particular for those with very low aqueous solubility, such as **983**.

An attempt to characterize the mechanism of toxicity led to testing compounds’ interactions with DNA molecular beacons, which are short strands of DNA tagged with a donor–acceptor pair of fluorophores. The proximity between the fluorophores when the DNA strand is correctly folded results in no fluorescence from the donor; should a compound interfere with DNA folding, a change in the melting temperature will be observed. No compounds tested showed reduction in DNA melting temperature (Supporting Information - Biology, Figure S5), meaning the compounds did not interfere with DNA folding. By comparison, doxorubicin showed a dose–response behavior and is a known DNA intercalator.^35^

### Kinase Target Selectivity

**Figure 7:**
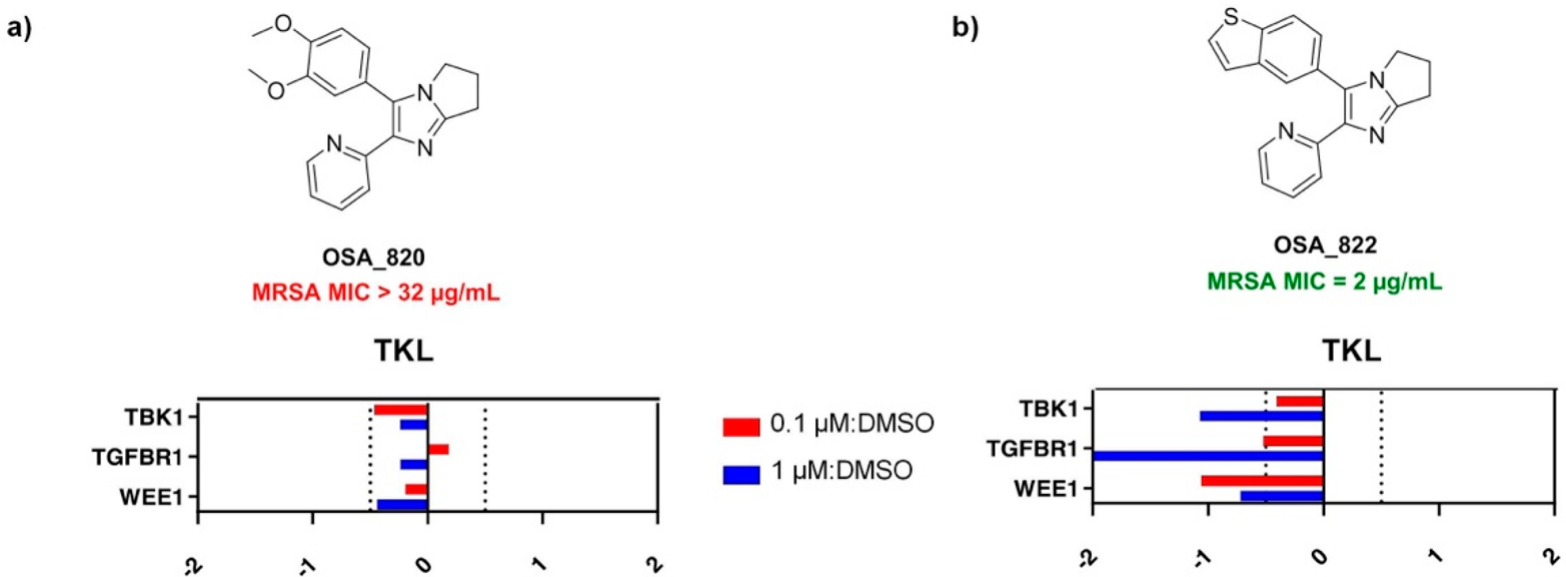
MIBs competition assay reveals potential kinase selectivity in mammalian cells. Shown is a subset of the kinome competition results from the incubation of HEK293 cell lysate with compounds **822** (active) and **820** (inactive). Following a brief incubation with these compounds, lysates were passed over MIBs (see Supporting Information - Biology), and the captured kinases were identified and quantified by mass spectrometry. Displaced kinases are shown to the left of the center line at the designated doses.

To understand the mammalian toxicity of these diarylimidazole derivatives and to aid in future compound design, we experimentally investigated the potential series’ mechanism of action (MoA). In the absence of a clear position where we might be able to attach a linker that would permit a pull-down experiment, and because these structures were originally based on known kinase inhibitors, a representative active (**822**) and inactive (**820**) compound were profiled using the multiplexed inhibitor beads with mass spectroscopic detection (MIB-MS) proteomic technique.^36−38^ This affinity composition assay has proven to be a useful approach to validate kinase inhibitor selectivity. The results from applying this method to HEK293 cell lysates suggested that TGF-β1 could potentially be a mammalian target for this series of compounds, as with the active compound both kinases were selectively competed off MIBs (1 μM), whereas similar results were not observed with the inactive compound (Figure 7; see Supporting Information - Biology for full data). Whether the inhibition of TGF-β1 contributes to the cytotoxicity observed remains to be determined.

Further support for TGF-β1 being a mammalian target comes from a report of compounds similar in structure to **822** bound to this protein (PDB: 3TZM and 1RW8)^39,40^ and the original GSK work on a precursor to this series mentioned above.

## Conclusions and Next Steps

Building on publicly available data derived from a CO-ADD screen, we investigated the promising activity against MRSA of a set of diarylimidazoles. Through synthetic variation of the core structure, we have identified simple molecules with good potency, good solubility, and low metabolic clearance; the identification of a metabolite was made possible by a private sector contribution to this open research project. Potency tended to correlate with toxicity to mammalian cells, and through experimental investigation of the series’ MoA, we have identified TGF-β1 as the potential mammalian target. Two key unanswered questions could be pursued next for this series: (1) confirmation of the MoA (both in MRSA and in mammalian cell lines to explain toxicity) using pull-down experiments with modified versions of the compounds, a strategy requiring the addition of a tether to the structure which, based on the SAR obtained to date, could be installed in place of the methoxy group in **980**, and (2) further analogue synthesis rounds based on the most attractive current compound, **975**. We thank the reviewers of this manuscript for additional suggestions for future work, including (i) modification of **821** to include “soft methyls” on the furan ring to slow clearance; (ii) assessment of the role of other clearance mechanisms such as via aldehyde oxidase; (iii) synthesis of the *N*-oxide of **975**; (iv) exploration of a combination of the original core (dihydro-5*H*-pyrrolo[1,2-*a*]imidazole) with the N-substituted aniline pendants used with the parallel imidazo[1,2-*a*]pyridine core; (v) determination of whether there is sufficient homology between the kinases implicated in this work as potential explanations for toxicity and bacterial kinases that might be involved in the MoA. Continuation of this research along these lines is made straightforward for the community by the availability of all data and discussions,^17^ and a platform for collaboration, on the Open Source Antibiotics infrastructure.

## Materials and Methods

### Synthesis

Details of the chemical syntheses and ^1^H and ^13^C NMR spectra for all compounds can be found in the Supporting Information - Chemistry. Reagents were purchased from Sigma-Aldrich, Alfa Aesar, Acros, Merck, Fisher Scientific, or Fluorochem. Unless otherwise specified, the reagents were used without further purification. Anhydrous solvents were obtained by drying over activated 3 Å molecular sieves. Argon gas was used as acquired. Reduced pressure means under rotary evaporation at 40 °C from 900 to 50 mbar. Purification of intermediates and final compounds was performed using silica gel or reversed-phase chromatography using the Biotage Selekt flash purification system. Analytical thin-layer chromatography was performed on Merck Silica Gel 60 F 254 precoated aluminum plates (0.2 mm) and visualized with UV irradiation (254 nm). High-temperature reactions were carried out in silicone oil baths or DrySyn blocks, controlled by a temperature probe. Analytical liquid chromatography/mass spectrometry (LCMS) was performed on an Agilent Infinity 1290 II system consisting of a quaternary pump (G7111A) and a diode array detector WR (G7115A) coupled to an InfinityLab LC/MSD (G6125B) using electrospray ionization (ESI). An Agilent Poroshell 120 EC-C18 column (2.7 μm, 4.6 mm × 50 mm) was eluted at a flow rate of 1.5 mL/min with a mobile phase of 0.05% formic acid in H_2_O and 0.05% formic acid in MeCN. All compounds tested had a purity of >95% as measured by LCMS, unless otherwise noted. Melting points (mp) were recorded on a Stanford Research Systems OptiMelt at 1 °C min (capillaries o.d. = 1.5–1.6 mm, 90 mm). Nuclear magnetic resonance spectroscopy was carried out at 300 K on Bruker spectrometers: either AVANCE III 400 (^1^H at 400 MHz, ^13^C at 101 MHz) or AVANCE III 500 (^1^H at 500 MHz, ^13^C at 126 MHz). Chemical shifts (δ, ppm) are reported relative to the solvent peak (CDCl_3_: 7.26 [^1^H]; DMSO-*d*_6_: 2.50 [^1^H]; CD_3_OD: 3.31 [^1^H]; acetone-*d*_6_: 2.05 [^1^H]; or acetic acid-*d*_4_: 2.04 [^1^H]). ^1^H signal multiplicity is reported as singlet (s), doublet (d), triplet (t), quartet (q), pentet (p), and combinations thereof, or multiplet (m). Broad signals are designated broad (br). Coupling constants (*J*) are reported in Hertz (Hz). Integrals are relative. app = apparent when the multiplicity was unexpected, e.g., coincidental or unresolved. High-resolution mass spectrometry (HRMS) was performed on a Bruker 7T FT-ICR or an Agilent 6545XT AdvanceBio LC/Q-TOF using ESI. Positive and negative detection is indicated by the charge of the ion; e.g., [M+H]^+^ indicates positive ion detection.

### Method for Antimicrobial Screening, Cytotoxicity and Hemolysis (CO-ADD)

The procedure employed by CO-ADD is provided as a Supporting Information file with this publication.

### *In Vitro* Antibacterial Activity (UCL School of Pharmacy Assay)

*Staphylococcus aureus* NCTC 13373 (*mecA*-positive; methicillin-resistant, NCTC 13373) was supplied by the National Collection of Type Cultures (NCTC), Public Health England, United Kingdom. Other organisms, *S. aureus* ATCC 25923 (methicillin-susceptible) and *Enterococcus faecalis* ATCC 51299 (*vanA*-positive; vancomycin-resistant), were obtained from the American Type Culture Collection (ATCC) via LGC Standards, UK. A suspension of each organism was prepared from an overnight plate culture (tryptone soya agar) in phosphate-buffered saline (PBS) and adjusted to an absorbance reading at 600 nm of 0.1, which is approximately equivalent to 1 × 10^8^ colony-forming units (CFU) per mL. The cultures were further diluted 1:100 in isosensitest broth (Oxoid, United Kingdom) before 100 μL was used to inoculate each well of a 96-well plate that contained diluted samples of the OSA series. Compounds were evaluated over a 2-fold dilution range from 32 to 0.03 μg/mL. Stocks of the samples were prepared in 100% DMSO at 3.2 equiv before being diluted in isosenstitest broth. The highest concentration of DMSO present was 2%, which has been previously shown not to inhibit the growth of the microorganisms (data not shown). Inoculated plates (total volume of 200 μL containing 5 × 10^5^ CFU/mL) were incubated at 37 °C for 16 h, with the MIC being recorded as the lowest concentration where growth was not visible. Vancomycin was used as a quality control compound.

### *In Vitro* ADME (Centre for Drug Candidate Optimization, Monash Institute of Pharmaceutical Sciences)

#### Kinetic Solubility Estimation Using Nephelometry

Compound in DMSO was spiked into either pH 6.5 phosphate buffer or 0.01 M HCl (approximately pH 2.0) with the final DMSO concentration being 1%. After 30 min had elapsed, samples were then analyzed via nephelometry to determine a solubility range.^41^

#### Distribution Coefficient Estimation Using Chromatography

Partition coefficient values (LogD) of the test compounds were estimated at pH 7.4 by the correlation of their chromatographic retention properties against the characteristics of a series of standard compounds with known partition coefficient values. The method employed is a gradient HPLC-based derivation of the method developed by Lombardo.^42^

#### *In Vitro* Metabolic Stability

The metabolic stability assay was performed by incubating each test compound with a suspension of liver microsomes (0.4 mg/mL protein, 1 μM substrate, 37 °C). Microsomes were purchased from XenoTech. The metabolic reaction was initiated by the addition of an NADPH-regenerating system and quenched at various time points over a 60 min incubation by the addition of acetonitrile containing metolazone as an internal standard. Control samples (containing no NADPH) were included (quenched at 2, 30, and 60 min) to monitor for potential degradation in the absence of cofactor.

### Toxicity (UCL School of Pharmacy)

#### Cell Culture

HEK293 cells (ATCC, UK) at passage 32 were a kind gift from Dr. Ben Allsop from the Translational Research Office at University College London. Cells were tested for mycoplasma using a MycoAlert Detection Kit (Lonza, Switzerland) as per manufacturer instructions, frozen stocks were stored in liquid nitrogen, and cells were re-tested whenever a new aliquot was thawed.

Cells were maintained in DMEM (Sigma-Aldrich, UK) supplemented with 10% heat-inactivated FBS (Sigma-Aldrich, UK), 1% GlutaMax (ThermoFisher, UK), and 1% sodium pyruvate (Lonza, UK) without antibiotics in an incubator at 37 °C and 5% CO_2_. Media was changed every 2 days, and cells were passaged every 3–4 days at a subcultivation ratio of 1:10, by detaching the cells with phenol-free TrypLE Express (ThermoFisher, UK), after washing with HBSS Ca^2+^/Mg^2+^ (ThermoFisher, UK). Up to 55 passages were used throughout the experiments.

#### 96-Well Plate Dose–Response Studies

*(a) Cell seeding:* At subculture, a dilute solution of cells was counted using Trypan Blue (Sigma-Aldrich, UK) and a hemocytometer. Cells were further diluted to 7000 or 50 000 cells/mL for experiments.

**For recovery experiments**, 700 cells (100 μL) were seeded in 96-well plates and incubated for 48 h while cells reached exponential phase.

**For non-recovery experiments**, 5000 cells (100 μL) were seeded in 96-well plates and incubated for 48 h while cells reached confluence. This is the same number of cells as per the CO-ADD protocol where 384-well plates were used, but we extended the growth time to 48 h to produce a similar confluency.

The surface area ratio between 384- and 96-well plates is approximately 5–6. In 48 h, at a doubling time of 12–24 h, cell numbers would have increased 4–8 times.

Cells were seeded only in the 60 inner wells of the plate as per Supporting Information - Biology, Figure S4; sterile water was added to the rest of the wells.

For formazan absorbance detection, transparent, flat bottom, cell-culture treated 96-well plates (Sarsted, Germany) were used. For resorufin fluorescence measurements, cells were seeded in black plates with a cell-culture treated, flat, transparent bottom (Greiner Bio-One, UK).

*(b) Compound solutions preparation for assay:* Compounds were provided in 100% sterile DMSO (Bio-Techne, Germany) at a 3.2 mg/mL stock concentration.

Stock solutions were diluted 100 times (to 1% DMSO) to 32 μg/mL in full medium (vortexed briefly) and then serially diluted using a digital pipet (Explorer, Eppendorf, Germany) at a dilution factor of 2 to a final concentration of 0.00625 μg/mL.

*(c) Dosing experiments:* At the specified time after seeding, media was removed from cells, cells were washed with HBSS Ca^2+^/Mg^2+^ (100 μL), and 100 μL of diluted compounds were added as per Supporting Information - Biology, Figure S4, and incubated for 24 h.

After treatment, solutions were removed, and cells were washed with HBSS Ca^2+^/Mg^2+^. For non-recovery experiments, viability was determined immediately as described below.

For recovery experiments, fresh media was added to plates (100 μL) and cells were returned to the incubator for another 72 h, after which viability was recorded as described below.

*(d) Viability assays:* Viability was measured via either formazan absorbance or resorufin fluorescence.

i. MTT assay: 3-[4,5-Dimethylthiazol-2-yl]-2,5-diphenyltetrazolium bromide (MTT) (Sigma-Aldrich, UK) was dissolved in sterile PBS at 5 mg/mL with sonication (20 min), protected from light. Stock solution was filtered through a 0.22 μM PES membrane syringe filter and further diluted 1:10 with full medium. 100 μL of this solution was added to washed cells and incubated for 2 h. Then, it was removed either by blotting on tissue or by multichannel pipet taking care not to disturb the formazan crystals, which were then solubilized in 100 μL of DMSO with shaking on a plate for 15 min in the dark. Absorbance at 570 and 630 nm was read using a SpectroStar (BMG Labtech, Germany) plate reader.

ii. Resazurin assay: Deep Blue Cell Viability Kit (Biolegend, US) was diluted in phenol-free full medium by adding 10 μL of stock to 100 μL of medium (e.g., 500 μL to 5 mL). 110 μL of this solution was added to washed cells and incubated for 4 h. Fluorescence (excitation: 530–570 nm, emission: 590–620 nm) was read with either a SpectraMax M2e (Molecular Devices, US) or a HIDEX Sense (Hidex, Finland) plate reader.

*(e) Data processing:* Cell-free wells were used as background, which was subtracted from experimental wells. In plate, technical replicates were averaged, and % viability was calculated by ratio to negative control (untreated) average wells. Biological replicates (different plates) were plotted in Origin 2022B and fitted using logistic regression curves against concentration transformed on a log2 scale. Software returns the EC_50_ (concentration at 50% of observed response) if the fit is successful. CC_50_s (concentration causing 50% reduction in viability) were also calculated from curve fits if observed.

*(f) Quality control:* 1% in PBS Triton solution incubated with cells for 15 min prior to viability measurements was used as positive control. Negative controls were untreated wells.

Tamoxifen solutions prepared in the same way as compounds were used to provide external validity and consensus with the CO-ADD data.

### Procedure for DNA Binding Assay

A molecular beacon DNA sequence [FAM] CGT ATA TAT ATA TTT TTA TAT ATA TAC G [TAM] (Eurofin Genomics, Germany) labeled with fluorescein–tetramethylrhodamine donor–acceptor pair was dissolved in nuclease-free water at 100 μM, aliquoted, and kept frozen at −20 °C.

A working 2 μM DNA solution and compound dilution at 2 and 20 μM in hybridization buffer (10 mM Tris, 1 mM EDTA, 50 mM NaCl, 1.5 mM MgCl_2_ pH 8) was made. DNA was annealed by heating up to 95 °C on a heat block and allowing to cool down to room temperature.

#### PCR Plates Preparation

Solutions were pipetted in MicroAmp Optical 96-well plates (ThermoFisher, UK) such that DNA concentration was fixed (400 nM) and compounds concentration was 0, 0.1, 0.5, 1, 2,4 and 5 μM, in duplicate. Total well volume was set to 40 μL with nuclease-free water.

#### DNA Melt Curve Experiment

Measurements on the prepared plates were made on an Applied Biosystems 7500/7500 FastReal-Time PCR System (ThermoFisher, UK). A standard melt curve experiment was setup where temperature was ramped up from 15 to 90 °C at a 1% step gradient and fluorescence was monitored for filters 1 (FAM) and 3 (TAMRA). Fluorescence first derivatives against temperature were exported to Microsoft Excel and peak fitting was done using Origin 2022B.

### Plotting and Figures

Figures were drawn in BioRender (biorender.com), and summarized data was plotted in R 4.2.1 with RStudio.

### Mechanism of Action (UNC Chapel Hill)

HEK cell lysate was treated with DMSO and with two different concentrations (0.1 and 1 μM) of either ALMDAI26/OSA_820 or ALMDAI28/OSA_822 followed by affinity kinase enrichment using the MIB-MS. Differential abundance (increase or decrease in MIB binding) was determined for kinases identified. The mass spectrometry proteomics data have been deposited to the ProteomeXchange Consortium via the PRIDE^43^ partner repository with the dataset identifier PXD040208.

### Metabolite Biotransformation (Hypha Discovery)

#### Dose Escalation

A previous screening study highlighted many PolyCYPs enzymes as capable of producing OSA_000821 metabolites and that the best of these was PolyCYP483. OSA_000821 was in limited supply; therefore, a dose escalation step was completed prior to the scale up reaction to try and increase the parent:metabolite ratio. The reactions were performed in triplicate in a V-well 96-well polypropylene microtiter plate at 100 μL reaction volume. The reaction comprised 10 μL cofactor reagent stock solution (50 mM d-glucose-6-phosphate (G6P), 10 mM β-nicotinamide adenine dinucleotide phosphate (NADP^+^), 10 U/mL glucose-6-phosphate dehydrogenase (G6PDH), 5 mM MgCl_2_, and 100 mM potassium phosphate buffer at pH 8 dissolved in cold H_2_O), either 89.2 μL (for 200 and 300 mg/L doses) or 89.6 μL (for 50 and 100 mg/L doses) PolyCYP483 enzyme (from 500 μL stock prepared in cold H_2_O to give a final buffer concentration of 100 mM potassium phosphate and 5 mM MgCl_2_), and either 0.4 or 0.8 μL OSA_000821 (from 12.5, 25, 37.5 mg/mL stock in DMSO) to give final concentrations of 50, 100, 200, or 300 mg/L, respectively. Reactions were shaken at 200 rpm on a Kuhner (AG Switzerland) 5 cm orbital shaker at 27 °C for 18 h and stopped by the addition of an equal volume of MeCN.

#### Scaled-Up Reaction

A previously prepared fed-batch-derived cell pellet of recombinant *E. coli* strain expressing PolyCYP483 was thawed and re-suspended with 100 mM potassium phosphate buffer pH 8, containing 5 mM MgCl_2,_ 0.1% (v/v) tris(2-carboxyethyl)phosphine (TCEP), 10 mg/L phenylmethylsulfonyl fluoride (PMSF), and 4 U/mL benzonase. The cellular suspension was homogenized using a cell disruptor (1.1 kW system, Constant Systems Ltd., UK) set at 20 kpsi, then again at 24 kpsi and finally 30 kpsi. The homogenate was centrifuged at 47500*g*, and the crude extract (supernatant) was used for the reaction. A stock solution of OSA_000821 was prepared by dissolving in DMSO at 37.5 mg/mL. The final reaction comprised 137 mL crude extract, 1.23 mL OSA_000821, and 15.36 mL cofactor stock solution (50 mM G6P, 10 mM NADP^+^, and 10 U/mL G6PDH, all dissolved in 100 mM KPi buffer at pH 8 + 5 mM MgCl_2_) to provide a total volume of 153 mL. The bulk reaction was transferred in equal volumes to 3 × 250 mL Erlenmeyer flasks and incubated overnight at 27 °C and 180 rpm (5 cm diameter orbit). The reaction was checked by LCMS, and the whole reaction was harvested and frozen at −80 °C until ready for extraction.

#### Product Extraction

The reaction plus flask washings (170 mL) were defrosted, mixed well with an equal volume of MeCN, and centrifuged. Ammonium sulfate (50 g) was added to the supernatant and mixed well. The centrifuged pellet was re-suspended, extracted with 50% MeCN (aq), and re-centrifuged, and the extract was added to the supernatant–MeCN–ammonium sulfate mixture and all stored at −20 °C overnight. Post-freezing, the resulting MeCN layer was decanted to collect, and the residual aqueous re-extracted with another aliquot of MeCN (100 mL). Both MeCN layers were combined, dried to aqueous under vacuum, and lyophilized to provide an orange solid extract (Batch ID: CD88/99x/1).

#### Reaction Optimization and Purification

The dose escalation experiments for PolyCYP483 (Supporting Information - Biology, Table S6) showed that increasing the dose of OSA_000821 provided an increase in the production of metabolites. The PolyCYP reaction proceeded as expected. Supporting Information - Biology, Figures S7–S13, provide chromatograms and spectra of the extract and the metabolites detected therein.

Purification of OSA_000997 was performed on a Biotage Selekt instrument using automated reversed-phase column chromatography. The initial crude metabolite mixture was eluted with 0–100% MeOH in H_2_O in two batches, one at 6 mL/min on a Biotage Sfär C18 6 g column and another at 12 mL/min on a Biotage Sfär C18 12 g column. The fractions containing the desired metabolite (*m*/*z* 318) were combined and purified again by eluting with 0–100% MeOH in at 6 mL/min on a Biotage Sfär C18 6 g column. A final purification of the desired fractions was performed, eluting with 25–100% MeOH in H_2_O at 6 mL/min on a Biotage Sfär C18 6 g column to give the desired metabolite as a pale yellow powder (1.3 mg).

## Supporting Information Available

Biological Methods: SAR, *in vitro* antibacterial activity and toxicity tables, and metabolite biotransformation UPLC traces (PDF)
Chemical Methods: synthetic procedures, compound characterization, and ^1^H and ^13^C NMR spectra (PDF)
Table of OSA Series 2 molecules and associated biological data (XLSX)
Physicochemical and metabolic evaluation report from Monash University (PDF)
Methods for antimicrobial screening, cytotoxicity, and hemolysis from CO-ADD (PDF)
