# Supplementary material for: Open Source Antibiotics: Simple Diarylimidazoles Are Potent against Methicillin-Resistant Staphylococcus aureus
Source: ACS Infect Dis. 2023 Nov 22;9(12):2423–35. doi: 10.1021/acsinfecdis.3c00286 (PMC10714399; doi:10.1021/acsinfecdis.3c00286)
Supplement: Supplementary file 1 — id3c00286_si_001.pdf [file id3c00286_si_001.pdf]

Supporting Information

Biological Methods

Open Source Antibiotics - Simple Diarylimidazoles are Potent Against Methicillin Resistant Staphylococcus Aureus.

Dana M. Klug[1], Edwin G. Tse[1], Daniel G. Silva[1,2], Yafeng Cao[3], Susan A. Charman[4], Jyoti Chauhan[5], Elly Crighton[4], Maria Dichiara[5], Chris Drake[6], David Drewry[7,8], Flavio da Silva Emery[2], Lori Ferrins[5], Lee Graves[9], Emily Hopkins[6], Thomas A. C. Kresina[5], Álvaro Lorente-Macías[9,10,11], Benjamin Perry[12], Richard Phipps[6], Bruno Quiroga[5], Antonio Quotadamo[5,13], Giada N. Sabatino[1], Anthony Sama[14], Andreas Schätzlein[1], Quillon J. Simpson[5], Jonathan Steele[6], Julia Shanu-Wilson[6], Peter Sjö[12], Paul Stapleton[1], Christopher J. Swain[15], Alexandra Vaideanu[1], Huanxu Xie[3], William Zuercher[7], Matthew H. Todd[1,16*]

[1] School of Pharmacy, University College London, 29-39 Brunswick Square, London WC1N 1AX, United Kingdom.

[2] School of Pharmaceutical Sciences of Ribeirão Preto, University of São Paulo, Ribeirão Preto, São Paulo, 14040-903 Brazil.

[3] WuXi AppTec Company Ltd., 666 Gaoxin Road, East Lake High-Tech Development Zone, Wuhan 430075, People's Republic of China.

[4] Centre for Drug Candidate Optimisation, Monash Institute of Pharmaceutical Sciences, Monash University, Parkville, VIC 3052, Australia.

[5] Department of Chemistry and Chemical Biology, Northeastern University, Boston, Massachusetts 02115, United States of America.

[6] Hypha Discovery, 154b Brook Dr, Milton, Abingdon OX14 4SD, United Kingdom.

[7] UNC Lineberger Comprehensive Cancer Center, School of Medicine, University of North Carolina at Chapel Hill, Chapel Hill, NC, 27599, USA

[8] Structural Genomics Consortium, UNC Eshelman School of Pharmacy, University of North Carolina at Chapel Hill, Chapel Hill, NC, 27599, USA

[9] Department of Pharmacology, University of North Carolina at Chapel Hill, Chapel Hill, NC 27599, USA

[10] Department of Medicinal & Organic Chemistry and Excellence Research Unit of "Chemistry Applied to Biomedicine and the Environment", Faculty of Pharmacy, University of Granada, Campus de Cartuja s/n, 18071 Granada, Spain

[11] A. L-M. Present address: Cancer Research UK Edinburgh Centre, Institute of Genetics & Cancer, University of Edinburgh, Edinburgh EH4 2XR, United Kingdom

[12] Drugs for Neglected Diseases *initiative* (DND*i*), 15 Chemin Camille-Vidart, 1202 Geneva, Switzerland

[13] *Clinical and Experimental Medicine PhD Program, University of Modena and Reggio Emilia, 41121 Modena, Italy*

[14] Citizen scientist.

[15] Cambridge MedChem Consulting, 8 Mangers Lane, Duxford, Cambridge CB22 4RN, United Kingdom

[16] Structural Genomics Consortium, University College London, 29-39 Brunswick Square, London WC1N 1AX, United Kingdom

[*] *Corresponding author: matthew.todd@ucl.ac.uk*

Table of Contents

FIGURE S1. DISCOVERY OF ORIGINAL HITS....S3

FIGURE S2. ORIGINAL HIT DATA....S3

TABLE S1. *IN VITRO* POTENCIES AGAINST MRSA AND MSSA, AS WELL AS VRE AND VSE FOR ANALOGUES WITH VARIATIONS AT THE R1, R2 AND CORE FOR COMPOUNDS INCLUDED IN THE MANUSCRIPT S4

TABLE S2. *IN VITRO* POTENCIES AGAINST MRSA AND MSSA, AS WELL AS VRE AND VSE FOR ANALOGUES WITH VARIATIONS AT THE R1, R2 AND CORE FOR ADDITIONAL COMPOUNDS S14

TABLE S3. ADME AND PHARMACOKINETICS: COMPOUNDS THAT ARE INCLUDED IN THE MANUSCRIPT S22

TABLE S4. ADME AND PHARMACOKINETICS: COMPOUNDS ONLY APPEARING IN THE SUPPLEMENTARY INFORMATION S24

FIGURE S3. HIGHLIGHTED POTENTIAL SITES OF METABOLISM OF THE BENZOTHIOPHENE AND THE DIARYL PORTIONS OF OSA_822 ....S25

TABLE S5. MRSA ACTIVITIES AND CYTOTOXICITY EXPRESSED IN TC50 (µG/ML)....S25

FIGURE S4. GRAPHIC ILLUSTRATION OF PLATE SETUP FOR DOSING EXPERIMENTS....S27

FIGURE S5. MELTING TEMPERATURE OF CUSTOM DESIGNED MOLECULAR BEACONS WAS MEASURED TO ASSESS COMPOUND INTERACTION WITH DNA....S28

FIGURE S6: HEK CELL LYSATE MIB-MS EXPERIMENTS. S29

TABLE S6: RESULTS FOR THE DOSE ESCALATION EXPERIMENT OF CYP483 DOSED WITH OSA_000821....S30

FIGURE S7: CHROMATOGRAMS OF A REACTION EXTRACT FROM POLYCYP483 VS UCL-BT-A (OSA_000821)....S31

FIGURE S8: EXPANSION OF THE CHROMATOGRAMS ABOVE BETWEEN 0.95 AND 1.40 MINUTES S32

FIGURE S9: UV, POSITIVE ION AND NEGATIVE ION ESI MS SPECTRA OF THE METABOLITE ELUTING AT 1.03 MINUTES IN THE RESULTING EXTRACT FROM THE REACTION OF POLYCYP483 DOSED WITH UCL-BT-A (OSA_000821) S33

FIGURE S10: UV, POSITIVE ION AND NEGATIVE ION ESI MS SPECTRA OF THE METABOLITE ELUTING AT 1.06 MINUTES IN THE RESULTING EXTRACT FROM THE REACTION OF POLYCYP483 DOSED WITH UCL-BT-A (OSA_000821). S34

FIGURE S11: UV, POSITIVE ION AND NEGATIVE ION ESI MS SPECTRA OF THE METABOLITE ELUTING AT 1.28 MINUTES IN THE RESULTING EXTRACT FROM THE REACTION OF POLYCYP483 DOSED WITH UCL-BT-A (OSA_000821). S35

FIGURE S12: UV, POSITIVE ION AND NEGATIVE ION ESI MS SPECTRA OF THE METABOLITE ELUTING AT 1.34 MINUTES IN THE RESULTING EXTRACT FROM THE REACTION OF POLYCYP483 DOSED WITH UCL-BT-A (OSA_000821) S36

FIGURE S13: UV, POSITIVE ION AND NEGATIVE ION ESI MS SPECTRA OF THE METABOLITE ELUTING AT 1.36 MINUTES IN THE RESULTING EXTRACT FROM THE REACTION OF POLYCYP483 DOSED WITH UCL-BT-A (OSA_000821). S37

REFERENCES....S38

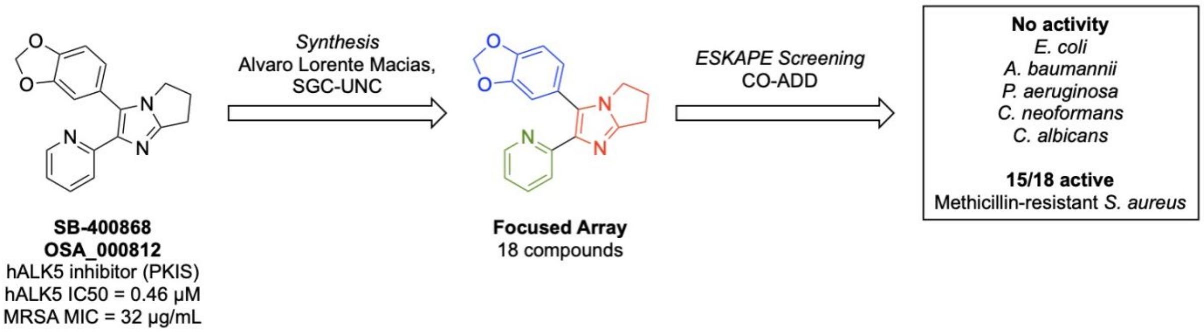

Figure S1. Discovery of Original Hits. SB-400868 (OSA ID = **OSA_812**) was identified as a hit and a focused array of 18 follow-up compounds were synthesized and tested for ESKAPE pathogen activity. Fifteen of 18 compounds were active against MRSA with MIC ≤ 32 μg/mL. Cytotoxicity in HEK293 cells was within 2-fold of 12 μg/mL and HC10 (concentration at which 10% hemolysis is observed) was >32 μg/mL for all compounds.

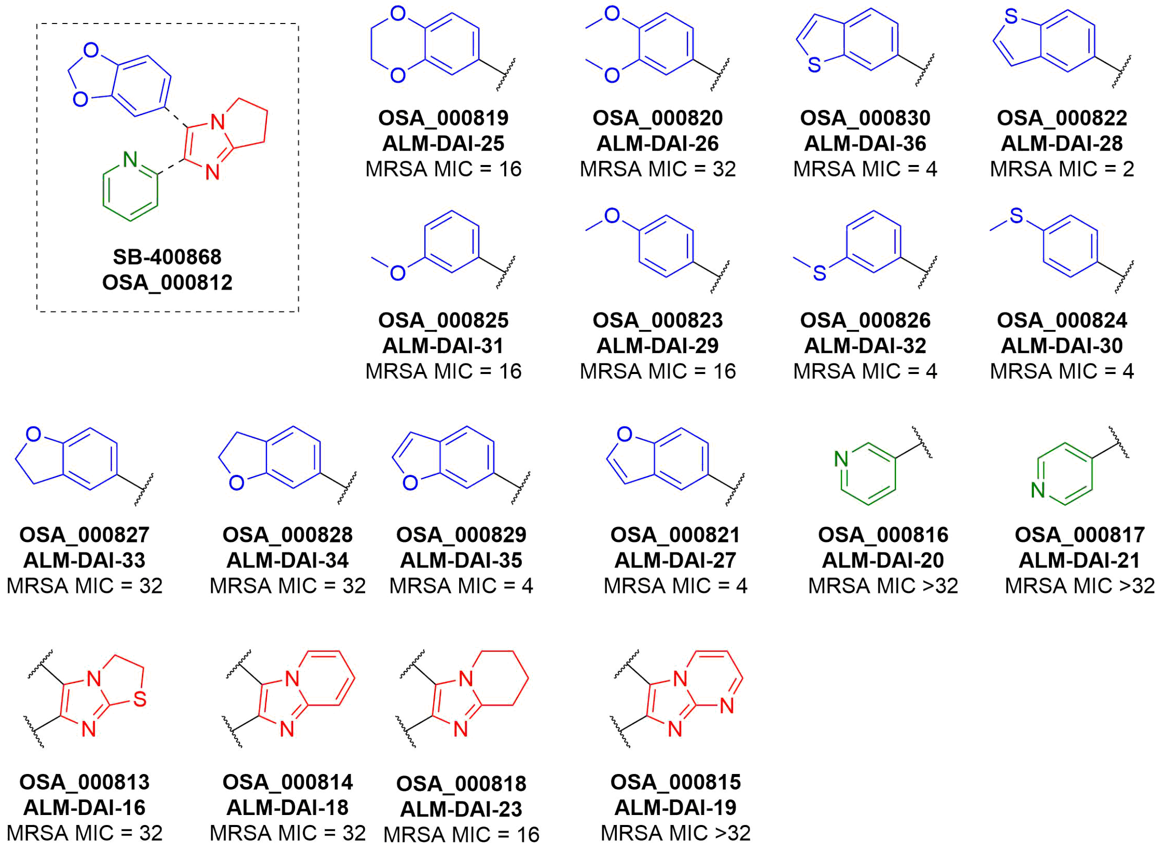

Figure S2. Original Hit Data. The MIC values (μg/mL) of the 18 compounds synthesized in the initial focused array are shown.

Table S1. *In vitro* potencies against MRSA and MSSA, as well as VRE and VSE for analogues with variations at the R1, R2 and core for compounds included in the manuscript.

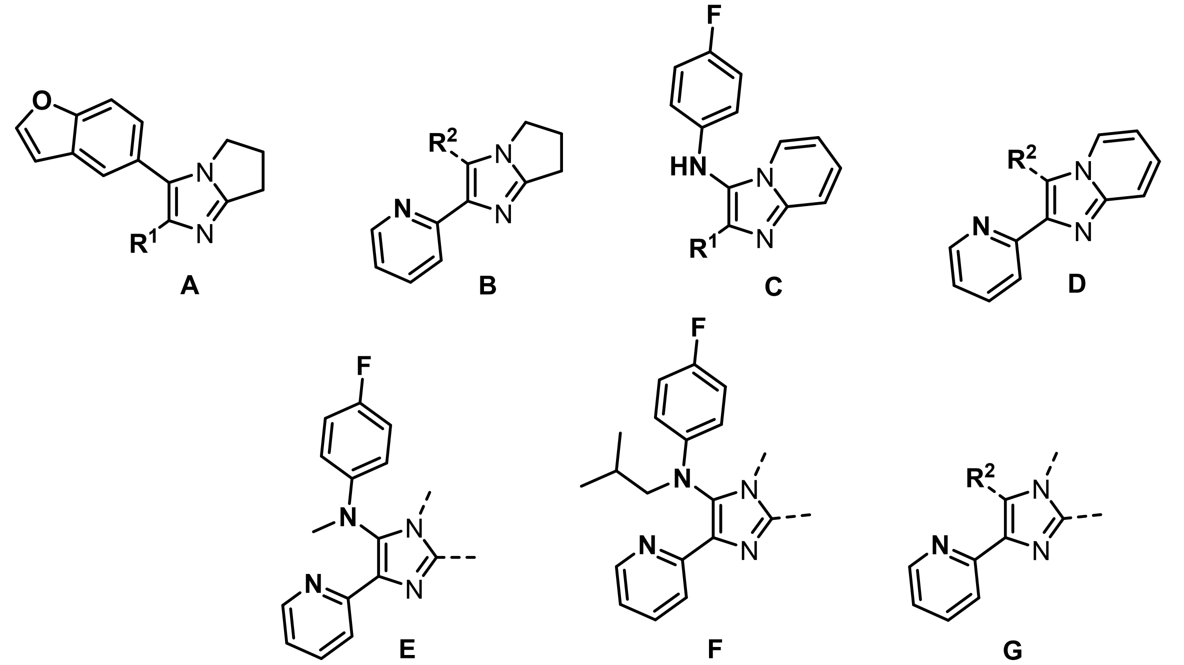

Cmpd	Structure	R^1	R^2	Core	MRSA MIC (µg/mL)	MSSA MIC (µg/mL)	VRE MIC (µg/mL)	VSE MIC (µg/mL)
**812**	B		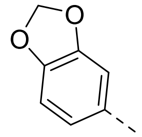		32			
**814**	D		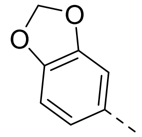		32			
**819**	B		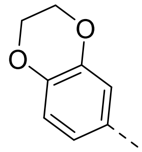		16			
**820**	B		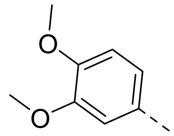		>32	>32		
**821**	A	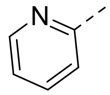			2		2	
**822**	B		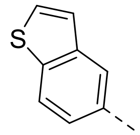		2	4	8	4

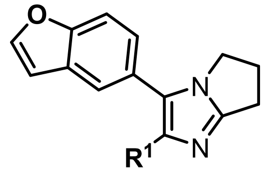

A

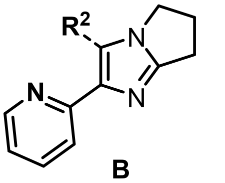

B

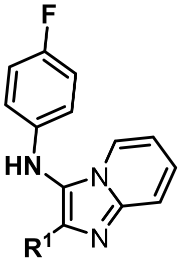

C

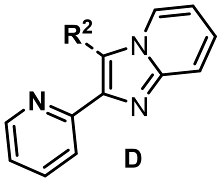

D

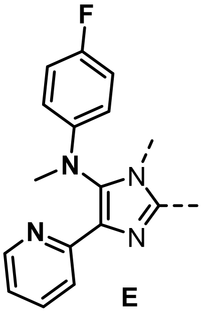

E

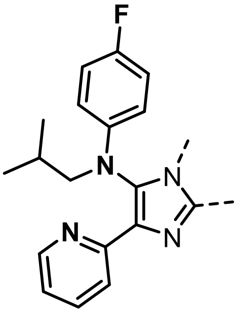

F

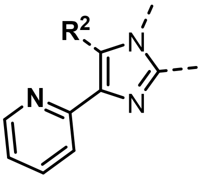

G

**Cmpd**	**Structure**	**R¹**	**R²**	**Core**	**MRSA MIC (µg/mL)**	**MSSA MIC (µg/mL)**	**VRE MIC (µg/mL)**	**VSE MIC (µg/mL)**
**823**	B		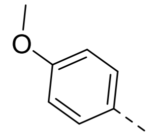		16			
**824**	B		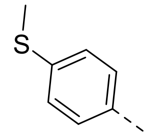		4			
**825**	B		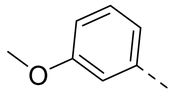		16			
**826**	B		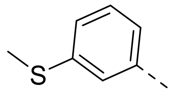		4			
**827**	B		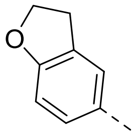		32			
**828**	B		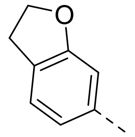		32			
**829**	B		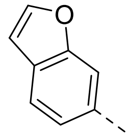		4			

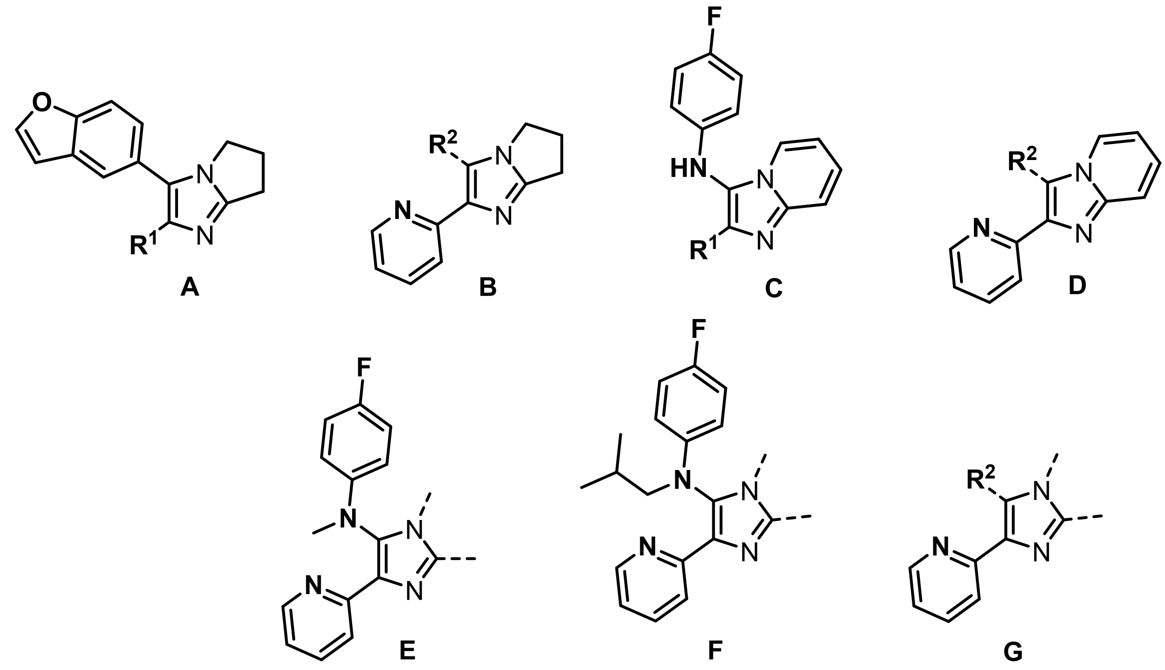

Cmpd	Structure	R1	R2	Core	MRSA MIC (µg/mL)	MSSA MIC (µg/mL)	VRE MIC (µg/mL)	VSE MIC (µg/mL)
**830**	B		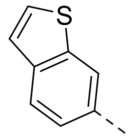		4			
**831**	B		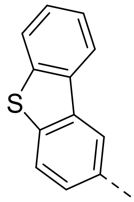		8			
**832**	B		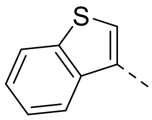		8			
**833**	B		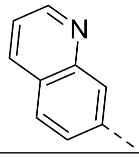		>32			
**835**	B		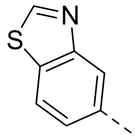		32			
**836**	B		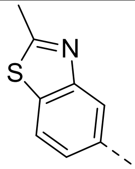		32			

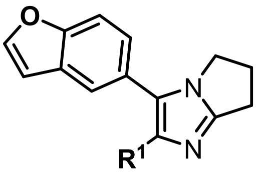
A

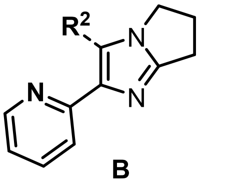
B

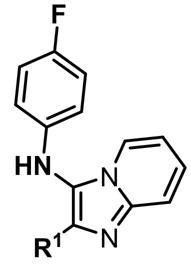
C

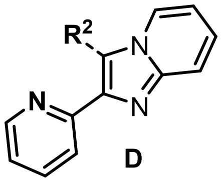
D

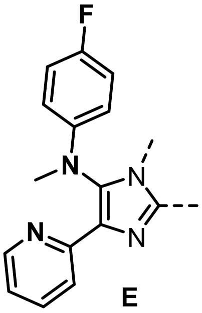
E

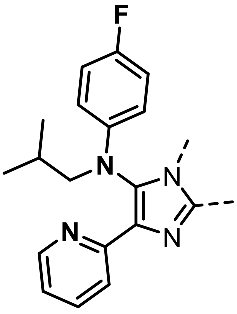
F

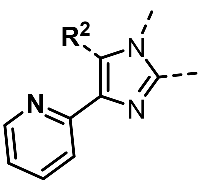
G

Cmpd	Structure	R^1	R^2	Core	MRSA MIC (µg/mL)	MSSA MIC (µg/mL)	VRE MIC (µg/mL)	VSE MIC (µg/mL)
**837**	B		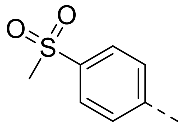		>32		>32	
**838**	B		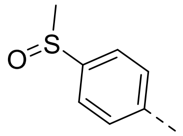		>32		>32	
**847**[a]	C	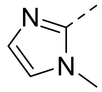			>32		>32	
**859** [a]	D		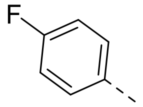		32		>32	
**861**	A	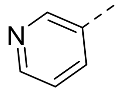			>32		>32	
**862**	A	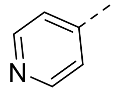			>32		>32	
**864**	B		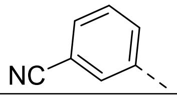		>32		>32	
**865**	B		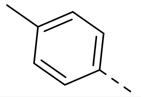		4	1	16	
**868**	B		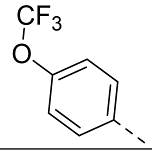		8		32	

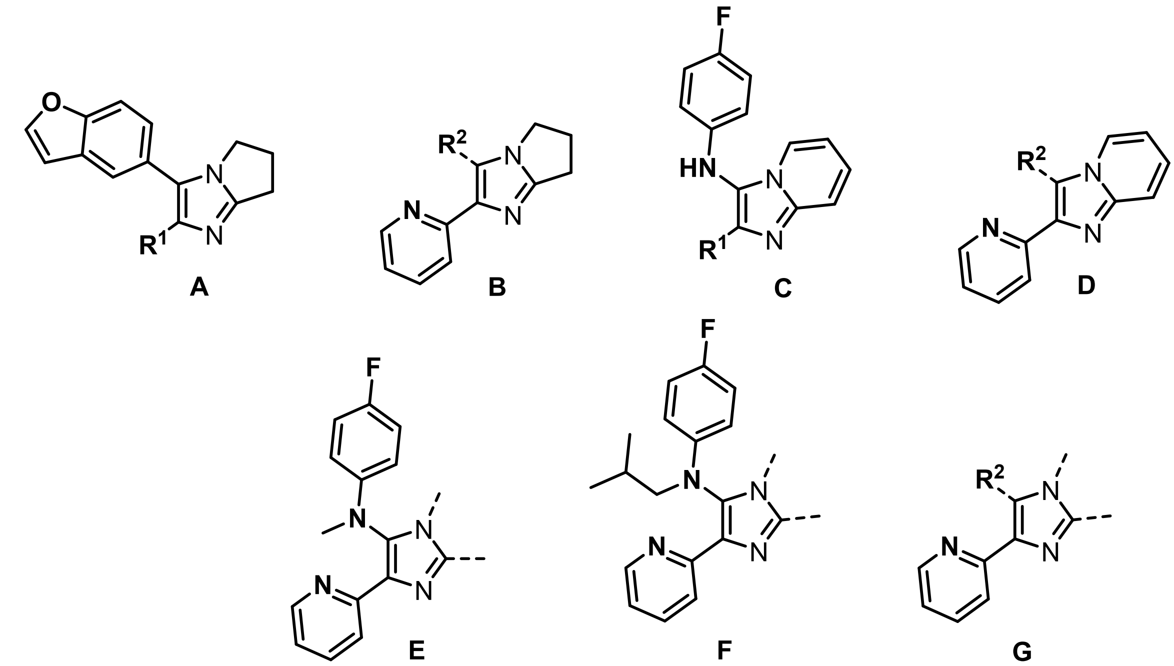

Cmpd	Structure	R¹	R²	Core	MRSA MIC (μg/mL)	MSSA MIC (μg/mL)	VRE MIC (μg/mL)	VSE MIC (μg/mL)
**869**	A	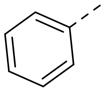			>32		>32	
**870**	B		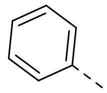		32		>32	
**871**	D		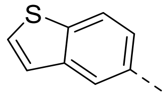		4		8	
**872**	B		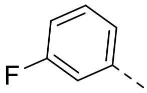		>32		>32	
**873**	A	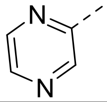			>32	>32	>32	
**874**	A	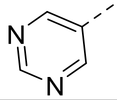			>32		>32	
**875**	B		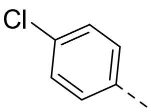		4		16	
**876**	B		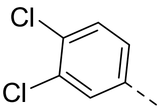		8		8	
**973**	B		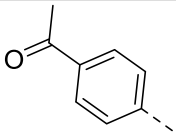		>32		>32	

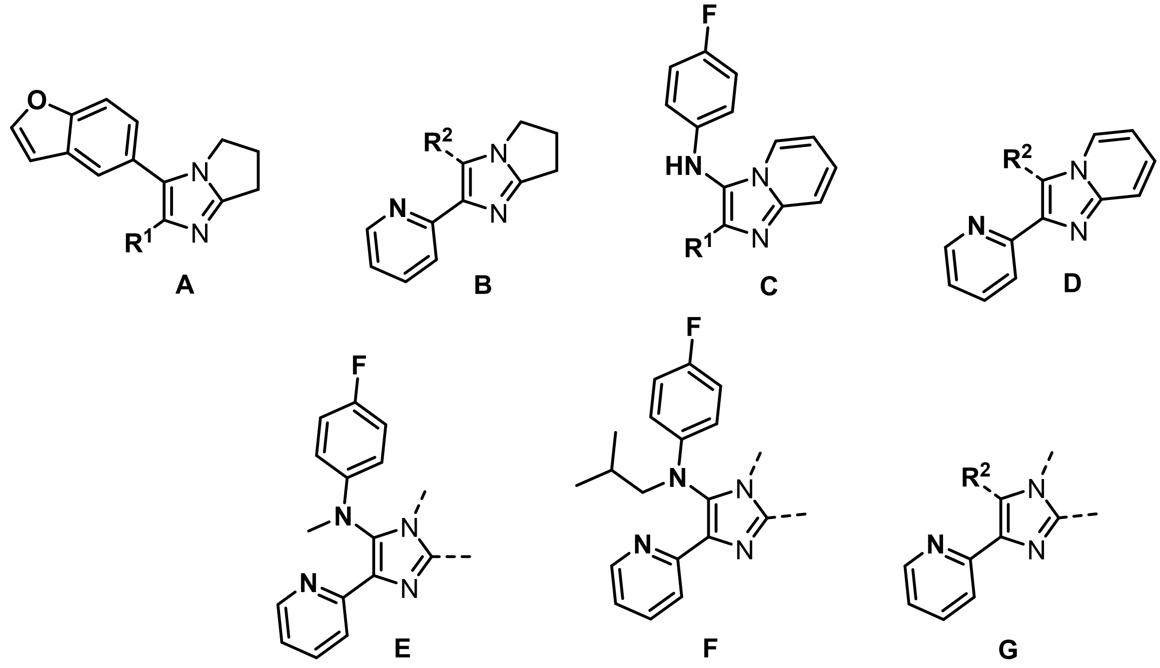

Cmpd	Structure	R¹	R²	Core	MRSA MIC (µg/mL)	MSSA MIC (µg/mL)	VRE MIC (µg/mL)	VSE MIC (µg/mL)
**974**	B		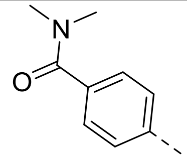		>32		>32	
**975**	B		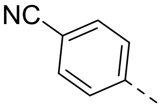		4		16	
**976**	B		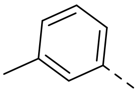		8		32	
**977**	B		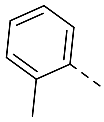		32		32	
**978**	D		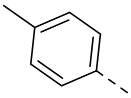	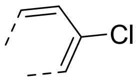	4		8	
**979**[b]	D		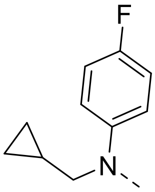		2	2	4	
**980**[b]	E			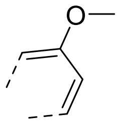	4	4	8	

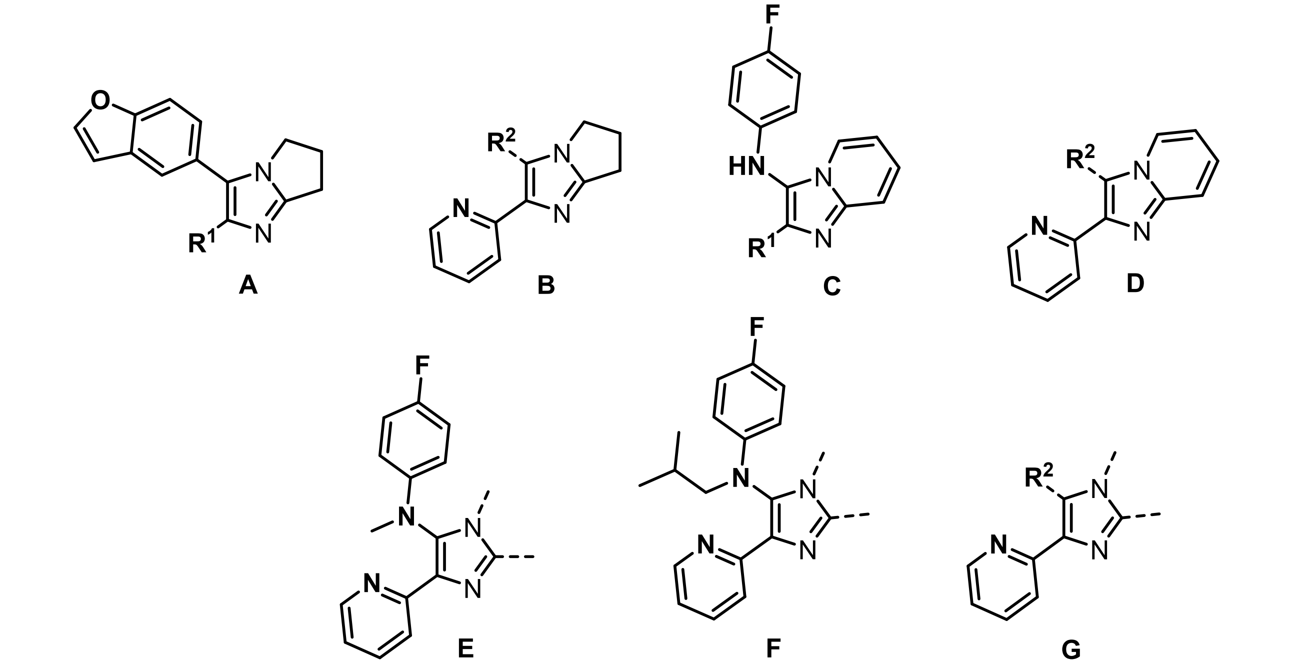

Cmpd	Structure	R[1]	R[2]	Core	MRSA MIC (μg/mL)	MSSA MIC (μg/mL)	VRE MIC (μg/mL)	VSE MIC (μg/mL)
**981**[b]	D		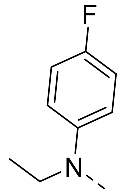		2	2	8	
**982**[b]	D		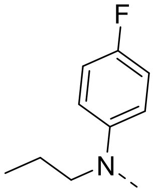		2	2	4	
**983**[b]	D		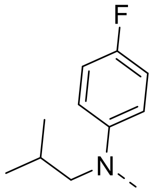		2	4	8	
**986**	D		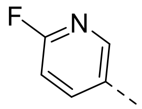		>32	>32	>32	>32
**987**	C	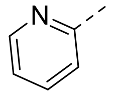			8	16	32	32
**988**	D		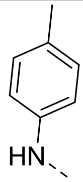		4	4	16	16

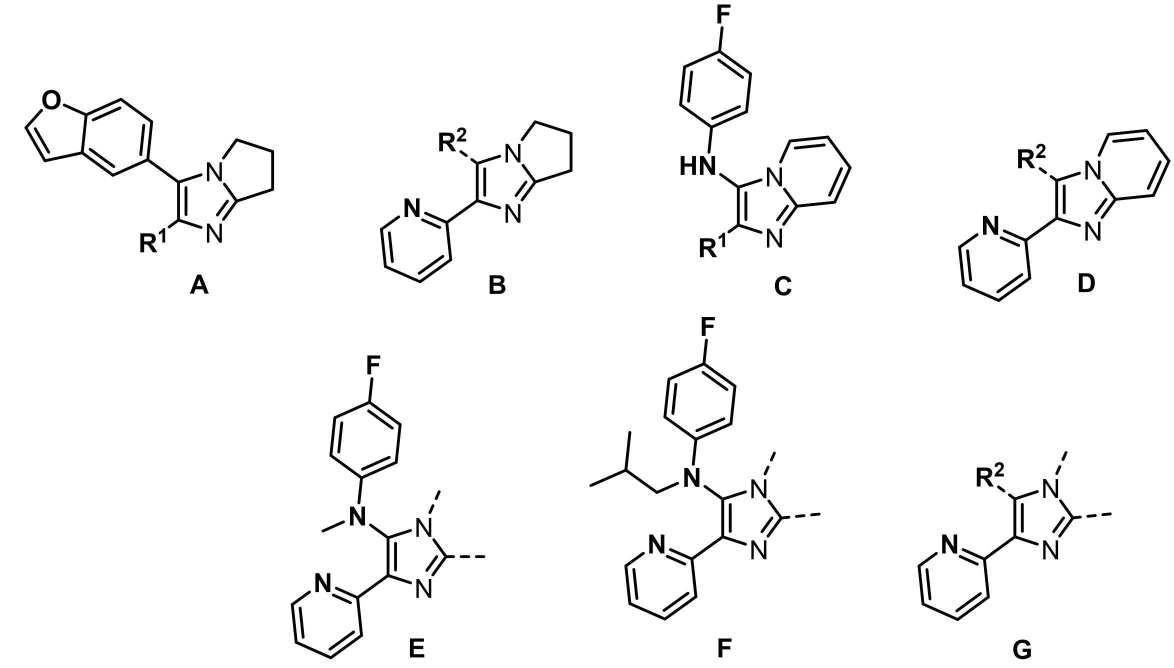

Cmpd	Structure	R¹	R²	Core	MRSA MIC (µg/mL)	MSSA MIC (µg/mL)	VRE MIC (µg/mL)	VSE MIC (µg/mL)
**1001[b]**	E			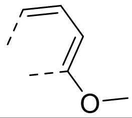	16	16		
**1002[b]**	D		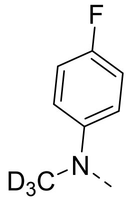		16	16		
**1003[b]**	E			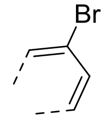	16	16		
**1004[b]**	E			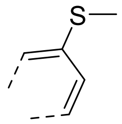	8	8		
**1005[b]**	E			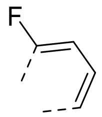	32	32		
**1006[b]**	E			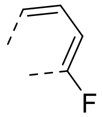	>32	>32		
**1008**	C	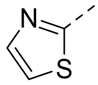			>32	>32		
**1009**	C	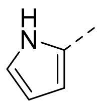			>32	>32		

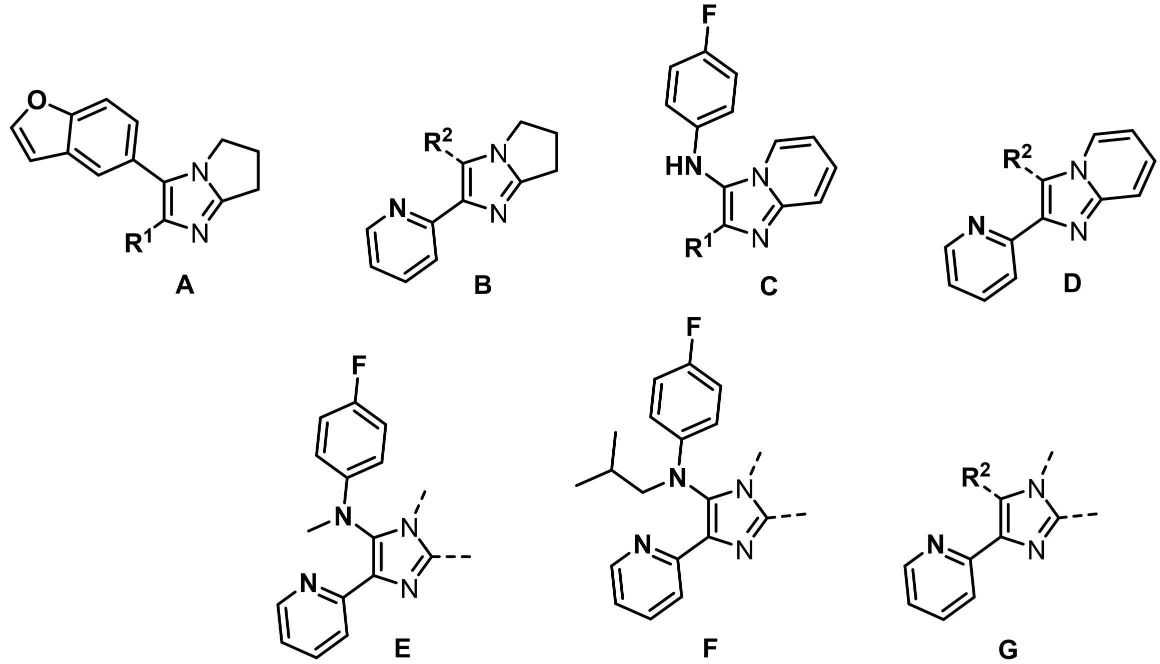

Cmpd	Structure	R¹	R²	Core	MRSA MIC (µg/mL)	MSSA MIC (µg/mL)	VRE MIC (µg/mL)	VSE MIC (µg/mL)
**1011**	D		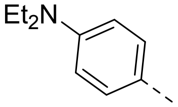		4	4		
**1012**	D		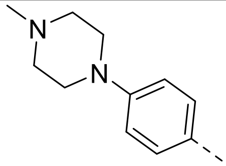		>32	>32		
**1016**	D		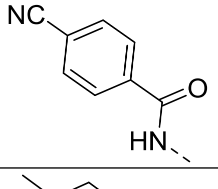		>32	>32		
**1017**	D		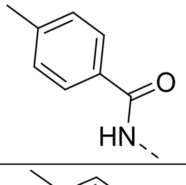		>32	>32		
**1018**	D		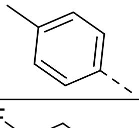		4	4		
**1019[b]**	D		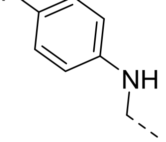		>32	>32		

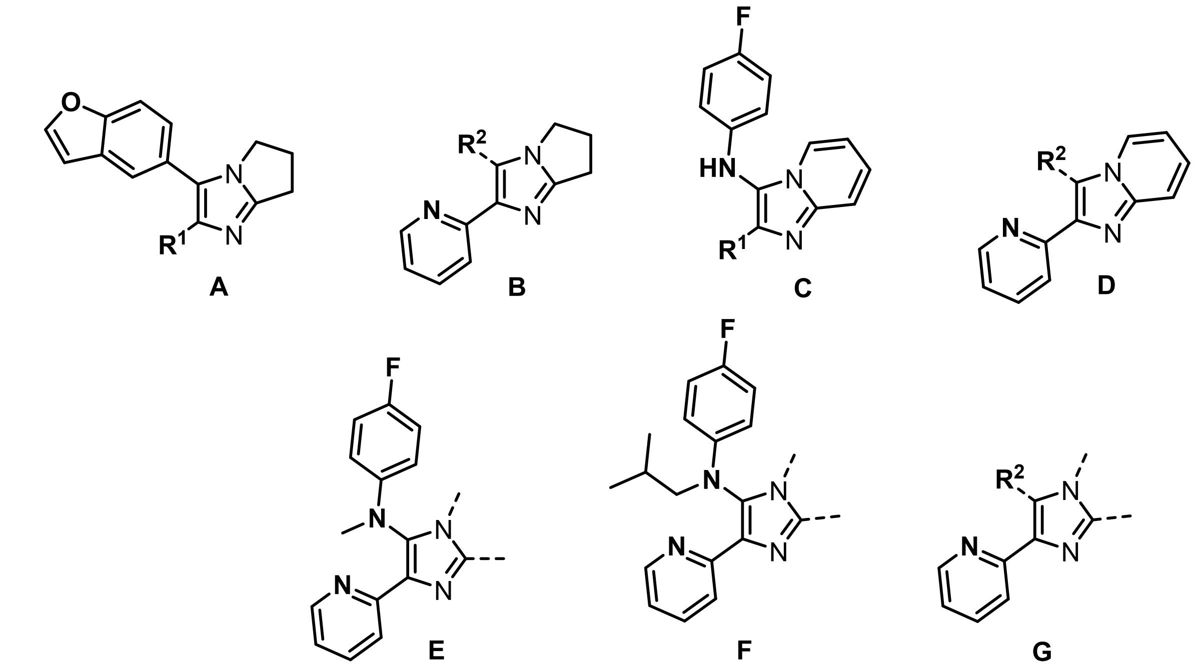

Cmpd	Structure	R¹	R²	Core	MRSA MIC (µg/mL)	MSSA MIC (µg/mL)	VRE MIC (µg/mL)	VSE MIC (µg/mL)
1021[b]	D		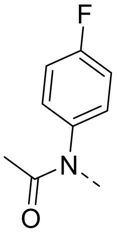		>32	>32		
1022[b]	C	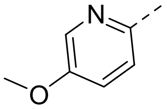			8	8		
1035[b]	F			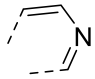	>32	>32		
1036[b]	F			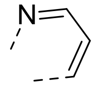	32	32		
1038[b]	F			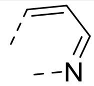	6	8		
1039[b]	F			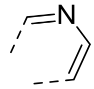	>32	>32		
1040[b]	D		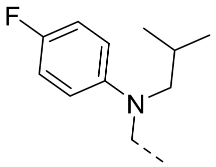		32	>32		
1052	B		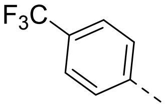		16	16		

a. Compounds were donated by the Drugs for Neglected Diseases *initiative* (DND*i*) for which the experimental data have been published.[1] b. Compounds were contributed from Northeastern University, the experimental details for which may be found in a separate publication.

Table S2. *In vitro* potencies against MRSA and MSSA, as well as VRE and VSE for analogues with variations at the R1, R2 and core for additional compounds.

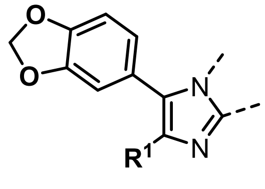
H

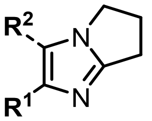
I

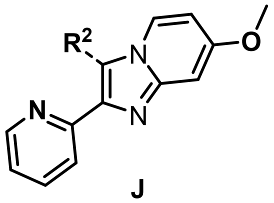
J

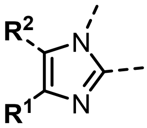
K

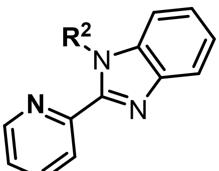
L

Cmpd	Structure	R1	R2	Core	MRSA MIC (µg/mL)	MSSA MIC (µg/mL)	VRE MIC (µg/mL)	VSE MIC (µg/mL)
**813**	H	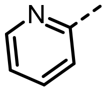		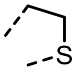	32			
**815**	H	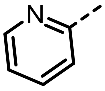		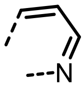	>32			
**816**	H	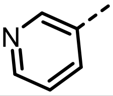		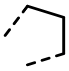	>32			
**817**	H	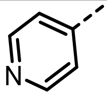		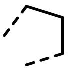	>32			
**818**	H	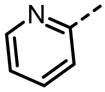		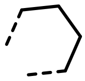	16			
**834**	I	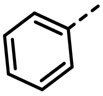	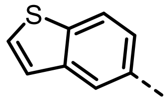		>32			
**839**[a]	J		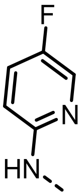		16		>32	
**840**[a]	K	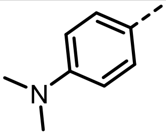	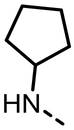	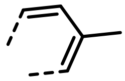	>32		>32	
**841**[a]	K	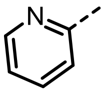	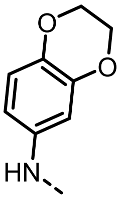	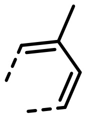	8		16	

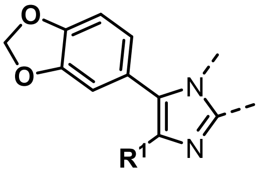
H
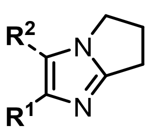
I
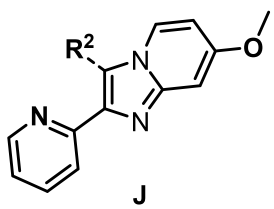
J
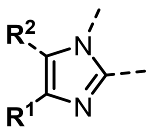
K
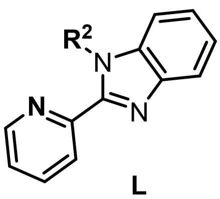
L

Cmpd	Structure	R¹	R²	Core	MRSA MIC (µg/mL)	MSSA MIC (µg/mL)	VRE MIC (µg/mL)	VSE MIC (µg/mL)
**842**[a]	K	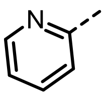	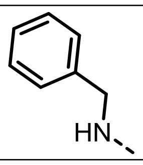	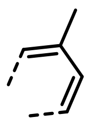	8	4	32	
**843**[a]	K	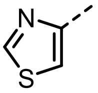	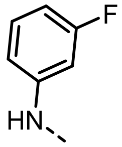	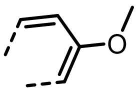	>32		>32	
**844**[a]	K	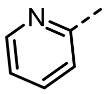	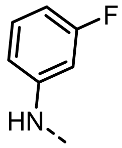	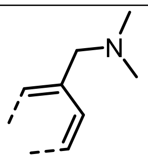	>32		>32	
**845**[a]	K	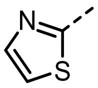	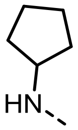	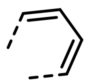	>32		>32	
**846**[a]	K	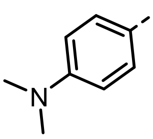	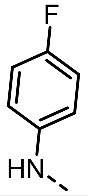	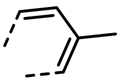	>32		>32	
**848**[a]	K	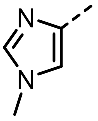	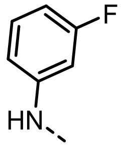	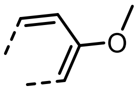	>32		>32	
**849**[a]	K	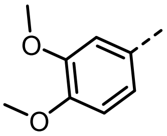	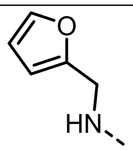	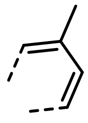	>32		>32	
**850**[a]	K	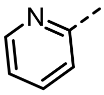	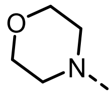	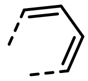	>32		>32	
**851**[a]	J		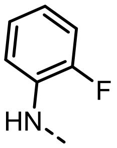		8	4	16	

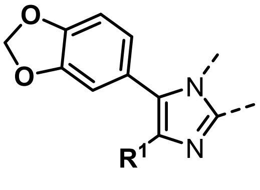 H	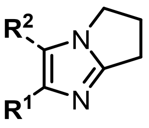 I	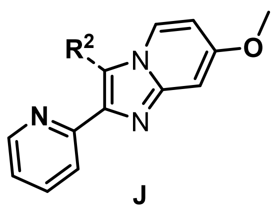 J	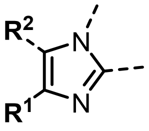 K	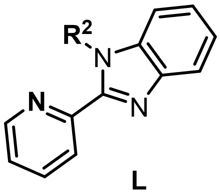 L

Cmpd	Structure	R^1	R^2	Core	MRSA MIC (µg/mL)	MSSA MIC (µg/mL)	VRE MIC (µg/mL)	VSE MIC (µg/mL)
**852**[a]	K	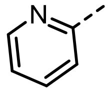	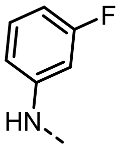	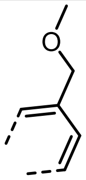	>32		>32	
**853**[a]	J		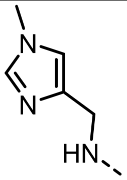		>32		>32	
**854**[a]	J		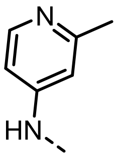		>32		>32	
**855**[a]	K	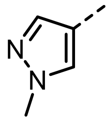	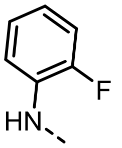	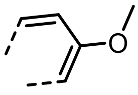	>32		>32	
**856**[a]	J		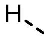		>32		>32	
**857**[a]	J		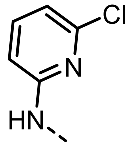		>32		>32	
**858**[a]	J		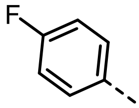		16		>32	
**860**[a]	J		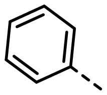		8		32	
**863**	K	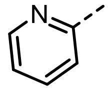	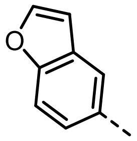	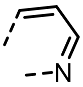	>32		>32	
**866**[a]	K	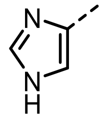	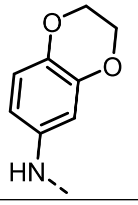	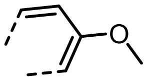	>32		>32	

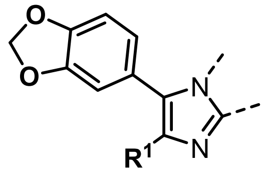
H

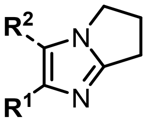
I

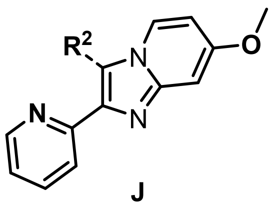
J

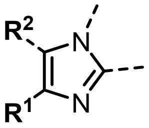
K

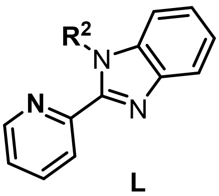
L

Cmpd	Structure	R¹	R²	Core	MRSA MIC (µg/mL)	MSSA MIC (µg/mL)	VRE MIC (µg/mL)	VSE MIC (µg/mL)
**867**[a]	K	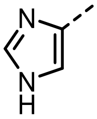	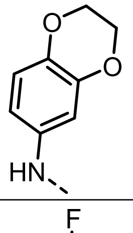	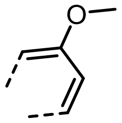	>32		>32	
**984**[b]	K	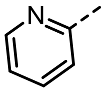	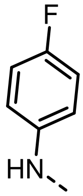	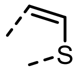	8	4	8	
**985**	K	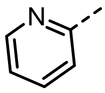	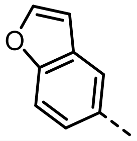	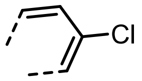	8	8	16	16
**989**	L		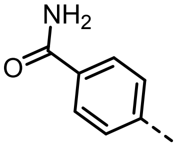		>32	>32	>32	>32
**990**	L		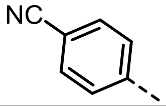		>32	>32	>32	>32
**991**	L		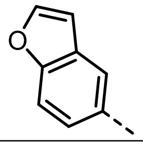		>23	>32	>32	>32
**992**	L		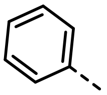		>32	>32	>32	>32
**993**	L		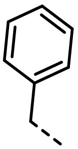		>32	>32	>32	>32
**994**	L		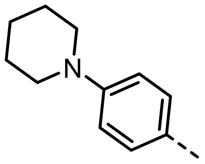		16	32	32	32

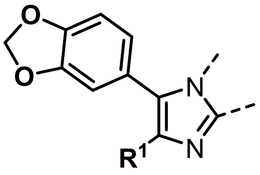
H

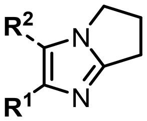
I

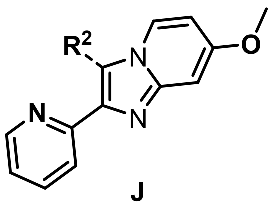
J

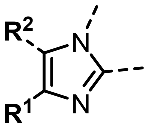
K

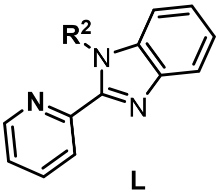
L

Cmpd	Structure	R¹	R²	Core	MRSA MIC (µg/mL)	MSSA MIC (µg/mL)	VRE MIC (µg/mL)	VSE MIC (µg/mL)
**995**	L		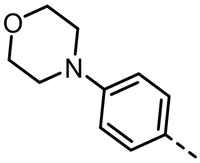		>32	>32	>32	>32
**996**	L		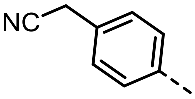		>32	32	>32	>32
**997**	K	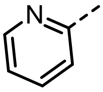	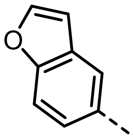	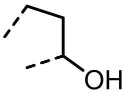	>32	>32	>32	>32
**998[b]**	K	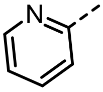	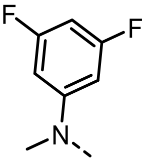	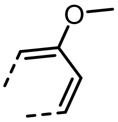	8	8	32	16
**999[b]**	K	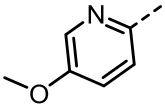	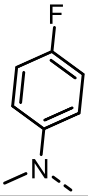	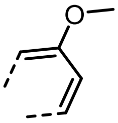	8	8	16	16
**1000[b]**	K	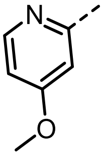	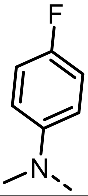	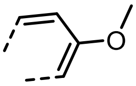	2	8	8	8
**1007[b]**	K	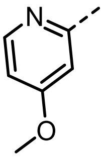	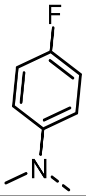	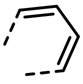	8	8		
**1010**	K	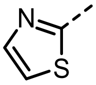	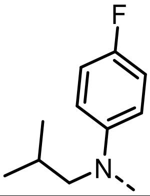	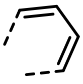	>32	>32		

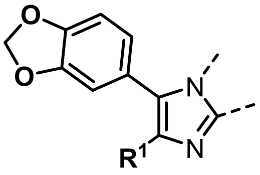

H

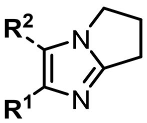

I

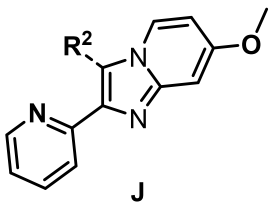

J

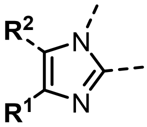

K

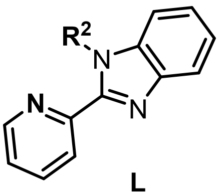

L

Cmpd	Structure	R1	R2	Core	MRSA MIC (µg/mL)	MSSA MIC (µg/mL)	VRE MIC (µg/mL)	VSE MIC (µg/mL)
**1013**	K	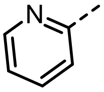	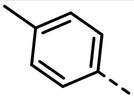	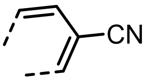	>32	>32		
**1014**	K	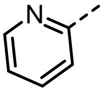	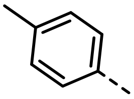	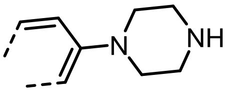	>32	>32		
**1015**	K	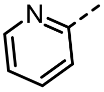	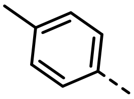	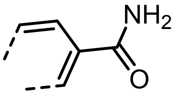	>32	>32		
**1020**[b]	K	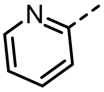	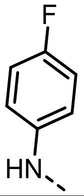	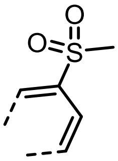	>32	>32		
**1023**[b]	K	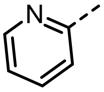	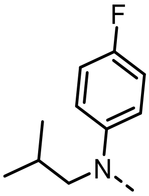	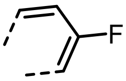	8	4		
**1024**[b]	K	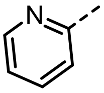	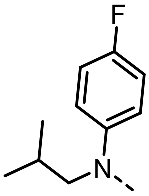	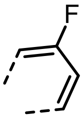	8	8		
**1025**[b]	K	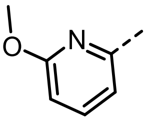	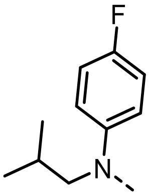	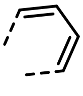	>32	>32		
**1026**	K	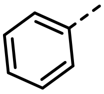	H	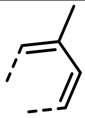	>32	>32		
**1027**	K	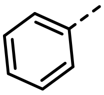	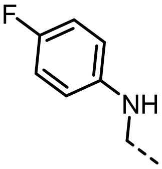	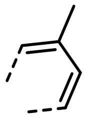	>32	>32		

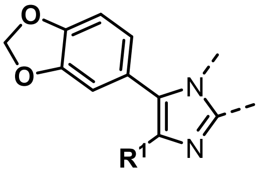

H

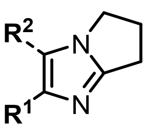

I

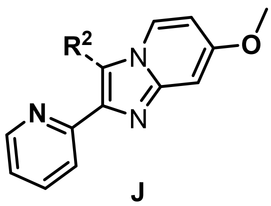

J

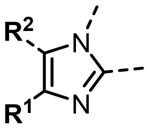

K

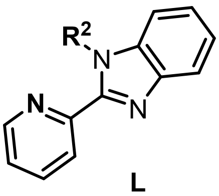

L

Cmpd	Structure	R[1]	R[2]	Core	MRSA MIC (µg/mL)	MSSA MIC (µg/mL)	VRE MIC (µg/mL)	VSE MIC (µg/mL)
**1028**	K	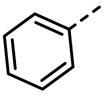	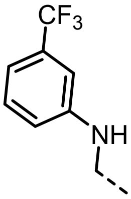	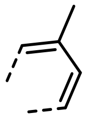	>32	>32		
**1029**	K	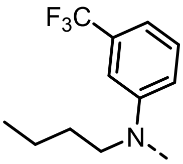	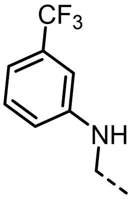	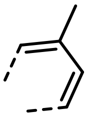	8	8		
**1030**	K	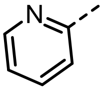	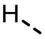	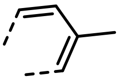	>32	>32		
**1031**	K	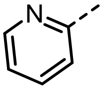	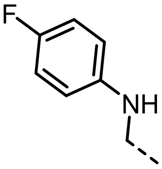	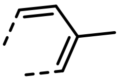	>32	>32		
**1032**	K	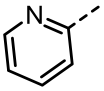	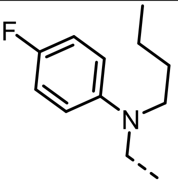	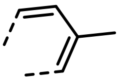	32	32		
**1033**	K	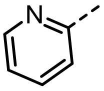	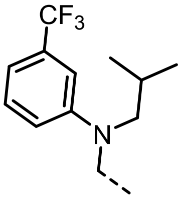	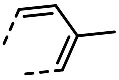	>32	>32		
**1034[b]**	K	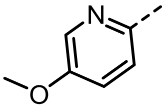	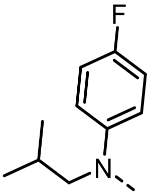	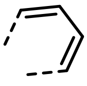	8	02-Apr		
**1037[b]**	K	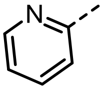	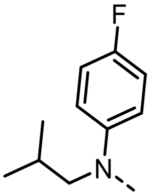	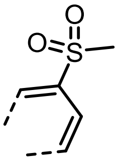	>32	>32		

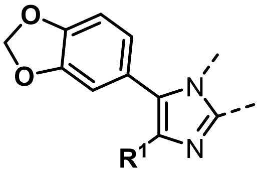

H

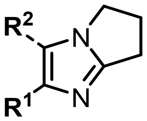

I

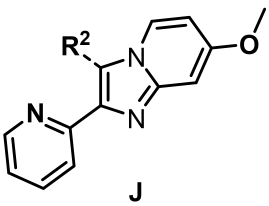

J

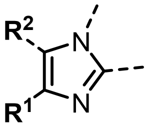

K

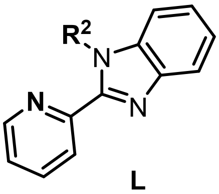

L

Cmpd	Structure	R1	R2	Core	MRSA MIC (µg/mL)	MSSA MIC (µg/mL)	VRE MIC (µg/mL)	VSE MIC (µg/mL)
**1053**	I	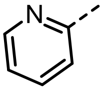	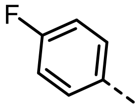		>32	>32		
**1072**	I	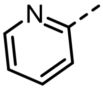	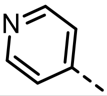		>32	>32		
**1073**	I	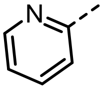	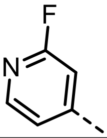		>32	>32		
**1074**	I	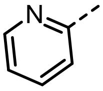	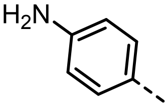		>32	>32		
**1075**	I	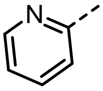	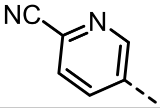		>32	>32		
**1076**	I	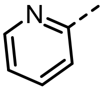	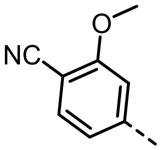		>32	>32		
**1077**	I	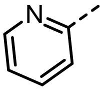	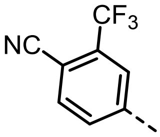		>32	>32		
**1102[b]**	K	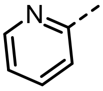	H	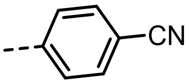	>32	>32		
**1103[b]**	K	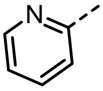	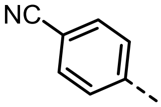	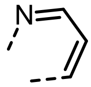	>32	>32		
**1104[b]**	L		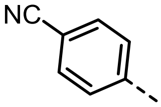		>32	>32		
**1105[b]**	K	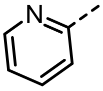	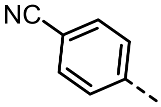	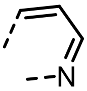	>32	>32		
**1106[b]**	K	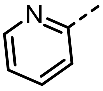	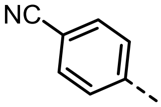	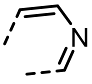	>32	>32		

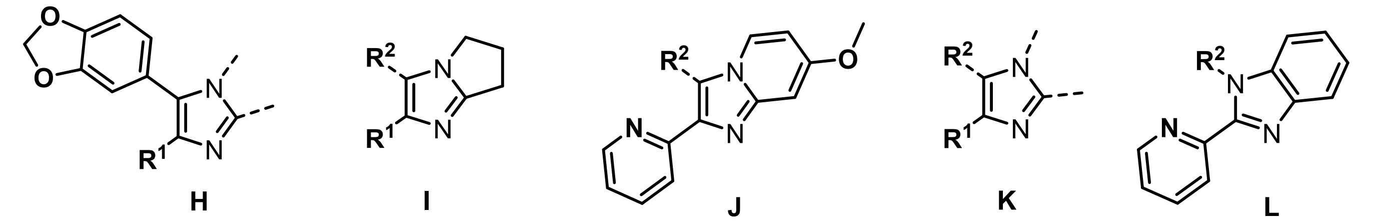

Cmpd	Structure	R[1]	R[2]	Core	MRSA MIC (µg/mL)	MSSA MIC (µg/mL)	VRE MIC (µg/mL)	VSE MIC (µg/mL)
**1107[b]**	K	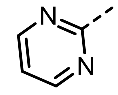	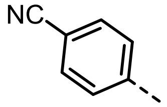	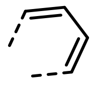	>32	>32		

a. Compounds were donated by the Drugs for Neglected Diseases *initiative* (DND*i*) for which the experimental data have been published.[1] b Compounds were contributed from Northeastern University, the experimental details for which may be found in a separate publication.[2]

Table S3. ADME and Pharmacokinetics: compounds that are included in the manuscript

Cmpd	MRSA MIC (µg/mL)	Aqueous Solubility (µM)	Human PPB (%)	HLM CLint (µL/min/mg protein)	Rat Hep CLint (µL/min/10x6 cells)	$LogD_{7.4}$
**812**	32	756	55	<3	<1	0.9
**814**	32	556	93.9	76.1	62.5	2.8
**819**	16	344	98.5	35.5	40.8	3.2
**820**	32	317	60	<3	<1	1.2
**820**	>32	179	85	64	25.9	2.2
**821**	2	178		168	49.8	3
**822**	2	117	97.2	63.5	13.6	2.7
**822**	4	116	96	138	> 300	2.6
**823**	16	89	98.3	36.8	44.5	3.2
**824**	4	64	96.5	79.3	> 300	1.8
**825**	16	43	98.9	122	57.7	3.3
**826**	4	38	95.6	62.2	54.1	2.9
**827**	32	38	71	99.1	7.99	1.9

Cmpd	MRSA MIC (µg/mL)	Aqueous Solubility (µM)	Human PPB (%)	HLM CLint (µL/min/mg protein)	Rat Hep CLint (µL/min/10x6 cells)	$LogD_{7.4}$
**828**	32	38	99.6	159	192	4.3
**829**	4	37	76	43.7	23.2	2
**830**	4	32	91.6	82	49.2	2.7
**831**	8	31	97.8	180	148	2.8
**832**	8	26	97.4	65.9	> 300	3.5
**833**	>32	26	96.6	127	209	3.3
**835**	32	21	99.69	224	34.5	4.2
**836**	32	17	98	251	> 300	2.9
**837**	>32	10	90.2	34.1	49.1	2.6
**838**	>32	10	83	19.8	81.2	2.5
**847**	>32	8	99.58	58.8	234	4.6
**859**	32	7	99.46	> 300	> 300	4.5
**861**	>32	6	99.26	105	>300	3.3
**862**	>32	5	99.45	65.6	104	3.1
**864**	>32	4	96.7	168	21.9	3
**865**	4	4	99.82	61.6	> 300	4.2
**868**	8	3	97.7	61.5	>300	2.9
**869**	>32	3	99.92	42.6	151	4.1
**870**	32	3	98.5	100	37.7	3.4
**871**	4	3	99.27	61.8	108	3.6
**872**	>32	3	98.3	34.8	88.2	3.8
**873**	>32	2	99.33	105	141	4
**874**	>32	0.5	98.3	148	151	3.3

Table S4. ADME and Pharmacokinetics: compounds only appearing in the Supplementary Information

Cmpd	MRSA MIC (µg/mL)	Aqueous Solubility (µM)	Human PPB (%)	HLM CLint (µL/min/mg protein)	Rat Hep CLint (µL/min/10x6 cells)	$LogD_{7.4}$
**813**	32	723	79	> 300	52.9	2.2
**815**	>32	85	94.6	> 300	25.1	2.9
**816**	>32	79	97.2	224	23.9	3.1
**817**	>32	54	98.6		>300	0.9
**818**	16	37	96.5	124	12.9	2.5
**834**	>32	27	99.37	261	>300	3.7
**839**	16	26	97.5	214	> 300	3.4
**840**	>32	22	Low recovery,unstable in plasma	< 3	82.3	
**841**	8	17	99.86	51.2	117	> 4.2
**842**	8	12	> 96.7	51.2	> 300	3.1
**843**	>32	11	99.77	13.6	110	4.3
**844**	>32	8	99.15	100	>300	4
**845**	>32	5	89	92	> 300	2.4
**846**	>32	0.7	> 99	86.8		> 3.7

ADME and toxicity for compounds were measured according to procedures outlined in the manuscript submitted in parallel to this one;[2] other procedures were used for those compounds analysed by Monash University (*in vitro* ADME) or UCL (toxicity), both described in the main manuscript.

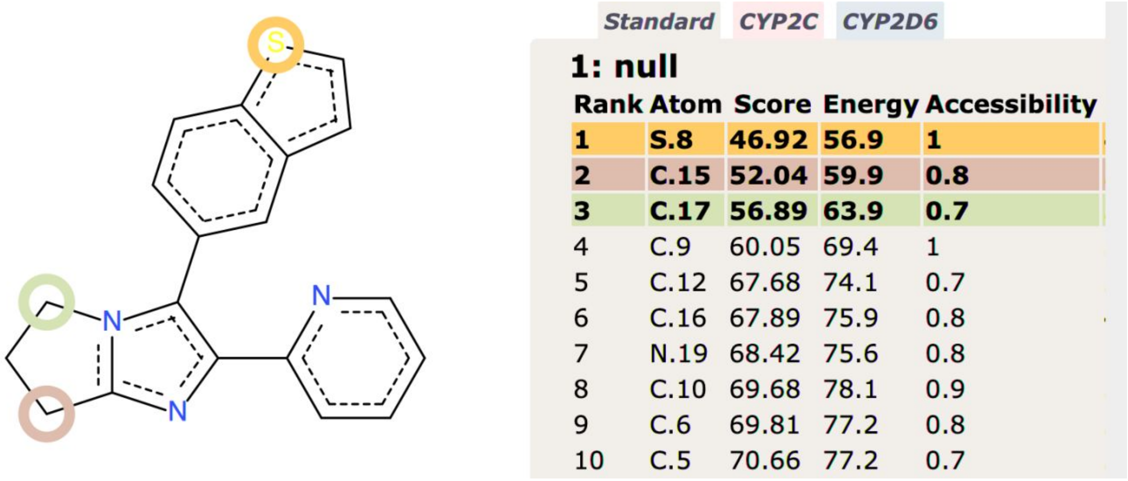

Figure S3. Highlighted potential sites of metabolism of the benzothiophene and the diaryl portions of **OSA_822**; data derived from https://smartcyp.sund.ku.dk/mol_to_som.

Table S5. MRSA activities and cytotoxicity expressed in TC50 (µg/mL). Selectivity index (SI) is expressed as the ratio TC_{50} (MRC-5 and PMM)/MRSA rounded to the nearest integer.

OSA_ID	MRSA MIC (µg/mL)	MRC5 TC_{50} (µg/mL)	PMM TC_{50} (µg/mL)	SI MRC5	SI PMM
**855**	32	21.6	21.6	1	1
**856**	32	11.6	14.4	0	0
**857**	32	8.2	21.6	0	1
**858**	16	1.2	2.6	0	0
**859**	32	3.0	9.3	0	0
**860**	8	1.2	2.4	0	0
**872**	32	2.0	15.3	0	0
**875**	4	1.6	2.4	0	1
**975**	4	7.4	18.3	2	5
**979**	2	2.8	1.4	1	1
**980**	4	0.7	2.8	0	1
**981**	2	1.6	2.7	1	1

OSA_ID	MRSA MIC (µg/mL)	MRC5 TC_{50} (µg/mL)	PMM TC_{50} (µg/mL)	SI MRC5	SI PMM
**982**	2	1.5	0.7	1	0
**983**	2	19.5	8.3	10	4
**984**	8	2.0	9.9	0	1
**987**	8	2.3	9.7	0	1
**989**	32	20.1	20.1	1	1
**990**	32	19.0	19.0	1	1
**991**	23	19.9	12.0	1	1
**992**	32	17.4	17.4	1	1
**993**	32	18.3	18.3	1	1
**994**	16	7.0	5.4	0	0
**995**	32	22.8	22.8	1	1
**996**	32	19.9	19.9	1	1
**1019**	32	20.4	20.4	1	1
**1020**	32	24.5	12.2	1	0
**1021**	32	22.2	22.2	1	1
**1022**	8	6.3	6.8	1	1
**1026**	32	13.3	13.3	0	0
**1027**	32	21.2	21.2	1	1
**1028**	32	24.4	24.4	1	1
**1029**	8	0.5	3.5	0	0
**1030**	32	4.6	13.4	0	0
**1031**	32	21.3	21.3	1	1
**1032**	32	6.2	12.4	0	0
**1033**	32	4.3	14.0	0	0
**1052**	16	2.7	10.5	0	1

OSA_ID	MRSA MIC (µg/mL)	MRC5 TC_{50} (µg/mL)	PMM TC_{50} (µg/mL)	SI MRC5	SI PMM
**1053**	32	2.1	8.9	0	0
**1072**	32	16.8	16.8	1	1
**1073**	32	8.6	9.0	0	0
**1074**	32	1.6	2.2	0	0
**1075**	32	18.4	18.4	1	1
**1076**	32	8.8	20.2	0	1
**1077**	32	22.7	22.7	1	1

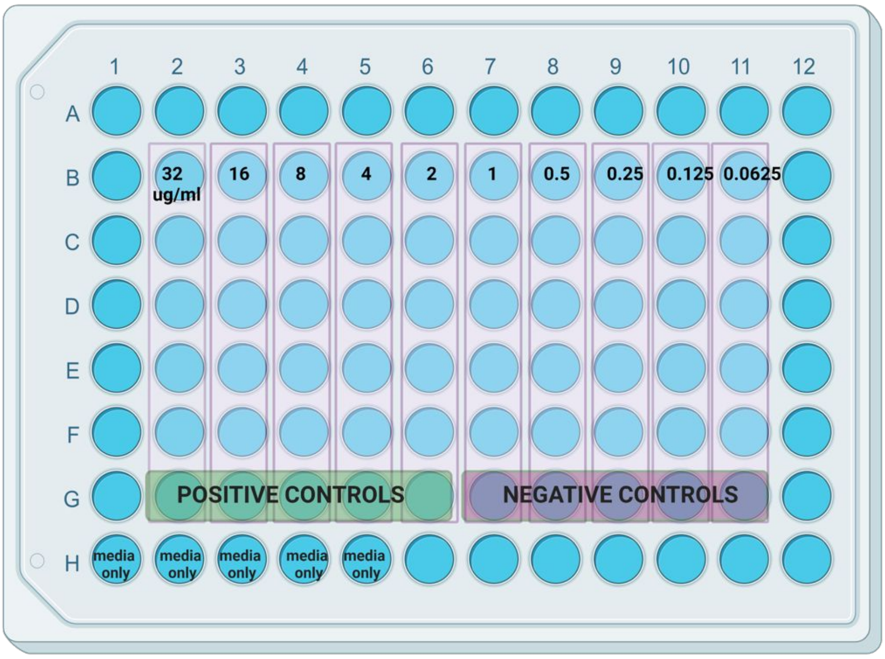

Figure S4. Graphic illustration of plate setup for dosing experiments showing negative, positive, media only controls and experimental wells (decreasing concentration).

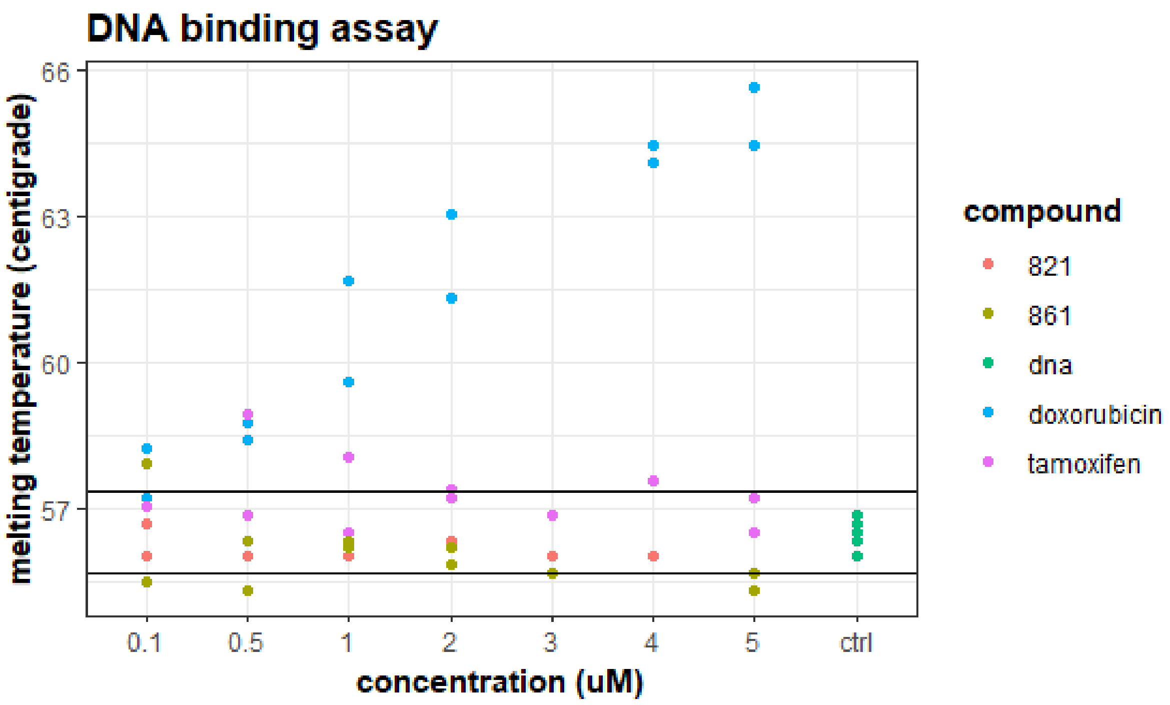

Figure S5. Melting temperature of custom designed molecular beacons was measured to assess compound interaction with DNA. Control DNA melting temperature was 56.53 ± 0.84 °C.

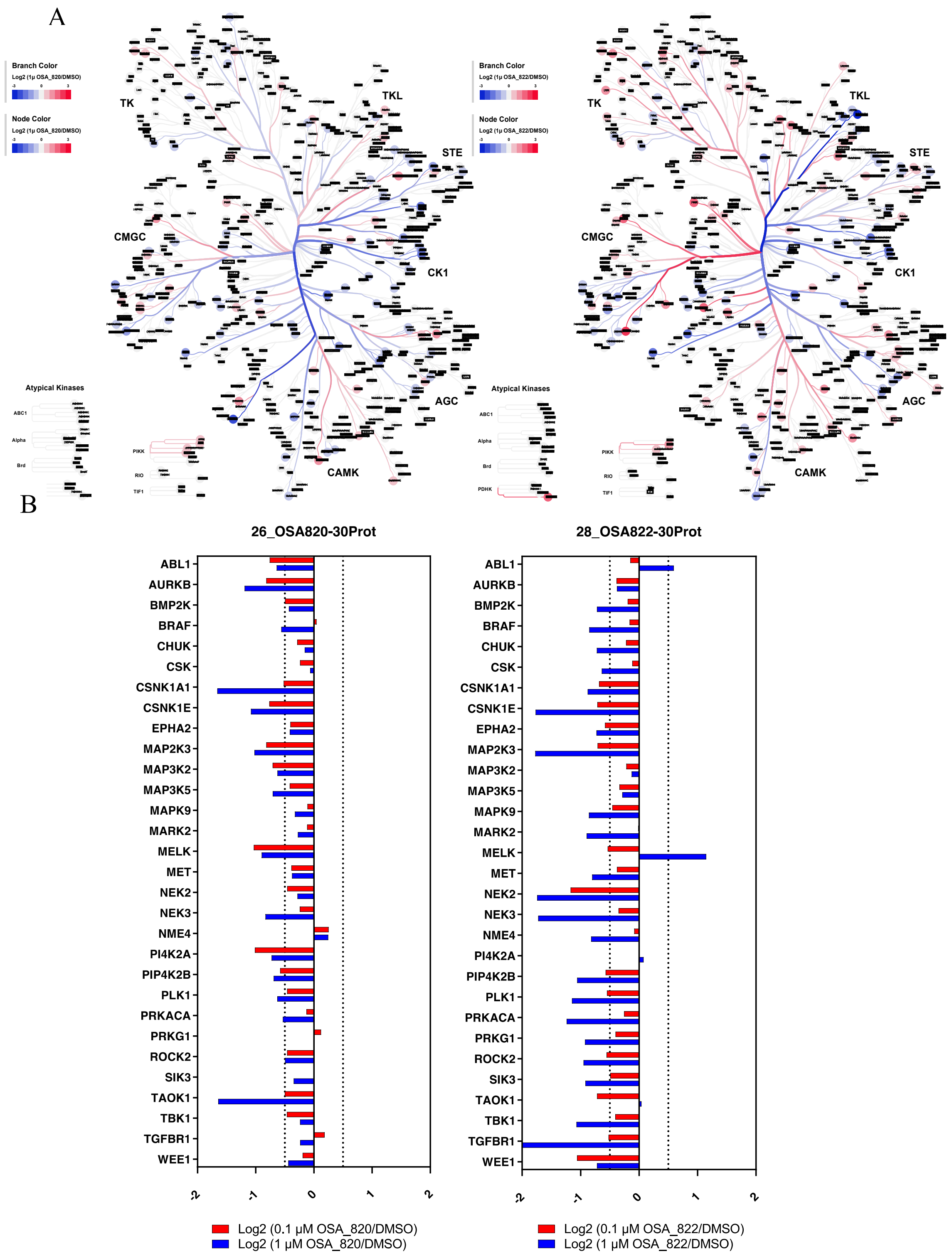

Figure S6: HEK Cell Lysate MIB-MS Experiments. A.) The kinase dendrogram plot shows increase or decrease in MIB binding for 211 kinases for **OSA_820** and for 209 kinases for **OSA_822** for the 1 uM drug treated samples. The Kinome tree was generated using Coral

(http://phanstiel-lab.med.unc.edu/CORAL/) and edited using Inskape. **B.**) Major human (HEK) kinases displaced from MIBs as determined by competition assay using two different concentrations of **OSA_820** and **OSA_822** (0.1 and 1.0 uM). Samples plotted relative to DMSO control.

Table S6: Results for the dose escalation experiment of CYP483 dosed with **OSA_000821** (values are peak areas of integrated chromatograms extracted at UV_{298nm}). The focus of metabolism was on the primary +16 Da product, with the other tentative 16 Da product not changing in yield and therefore suspected as potentially endogenous-derived isobaric component.

Dose (mg/L)	Parent (1.89 mins) 302m/z	+16Da (1.59 mins) 318m/z
50	312	144
100	1311	329
200	4170	401
300	6555	438

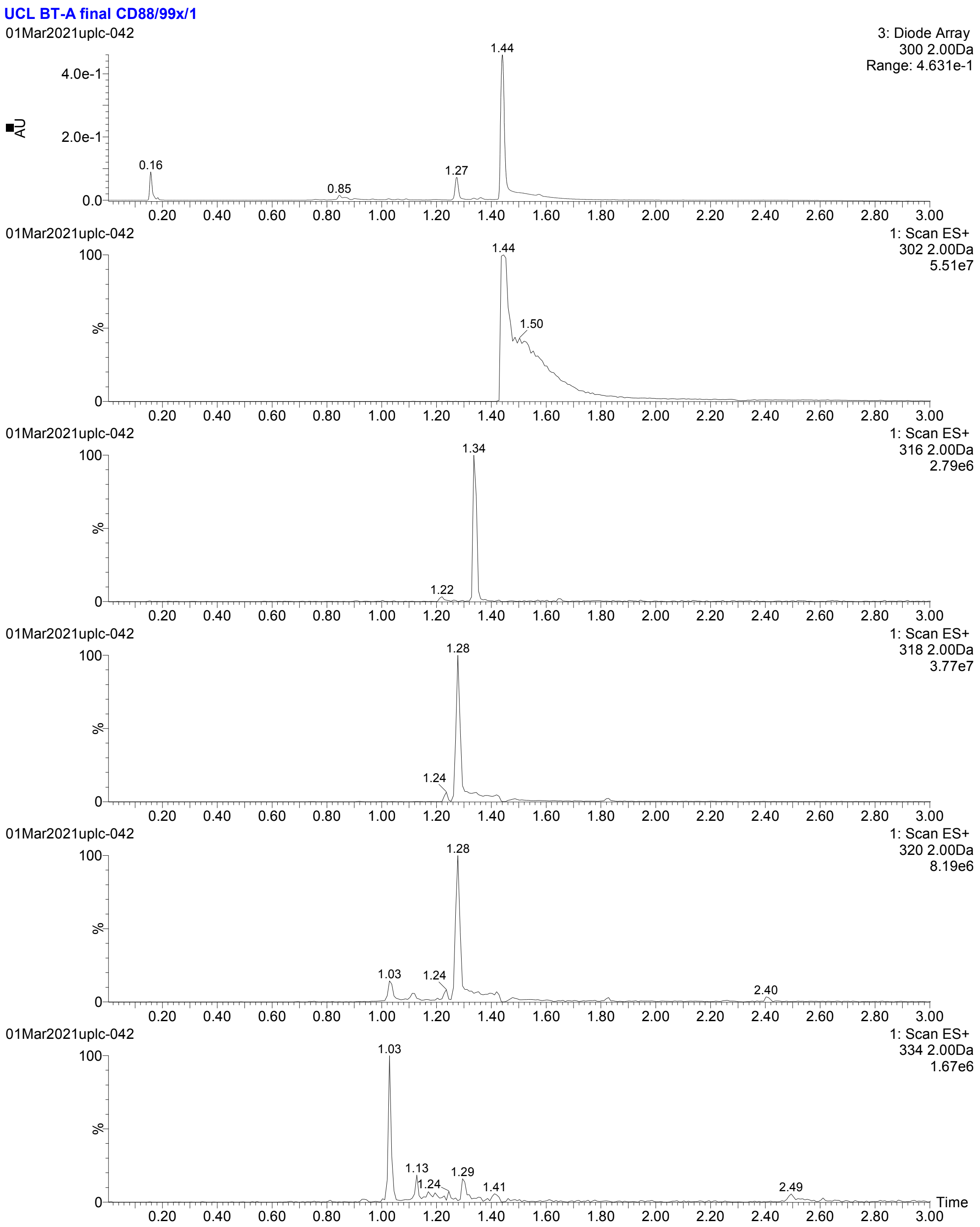

Figure S7: Chromatograms of a reaction extract from PolyCYP483 vs UCL-BT-A

(**OSA_000821**). From top to bottom the chromatograms are UV 300 nm, EIC 302 *m/z* (UCL-BT-A), EIC316m/z (+14Da), EIC318m/z (+16Da), EIC320m/z (+18Da) and EIC334m/z

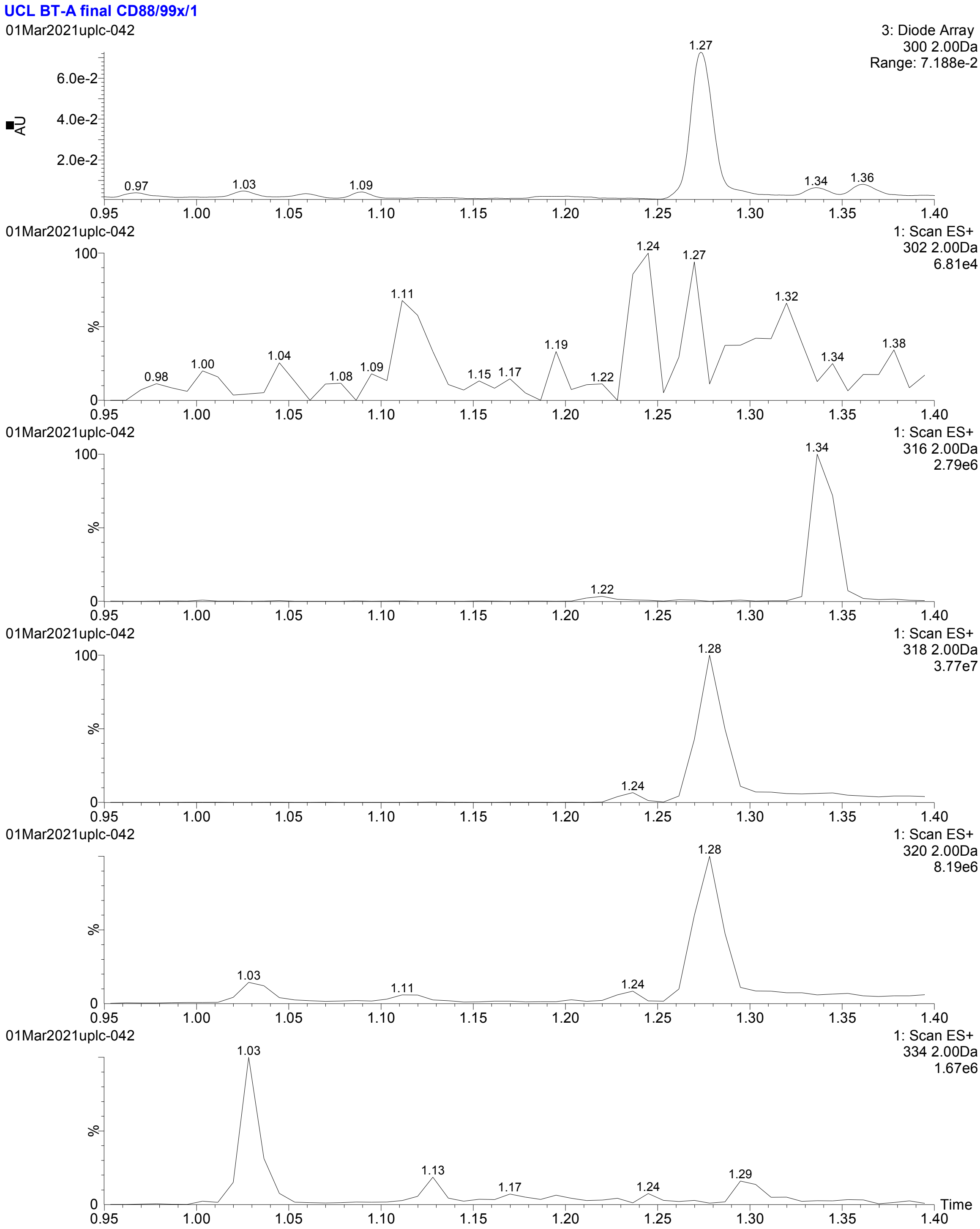

(+32Da). UCL-BT-A elutes at 1.44 minutes. An expansion of the region between 0.95 and 1.40 minutes is provided in Figure S8 below.

Figure S8: Expansion of the chromatograms above between 0.95 and 1.40 minutes (excluding the residual parent component peak). The UV and ESI MS spectra of the component peaks at 1.03, 1.06, 1.27, 1.34 and 1.36 minutes are provided in Figures S9 to S13 below.

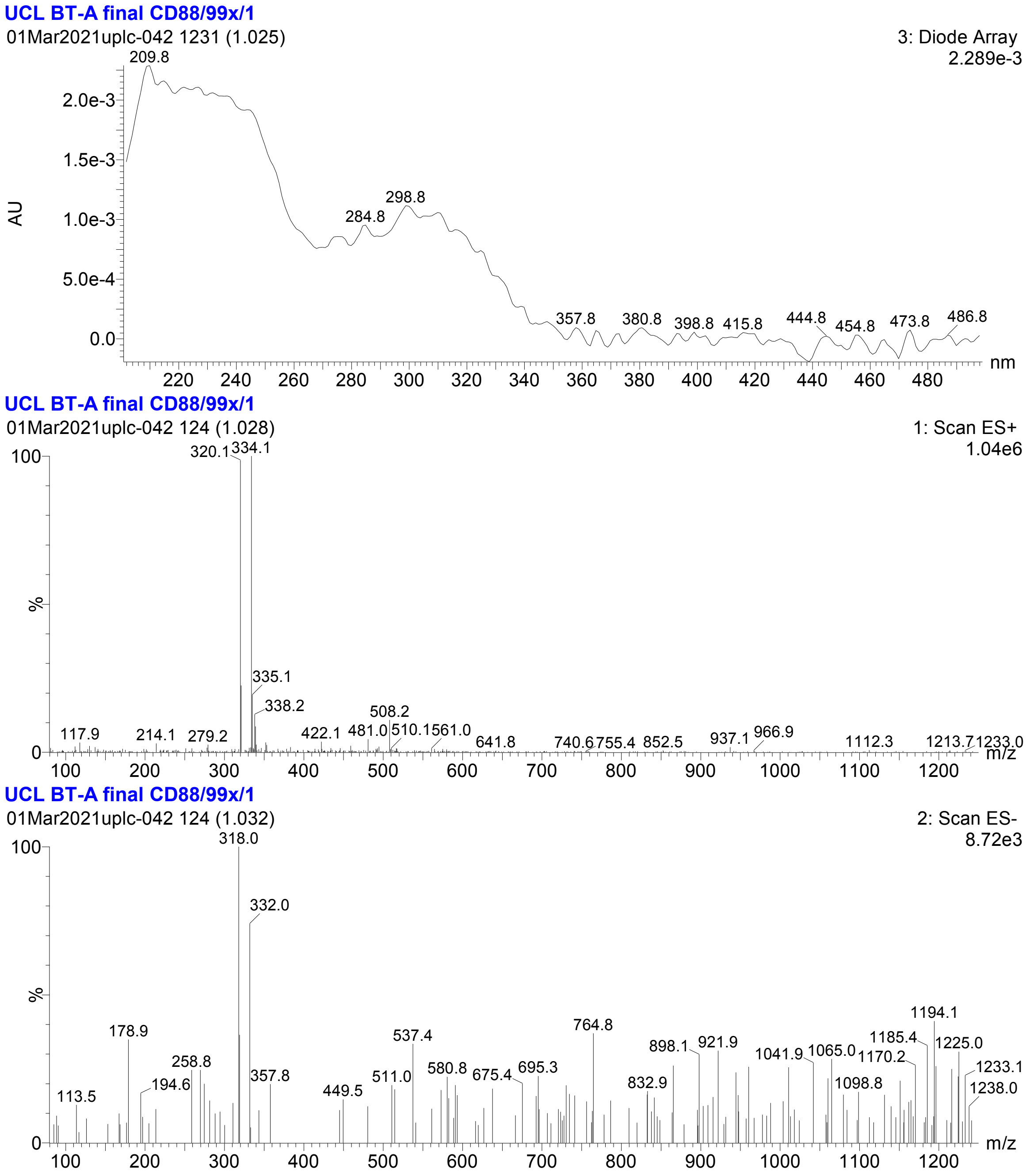

Figure S9: UV, positive ion and negative ion ESI MS spectra of the metabolite eluting at 1.03 minutes in the resulting extract from the reaction of PolyCYP483 dosed with UCL-BT-A (**OSA_000821**).

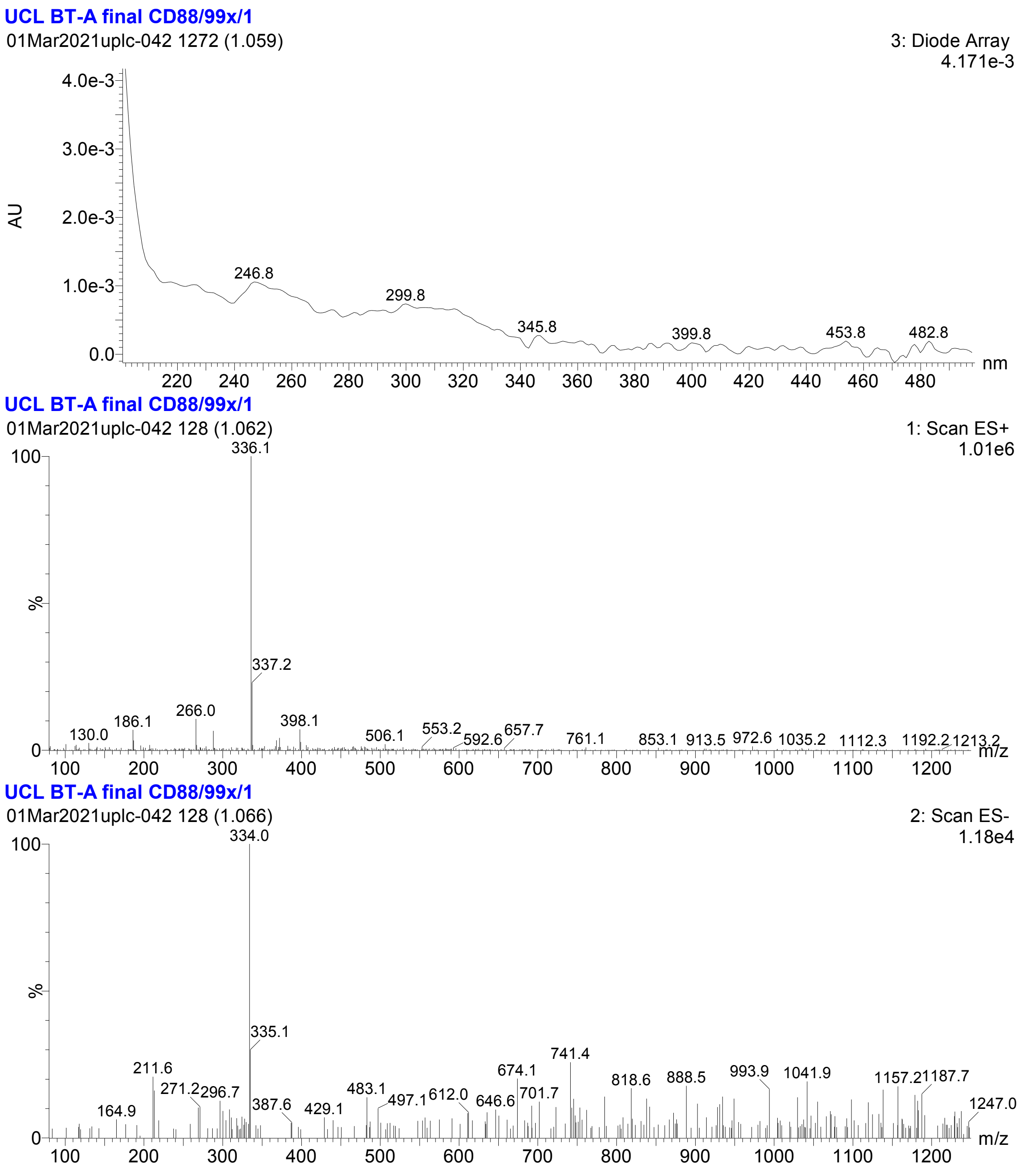

Figure S10: UV, positive ion and negative ion ESI MS spectra of the metabolite eluting at 1.06 minutes in the resulting extract from the reaction of PolyCYP483 dosed with UCL-BT-A (**OSA_000821**).

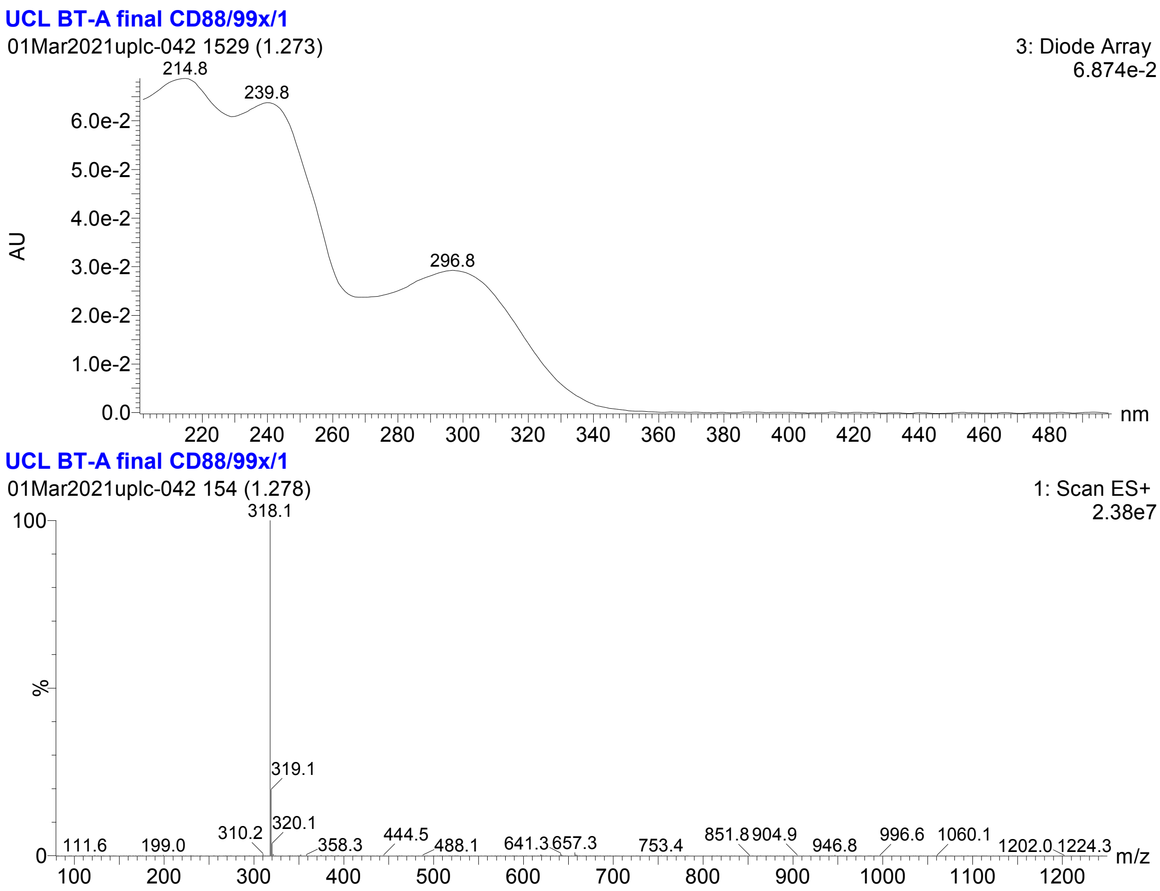

Figure S11: UV, positive ion and negative ion ESI MS spectra of the metabolite eluting at 1.28 minutes in the resulting extract from the reaction of PolyCYP483 dosed with UCL-BT-A (**OSA_000821**). This metabolite did not ionise under standard negative ion conditions.

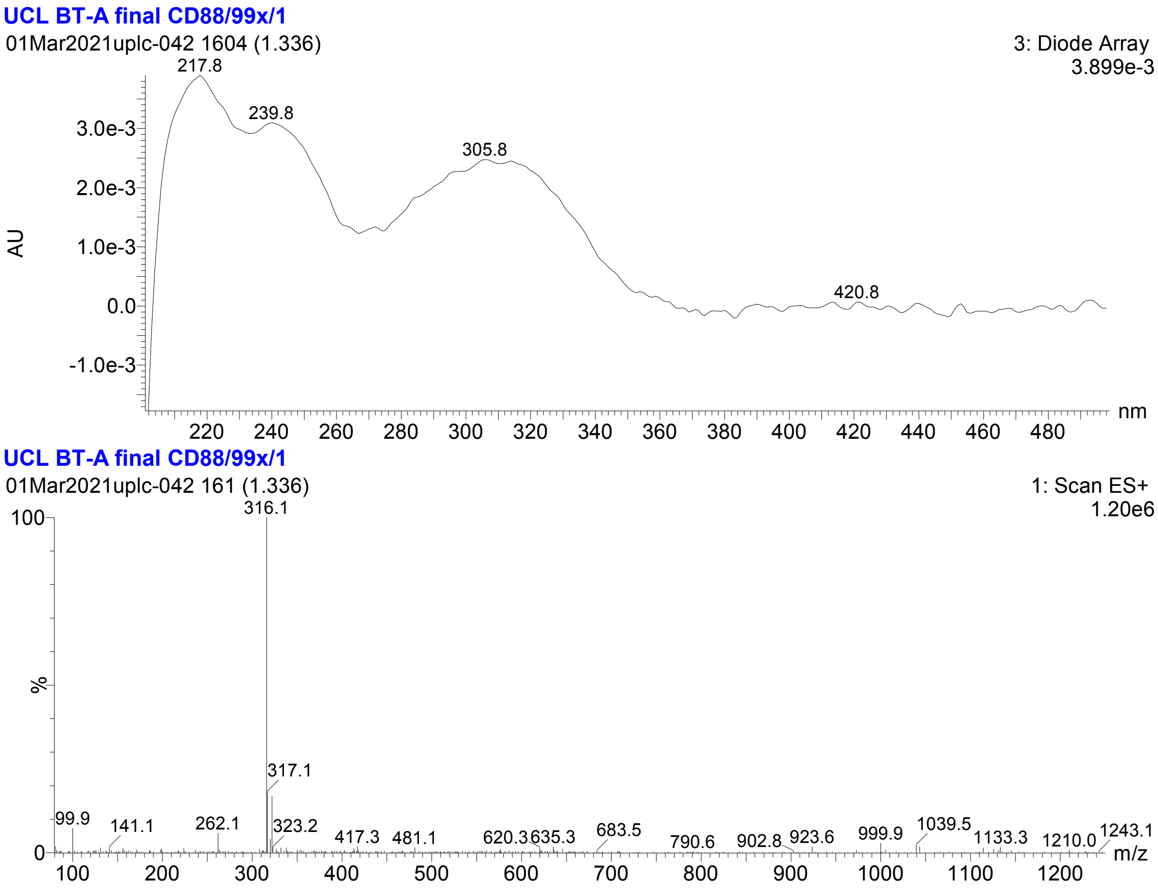

Figure S12: UV, positive ion and negative ion ESI MS spectra of the metabolite eluting at 1.34 minutes in the resulting extract from the reaction of PolyCYP483 dosed with UCL-BT-A (**OSA_000821**). This metabolite did not ionise under standard negative ion conditions.

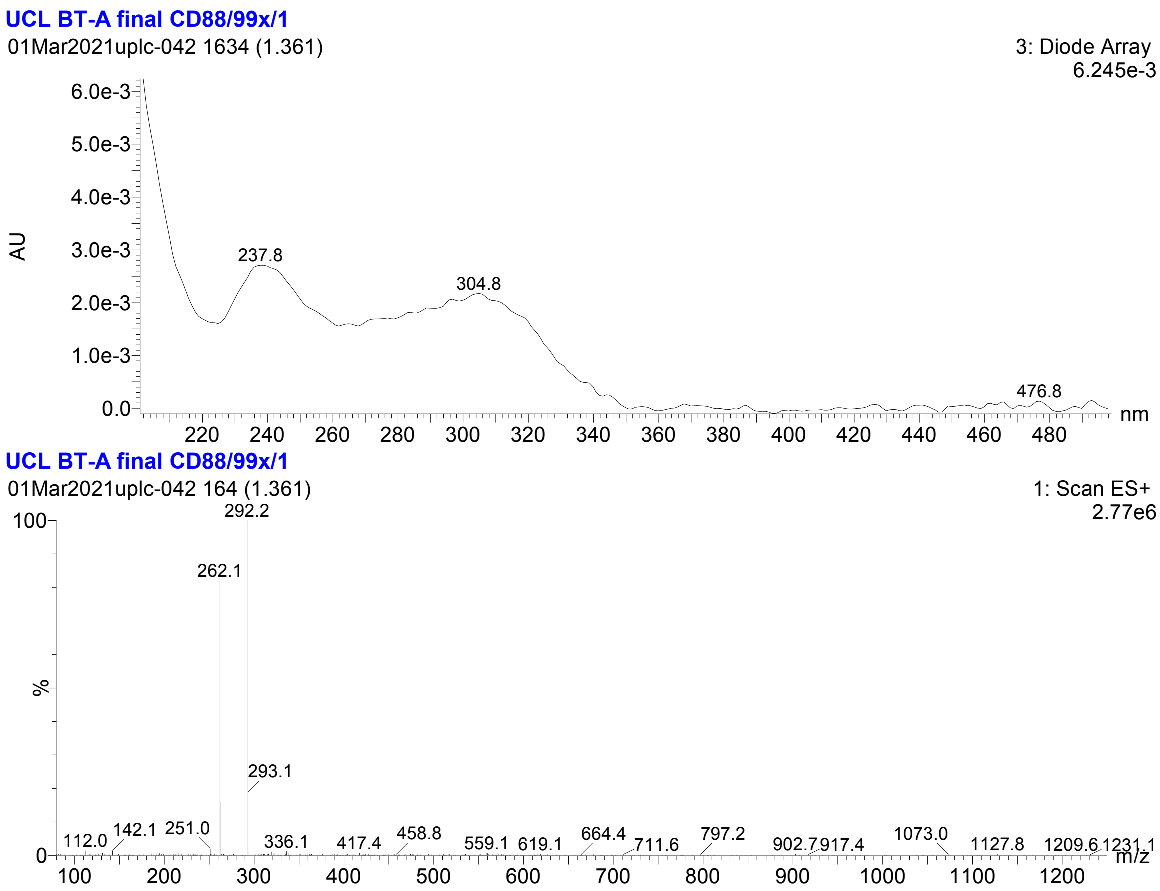

Figure S13: UV, positive ion and negative ion ESI MS spectra of the metabolite eluting at 1.36 minutes in the resulting extract from the reaction of PolyCYP483 dosed with UCL-BT-A (**OSA_000821**). This metabolite did not ionise under standard negative ion conditions.

References

1) Akao, Y.; Canan, S.; Cao, Y.; Condroski, K.; Engkvist, O.; Itono, S.; Kaki, R.; Kimura, C.; Kogej, T.; Nagaoka, K.; Naito, A.; Nakai, H.; Pairaudeau, G.; Radu, C.; Roberts, I.; Shimada, M.; Shum, D.; Watanabe, N.-a.; Xie, H.; Yonezawa, S.; Yoshida, O.; Yoshida, R.; Mowbray, C.; Perry, B., Collaborative Virtual Screening to Elaborate an Imidazo[1,2-a]pyridine Hit Series for Visceral Leishmaniasis. *RSC Med. Chem.* **2021,** *12* (3), 384–393.
2) Dichiara, M.; Simpson, Q. J.; Quotadamo, A.; Jalani, H. B.; Huang, A. X.; Millard, C. C.; Klug, D. M.; Tse, E. G.; Todd, M. H.; Gedder, D.; da Silva Emery, F.; Carlson, J. E.; Zheng, S.-L.; Vleminckx, M.; Matheeussen, A.; Caljon, G.; Pollastri, M. P.; Sjö, P.; Perry, B.; Ferrins, L., Structure-property Optimization of a Series of Imidazopyridines for Visceral Leishmaniasis*, ACS Infect. Dis.* **2023**, *in press* (id-2023-000406, accepted 01-Jun-2023).
