# Supplementary material for: Open Source Antibiotics: Simple Diarylimidazoles Are Potent against Methicillin-Resistant Staphylococcus aureus
Source: ACS Infect Dis. 2023 Nov 22;9(12):2423–35. doi: 10.1021/acsinfecdis.3c00286 (PMC10714399; doi:10.1021/acsinfecdis.3c00286)
Supplement: Supplementary file 2 — id3c00286_si_005.pdf [file id3c00286_si_005.pdf]

Supporting Information

Chemical Methods

Open Source Antibiotics - Simple Diarylimidazoles are Potent Against Methicillin Resistant Staphylococcus Aureus.

Dana M. Klug[1], Edwin G. Tse[1], Daniel G. Silva[1,2], Yafeng Cao[3], Susan A. Charman[4], Jyoti Chauhan[5], Elly Crighton[4], Maria Dichiara[5], Chris Drake[6], David Drewry[7,8], Flavio da Silva Emery[2], Lori Ferrins[5], Lee Graves[9], Emily Hopkins[6], Thomas A. C. Kresina[5], Álvaro Lorente-Macías[9,10,11], Benjamin Perry[12], Richard Phipps[6], Bruno Quiroga[5], Antonio Quotadamo[5,13], Giada N. Sabatino[1], Anthony Sama[14], Andreas Schätzlein[1], Quillon J. Simpson[5], Jonathan Steele[6], Julia Shanu-Wilson[6], Peter Sjö[12], Paul Stapleton[1], Christopher J. Swain[15], Alexandra Vaideanu[1], Huanxu Xie[3], William Zuercher[7], Matthew H. Todd[1,16*]

[1] School of Pharmacy, University College London, 29-39 Brunswick Square, London WC1N 1AX, United Kingdom.

[2] School of Pharmaceutical Sciences of Ribeirão Preto, University of São Paulo, Ribeirão Preto, São Paulo, 14040-903 Brazil.

[3] WuXi AppTec Company Ltd., 666 Gaoxin Road, East Lake High-Tech Development Zone, Wuhan 430075, People's Republic of China.

[4] Centre for Drug Candidate Optimisation, Monash Institute of Pharmaceutical Sciences, Monash University, Parkville, VIC 3052, Australia.

[5] Department of Chemistry and Chemical Biology, Northeastern University, Boston, Massachusetts 02115, United States of America.

[6] Hypha Discovery, 154b Brook Dr, Milton, Abingdon OX14 4SD, United Kingdom.

[7] UNC Lineberger Comprehensive Cancer Center, School of Medicine, University of North Carolina at Chapel Hill, Chapel Hill, NC, 27599, USA

[8] Structural Genomics Consortium, UNC Eshelman School of Pharmacy, University of North Carolina at Chapel Hill, Chapel Hill, NC, 27599, USA

[9] Department of Pharmacology, University of North Carolina at Chapel Hill, Chapel Hill, NC 27599, USA

[10] Department of Medicinal & Organic Chemistry and Excellence Research Unit of "Chemistry Applied to Biomedicine and the Environment", Faculty of Pharmacy, University of Granada, Campus de Cartuja s/n, 18071 Granada, Spain

[11] A. L-M. Present address: Cancer Research UK Edinburgh Centre, Institute of Genetics & Cancer, University of Edinburgh, Edinburgh EH4 2XR, United Kingdom

[12] Drugs for Neglected Diseases *initiative* (DND*i*), 15 Chemin Camille-Vidart, 1202 Geneva, Switzerland

[13] *Clinical and Experimental Medicine PhD Program, University of Modena and Reggio Emilia, 41121 Modena, Italy*

[14] Citizen scientist.

[15] Cambridge MedChem Consulting, 8 Mangers Lane, Duxford, Cambridge CB22 4RN, United Kingdom

[16] Structural Genomics Consortium, University College London, 29-39 Brunswick Square, London WC1N 1AX, United Kingdom

**Corresponding author: matthew.todd@ucl.ac.uk*

Table of Contents

MOLECULE NUMBERING CONVENTION S2
ONLINE LABORATORY NOTEBOOKS S2
MATERIALS AND METHODS S3
GENERAL PROCEDURES S3
EXPERIMENTAL PROCEDURES S7
HYPHA METABOLISM SCREEN S70
^{1}H AND ^{13}C NMR SPECTRA OF FINAL COMPOUNDS S71
REFERENCES S160

Molecule Numbering Convention

Molecules in Open Source Antibiotics are numbered using the convention **OSA_000123_XX_YY** where the main number is unique to a molecule, the **XX** refers to the salt form and the last two digits (**YY**) are the batch number. The salt codes are XX = No salt (free base); CL = HCl salt; FA = Formic acid salt; TF = TFA salt.

If the free base **OSA_000123_XX_01** were made again as the HCl salt, its code would be **OSA_000123_CL_01**, whereas if it were made again as the free base its code would be **OSA_000123_XX_02** and so on.

This convention was discussed in https://github.com/opensourceantibiotics/murligase/issues/9.

The OSA Compound Master List (https://docs.google.com/spreadsheets/d/1fAxwae9W--0BLCLU1KIGdXcVGvxiLiO7VxjKoEO2XHE/edit?usp=sharing) can be used to search for molecules and data.

Online Laboratory Notebooks

All chemical data in this project were recorded in openly available laboratory notebooks (see https://github.com/opensourceantibiotics/Series-2-Diarylimidazoles/wiki/Submissions%2C-Resources%2C-and-Data#lab-notebooks). To ensure their continued availability, offline copies have been archived at UCL's e-Repository at https://doi.org/10.5522/04/21749750.

Materials and Methods

OSA_001017 and **OSA_001016** were purchased from Chemspace and evaluated as provided. **OSA_000841**, **OSA_000840**, **OSA_000847**, **OSA_000845**, **OSA_000842**, **OSA_000850**, **OSA_000851**, **OSA_000839**, **OSA_000844**, **OSA_000848**, **OSA_000843**, **OSA_000852**, **OSA_000856** were synthesized as reported in the literature.[1] **OSA_000859** was synthesized as reported in the literature.[2] **OSA_001022**, **OSA_000979**, **OSA_000984**, **OSA_000982**, **OSA_000981**, **OSA_000980**, **OSA_000983**, **OSA_000998**, **OSA_000999**, **OSA_001000**, **OSA_001021**, **OSA_001020**, **OSA_001019**, **OSA_001007**, **OSA_001006**, **OSA_001005**, **OSA_001004**, **OSA_001003**, **OSA_001002**, **OSA_001001**, **OSA_001024**, **OSA_001023**, **OSA_001025**, **OSA_001034**, **OSA_001035**, **OSA_001036**, **OSA_001037**, **OSA_001038**, **OSA_001039**, **OSA_001040**, **OSA_001107**, **OSA_001102**, **OSA_001104**, **OSA_001105**, **OSA_001106** and **OSA_001103** were synthesized as reported in the literature.[3]

General Procedures

General Procedure A (Cyclisation to imidazole)

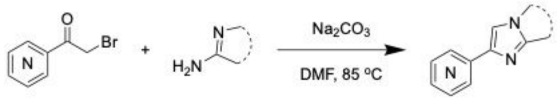

α-Bromoketone (1.00 equiv.), amine (1.50 equiv.) and Na_2CO_3 (4.02 equiv.) were stirred in DMF (0.15 M) at 85 °C until completion as indicated by TLC. The reaction was cooled to rt and partitioned between CH_2Cl_2 and H_2O. The aqueous layer was separated and extracted with CH_2Cl_2 (3 x) and the combined organic layers washed with brine, dried ($MgSO_4$), filtered and concentrated under reduced pressure to give the crude product which was purified by flash chromatography on silica to give the corresponding imidazole product.

General Procedure B (Cyclisation to Imidazole 2)

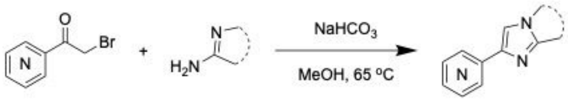

Amine (1.00 equiv.) and $NaHCO_3$ (1.05 equiv.) were stirred in MeOH (0.15 M) under Ar and α-bromoketone (1.05 equiv.) was added slowly at rt. The reaction was heated at reflux until completion as indicated by TLC. The reaction was cooled to rt, diluted with H_2O and the organic solvent was removed under reduced pressure. The aqueous layer was extracted with EtOAc, dried (Na_2SO_4), filtered and concentrated under reduced pressure to give the crude product which was purified by flash chromatography on silica to give the corresponding imidazole product.

General Procedure C (Bromination)

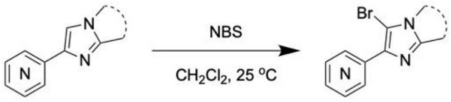

To the product from General Procedure A or B (1 equiv.) dissolved in CH_2Cl_2 (0.25 M), was added NBS (1 equiv.) and the reaction stirred at rt until completion as indicated by TLC. The solvent was removed and the residue dissolved in EtOAc, washed with a sat. aq. $NaHCO_3$ solution, brine, dried ($MgSO_4$), filtered and concentrated under reduced pressure to give the corresponding brominated product that was used without further purification unless otherwise stated.

General Procedure D (Suzuki Coupling)

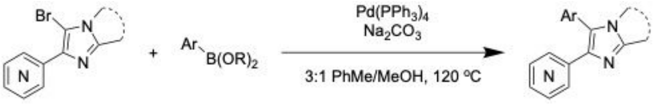

A microwave vial was charged with the product from General Procedure C (1.00 equiv.), boronic ester/acid (1.30 equiv.) and $Pd(PPh_3)_4$ (0.12 equiv.). The vial was closed with a crimp seal and a 3:1 v/v mixture of PhMe/EtOH (0.9 mL) was added, followed by 2 M Na_2CO_3 (0.5 mL). The vial was degassed with Ar then the mixture heated conventionally at 120 °C overnight or in a microwave at 120 °C for 30 min. The reaction was cooled to rt, diluted with CH_2Cl_2 and washed with brine, dried ($MgSO_4$), filtered and concentrated under reduced pressure; or alternatively, the reaction mixture was diluted with MeOH, filtered through a pad of celite and concentrated under reduced pressure to give the crude product that was purified by one, or a combination of, the following methods to give the corresponding Suzuki product:

1. flash chromatography on silica (1-10% MeOH in CH_2Cl_2)
2. reversed-phase flash chromatography on silica (5-100% MeOH in H_2O)
3. trituration with 1:10 EtOH/hexanes

General Procedure E (Suzuki coupling 2)

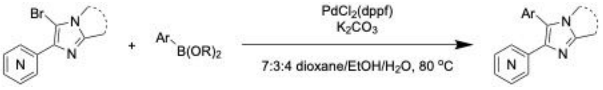

A microwave vial was charged with the product from General Procedure C (1.00 equiv.), boronic ester/acid (1.10 – 1.50 equiv.), $PdCl_2$(dppf) (0.05 equiv.) and K_2CO_3 (2.00 equiv.). A 7:3:4 mixture of 1,4-dioxane/EtOH/H_2O (5 mL) and the vial closed with a crimp seal. The mixture was heated at 80 °C until completion as indicated by LCMS. The reaction was cooled to rt, diluted with MeOH, filtered through a pad of celite and concentrated under reduced pressure to give the crude product that was purified by flash chromatograph on silica to give the corresponding Suzuki product.

General Procedure F (Miyaura borylation)

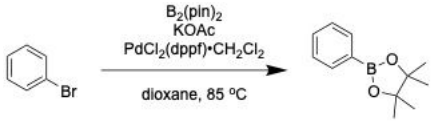

A microwave vial was charged with bromide (1.00 equiv.), bis(pinacolato)diboron) (1.49 equiv.), KOAc (3.00 equiv.) and $PdCl_2$(dppf)•CH_2Cl_2 (0.05 equiv.). The vial was closed with a crimp seal and the vial backfilled with Ar three times. Anhydrous 1,4-dioxane (0.15 M) was added and the reaction heated at 85 °C overnight. The reaction was cooled to rt, diluted with EtOAc, filtered through celite and concentrated under reduced pressure to give the crude product that was purified by flash chromatography on silica to give the corresponding borylation product.

General Procedure G (Mannich Reaction)

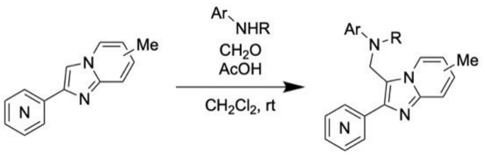

A solution of imidazopyridine (1.0 mmol) and the appropriate amine (1.2 mmol) in CH_2Cl_2 (10 mL) was added AcOH (0.8 mL) and formaldehyde (37% in H_2O, 0.2 mL). The reaction mixture was stirred at rt overnight then quenched with H_2O (16.0 mL) and extracted with CH_2Cl_2. The solvent was removed under reduced pressure to give the crude product that was purified by flash chromatography on silica to give the corresponding Mannich product.

General Procedure H (*N*-Arylation of Benzimidazole)

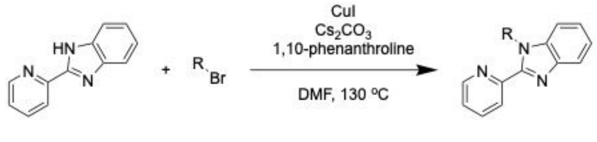

A microwave vial was charged with 2-(pyridin-2-yl)-1*H*-benzo[*d*]imidazole (1.00 equiv.), bromide (1.60 – 3.20 equiv.), 1,10-phenanthroline (0.63 equiv.), Cs_2CO_3 (1.50 equiv.) and CuI (0.33 equiv.). The vial was closed with a crimp seal and anhydrous DMF (0.5 M) was added. The vial was degassed with Ar and the mixture heated at 130 °C overnight. The mixture was cooled to rt, diluted with EtOAc and washed with H_2O, brine, dried ($MgSO_4$), filtered and concentrated under reduced pressure to give the crude product that was purified by flash chromatography on silica to give the corresponding *N*-arylated benzimidazole product.

Experimental Procedures

2-(Pyridin-2-yl)-6,7-dihydro-5*H*-pyrrolo[1,2-*a*]imidazole (S1)

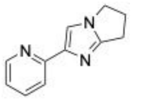

Prepared according to General Procedure A from: 2-bromo-1-(pyridine-2-yl)ethan-1-one hydrobromide (10.0 g, 35.6 mmol) and 3,4-dihydro-2*H*-pyrrol-5-amine hydrochloride (6.44 g, 53.4 mmol); purified by flash chromatography on silica (1-10% MeOH in CH_2Cl_2) to give *the title compound* as an orange powder (2.84 g, 43%); **^{1}H NMR** (500 MHz, $CDCl_3$): δ 8.79 – 8.21 (m, 1H), 7.92 (d, *J* = 8.0 Hz, 1H), 7.67 (td, *J* = 7.8, 1.8 Hz, 1H), 7.56 (s, 1H), 7.09 (ddd, *J* = 7.4, 4.9, 1.1 Hz, 1H), 4.19 – 3.69 (m, 2H), 3.29 – 2.80 (m, 2H), 2.63 (p, *J* = 7.4 Hz, 2H); **^{13}C NMR** (126 MHz, $CDCl_3$): δ 155.1, 153.9, 149.3, 146.7, 136.7, 121.4, 119.0, 113.5, 45.0, 26.3, 23.2; ***m/z*** (ESI+) 186 ($[M+H]^+$, 100%).

3-Bromo-2-(pyridin-2-yl)-6,7-dihydro-5*H*-pyrrolo[1,2-*a*]imidazole (S2)

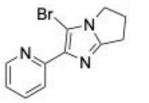

Prepared according to General Procedure C from: **S1** (2.50 g, 13.5 mmol) to give *the title compound* as a brown powder (3.32 g, 93%); **^{1}H NMR** (500 MHz, $CDCl_3$): δ 8.89 – 8.33 (m, 1H), 7.95 (d, *J* = 8.0 Hz, 1H), 7.69 (td, *J* = 7.8, 1.8 Hz, 1H), 7.14 (ddd, *J* = 7.4, 4.9, 1.1 Hz, 1H), 4.14 – 3.66 (m, 2H), 3.27 – 2.85 (m, 2H), 2.64 (p, *J* = 7.4 Hz, 2H); **^{13}C**

NMR (126 MHz, $CDCl_3$): δ 154.2, 153.0, 149.3, 141.5, 136.4, 121.6, 120.7, 96.8, 44.7, 25.4, 24.4; ***m/z*** (ESI+) 264 ($[M+H]^+$, 100%).

2-(Benzo[*b*]thiophen-5-yl)-4,4,5,5-tetramethyl-1,3,2-dioxaborolane (S3)

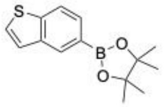

Prepared according to General Procedure F from: 5-bromobenzo[*b*]thiophene (1.00 g, 4.70 mmol); purified by flash chromatography on silica (12-100% EtOAc in hexanes) to give *the title compound* as a white powder (1.04 g, 86%). **^{1}H NMR** (500 MHz, $CDCl_3$): δ 8.31 (s, 1H), 7.89 (d, *J* = 8.1 Hz, 1H), 7.75 (d, *J* = 8.1 Hz, 1H), 7.42 (d, *J* = 5.4 Hz, 1H), 7.35 (d, *J* = 5.4 Hz, 1H), 1.38 (s, 12H); ***m/z*** (ESI+) 261 ($[M+H]^+$, 100%).

3-(Benzo[*b*]thiophen-5-yl)-2-(pyridin-2-yl)-6,7-dihydro-5*H*-pyrrolo[1,2-*a*]imidazole (OSA_000822)

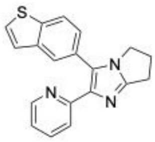

Prepared according to General Procedure D from: **S2** (125 mg, 0.47 mmol) and **S3** (144 mg, 0.55 mmol); purified by methods 1, then 3, to give *the title compound* as a tan powder (96.8 mg, 65%); **^{1}H NMR** (500 MHz, $CDCl_3$): δ 8.46 (dt, *J* = 4.7, 1.4 Hz, 1H), 8.20 – 7.75 (m, 2H), 7.58 (d, *J* = 8.0 Hz, 1H), 7.52 (td, *J* = 7.7, 1.9 Hz, 1H), 7.48 (d, *J* = 5.4 Hz, 1H), 7.39 (dd, *J* = 8.3, 1.7 Hz, 1H), 7.32 (d, *J* = 5.4 Hz, 1H), 7.03 (ddd, *J* = 7.4, 4.9, 1.3 Hz, 1H), 3.97 (t, *J* = 7.0 Hz, 2H), 3.02 (t, *J* = 7.6 Hz, 2H), 2.63 (p, *J* = 7.3 Hz, 2H); **^{13}C NMR** (126 MHz, $CDCl_3$): δ 154.3, 154.1, 149.4, 141.4, 139.9, 139.4, 136.0, 128.0, 127.3, 127.2, 125.9, 124.14, 124.10, 122.6, 121.4, 121.2, 44.6, 26.3, 23.7; ***m/z*** (ESI+) 318 ($[M+H]^+$, 100%); **HRMS** (ESI+) found 318.1057 ($[M+H]^+$), $C_{19}H_{16}N_3S^+$ requires 318.1059.

3-(Benzofuran-6-yl)-2-(pyridin-2-yl)-6,7-dihydro-5*H*-pyrrolo[1,2-*a*]imidazole (OSA_000829)

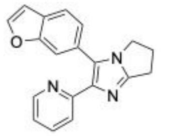

Prepared according to General Procedure D from: **S2** (80.0 mg, 0.30 mmol) and benzofuran-6-ylboronic acid (59 mg, 0.36 mmol); purified by methods 1 (60-90% EtOAc in hexane), then 3, to give *the title compound* as a pale yellow solid (54.0 mg, 59%); **^{1}H NMR** (400 MHz, $CDCl_3$): δ 8.50 – 8.42 (m, 1H), 7.64 (d, *J* = 2.2 Hz, 1H), 7.62 – 7.55 (m, 3H), 7.52 (td, *J* = 7.7, 1.9 Hz, 1H), 7.28 (dd, *J* = 8.1, 1.5 Hz, 1H), 7.02 (ddd, *J* = 7.4, 4.8, 1.3 Hz, 1H), 6.78 (d, *J* = 2.0 Hz, 1H), 3.96 (t, *J* = 7.1 Hz, 2H), 3.01 (t, *J* = 7.6 Hz, 2H), 2.61 (p, *J* = 7.3 Hz, 2H); **^{13}C NMR** (101 MHz, $CDCl_3$): δ 154.9, 153.96, 153.95, 149.3, 145.8, 141.0, 136.0, 128.0, 127.2, 127.1, 124.4, 121.5, 121.2, 121.1, 112.2, 106.7, 44.7, 26.2, 23.7; ***m/z*** (ESI+) 302 ($[M+H]^+$, 100%); **HRMS** (ESI+) found 302.1293 ($[M+H]^+$), $C_{19}H_{16}N_3O^+$ requires 302.1288.

2-(Benzofuran-5-yl)-4,4,5,5-tetramethyl-1,3,2-dioxaborolane (S4)

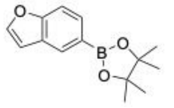

Prepared according to General Procedure 4 from: 5-bromobenzofuran (500 mg, 2.54 mmol); purified by flash chromatography on silica (12-100% EtOAc in hexanes) to give *the title compound* as a pale yellow powder (529 mg, 85%); **^{1}H NMR** (400 MHz, $CDCl_3$): δ 8.11 (s, 1H), 7.75 (d, *J* = 8.3 Hz, 1H), 7.61 (s, 1H), 7.50 (d, *J* = 8.6 Hz, 1H), 6.76 (s, 1H), 1.37 (s, 12H); ***m/z*** (ESI+) 245 ($[M+H]^+$, 100%).

3-(Benzofuran-5-yl)-2-(pyridin-2-yl)-6,7-dihydro-5*H*-pyrrolo[1,2-*a*]imidazole (OSA_000821)

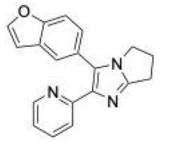

Prepared according to General Procedure D from: **S2** (150 mg, 0.57 mmol) and **S4** (180 mg, 0.74 mmol); purified by methods 1, then 3, to give *the title compound* as a light brown powder (75.3 mg, 44%); **^{1}H NMR** (500 MHz, $CDCl_3$): δ 8.55 – 8.35 (m, 1H), 7.66 (d, *J* = 1.8 Hz, 2H), 7.60 – 7.45 (m, 3H), 7.35 (dd, *J* = 8.5, 1.6 Hz, 1H), 7.02 (ddd, *J* = 6.5, 4.9, 1.4 Hz, 1H), 6.85 – 6.67 (m, 1H), 3.95 (t, *J* = 7.0 Hz, 2H), 3.06 – 2.90 (m,

2H), 2.63 (p, *J* = 7.4 Hz, 2H); **^{13}C NMR** (126 MHz, $CDCl_3$): δ 154.7, 154.4, 153.8, 149.4, 145.7, 141.2, 135.9, 128.2, 127.8, 126.2, 125.9, 122.1, 121.4, 121.1, 111.6, 106.9, 44.5, 26.3, 23.7; ***m/z*** (ESI+) 302 ([M+H]$^+$, 100%); **HRMS** (ESI+) found 302.1284 ([M+H]$^+$), $C_{19}H_{16}N_3O^+$ requires 302.1288.

3-(Benzo[*b*]thiophen-6-yl)-2-(pyridin-2-yl)-6,7-dihydro-5*H*-pyrrolo[1,2-*a*]imidazole (OSA_000830)

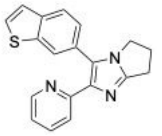

Prepared according to General Procedure D from: **S2** (80.0 mg, 0.30 mmol) and 2-(benzo[*b*]thiophen-6-yl)-4,4,5,5-tetramethyl-1,3,2-dioxoborolane (95.0 mg, 0.36 mmol); purified by methods 1 (65-95% EtOAc in hexane), then 3, to give *the title compound* as a pale yellow solid (58.1 mg, 61%); **^{1}H NMR** (400 MHz, $CDCl_3$): δ 8.48 – 8.42 (m, 1H), 7.95 (s, 1H), 7.79 (d, *J* = 8.3 Hz, 1H), 7.61 (d, 1H), 7.52 (td, *J* = 7.7, 1.8 Hz, 1H), 7.46 (d, *J* = 5.4 Hz, 1H), 7.40 (dd, *J* = 8.3, 1.6 Hz, 1H), 7.33 (d, *J* = 5.4 Hz, 1H), 7.03 (ddd, *J* = 7.5, 4.8, 1.2 Hz, 1H), 3.96 (t, *J* = 7.0 Hz, 2H), 3.01 (t, *J* = 7.6 Hz, 2H), 2.61 (p, *J* = 7.3 Hz, 2H); **^{13}C NMR** (101 MHz, $CDCl_3$): δ 154.0, 153.9, 149.3, 141.1, 139.9, 139.2, 136.0, 127.8, 127.3, 127.0, 125.9, 123.8, 123.5, 123.0, 121.5, 121.3, 44.8, 26.2, 23.7; ***m/z*** (ESI+) 318 ([M+H]$^+$, 100%); **HRMS** (ESI+) found 318.1065 ([M+H]$^+$), $C_{19}H_{16}N_3S^+$ requires 318.1059.

2-(Pyridin-2-yl)-3-(*p*-tolyl)-6,7-dihydro-5*H*-pyrrolo[1,2-*a*]imidazole (OSA_000865)

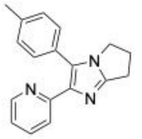

Prepared according to General Procedure D from: **S2** (50.0 mg, 0.19 mmol) and *p*-tolylboronic acid (33.5 mg, 0.25 mmol); purified by methods 1, then 3, to give *the title compound* as a white powder (15.9 mg, 31%); **^{1}H NMR** (500 MHz, $CDCl_3$): δ 8.49 (d, *J* = 4.7 Hz, 1H), 7.65 – 7.45 (m, 2H), 7.32 (d, *J* = 8.1 Hz, 2H), 7.19 (d, *J* = 7.9 Hz, 2H), 7.03 (ddd, *J* = 6.8, 4.9, 1.5 Hz, 1H), 3.94 (t, *J* = 7.0 Hz, 2H), 3.58 – 2.81 (m, 2H), 2.73 – 2.48 (m, 2H), 2.39 (s, 3H); **^{13}C NMR** (126 MHz, $CDCl_3$): δ 154.4, 153.9, 149.4, 141.2, 137.8, 135.9,

129.3, 129.2, 128.2, 128.0, 121.5, 121.1, 44.6, 26.3, 23.7, 21.5; ***m/z*** (ESI+) 276 ([M+H]$^+$, 100%); **HRMS** (ESI+) found 276.1496 ([M+H]$^+$), $C_{18}H_{18}N_3^+$ requires 276.1495.

3-(4-(Methylthio)phenyl)-2-(pyridin-2-yl)-6,7-dihydro-5*H*-pyrrolo[1,2-*a*]imidazole (OSA_000824)

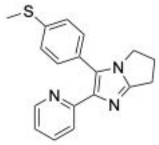

Prepared according to General Procedure D from: **S2** (80.0 mg, 0.30 mmol) and (4-(methylthio)phenyl)boronic acid (61.0 mg, 0.36 mmol); purified by methods 1 (65-90% EtOAc in hexane), then 3, to give *the title compound* as a pale yellow solid (23.6 mg, 26%); **^{1}H NMR** (400 MHz, $CDCl_3$): δ 8.48 (d, *J* = 4.9 Hz, 1H), 7.66 (d, *J* = 8.1 Hz, 1H), 7.58 (td, *J* = 7.7, 1.9 Hz, 1H), 7.36 (d, *J* = 8.0 Hz, 2H), 7.25 (d, *J* = 7.9 Hz, 2H), 7.11 – 7.03 (m, 1H), 3.97 (t, *J* = 7.1 Hz, 2H), 3.07 (t, *J* = 7.6 Hz, 2H), 2.64 (p, *J* = 7.4 Hz, 2H), 2.51 (s, 3H); **^{13}C NMR** (101 MHz, $CDCl_3$): δ 153.8, 153.4, 149.2, 140.3, 138.8, 136.2, 129.7, 127.6, 127.1, 127.0, 126.3, 121.5, 44.9, 26.2, 23.7, 15.6; ***m/z*** (ESI+) 308 ([M+H]$^+$, 100%); **HRMS** (ESI+) found 308.1220 ([M+H]$^+$), $C_{18}H_{18}N_3S^+$ requires 308.1216.

3-(4-Chlorophenyl)-2-(pyridin-2-yl)-6,7-dihydro-5*H*-pyrrolo[1,2-*a*]imidazole (OSA_000875)

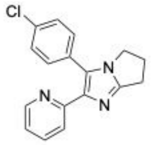

Prepared according to General Procedure D from: **S2** (50.0 mg, 0.19 mmol) and (4-chlorophenyl)boronic acid (38.5 mg, 0.25 mmol); purified by methods 1, then 2, to give *the title compound* as a pale yellow powder (5.5 mg, 10%); insufficient material remaining for complete characterization; **^{1}H NMR** (500 MHz, $CDCl_3$): δ 8.45 (d, *J* = 4.3 Hz, 1H), 7.74 (d, *J* = 6.8 Hz, 1H), 7.63 (t, *J* = 7.1 Hz, 1H), 7.40 (d, *J* = 8.8 Hz, 2H), 7.37 (d, *J* = 8.7 Hz, 2H), 7.09 (t, *J* = 6.4 Hz, 1H), 3.98 (t, *J* = 7.2 Hz, 2H), 3.08 (br s, 2H), 2.67 (p, *J* = 7.2 Hz, 2H); ***m/z*** (ESI+) 296 ([M+H]$^+$, 100%); **HRMS** (ESI+) found 296.0946 ([M+H]$^+$), $C_{17}H_{15}ClN_3^+$ requires 296.0949.

4-(2-(Pyridin-2-yl)-6,7-dihydro-5*H*-pyrrolo[1,2-*a*]imidazol-3-yl)benzonitrile (OSA_000975)

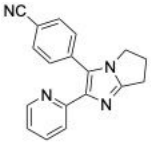

Prepared according to General Procedure D from: **S2** (50.0 mg, 0.19 mmol) and 4-cyanobenzeneboronic acid (36.2 mg, 0.25 mmol); purified by methods 1, then 3, to give *the title compound* as a white powder (13.7 mg, 25%); **^{1}H NMR** (500 MHz, $CDCl_3$): δ 8.40 (d, *J* = 4.6 Hz, 1H), 7.87 (d, *J* = 7.7 Hz, 1H), 7.70 (br s, 1H), 7.67 (d, *J* = 8.4 Hz, 2H), 7.59 (d, *J* = 8.5 Hz, 2H), 7.14 (t, *J* = 5.8 Hz, 1H), 4.03 (t, *J* = 7.2 Hz, 2H), 3.10 (br s, 2H), 2.70 (p, *J* = 7.0 Hz, 2H); **^{13}C NMR** (101 MHz, $CDCl_3$): δ 155.2, 153.9, 149.1, 143.0, 136.5, 136.0, 132.1, 129.8, 126.1, 121.9, 121.6, 119.0, 111.1, 45.1, 26.3, 23.7; ***m/z*** (ESI+) 287 ($[M+H]^+$, 100%); **HRMS** (ESI+) found 287.1289 ($[M+H]^+$), $C_{18}H_{15}N_4^+$ requires 287.1291.

3-(3-(Methylthio)phenyl)-2-(pyridin-2-yl)-6,7-dihydro-5*H*-pyrrolo[1,2-*a*]imidazole (OSA_000826)

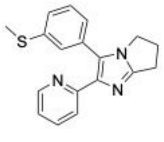

Prepared according to General Procedure D from: **S2** (80.0 mg, 0.30 mmol) and (3-(methylthio)phenyl)boronic acid (61.0 mg, 0.36 mmol); purified by methods 1 (65-90% EtOAc in hexane), then 3, to give *the title compound* as a yellow solid (25.9 mg, 32%); **^{1}H NMR** (400 MHz, $CDCl_3$): δ 8.47 (ddd, *J* = 4.8, 1.9, 1.0 Hz, 1H), 7.66 (dt, *J* = 8.0, 1.1 Hz, 1H), 7.58 (ddd, *J* = 8.0, 7.3, 1.8 Hz, 1H), 7.38 – 7.33 (m, 1H), 7.33 – 7.27 (m, 1H), 7.23 (ddd, *J* = 7.9, 2.0, 1.2 Hz, 1H), 7.18 (dt, *J* = 7.5, 1.5 Hz, 1H), 7.07 (ddd, *J* = 7.4, 4.9, 1.3 Hz, 1H), 3.98 (t, 2H), 3.09 – 3.00 (m, 2H), 2.71 – 2.58 (m, 2H), 2.42 (s, 3H); **^{13}C NMR** (101 MHz, $CDCl_3$): δ 154.0, 153.3, 149.2, 140.6, 138.9, 136.3, 131.2, 128.9, 127.5, 127.3, 126.3, 125.8, 121.7, 121.6, 45.1, 26.2, 23.7, 15.8; ***m/z*** (ESI+) 308 ($[M+H]^+$, 100%); **HRMS** (ESI+) found 308.1221 ($[M+H]^+$), $C_{18}H_{18}N_3S^+$ requires 308.1216.

3-(2,3-Dihydrobenzo[*b*][1,4]dioxin-6-yl)-2-(pyridin-2-yl)-6,7-dihydro-5*H*-pyrrolo[1,2-*a*]imidazole (OSA_000819)

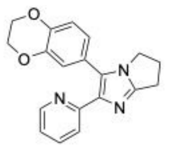

Prepared according to General Procedure D from: **S2** (70.0 mg, 0.27 mmol) and (2,3-dihydrobenzo[*b*][1,4]dioxin-6-yl)boronic acid (57.0 mg, 0.32 mmol); purified by methods 1 (60-95% EtOAc in hexane), then 3, to give *the title compound* as a pale yellow solid (28.9 mg, 34%); ^{1}**H NMR** (400 MHz, $CDCl_3$): δ 8.50 (d, J = 4.8 Hz, 1H), 7.60 (d, J = 7.9 Hz, 1H), 7.55 (td, J = 7.7, 1.9 Hz, 1H), 7.08 – 7.00 (m, 1H), 6.94 (d, J = 2.0 Hz, 1H), 6.92 – 6.82 (m, 2H), 4.31 – 4.23 (m, 4H), 3.94 (t, J = 7.0 Hz, 2H), 3.01 (t, J = 7.5 Hz, 2H), 2.61 (p, J = 7.3 Hz, 2H); ^{13}**C NMR** (101 MHz, $CDCl_3$): δ 153.62, 153.56, 149.3, 143.7, 143.5, 140.2, 136.1, 127.5, 123.8, 122.8, 121.5, 121.3, 118.1, 117.5, 64.5, 64.4, 44.8, 26.2, 23.7; ***m/z*** (ESI+) 320 ($[M+H]^+$, 100%); **HRMS** (ESI+) found 320.1397 ($[M+H]^+$), $C_{19}H_{18}N_3O_2^+$ requires 320.1394.

3-(Dibenzo[*b*,*d*]thiophen-2-yl)-2-(pyridin-2-yl)-6,7-dihydro-5*H*-pyrrolo[1,2-*a*]imidazole (OSA_000831)

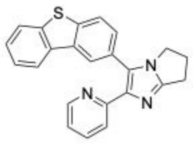

Prepared according to General Procedure D from: **S2** (50.0 mg, 0.19 mmol) and dibenzo[*b*,*d*]thiophen-2-ylboronic acid (56.1 mg, 0.25 mmol); purified by methods 1, then 3, to give *the title compound* as a light brown powder (19.4 mg, 28%); ^{1}**H NMR** (500 MHz, $CDCl_3$): δ 8.52 – 8.35 (m, 1H), 8.31 – 8.18 (m, 1H), 8.14 – 7.96 (m, 1H), 7.93 – 7.80 (m, 2H), 7.66 (d, J = 8.0 Hz, 1H), 7.54 (ddd, J = 14.8, 8.0, 1.7 Hz, 2H), 7.46 (pd, J = 7.1, 1.2 Hz, 2H), 7.10 – 6.86 (m, 1H), 4.02 (t, J = 7.0 Hz, 2H), 3.44 – 2.93 (m, 2H), 2.67 (p, J = 7.4 Hz, 2H); ^{13}**C NMR** (126 MHz, $CDCl_3$): δ 154.3, 154.2, 149.4, 141.6, 139.9, 139.1, 136.1, 135.9, 135.5, 128.2, 127.9, 127.5, 127.1, 124.7, 123.1, 122.9, 122.3, 121.7, 121.4, 121.3, 44.7, 26.4, 23.7; ***m/z*** (ESI+) 368 ($[M+H]^+$, 100%); **HRMS** (ESI+) found 368.1212 ($[M+H]^+$), $C_{23}H_{18}N_3S^+$ requires 368.1216.

2-(Benzo[*b*]thiophen-3-yl)-4,4,5,5-tetramethyl-1,3,2-dioxaborolane (S5)

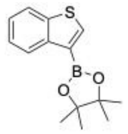

Prepared according to General Procedure F from: 3-bromobenzo[*b*]thiophene (100 mg, 0.47 mmol); purified by flash chromatography on silica (0-20% EtOAc in hexanes) to give *the title compound* as a pale yellow solid (42.9 mg, 35%); **^{1}H NMR** (500 MHz, $CDCl_3$): δ 8.37 (d, *J* = 8.0 Hz, 1H), 8.07 (s, 1H), 7.89 (d, *J* = 8.0 Hz, 1H), 7.42 – 7.38 (m, 1H), 7.36 – 7.31 (m, 1H), 1.39 (s, 12H); **^{13}C NMR** (126 MHz, $CDCl_3$): δ 142.9, 140.9, 139.2, 125.5, 124.4, 124.3, 122.3, 83.7, 25.1. Spectroscopic data matched those in the literature.[4]

3-(Benzo[*b*]thiophen-3-yl)-2-(pyridin-2-yl)-6,7-dihydro-5*H*-pyrrolo[1,2-*a*]imidazole (OSA_000832)

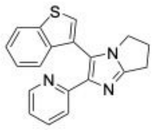

Prepared according to General Procedure D from: **S2** (25.0 mg, 0.09 mmol) and **S5** (32.0 mg, 0.12 mmol); purified by methods 1, then 2, to give *the title compound* as a yellow powder (8.80 mg, 29%); **^{1}H NMR** (500 MHz, $CDCl_3$): δ 8.40 (d, *J* = 4.7 Hz, 1H), 7.90 (d, *J* = 8.1 Hz, 1H), 7.55 (s, 1H), 7.44 (dt, *J* = 4.0, 1.5 Hz, 2H), 7.40 (d, *J* = 8.1 Hz, 1H), 7.35 (t, *J*= 7.5 Hz, 1H), 7.27 – 7.17 (m, 1H), 6.97 (td, *J* = 5.1, 3.1 Hz, 1H), 3.86 (t, *J* = 7.1 Hz, 2H), 3.04 (t, *J* = 7.6 Hz, 2H), 2.63 (p, *J* = 7.4 Hz, 2H); **^{13}C NMR** (126 MHz, $CDCl_3$): δ 154.6, 153.8, 149.5, 143.3, 140.0, 138.0, 135.9, 127.0, 126.5, 124.7, 124.4, 123.6, 122.9, 121.3, 121.1, 120.9, 44.4, 26.2, 23.8; ***m/z*** (ESI+) 318 ($[M+H]^+$, 100%); **HRMS** (ESI+) found 318.1069 ($[M+H]^+$), $C_{19}H_{16}N_3S^+$ requires 318.1059.

3-(4-Methoxyphenyl)-2-(pyridin-2-yl)-6,7-dihydro-5*H*-pyrrolo[1,2-*a*]imidazole (OSA_000823)

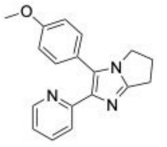

Prepared according to General Procedure D from: **S2** (70.0 mg, 0.27 mmol) and (4-methoxyphenyl)boronic acid (48.0 mg, 0.32 mmol); purified by methods 1 (65-95% EtOAc in hexane), then 3, to give *the title compound* as a pale yellow solid (28 mg, 36%); **^{1}H NMR** (400 MHz, $CDCl_3$): δ 8.48 (d, J = 4.6 Hz, 1H), 7.58 (d, J = 7.9 Hz, 1H), 7.53 (td, J = 7.6, 1.8 Hz, 1H), 7.35 (d, J = 8.6 Hz, 2H), 7.02 (ddd, J = 6.8, 4.8, 1.4 Hz, 1H), 6.91 (d, J = 8.6 Hz, 2H), 3.93 (t, J = 7.1 Hz, 2H), 3.83 (s, 3H), 3.00 (t, J = 7.5 Hz, 2H), 2.61 (p, J = 7.3 Hz, 2H); **^{13}C NMR** (101 MHz, $CDCl_3$): δ 159.5, 153.8, 153.6, 149.3, 140.2, 136.0, 130.7, 127.8, 123.1, 121.4, 121.2, 114.1, 55.4, 44.6, 26.2, 23.7; ***m/z*** (ESI+) 292 ([M+H]$^+$, 100%); **HRMS** (ESI+) found 292.1449 ([M+H]$^+$), $C_{18}H_{18}N_3O^+$ requires 292.1444.

2-(Pyridin-2-yl)-3-(4-(trifluoromethoxy)phenyl)-6,7-dihydro-5*H*-pyrrolo[1,2-*a*]imidazole (OSA_000868)

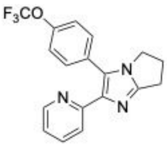

Prepared according to General Procedure D from: **S2** (100 mg, 0.38 mmol) and 4-(trifluoromethoxy)benzeneboronic acid (101 mg, 0.49 mmol); purified by methods 1, then 3, to give *the title compound* as a white powder (4.5 mg, 3%); **^{1}H NMR** (500 MHz, $CDCl_3$): δ 8.44 (d, J = 4.6 Hz, 1H), 7.70 (d, J = 7.9 Hz, 1H), 7.65 – 7.57 (m, 1H), 7.49 (d, J = 8.6 Hz, 2H), 7.22 (d, J = 8.3 Hz, 2H), 7.15 – 7.03 (m, 1H), 3.97 (t, J = 7.0 Hz, 2H), 3.01 (t, J = 7.6 Hz, 2H), 2.65 (p, J = 7.4 Hz, 2H); **^{13}C NMR** (126 MHz, $CDCl_3$): δ 154.4, 154.2, 149.2, 148.8, 141.9, 136.2, 130.8, 129.9, 126.6, 121.5, 121.4, 120.8, 44.8, 26.3, 23.7 (one obscured signal); ***m/z*** (ESI+) 346 ([M+H]$^+$, 100%); **HRMS** (ESI+) found 346.1160 ([M+H]$^+$), $C_{18}H_{15}F_3N_3O^+$ requires 346.1162.

2-(Pyridin-2-yl)-3-(4-(trifluoromethyl)phenyl)-6,7-dihydro-5*H*-pyrrolo[1,2-*a*]imidazole (OSA_001052)

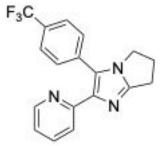
Prepared according to General Procedure E from: **S2** (25.0 mg, 0.09 mmol) and 4-(trifluoromethyl)phenylboronic acid (19.8 mg, 0.10 mmol); purified by flash chromatography on silica (1-10% MeOH in CH_2Cl_2), followed by reversed-phase flash chromatography on silica (5-100% MeOH in H_2O) to give *the title compound* as a brown solid (7.70 mg, 26%); **^{1}H NMR** (400 MHz, $CDCl_3$): δ 8.35 (d, *J* = 4.7 Hz, 1H), 7.67 (d, *J* = 8.0 Hz, 1H), 7.56 (d, *J* = 8.3 Hz, 2H), 7.51 (d, *J* = 8.4 Hz, 2H), 7.37 (m, 1H), 7.02 (t, 1H), 3.92 (t, *J* = 7.1 Hz, 2H), 2.95 (t, *J* = 7.6 Hz, 2H), 2.60 (p, *J* = 7.3 Hz, 2H); **^{13}C NMR** (101 MHz, $CDCl_3$): δ 154.7, 154.0, 149.2, 142.3, 136.4, 134.8, 131.4 (d, *J* = 9.9 Hz), 129.6, 128.4 (d, *J* = 12.2 Hz), 126.5, 125.4 (q, J = 3.7 Hz), 121.7, 121.6, 45.0, 26.3, 23.7; ***m/z*** (ESI+) 330 ($[M+H]^+$, 100%); **HRMS** (ESI+) found 330.1229 ($[M+H]^+$), $C_{18}H_{15}F_3N_3^+$ requires 330.1215.

3-(3-Methoxyphenyl)-2-(pyridin-2-yl)-6,7-dihydro-5*H*-pyrrolo[1,2-*a*]imidazole (OSA_000825)

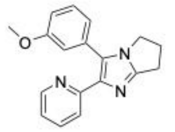
Prepared according to General Procedure D from: **S2** (80.0 mg, 0.30 mmol) and (3-methoxyphenyl)boronic acid (55.0 mg, 0.36 mmol); purified by methods 1 (65-90% EtOAc in hexane), then 3, to give *the title compound* as a brown solid (36.5 mg, 41%); **^{1}H NMR** (400 MHz, $CDCl_3$): δ 8.50 (ddd, *J* = 4.8, 1.8, 1.0 Hz, 1H), 7.60 (dt, *J* = 8.1, 1.2 Hz, 1H), 7.55 (td, *J* = 7.7, 1.8 Hz, 1H), 7.29 (t, *J* = 7.9 Hz, 1H), 7.05 (ddd, *J* = 7.3, 4.8, 1.4 Hz, 1H), 7.03 – 6.95 (m, 2H), 6.88 (ddd, *J* = 8.3, 2.6, 1.0 Hz, 1H), 3.98 (t, *J* = 7.1 Hz, 2H), 3.76 (s, 3H), 3.02 (t, *J* = 7.6 Hz, 2H), 2.62 (p, *J* = 7.3 Hz, 2H); **^{13}C NMR** (101 MHz, $CDCl_3$): δ 159.6, 153.9, 153.6, 149.3, 140.8, 136.1, 132.0, 129.6, 127.8, 121.7, 121.5, 121.4, 115.0, 113.8, 55.4, 44.9, 26.3, 23.7; ***m/z*** (ESI+) 292 ($[M+H]^+$, 100%); **HRMS** (ESI+) found 292.1447 ($[M+H]^+$), $C_{18}H_{18}N_3O^+$ requires 292.1444.

2-(Pyridin-2-yl)-3-(*m*-tolyl)-6,7-dihydro-5*H*-pyrrolo[1,2-*a*]imidazole (OSA_000976)

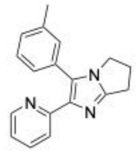

Prepared according to General Procedure D from: **S2** (50.0 mg, 0.19 mmol) and *m*-tolylboronic acid (33.5 mg, 0.25 mmol); purified by methods 1, then 2, to give *the title compound* as a light brown powder (21.9 mg, 42%); **^{1}H NMR** (500 MHz, $CDCl_3$): δ 8.49 (d, *J* = 4.6 Hz, 1H), 7.60 – 7.50 (m, 2H), 7.32 – 7.19 (m, 3H), 7.16 (d, *J* = 7.3 Hz, 1H), 7.04 (ddd, *J* = 6.8, 4.8, 1.6 Hz, 1H), 3.96 (t, *J* = 7.0 Hz, 2H), 3.00 (t, *J* = 7.6 Hz, 2H), 2.62 (p, *J* = 7.4 Hz, 2H), 2.35 (s, 3H); **^{13}C NMR** (101 MHz, $CDCl_3$): δ 154.4, 154.0, 149.4, 141.4, 138.2, 135.9, 131.1, 129.8, 128.7, 128.5, 128.1, 126.5, 121.5, 121.1, 44.7, 26.3, 23.7, 21.6; ***m/z*** (ESI+) 276 ([M+H]$^+$, 100%); **HRMS** (ESI+) found 276.1491 ([M+H]$^+$), $C_{18}H_{18}N_3^+$ requires 276.1495.

3-(3,4-Dichlorophenyl)-2-(pyridin-2-yl)-6,7-dihydro-5*H*-pyrrolo[1,2-*a*]imidazole (OSA_000876)

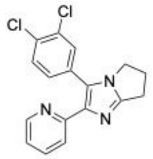

Prepared according to General Procedure D from: **S2** (50.0 mg, 0.19 mmol) and (3,4-dichlorophenyl)boronic acid (47.0 mg, 0.25 mmol); purified by methods 1, then 2, to give *the title compound* as a yellow powder (13.1 mg, 21%); **^{1}H NMR** (400 MHz, $CDCl_3$): δ 8.41 (d, *J* = 4.9 Hz, 1H), 7.77 (d, *J* = 8.0 Hz, 1H), 7.69 – 7.53 (m, 1H), 7.58 (s, 1H), 7.42 (d, *J* = 8.3 Hz, 1H), 7.30 (d, *J* = 8.3 Hz, 1H), 7.15 – 7.00 (m, 1H), 3.96 (t, *J* = 7.1 Hz, 2H), 2.99 (t, *J* = 7.6 Hz, 2H), 2.64 (p, *J* = 7.2 Hz, 2H); **^{13}C NMR** (101 MHz, $CDCl_3$): δ 154.6, 154.0, 149.1, 142.3, 136.4, 132.5, 131.8, 131.3, 131.0, 130.3, 128.9, 125.6, 121.7, 121.4, 44.8, 26.3, 23.7; ***m/z*** (ESI+) 330 ([M+H]$^+$, 100%); **HRMS** (ESI+) found 330.0555 ([M+H]$^+$), $C_{17}H_{14}Cl_2N_3^+$ requires 330.0559.

3-(Benzo[*d*][1,3]dioxol-5-yl)-2-(pyridin-2-yl)-6,7-dihydro-5*H*-pyrrolo[1,2-*a*]imidazole (OSA_000812)

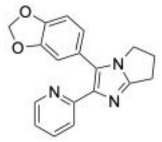

Prepared according to General Procedure D from: **S2** (50.0 mg, 0.19 mmol) and benzo[*d*][1,3]dioxol-5-ylboronic acid (40.8 mg, 0.25 mmol); purified by methods 1, then 3, to give *the title compound* as a light brown powder (18.2 mg, 31%); **^{1}H NMR** (500 MHz, $CDCl_3$): δ 8.49 (d, *J* = 4.7 Hz, 1H), 7.60 (d, *J* = 8.0 Hz, 1H), 7.55 (td, *J* = 7.7, 1.7 Hz, 1H), 7.04 (ddd, *J* = 7.0, 4.9, 1.0 Hz, 1H), 6.92 – 6.88 (m, 2H), 6.83 (d, *J* = 7.9 Hz, 1H), 6.00 (s, 2H), 3.93 (t, *J* = 7.0 Hz, 2H), 2.99 (t, *J* = 7.6 Hz, 2H), 2.62 (p, *J* = 7.3 Hz, 2H); **^{13}C NMR** (126 MHz, $CDCl_3$): δ 154.3, 153.8, 149.4, 147.7, 147.5, 141.2, 136.0, 127.6, 124.8, 123.1, 121.5, 121.1, 110.1, 108.6, 101.3, 44.6, 26.3, 23.7; ***m/z*** (ESI+) 306 ($[M+H]^+$, 100%); **HRMS** (ESI+) found 306.1244 ($[M+H]^+$), $C_{18}H_{16}N_3O_2^+$ requires 306.1237.

3-(2,3-Dihydrobenzofuran-5-yl)-2-(pyridin-2-yl)-6,7-dihydro-5*H*-pyrrolo[1,2-*a*]imidazole (OSA_000827)

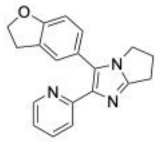

Prepared according to General Procedure D from: **S2** (80.0 mg, 0.30 mmol) and (2,3-dihydrobenzofuran-5-yl)boronic acid (60.0 mg, 0.36 mmol); purified by methods 1 (65-90% EtOAc in hexane), then 3, to give *the title compound* as a brown solid (41.1 mg, 45%); **^{1}H NMR** (400 MHz, $CDCl_3$): δ 8.49 (ddd, *J* = 4.9, 1.8, 1.0 Hz, 1H), 7.62 (dt, *J* = 8.0, 1.2 Hz, 1H), 7.56 (td, *J* = 7.7, 1.8 Hz, 1H), 7.26 (s, 1H), 7.21 – 7.12 (m, 1H), 7.05 (ddd, *J* = 7.3, 4.8, 1.3 Hz, 1H), 6.80 (d, *J* = 8.2 Hz, 1H), 4.61 (t, *J* = 8.7 Hz, 2H), 3.95 (t, 2H), 3.22 (t, *J* = 8.7 Hz, 2H), 3.07 (t, *J* = 7.6 Hz, 2H), 2.71 – 2.58 (m, 2H); **^{13}C NMR** (101 MHz, $CDCl_3$): δ 160.4, 153.2, 152.9, 149.3, 139.0, 136.3, 129.6, 128.4, 127.6, 126.2, 122.3, 121.5, 121.4, 109.5, 71.6, 44.9, 29.8, 26.2, 23.8; ***m/z*** (ESI+) 304 ($[M+H]^+$, 100%); **HRMS** (ESI+) found 304.1450 ($[M+H]^+$), $C_{19}H_{18}N_3O^+$ requires 304.1444.

3-(2,3-Dihydrobenzofuran-6-yl)-2-(pyridin-2-yl)-6,7-dihydro-5*H*-pyrrolo[1,2-*a*]imidazole (OSA_000828)

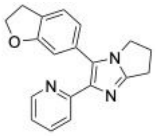

Prepared according to General Procedure D from: **S2** (80.0 mg, 0.30 mmol) and (2,3-dihydrobenzofuran-6-yl)boronic acid (60 mg, 0.36 mmol); purified by methods 1 (65-90% EtOAc in hexane), then 3, to give *the title compound* as a pale yellow solid (44.1 mg, 48%); **^{1}H NMR** (400 MHz, $CDCl_3$): δ 8.49 (ddd, *J* = 4.9, 1.8, 1.0 Hz, 1H), 7.59 – 7.47 (m, 2H), 7.17 (dt, *J* = 7.6, 1.2 Hz, 1H), 7.02 (ddd, *J* = 6.9, 4.9, 1.6 Hz, 1H), 6.88 (dd, *J* = 7.6, 1.5 Hz, 1H), 6.82 (d, *J* = 1.5 Hz, 1H), 4.58 (t, *J* = 8.7 Hz, 2H), 3.92 (t, *J* = 7.1 Hz, 2H), 3.22 (t, *J* = 8.7 Hz, 2H), 2.97 (t, 2H), 2.64 – 2.52 (m, 2H); **^{13}C NMR** (101 MHz, $CDCl_3$): δ 160.3, 154.1, 153.8, 149.4, 141.1, 135.9, 130.8, 127.9, 126.9, 124.9, 121.7, 121.6, 121.1, 110.1, 71.4, 44.7, 29.7, 26.2, 23.6; ***m/z*** (ESI+) 304 ([M+H]$^+$, 100%); **HRMS** (ESI+) found 304.1449 ([M+H]$^+$), $C_{19}H_{18}N_3O^+$ requires 304.1444.

2-Methyl-5-(4,4,5,5-tetramethyl-1,3,2-dioxaborolan-2-yl)benzo[*d*]thiazole (S6)

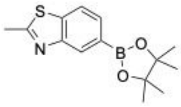

Prepared according to General Procedure F from: 5-bromo-2-methylbenzo[*d*]thiazole (100 mg, 0.47 mmol); purified by flash chromatography on silica (0-20% EtOAc in hexanes) to give *the title compound* as a white solid (88.1 mg, 72%); **^{1}H NMR** (400 MHz, $CDCl_3$): δ 8.39 (s, 1H), 7.83 (d, *J* = 8.1 Hz, 1H), 7.76 (d, *J* = 7.8 Hz, 1H), 2.85 (s, 3H) 1.38 (s, 12H); ***m/z*** (ESI+) 276 ([M+H]$^+$, 100%).

2-Methyl-5-(2-(pyridin-2-yl)-6,7-dihydro-5*H*-pyrrolo[1,2-*a*]imidazol-3-yl)benzo[*d*]thiazole (OSA_000836)

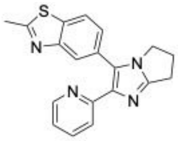

Prepared according to General Procedure D from: **S2** (65.0 mg, 0.25 mmol) and **S6** (88.0 mg, 0.32 mmol); purified by methods 1, then 3, to give *the title compound* as a tan powder (23.5 mg, 29%); **^{1}H NMR** (500 MHz, $CDCl_3$): δ 8.44 (ddd, *J* = 4.9, 1.7, 0.9 Hz, 1H), 8.01 (d, *J* = 1.6 Hz, 1H), 7.79 (d, *J* = 8.2 Hz, 1H), 7.64 (d, *J* = 8.0 Hz, 1H), 7.55 (td, *J* = 7.7, 1.9 Hz, 1H), 7.43 (dd, *J* = 8.3, 1.7 Hz, 1H), 7.04 (ddd, *J* = 7.5, 4.8, 1.2 Hz,

1H), 4.00 (t, J = 7.1 Hz, 2H), 3.02 (t, J = 7.5 Hz, 2H), 2.85 (s, 3H), 2.64 (p, J = 7.3 Hz, 2H); **^{13}C NMR** (126 MHz, $CDCl_3$): δ 167.8, 154.3, 154.2, 153.7, 149.3, 141.6, 136.1, 135.2, 129.2, 127.4, 126.6, 122.5, 121.6, 121.38, 121.36, 44.8, 26.3, 23.7, 20.4; ***m/z*** (ESI+) 333 ($[M+H]^+$, 100%); **HRMS** (ESI+) found 333.1167 ($[M+H]^+$), $C_{19}H_{17}N_4S^+$ requires 333.1168.

7-(4,4,5,5-Tetramethyl-1,3,2-dioxaborolan-2-yl)quinoline (S7)

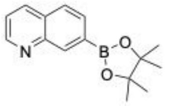

Prepared according to General Procedure F from: 4-bromoquinoline (100 mg, 0.48 mmol); purified by flash chromatography on silica (0-20% EtOAc in hexanes) to give *the title compound* as a white powder (106 mg, 86%); **^{1}H NMR** (500 MHz, $CDCl_3$): δ 8.94 (dd, J = 4.2, 1.6 Hz, 1H), 8.61 (s, 1H), 8.14 (d, J = 8.2 Hz, 1H), 7.90 (d, J = 8.1 Hz, 1H), 7.80 (d, J = 8.1 Hz, 1H), 7.41 (dd, J = 8.3, 4.2 Hz, 1H), 1.39 (s, 12H); **^{13}C NMR** (126 MHz, $CDCl_3$): δ 150.6, 147.8, 137.5, 136.0, 131.3, 130.1, 127.0, 121.9, 84.3, 25.0. Spectroscopic data matched those in the literature.[5]

7-(2-(Pyridin-2-yl)-6,7-dihydro-5*H*-pyrrolo[1,2-*a*]imidazol-3-yl)quinoline (OSA_000833)

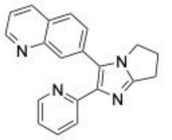

Prepared according to General Procedure D from: **S2** (50.0 mg, 0.19 mmol) and **S7** (62.8 mg, 0.25 mmol); purified by method 1 to give *the title compound* as a light brown powder (36.0 mg, 61%); **^{1}H NMR** (500 MHz, $CDCl_3$): δ 8.92 (dd, J = 4.2, 1.5 Hz, 1H), 8.56 – 8.33 (m, 1H), 8.24 – 8.08 (m, 2H), 7.76 (t, J = 9.1 Hz, 2H), 7.67 – 7.56 (m, 2H), 7.41 (dd, J= 8.2, 4.2 Hz, 1H), 7.08 (ddd, J = 7.4, 4.9, 1.0 Hz, 1H), 4.11 (t, J = 7.0 Hz, 2H), 3.15 – 2.87 (m, 2H), 2.67 (p, J = 7.4 Hz, 2H); **^{13}C NMR** (126 MHz, $CDCl_3$): δ 154.8, 154.2, 150.9, 149.3, 148.4, 142.3, 136.2, 135.9, 132.6, 129.0, 128.3, 127.8, 127.6, 127.2, 121.7, 121.6, 121.4, 45.1, 26.4, 23.8; ***m/z*** (ESI+) 313 ($[M+H]^+$, 100%); **HRMS** (ESI+) found 313.1458 ($[M+H]^+$), $C_{20}H_{17}N_4^+$ requires 313.1448.

5-(4,4,5,5-Tetramethyl-1,3,2-dioxaborolan-2-yl)benzo[*d*]thiazole (S8)

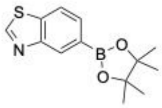

Prepared according to General Procedure F from: 5-bromobenzo[*d*]thiazole (100 mg, 0.47 mmol); purified by flash chromatography on silica (0-20% EtOAc in hexanes) to give *the title compound* as a yellow solid (91.5 mg, 75%); **^{1}H NMR** (400 MHz, $CDCl_3$): δ 9.01 (s, 1H), 8.60 (s, 1H), 7.98 (d, *J* = 8.1 Hz, 1H), 7.87 (d, *J* = 8.1 Hz, 1 H), 1.39 (s, 12H); ***m/z*** (ESI+) 262 ($[M+H]^+$, 100%).

5-(2-(Pyridin-2-yl)-6,7-dihydro-5*H*-pyrrolo[1,2-*a*]imidazol-3-yl)benzo[*d*]thiazole (OSA_000835)

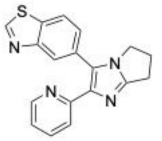

Prepared according to General Procedure D from: **S2** (70.0 mg, 0.27 mmol) and **S8** (92.0 mg, 0.35 mmol); purified by methods 1, then 3, to give *the title compound* as an orange powder (10.2 mg, 12%); **^{1}H NMR** (500 MHz, $CDCl_3$): δ 9.03 (s, 1H), 8.42 (ddd, *J* = 4.9, 1.9, 0.9 Hz, 1H), 8.21 (d, *J* = 1.7 Hz, 1H), 7.94 (d, *J* = 8.3 Hz, 1H), 7.70 (d, *J* = 8.0 Hz, 1H), 7.63 – 7.40 (m, 2H), 7.05 (ddd, *J* = 7.5, 4.9, 1.2 Hz, 1H), 4.03 (t, *J* = 7.1 Hz, 2H), 3.04 (t, *J* = 7.6 Hz, 2H), 2.66 (p, *J* = 7.3 Hz, 2H); **^{13}C NMR** (126 MHz, $CDCl_3$): δ 154.7, 154.4, 154.2, 153.6, 149.3, 141.8, 136.2, 133.2, 129.6, 127.6, 127.2, 123.7, 121.8, 121.5, 121.4, 44.8, 26.3, 23.7; ***m/z*** (ESI+) 319 ($[M+H]^+$, 100%); **HRMS** (ESI–) found 317.0854 ($[M–H]^-$), $C_{18}H_{13}N_4S^-$ requires 317.0866.

3-Phenyl-2-(pyridin-2-yl)-6,7-dihydro-5*H*-pyrrolo[1,2-*a*]imidazole (OSA_000870)

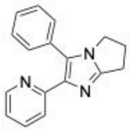

Prepared according to General Procedure D from: **S2** (75.0 mg, 0.28 mmol) and phenylboronic acid (42.0 mg, 0.34 mmol); purified by methods 1, then 2, to give *the title compound* as a tan powder (17.9 mg, 24%); **^{1}H NMR** (500 MHz, $CDCl_3$): δ 8.48 (d, *J* = 4.9 Hz, 1H), 7.83 – 7.48 (m, 2H), 7.43 (d, *J* = 6.9 Hz, 2H), 7.38 (t, *J* = 7.3 Hz, 2H), 7.36 – 7.30 (m, 1H), 7.04 (ddd, *J* = 6.8, 4.8, 1.5 Hz, 1H), 3.96 (t, *J* = 7.1 Hz, 2H), 3.00 (t, *J* = 7.5 Hz,

2H), 2.62 (p, *J* = 7.3 Hz, 2H); **^{13}C NMR** (126 MHz, $CDCl_3$): δ 154.3, 154.1, 149.4, 141.5, 135.9, 131.2, 129.3, 128.6, 127.9, 121.5, 121.2, 44.7, 26.3, 23.7 ***m/z*** (ESI+) 262 ([M+H]$^+$, 100%); **HRMS** (ESI+) found 262.1354 ([M+H]$^+$), $C_{17}H_{16}N_3^+$ requires 262.1339.

4,4,5,5-Tetramethyl-2-(4-(methylsulfinyl)phenyl)-1,3,2-dioxaborolane (S9)

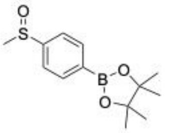

Prepared according to General Procedure F from: 4-bromophenylmethylsulfoxide (200 mg, 0.91 mmol); purified by flash chromatography on silica (12-100% EtOAc in hexanes) to give *the title compound* as a grey powder (223 mg, 92%); **^{1}H NMR** (400 MHz, $CDCl_3$): δ 7.96 (d, *J* = 8.2 Hz, 2H), 7.64 (d, *J* = 8.2 Hz, 2H), 2.72 (s, 3H), 1.36 (s, 12H); ***m/z*** (ESI+) 267 ([M+H]$^+$, 100%).

3-(4-(Methylsulfinyl)phenyl)-2-(pyridin-2-yl)-6,7-dihydro-5*H*-pyrrolo[1,2-*a*]imidazole (OSA_000838)

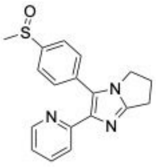

Prepared according to General Procedure D from: **S2** (50.0 mg, 0.19 mmol) and **S9** (45.3 mg, 0.25 mmol); purified by methods 1, then 3, to give *the title compound* as a light brown powder (25.3 mg, 41%); **^{1}H NMR** (500 MHz, $CDCl_3$): δ 8.41 (d, *J* = 4.7 Hz, 1H), 7.76 (d, *J* = 8.0 Hz, 1H), 7.71 – 7.56 (m, 5H), 7.22 – 6.94 (m, 1H), 4.00 (dt, *J* = 10.5, 5.3 Hz, 2H), 3.02 (t, *J* = 7.6 Hz, 2H), 2.79 (s, 3H), 2.66 (p, *J* = 7.2 Hz, 2H); **^{13}C NMR** (126 MHz, $CDCl_3$): δ 154.8, 154.1, 149.1, 144.7, 142.4, 136.4, 134.2, 130.2, 126.6, 123.7, 121.7, 121.6, 45.0, 43.9, 26.3, 23.7; ***m/z*** (ESI+) 324 ([M+H]$^+$, 100%); **HRMS** (ESI+) found 324.1160 ([M+H]$^+$), $C_{18}H_{18}N_3OS^+$ requires 324.1165.

1-(4-(2-(Pyridin-2-yl)-6,7-dihydro-5*H*-pyrrolo[1,2-*a*]imidazol-3-yl)phenyl)ethan-1-one (OSA_000973)

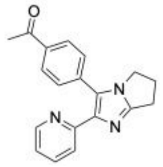

Prepared according to General Procedure D from: **S2** (50.0 mg, 0.19 mmol) and 4-acetylbenzeneboronic acid (40.4 mg, 0.25 mmol); purified by methods 1, then 2, to give *the title compound* as an off-white powder (22.7 mg, 40%); **^{1}H NMR** (400 MHz, $CDCl_3$): δ 8.44 (d, *J* = 4.7 Hz, 1H), 7.97 (d, *J* = 8.3 Hz, 2H), 7.75 (d, *J* = 8.1 Hz, 1H), 7.63 (t, *J* = 7.6 Hz, 1H), 7.56 (d, *J* = 8.3 Hz, 2H), 7.17 – 7.03 (m, 1H), 4.02 (t, *J* = 7.1 Hz, 2H), 3.05 (t, *J* = 7.5 Hz, 2H), 2.68 (p, *J* = 7.3 Hz, 2H), 2.63 (s, 3H); **^{13}C NMR** (101 MHz, $CDCl_3$): δ 197.7, 154.8, 154.0, 149.2, 142.5, 136.3, 136.1, 135.9, 129.3, 128.5, 126.9, 121.71, 121.69, 45.1, 26.7, 26.3, 23.7; ***m/z*** (ESI+) 304 ($[M+H]^+$, 100%); **HRMS** (ESI+) found 304.1442 ($[M+H]^+$), $C_{19}H_{18}N_3O^+$ requires 304.1444.

3-(4-(Methylsulfonyl)phenyl)-2-(pyridin-2-yl)-6,7-dihydro-5*H*-pyrrolo[1,2-*a*]imidazole (OSA_000837)

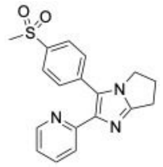

Prepared according to General Procedure D from: **S2** (50.0 mg, 0.19 mmol) and (4-(methylsulfonyl)phenyl)boronic acid (49.2 mg, 0.25 mmol); purified by methods 1, then 2, to give *the title compound* as a white powder (26.1 mg, 41%); **^{1}H NMR** (500 MHz, $CDCl_3$): δ 8.40 (d, *J* = 4.1 Hz, 1H), 7.93 (d, *J* = 8.4 Hz, 2H), 7.82 (d, *J* = 8.0 Hz, 1H), 7.69 – 7.64 (m, 3H), 7.17 – 7.01 (m, 2H), 4.02 (t, *J* = 7.1 Hz, 2H), 3.11 (s, 3H), 3.03 (t, *J* = 7.6 Hz, 2H), 2.68 (p, *J* = 7.4 Hz, 2H); **^{13}C NMR** (126 MHz, $CDCl_3$): δ 155.3, 154.0, 149.0, 143.0, 139.1, 136.9, 136.5, 130.0, 127.4, 126.0, 122.0, 121.7, 45.2, 44.7, 31.1, 26.3, 23.7; ***m/z*** (ESI+) 340 ($[M+H]^+$, 100%); **HRMS** (ESI+) found 340.1110 ($[M+H]^+$), $C_{18}H_{18}N_3O_2S^+$ requires 340.1114.

***N*,*N*-Dimethyl-4-(2-(pyridin-2-yl)-6,7-dihydro-5*H*-pyrrolo[1,2-*a*]imidazol-3-yl)benzamide (OSA_000974)**

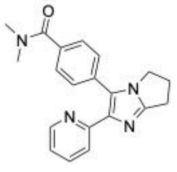

Prepared according to General Procedure D from: **S2** (50.0 mg, 0.19 mmol) and 4-(dimethylcarbamoyl)benzeneboronic acid (47.5 mg, 0.25 mmol); purified by methods 1, then 2, to give *the title compound* as an off-white powder (26.4 mg, 42%); **^{1}H NMR** (400 MHz, $CDCl_3$): δ 8.45 (d, J = 4.5 Hz, 1H), 7.70 (d, J = 8.3 Hz, 1H), 7.60 (t, J = 7.6 Hz, 1H), 7.49 (d, J = 8.3 Hz, 2H), 7.45 (d, J = 8.5 Hz, 2H), 7.12 – 7.05 (m, 1H), 3.98 (t, J = 6.9 Hz, 2H), 3.28 – 2.90 (m, 8H), 2.65 (p, J = 7.2 Hz, 2H); **^{13}C NMR** (101 MHz, $CDCl_3$): δ 171.4, 154.4, 154.0, 149.2, 141.7, 136.2, 135.6, 132.4, 129.2, 127.4, 127.2, 121.6, 121.5, 44.9, 39.8, 26.3, 23.7; ***m/z*** (ESI+) 333 ($[M+H]^+$, 100%); **HRMS** (ESI+) found 333.1704 ($[M+H]^+$), $C_{20}H_{21}N_4O^+$ requires 333.1710.

3-(2-(Pyridin-2-yl)-6,7-dihydro-5*H*-pyrrolo[1,2-*a*]imidazol-3-yl)benzonitrile (OSA_000864)

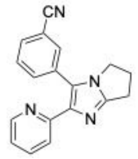

Prepared according to General Procedure D from: **S2** (50.0 mg, 0.19 mmol) and (3-cyanophenyl)boronic acid (36.2 mg, 0.25 mmol); purified by methods 1, then 3, to give *the title compound* as a white powder (16.1 mg, 30%); **^{1}H NMR** (500 MHz, $CDCl_3$): δ 8.80 – 8.26 (m, 1H), 7.86 – 7.77 (m, 2H), 7.71 (d, J = 7.9 Hz, 1H), 7.65 (td, J = 7.8, 1.7 Hz, 1H), 7.61 (d, J = 7.8 Hz, 1H), 7.48 (t, J = 7.8 Hz, 1H), 7.12 – 7.05 (m, 1H), 3.99 (t, J = 7.0 Hz, 2H), 3.02 (t, J = 7.6 Hz, 2H), 2.67 (p, J = 7.3 Hz, 2H); **^{13}C NMR** (126 MHz, $CDCl_3$): δ 154.8, 153.9, 149.0, 142.5, 136.5, 133.8, 132.9, 132.7, 131.1, 129.1, 125.6, 121.9, 121.3, 118.9, 112.5, 44.9, 31.1, 26.3, 23.7; ***m/z*** (ESI+) 287 ($[M+H]^+$, 100%); **HRMS** (ESI+) found 287.1287 ($[M+H]^+$), $C_{18}H_{15}N_4^+$ requires 287.1291.

3-(3-Fluorophenyl)-2-(pyridin-2-yl)-6,7-dihydro-5*H*-pyrrolo[1,2-*a*]imidazole (OSA_000872)

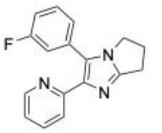

Prepared according to General Procedure D from: **S2** (75.0 mg, 0.28 mmol) and (3-fluorophenyl)boronic acid (50.0 mg, 0.36 mmol); purified by methods 1, 3, then 2 (5-100% MeCN in H_2O), to give *the title compound* as a tan powder (12.9 mg, 16%); **^{1}H NMR** (500 MHz, $CDCl_3$): δ 8.46 (ddd, *J* = 4.9, 1.9, 1.0 Hz, 1H), 7.68 (dt, *J* = 8.0, 1.2 Hz, 1H), 7.60 (td, *J* = 7.7, 1.8 Hz, 1H), 7.34 (td, *J* = 8.0, 6.0 Hz, 1H), 7.20 (dt, *J* = 7.7, 1.2 Hz, 1H), 7.17 (ddd, *J* = 9.9, 2.6, 1.5 Hz, 1H), 7.08 (ddd, *J* = 7.4, 4.8, 1.2 Hz, 1H), 7.03 (tdd, *J* = 8.4, 2.6, 1.0 Hz, 1H), 3.98 (t, *J* = 7.1 Hz, 2H), 3.03 (t, *J* = 7.6 Hz, 2H), 2.64 (p, *J* = 7.3 Hz, 2H); **^{13}C NMR** (126 MHz, $CDCl_3$): δ 163.7, 161.7, 154.2, 153.5, 149.2, 141.3, 136.3, 132.9 (d, *J* = 8.2 Hz), 130.0 (d, *J* = 8.5 Hz), 126.7, 125.0 (d, *J* = 2.8 Hz), 121.6, 116.3 (d, *J* = 22.1 Hz), 114.9 (d, *J* = 21.1 Hz), 45.0, 26.3, 23.7; ***m/z*** (ESI+) 280 ($[M+H]^+$, 100%); **HRMS** (ESI+) found 280.1251 ($[M+H]^+$), $C_{17}H_{15}FN_3^+$ requires 280.1245.

2-(Pyridin-2-yl)-3-(*o*-tolyl)-6,7-dihydro-5*H*-pyrrolo[1,2-*a*]imidazole (OSA_000977)

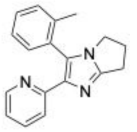

Prepared according to General Procedure D from: **S2** (100 mg, 0.38 mmol) and *o*-tolylboronic acid (56.6 mg, 0.42 mmol); purified by methods 1, then 2, to give *the title compound* as a pale yellow powder (20.2 mg, 19%); **^{1}H NMR** (500 MHz, $CDCl_3$): δ 8.49 (d, *J* = 4.7 Hz, 1H), 7.48 (t, *J* = 6.5 Hz, 1H), 7.42 – 7.33 (m, 2H), 7.33 – 7.27 (m, 3H), 7.00 (t, *J* = 6.1 Hz, 1H), 3.94 – 3.79 (m, 1H), 3.74 – 3.64 (m, 1H), 3.08 (s, 2H), 2.63 (t, *J* = 6.9 Hz, 2H), 2.10 (s, 3H); **^{13}C NMR** (101 MHz, $CDCl_3$): δ 153.9, 153.6, 149.6, 141.6, 138.2, 135.9, 131.1, 130.5, 130.4, 128.8, 126.9, 126.1, 120.8, 120.1, 44.0, 26.2, 23.7, 20.0; ***m/z*** (ESI+) 276 ($[M+H]^+$, 100%); **HRMS** (ESI+) found 276.1494 ($[M+H]^+$), $C_{18}H_{18}N_3^+$ requires 276.1495.

3-(3,4-Dimethoxyphenyl)-2-(pyridin-2-yl)-6,7-dihydro-5*H*-pyrrolo[1,2-*a*]imidazole (OSA_000820)

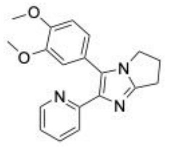

Prepared according to General Procedure D from: **S2** (75.0 mg, 0.28 mmol) and (3,4-dimethoxyphenyl)boronic acid (67.0 mg, 0.37 mmol); purified by methods 1 (with 5% NH_4OH in MeOH), then 2, to give *the title compound* as a tan powder (35.6 mg, 39%); **^{1}H NMR** (500 MHz, $CDCl_3$): δ 8.62 – 8.30 (m, 1H), 7.60 (d, *J* = 8.0 Hz, 1H), 7.54 (td, *J* = 7.7, 1.9 Hz, 1H), 7.10 – 7.00 (m, 2H), 6.97 (dd, *J* = 8.2, 2.0 Hz, 1H), 6.89 (d, *J* = 8.3 Hz, 1H), 3.95 (t, *J* = 7.1 Hz, 2H), 3.91 (s, 3H), 3.77 (s, 3H), 2.98 (t, *J* = 7.6 Hz, 2H), 2.61 (p, *J* = 7.3 Hz, 2H); **^{13}C NMR** (126 MHz, $CDCl_3$): δ 154.4, 153.7, 149.2, 148.8, 148.7, 141.0, 135.9, 127.9, 123.7, 121.5, 121.3, 121.1, 113.2, 111.1, 55.99, 55.96, 44.6, 26.3, 23.6; ***m/z*** (ESI+) 322 ($[M+H]^+$, 100%); **HRMS** (ESI+) found 322.1549 ($[M+H]^+$), $C_{19}H_{20}N_3O_2^+$ requires 322.1550.

2-Bromo-1-(pyrazin-2-yl)ethan-1-one hydrobromide (S10)

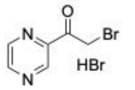

Acetylpyrazine (1.00 g, 8.19 mmol, 1.00 equiv.) was dissolved in glacial AcOH (7 mL, 1.17 M). HBr (33% in glacial AcOH, 1.84 mL, 10.2 mmol, 1.24 equiv.) and pyridinium tribromide (2.66 g, 8.32 mmol, 1.02 equiv.) were added and the reaction stirred at rt overnight. Et_2O (20 mL) was added and the mixture sonicated to give a black solid, which was collected by filtration and washed with MeCN, followed by Et_2O and dried *in vacuo* to give *the title compound* as a black powder (1.14 g, 49%); **^{1}H NMR** (500 MHz, DMSO-d_6): δ 9.18 (d, *J* = 1.3 Hz, 1H), 8.95 (d, *J* = 2.4 Hz, 1H), 8.83 (dd, *J* = 2.3, 1.5 Hz, 1H), 5.01 (s, 2H). Spectroscopic data matched those in the literature.[6]

2-(Pyrazin-2-yl)-6,7-dihydro-5*H*-pyrrolo[1,2-*a*]imidazole (S11)

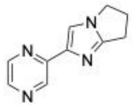

Prepared according to General Procedure A from: **S10** (750 mg, 2.66 mmol) and 3,4-dihydro-2*H*-pyrrol-5-amine hydrochloride (481 mg, 3.99 mmol); purified by flash chromatography on silica (1-10% MeOH in CH_2Cl_2) to give *the title compound* as a reddish-brown powder (72.0 mg, 15%); **^{1}H NMR** (500 MHz, $CDCl_3$): δ 9.16 (s, 1H), 8.44 (s,

1H), 8.36 (d, *J* = 2.4 Hz, 1H), 7.60 (s, 1H), 4.06 (t, *J* = 7.1 Hz, 2H), 3.03 – 2.89 (m, 2H), 2.66 (p, *J* = 7.4 Hz, 2H); **^{13}C NMR** (126 MHz, $CDCl_3$): δ 155.9, 149.3, 144.1, 143.8, 142.1, 141.4, 115.0, 45.1, 26.3, 23.2; ***m/z*** (ESI+): 187 ($[M+H]^+$, 100%).

3-Bromo-2-(pyrazin-2-yl)-6,7-dihydro-5*H*-pyrrolo[1,2-*a*]imidazole (S12)

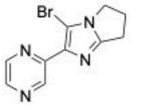

Prepared according to General Procedure C from: **S11** (50.0 mg, 0.27 mmol) to give *the title compound* as an orange powder (28.3 mg, 40%); **^{1}H NMR** (400 MHz, $CDCl_3$): δ 9.32 (s, 1H), 8.61 (t, *J* = 2.0 Hz, 1H), 8.48 (d, *J* = 2.5 Hz, 1H), 4.11 (t, *J* = 7.2 Hz, 2H), 3.21 (t, *J* = 7.7 Hz, 2H), 2.74 (p, *J* = 7.5 Hz, 2H); ***m/z*** (ESI+) 265 ($[M+H]^+$, 100%).

3-(Benzofuran-5-yl)-2-(pyrazin-2-yl)-6,7-dihydro-5*H*-pyrrolo[1,2-*a*]imidazole (OSA_000873)

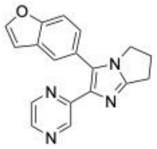

Prepared according to General Procedure D from: **S12** (20.0 mg, 0.08 mmol) and **S4** (23.9 mg, 0.10 mmol); purified by methods 1, then 2, to give *the title compound* as a pale brown powder (6.3 mg, 28%); **^{1}H NMR** (500 MHz, $CDCl_3$): δ 8.81 (s, 1H), 8.44 (s, 1H), 8.33 (s, 1H), 7.70 (d, *J* = 2.3 Hz, 1H), 7.67 (d, *J* = 1.8 Hz, 1H), 7.58 (d, *J* = 8.6 Hz, 1H), 7.34 (dd, *J* = 8.5, 1.8 Hz, 1H), 6.80 (dd, *J* = 2.3, 1.0 Hz, 1H), 4.00 (t, *J* = 7.2 Hz, 2H), 3.23 – 3.14 (m, 2H), 2.71 (p, *J* = 7.4 Hz, 2H); **^{13}C NMR** (101 MHz, $CDCl_3$): δ 154.9, 154.6, 150.2, 146.0, 143.8, 143.1, 141.5, 138.5, 129.8, 128.0, 126.0, 125.2, 122.2, 111.9, 106.9, 44.5, 26.3, 23.7; ***m/z*** (ESI+) 303 ($[M+H]^+$, 100%); **HRMS** (ESI+) found 303.1238 ($[M+H]^+$), $C_{18}H_{15}N_4O^+$ requires 303.1240.

2-Bromo-1-(pyrimidin-5-yl)ethan-1-one hydrobromide (S13)

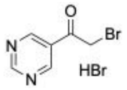

1-(5-Pyrimidinyl)ethanone (750 mg, 6.14 mmol, 1.00 equiv.) was dissolved in glacial AcOH (5.25 mL, 1.17 M). HBr (33% in glacial AcOH, 1.38 mL, 10.2 mmol, 1.24 equiv.) and pyridinium tribromide (2.00 g, 6.26 mmol, 1.02 equiv.) were added and the reaction stirred at rt overnight. Et_2O (15 mL) was added and the mixture sonicated to give a brown solid, which was collected by filtration and washed with MeCN, followed by Et_2O and dried *in vacuo* to give *the title compound* as a brown powder (712 mg, 41%); **^{1}H NMR** (500 MHz, DMSO-d_6): δ 9.41 (s, 1H), 9.31 (s, 2H), 5.05 (s, 2H).

2-(Pyrimidin-5-yl)-6,7-dihydro-5*H*-pyrrolo[1,2-*a*]imidazole (S14)

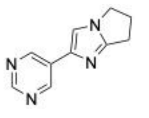

Prepared according to General Procedure A from: **S13** (500 mg, 1.77 mmol) and 3,4-dihydro-2*H*-pyrrol-5-amine hydrochloride (321 mg, 2.66 mmol); purified by flash chromatography on silica (1-10% MeOH in CH_2Cl_2) to give *the title compound* as an orange powder (27.5 mg, 8%); **^{1}H NMR** (400 MHz, $CDCl_3$): δ 9.08 (s, 2H), 9.07 (s, 1H), 7.31 (s, 1H), 4.08 (t, J = 7.1 Hz, 2H), 3.00 (t, J = 7.5 Hz, 2H), 2.68 (p, J = 7.4 Hz, 2H); ***m/z*** (ESI+) 187 ($[M+H]^+$, 100%).

3-Bromo-2-(pyrimidin-5-yl)-6,7-dihydro-5*H*-pyrrolo[1,2-*a*]imidazole (S15)

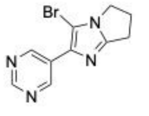

Prepared according to General Procedure C from: **S14** (20.0 mg, 0.10 mmol) to give *the title compound* as a brown powder (11.2 mg, 39%); **^{1}H NMR** (500 MHz, $CDCl_3$): δ 9.32 (s, 2H), 9.14 (s, 1H), 4.06 (t, J = 7.2 Hz, 2H), 3.40 – 2.90 (m, 2H), 2.71 (p, J = 8.7, 8.0 Hz, 2H); ***m/z*** (ESI+): 265 ($[M+H]^+$, 100%).

3-(4-Chlorophenyl)-2-(pyridin-2-yl)-6,7-dihydro-5*H*-pyrrolo[1,2-*a*]imidazole (OSA_000874)

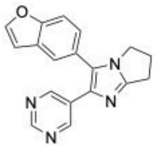

Prepared according to General Procedure D from: **S15** (9.50 mg, 0.04 mmol) and **S4** (11.4 mg, 0.05 mmol); purified by methods 1, then 2, to give *the title compound* as a pale yellow powder (5.3 mg, 49%); **^{1}H NMR** (400 MHz, $CDCl_3$): δ 9.00 (s, 1H), 8.88 (d, *J* = 2.8 Hz, 2H), 7.71 (d, *J* = 2.2 Hz, 1H), 7.60 (dd, *J* = 8.0, 2.7 Hz, 2H), 7.26 (dd, *J* = 8.7, 1.6 Hz, 1H), 6.79 (d, *J* = 2.2 Hz, 1H), 3.99 (t, *J* = 7.2 Hz, 2H), 3.14 (t, *J* = 7.6 Hz, 2H), 2.71 (p, *J* = 7.3 Hz, 2H); **^{13}C NMR** (101 MHz, $CDCl_3$): δ 156.5, 155.2, 154.5, 154.4, 146.5, 129.6, 128.7, 128.4, 127.9, 125.4, 124.1, 122.1, 112.8, 106.8, 44.7, 26.3, 23.9; ***m/z*** (ESI+) 303 ([M+H]$^+$, 100%); **HRMS** (ESI+) found 303.1236 ([M+H]$^+$), $C_{18}H_{15}N_4O^+$ requires 303.1240.

2-(Pyridin-3-yl)-6,7-dihydro-5*H*-pyrrolo[1,2-*a*]imidazole (S16)

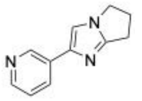

Prepared according to General Procedure A from: 2-bromo-1-(pyridin-3-yl)ethan-1-one hydrobromide (1.00 g, 3.56 mmol) and 3,4-dihydro-2*H*-pyrrol-5-amine hydrochloride (644 mg, 5.34 mmol); purified by flash chromatograph on silica (1-10% MeOH in CH_2Cl_2) to give *the title compound* as an orange powder (230 mg, 35%); **^{1}H NMR** (500 MHz, $CDCl_3$): δ 8.94 (d, *J* = 2.5 Hz, 1H), 8.44 (dd, *J* = 4.9, 1.7 Hz, 1H), 8.06 (dt, *J* = 7.9, 2.0 Hz, 1H), 7.30 – 7.27 (m, 1H), 7.25 (s, 1H), 4.04 (t, *J* = 7.2 Hz, 2H), 2.94 (t, *J* = 7.6 Hz, 2H), 2.64 (p, *J* = 7.3 Hz, 2H); ***m/z*** (ESI+) 186 ([M+H]$^+$, 100%).

3-Bromo-2-(pyridin-3-yl)-6,7-dihydro-5*H*-pyrrolo[1,2-*a*]imidazole (S17)

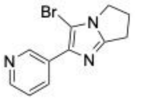

Prepared according to General Procedure C from: **S16** (175 mg, 0.94 mmol) to give *the title compound* as a brown powder (165 mg, 66%); **^{1}H NMR** (400 MHz, $CDCl_3$): δ 9.21 (s, 1H), 8.51 (d, *J* = 4.9 Hz, 1H), 8.22 (d, *J* = 8.0 Hz, 1H), 7.31 (dd, *J* = 7.9, 4.9 Hz, 1H), 4.00 (t, *J* = 7.1 Hz, 2H), 3.01 (t, *J* = 7.5 Hz, 2H), 2.65 (p, *J* = 7.7 Hz, 2H); ***m/z*** (ESI+) 264 ([M+H]$^+$, 100%).

3-(Benzofuran-5-yl)-2-(pyridin-3-yl)-6,7-dihydro-5*H*-pyrrolo[1,2-*a*]imidazole (OSA_000861)

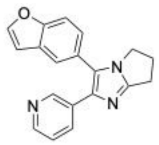

Prepared according to General Procedure D from: **S17** (50.0 mg, 0.19 mmol) and **S4** (60.1 mg, 0.25 mmol); purified by methods 1, then 2, to give *the title compound* as a white powder (34.9 mg, 61%); **^{1}H NMR** (500 MHz, $CDCl_3$): δ 8.78 – 8.69 (m, 1H), 8.37 (dd, *J* = 4.8, 1.6 Hz, 1H), 7.86 (dt, *J* = 8.0, 1.9 Hz, 1H), 7.68 (d, *J* = 2.1 Hz, 1H), 7.58 (d, *J* = 1.2 Hz, 1H), 7.54 (d, *J* = 8.5 Hz, 1H), 7.29 – 7.23 (m, 1H), 7.15 (dd, *J* = 8.0, 4.8 Hz, 1H), 6.79 – 6.74 (m, 1H), 4.08 – 3.77 (m, 2H), 3.06 – 2.97 (m, 2H), 2.65 (p, *J* = 7.3 Hz, 2H); **^{13}C NMR** (126 MHz, $CDCl_3$): δ 154.8, 154.1, 148.3, 147.3, 146.1, 138.5, 133.9, 131.4, 128.4, 126.8, 125.6, 125.5, 123.3, 122.0, 112.4, 106.8, 44.4, 26.3, 23.8; ***m/z*** (ESI+) 302 ([M+H]$^+$, 100%); **HRMS** (ESI+) found 302.1286 ([M+H]$^+$), $C_{19}H_{16}N_3O^+$ requires 302.1288.

2-(Pyridin-4-yl)-6,7-dihydro-5*H*-pyrrolo[1,2-*a*]imidazole (S18)

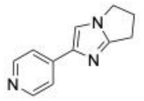

Prepared according to General Procedure A from: 2-bromo-1-(pyridin-4-yl)ethan-1-one hydrobromide (1.00 g, 3.56 mmol) and 3,4-dihydro-2*H*-pyrrol-5-amine hydrochloride (644 mg, 5.34 mmol); purified by flash chromatograph on silica (1-10% MeOH in CH_2Cl_2) to give *the title compound* as a brown powder (246 mg, 37%); **^{1}H NMR** (500 MHz, $CDCl_3$): δ 8.54 (d, *J* = 6.3 Hz, 2H), 7.60 (d, *J* = 6.1 Hz, 2H), 7.35 (s, 1H), 4.04 (t, *J* = 7.1 Hz, 2H), 2.94 (t, *J* = 7.6 Hz, 2H), 2.64 (p, *J* = 7.4 Hz, 2H); ***m/z*** (ESI+) 186 ([M+H]$^+$, 100%).

3-Bromo-2-(pyridin-4-yl)-6,7-dihydro-5*H*-pyrrolo[1,2-*a*]imidazole (S19)

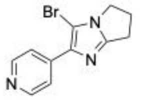

Prepared according to General Procedure C from: **S18** (175 mg, 0.94 mmol) to give *the title compound* as an orange powder (166 mg, 66%); **^{1}H NMR** (400 MHz,

$CDCl_3$): δ 8.60 (d, *J* = 4.9 Hz, 2H), 7.88 (d, *J* = 4.8 Hz, 2H), 4.00 (t, *J* = 7.2 Hz, 2H), 3.01 (t, *J* = 7.6 Hz, 2H), 2.66 (p, *J* = 7.3 Hz, 2H); ***m/z*** (ESI+) 264 ($[M+H]^+$, 100%).

3-(Benzofuran-5-yl)-2-(pyridin-4-yl)-6,7-dihydro-5*H*-pyrrolo[1,2-*a*]imidazole (OSA_000862)

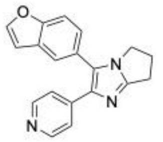

Prepared according to General Procedure D from: **S19** (50.0 mg, 0.19 mmol) and **S4** (60.1 mg, 0.25 mmol); purified by methods 1, then 2, to give *the title compound* as a white powder (32.5 mg, 57%); **^{1}H NMR** (500 MHz, $CDCl_3$): δ 8.39 (d, *J* = 6.1 Hz, 2H), 7.71 (d, *J* = 2.1 Hz, 1H), 7.60 (d, *J* = 1.3 Hz, 1H), 7.58 (d, *J* = 8.5 Hz, 1H), 7.49 – 7.36 (m, 2H), 7.29 (dd, *J* = 8.5, 1.6 Hz, 1H), 6.93 – 6.51 (m, 1H), 3.90 (t, *J* = 7.1 Hz, 2H), 3.03 (t, *J* = 7.6 Hz, 2H), 2.64 (p, *J* = 7.3 Hz, 2H); **^{13}C NMR** (126 MHz, $CDCl_3$): δ 155.0, 154.1, 149.9, 146.2, 142.9, 138.7, 128.39, 128.37, 125.7, 125.3, 122.2, 120.7, 112.4, 106.9, 44.3, 31.1, 26.2, 23.8; ***m/z*** (ESI+) 302 ($[M+H]^+$, 100%); **HRMS** (ESI+) found 302.1286 ($[M+H]^+$), $C_{19}H_{16}N_3O^+$ requires 302.1288.

2-Phenyl-6,7-dihydro-5*H*-pyrrolo[1,2-*a*]imidazole (S20)

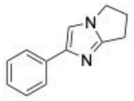

Prepared according to General Procedure A from: 2-bromoacetophenone (1.00 g, 5.02 mmol) and 3,4-dihydro-2*H*-pyrrol-5-amine hydrochloride (1.82 g, 15.1 mmol, 3 equiv.); the residue was diluted in Et_2O, washed with cold H_2O (3 x) and the organic layer concentrated under reduced pressure to give *the title compound* as an orange powder (631 mg, 68%); **^{1}H NMR** (500 MHz, $CDCl_3$): δ 7.74 (d, *J* = 7.2 Hz, 2H), 7.35 (t, *J* = 7.7 Hz, 2H), 7.21 (t, *J* = 7.4 Hz, 1H), 7.18 (s, 1H), 4.01 (t, *J* = 7.1 Hz, 2H), 2.99 – 2.87 (m, 2H), 2.62 (p, *J* = 7.3 Hz, 2H); **^{13}C NMR** (126 MHz, $CDCl_3$): δ 155.0, 146.6, 135.1, 128.7, 126.6, 124.7, 110.4, 45.0, 26.2, 23.3; ***m/z*** (ESI+) 185 ($[M+H]^+$, 100%). Spectroscopic data matched those in the literature.[7]

3-Bromo-2-phenyl-6,7-dihydro-5*H*-pyrrolo[1,2-*a*]imidazole (S21)

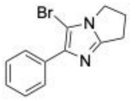

Prepared according to General Procedure C from: **S20** (400 mg, 2.17 mmol) to give *the title compound* as a brown powder (478 mg, 84%); **^{1}H NMR** (500 MHz, $CDCl_3$): δ 7.94 (d, J = 7.3 Hz, 2H), 7.39 (t, J = 7.8 Hz, 2H), 7.28 (d_{app}, J = 7.4 Hz, 2H), 3.98 (t, J = 7.1 Hz, 2H), 3.45 – 2.79 (m, 2H), 2.63 (p, J = 7.3z Hz, 2H); ***m/z*** (ESI+) 263 ($[M+H]^+$, 100%).

3-(Benzofuran-5-yl)-2-phenyl-6,7-dihydro-5*H*-pyrrolo[1,2-*a*]imidazole (OSA_000869)

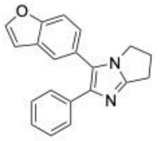

Prepared according to General Procedure D from: **S21** (40.0 mg, 0.15 mmol) and **S4** (48.2 mg, 0.20 mmol); purified by methods 1, then 3, to give *the title compound* as a brown powder (14.2 mg, 31%); **^{1}H NMR** (500 MHz, $CDCl_3$): δ 7.67 (d, J = 2.0 Hz, 1H), 7.65 – 7.58 (m, 1H), 7.56 – 7.48 (m, 3H), 7.32 – 7.26 (m, 1H), 7.21 (t, J = 7.5 Hz, 2H), 7.14 (t, J = 7.3 Hz, 1H), 6.93 – 6.70 (m, 1H), 3.93 (t, J = 7.0 Hz, 2H), 3.02 (t, J = 7.5 Hz, 2H), 2.63 (p, J = 7.2 Hz, 2H); **^{13}C NMR** (101 MHz, $CDCl_3$): δ 154.7, 153.5, 145.8, 141.6, 135.5, 128.3, 128.1, 127.0, 126.33, 126.30, 125.9, 125.7, 121.9, 112.0, 106.9, 44.4, 26.2, 23.9; ***m/z*** (ESI+) 301 ($[M+H]^+$, 100%); **HRMS** (ESI+) found 301.1337 ($[M+H]^+$), $C_{20}H_{17}N_2O^+$ requires 301.1335.

2-(Pyridin-2-yl)imidazo[1,2-*a*]pyridine (S22)

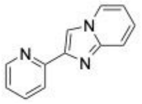

Prepared according to General Procedure A from: 2-bromo-1-(pyridin-2-yl)ethan-1-one hydrobromide (1.50 g, 5.34 mmol) and 2-aminopyridine (750 mg, 7.97 mmol); purified by flash chromatography on silica (1-10% MeOH in CH_2Cl_2) to give *the title compound* as an orange powder (808 mg, 78%); **^{1}H NMR** (500 MHz, $CDCl_3$): δ 8.63 (dq, J = 2.2, 0.9 Hz, 1H), 8.27 (s, 1H), 8.21 (d, J = 7.9 Hz, 1H), 8.16 (dt, J = 1.3 Hz, 1H), 7.80 (td, J =

7.7, 1.9 Hz, 1H), 7.66 (dd, J = 9.1, 0.6 Hz, 1H), 7.26 – 7.19 (m, 2H), 6.82 (td, J = 6.8, 0.9 Hz, 1H); ***m/z*** (ESI+) 196 ([M+H]$^+$, 100%).

3-Bromo-2-(pyridin-2-yl)imidazo[1,2-*a*]pyridine (S23)

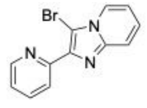

Prepared according to General Procedure C from: **S22** (808 mg, 0.40 mmol) to give *the title compound* as a dark brown powder (1.00 g, 89%); **^{1}H NMR** (500 MHz, $CDCl_3$): δ 8.82 – 8.78 (m, 1H), 8.33 – 8.28 (m, 2H), 7.86 (td, J = 7.7, 1.9 Hz, 1H), 7.82 (d, J = 9.1 Hz, 1H), 7.39 (ddd, J = 9.1, 6.9, 1.1 Hz, 1H), 7.36 – 7.31 (m, 1H), 7.09 – 7.02 (m, 1H); ***m/z*** (ESI+) 274 ([M+H]$^+$, 100%).

3-(Benzo[*b*]thiophen-5-yl)-2-(pyridin-2-yl)imidazo[1,2-*a*]pyridine (OSA_000871)

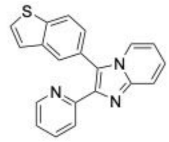

Prepared according to General Procedure D from: **S23** (100 mg, 0.37 mmol) and **S3** (124 mg, 0.48 mmol); purified by methods 1 (1-15% MeOH in CH_2Cl_2, followed by 20-100% EtOAc in hexanes and 0-15% MeOH in CH_2Cl_2), 2, then 3, to give *the title compound* as a dull yellow powder (38.9 mg, 33%); **^{1}H NMR** (500 MHz, $CDCl_3$): δ 8.56 (ddd, J = 4.9, 1.8, 0.9 Hz, 1H), 8.03 (d, J = 8.2 Hz, 1H), 8.01 (d, J = 6.9 Hz, 1H), 7.98 (s, 1H), 7.83 (d, J = 9.1 Hz, 1H), 7.71 (d, J = 7.9 Hz, 1H), 7.63 – 7.49 (m, 2H), 7.44 (dd, J = 8.3, 1.7 Hz, 1H), 7.39 (d, J = 5.4 Hz, 1H), 7.34 – 7.25 (m, 1H), 7.13 (ddd, J = 7.5, 4.9, 1.2 Hz, 1H), 6.80 (t, J = 6.8 Hz, 1H); **^{13}C NMR** (126 MHz, $CDCl_3$): δ 153.4, 149.8, 145.1, 142.0, 140.4, 140.3, 136.1, 127.6, 126.9, 125.9, 125.8, 125.1, 124.2, 123.7, 123.5, 123.2, 122.8, 122.2, 118.3, 112.7; ***m/z*** (ESI+) 328 ([M+H]$^+$, 100%); **HRMS** (ESI+) found 328.0909 ([M+H]$^+$), $C_{20}H_{14}N_3S^+$ requires 328.0903.

2-(Pyridin-2-yl)-3-(*p*-tolyl)imidazo[1,2-*a*]pyridine (OSA_001018)

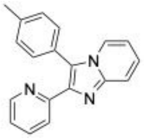

Prepared according to General Procedure D from: **S23** (100 mg, 0.37 mmol) and *p*-tolylboronic acid (59.0 mg, 0.43 mmol); purified by methods 1 (with 5% NH_4OH in MeOH), then 2 (5-100% MeCN in H_2O), to give *the title compound* as a tan powder (51.5 mg, 50%); **^{1}H NMR** (400 MHz, $CDCl_3$): δ 8.59 (ddd, *J* = 4.9, 1.8, 1.0 Hz, 1H), 7.97 (dt, *J* = 7.0, 1.2 Hz, 1H), 7.70 (dt, *J* = 9.1, 1.1 Hz, 1H), 7.63 (dt, *J* = 8.0, 1.2 Hz, 1H), 7.57 (td, *J* = 7.6, 1.8 Hz, 1H), 7.38 (d, *J* = 8.2 Hz, 2H), 7.32 (d, *J* = 8.0 Hz, 2H), 7.19 (ddd, *J* = 9.1, 6.7, 1.3 Hz, 1H), 7.12 (ddd, *J* = 7.3, 4.9, 1.3 Hz, 1H), 6.73 (td, *J* = 6.8, 1.2 Hz, 1H), 2.45 (s, 3H); **^{13}C NMR** (101 MHz, $CDCl_3$): δ 153.5, 149.8, 145.0, 141.8, 138.9, 136.0, 130.8, 130.1, 126.8, 125.0, 123.8, 123.3, 122.8, 122.1, 118.3, 112.6, 21.6; ***m/z*** (ESI+) 286 ($[M+H]^+$, 100%); **HRMS** (ESI+) found 286.1346 ($[M+H]^+$), $C_{19}H_{16}N_3^+$ requires 286.1339.

3-(4-Chlorophenyl)-2-(pyridin-2-yl)imidazo[1,2-*a*]pyridine (S24)

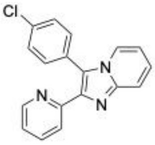

Prepared according to General Procedure D from: **S23** (1.04 g, 3.79 mmol) and (4-chlorophenyl)boronic acid (771 mg, 4.93 mmol); purified by method 3 to give *the title compound* as a white powder (464 mg, 40%); **^{1}H NMR** (300 MHz, DMSO-d_6): δ 8.36 (ddd, *J* = 4.7, 1.8, 0.9 Hz, 1H), 8.10 (ddt, *J* = 8.0, 6.9, 1.2 Hz, 2H), 7.84 (td, *J* = 7.7, 1.8 Hz, 1H), 7.70 (dt, *J* = 9.1, 1.2 Hz, 1H), 7.63 – 7.51 (m, 5H), 7.36 (ddd, *J* = 9.1, 6.7, 1.2 Hz, 1H), 7.26 (ddd, *J* = 7.5, 4.8, 1.3 Hz, 1H), 6.93 (td, *J* = 6.8, 1.3 Hz, 1H); **^{13}C NMR** (75 MHz, DMSO-d_6): δ 153.4, 148.8, 144.1, 136.7, 133.1, 132.8, 128.7, 128.6, 125.9, 124.1, 122.6, 122.2, 121.5, 117.3, 113.2; ***m/z*** (ESI+) 306 ($[M+H]^+$, 100%).

***N*,*N*-Diethyl-4-(2-(pyridin-2-yl)imidazo[1,2-*a*]pyridin-3-yl)aniline (OSA_001011)**

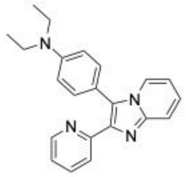

Compound **S24** (100 mg, 0.33 mmol, 1.00 equiv.), diethylamine (0.1 mL, 0.98 mmol, 3.00 equiv.) and NaO*t*-Bu (37.7 mg, 0.39 mmol, 1.20 equiv.) were dissolved in 1,4-dioxane (1.3 mL, 0.25 M). The reaction mixture was degassed under Ar then $Pd(OAc)_2$ (2.94 mg, 13.1 μmol, 0.04 equiv) and RuPhos (3.01 mg, 6.54 μmol, 0.02 equiv) were added and the reaction mixture degassed under Ar again. The reaction mixture was heated at 100 °C overnight, then cooled to room temperature, diluted with EtOAc and filtered through a pad of celite to give the crude product that was purified by reversed-phase flash chromatography on silica (50% MeOH in H_2O) to give *the title compound* as a white powder (18.0 mg, 16%); **^{1}H NMR** (500 MHz, $CDCl_3$): δ 8.66 (ddd, *J* = 4.8, 1.8, 1.0 Hz, 1H), 8.02 (dt, *J* = 6.9, 1.2 Hz, 1H), 7.74 (dt, *J* = 9.2, 1.2 Hz, 1H), 7.59 (dt, *J* = 8.0, 1.2 Hz, 1H), 7.57 – 7.52 (m, 1H), 7.28 (d, *J* = 8.9 Hz, 2H), 7.19 (ddd, *J* = 9.0, 6.7, 1.3 Hz, 1H), 7.12 (ddd, *J* = 7.3, 4.8, 1.3 Hz, 1H), 6.78 (d, *J* = 8.8 Hz, 2H), 6.72 (dd, *J* = 6.8, 1.2 Hz, 1H), 3.43 (q, *J* = 7.1 Hz, 4H), 1.23 (t, *J* = 7.1 Hz, 6H); **^{13}C NMR** (126 MHz, $CDCl_3$): δ 152.9, 149.9, 148.2, 144.3, 140.3, 136.0, 131.9, 125.4, 124.14, 124.06, 122.9, 122.1, 117.9, 114.7, 112.7, 111.9, 44.5, 12.7; ***m/z*** (ESI+) 343 ($[M+H]^+$, 100%); **HRMS** (ESI+) found 343.1925 ($[M+H]^+$), $C_{22}H_{23}N_4^+$ requires 343.1917.

1-Isocyano-4-methylbenzene (S25)

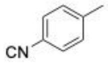

To a vigorously stirred solution of 50% aq. NaOH (12 mL) was added TEBA chloride (125 mg, 0.55 mmol, 1.00 equiv.). The reaction mixture was heated to 40 °C and a solution of *p*-toluidine (59.0 mg, 0.55 mmol, 1.00 equiv.) and $CHCl_3$ (50 μL, 0.624 mmol, 1.13 equiv.) in CH_2Cl_2 (12 mL) was added *via* syringe pump at 0.2 mL/min. The reaction mixture was stirred at 40 ºC. Upon completion as indicated by TLC, the reaction mixture was allowed to cool to rt, and cold H_2O (50–100 mL) was added. The aqueous phase was extracted with CH_2Cl_2 and the organic layer washed with brine, dried ($MgSO_4$), filtered and concentrated under reduced

pressure to give *the title compound* as an orange residue (64.5 mg, quant.); **^{1}H NMR** (400 MHz, $CDCl_3$): δ 7.30 – 7.28 (m, 2H), 7.24 – 7.17 (m, 2H), 2.39 (s, 3H).

2-(Pyridin-2-yl)-*N*-(*p*-tolyl)imidazo[1,2-*a*]pyridin-3-amine (OSA_000988)

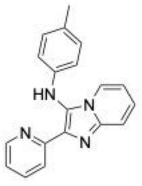

A microwave vial was charged with pyridin-2-amine (25.0 mg, 0.27 mmol, 1.00 equiv.), $Yb(OTf)_3$ (5.00 mg, 0.01 mmol, 0.03 equiv.), picolinaldehyde (50 µL, 0.53 mmol, 1.98 equiv.), and **S25** (65.0 mg, 0.55 mmol, 2.09 equiv.). The vial was closed with a crimp seal and the mixture heated in the microwave at 115 °C for 30 min. The reaction was diluted with EtOAc and the organic layer washed with H_2O, brine, dried ($MgSO_4$), filtered and concentrated under reduced pressure to give the crude product that was purified by flash chromatography on silica (0-10% 5% NH_4OH/MeOH in EtOAc), followed by reversed-phase flash chromatograph on silica (5-100% MeOH in H_2O) to give *the title compound* as a bright yellow powder (11.6 mg, 15%); **^{1}H NMR** (500 MHz, CD_3OD): δ 8.59 (dd, *J* = 5.3, 1.6 Hz, 1H), 8.01 (d, *J* = 8.0 Hz, 1H), 7.90 – 7.77 (m, 2H), 7.61 (d, *J* = 9.1 Hz, 1H), 7.34 (ddd, *J* = 9.0, 6.7, 1.3 Hz, 1H), 7.26 (ddd, *J* = 7.5, 4.9, 1.2 Hz, 1H), 6.97 (d, *J* = 8.1 Hz, 2H), 6.90 (t, *J* = 6.8 Hz, 1H), 6.46 (d, *J* = 8.4 Hz, 2H), 2.20 (s, 3H); **^{13}C NMR** (126 MHz, CD_3OD): δ 154.2, 150.3, 143.3, 143.2, 138.1, 135.1, 131.0, 130.7, 127.0, 125.5, 124.9, 123.5, 122.8, 118.0, 116.2, 113.8, 20.5; ***m/z*** (ESI+) 301 ($[M+H]^+$, 100%); **HRMS** (ESI+) found 301.1456 ($[M+H]^+$), $C_{19}H_{17}N_4^+$ requires 301.1448.

***N*-(4-Fluorophenyl)-2-(pyridin-2-yl)imidazo[1,2-*a*]pyridin-3-amine (OSA_000987)**

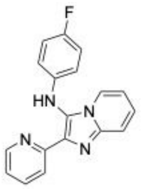

A microwave vial was charged with pyridin-2-amine (50.0 mg, 0.53 mmol, 1.00 equiv.), $Yb(OTf)_3$ (10.0 mg, 0.02 mmol, 0.03 equiv.), picolinaldehyde (118 mg, 1.10 mmol, 2.07 equiv.), and 1-fluoro-4-isocyanobenzene (0.10 mL, 0.98 mmol, 1.85 equiv.). The vial was closed with a crimp seal and the mixture heated in the microwave at

115 °C for 30 min. The reaction was diluted with EtOAc and the organic layer washed with H_2O, brine, dried ($MgSO_4$), filtered and concentrated under reduced pressure to give the crude product that was purified by flash chromatography on silica (20-100% EtOAc in hexanes and 0-10% 5% NH_4OH/MeOH in EtOAc), followed by reversed-phase flash chromatograph on silica (0-100% MeOH in H_2O), then trituration with 1:1 MeOH/H_2O, and finally by flash chromatograph on silica (1-10% 5% NH_4OH/MeOH in CH_2Cl_2) to give *the title compound* as a tan powder (38.4 mg, 24%); **^{1}H NMR** (400 MHz, CD_3OD): δ 8.60 (ddd, *J* = 4.9, 1.8, 0.9 Hz, 1H), 8.03 (dt, *J* = 8.0, 1.1 Hz, 1H), 7.90 (dt, *J* = 7.0, 1.2 Hz, 1H), 7.83 (td, *J* = 7.8, 1.8 Hz, 1H), 7.63 (dt, *J* = 9.2, 1.1 Hz, 1H), 7.37 (ddd, *J* = 9.2, 6.7, 1.3 Hz, 1H), 7.28 (ddd, *J* = 7.5, 4.9, 1.2 Hz, 1H), 6.98 – 6.93 (m, 1H), 6.90 (t, *J* = 8.8 Hz, 2H), 6.61 – 6.50 (m, 2H); **^{13}C NMR** (101 MHz, CD_3OD): δ 158.4 (d, *J* = 236.3 Hz), 153.6, 150.2, 143.5, 142.22 (d, *J* = 2.3 Hz), 142.16 (d, *J* = 2.2 Hz), 138.4, 135.9, 127.4, 124.6, 123.8, 123.1, 118.0, 116.9 (d, *J* = 7.5 Hz), 116.7 (d, *J* = 7.6 Hz), 114.2; ***m/z*** (ESI+) 305 ($[M+H]^+$, 100%); **HRMS** (ESI+) found 305.1212 ($[M+H]^+$), $C_{18}H_{14}FN_4^+$ requires 305.1197.

3-(Benzo[*d*][1,3]dioxol-5-yl)-2-(pyridin-2-yl)imidazo[1,2-*a*]pyridine (OSA_000814)

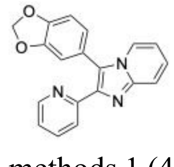

Prepared according to General Procedure D from: **S23** (75.0 mg, 0.27 mmol) and benzo[*d*][1,3]dioxol-5-ylboronic acid (59.0 mg, 0.36 mmol); purified by methods 1 (40-70% EtOAc in hexane), then 3, to give *the title compound* as a pale yellow solid (17.4 mg, 20%); **^{1}H NMR** (400 MHz, $CDCl_3$): δ 8.60 (ddd, *J* = 4.8, 1.8, 1.0 Hz, 1H), 7.99 (dt, *J* = 7.0, 1.2 Hz, 1H), 7.79 (d, *J* = 9.1 Hz, 1H), 7.77 – 7.71 (m, 1H), 7.62 (td, *J* = 7.7, 1.9 Hz, 1H), 7.30 – 7.22 (m, 1H), 7.15 (ddd, *J* = 7.5, 4.8, 1.2 Hz, 1H), 6.95 (dd, *J* = 8.9, 1.1 Hz, 3H), 6.80 (td, *J* = 6.8, 1.2 Hz, 1H), 6.06 (s, 2H); **^{13}C NMR** (101 MHz, $CDCl_3$): δ 152.5, 149.8, 148.5, 148.4, 144.3, 140.7, 136.3, 125.9, 124.9, 123.9, 122.90, 122.86, 122.5, 122.4, 117.9,

113.2, 111.2, 109.3, 101.6; ***m/z*** (ESI+) 316 ([M+H]$^+$, 100%); **HRMS** (ESI+) found 316.1083 ([M+H]$^+$), $C_{19}H_{14}N_3O_2^+$ requires 316.1081.

3-(2-Fluoropyridin-4-yl)-2-(pyridin-2-yl)imidazo[1,2-*a*]pyridine (OSA_000986)

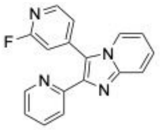

Prepared according to General Procedure D from: **S23** (100 mg, 0.37 mmol) and (2-fluoropyridin-4-yl)boronic acid (67.0 mg, 0.48 mmol); purified by methods 1, then 3 (with MeOH), to give *the title compound* as a light yellow powder (10.4 mg, 10%); **^{1}H NMR** (500 MHz, $CDCl_3$): δ 8.45 (ddd, *J* = 4.8, 1.8, 0.9 Hz, 1H), 8.41 (d, *J* = 5.1 Hz, 1H), 8.25 (d, *J* = 8.0 Hz, 1H), 8.13 (dt, *J* = 6.9, 1.1 Hz, 1H), 8.03 (d, *J* = 9.1 Hz, 1H), 7.82 (td, *J* = 7.8, 1.8 Hz, 1H), 7.50 (t, *J* = 7.9 Hz, 1H), 7.38 (dt, *J* = 5.2, 1.6 Hz, 1H), 7.32 – 7.21 (m, 1H), 7.21 – 7.15 (m, 1H), 7.04 (t, *J* = 6.9 Hz, 1H); **^{13}C NMR** (126 MHz, $CDCl_3$): δ 165.3, 163.3, 149.4, 148.4 (d, *J* = 15.2 Hz), 145.1, 143.0 (d, *J* = 9.2 Hz), 136.9, 127.1, 123.5, 123.3, 123.1, 123.0 (d, *J* = 4.4 Hz), 119.3 (d, *J* = 3.7 Hz), 118.1, 114.3, 111.5, 111.2; ***m/z*** (ESI+) 291 ([M+H]$^+$, 100%); **HRMS** (ESI+) found 291.1052 ([M+H]$^+$), $C_{17}H_{12}FN_4^+$ requires 291.1041.

3-(4-(4-Methylpiperazin-1-yl)phenyl)-2-(pyridin-2-yl)imidazo[1,2-*a*]pyridine (OSA_001012)

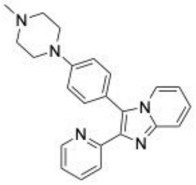

Compound **S24** (100 mg, 0.33 mmol, 1.00 equiv.), 1-methylpiperazine (0.11 mL, 0.98 mmol, 3.00 equiv.) and NaO*t*-Bu (37.7 mg, 0.39 mmol, 1.20 equiv.) were dissolved in 1,4-dioxane (1.3 mL, 0.25 M). The reaction mixture was degassed under Ar then $Pd(OAc)_2$ (2.94 mg, 13.1 μmol, 0.04 equiv) and RuPhos (3.01 mg, 6.54 μmol, 0.02 equiv) were added and the reaction mixture degassed under Ar again. The reaction mixture was heated at 100 °C overnight, then cooled to room temperature, diluted with EtOAc and filtered through a pad of celite to give the crude product that was purified by reversed-phase flash chromatography on silica (50% MeOH in H_2O) to give *the title compound* as a

yellow-orange powder (44.3 mg, 37%); **^{1}H NMR** (500 MHz, $CDCl_3$): δ 8.61 (ddd, *J* = 4.9, 1.8, 1.0 Hz, 1H), 7.96 (dt, *J* = 6.9, 1.2 Hz, 1H), 7.68 (dt, *J* = 9.1, 1.1 Hz, 1H), 7.58 (dt, *J* = 8.0, 1.2 Hz, 1H), 7.54 (td, *J* = 7.6, 1.8 Hz, 1H), 7.35 (d, *J* = 8.8 Hz, 2H), 7.17 (ddd, *J* = 9.0, 6.7, 1.3 Hz, 1H), 7.10 (ddd, *J* = 7.3, 4.8, 1.4 Hz, 1H), 7.03 (d, *J* = 8.8 Hz, 2H), 6.71 (td, *J* = 6.8, 1.2 Hz, 1H), 3.70 – 3.05 (m, 4H), 3.00 – 2.54 (m, 4H), 2.38 (s, 3H); **^{13}C NMR** (126 MHz, $CDCl_3$): δ 153.5, 151.3, 149.8, 144.8, 141.5, 136.0, 131.7, 124.8, 123.8, 123.4, 122.8, 122.0, 119.8, 118.2, 116.0, 112.4, 55.1, 48.4, 46.2; ***m/z*** (ESI+) 370 ($[M+H]^+$, 100%); **HRMS** (ESI+) found 370.2032 ($[M+H]^+$), $C_{23}H_{24}N_5^+$ requires 370.2026.

***N*-(4-Fluorophenyl)-2-(1*H*-pyrrol-2-yl)imidazo[1,2-*a*]pyridin-3-amine (OSA_001009)**

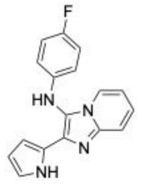

A microwave vial was charged with pyridin-2-amine (200 mg, 2.13 mmol, 1.00 equiv.), $Yb(OTf)_3$ (33.0 mg, 0.05 mmol, 0.03 equiv.), 1*H*-pyrrole-2-carbaldehyde (202 mg, 2.12 mmol, 1.00 equiv.), and 1-fluoro-4-isocyanobenzene (0.22 mL, 2.12 mmol, 1.00 equiv.). The vial was closed with a crimp seal and the mixture heated in the microwave at 160 °C for 5 min. The mixture was dissolved in acetone and the solvent removed under reduced pressure to give the crude product that was purified by flash chromatography on silica (0-100% EtOAc in hexanes), followed by reversed-phase flash chromatography on silica (30-100% MeOH in H_2O) to give a dark brown residue that was dissolved in 1:1 MeOH/H_2O. The volatiles were removed and the suspension filtered and dried *in vacuo* to give *the title compound* as a beige powder (12.1 mg, 2%); insufficient material remaining for complete characterization; **^{1}H NMR** (400 MHz, $CDCl_3$): δ 9.63 (br s, 1H), 7.86 (d, *J* = 6.7 Hz, 1H), 7.54 (d, *J* = 9.1 Hz, 1H), 7.24 – 7.16 (m, 1H), 6.90 (t, *J* = 8.7 Hz, 2H), 6.86 (q, *J* = 2.7 Hz, 1H), 6.79 (t, *J* = 6.7 Hz, 1H), 6.57 – 6.51 (m, 2H), 6.48 (p, *J* = 1.6 Hz, 1H), 6.23 (q, *J* = 2.9 Hz, 1H), 5.41 (br s, 1H); ***m/z*** (ESI+) 293 ($[M+H]^+$, 100%); **HRMS** (ESI+) found 293.1194 ($[M+H]^+$), $C_{17}H_{14}FN_4^+$ requires 293.1197.

N-(4-Fluorophenyl)-2-(thiazol-2-yl)imidazo[1,2-*a*]pyridin-3-amine (OSA_001008)

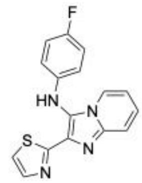

A microwave vial was charged with pyridin-2-amine (200 mg, 2.13 mmol, 1.00 equiv.), $Yb(OTf)_3$ (66.0 mg, 0.11 mmol, 0.05 equiv.), thiazole-2-carbaldehyde (0.4 mL, 4.55 mmol, 2.14 equiv.), and 1-fluoro-4-isocyanobenzene (0.25 mL, 2.46 mmol, 1.16 equiv.). The vial was closed with a crimp seal and the mixture heated in the microwave at 115 °C for 30 min. The reaction was diluted with EtOAc and the organic layer washed with H_2O, brine, dried ($MgSO_4$), filtered and concentrated under reduced pressure to give the crude product that was purified by flash chromatography on silica (20-100% EtOAc in hexanes), followed by trituration with MeOH to give *the title compound* as a tan powder (216 mg, 33%); **^{1}H NMR** (500 MHz, $CDCl_3$): δ 7.82 (d, *J* = 3.2 Hz, 1H), 7.63 (dd, *J* = 14.4, 8.0 Hz, 2H), 7.36 (br s, 1H), 7.30 (d, *J* = 3.2 Hz, 1H), 7.22 (ddd, *J* = 9.1, 6.6, 1.3 Hz, 1H), 7.07 – 6.88 (m, 2H), 6.79 (t, *J* = 6.7 Hz, 1H), 6.71 – 6.48 (m, 2H); **^{13}C NMR** (101 MHz, $CDCl_3$): δ 163.7, 158.1 (d, *J* = 239.8 Hz), 143.7, 142.0, 139.21, 139.19, 129.3, 124.8, 123.74, 123.68, 118.3, 117.9 (d, *J* = 7.7 Hz), 116.2 (d, *J* = 22.8 Hz), 112.7; ***m/z*** (ESI+) 311 ($[M+H]^+$, 100%); **HRMS** (ESI+) found 311.0769 ($[M+H]^+$), $C_{16}H_{12}FN_4S^+$ requires 311.0761.

7-Chloro-2-(pyridin-2-yl)imidazo[1,2-*a*]pyridine (S26)

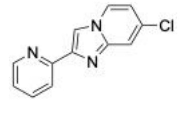

Prepared according to General Procedure A from: 2-bromo-1-(pyridin-2-yl)ethan-1-one hydrobromide (700 mg, 2.49 mmol) and 4-chloropyridin-2-amine (480 mg, 3.73 mmol); purified by flash chromatography on silica (1-10% 5% NH_4OH/MeOH in CH_2Cl_2), followed by flash chromatography on silica (30-100% EtOAc in hexanes) to give *the title compound* as a pale yellow powder (208 mg, 36%); **^{1}H NMR** (500 MHz, $CDCl_3$): δ 8.66 (d, *J* = 5.0 Hz, 1H), 8.59 (br s, 1H), 8.34 (d, *J* = 7.9 Hz, 1H), 8.14 (d, *J* =

7.3 Hz, 1H), 7.94 (d, J = 7.9 Hz, 1H), 7.72 (s, 1H), 7.38 (dd, J = 6.3 Hz, 1H), 6.88 (dd, J = 7.3, 1.6 Hz, 1H); ***m/z*** (ESI+) 230 ($[M+H]^+$, 100%).

3-Bromo-7-chloro-2-(pyridin-2-yl)imidazo[1,2-*a*]pyridine (S27)

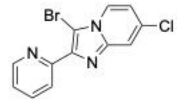

Prepared according to General Procedure C from: **S26** (208 mg, 0.91 mmol) to give *the title compound* as an orange powder (162 g, 58%); **^{1}H NMR** (500 MHz, $CDCl_3$): δ 8.86 (d, J = 4.7 Hz, 1H), 8.31 (d, J = 8.2 Hz, 1H), 8.21 (d, J = 7.3 Hz, 1H), 7.97 – 7.91 (m, 1H), 7.80 – 7.74 (m, 1H), 7.44 – 7.37 (m, 1H), 7.02 (dd, J = 7.3, 1.6 Hz, 1H); ***m/z*** (ESI+) 308 ($[M+H]^+$, 100%).

7-Chloro-2-(pyridin-2-yl)-3-(*p*-tolyl)imidazo[1,2-*a*]pyridine (OSA_000978)

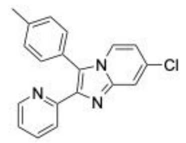

Prepared according to General Procedure D from: **S27** (75.0 mg, 0.28 mmol) and *p*-tolylboronic acid (40.0 mg, 0.29 mmol); purified by methods 1 (with 5% NH_4OH in MeOH), then 2 (5-100% MeCN in H_2O), to give *the title compound* as a tan powder (26.8 mg, 34%); **^{1}H NMR** (500 MHz, $CDCl_3$): δ 8.61 (d, J = 4.9 Hz, 1H), 7.91 (d, J = 7.4 Hz, 1H), 7.86 (br s, 1H), 7.67 (d, J = 7.9 Hz, 1H), 7.62 (t, J = 7.4 Hz, 1H), 7.36 (s, 4H), 7.19 – 7.08 (m, 1H), 6.80 (dd, J = 7.4, 2.1 Hz, 1H), 2.47 (s, 3H); **^{13}C NMR** (126 MHz, $CDCl_3$): δ 151.6, 149.8, 143.9, 140.7, 139.8, 136.5, 132.9, 130.7, 130.3, 125.3, 124.3, 123.7, 123.0, 122.8, 116.5, 115.2, 21.7; ***m/z*** (ESI+) 320 ($[M+H]^+$, 100%); **HRMS** (ESI+) found 320.0949 ($[M+H]^+$), $C_{19}H_{15}ClN_3^+$ requires 320.0949.

3-(Benzo[*b*]thiophen-5-yl)-2-phenyl-6,7-dihydro-5*H*-pyrrolo[1,2-*a*]imidazole (OSA_000834)

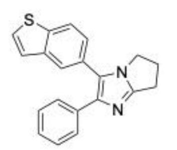

Prepared according to General Procedure D from: **S21** (50.0 mg, 0.19 mmol) and **S3** (64.3 mg, 0.25 mmol); purified by methods 1, then 3, to give *the title*

compound as a light beige powder (10.3 mg, 17%); **^{1}H NMR** (500 MHz, $CDCl_3$): δ 7.88 (d, *J* = 8.3 Hz, 1H), 7.85 – 7.79 (m, 1H), 7.56 (d, *J* = 7.3 Hz, 2H), 7.50 (d, *J* = 5.4 Hz, 1H), 7.39 – 7.31 (m, 2H), 7.22 (t, *J* = 7.5 Hz, 2H), 7.16 (t, *J* = 7.3 Hz, 1H), 3.96 (t, *J* = 7.0 Hz, 2H), 3.03 (t, *J* = 7.5 Hz, 2H), 2.64 (p, *J* = 7.3 Hz, 2H); **^{13}C NMR** (101 MHz, $CDCl_3$): δ 153.8, 141.9, 140.2, 139.3, 135.5, 128.3, 127.7, 127.4, 127.2, 126.5, 125.6, 125.5, 124.1, 123.9, 123.1, 44.6, 26.3, 23.9; ***m/z*** (ESI+) 317 ($[M+H]^+$, 100%); **HRMS** (ESI+) found 317.1106 ($[M+H]^+$), $C_{20}H_{17}N_2S^+$ requires 317.1107.

3-(4-Fluorophenyl)-2-(pyridin-2-yl)-6,7-dihydro-5*H*-pyrrolo[1,2-*a*]imidazole (OSA_001053)

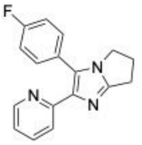

Prepared according to General Procedure E from: **S2** (50.0 mg, 0.19 mmol) and 4-fluorophenylboronic acid (39.7 mg, 0.28 mmol); purified by reversed-phase flash chromatography on silica (5-100% MeOH in H_2O) to afford *the title compound* as a brown solid (16.9 mg, 30%); **^{1}H NMR** (400 MHz, $CDCl_3$): δ 8.45 (d, *J* = 4.8 Hz, 1H), 7.60 – 7.48 (m, 2H), 7.45 – 7.34 (m, 2H), 7.14 – 6.99 (m, 3H), 3.93 (t, *J* = 7.0 Hz, 2H), 3.00 (t, J = 7.6 Hz, 2H), 2.63 (p, *J* = 6.9 Hz, 2H); **^{13}C NMR** (101 MHz, $CDCl_3$): δ 163.8, 161.4, 154.2, 154.0, 149.3, 141.3, 136.2, 131.3 (d, *J* = 8.2 Hz), 127.2 (d, *J* = 3.4 Hz), 127.1, 121.4, 115.7 (d, *J* = 21.6 Hz), 44.6, 26.3, 23.7; ***m/z*** (ESI+) 280 ($[M+H]^+$, 100%); **HRMS** (ESI+) found 280.1242 ($[M+H]^+$), $C_{17}H_{15}FN_3^+$ requires 280.1245.

2-(Pyridin-2-yl)-3-(pyridin-4-yl)-6,7-dihydro-5*H*-pyrrolo[1,2-*a*]imidazole (OSA_001072)

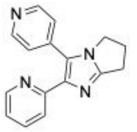

Prepared according to General Procedure E from: **S2** (50.0 mg, 0.19 mmol) and 4-pyridineboronic acid (34.9 mg, 0.28 mmol); purified by reversed-phase flash chromatography on silica (5-100% MeOH in H_2O) to afford *the title compound* as a yellow solid (14.8 mg, 28%); **^{1}H NMR** (400 MHz, $CDCl_3$): δ 8.60 (d, *J* = 4.7 Hz, 2H), 8.44 (d, *J* = 4.8

Hz, 1H), 7.78 (d, *J* = 7.9 Hz, 1H), 7.65 (t, *J* = 7.8 Hz, 1H), 7.38 (d, *J* = 4.7 Hz, 2H), 7.12 (t, *J* = 6.2 Hz, 1H), 4.04 (t, *J* = 7.1 Hz, 2H), 3.02 (t, *J* = 7.6 Hz, 2H), 2.67 (p, *J* = 7.4 Hz, 2H); **^{13}C NMR** (126 MHz, $CDCl_3$): δ 155.4, 153.8, 149.8, 149.1, 143.5, 139.0, 136.5, 125.1, 123.4, 122.1, 121.8, 45.2, 26.3, 23.6; ***m/z*** (ESI+) 263 ($[M+H]^+$, 100%); **HRMS** (ESI+) found 263.1294 ($[M+H]^+$), $C_{16}H_{15}N_4^+$ requires 263.1291.

3-(2-Fluoropyridin-4-yl)-2-(pyridin-2-yl)-6,7-dihydro-5*H*-pyrrolo[1,2-*a*]imidazole (OSA_001073)

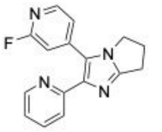

Prepared according to General Procedure E from: **S2** (50.0 mg, 0.19 mmol) and 2-fluoro-4-pyridylboronic acid (40.0 mg, 0.28 mmol); purified by reversed-phase flash chromatography on silica (5-100% MeOH in H_2O) to afford *the title compound* as a brown solid (11.7 mg, 22%); **^{1}H NMR** (400 MHz, $CDCl_3$): δ 8.43 (d, *J* = 4.8 Hz, 1H), 8.18 (d, *J* = 5.3 Hz, 1H), 7.84 (d, *J* = 7.9 Hz, 1H), 7.69 (td, *J* = 7.8, 1.9 Hz, 1H), 7.29 – 7.25 (m, 1H), 7.14 (dd, *J* = 7.5, 4.9 Hz, 1H), 7.07 (s, 1H), 4.06 (t, *J* = 7.0 Hz, 2H), 3.02 (t, *J* = 7.6 Hz, 2H), 2.69 (p, *J* = 7.3 Hz, 2H); **^{13}C NMR** (126 MHz, $CDCl_3$): δ 164.0 (d, *J* = 237.5 Hz), 155.8, 153.6, 149.1, 147.5 (d, *J* = 15.6 Hz), 144.1 (d, *J* = 8.2 Hz), 136.7, 132.2, 128.7, 122.4, 122.0, 121.3 (d, *J* = 4.0 Hz), 108.9 (d, *J* = 38.6 Hz), 45.5, 26.3, 23.6; ***m/z*** (ESI+) 281 ($[M+H]^+$, 100%); **HRMS** (ESI+) found 281.1203 ($[M+H]^+$), $C_{16}H_{14}FN_4^+$ requires 281.1197.

4-(2-(Pyridin-2-yl)-6,7-dihydro-5*H*-pyrrolo[1,2-*a*]imidazol-3-yl)aniline (OSA_001074)

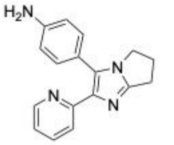

Prepared according to General Procedure E with $PdCl_2(dppf)$ (23.9 mg, 0.03 mmol, 0.15 equiv.) and K_2CO_3 (121 mg, 0.87 mmol, 4.00 equiv.) instead from: **S2** (57.6 mg, 0.22 mmol) and (4-aminophenyl)boronic acid (38.8 mg, 0.28 mmol); purified by reversed-phase flash chromatography on silica (5-100% MeOH in H_2O) to afford *the title compound* as a brown solid (18.8 mg, 31%); **^{1}H NMR** (400 MHz, DMSO-d_6): δ 8.32 (dd, *J* =

6.0, 3.1 Hz, 1H), 7.74 (d, *J* = 8.0 Hz, 1H), 7.68 (td, *J* = 7.6, 2.0 Hz, 1H), 7.15 (d, *J* = 8.1 Hz, 2 H), 7.09 (dd, *J* = 7.1, 5.1 Hz, 1H), 6.56 (d, *J* = 8.0 Hz, 2H), 5.23 (br s, 2H), 3.90 (t, *J* = 7.0 Hz, 2H), 2.82 (t, *J* = 7.5 Hz, 2H), 2.52 (p, 2H); **^{13}C NMR** (101 MHz, DMSO-d_6): δ 155.0, 152.4, 148.2, 148.1, 138.6, 135.9, 130.0, 128.5, 120.7, 120.6, 118.0, 113.2, 43.9, 25.7, 22.9; ***m/z*** (ESI+) 277 ([M+H]$^+$, 100%); **HRMS** (ESI+) found 277.1460 ([M+H]$^+$), $C_{17}H_{17}N_4^+$ requires 277.1448.

Mono(3-(6-cyanopyridin-1-ium-3-yl)-2-(pyridin-2-yl)-6,7-dihydro-5*H*-pyrrolo[1,2-*a*]imidazol-1-ium) monoamide (OSA_001075)

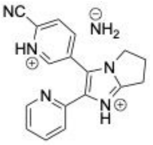

Prepared according to General Procedure E with $Pd(PPh_3)_4$ (47.4 mg, 0.04 mmol, 0.15 equiv.) and K_2CO_3 (151 mg, 1.09 mmol, 4.00 equiv.) instead from: **S2** (72.0 mg, 0.27 mmol) and (6-cyanopyridin-3-yl)boronic acid (52.4 mg, 0.35 mmol); purified by reversed-phase flash chromatography on silica (5-100% MeOH in H_2O) to afford *the title compound* as a brown solid (15.5 mg, 20%); **^{1}H NMR** (500 MHz, $CDCl_3$): δ 8.70 (d, *J* = 2.1 Hz, 1H), 8.37 (dd, *J* = 5.2, 1.8 Hz, 1H), 8.22 (d, *J* = 8.1 Hz, 1H), 7.98 (dd, *J* = 8.2, 2.2 Hz, 1H), 7.88 (d, *J* = 8.0 Hz, 1H), 7.82 (br, 1H), 7.67 (td, *J* = 7.7, 1.9 Hz, 1H), 7.11 (ddd, *J* = 7.5, 4.8, 1.2 Hz, 1H), 5.60 (br, 1H), 4.03 (t, *J* = 7.1 Hz, 2H), 3.04 (t, *J* = 7.6 Hz, 2H), 2.69 (p, 2H); **^{13}C NMR** (126 MHz, $CDCl_3$): δ 166.6, 155.5, 153.8, 149.0, 148.8, 147.9, 143.3, 137.5, 136.6, 130.5, 123.9, 122.1, 122.0, 121.2, 45.0, 26.3, 23.7; ***m/z*** (ESI+) 323 ([M+NH$_4$]$^+$, 100%); **HRMS** (ESI+) found 306.1368 ([M+H]$^+$), $C_{17}H_{18}N_6^+$ requires 306.1587.

2-Methoxy-4-(2-(pyridin-2-yl)-6,7-dihydro-5*H*-pyrrolo[1,2-*a*]imidazol-3-yl)benzonitrile (OSA_001076)

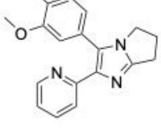

Prepared according to General Procedure E with $PdCl_2(dppf)$ (23.9 mg, 0.03 mmol, 0.15 equiv.) and K_2CO_3 (121 mg, 0.87 mmol, 4.00 equiv.) instead from:

S2 (57.6 mg, 0.22 mmol) and (4-cyano-3-methoxyphenyl)boronic acid (38.8 mg, 0.28 mmol); purified by reversed-phase flash chromatography on silica (5-100% MeOH in H_2O) to afford *the title compound* as a brown solid (24.0 mg, 35%); **^{1}H NMR** (400 MHz, $CDCl_3$): δ 8.41 (d, *J* = 4.7 Hz, 1H), 7.79 (d, *J* = 8.0 Hz, 1H), 7.65 (td, *J* = 7.8, 1.9 Hz, 1H), 7.54 (d, *J* = 8.0 Hz, 1H), 7.25 (d, *J* = 4.4 Hz, 1H), 7.10 (ddd, *J* = 7.7, 4.9, 1.4 Hz, 1H), 7.04 (dd, *J* = 7.9, 1.6 Hz, 1H), 4.01 (t, *J* = 7.0 Hz, 2H), 3.83 (s, 3H), 3.00 (t, *J* = 7.6 Hz, 2H), 2.66 (p, 2H); **^{13}C NMR** (101 MHz, $CDCl_3$): δ 161.0, 155.1, 154.0, 148.9, 143.0, 137.4, 136.5, 133.5, 126.4, 122.0, 120.9, 116.7, 113.1, 100.7, 56.1, 45.3, 26.3, 23.6; ***m/z*** (ESI+) 317 ($[M+H]^+$, 100%); **HRMS** (ESI+) found 317.1406 ($[M+H]^+$), $C_{19}H_{17}N_4O^+$ requires 317.1397.

4-(2-(Pyridin-2-yl)-6,7-dihydro-5*H*-pyrrolo[1,2-*a*]imidazol-3-yl)-2-(trifluoromethyl)benzonitrile (OSA_001077)

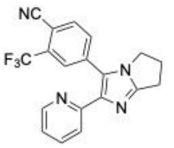

Prepared according to General Procedure E with $PdCl_2$(dppf) (41.6 mg, 0.06 mmol, 0.15 equiv.) and K_2CO_3 (212 mg, 1.54 mmol, 4.00 equiv.) instead from: **S2** (102 mg, 0.38 mmol) and (4-cyano-3-(trifluoromethyl)phenyl)boronic acid (124 mg, 0.58 mmol); purified by reversed-phase flash chromatography on silica (5-100% MeOH in H_2O) to afford *the title compound* as a brown solid (40.0 mg, 30%); **^{1}H NMR** (500 MHz, $CDCl_3$): δ 8.37 – 8.28 (m, 1H), 8.01 (s, 1H), 7.92 (d, *J* = 8.0 Hz, 1H), 7.83 – 7.75 (m, 2H), 7.69 (td, *J* = 7.7, 1.9 Hz, 1H), 7.13 (dd, *J* = 7.5, 4.8 Hz, 1H), 4.03 (t, *J* = 7.1 Hz, 2H), 3.03 (t, *J* = 7.6 Hz, 2H), 2.69 (p, *J* = 7.3 Hz, 2H); **^{13}C NMR** (126 MHz, $CDCl_3$): δ 155.9, 153.5, 148.7, 143.8, 136.7, 136.3, 134.4, 132.5 (q, *J* = 32.4 Hz), 132.2, 127.7 (q, *J* = 4.6 Hz), 124.9, 123.6, 122.4, 121.5, 115.8, 108.1, 45.3, 26.3, 23.6; ***m/z*** (ESI+) 355 ($[M+H]^+$, 100%); **HRMS** (ESI+) found 355.1170 ($[M+H]^+$), $C_{19}H_{14}F_3N_4^+$ requires 355.1165.

3-(Benzo[*d*][1,3]dioxol-5-yl)-2-(pyridin-3-yl)-6,7-dihydro-5*H*-pyrrolo[1,2-*a*]imidazole (OSA_000816)

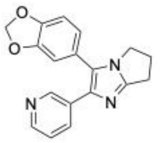

Prepared according to General Procedure D from: **S17** (75.0 mg, 0.28 mmol) and benzo[*d*][1,3]dioxol-5-ylboronic acid (57.0 mg, 0.34 mmol); purified by methods 1 (40-85% EtOAc in hexane), then 3, to give *the title compound* as a yellow solid (30.8 mg, 35%); **^{1}H NMR** (400 MHz, $CDCl_3$): δ 8.70 (dd, *J* = 2.3, 0.9 Hz, 1H), 8.37 (dd, *J* = 4.8, 1.7 Hz, 1H), 7.92 (ddd, *J* = 8.0, 2.3, 1.7 Hz, 1H), 7.19 (ddd, *J* = 7.9, 4.8, 0.9 Hz, 1H), 6.86 – 6.73 (m, 3H), 5.99 (s, 2H), 3.90 (t, 2H), 2.99 (t, 2H), 2.67 – 2.55 (m, 2H); **^{13}C NMR** (101 MHz, $CDCl_3$): δ 153.9, 148.3, 147.93, 147.89, 147.1, 137.9, 134.2, 131.1, 126.2, 124.0, 123.4, 122.9, 109.4, 109.2, 101.5, 44.4, 26.2, 23.7; ***m/z*** (ESI+) 306 ($[M+H]^+$, 100%); **HRMS** (ESI+) found 306.1240 ($[M+H]^+$), $C_{18}H_{16}N_3O_2^+$ requires 306.1237.

3-(Benzo[*d*][1,3]dioxol-5-yl)-2-(pyridin-4-yl)-6,7-dihydro-5*H*-pyrrolo[1,2-*a*]imidazole (OSA_000817)

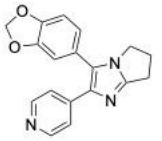

Prepared according to General Procedure D from: **S19** (75.0 mg, 0.28 mmol) and benzo[*d*][1,3]dioxol-5-ylboronic acid (57.0 mg, 0.34 mmol); purified by methods 1 (60-100% EtOAc in hexane), then 3, to give *the title compound* as a yellow solid (29.0 mg, 33%); **^{1}H NMR** (400 MHz, $CDCl_3$): δ 8.43 – 8.37 (m, 2H), 7.51 – 7.45 (m, 2H), 6.91 – 6.76 (m, 3H), 6.03 (s, 2H), 3.86 (t, *J* = 7.1 Hz, 2H), 2.97 (t, *J* = 7.6 Hz, 2H), 2.61 (p, *J* = 7.4 Hz, 2H); **^{13}C NMR** (101 MHz, $CDCl_3$): δ 154.2, 148.9, 148.4, 148.2, 143.5, 138.3, 128.2, 123.9, 123.2, 120.8, 109.4, 109.2, 101.6, 44.2, 26.2, 23.7; ***m/z*** (ESI+) 306 ($[M+H]^+$, 100%); **HRMS** (ESI+) found 306.1239 ($[M+H]^+$), $C_{18}H_{16}N_3O_2^+$ requires 306.1237.

6-(Pyridin-2-yl)-2,3-dihydroimidazo[2,1-*b*]thiazole (S28)

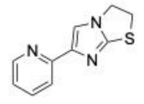

Prepared according to General Procedure A from: 2-bromo-1-(pyridin-2-yl)ethan-1-one hydrobromide (1.10 g, 3.92 mmol) and 4,5-dihydrothiazol-2-amine (500 mg, 4.89 mmol); purified by flash chromatography on silica (0-10% MeOH in CH_2Cl_2) to give *the title compound* as an orange solid (509 mg, 64%); **^{1}H NMR** (400 MHz, $CDCl_3$): δ 8.51 – 8.45 (m, 1H), 7.89 (d, J = 8.0 Hz, 1H), 7.71 (s, 1H), 7.68 (dd, J = 7.8, 1.6 Hz, 1H), 7.15 – 7.07 (m, 1H), 4.20 (t, J = 7.3 Hz, 2H), 3.82 (t, J = 7.2 Hz, 2H); **^{13}C NMR** (101 MHz, $CDCl_3$): δ 152.7, 150.6, 148.6, 146.9, 137.3, 121.7, 119.3, 116.0, 46.3, 34.8.

5-Bromo-6-(pyridin-2-yl)-2,3-dihydroimidazo[2,1-*b*]thiazole (S29)

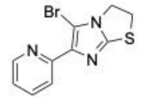

Prepared according to General Procedure C from: **S28** (509 mg, 2.51 mmol) to give *the title compound* as a pale yellow solid (663 mg, 94%); **^{1}H NMR** (400 MHz, $CDCl_3$): δ 8.66 (ddd, J = 4.9, 1.8, 0.9 Hz, 1H), 7.99 – 7.88 (m, 1H), 7.72 (ddd, J = 8.0, 7.5, 1.9 Hz, 1H), 7.22 – 7.13 (m, 1H), 4.20 (t, J = 7.3 Hz, 2H), 3.85 (t, J = 7.3 Hz, 2H); **^{13}C NMR** (101 MHz, $CDCl_3$): δ 151.6, 149.8, 148.6, 141.8, 137.2, 121.9, 120.9, 98.8, 46.2, 34.0.

5-(Benzo[*d*][1,3]dioxol-5-yl)-6-(pyridin-2-yl)-2,3-dihydroimidazo[2,1-*b*]thiazole (OSA_000813)

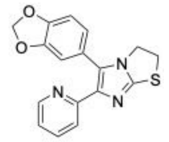

Prepared according to General Procedure D from: **S29** (70.0 g, 0.25 mmol) and benzo[*d*][1,3]dioxol-5-ylboronic acid (54.0 mg, 0.32 mmol); purified by methods 1 (20-60% EtOAc in hexane), then 3, to give *the title compound* as a yellow solid (15.3 mg, 19%); **^{1}H NMR** (400 MHz, $CDCl_3$): δ 8.61 – 8.54 (m, 1H), 7.63 (td, J = 7.8, 1.8 Hz, 1H), 7.55 (dt, J = 8.1, 1.1 Hz, 1H), 7.17 – 7.09 (m, 1H), 6.93 – 6.80 (m, 3H), 6.01 (s, 2H), 4.12 (t, J = 7.2 Hz, 2H), 3.85 (t, J = 7.2 Hz, 2H); **^{13}C NMR** (101 MHz, $CDCl_3$): δ 151.7, 149.8, 148.3, 148.10, 148.08, 139.8, 137.6, 130.6, 123.5, 123.4, 121.7, 121.5, 110.0, 108.9, 101.6,

45.9, 35.0; ***m/z*** (ESI+) 324 ($[M+H]^+$, 100%); **HRMS** (ESI+) found 324.0804 ($[M+H]^+$), $C_{17}H_{14}N_3O_2S^+$ requires 324.0801.

2-(Pyridin-2-yl)-5,6,7,8-tetrahydroimidazo[1,2-*a*]pyridine (S30)

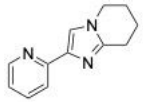

Prepared according to General Procedure A from: 2-bromo-1-(pyridin-2-yl)ethan-1-one hydrobromide (1.00 g, 2.56 mmol) and 3,4,5,6-tetrahydropyridin-2-amine hydrochloride (958 mg, 7.12 mmol); purified by flash chromatography on silica (0-10% MeOH in CH_2Cl_2) to give *the title compound* as an orange solid (355 mg, 50%); **^{1}H NMR** (400 MHz, $CDCl_3$): δ 8.49 (ddd, *J* = 4.9, 1.8, 0.9 Hz, 1H), 7.93 (dt, *J* = 8.0, 1.1 Hz, 1H), 7.66 (td, *J* = 7.7, 1.8 Hz, 1H), 7.47 (s, 1H), 7.08 (ddd, *J* = 7.5, 4.9, 1.2 Hz, 1H), 3.99 (t, *J* = 5.8 Hz, 2H), 2.94 (t, *J* = 6.2 Hz, 2H), 2.05 – 1.89 (m, 4H); **^{13}C NMR** (101 MHz, $CDCl_3$): δ 153.1, 149.1, 145.5, 140.4, 136.8, 121.5, 119.2, 117.2, 45.2, 24.6, 23.1, 21.1.

3-Bromo-2-(pyridin-2-yl)-5,6,7,8-tetrahydroimidazo[1,2-*a*]pyridine (S31)

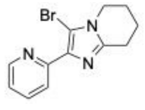

Prepared according to General Procedure C from: **S30** (355 mg, 1.78 mmol) to give *the title compound* as a brown solid (332 mg, 67%); **^{1}H NMR** (400 MHz, $CDCl_3$): δ 8.67 (ddd, *J* = 4.9, 1.8, 1.0 Hz, 1H), 8.00 (dt, *J* = 8.0, 1.1 Hz, 1H), 7.70 (td, *J* = 7.8, 1.9 Hz, 1H), 7.16 (ddd, *J* = 7.5, 4.9, 1.2 Hz, 1H), 3.91 (t, *J* = 6.0 Hz, 2H), 2.97 (t, *J* = 6.4 Hz, 2H), 2.10 – 2.00 (m, 2H), 2.00 – 1.89 (m, 2H); **^{13}C NMR** (101 MHz, $CDCl_3$): δ 151.9, 149.3, 146.5, 136.6, 135.9, 121.9, 121.2, 101.0, 44.6, 25.1, 22.8, 20.6.

3-(Benzo[*d*][1,3]dioxol-5-yl)-2-(pyridin-2-yl)-5,6,7,8-tetrahydroimidazo[1,2-*a*]pyridine (OSA_000818)

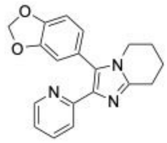

Prepared according to General Procedure D from: **S31** (80.0 mg, 0.29 mmol) and benzo[*d*][1,3]dioxol-5-ylboronic acid (57.0 mg, 0.35 mmol); purified by

methods 1 (70-100% EtOAc in hexane), then 3, to give *the title compound* as a pale yellow solid (17.0 mg, 18%); **^{1}H NMR** (400 MHz, $CDCl_3$): δ 8.54 – 8.48 (m, 1H), 7.48 (td, *J* = 7.7, 1.9 Hz, 1H), 7.40 (d, *J* = 8.0 Hz, 1H), 7.01 (ddd, *J* = 7.3, 4.9, 1.2 Hz, 1H), 6.86 (d, *J* = 8.3 Hz, 1H), 6.84 – 6.78 (m, 2H), 6.02 (s, 2H), 3.73 – 3.65 (m, 2H), 3.10 – 3.02 (m, 2H), 2.00 – 1.90 (m, 4H); **^{13}C NMR** (101 MHz, $CDCl_3$): δ 152.8, 149.6, 148.04, 147.97, 144.9, 135.9, 135.7, 129.5, 124.7, 123.7, 121.4, 121.2, 111.1, 108.8, 101.5, 44.1, 24.7, 23.0, 20.6; ***m/z*** (ESI+) 320 ($[M+H]^+$, 100%); **HRMS** (ESI+) found 320.1397 ($[M+H]^+$), $C_{19}H_{18}N_3O_2^+$ requires 320.1394.

3-(Benzofuran-5-yl)-7-chloro-2-(pyridin-2-yl)imidazo[1,2-*a*]pyridine (OSA_000985)

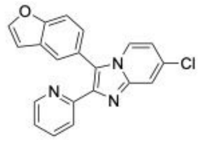

Prepared according to General Procedure D from: **S27** (75.0 mg, 0.28 mmol) and **S4** (72.0 mg, 0.30 mmol); purified by methods 1, then 2, to give *the title compound* as a light beige powder (14.0 mg, 17%); **^{1}H NMR** (500 MHz, $CDCl_3$): δ 8.59 (d, *J* = 4.1 Hz, 1H), 8.02 (br s, 1H), 7.90 (d, *J* = 7.3 Hz, 1H), 7.76 (d, *J* = 2.2 Hz, 1H), 7.74 (d, *J* = 1.7 Hz, 1H), 7.70 (t, *J* = 8.2 Hz, 1H), 7.62 (td, *J* = 7.7, 1.8 Hz, 1H), 7.38 (dd, *J* = 8.5, 1.8 Hz, 1H), 7.19 (dd, *J* = 6.3, 4.7 Hz, 1H), 6.87 (dd, *J* = 7.2, 2.0 Hz, 1H), 6.86 (dd, *J* = 2.2, 0.9 Hz, 1H); **^{13}C NMR** (126 MHz, $CDCl_3$): δ 155.5, 150.5, 149.8, 146.5, 143.3, 139.4, 136.9, 134.2, 128.8, 127.1, 124.4, 124.1, 123.9, 123.2, 123.0, 122.3, 116.1, 115.9, 113.0, 107.0; ***m/z*** (ESI+) 346 ([M+H]+, 100%); **HRMS** (ESI+) found 346.0739 ($[M+H]^+$), $C_{20}H_{13}ClN_3O^+$ requires 346.0742.

2-(Pyridin-2-yl)imidazo[1,2-*a*]pyridine-7-carbonitrile (S32)

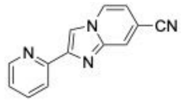

Prepared according to General Procedure A from: 2-bromo-1-(pyridin-2-yl)ethan-1-one hydrobromide (500 mg, 1.78 mmol) and 2-aminoisonicotinonitrile (318 mg, 2.67 mmol); purified by flash chromatography on silica (1-10% MeOH in CH_2Cl_2); repurified by flash chromatography on silica (25-100% EtOAc in

hexanes) to give *the title compound* as a brown powder (64.9 mg, 17%); **^{1}H NMR** (400 MHz, $CDCl_3$): δ 8.65 (d, *J* = 4.5 Hz, 1H), 8.42 (s, 1H), 8.32 – 8.22 (m, 2H), 8.04 (s, 1H), 7.84 (t, *J* = 8.0 Hz, 1H), 7.48 – 7.28 (m, 1H), 7.09 – 6.91 (m, 1H); ***m/z*** (ESI+) 221 ($[M+H]^+$, 100%).

3-Bromo-2-(pyridin-2-yl)imidazo[1,2-*a*]pyridine-7-carbonitrile (S33)

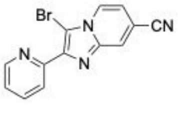

Prepared according to General Procedure C from: **S32** (55.0 mg, 0.25 mmol) to give *the title compound* as a brown powder (71.0 mg, 95%); **^{1}H NMR** (500 MHz, $CDCl_3$): δ 8.80 (d, *J* = 4.8 Hz, 1H), 8.35 (d, *J* = 7.1 Hz, 1H), 8.25 (q, *J* = 7.4 Hz, 1H), 8.06 (s, 1H), 7.86 (td, *J* = 7.8, 1.8 Hz, 1H), 7.64 – 7.29 (m, 1H), 7.12 (dt, *J* = 7.2, 1.8 Hz, 1H); ***m/z*** (ESI+) 299 ($[M+H]^+$, 100%).

2-(Pyridin-2-yl)-3-(*p*-tolyl)imidazo[1,2-*a*]pyridine-7-carbonitrile (OSA_001013)

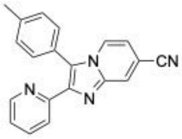

Prepared according to General Procedure D from: **S33** (50.0 mg, 0.17 mmol) and *p*-tolylboronic acid (29.5 mg, 0.22 mmol); purified by methods 1, then 3, to give *the title compound* as an orange powder (24.7 mg, 48%); **^{1}H NMR** (400 MHz, $CDCl_3$): δ 8.64 (d, *J* = 4.8 Hz, 1H), 8.13 (s, 1H), 8.05 (d, *J* = 7.2 Hz, 1H), 7.69 – 7.62 (m, 2H), 7.37 (s, 4H), 7.24 – 7.19 (m, 1H), 6.89 (dd, *J* = 7.2, 1.6 Hz, 1H), 2.48 (s, 3H); **^{13}C NMR** (101 MHz, $CDCl_3$): δ 152.4, 150.0, 144.8, 142.7, 139.9, 136.3, 130.5, 130.3, 125.6, 125.3, 124.5, 124.4, 123.1, 122.9, 117.8, 112.8, 107.5, 21.6; ***m/z*** (ESI+) 311 ($[M+H]^+$, 100%); **HRMS** (ESI+) found 311.1289 ($[M+H]^+$), $C_{20}H_{15}N_4^+$ requires 311.1291.

2-(Pyridin-2-yl)-3-(*p*-tolyl)imidazo[1,2-*a*]pyridine-7-carboxamide (OSA_001015)

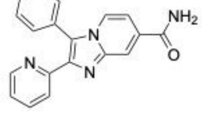

Compound **EGT 545-1** (10.0 mg, 32.2 μmol, 1.0 equiv.) was dissolved in 1-propanol (1.79 mL, 18 mM), KOH (3.98 mg, 70.9 μmol, 2.2 equiv.) was added and the mixture stirred at 110 °C for 12 h. The solvent was removed, the residue purified

by flash chromatography on silica (1-10% MeOH in CH_2Cl_2) to give *the title compound* as a yellow powder (4.90 mg, 46%); **^{1}H NMR** (400 MHz, $CDCl_3$): δ 8.61 (d, *J* = 4.8 Hz, 1H), 8.27 (s, 1H), 8.01 (d, *J* = 7.2 Hz, 1H), 7.83 – 7.55 (m, 2H), 7.46 – 7.31 (m, 5H), 7.18 (q, *J* = 4.6 Hz, 1H), 2.47 (s, 3H) (amide NH_2 signals not observed); **^{13}C NMR** (101 MHz, $CDCl_3$): δ 167.3, 152.1, 149.8, 143.4, 142.8, 139.8, 137.4, 136.5, 130.6, 130.4, 125.5, 124.8, 123.8, 123.1, 122.8, 117.0, 112.1, 21.7; ***m/z*** (ESI+) 329 ($[M+H]^+$, 100%); **HRMS** (ESI+) found 329.1398 ($[M+H]^+$), $C_{20}H_{17}N_4O^+$ requires 329.1397.

2-(Pyridin-2-yl)imidazo[1,2-*a*]pyrimidine (S34)

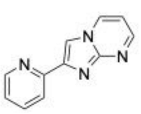

Prepared according to General Procedure A from: 2-Bromo-1-(pyridin-2-yl)ethan-1-one hydrobromide (1.00 g, 3.56 mmol) and 2-aminopyrimidine (508 mg, 5.34 mmol) to give *the title compound* as a reddish-brown powder (182 mg, 26%); **^{1}H NMR** (400 MHz, $CDCl_3$): δ 8.60 (ddd, *J* = 4.9, 1.8, 0.9 Hz, 1H), 8.55 (dd, *J* = 4.1, 2.0 Hz, 1H), 8.47 (dd, *J* = 6.8, 2.0 Hz, 1H), 8.36 (dt, *J* = 7.9, 1.1 Hz, 1H), 8.31 (s, 1H), 7.83 (td, *J* = 7.7, 1.8 Hz, 1H), 7.27 (ddd, *J* = 7.6, 4.9, 1.3 Hz, 1H), 6.87 (dd, *J* = 6.7, 4.1 Hz, 1H); **^{13}C NMR** (101 MHz, $CDCl_3$): δ 152.1, 150.6, 148.9, 148.7, 146.5, 137.6, 133.7, 123.4, 121.6, 109.5, 109.3; ***m/z*** (ESI+) 197 ($[M+H]^+$, 100%).

3-Bromo-2-(pyridin-2-yl)imidazo[1,2-*a*]pyrimidine (S35)

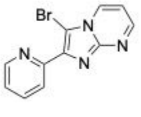

Prepared according to General Procedure C from: **S34** (125 mg, 0.64 mmol) to give *the title compound* as a brown powder (84.5 mg, 48%); **^{1}H NMR** (400 MHz, $CDCl_3$): δ 8.76 (d, *J* = 4.5 Hz, 1H), 8.63 (br s, 1H), 8.55 (d, *J* = 6.8 Hz, 1H), 8.38 (d, *J* = 8.0 Hz, 1H), 7.83 (t, *J* = 7.7 Hz, 1H), 7.40 – 7.27 (m, 1H), 7.03 (dd, *J* = 7.1, 4.2 Hz, 1H); ***m/z*** (ESI+) 275 ($[M+H]^+$, 100%).

3-(Benzofuran-5-yl)-2-(pyridin-2-yl)imidazo[1,2-*a*]pyrimidine (OSA_000863)

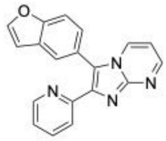

Prepared according to General Procedure D with $PdCl_2(dppf) \cdot CH_2Cl_2$ (8.91 mg, 0.01 mmol, 0.12 equiv.) instead from: **S35** (25.0 mg, 0.09 mmol) and **S4** (28.9 mg, 0.12 mmol); purified by methods 1, then 2, to give *the title compound* as a red powder (3.80 mg, 13%); **^{1}H NMR** (400 MHz, $CDCl_3$): δ 9.67 (dd, *J* = 7.0, 2.0 Hz, 1H), 9.14 – 8.75 (m, 1H), 8.66 (dd, *J* = 4.2, 2.0 Hz, 1H), 8.03 (d, *J* = 1.7 Hz, 1H), 7.66 (d, *J* = 2.2 Hz, 1H), 7.63 (dd, *J* = 8.6, 1.8 Hz, 1H), 7.57 (td, *J* = 7.8, 1.9 Hz, 1H), 7.51 (d, *J* = 8.6 Hz, 1H), 7.34 (d, *J* = 8.1 Hz, 1H), 7.27 – 7.19 (m, 1H), 6.97 (dd, *J* = 7.0, 4.1 Hz, 1H), 6.87 – 6.74 (m, 1H); **^{13}C NMR** (101 MHz, $CDCl_3$): δ 155.4, 151.1, 150.1, 149.5, 148.6, 147.3, 145.8, 136.7, 134.3, 128.7, 128.0, 126.1, 125.2, 122.7, 122.2, 118.0, 111.7, 109.3, 107.1; ***m/z*** (ESI+) 313 ($[M+H]^+$, 100%); **HRMS** (ESI+) found 313.1081 ($[M+H]^+$), $C_{19}H_{13}N_4O^+$ requires 313.1084.

3-(Benzo[*d*][1,3]dioxol-5-yl)-2-(pyridin-2-yl)imidazo[1,2-*a*]pyrimidine (OSA_000815)

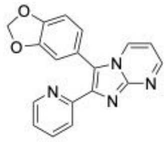

Prepared according to General Procedure D from: **S35** (75.0 mg, 0.27 mmol) and benzo[*d*][1,3]dioxol-5-ylboronic acid (59.0 mg, 0.35 mmol); purified by methods 1 (40-85% EtOAc in hexane), then 3, to give *the title compound* as a yellow solid (20.1 mg, 23%); **^{1}H NMR** (400 MHz, $CDCl_3$): δ 9.57 (dd, *J* = 7.0, 2.1 Hz, 1H), 8.77 – 8.71 (m, 1H), 8.62 (dd, *J* = 4.2, 2.0 Hz, 1H), 7.63 (td, *J* = 7.8, 1.9 Hz, 1H), 7.42 (d, *J* = 8.0 Hz, 1H), 7.28 – 7.18 (m, 3H), 6.95 (dd, *J* = 7.0, 4.1 Hz, 1H), 6.82 (d, *J* = 8.0 Hz, 1H), 5.99 (s, 2H); **^{13}C NMR** (101 MHz, $CDCl_3$): δ 151.3, 149.8, 149.5, 148.4, 148.2, 148.0, 146.0, 136.8, 134.1, 127.3, 125.3, 123.7, 122.4, 117.7, 109.8, 109.4, 108.7, 101.4; ***m/z*** (ESI+) 317 ($[M+H]^+$, 100%); **HRMS** (ESI+) found 317.1035 ($[M+H]^+$), $C_{18}H_{13}N_4O_2^+$ requires 317.1033.

N-(4-Fluorophenyl)-N-isobutyl-2-(thiazol-2-yl)imidazo[1,2-a]pyridin-3-amine (OSA_001010)

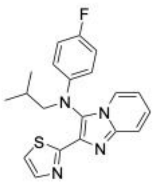

To a mixture of **DMK 195-1; OSA_001008** (150 mg, 0.48 mmol, 1.00 equiv.) and Cs_2CO_3 (190 mg, 0.58 mmol, 1.21 equiv.) in DMF (3.2 mL, 0.15 M) was added 1-iodo-2-methylpropane (83.0 µL, 0.72 mmol, 1.49 equiv.). The reaction mixture was stirred overnight at rt. The reaction temperature increased to 50 ºC and stirred for another 24 h. The reaction temperature was further increased to 100 ºC and stirred for another 24 h. Additional 1-iodo-2-methylpropane (83.0 µL, 0.72 mmol, 1.49 equiv.) was added and the reaction was stirred at 100 ºC for another 3 days. Additional Cs_2CO_3 (190 mg, 0.58 mmol, 1.21 equiv.) was added and the temperature lowered to 50 ºC for 24 h. The reaction was cooled to rt, diluted with EtOAc and the organic layer was washed with H_2O (5 ×), brine, dried (Na_2SO_4), filtered and concentrated under reduced pressure to give the crude product that was purified by flash chromatography on silica (20-100% EtOAc in hexanes), then triturated with MeOH to give *the title compound* as a bright yellow powder (23.9 mg, 14%); **^{1}H NMR** (400 MHz, $CDCl_3$): δ 7.86 (d, J = 3.2 Hz, 1H), 7.76 (d, J = 6.9 Hz, 1H), 7.68 (d, J = 9.1 Hz, 1H), 7.33 (d, J = 3.2 Hz, 1H), 7.32 – 7.23 (m, 1H), 6.92 – 6.83 (m, 2H), 6.82 (td, J = 6.8, 1.1 Hz, 1H), 6.57 – 6.47 (m, 2H), 3.73 (d, J = 6.8 Hz, 2H), 1.90 (hept, J = 6.7 Hz, 1H), 0.99 – 0.76 (m, 6H); **^{13}C NMR** (126 MHz, $CDCl_3$): δ 161.9, 156.9 (d, J = 237.6 Hz), 144.4, 143.3, 142.3, 133.2, 126.1, 125.3, 123.1, 119.3, 118.3, 116.1 (d, J = 22.6 Hz), 115.2 (d, J = 7.4 Hz), 113.2, 61.2, 28.7, 21.0; ***m/z*** (ESI+) 367 ([M+H]$^+$, 100%); **HRMS** (ESI+) found 367.1397 ([M+H]$^+$), $C_{20}H_{20}FN_4S^+$ requires 367.1387.

6-Methyl-2-phenylimidazo[1,2-a]pyridine (OSA_001026)

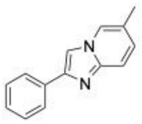

Prepared according to General Procedure B from: 5-methylpyridin-2-amine (1.00 g, 9.25 mmol) and 2-bromoacetophenone (1.93 g, 9.71 mmol); purified by flash

chromatography on silica (40% EtOAc in cyclohexane) to give *the title compound* as a yellow solid (1.80 g, 95%); **m.p.** 168 – 170 °C; **^{1}H NMR** (300 MHz, $CDCl_3$): δ 7.97 – 7.91 (m, 2H), 7.87 (q, *J* = 1.2 Hz, 1H), 7.75 (s, 1H), 7.52 (d, *J* = 9.2 Hz, 1H), 7.46 – 7.38 (m, 2H), 7.36 – 7.27 (m, 1H), 7.00 (dd, *J* = 9.2, 1.6 Hz, 1H), 2.30 (d, *J* = 1.2 Hz, 3H); **^{13}C NMR** (75 MHz, $CDCl_3$): δ 145.5, 144.8, 133.9, 128.7, 127.9, 127.9, 126.0, 123.4, 122.1, 116.87, 107.9, 18.1; ***m/z*** (ESI+) 209 ($[M+H]^+$, 100%); **HRMS** (ESI+) found 209.1072 ($[M+H]^+$), $C_{14}H_{13}N_2^+$ requires 209.1073.

N-((6-Methyl-2-phenylimidazo[1,2-*a*]pyridin-3-yl)methyl)-3-(trifluoromethyl)aniline (OSA_001028)

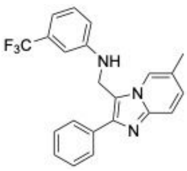

Prepared according to General Procedure G from: **OSA_001026** (100 mg, 0.48 mmol) and 3-(trifluoromethyl)aniline (92.8 mg, 0.58 mmol); purified by flash chromatograph on silica (50% EtOAc in hexanes) to give *the title compound* as a brown solid (99.5 mg, 45%); **m.p.** 213 – 218 °C; **^{1}H NMR** (300 MHz, acetic acid-d_4): δ 8.61 (s, 1H), 7.94 (d, *J* = 9.2 Hz, 1H), 7.81 – 7.71 (m, 3H), 7.63 – 7.50 (m, 3H), 7.21 (t, *J* = 7.8 Hz, 1H), 6.92 (d, *J* = 7.6 Hz, 1H), 6.86 – 6.76 (m, *J* = 8.6 Hz, 2H), 4.84 (s, 2H), 2.45 (s, 3H); **^{13}C NMR** (75 MHz, acetic acid-d_4): δ 149.6, 140.6, 137.7, 136.7, 133.1, 132.7, 132.3, 131.5, 131.2, 130.6, 129.7, 128.3, 126.4, 124.4, 120.6, 118.3, 116.0, 113.9, 110.5, 37.9, 18.9; ***m/z*** (ESI+) 382 ($[M+H]^+$, 100%); **HRMS** (ESI+) found 382.1525 ($[M+H]^+$), $C_{22}H_{19}F_3N_3^+$ requires 382.1526.

4-Fluoro-*N*-((6-methyl-2-phenylimidazo[1,2-*a*]pyridin-3-yl)methyl)aniline (OSA_001027)

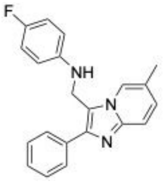

Prepared according to General Procedure G from: **RDGS 98-1; OSA_001026** (100 mg, 0.48 mmol) and 4-fluoroaniline (64.0 mg, 0.58 mmol); purified by flash chromatograph on silica (40% EtOAc in hexanes) to give *the title compound* as a yellow solid (60.0 mg, 38%); **^{1}H NMR** (300 MHz, DMSO-d_6): δ 8.16 (s, 1H), 7.82 – 7.75 (m, 2H), 7.55 (d, J = 9.2 Hz, 1H), 7.51 – 7.41 (m, 2H), 7.40 – 7.32 (m, H), 7.17 (dd, J = 9.2, 1.6 Hz, 1H), 6.95 (dd, J = 9.9, 7.9 Hz, 2H), 6.67 (dd, J = 9.0, 4.5 Hz, 2H), 6.07 (t, J = 4.8 Hz, 1H), 4.55 (d, J = 4.8 Hz, 2H), 2.31 (s, 3H); **^{13}C NMR** (75 MHz, DMSO-d_6): δ 154.7 (d, J = 231.5 Hz), 145.3 (d, J = 1.2 Hz), 143.2, 143.1, 134.5, 128.6, 127.94, 127.85, 127.6, 122.4, 121.4, 117.1, 116.2, 115.3 (d, J = 21.9 Hz), 113.3 (d, J = 7.3 Hz), 37.5, 17.8; ***m/z*** (ESI+) 332 ([M+H]$^+$, 100%); **HRMS** (ESI+) found 332.1547 ([M+H]$^+$), $C_{21}H_{19}FN_3^+$ requires 332.1558.

***N*-Butyl-4-fluoroaniline (S36)**

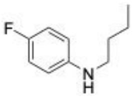

A solution of 4-fluoroaniline (1.00 g, 4.80 mmol, 1.00 equiv.), 1-iodobutane (0.97 g, 5.28 mmol, 1.10 equiv.) and K_2CO_3 (80.0 mg, 0.57 mmol, 0.12 equiv.) in NMP (20 mL) was stirred at 100 °C for 3 days. The reaction was cooled to rt and the solvent removed under reduced pressure. The residue was partitioned between EtOAc and H_2O. The aqueous layer was separated and extracted with EtOAc (3 ×) and the combined organic layers dried ($MgSO_4$), filtered and concentrated under reduced pressure to give the crude product which was purified by flash chromatography on silica (1% EtOAc in hexanes) to give *the title compound* as a brown oil (626 mg, 78%); **^{1}H NMR** (300 MHz, DMSO-d_6): δ 6.94 – 6.83 (m, 2H), 6.56 – 6.46 (m, 2H), 5.40 (br s, 1H), 2.94 (t, J = 7.0 Hz, 1H), 1.57 – 1.45 (m, 2H), 1.44 – 1.30 (m, 2H), 0.90 (t, J = 7.2 Hz, 3H).

N-Butyl-N-((6-methyl-2-phenylimidazo[1,2-*a*]pyridin-3-yl)methyl)-3-(trifluoromethyl)aniline (OSA_001029)

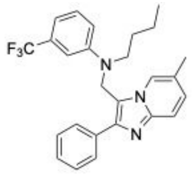

Prepared according to General Procedure G from: **OSA_001026** (124 mg, 0.32 mmol) and **S36** (88.0 mg, 0.38 mmol); purified by flash chromatograph on silica (1% MeOH in CH_2Cl_2) to give *the title compound* as a yellow solid (56.0 mg, 40%); **^{1}H NMR** (300 MHz, DMSO-d_6): δ 8.87 (s, 1H), 8.36 (d, J = 9.3 Hz, 1H), 8.04 (dd, J = 9.3, 1.4 Hz, 1H), 7.75 – 7.63 (m, 4H), 7.22 (t, J = 7.9 Hz, 1H), 6.86(d, J = 7.7 Hz, 1H), 6.76 – 6.67 (m, 2H), 6.67 – 6.59 (m, 1H), 4.57 (d, J = 5.1 Hz, 2H), 4.37 (t, J = 7.2 Hz, 2H), 1.48 (p, J = 7.5 Hz, 2H), 1.22 (s, 3H), 1.08 (p, J = 7.4 Hz, 2H), 0.66 (t, J = 7.3 Hz, 3H); **^{13}C NMR** (75 MHz, DMSO-d_6): δ 147.9, 137.6, 136.5, 135.0, 131.0, 130.5, 129.9, 129.4, 127.7, 125.4, 124.7, 122.5, 120.6, 116.3, 113.0, 110.9, 107.8, 44.3, 35.5, 30.7, 18.8, 17.5, 13.2; ***m/z*** (ESI+) 438 ([M+H]$^+$, 100%); **HRMS** (ESI+) found 438.2123 ([M+H]$^+$), $C_{26}H_{27}F_3N_3^+$ requires 438.2152.

7-Methyl-2-(pyridin-2-yl)imidazo[1,2-*a*]pyridine (OSA_001030)

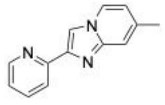

Prepared according to General Procedure B from: 4-methylpyridin-2-amine (1.00 g, 9.25 mmol) and 2-bromo-1-(pyridin-2-yl)ethan-1-one (1.94 g, 9.71 mmol); purified by flash chromatography on silica (90% EtOAc in hexanes) to give *the title compound* as a white solid (1.20 g, 60% yield); **^{1}H NMR** (300 MHz, DMSO-d_6): δ 8.58 (ddd, J = 4.8, 1.8, 0.9 Hz, 1H), 8.46 (d, J = 6.9 Hz, 1H), 8.38 (s, 1H), 8.08 (dt, J = 7.9, 1.2 Hz, 1H), 7.86 (td, J = 7.7, 1.8 Hz, 1H), 7.37 (s, 1H), 7.30 (ddd, J = 7.5, 4.8, 1.2 Hz, 1H), 6.77 (dd, J = 6.9, 1.6 Hz, 1H), 2.35 (s, 3H); **^{13}C NMR** (75 MHz, DMSO-d_6): δ 153.0, 149.4, 145.2, 144.4, 137.0, 135.8, 126.5, 122.7, 119.7, 115.14, 115.11, 110.8, 20.9; ***m/z*** (ESI+) 210 ([M+H]$^+$, 100%); **HRMS** (ESI+) found 210.1011 ([M+H]$^+$), $C_{13}H_{12}N_3^+$ requires 210.1026.

4-Fluoro-*N*-((7-methyl-2-(pyridin-2-yl)imidazo[1,2-*a*]pyridin-3-yl)methyl)aniline (OSA_001031)

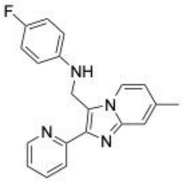

Prepared according to General Procedure G from: **OSA_001030** (100 mg, 0.48 mmol) and 4-fluoroaniline (63.7 mg, 0.57 mmol); purified by flash chromatography on silica (80% EtOAc in hexanes) to give *the title compound* as a grey solid (60.0 mg, 35%); **^{1}H NMR** (300 MHz, DMSO-d_6): δ 8.66 (dd, J = 5.0, 1.9 Hz, 1H), 8.39 (d, J = 7.0 Hz, 1H), 8.21 (d, J = 8.0 Hz, 1H), 7.90 (td, J = 7.7, 1.9 Hz, 1H), 7.38 (s, 1H), 7.33 (ddd, J = 7.6, 4.8, 1.2 Hz, 1H), 6.87 – 6.76 (m, 3H), 6.74 – 6.64 (m, 2H), 6.10 (s, 1H), 5.16 (s, 2H), 2.36 (s, 3H); **^{13}C NMR** (75 MHz, DMSO-d_6): δ 154.4 (d, J = 231.3 Hz), 154.5, 149.0, 145.2 (d, J = 1.3 Hz), 144.3, 140.3, 137.0, 135.8, 124.8, 122.3, 121.8, 120.1, 115.3, 115.2, 115.0 (d, J = 7.0 Hz), 113.3 (d, J = 7.3 Hz); **HRMS** (ESI+) found 355.1296 ([M+Na]$^+$), $C_{20}H_{17}FN_4Na^+$ requires 355.1329.

***N*-Butyl-4-fluoro-*N*-((7-methyl-2-(pyridin-2-yl)imidazo[1,2-*a*]pyridin-3-yl)methyl)aniline (OSA_001032)**

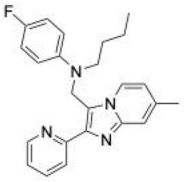

Prepared according to General Procedure G from: **OSA_001030** (100 mg, 0.48 mmol) and **S36** (95.9 mg, 0.57 mmol); purified by flash chromatograph on silica (40% EtOAc in hexanes) to give *the title compound* as a brown solid (130 mg, 70%); **^{1}H NMR** (300 MHz, $CDCl_3$): δ 8.71 – 8.54 (m, 1H), 8.25 (dd, J = 7.8, 1.4 Hz, 1H), 7.95 (d, J = 7.0 Hz, 1H), 7.78 (td, J = 7.8, 1.9 Hz, 1H), 7.40 (s, 1H), 7.24 – 7.15 (m, 1H), 7.01 – 6.82 (m, 4H), 6.59 (dd, J = 7.1, 1.7 Hz, 1H), 5.25 (s, 2H), 3.12 – 3.02 (m, 2H), 2.38 (s, 3H), 1.41 – 1.27 (m, 2H), 1.03 (h, J = 7.3 Hz, 2H), 0.67 (t, J = 7.3 Hz, 3H); **^{13}C NMR** (75 MHz, $CDCl_3$): δ 156.5 (d, J = 237.5 Hz), 154.3, 148.7, 146.2 (d, J = 2.1 Hz), 145.2, 141.9, 136.8, 136.2, 124.5, 122.2 (d, J = 19.4 Hz), 119.3, 117.8 (d, J = 7.4 Hz), 115.70, 115.68, 115.4,

115.2, 50.9, 44.4, 27.9, 21.4, 20.1, 13.7; ***m/z*** (ESI+) 389 ($[M+H]^+$, 100%); **HRMS** (ESI+) found 389.2120 ($[M+H]^+$), $C_{24}H_{26}FN_4^+$ requires 389.2136.

***N*-isobutyl-3-(trifluoromethyl)aniline (S37)**

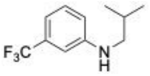

A solution of 3-(trifluoromethyl)aniline (1.00 g, 6.20 mmol, 1.00 equiv.), 1-iodo-2-methylpropane (1.25 g, 6.82 mmol, 1.10 equiv.) and K_2CO_3 (103 mg, 0.74 mmol, 0.12 equiv.) in NMP (20 mL) was stirred at 100 °C for 3 days. The reaction was cooled to rt and the solvent removed under reduced pressure. The residue was partitioned between EtOAc and H_2O. The aqueous layer was separated and extracted with EtOAc (3 ×) and the combined organic layers dried ($MgSO_4$), filtered and concentrated under reduced pressure to give the crude product which was purified by flash chromatography on silica (1% EtOAc in hexanes) to give *the title compound* as a brown oil (1.10 g, 82%); **^{1}H NMR** (300 MHz, $CDCl_3$): δ 7.24 – 7.12 (m, 1H), 6.90 – 6.78 (m, 1H), 6.72 (d, *J* = 2.4 Hz, 1H), 6.66 (dd, *J* = 8.2, 2.4 Hz, 1H), 4.03 (br, 1H), 2.88 (dd, *J* = 6.8, 0.9 Hz, 2H), 1.92 – 1.64 (m, 1H), 0.92 (dd, *J* = 6.7, 0.9 Hz, 6H).

***N*-Isobutyl-*N*-((7-methyl-2-(pyridin-2-yl)imidazo[1,2-*a*]pyridin-3-yl)methyl)-3-(trifluoromethyl)aniline (OSA_001033)**

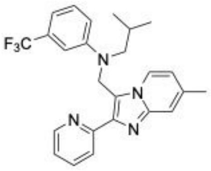

Prepared according to General Procedure G from: **OSA_001030** (50.0 mg, 0.23 mmol) and 4-fluoro-*N*-isobutylaniline (60.0 mg, 0.27 mmol, synthesized according to the same procedure for **S36**); purified flash chromatograph on silica (40% EtOAc in cyclohexane) to give *the title compound* as a white power (39.0 mg, 39%); **^{1}H NMR** (300 MHz, acetone-d_6): δ 8.68 (dq, *J* = 4.8, 1.9, 1.2 Hz, 1H), 8.36 (dt, *J* = 8.0, 1.0 Hz, 1H), 7.99 (dd, *J* = 7.0, 0.9 Hz, 1H), 7.90 (td, *J* = 7.8, 1.8 Hz, 1H), 7.38 (s, 1H), 7.35 – 7.27 (m, 4H), 6.91 – 6.86 (m, 1H), 6.74 (dd, *J* = 7.1, 1.8 Hz, 1H), 5.67 (s, 2H), 3.40 (d, *J* = 7.3 Hz, 2H), 2.36 (d, *J* = 1.1 Hz, 3H), 2.07 – 2.02 (m, 1H), 0.82 (s, 3H), 0.80 (s,

3H); ^{13}**C NMR** (75 MHz, acetone-d_6): δ 156.0, 150.2, 149.6, 146.0, 142.7, 137.5, 136.6, 132.6 – 130.7 (m), 130.4, 125.4, 123.0, 119.8, 119.2, 116.6, 115.9, 114.0 (d, J = 3.8 Hz), 112.0, 59.2, 46.4, 27.5, 21.1, 20.5; ***m/z*** (ESI+) 439 ([M+H]$^+$, 100%); **HRMS** (ESI+) found 439.2086 ([M+H]$^+$), $C_{25}H_{26}F_3N_4^+$ requires 439.2104.

1-(Benzofuran-5-yl)-2-(pyridin-2-yl)-1*H*-benzo[*d*]imidazole (OSA_000991)

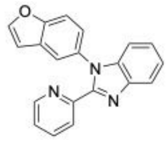

Prepared according to General Procedure H from: 2-(pyridin-2-yl)-1*H*-benzo[*d*]imidazole (100 mg, 0.51 mmol) and 5-bromobenzofuran (202 mg, 1.02 mmol); purified by flash chromatograph on silica (1-10% MeOH in CH_2Cl_2), followed by reversed-phase flash chromatograph on silica (5-100% MeOH in H_2O) to give *the title compound* as a brown powder (38.3 mg, 24%); ^{1}**H NMR** (500 MHz, $CDCl_3$): δ 8.37 (ddd, J = 4.9, 1.8, 1.0 Hz, 1H), 8.04 (dt, J = 8.0, 1.0 Hz, 1H), 7.92 (dt, J = 8.1, 1.0 Hz, 1H), 7.74 (d, J = 2.2 Hz, 1H), 7.72 (td, J = 7.7, 1.8 Hz, 1H), 7.60 (d, J = 2.1 Hz, 1H), 7.58 (d, J = 8.8 Hz, 1H), 7.36 (ddd, J = 8.1, 7.0, 1.2 Hz, 1H), 7.29 (ddd, J = 8.2, 7.1, 1.1 Hz, 1H), 7.24 – 7.14 (m, 3H), 6.82 (dd, J = 2.2, 1.0 Hz, 1H); ^{13}**C NMR** (101 MHz, $CDCl_3$): δ 154.3, 151.1, 149.6, 149.2, 146.5, 142.8, 138.3, 136.5, 132.9, 128.3, 124.7, 124.1, 124.0, 123.7, 123.2, 120.4, 120.1, 112.1, 111.1, 107.1; ***m/z*** (ESI+) 312 ([M+H]$^+$, 100%); **HRMS** (ESI+) found 312.1132 ([M+H]$^+$), $C_{20}H_{14}N_3O^+$ requires 312.1131.

1-Phenyl-2-(pyridin-2-yl)-1*H*-benzo[*d*]imidazole (OSA_000992)

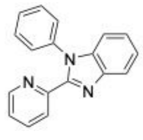

Prepared according to General Procedure H from: 2-(pyridin-2-yl)-1*H*-benzo[*d*]imidazole (100 mg, 0.51 mmol) and bromobenzene (169 µL, 1.64 mmol); purified by flash chromatograph on silica (1-10% MeOH in CH_2Cl_2), followed by reversed-phase flash chromatograph on silica (5-100% MeOH in H_2O) to give *the title compound* as a white powder (23.1 mg, 17%); ^{1}**H NMR** (500 MHz, $CDCl_3$): δ 8.41 (ddd, J = 4.9, 1.8, 0.9 Hz,

1H), 8.05 (dt, J = 8.0, 1.1 Hz, 1H), 7.91 (dt, J = 8.0, 0.9 Hz, 1H), 7.74 (td, J = 7.8, 1.8 Hz, 1H), 7.59 – 7.41 (m, 3H), 7.38 – 7.28 (m, 4H), 7.25 – 7.20 (m, 2H); **^{13}C NMR** (101 MHz, $CDCl_3$): δ 150.8, 149.6, 149.2, 142.9, 137.83, 137.75, 136.5, 129.4, 128.2, 127.5, 124.7, 124.0, 123.8, 123.3, 120.4, 111.0; ***m/z*** (ESI+) 272 ([M+H]$^+$, 100%); **HRMS** (ESI+) found 272.1182 ([M+H]$^+$), $C_{18}H_{14}N_3^+$ requires 272.1182.

1-Benzyl-2-(pyridin-2-yl)-1*H*-benzo[*d*]imidazole (OSA_000993)

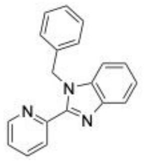

Prepared according to General Procedure H from: 2-(pyridin-2-yl)-1*H*-benzo[*d*]imidazole (100 mg, 0.51 mmol) and benzyl bromide (97.3 µL, 0.82 mmol); purified by flash chromatograph on silica (1-10% MeOH in CH_2Cl_2), followed by reversed-phase flash chromatograph on silica (5-100% MeOH in H_2O) to give *the title compound* as a brown powder (76.6 mg, 52%); **^{1}H NMR** (500 MHz, $CDCl_3$): δ 8.63 (ddd, J = 4.9, 1.9, 0.9 Hz, 1H), 8.43 (dt, J = 7.9, 1.0 Hz, 1H), 7.93 – 7.73 (m, 2H), 7.39 – 7.27 (m, 4H), 7.26 – 7.19 (m, 3H), 7.19 – 7.12 (m, 2H), 6.20 (s, 2H); **^{13}C NMR** (101 MHz, $CDCl_3$): δ 150.7, 150.1, 148.7, 142.9, 137.6, 137.0, 136.9, 128.7, 127.5, 126.9, 124.8, 124.0, 123.7, 122.9, 120.3, 110.9, 49.0; ***m/z*** (ESI+) 286 ([M+H]$^+$, 100%); **HRMS** (ESI+) found 286.1338 ([M+H]$^+$), $C_{19}H_{16}N_3^+$ requires 286.1339.

1-(4-(Piperidin-1-yl)phenyl)-2-(pyridin-2-yl)-1*H*-benzo[d]imidazole (OSA_000994)

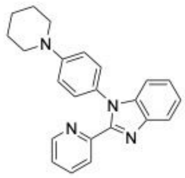

Prepared according to General Procedure H from: 2-(pyridin-2-yl)-1*H*-benzo[*d*]imidazole (100 mg, 0.51 mmol) and 1-(4-bromophenyl)piperidine (197 mg, 0.82 mmol); purified by flash chromatograph on silica (1-10% MeOH in CH_2Cl_2), followed by reversed-phase flash chromatograph on silica (5-100% MeOH in H_2O) to give *the title compound* as a yellow powder (110 mg, 61%); **^{1}H NMR** (500 MHz, $CDCl_3$): δ 8.51 (ddd, J = 4.8, 1.8, 0.9 Hz, 1H), 7.89 (ddt, J = 7.5, 5.5, 1.1 Hz, 2H), 7.69 (td, J = 7.8, 1.8

Hz, 1H), 7.33 (ddd, *J* = 8.1, 6.4, 1.9 Hz, 1H), 7.30 – 7.24 (m, 2H), 7.26 – 7.19 (m, 1H), 7.18 (d, *J* = 8.8 Hz, 2H), 6.98 (d, *J* = 9.0 Hz, 2H), 3.47 – 2.92 (m, 4H), 1.85 – 1.69 (m, 4H), 1.65 – 1.59 (m, 2H); **^{13}C NMR** (101 MHz, $CDCl_3$): δ 151.7, 151.0, 149.7, 149.4, 142.8, 138.1, 136.3, 128.3, 128.0, 124.7, 123.7, 123.6, 123.0, 120.3, 116.4, 111.2, 50.3, 25.9, 24.3; ***m/z*** (ESI+) 355 ([M+H]$^+$, 100%); **HRMS** (ESI+) found 355.1917 ([M+H]$^+$), $C_{23}H_{23}N_4^+$ requires 355.1917.

4-(4-(2-(Pyridin-2-yl)-1*H*-benzo[*d*]imidazol-1-yl)phenyl)morpholine (OSA_000995)

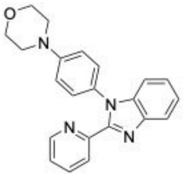

Prepared according to General Procedure H from: 2-(pyridin-2-yl)-1*H*-benzo[*d*]imidazole (100 mg, 0.51 mmol) and 4-(4-bromophenyl)morpholine (198 mg, 0.82 mmol); purified by flash chromatograph on silica (1-10% MeOH in CH_2Cl_2), followed by reversed-phase flash chromatograph on silica (5-100% MeOH in H_2O) to give *the title compound* as an off-white powder (109 mg, 60%); **^{1}H NMR** (500 MHz, $CDCl_3$): δ 8.48 (ddd, *J* = 4.8, 1.8, 0.9 Hz, 1H), 7.97 (dt, *J* = 7.9, 1.1 Hz, 1H), 7.90 (dt, *J* = 8.1, 0.9 Hz, 1H), 7.72 (td, *J* = 7.8, 1.8 Hz, 1H), 7.34 (ddd, *J* = 8.1, 6.9, 1.4 Hz, 1H), 7.30 – 7.26 (m, 1H), 7.25 – 7.21 (m, 2H), 7.23 (d, *J* = 8.9 Hz, 2H), 6.97 (d, *J* = 9.0 Hz, 2H), 4.14 – 3.76 (m, 4H), 3.60 – 3.09 (m, 4H); **^{13}C NMR** (101 MHz, $CDCl_3$): δ 150.91, 150.90, 149.6, 149.3, 142.7, 138.0, 136.4, 129.4, 128.2, 124.7, 123.9, 123.7, 123.1, 120.3, 115.8, 111.1, 67.0, 49.0; ***m/z*** (ESI+) 357 ([M+H]$^+$, 100%); **HRMS** (ESI+) found 357.1710 ([M+H]$^+$), $C_{22}H_{21}N_4O^+$ requires 357.1710.

2-(4-(2-(Pyridin-2-yl)-1*H*-benzo[*d*]imidazol-1-yl)phenyl)acetonitrile (OSA_000996)

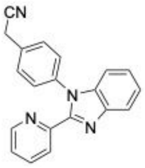

Prepared according to General Procedure H from: 2-(pyridin-2-yl)-1*H*-benzo[*d*]imidazole (100 mg, 0.51 mmol) and 2-(4-bromophenyl)acetonitrile (161 mg, 0.82 mmol); purified by flash chromatograph on silica (1-10% MeOH in CH_2Cl_2), followed by reversed-phase flash chromatograph on silica (5-100% MeOH in H_2O)

to give *the title compound* as a pale purple powder (18.3 mg, 12%); **^{1}H NMR** (500 MHz, $CDCl_3$): δ 8.37 (dt, *J* = 4.9, 1.2 Hz, 1H), 8.17 (d, *J* = 7.9 Hz, 1H), 7.91 (d, *J* = 7.9 Hz, 1H), 7.78 (td, *J* = 7.8, 1.8 Hz, 1H), 7.46 (d, *J* = 8.3 Hz, 2H), 7.40 – 7.34 (m, 1H), 7.36 (d, *J* = 8.2 Hz, 2H), 7.31 (ddd, *J* = 8.3, 7.2, 1.3 Hz, 1H), 7.25 – 7.19 (m, 2H), 3.87 (s, 2H); **^{13}C NMR** (101 MHz, $CDCl_3$): δ 150.6, 149.3, 149.0, 142.8, 137.9, 137.6, 136.7, 129.9, 129.0, 128.3, 124.8, 124.3, 124.0, 123.5, 120.4, 117.6, 110.8, 23.6; ***m/z*** (ESI+) 311 ($[M+H]^+$, 100%); **HRMS** (ESI+) found 311.1288 ($[M+H]^+$), $C_{20}H_{15}N_4^+$ requires 311.1291.

4-(2-(Pyridin-2-yl)-1*H*-benzo[*d*]imidazol-1-yl)benzonitrile (OSA_000990)

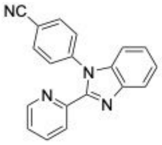

Prepared according to General Procedure H from: 2-(pyridin-2-yl)-1*H*-benzo[*d*]imidazole (100 mg, 0.51 mmol) and 4-bromobenzonitrile (0.1 mL, 0.81 mmol); purified by flash chromatograph on silica (1-10% MeOH in CH_2Cl_2), followed by reversed-phase flash chromatograph on silica (5-100% MeOH in H_2O) to give *the title compound* as a light yellow powder (20.2 mg, 13%); **^{1}H NMR** (500 MHz, CD_3OD): δ 8.35 (d, *J* = 4.7 Hz, 1H), 8.15 (d, *J* = 7.9 Hz, 1H), 7.96 (td, *J* = 7.8, 1.7 Hz, 1H), 7.90 (d, *J* = 8.5 Hz, 2H), 7.84 (d, *J* = 8.1 Hz, 1H), 7.56 (d, *J* = 8.5 Hz, 2H), 7.45 – 7.34 (m, 3H), 7.32 (d, *J* = 7.3 Hz, 1H); **^{13}C NMR** (126 MHz, CD_3OD): δ 151.8, 150.1, 149.6, 143.4, 142.8, 138.6, 138.0, 134.7, 129.7, 126.1, 126.04, 125.95, 125.2, 120.6, 119.1, 113.3, 111.9; ***m/z*** (ESI+) 297 ($[M+H]^+$, 100%); **HRMS** (ESI+) found 297.1141 ($[M+H]^+$), $C_{19}H_{15}N_4^+$ requires 299.1291.

4-(2-(Pyridin-2-yl)-1*H*-benzo[*d*]imidazol-1-yl)benzamide (OSA_000989)

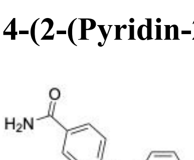

Isolated from the same reaction as for **OSA_000990** to give *the title compound* as an off-white powder (37.8 mg, 24%); **^{1}H NMR** (500 MHz, DMSO-d_6): δ 8.33 (ddd, *J* = 4.9, 1.8, 0.9 Hz, 1H), 8.22 (dt, *J* = 7.9, 1.1 Hz, 1H), 8.12 (br s, 1H), 7.99 (d, *J* = 8.6 Hz, 2H), 8.03 – 7.93 (m, 1H), 7.88 – 7.82 (m, 1H), 7.51 (br s, 1H), 7.46

(d, J = 8.5 Hz, 2H), 7.40 (ddd, J = 7.6, 4.8, 1.2 Hz, 1H), 7.37 – 7.29 (m, 2H), 7.28 – 7.21 (m, 1H); **^{13}C NMR** (126 MHz, DMSO-d_6): δ 151.7, 150.1, 149.6, 143.3, 142.8, 138.6, 137.9, 134.6, 129.7, 126.1, 126.0, 125.9, 125.1, 120.6, 119.1, 113.3, 111.9; ***m/z*** (ESI+) 315 ([M+H]$^+$, 100%); **HRMS** (ESI+) found 315.1248 ([M+H]$^+$), $C_{19}H_{15}N_4O^+$ requires 315.1240.

7-Methoxy-3-phenyl-2-(pyridin-2-yl)imidazo[1,2-*a*]pyridine (OSA_000860)

A vial was charged with 3-bromo-7-methoxy-2-(pyridin-2-yl)imidazo[1,2-*a*]pyridine (100 mg,

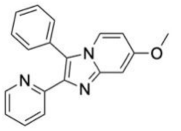

0.33 mmol), phenylboronic acid (60.1 mg, 0.49 mmol), K_2CO_3 (90.9 mg, 0.66 mmol), $Pd(OAc)_2$ (7.38 mg, 10% mol) and Xantphos (19.0 mg, 32.9 µmol), then backfilled with N_2 three times. A 3:1 mixture of 1,4-dioxane/H_2O (2.5 mL) was added and the reaction was degassed for 5 min then heated at 100 °C for 2 days. The crude product was directly purified by reversed-phase flash chromatography on silica (5-55% MeOH in H_2O) to give *the title compound* as a white solid (22.0 mg, 22%); **^{1}H NMR** (500 MHz, CD_3OD): δ 8.51 – 8.45 (m, 1H), 8.00 (d, J = 7.6 Hz, 1H), 7.74 (td, J = 7.8, 1.8 Hz, 1H), 7.58 (dt, J = 7.9, 1.1 Hz, 1H), 7.55 – 7.39 (m, 5H), 7.28 (ddd, J = 7.6, 5.0, 1.2 Hz, 1H), 6.97 (d, J = 2.4 Hz, 1H), 6.66 (dd, J = 7.6, 2.5 Hz, 1H), 3.93 (s, 3H); ***m/z*** (ESI+) 302 ([M+H]$^+$, 100%).

3-(4-Fluorophenyl)-7-methoxy-2-(pyridin-2-yl)imidazo[1,2-*a*]pyridine (OSA_000858)

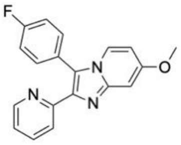

A vial was charged with 3-bromo-7-methoxy-2-(pyridin-2-yl)imidazo[1,2-*a*]pyridine (40.0 mg, 0.13 mmol), (4-fluorophenyl)boronic acid (22.1 mg, 0.16 mmol), K_2CO_3 (36.4 mg, 0.26 mmol), $Pd(OAc)_2$ (2.95 mg, 10% mol) and Xantphos (7.61 mg, 13.2 µmol), then backfilled with N_2 three times. A 3:1 mixture of 1,4-dioxane/H_2O (4 mL) was added and the reaction degassed for 5 min then heated at 100 °C for 8 h. The reaction was cooled to rt and filtered through celite and the solvent removed under reduced pressure to give the crude product that was purified by reversed-phase flash chromatography on silica (5-55%

MeOH in H_2O) to give *the title compound* as a light yellow solid (17.0 mg, 40%); **^{1}H NMR** (500 MHz, CD_3OD): δ 8.46 (dt, *J* = 4.9, 1.3 Hz, 1H), 7.97 (d, *J* = 7.6 Hz, 1H), 7.77 (td, *J* = 7.7, 1.8 Hz, 1H), 7.65 (dt, *J* = 8.0, 1.1 Hz, 1H), 7.49 – 7.42 (m, 2H), 7.35 – 7.21 (m, 3H), 6.96 (d, *J* = 2.5 Hz, 1H), 6.66 (dd, *J* = 7.6, 2.5 Hz, 1H), 3.93 (s, 3H); ***m/z*** (ESI+) 320 ($[M+H]^+$, 100%).

3-(6-Chloropyridin-2-yl)-7-methoxy-2-(pyridin-2-yl)imidazo[1,2-*a*]pyridine (OSA_000857)

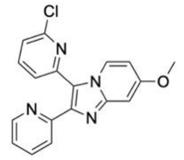

A microwave vial was charged with 4-methoxypyridin-2-amine (50.0 mg, 0.40 mmol), 2-bromo-1-(pyridin-2-yl)ethan-1-one hydrobromide (113 mg, 0.40 mmol), 2-bromo-6-chloropyridine (155 mg, 0.81 mmol), and KOAc (79.0 mg, 0.81 mmol). DMF (1.3 mL) was added and the mixture was purged with N_2 for 1 min, then $Pd(OAc)_2$ (10 mol %) was added. The mixture was heated under microwave conditions at 160 °C for 2.5 h. The crude product was directly purified by reversed-phase flash chromatography on silica (10-80% MeOH in H_2O) to give *the title compound* as a yellow solid (13.0 mg, 9%); **^{1}H NMR** (500 MHz, $CDCl_3$): δ 9.02 (d, *J* = 7.7 Hz, 1H), 8.57 (d, *J* = 4.8 Hz, 1H), 7.94 (d, *J* = 7.9 Hz, 1H), 7.76 (td, *J* = 7.8, 1.8 Hz, 1H), 7.57 (d, *J* = 7.4 Hz, 2H), 7.25 – 7.21 (m, 2H), 7.03 – 6.94 (m, 1H), 6.64 (dd, *J* = 7.7, 2.5 Hz, 1H), 3.91 (s, 3H); ***m/z*** (ESI+) 337 ($[M+H]^+$, 100%).

***N*-(2-Fluorophenyl)-7-methoxy-2-(1-methyl-1*H*-pyrazol-4-yl)imidazo[1,2-*a*]pyridin-3-amine (OSA_000855)**

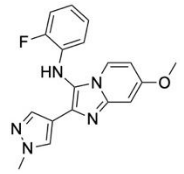

A microwave vial was charged with 4-methoxypyridin-2-amine (50.0 mg, 0.40 mmol, 1.00 equiv.), $Yb(OTf)_3$ (7.49 mg, 12.1 µmol, 0.03 equiv.), 1-methyl-1*H*-pyrazole-4-carbaldehyde (88.7 mg, 0.81 mmol, 2.00 equiv.), and 1-fluoro-2-isocyanobenzene (synthesised according to the procedure for **S25**, 97.6 mg, 0.81 mmol, 2.00 equiv.). The vial was closed with a crimp seal and the mixture heated in the microwave at 120

°C for 30 min. The reaction was diluted with EtOAc and the organic layer washed with H_2O, brine, dried ($MgSO_4$), filtered and concentrated under reduced pressure to give the crude product that was purified by reversed-phase flash chromatography on silica (30-70% MeOH in H_2O) to give *the title compound* as a light orange solid (35.0 mg, 26%); **^{1}H NMR** (500 MHz, $CDCl_3$): δ 7.85 (s, 1H), 7.78 (s, 1H), 7.68 (d, *J* = 7.4 Hz, 1H), 7.13 (ddd, *J* = 11.6, 8.1, 1.5 Hz, 1H), 6.89 (d, *J* = 2.3 Hz, 1H), 6.87 – 6.82 (m, 1H), 6.80 – 6.73 (m, 1H), 6.51 (dd, *J* = 7.4, 2.4 Hz, 1H), 6.28 (ddd, *J* = 9.2, 8.0, 1.6 Hz, 1H), 5.70 (s, 1H), 3.89 (s, 3H), 3.87 (s, 3H); ***m/z*** (ESI+) 338 ($[M+H]^+$, 100%).

2-(4-(Dimethylamino)phenyl)-*N*-(4-fluorophenyl)-7-methylimidazo[1,2-*a*]pyridin-3-amine (OSA_000846)

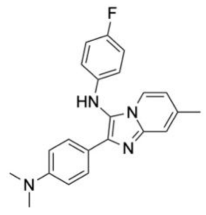

To a mixture of 4-methylpyridin-2-amine (100 mg, 0.92 mmol, 1.0 equiv.), 1-fluoro-4-isocyanobenzene (160 mg, 0.92 mmol, 1.0 equiv.), 4-(dimethylamino)benzaldehyde (138 mg, 0.92 mmol, 1.0 equiv.) in MeOH (5 mL, 184 mM) was added *p*-TsOH (79.6 mg, 0.46 mmol, 0.5 equiv.). The reaction mixture was stirred at 80 °C for 12 h. The reaction mixture was concentrated under reduced pressure to give a residue that was purified by prep-HPLC (HCl condition) to give *the title compound* as a yellow solid (9.00 mg, 2%); **^{1}H NMR** (400MHz, DMSO-d_6): δ 8.71 (s, 1H), 8.27 (d, *J* = 6.0 Hz, 1H), 7.86 (s, *J* = 8.8 Hz, 2H), 7.77 (s, 1H), 7.29 (d, *J* = 6.0 Hz, 1H), 7.01 (dd, *J* = 8.8, 8.8 Hz, 2H), 6.86 (s, *J* = 8.8 Hz, 2H), 6.72 (dd, *J* = 8.8, 4.4 Hz, 2H), 2.96 (s, 6H), 2.55 (s, 3H); ***m/z*** (ESI+) 361 ($[M+H]^+$, 100%).

7-Methoxy-2-(pyridin-2-yl)-*N*-(2,4,4-trimethylpentan-2-yl)imidazo[1,2-*a*]pyridin-3-amine (S38)

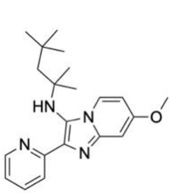

To a mixture of 4-methoxypyridin-2-amine (2.00 g, 16.1 mmol, 1.0 equiv.), 2-isocyano-2,4,4-trimethylpentane (2.24 g, 16.1 mmol, 1.0 equiv.), picolinaldehyde (1.73 g, 16.1 mmol, 1.0 equiv.) in MeOH (88 mL, 184 mM) was added *p*-TsOH (1.39 g, 8.06 mmol, 0.5 equiv.). The reaction mixture was stirred at 80 °C for 12 h. The reaction mixture was concentrated under reduced pressure to give *the title compound* as a yellow oil (5.30 g, 93%); ***m/z*** (ESI+) 353 ($[M+H]^+$, 100%).

7-Methoxy-2-(pyridin-2-yl)imidazo[1,2-*a*]pyridin-3-amine hydrochloride (S39)

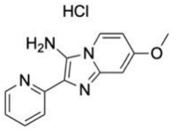

To a solution of **S38** (1.00 g, 2.84 mmol) in MeOH (10 mL) was added HCl/dioxane (4 M, 20 mL). The mixture was stirred at 25 °C for 12 h. The mixture was concentrated under reduced pressure to give *the title compound* as a yellow solid (800 mg); **^{1}H NMR** (400MHz, DMSO-d_6): δ 8.67 – 8.65 (m, 2H), 8.06 (d, *J* = 8.0 Hz, 1H), 7.97 (dd, *J* = 6.0, 2.0 Hz, 1H), 7.32 (dd, *J* = 6.0, 1.6 Hz, 1H), 7.19 (dd, *J* = 7.6, 2.4 Hz, 1H), 7.04 (d, *J* = 2.4 Hz, 1H), 3.98 (s, 3H).

7-Methoxy-*N*-(2-methylpyridin-4-yl)-2-(pyridin-2-yl)imidazo[1,2-*a*]pyridin-3-amine (OSA_000854)

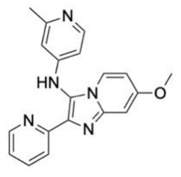

To a solution of **S39** (200 mg, 0.72 mmol, 1.0 equiv.) and 4-bromo-2-methylpyridine (124 mg, 0.72 mmol, 1.0 equiv.) in PhMe (5 mL, 144 mM) was added Xantphos (83.6 mg, 0.14 mmol, 0.2 equiv.), $Pd_2(dba)_3$ (66.2 mg, 72.3 μmol, 0.1 equiv.) and *t*-BuONa (208.38 mg, 2.17 mmol, 3.0 equiv.). The mixture was degassed and stirred at 110 °C for 12 h under N_2 atmosphere. The reaction mixture was concentrated under reduced pressure to give a residue that was purified by prep-HPLC (NH_4OH condition) to give *the title compound* as a yellow solid (51.2 mg, 21%); **^{1}H NMR** (400 MHz, DMSO-d_6): δ 8.73 (s, 1H), 8.52 (d, *J* = 4.0 Hz, 1H), 8.06 (d, *J* = 7.6 Hz, 1H), 7.98 (d, *J* = 4.8 Hz, 1H), 7.86

– 7.84 (m, 1H), 7.77 (d, J = 7.6 Hz, 1H), 7.24 (dd, J = 7.2, 4.8 Hz, 1H), 7.04 (d, J = 2.4 Hz, 1H), 6.66 (dd, J = 7.2, 2.4 Hz, 1H), 6.28 – 6.25 (m, 1H), 3.88 (s, 3H), 3.23 (s, 3H); ***m/z*** (ESI+) 332 ($[M+H]^+$, 100%).

1-Methyl-1*H*-imidazole-4-carbonyl chloride (S40)

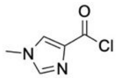

To a solution of 1-methylimidazole-4-carboxylic acid (300 mg, 2.38 mmol, 1.0 equiv.) and DMF (17.4 mg, 0.24 mmol, 0.1 equiv.) in CH_2Cl_2 (5 mL, 476 mM) was added oxalyl chloride (332 mg, 2.62 mmol, 1.1 equiv.) dropwise at 20 °C. The mixture was stirred at 20 °C for 1 h. The mixture was concentrated under reduced pressure to give *the title compound* as yellow oil (320 mg, 93%); used without further purification or characterization.

***N*-(7-Methoxy-2-(pyridin-2-yl)imidazo[1,2-*a*]pyridin-3-yl)-1-methyl-1*H*-imidazole-4-carboxamide (S41)**

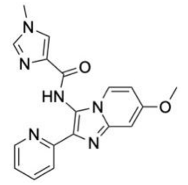

To a solution of **S40** (220 mg, 1.52 mmol, 1 equiv.) in CH_2Cl_2 (4 mL) was added a solution of **S39** (421 mg, 1.52 mmol, 1 equiv.) and Et_3N (0.64 mL, 4.57 mmol, 3 equiv.) in CH_2Cl_2 (1 mL) dropwise at 0 °C. The resulting mixture was stirred at 25 °C for 1 h. The mixture was concentrated under reduced pressure to give a residue that was purified by flash column chromatography to give *the title compound* as yellow solid (400 mg, 75% yield); ***m/z*** (ESI+) 349 ($[M+H]^+$, 100%).

7-Methoxy-*N*-((1-methyl-1*H*-imidazol-4-yl)methyl)-2-(pyridin-2-yl)imidazo[1,2-*a*]pyridin-3-amine (OSA_000853)

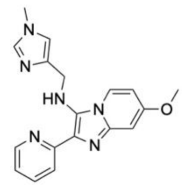

To a solution of **S41** (220 mg, 0.63 mmol, 1 equiv.) in THF (10 mL, 63 mM) was added BH_3•Me_2S complex (10 M, 0.63 mL, 6.32 mmol, 10 equiv.) dropwise at 25 °C. After stirring at 25 °C for 1 h, the mixture was stirred at 70 °C for 3 h. The mixture was quenched by addition MeOH (5 mL) at 0 °C, followed by stirring at 70 °C for 1 h. The mixture was concentrated under reduced pressure to give a residue that was purified by prep-HPLC (HCl condition) to give *the title compound* as a yellow oil (28.5 mg, 10%, FA salt); **^{1}H NMR** (400 MHz, DMSO-d_6): δ 8.53 (d, J = 4.0 Hz 1H), 8.19 – 8.18 (m, 1H), 8.17 (s, 2H), 7.96 (d, J = 8.0 Hz, 1H), 7.84 – 7.80 (m, 1H), 7.47 (s, 1H), 7.21 – 7.18 (m, 1H), 6.95 (s, 1H), 6.85 (d, J = 2.0 Hz, 1H), 6.58 (dd, J = 7.6, 2.4 Hz, 1H) 6.46 (s, 1H), 4.08 (d, J = 4.8 Hz, 2H), 3.83 (s, 3H), 3.55 (s, 3H); ***m/z*** (ESI+) 335 ($[M+H]^+$, 100%).

2-(3,4-Dimethoxyphenyl)-6-methyl-*N*-(2,4,4-trimethylpentan-2-yl)imidazo[1,2-*a*]pyridin-3-amine (S42)

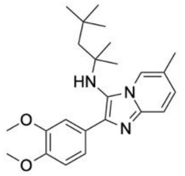

To a mixture of 5-methylpyridin-2-amine (450 mg, 4.16 mmol, 1.0 equiv.), 2-isocyano-2,4,4-trimethylpentane (579 mg, 4.16 mmol, 1.0 equiv.), 3,4-dimethoxybenzaldehyde (691 mg, 4.16 mmol, 1.0 equiv.) in MeOH (22.6 mL, 184 mM) was added *p*-TsOH (358 mg, 2.08 mmol, 0.5 equiv.). The reaction mixture was stirred at 80 °C for 12 h. The reaction mixture was concentrated under reduced pressure to give *the title compound* as yellow oil (1.7 g, crude); used without further purification; **^{1}H NMR** (400 MHz, DMSO-d_6): δ 8.16 (s, 1H), 7.67 – 7.63 (m, 2H), 7.35 (d, J = 9.2 Hz, 1H), 7.01 (dd, J = 9.2, 1.2 Hz, 1H), 6.96 (d, J = 8.4 Hz, 1H), 4.32 (s, 1H), 3.82 (s, 3H), 3.78 (s, 3H), 2.31 (s, 3H), 1.63 (s, 2H), 0.99 (s, 9H), 0.93 (s, 6H).

***N*-(2-(3,4-Dimethoxyphenyl)-6-methylimidazo[1,2-*a*]pyridin-3-yl)-2,2,2-trifluoroacetamide (S43)**

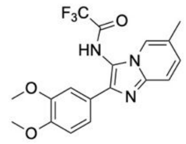

To a solution of **S42** (2.10 g, 5.31 mmol) in CH_2Cl_2 (20 mL) was added TFA (30.8 g, 270 mmol). The mixture was stirred at rt for 16 h. The mixture was poured into H_2O (50 mL) and neutralized with $NaHCO_3$ powder to pH 7-8, then extracted with CH_2Cl_2 (50 mL × 4) and the combined organic layers were dried (Na_2SO_4), filtered and concentrated under reduced pressure to give a yellow solid which was triturated with EtOAc (20 mL) to give *the title compound* as an off-white solid (1.35 g, 66%); ***m/z*** (ESI+) 380 ($[M+H]^+$, 100%).

2-(3,4-Dimethoxyphenyl)-6-methylimidazo[1,2-*a*]pyridin-3-amine (S44)

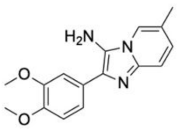

To a solution of **S42** (1.25 g, 3.30 mmol) in EtOH (20 mL) was added 2.5 M NaOH (15 mL). The reaction mixture was stirred at 90 °C for 4 h, then neutralized with conc. HCl solution and extracted with CH_2Cl_2 (50 mL x 3). The combined organic layers were dried (Na_2SO_4), filtered and concentrated under reduced pressure to give a residue that was purified by flash chromatography on silica to give *the title compound* as a yellow solid (860 mg, 92%); **^{1}H NMR** (400 MHz, DMSO-d_6): δ 8.03 (s, 1H), 7.64 (d, J = 2.0 Hz, 1H), 7.57 (dd, J = 8.4, 2.0 Hz, 1H), 7.32 (d, J = 8.8 Hz, 1H), 6.98 (d, J = 8.4 Hz, 1H), 6.91 (d, J = 7.6 Hz, 1H), 4.93 (s, 2H), 3.83 (s, 3H), 3.78 (s, 3H), 2.28 (s, 3H).

2-(3,4-Dimethoxyphenyl)-*N*-(furan-2-ylmethyl)-6-methylimidazo[1,2-*a*]pyridin-3-amine hydrochloride (OSA_000849)

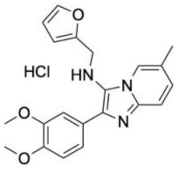

To a solution of **S44** (450 mg, 1.59 mmol, 1.00 equiv.) in MeOH (10 mL, 159 mM) was added furan-2-carbaldehyde (381 mg, 3.97 mmol, 2.50 equiv.) and TFA (232 mg, 2.03 mmol, 1.28 equiv.). The mixture was stirred at rt for 20 h. $NaBH_3CN$ (499 mg, 7.94 mmol, 5.00 equiv.) was added and the mixture was stirred at rt for 20 h. H_2O (30 mL) was added and the mixture was extracted with EtOAc (30 mL x 3). The

combined organic layers were dried (Na_2SO_4), filtered and concentrated under reduced pressure to give a residue that was purified by prep-HPLC (HCl condition) to give *the title compound* as a light yellow solid (133 mg, 20%); **^{1}H NMR** (400 MHz, DMSO-d_6): δ 15.14 (br s, 1H), 8.30 (s, 1H), 7.80 (d, J = 9.2 Hz, 1H), 7.79 – 7.25 (m, 2H), 7.68 – 7.67 (m, 1H), 7.13 (d, J = 8.4 Hz, 1H), 6.29 – 6.28 (m, 1H), 6.18 (J = 2.8 Hz, 1H), 5.93 (br s, 1H), 4.18 (J = 4.4 Hz, 2H), 3.87 (s, 3H), 3.84 (s, 3H), 2.40 (s, 3H); ***m/z*** (ESI+) 364 ([M+H]$^+$, 100%).

Hypha Metabolism Screen

3-(Benzofuran-5-yl)-2-(pyridin-2-yl)-6,7-dihydro-5*H*-pyrrolo[1,2-*a*]imidazol-7-ol (OSA_000997)

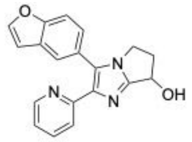

Isolated following the procedure in Supplementary Information - Biology; **^{1}H NMR** (500 MHz, CD_3OD): δ 8.40 (d, J = 4.1 Hz, 1H), 7.81 (d, J = 2.4 Hz, 1H), 7.71 (td, J = 7.8, 1.8 Hz, 1H), 7.66 (d, J = 1.8 Hz, 1H), 7.53 (d, J = 8.5 Hz, 1H), 7.51 (d, J = 7.8 Hz, 1H), 7.29 (dd, J = 8.4, 1.8 Hz, 1H), 7.23 (dd, J = 7.5, 5.2 Hz, 1H), 6.86 (d, J = 1.9 Hz, 1H), 5.17 (dd, J = 7.1, 3.0 Hz, 1H), 4.22 (dt, J = 10.9, 7.1 Hz, 1H), 4.00 (ddd, J = 11.0, 8.5, 3.9 Hz, 1H), 2.99 (ddd, J = 15.4, 13.6, 7.3 Hz, 1H), 2.50 (ddt, J = 14.2, 7.3, 3.5 Hz, 1H); **^{13}C NMR** (126 MHz, CD_3OD): δ 156.2, 156.0, 154.9, 149.9, 147.5, 141.7, 138.1, 129.9, 129.4, 126.9, 125.9, 124.0, 123.2 (2C), 112.5, 107.7, 66.7, 43.9, 38.1; ***m/z*** (ESI+) 318 ([M+H]$^+$, 100%); **HRMS** (ESI+) found 318.1234 ([M+H]$^+$), $C_{19}H_{16}N_3O_2^+$ requires 318.1237.

^{1}H and ^{13}C NMR Spectra of Final Compounds

3-(Benzo[*b*]thiophen-5-yl)-2-(pyridin-2-yl)-6,7-dihydro-5*H*-pyrrolo[1,2-*a*]imidazole (OSA_000822)

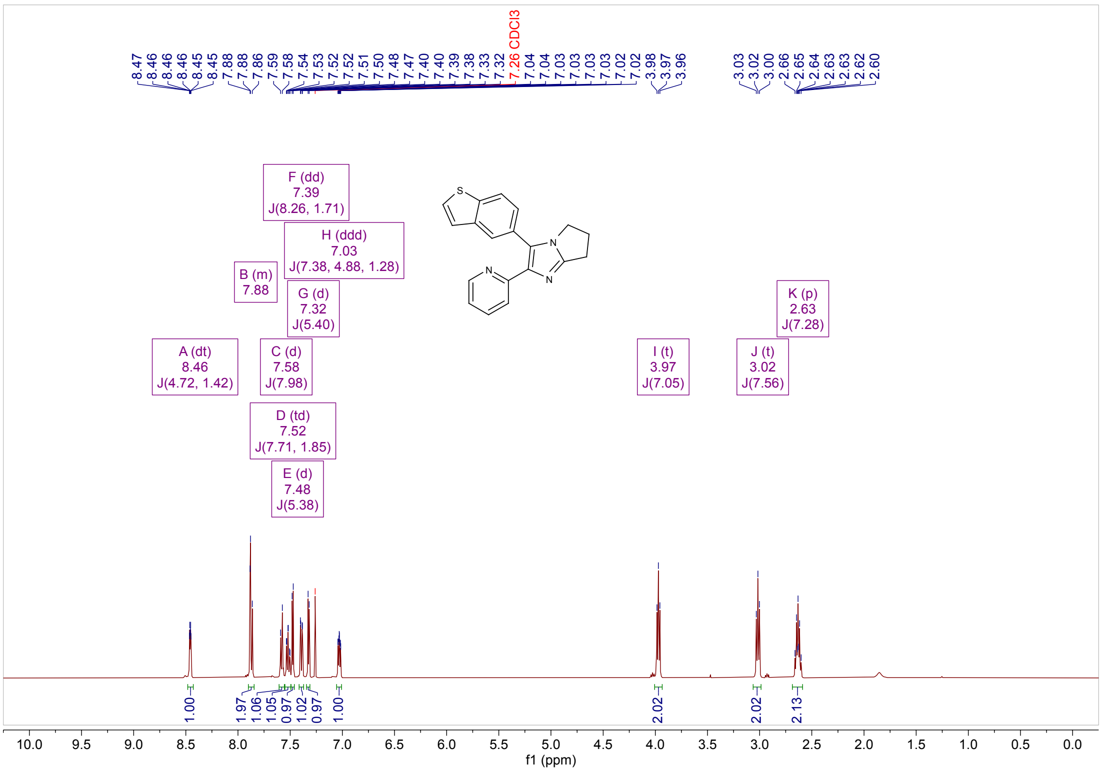
8.47
8.46
8.46
8.46
8.45
8.45
7.88
7.88
7.86
7.59
7.58
7.54
7.53
7.52
7.52
7.51
7.50
7.48
7.47
7.40
7.39
7.38
7.33
7.32
7.26 CDCl3
7.04
7.04
7.03
7.03
7.03
7.03
7.02
7.02
3.98
3.97
3.96
3.03
3.02
3.00
2.66
2.65
2.64
2.63
2.63
2.62
2.60
F (dd) 7.39 J(8.26, 1.71)
H (ddd) 7.03 J(7.38, 4.88, 1.28)
B (m) 7.88
G (d) 7.32 J(5.40)
K (p) 2.63 J(7.28)
A (dt) 8.46 J(4.72, 1.42)
C (d) 7.58 J(7.98)
I (t) 3.97 J(7.05)
J (t) 3.02 J(7.56)
D (td) 7.52 J(7.71, 1.85)
E (d) 7.48 J(5.38)
S
N
N
N
1.00
1.97
1.06
1.05
0.97
1.02
0.97
1.00
2.02
2.02
2.13
10.0 9.5 9.0 8.5 8.0 7.5 7.0 6.5 6.0 5.5 5.0 4.5 4.0 3.5 3.0 2.5 2.0 1.5 1.0 0.5 0.0
f1 (ppm)

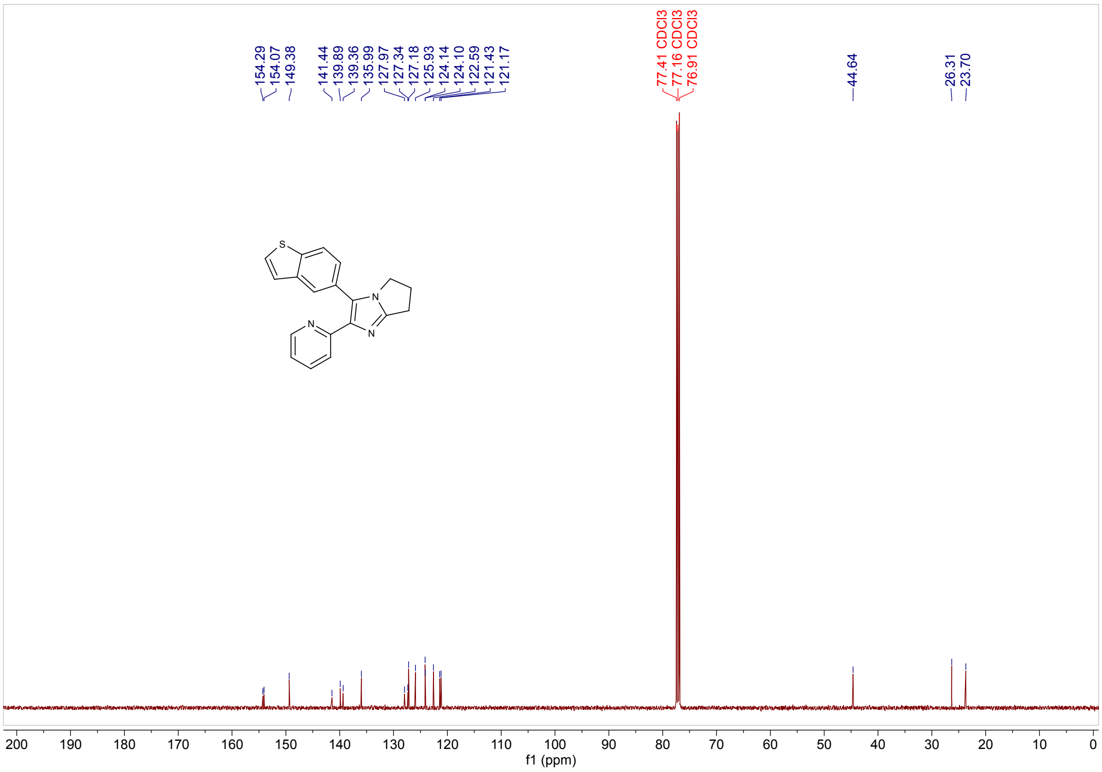
154.29
154.07
149.38
141.44
139.89
139.36
135.99
127.97
127.34
127.18
125.93
124.14
124.10
122.59
121.43
121.17
77.41 CDCl3
77.16 CDCl3
76.91 CDCl3
44.64
26.31
23.70
S
N
N
N
200 190 180 170 160 150 140 130 120 110 100 90 80 70 60 50 40 30 20 10 0
f1 (ppm)

3-(Benzofuran-6-yl)-2-(pyridin-2-yl)-6,7-dihydro-5*H*-pyrrolo[1,2-*a*]imidazole

(OSA_000829)

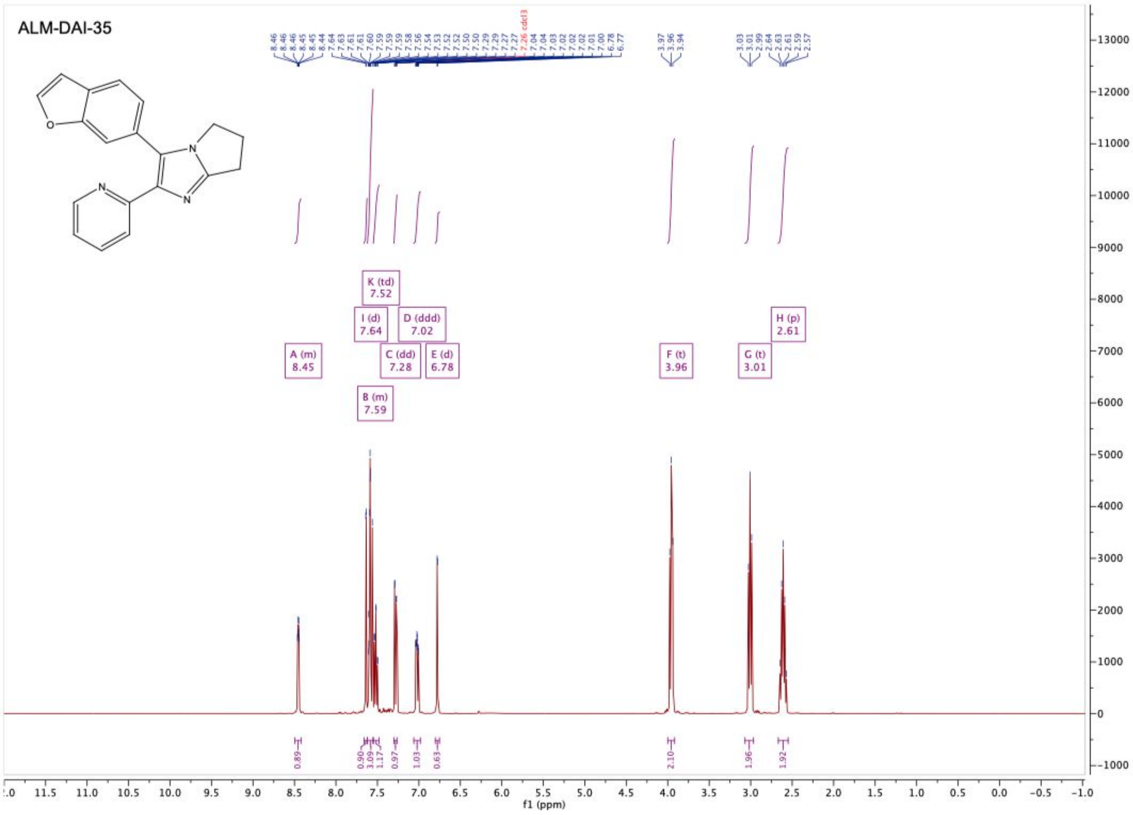

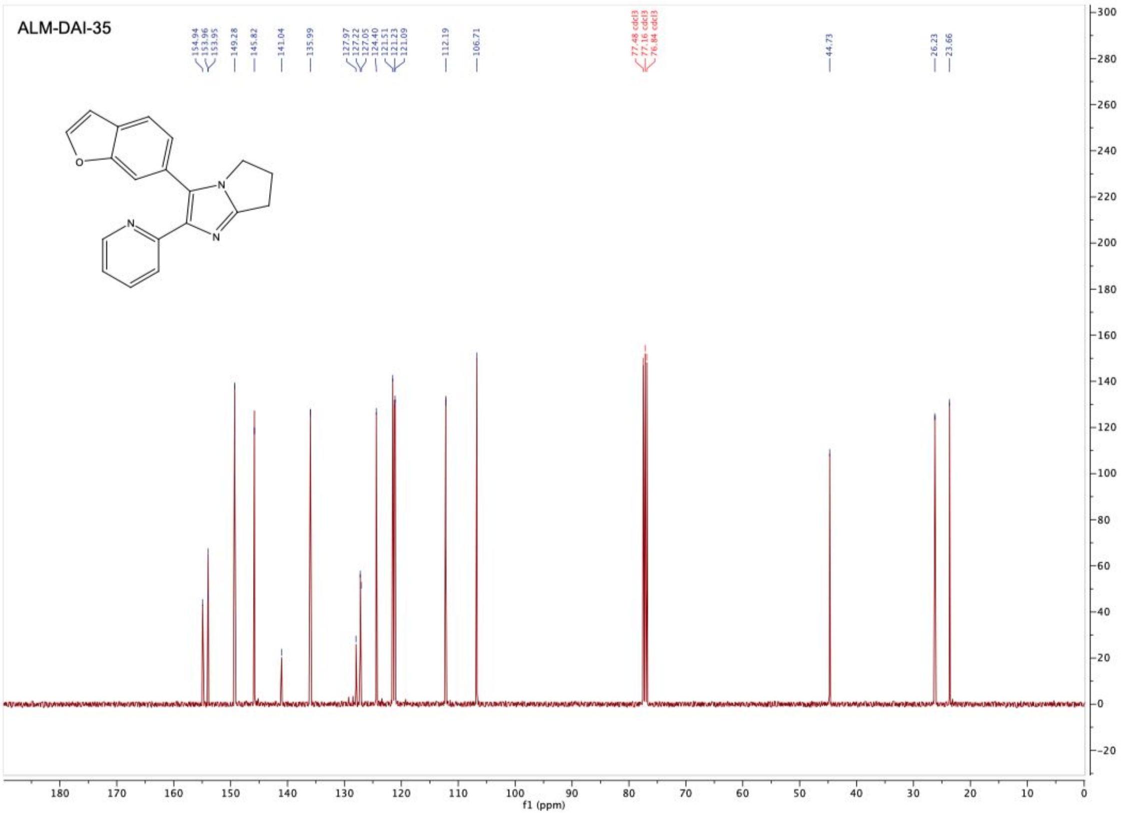

3-(Benzofuran-5-yl)-2-(pyridin-2-yl)-6,7-dihydro-5*H*-pyrrolo[1,2-*a*]imidazole (OSA_000821)

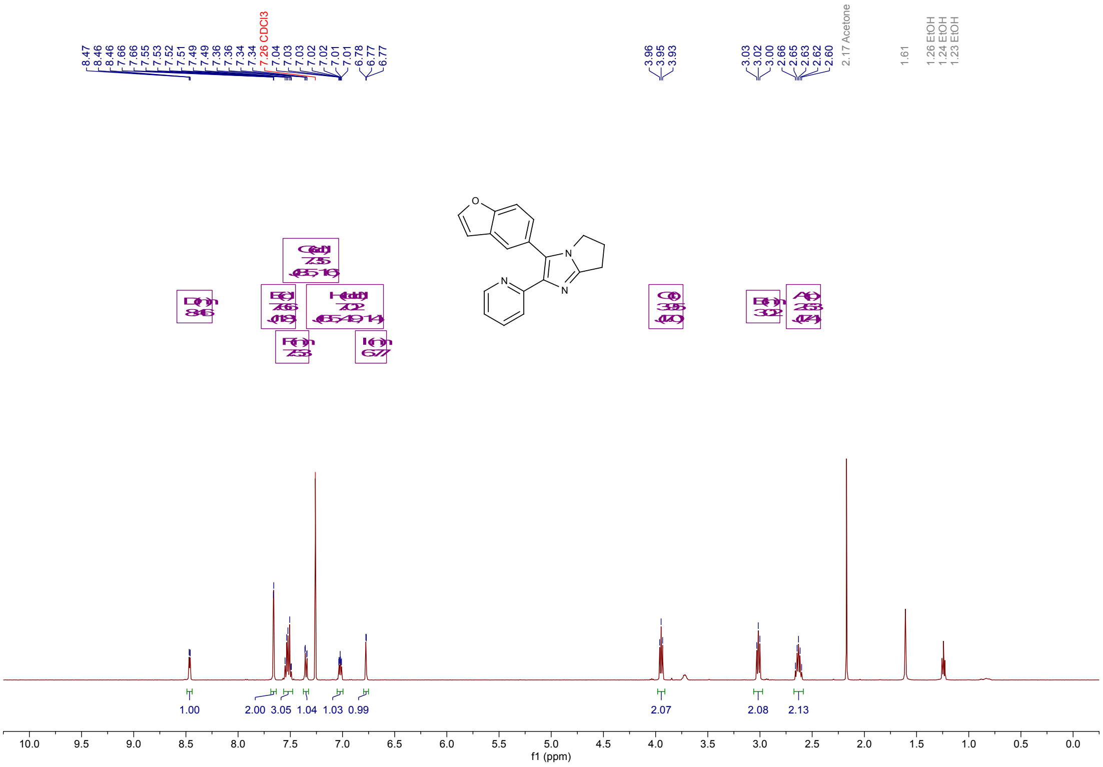

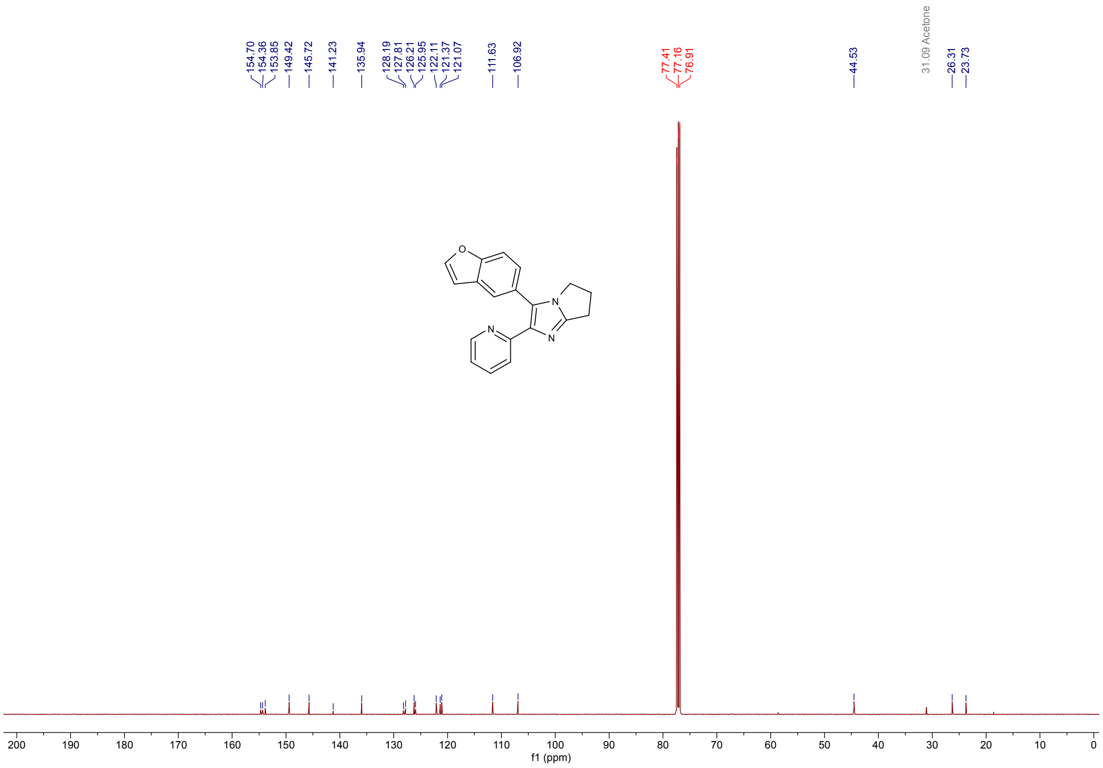

3-(Benzo[*b*]thiophen-6-yl)-2-(pyridin-2-yl)-6,7-dihydro-5*H*-pyrrolo[1,2-*a*]imidazole (OSA_000830)

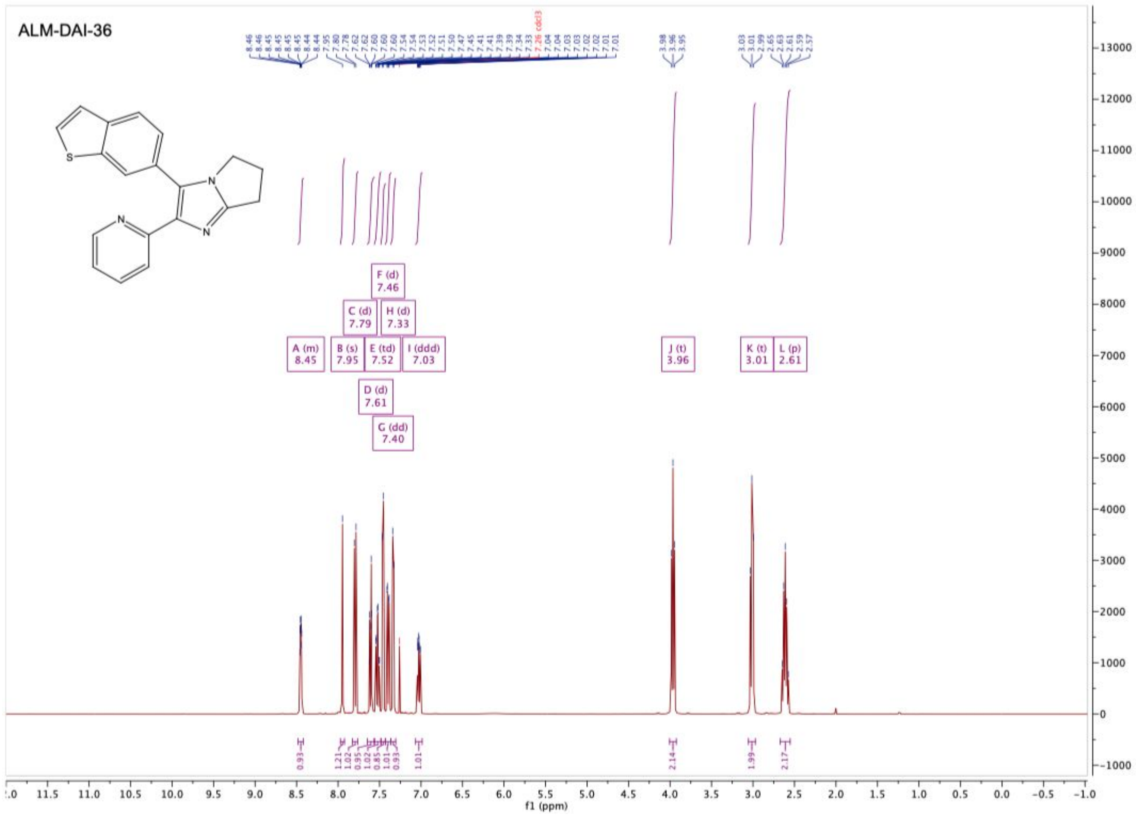

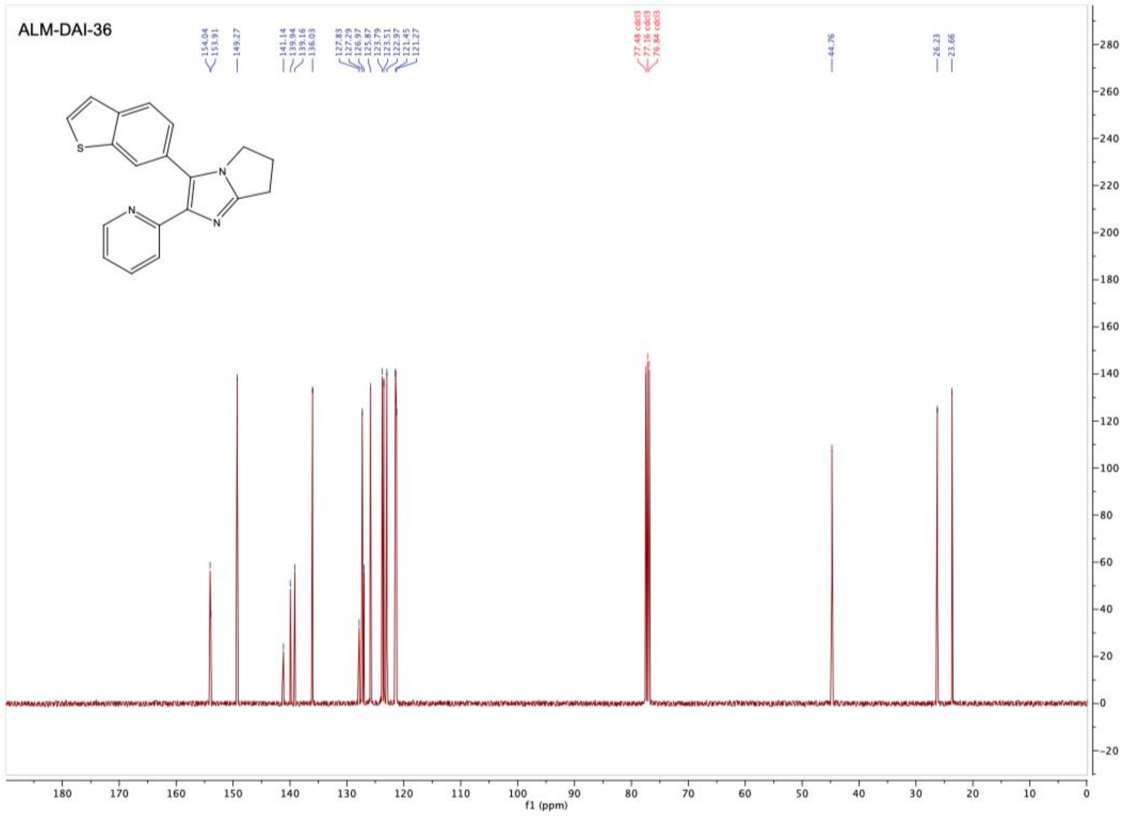

2-(Pyridin-2-yl)-3-(*p*-tolyl)-6,7-dihydro-5*H*-pyrrolo[1,2-*a*]imidazole (OSA_000865)

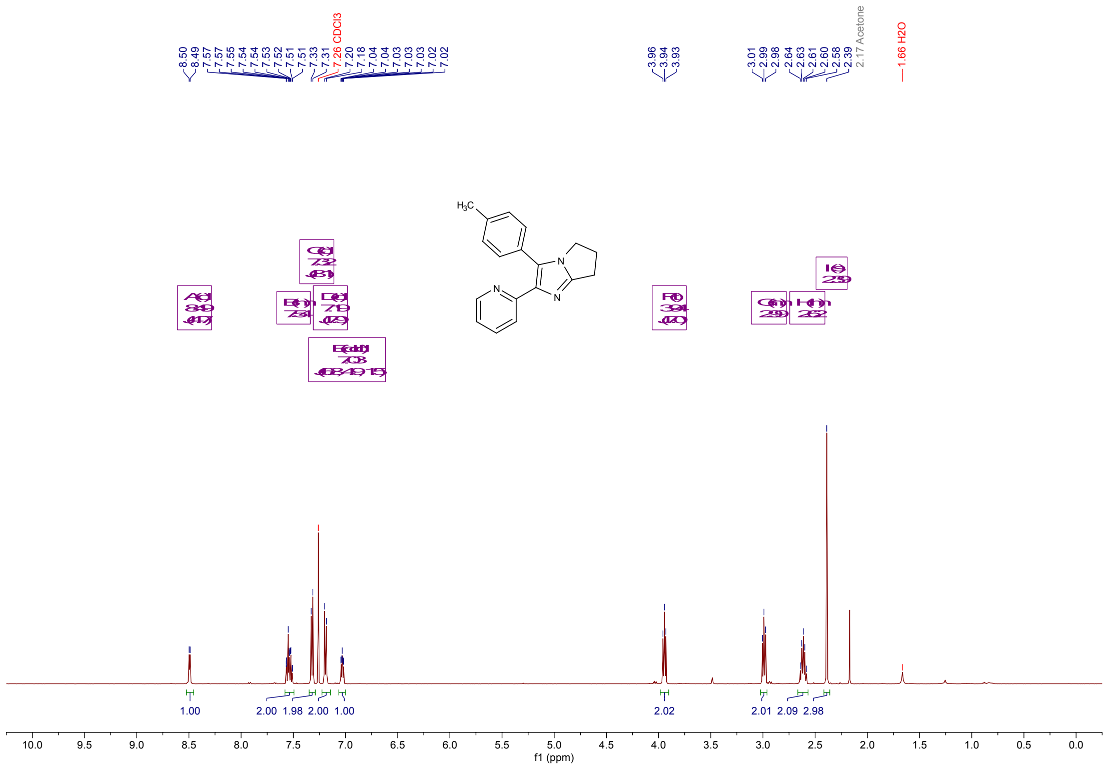

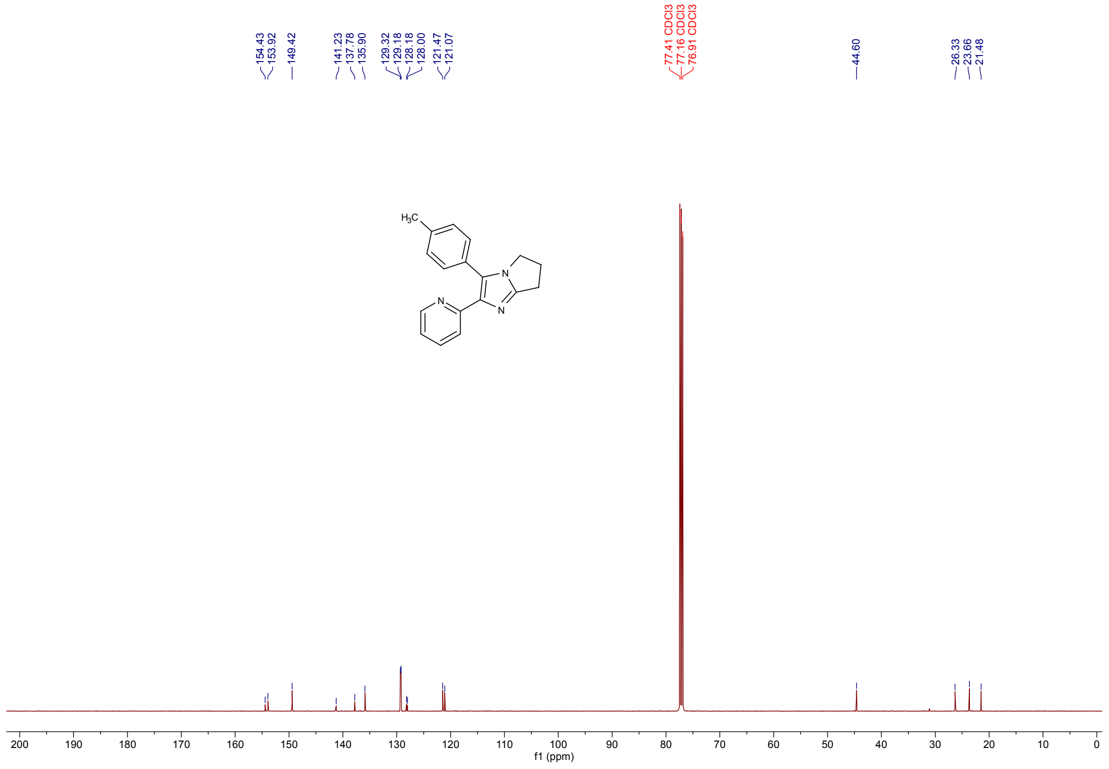

3-(4-(Methylthio)phenyl)-2-(pyridin-2-yl)-6,7-dihydro-5*H*-pyrrolo[1,2-*a*]imidazole (OSA_000824)

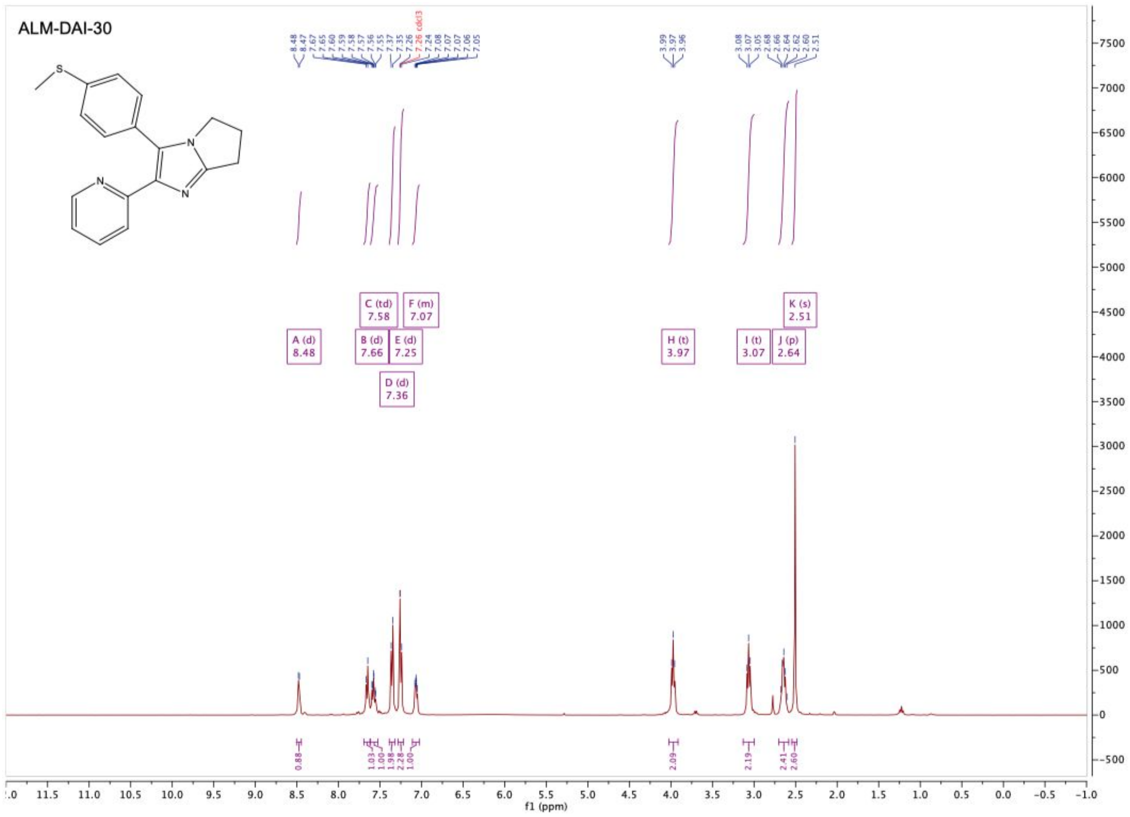

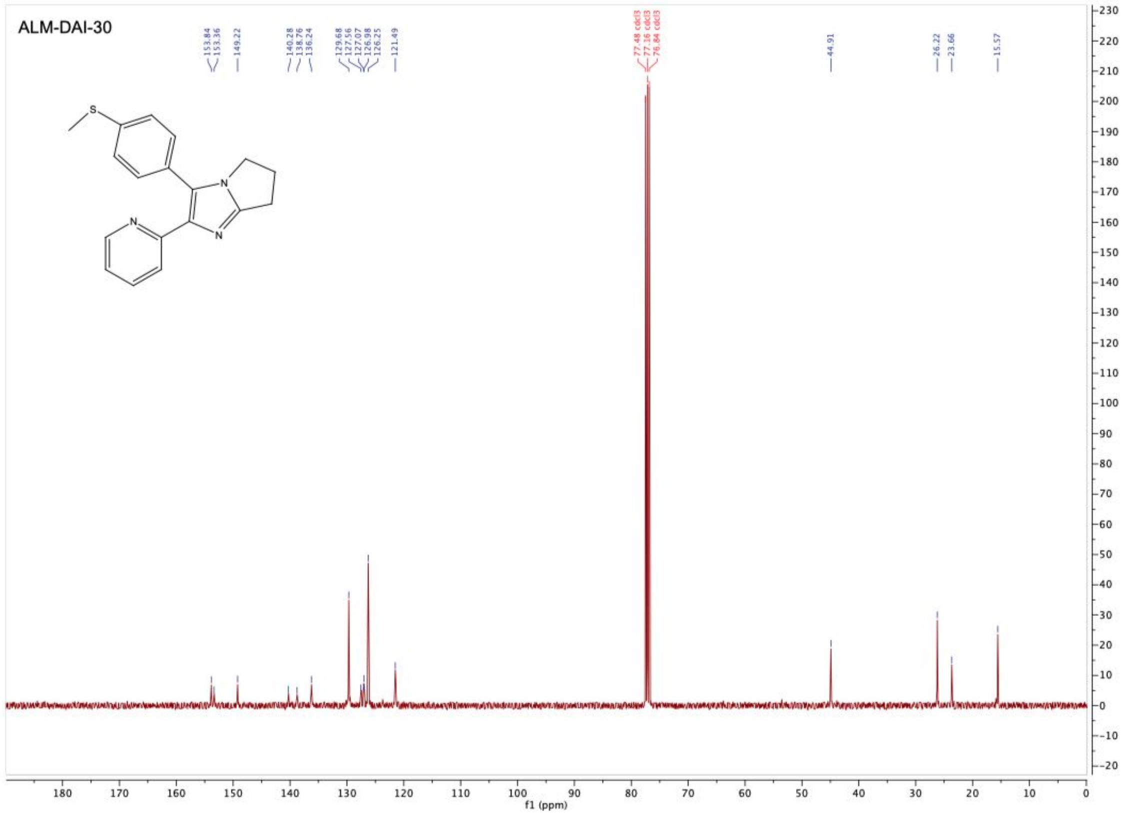

4-(2-(Pyridin-2-yl)-6,7-dihydro-5*H*-pyrrolo[1,2-*a*]imidazol-3-yl)benzonitrile

(OSA_000975)

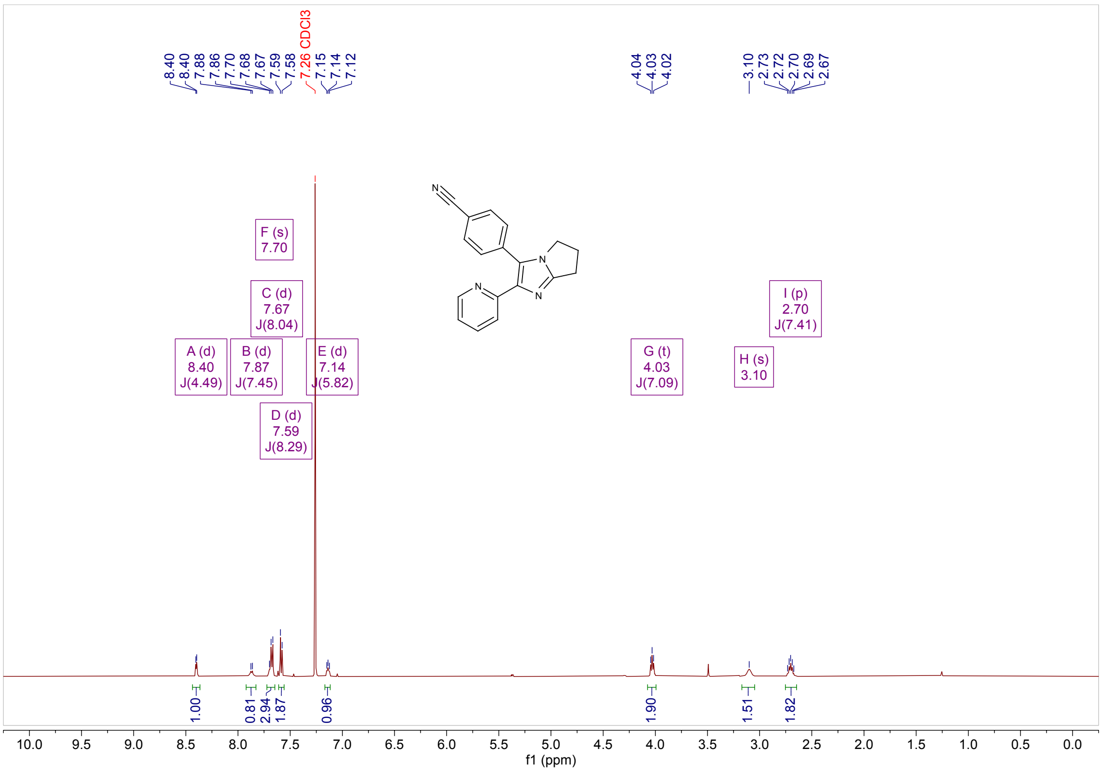

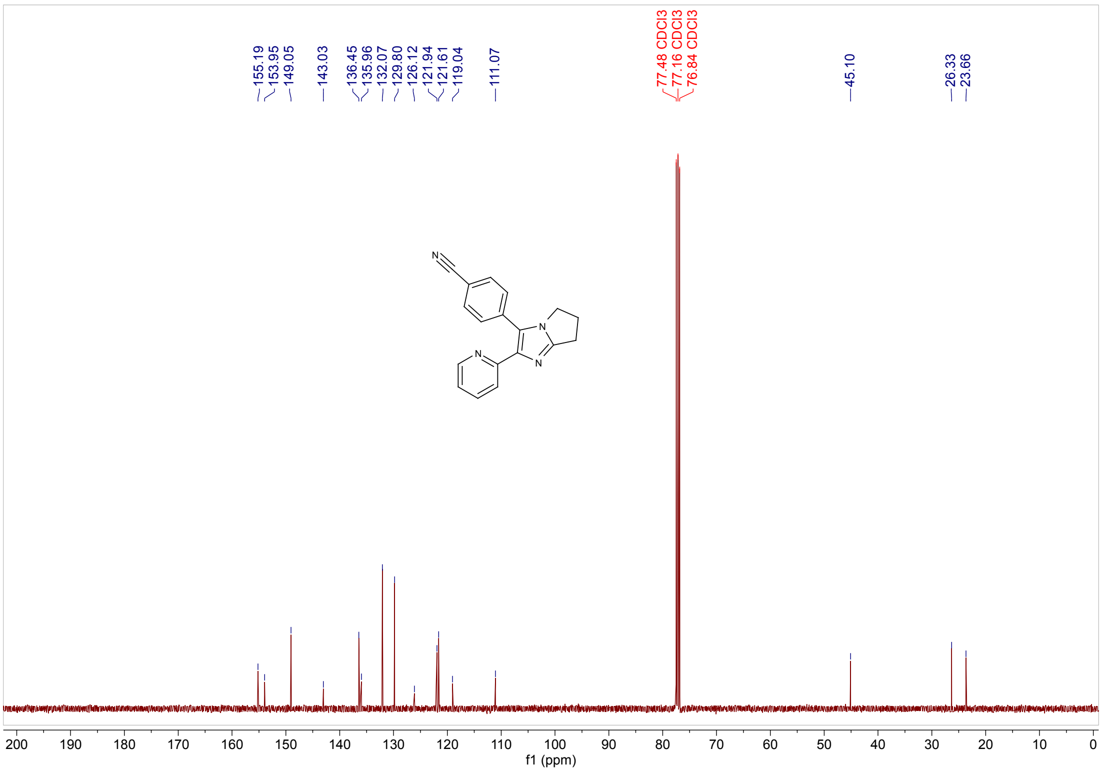

3-(3-(Methylthio)phenyl)-2-(pyridin-2-yl)-6,7-dihydro-5*H*-pyrrolo[1,2-*a*]imidazole (OSA_000826)

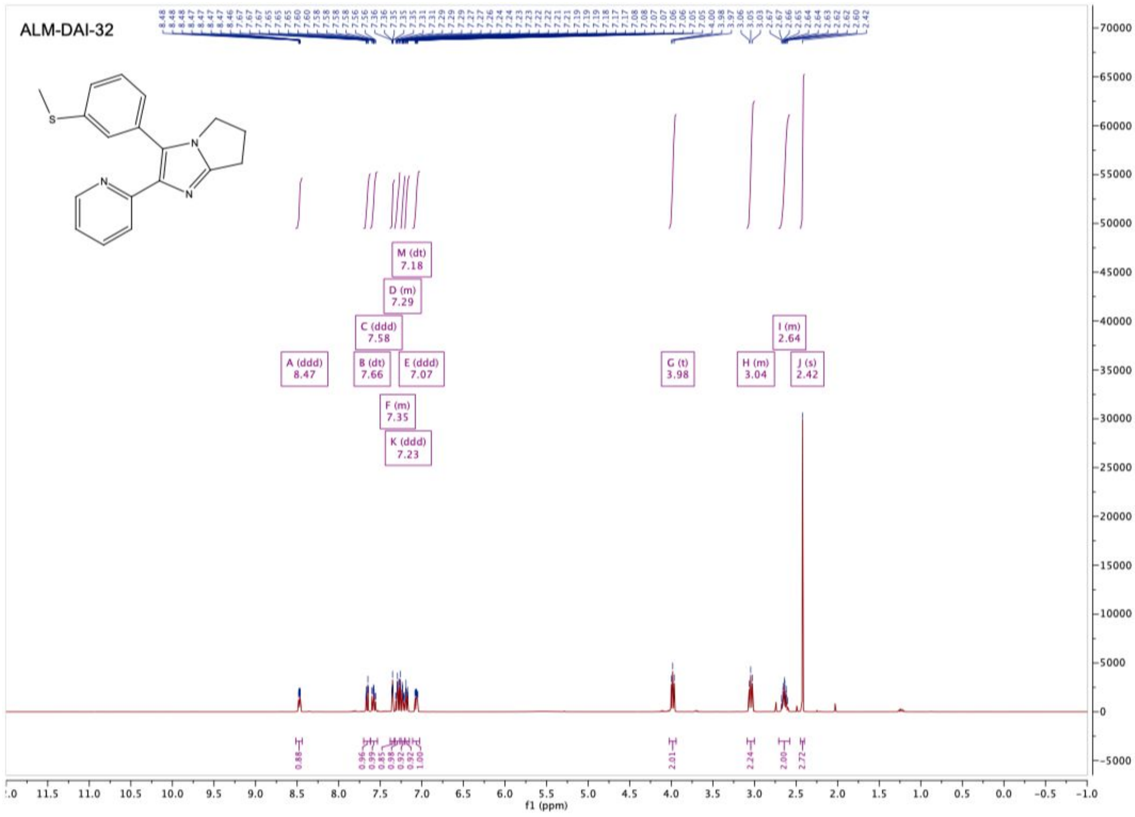

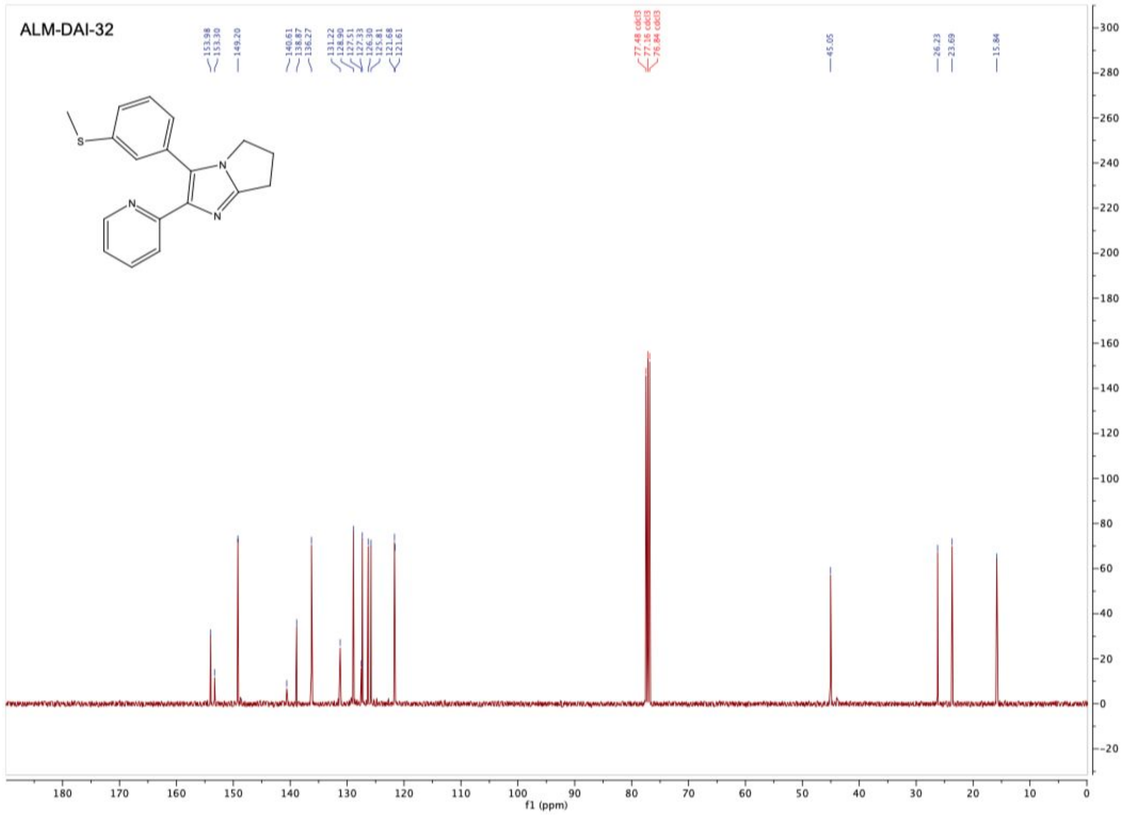

3-(2,3-Dihydrobenzo[*b*][1,4]dioxin-6-yl)-2-(pyridin-2-yl)-6,7-dihydro-5*H*-pyrrolo[1,2-*a*]imidazole (OSA_000819)

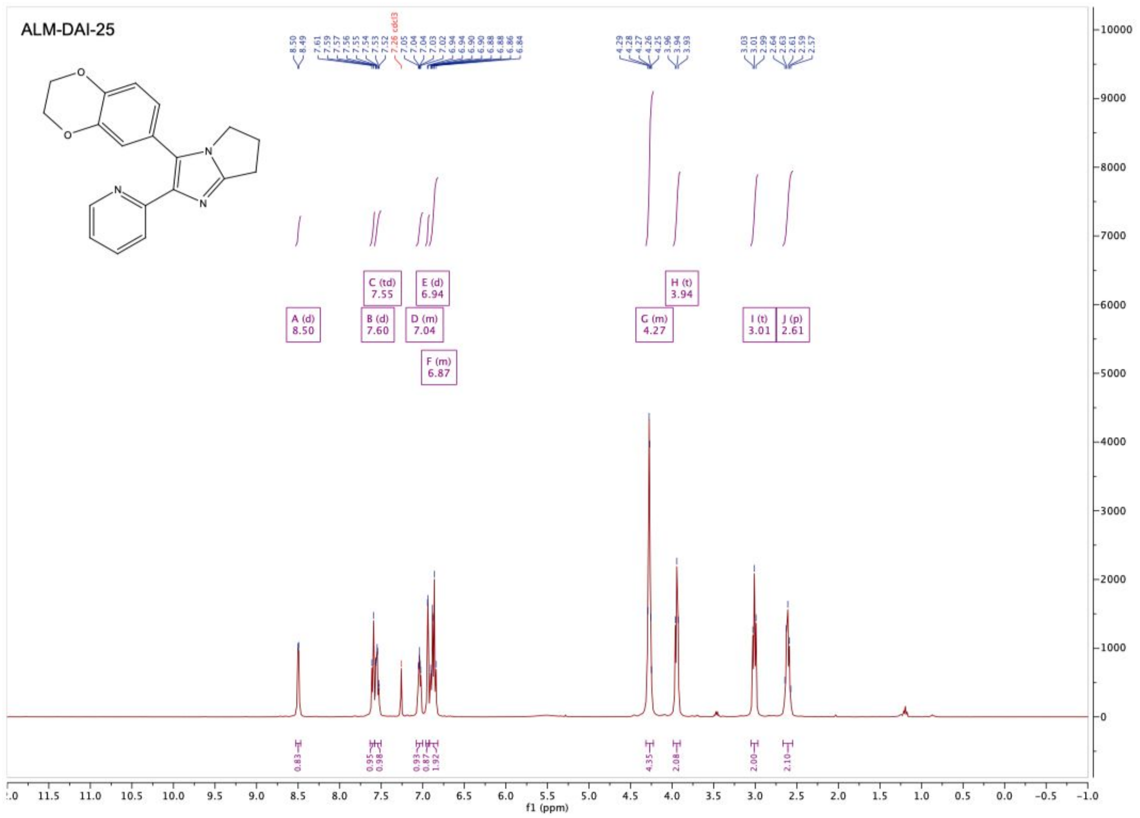

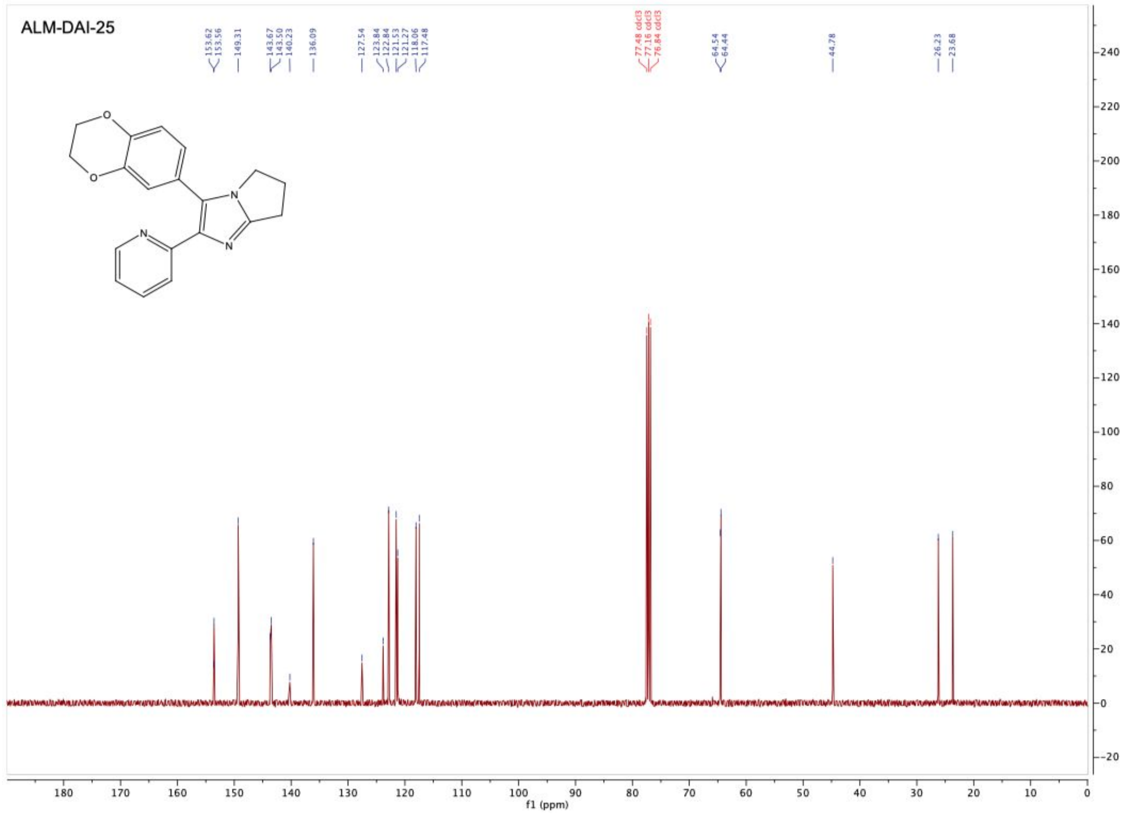

3-(Dibenzo[*b*,*d*]thiophen-2-yl)-2-(pyridin-2-yl)-6,7-dihydro-5*H*-pyrrolo[1,2-*a*]imidazole

(OSA_000831)

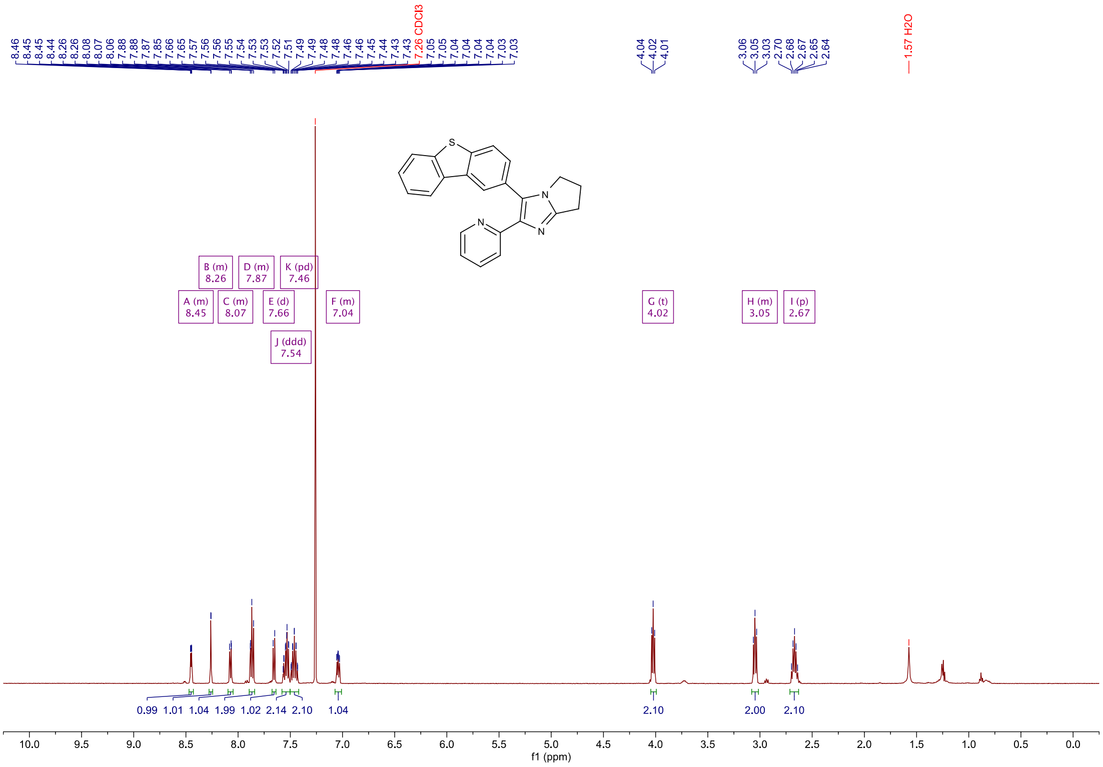

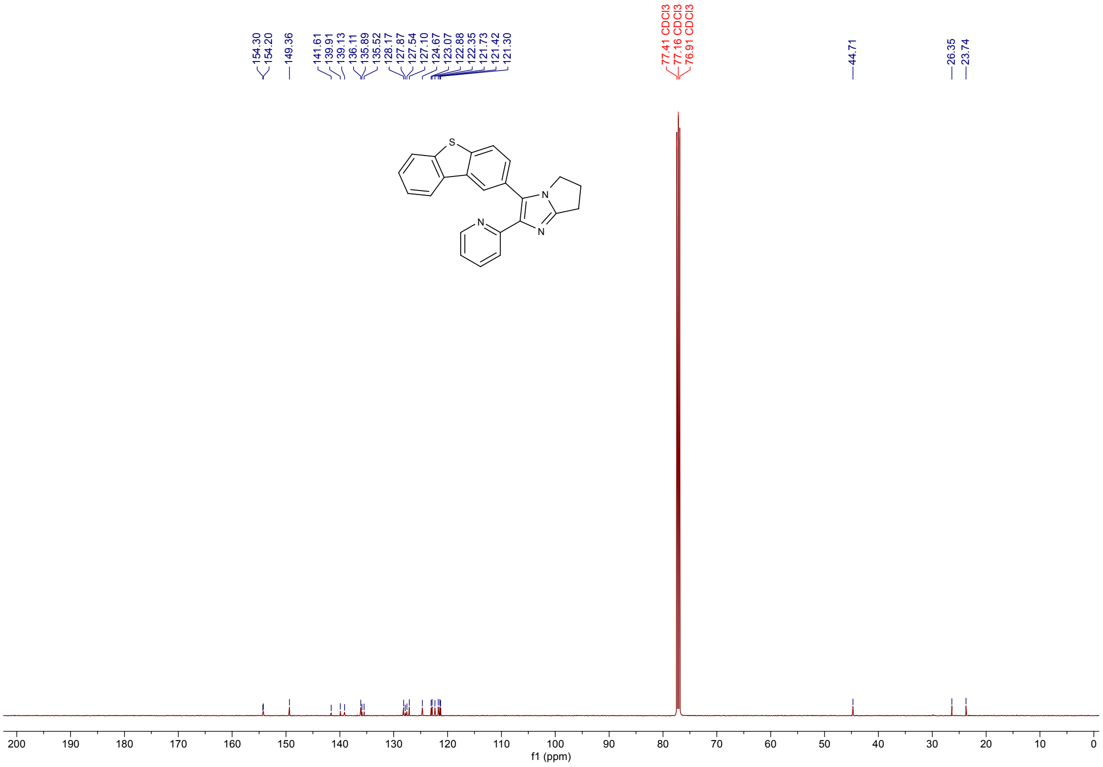

3-(Benzo[*b*]thiophen-3-yl)-2-(pyridin-2-yl)-6,7-dihydro-5*H*-pyrrolo[1,2-*a*]imidazole (OSA_000832)

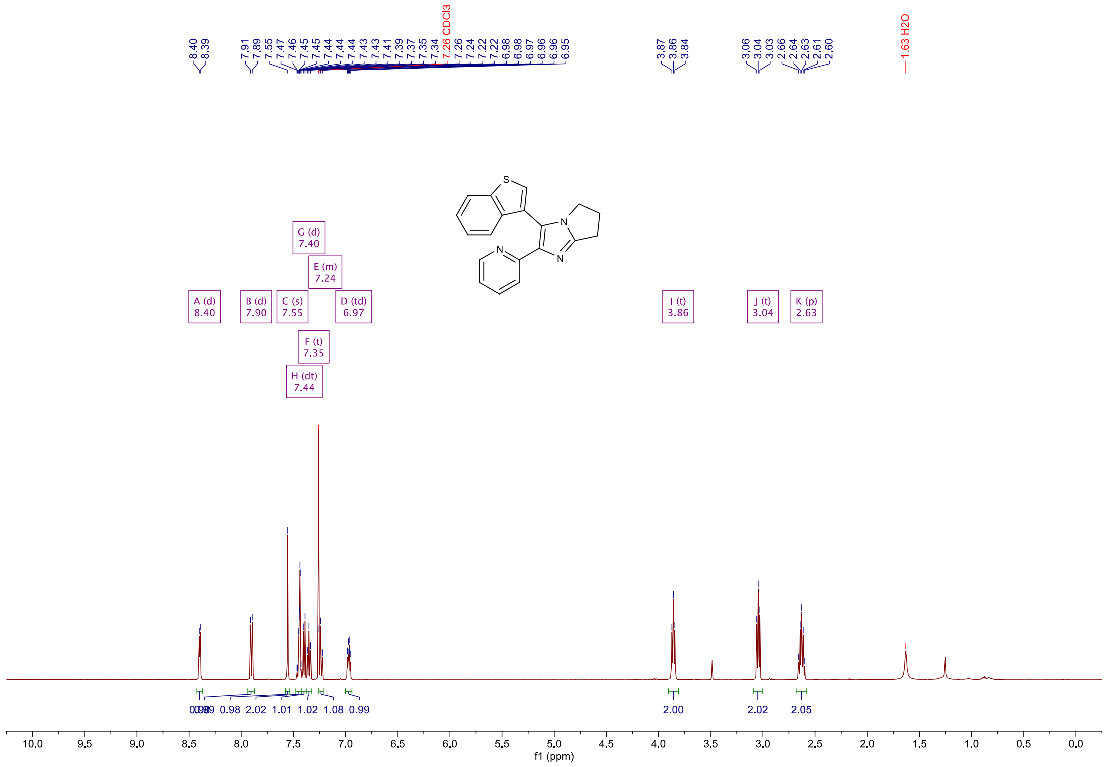

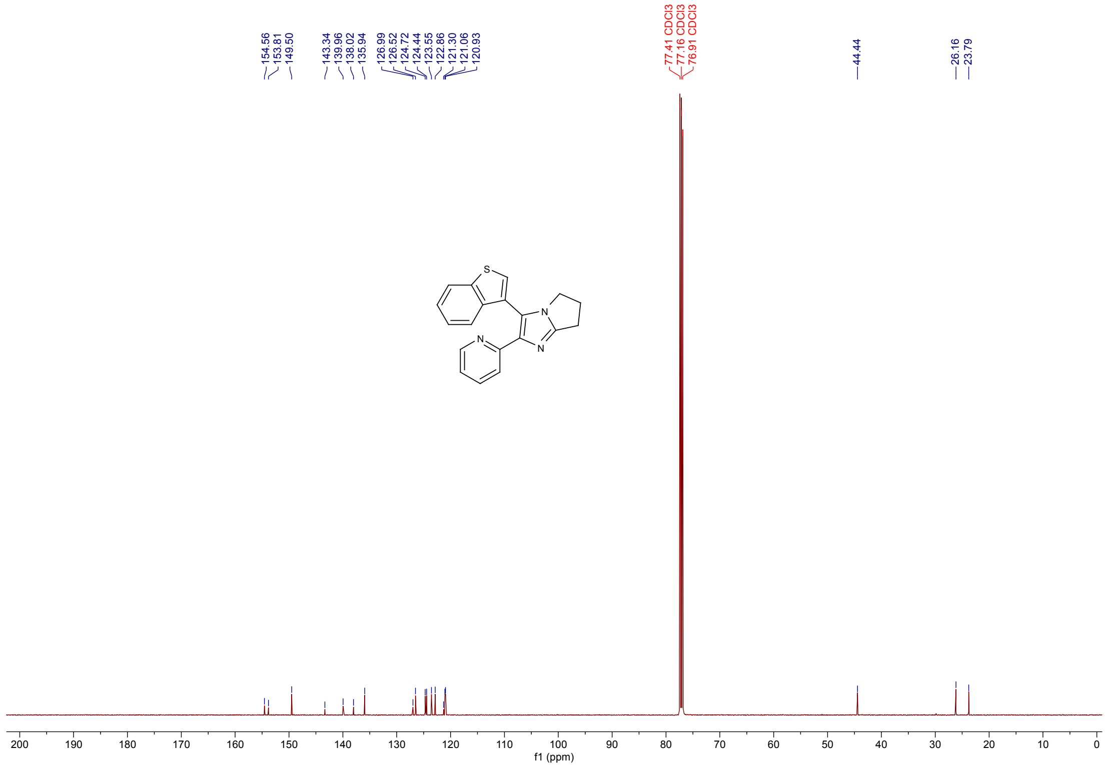

3-(4-Methoxyphenyl)-2-(pyridin-2-yl)-6,7-dihydro-5*H*-pyrrolo[1,2-*a*]imidazole (OSA_000823)

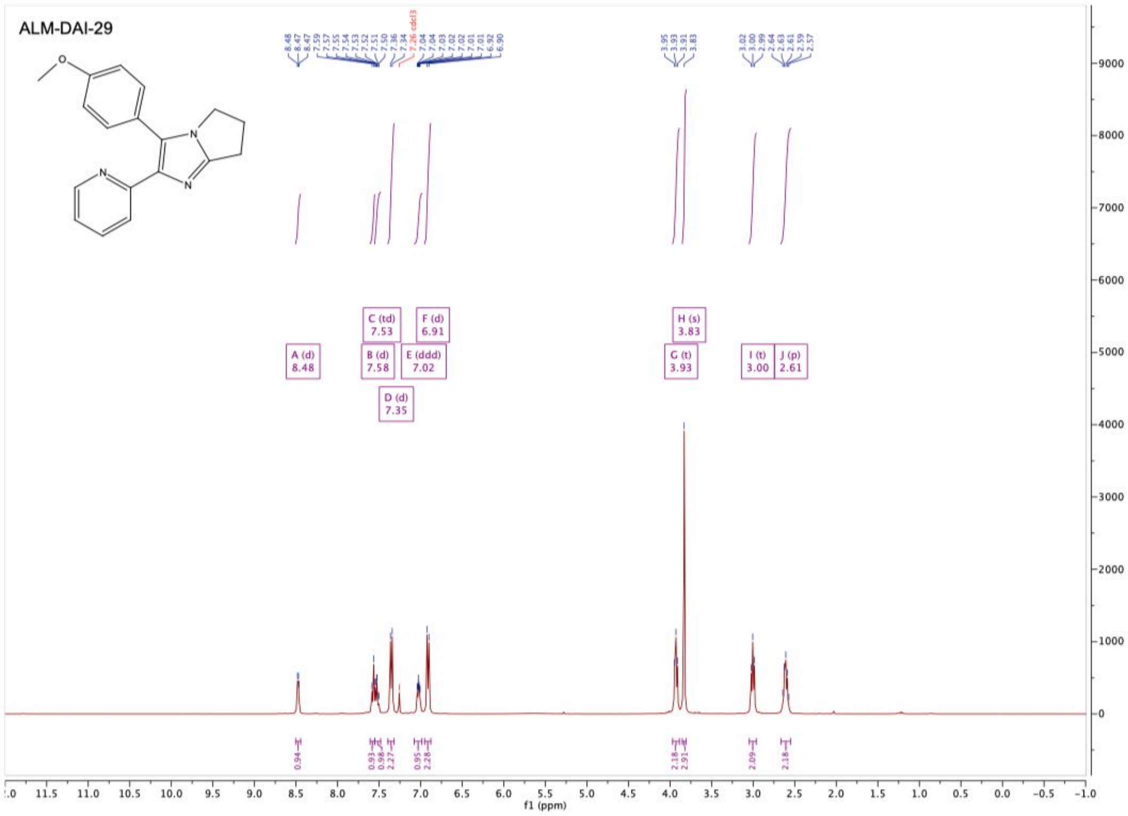

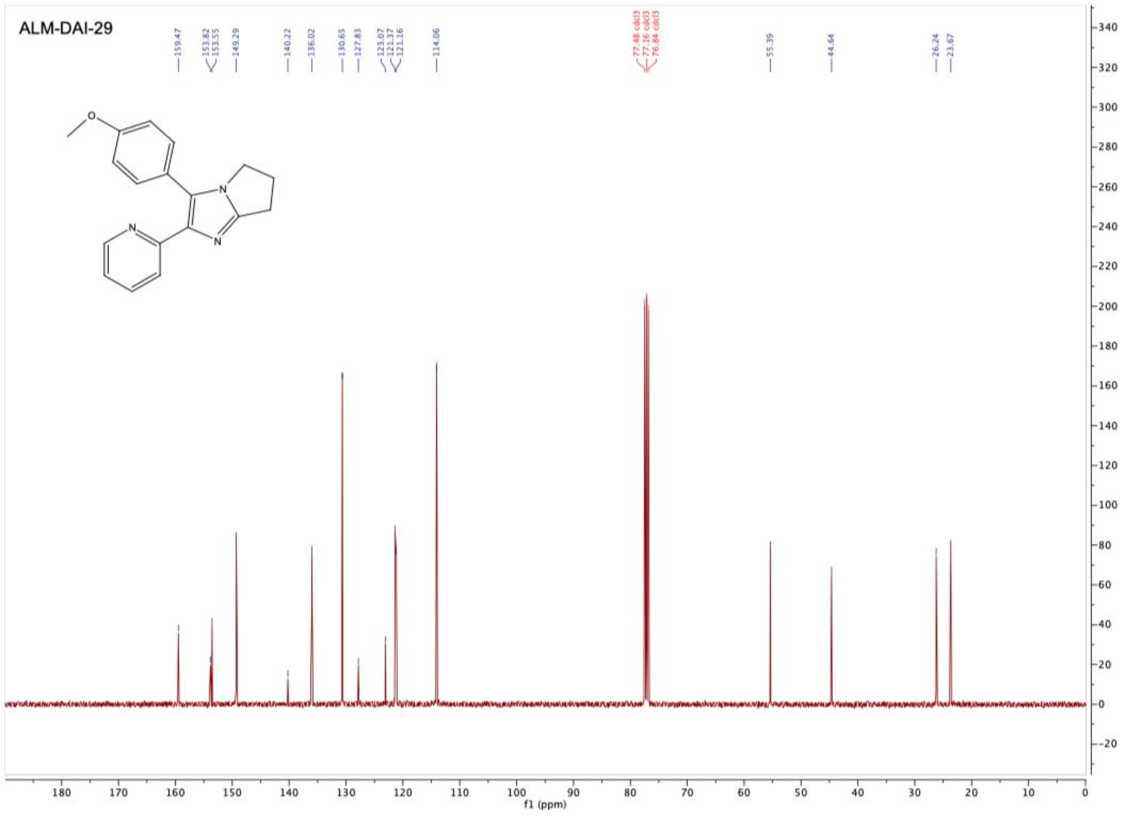

2-(Pyridin-2-yl)-3-(4-(trifluoromethoxy)phenyl)-6,7-dihydro-5*H*-pyrrolo[1,2-*a*]imidazole (OSA_000868)

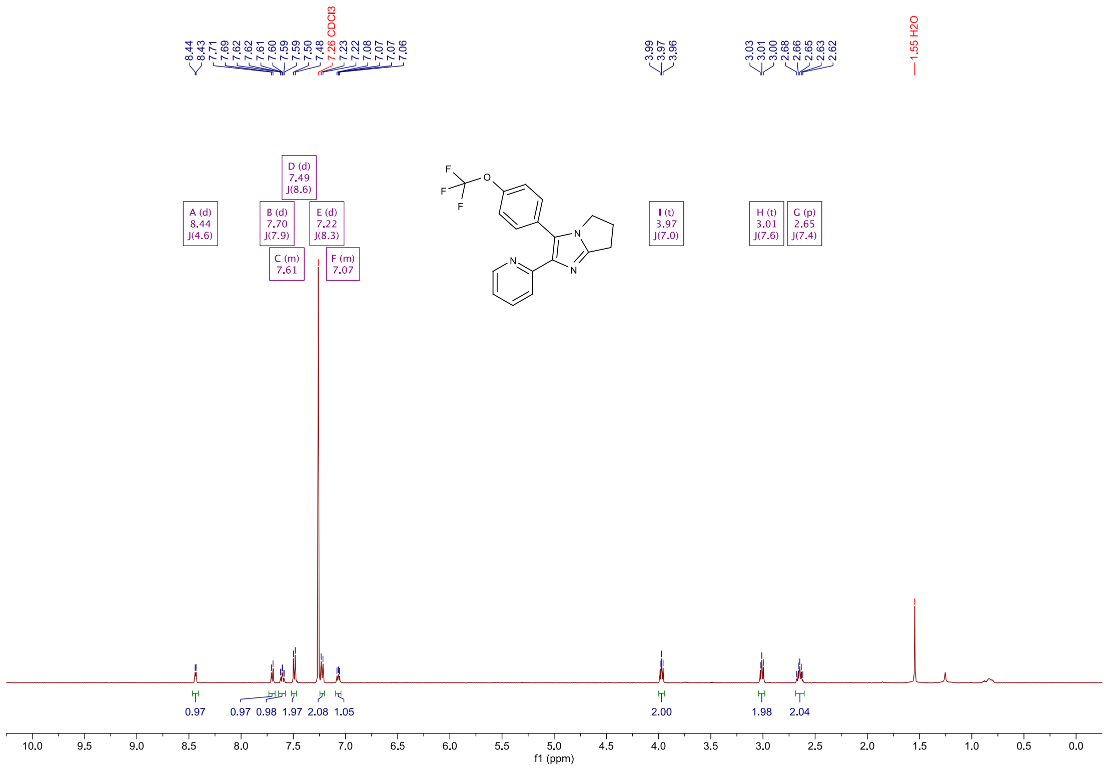

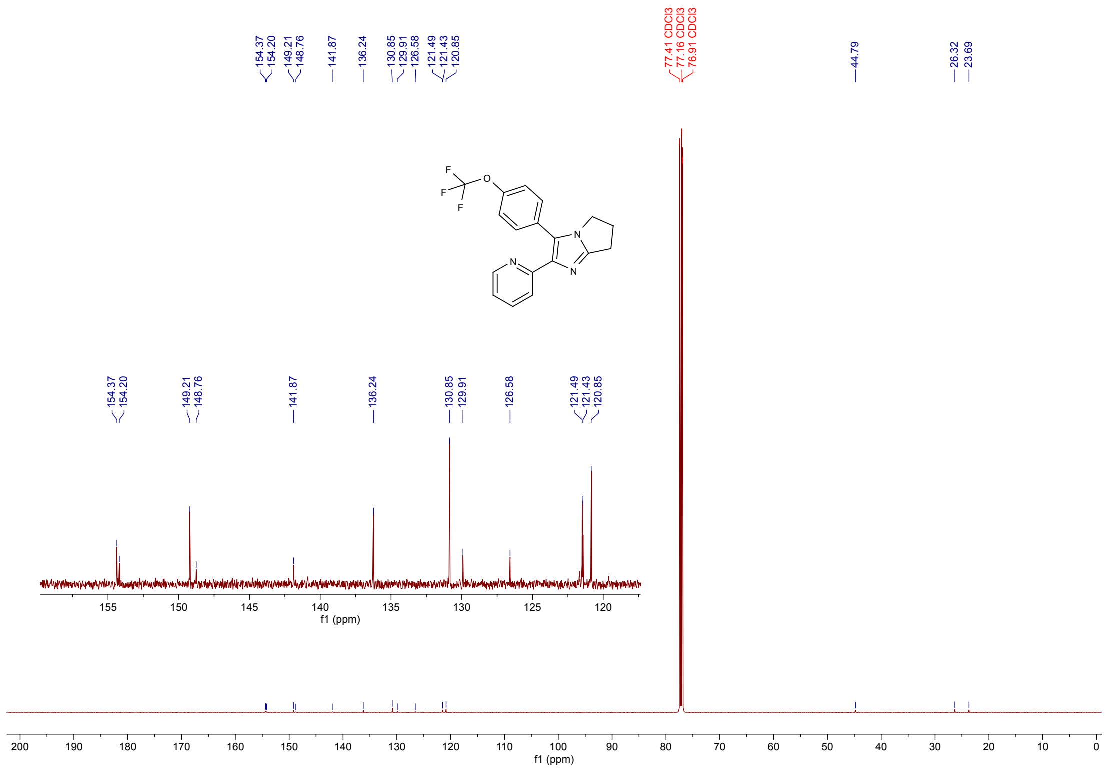

2-(Pyridin-2-yl)-3-(4-(trifluoromethyl)phenyl)-6,7-dihydro-5*H*-pyrrolo[1,2-*a*]imidazole (OSA_001052)

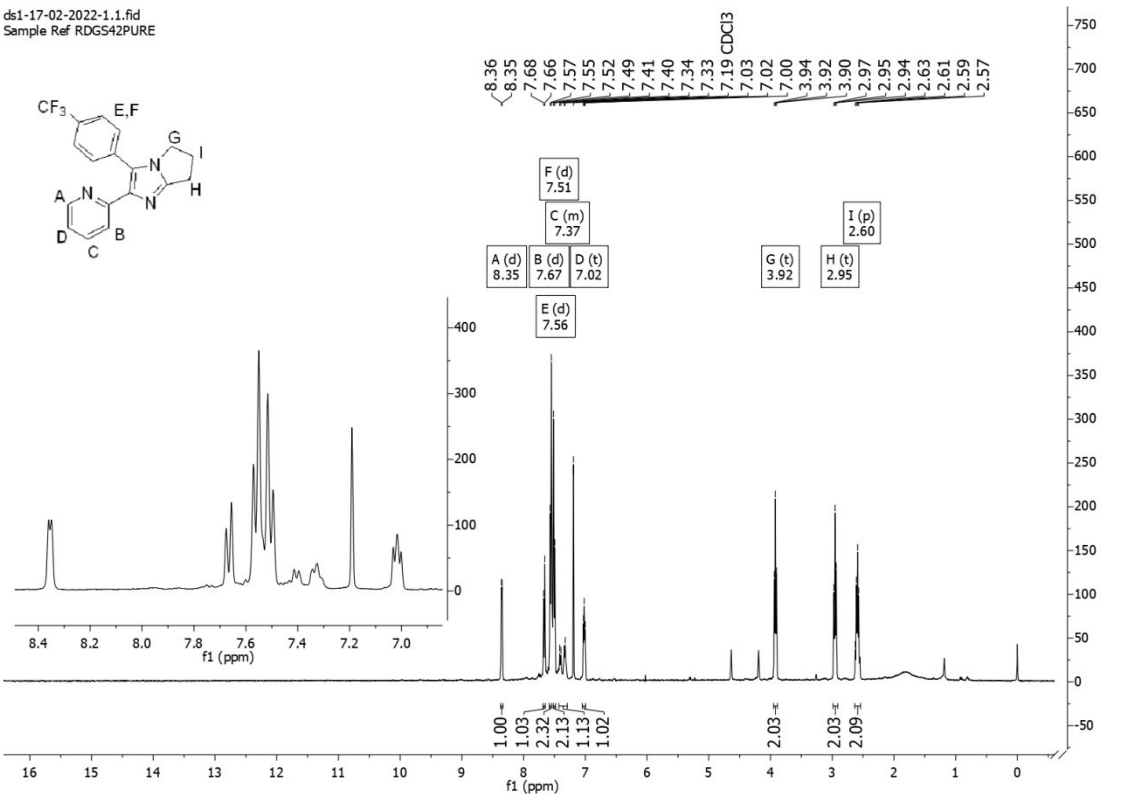

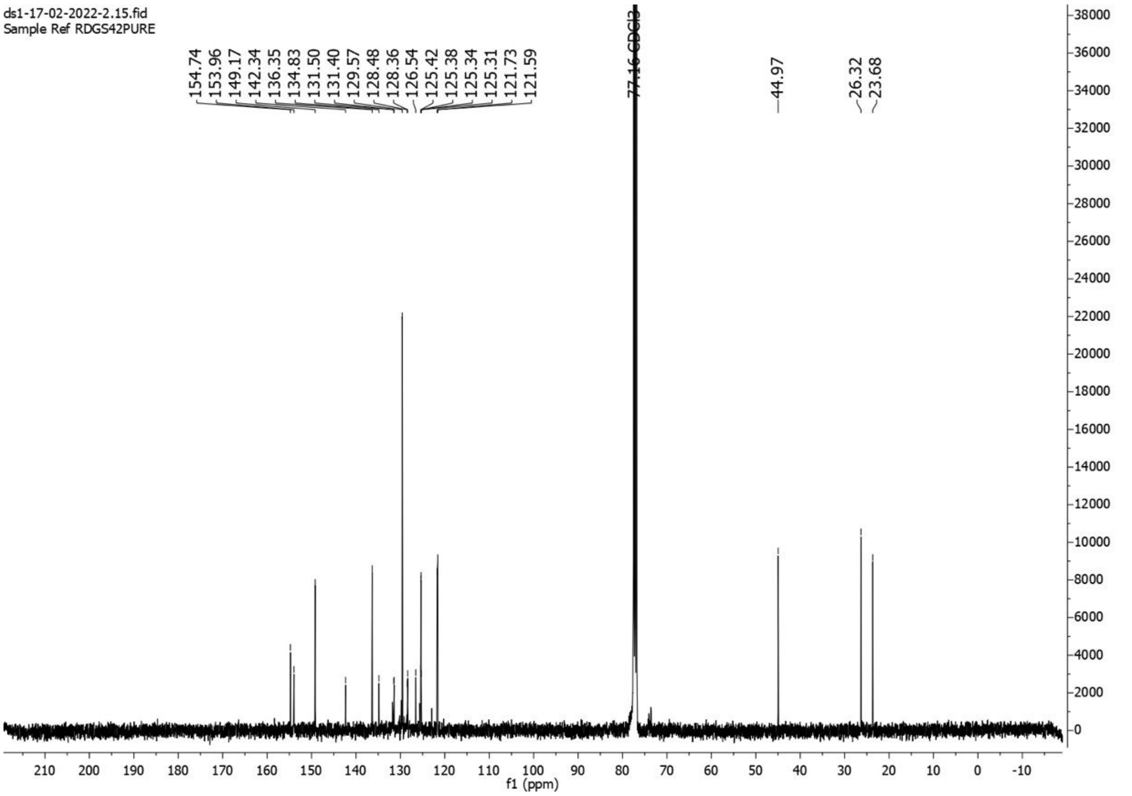

3-(3-Methoxyphenyl)-2-(pyridin-2-yl)-6,7-dihydro-5*H*-pyrrolo[1,2-*a*]imidazole

(OSA_000825)

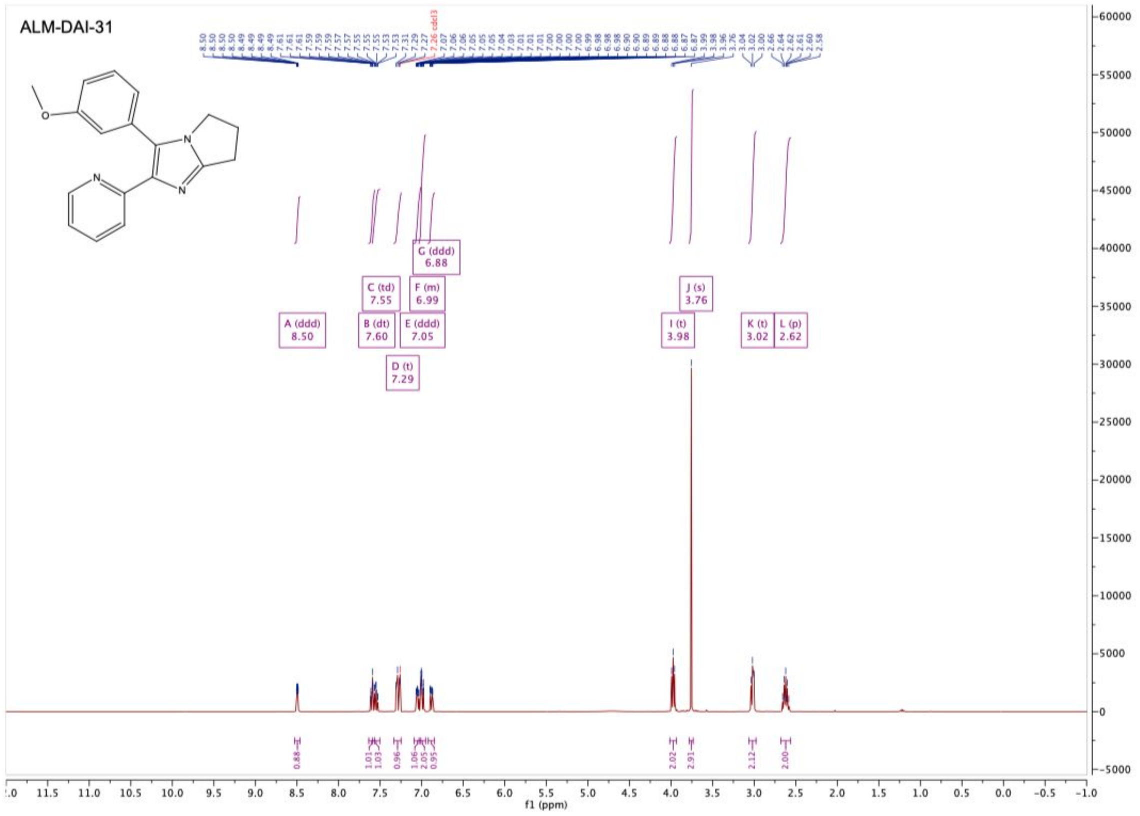

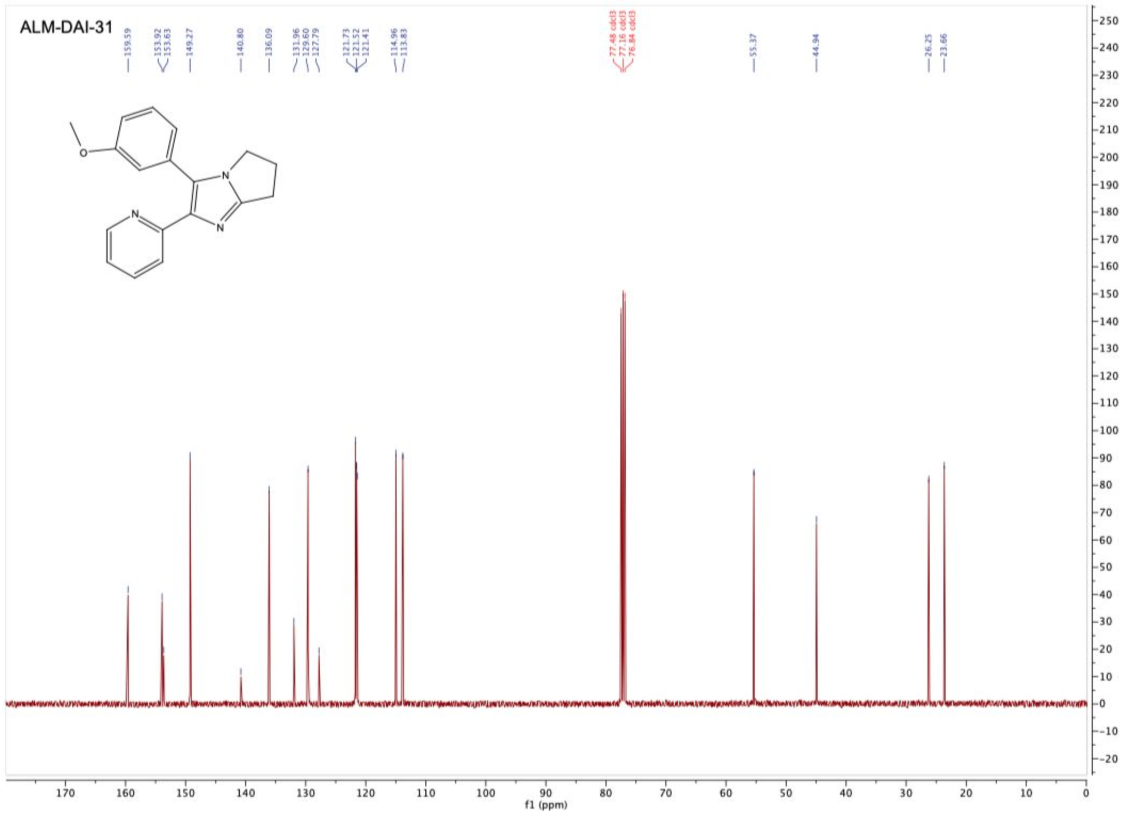

2-(Pyridin-2-yl)-3-(*m*-tolyl)-6,7-dihydro-5*H*-pyrrolo[1,2-*a*]imidazole (OSA_000976)

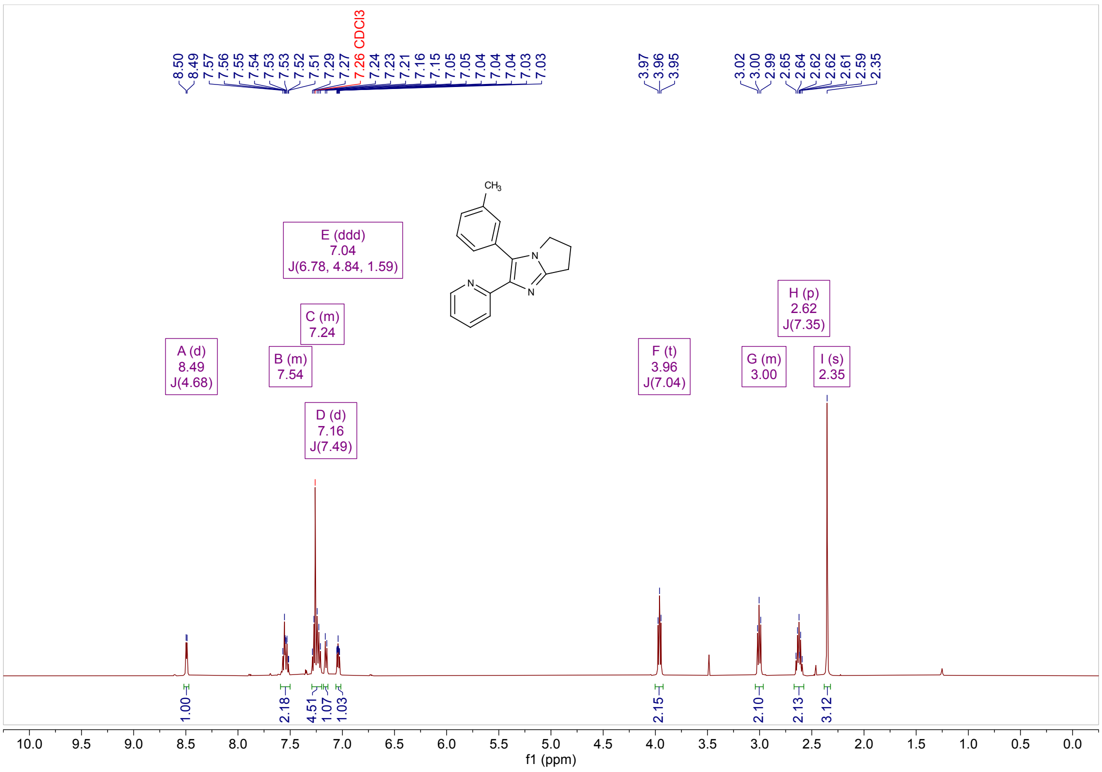

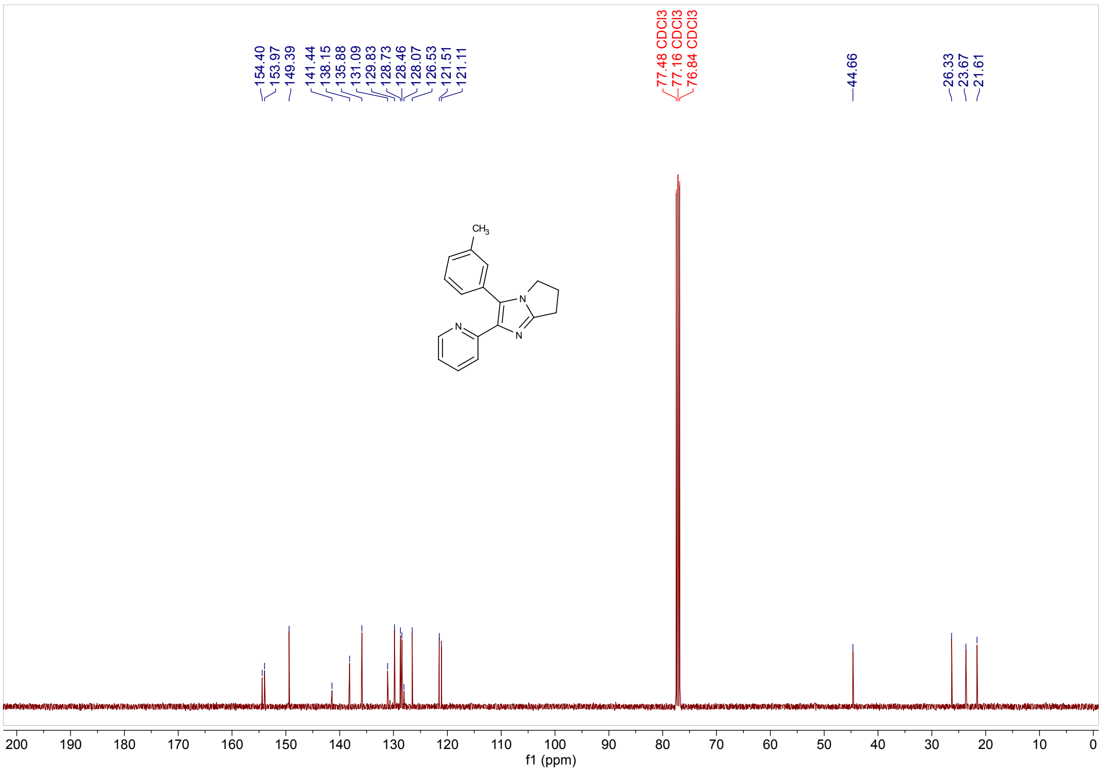

3-(3,4-Dichlorophenyl)-2-(pyridin-2-yl)-6,7-dihydro-5*H*-pyrrolo[1,2-*a*]imidazole

(OSA_000876)

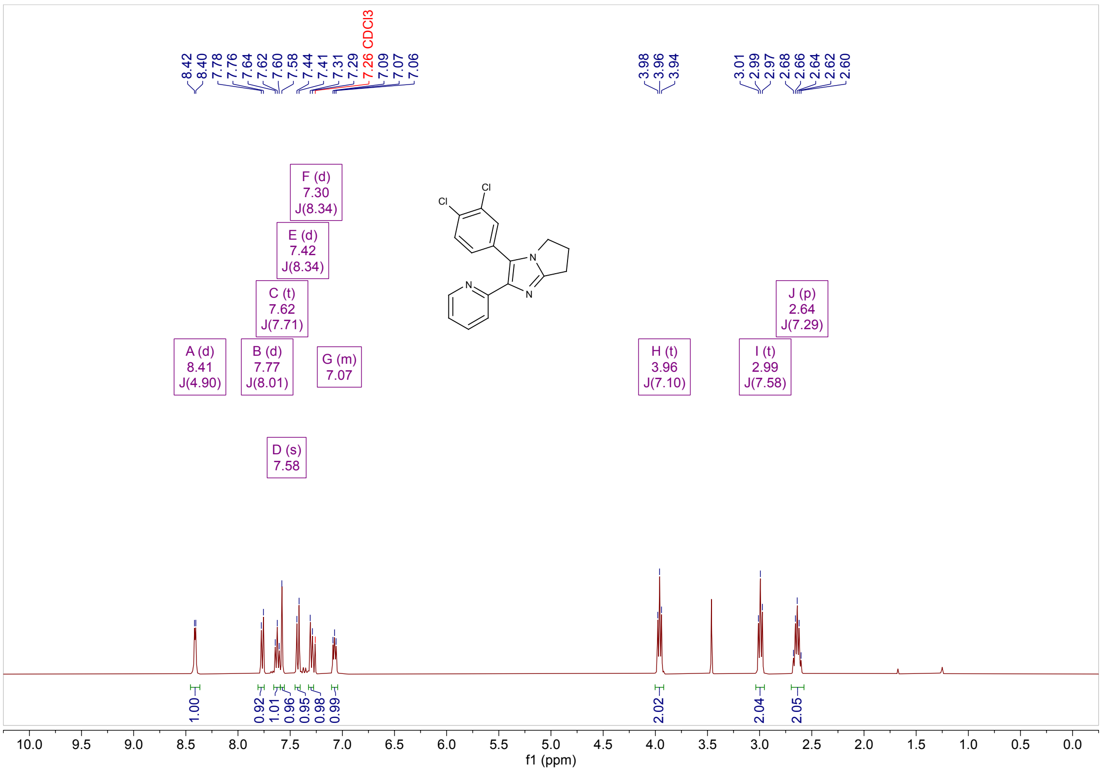

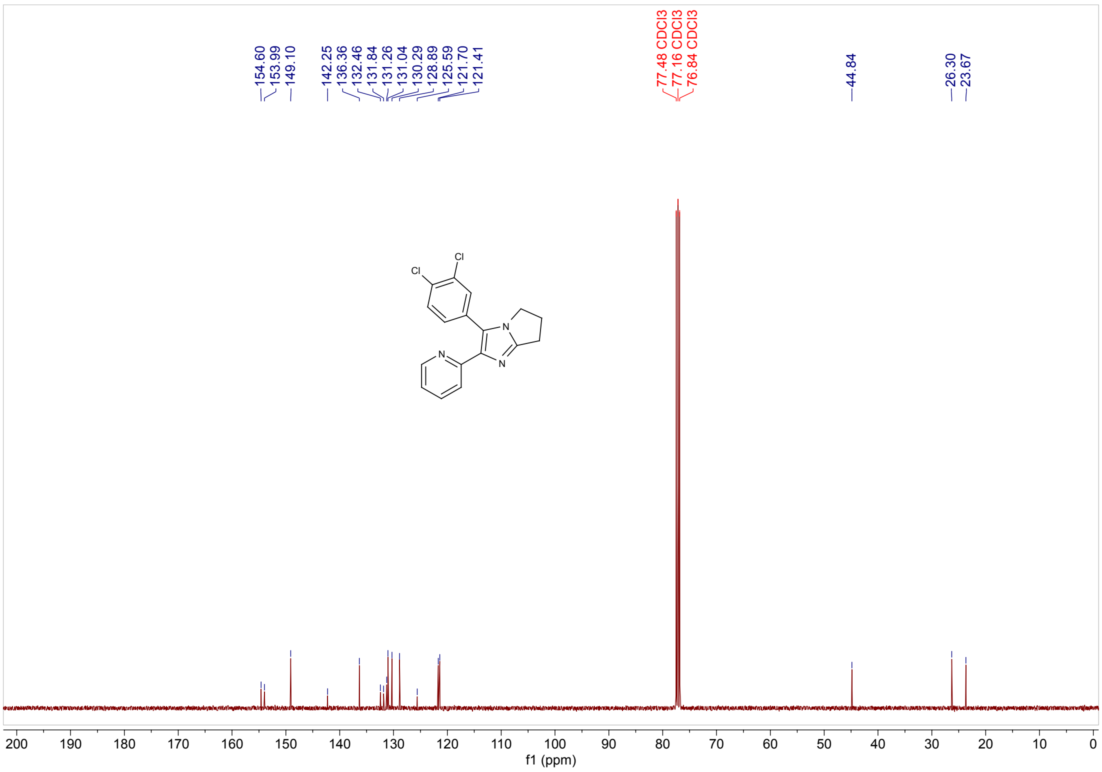

3-(Benzo[*d*][1,3]dioxol-5-yl)-2-(pyridin-2-yl)-6,7-dihydro-5*H*-pyrrolo[1,2-*a*]imidazole (OSA_000812)

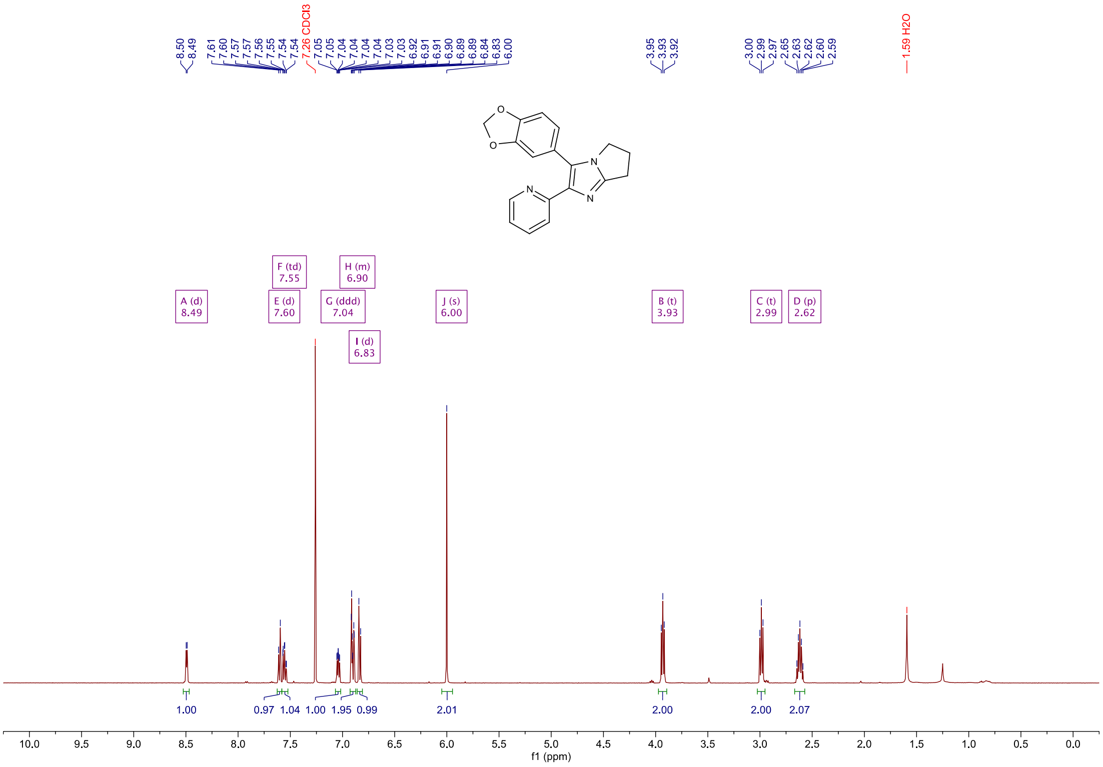

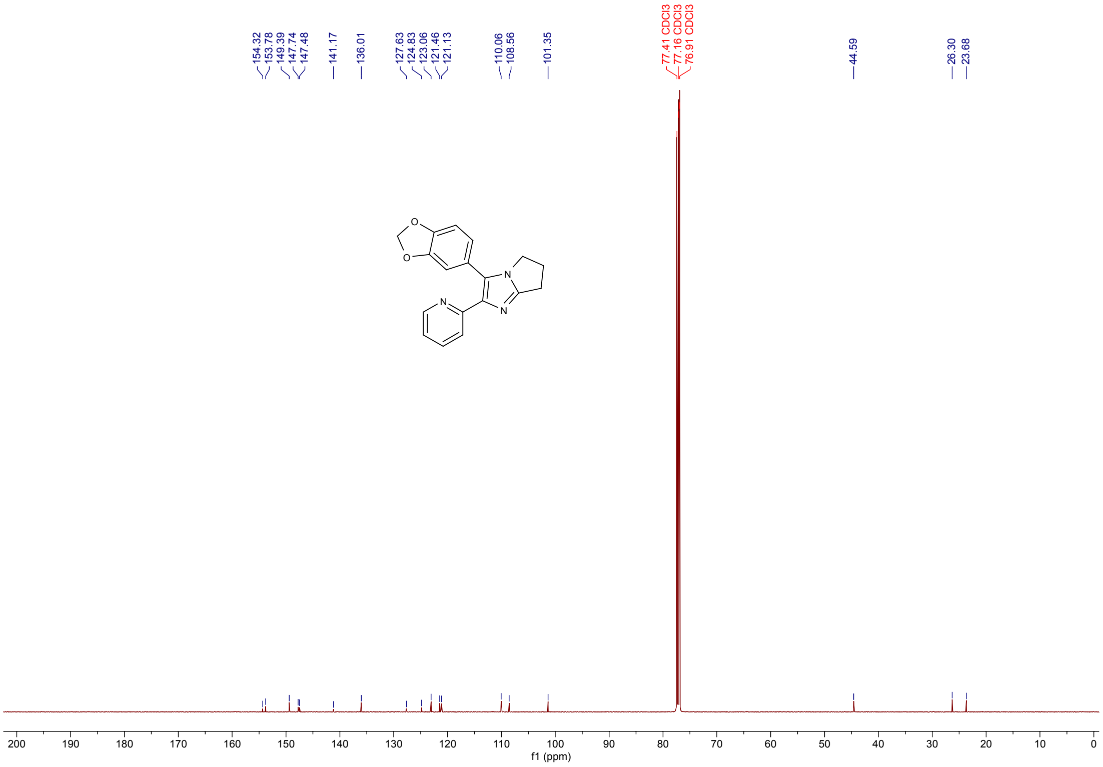

3-(2,3-Dihydrobenzofuran-5-yl)-2-(pyridin-2-yl)-6,7-dihydro-5*H*-pyrrolo[1,2-*a*]imidazole (OSA_000827)

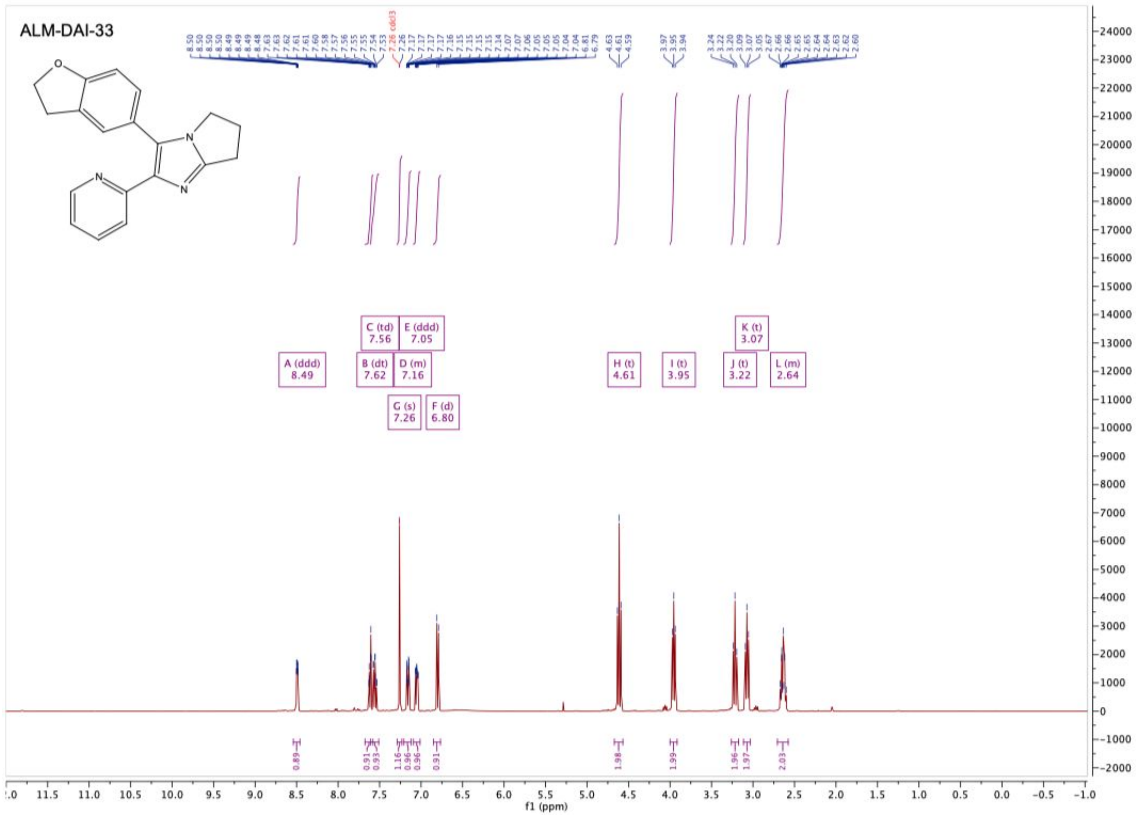

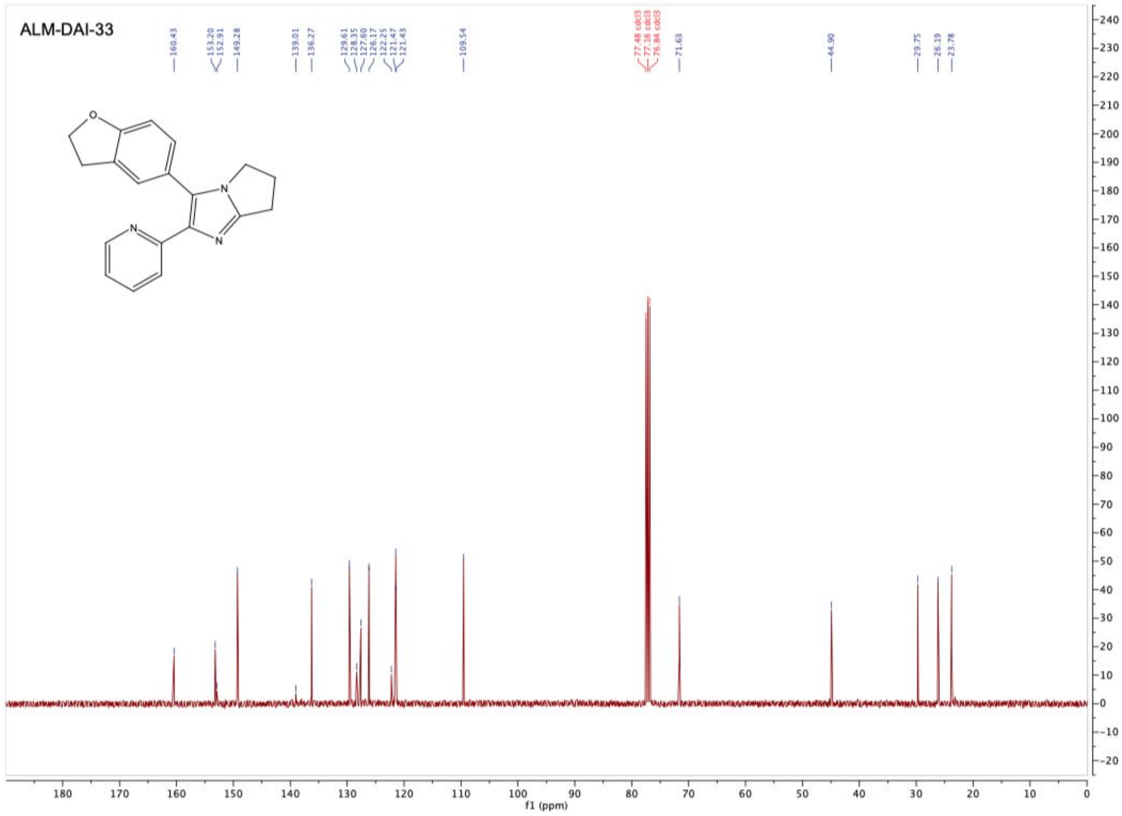

3-(2,3-Dihydrobenzofuran-6-yl)-2-(pyridin-2-yl)-6,7-dihydro-5*H*-pyrrolo[1,2-*a*]imidazole (OSA_000828)

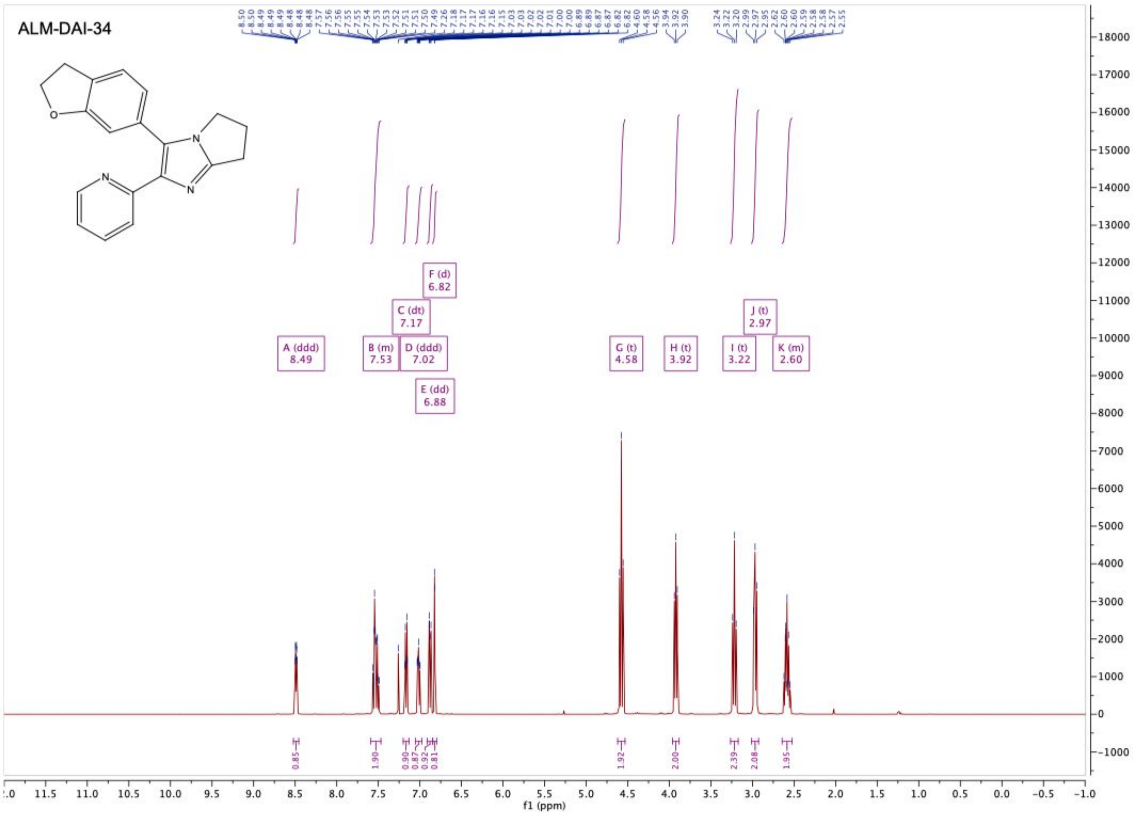

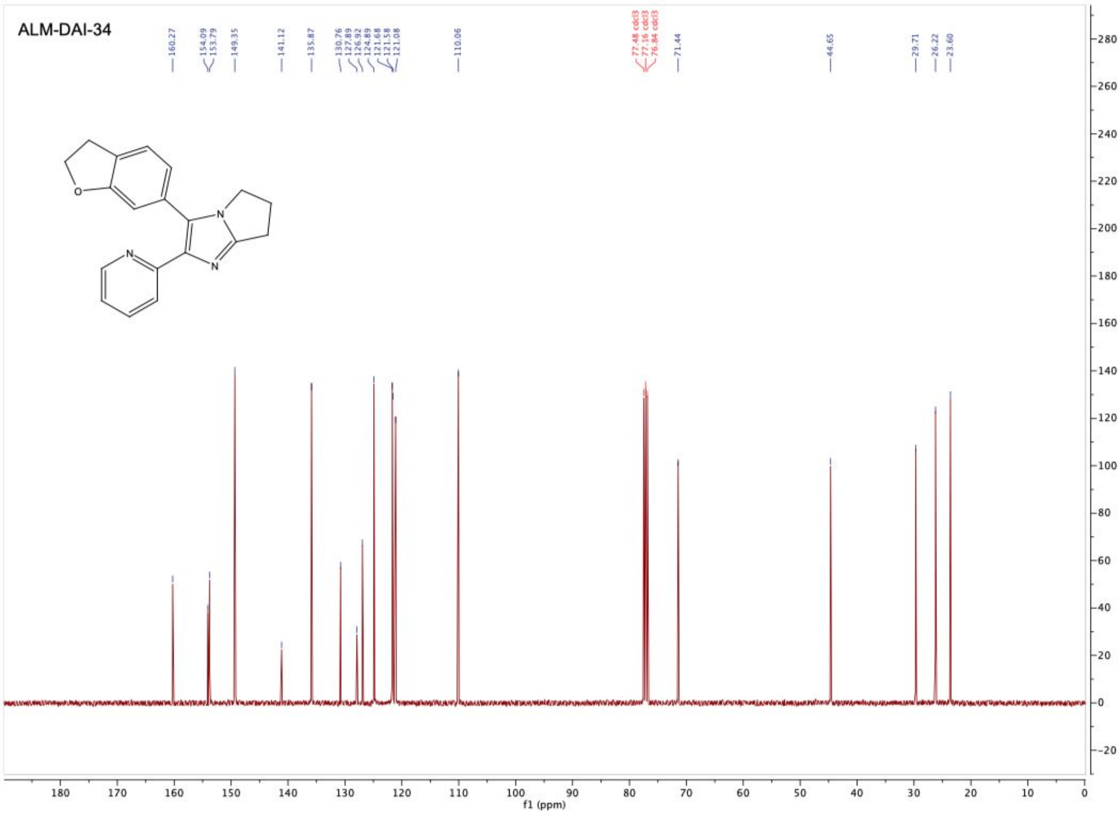

2-Methyl-5-(2-(pyridin-2-yl)-6,7-dihydro-5*H*-pyrrolo[1,2-*a*]imidazol-3-yl)benzo[*d*]thiazole (OSA_000836)

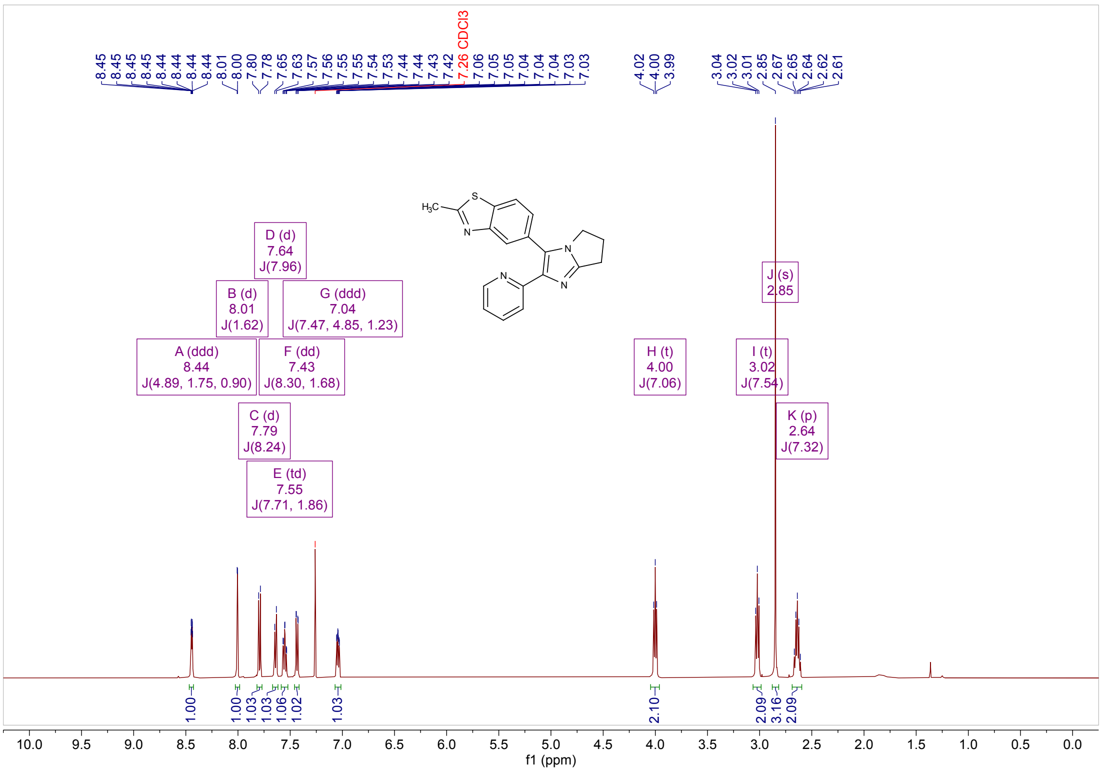

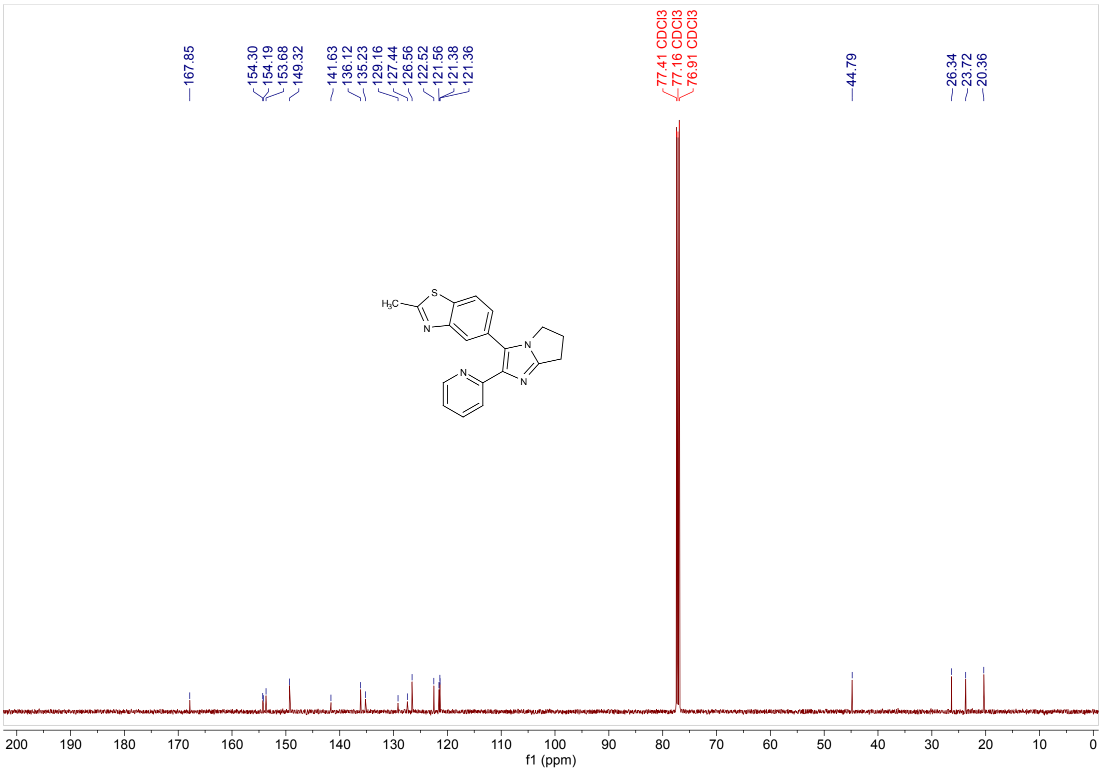

7-(2-(Pyridin-2-yl)-6,7-dihydro-5*H*-pyrrolo[1,2-*a*]imidazol-3-yl)quinoline (OSA_000833)

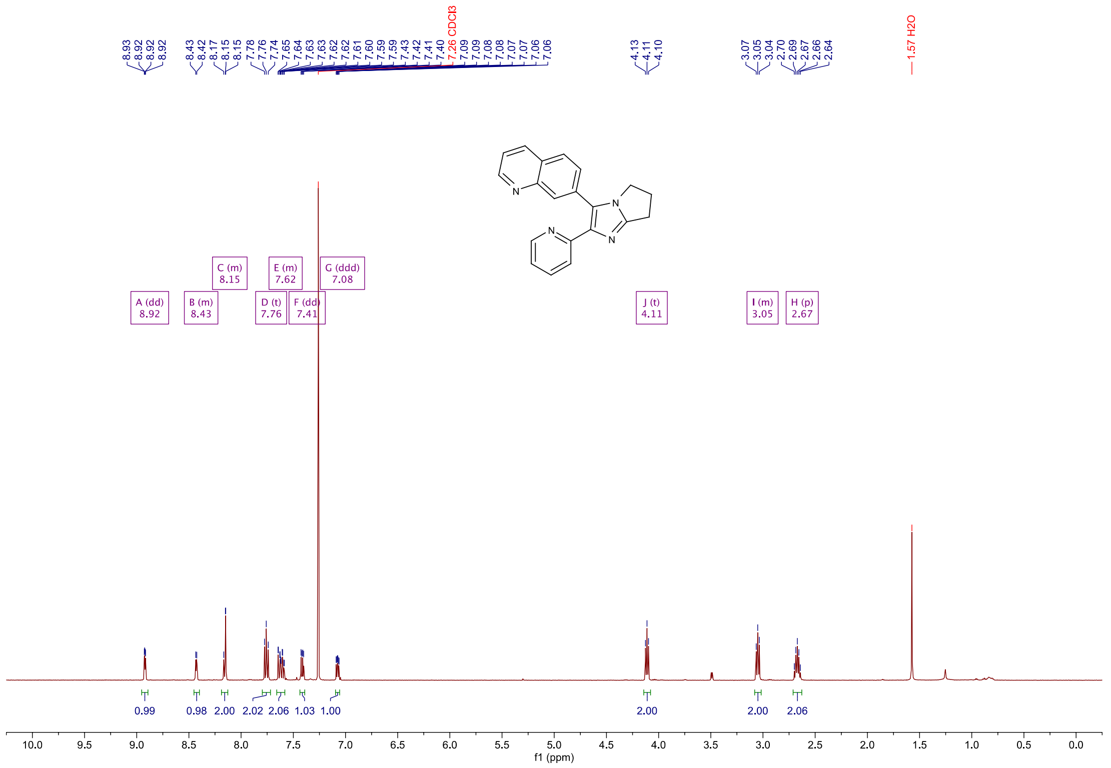

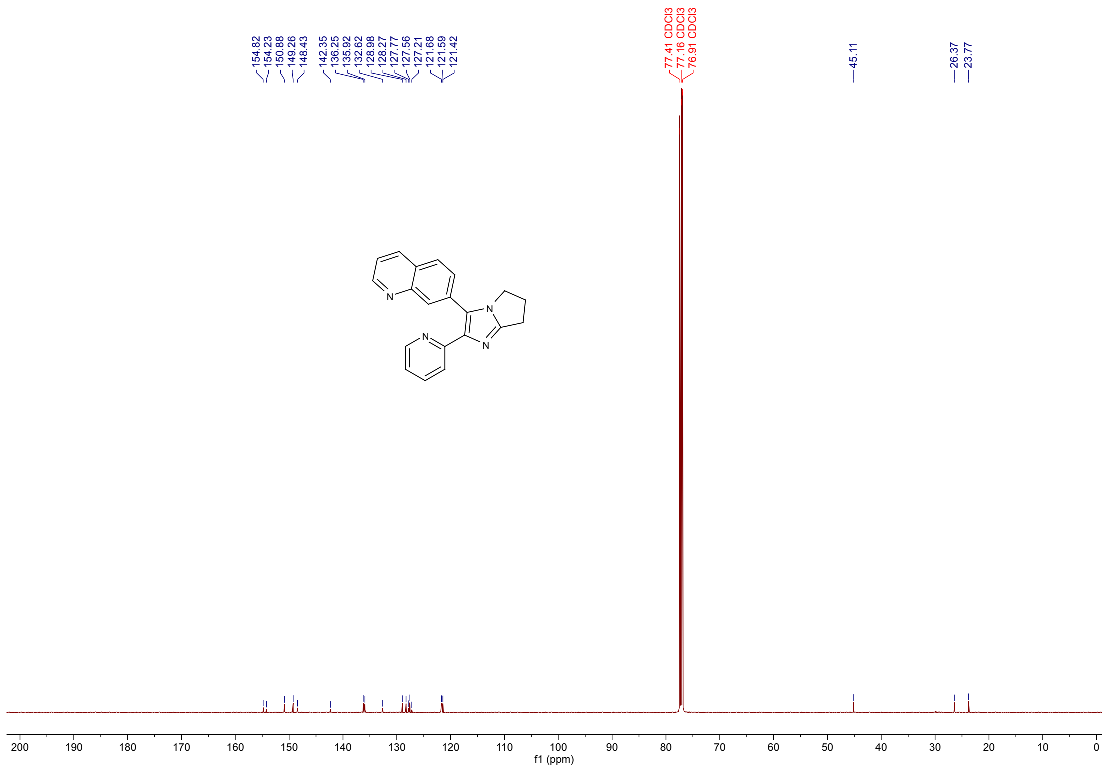

5-(2-(Pyridin-2-yl)-6,7-dihydro-5*H*-pyrrolo[1,2-*a*]imidazol-3-yl)benzo[*d*]thiazole (OSA_000835)

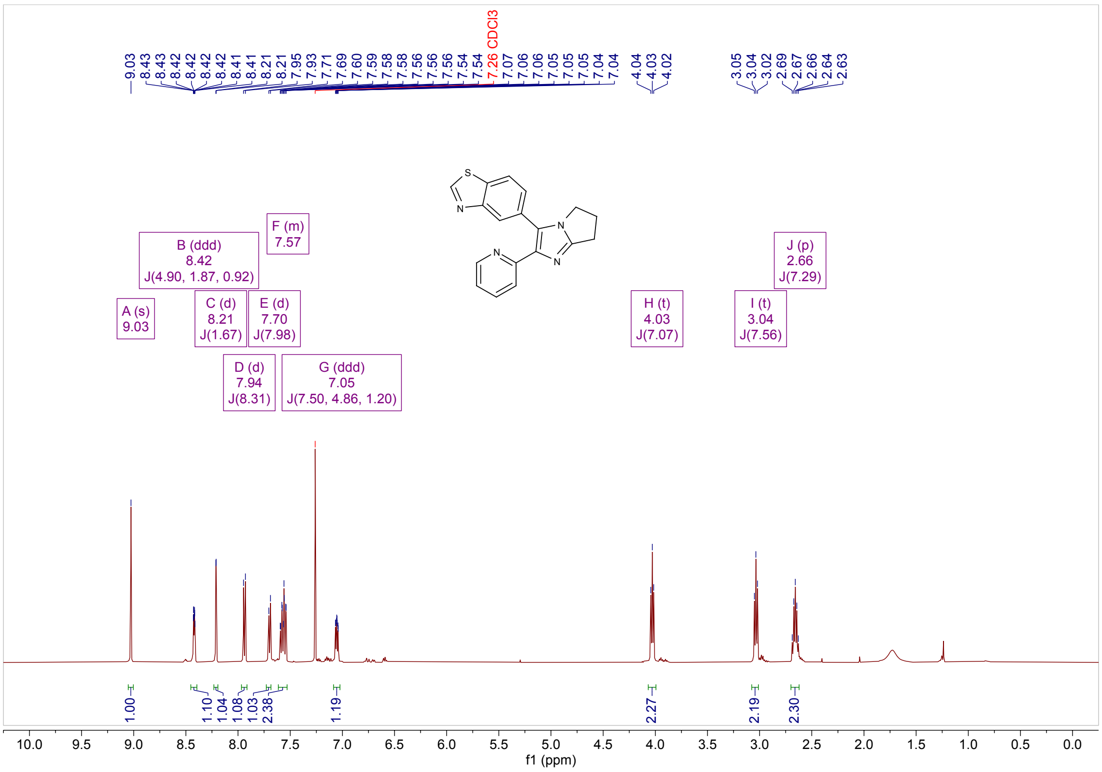

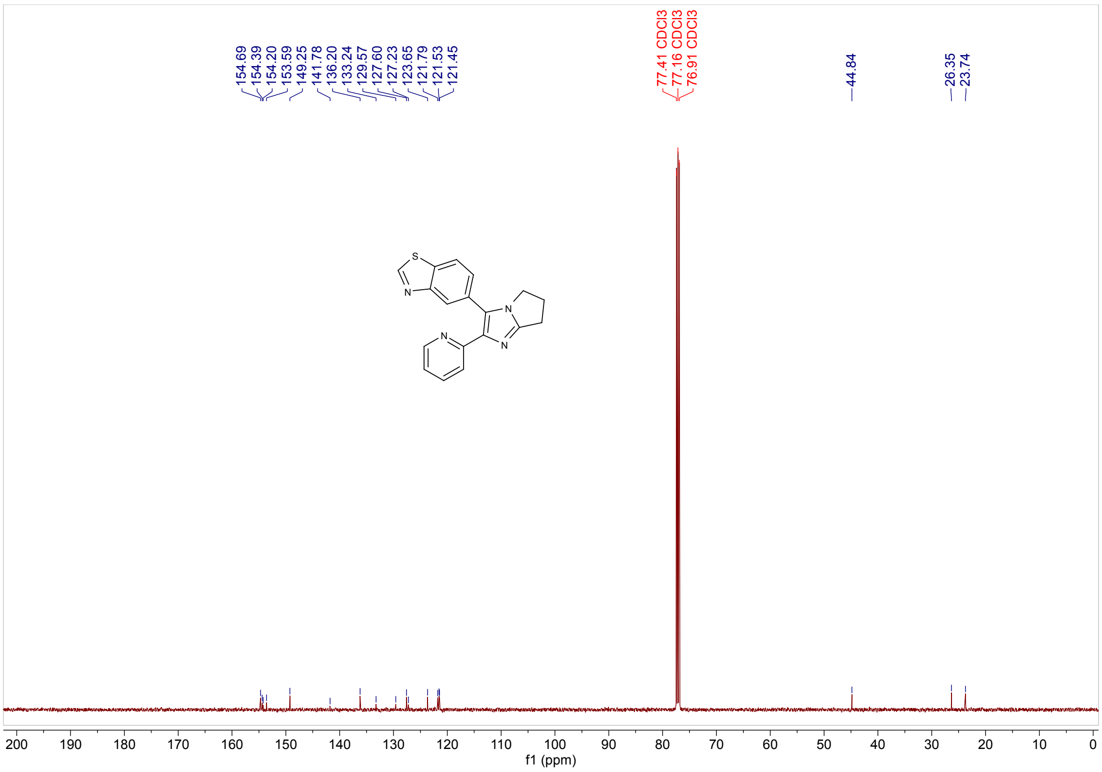

3-Phenyl-2-(pyridin-2-yl)-6,7-dihydro-5*H*-pyrrolo[1,2-*a*]imidazole (OSA_000870)

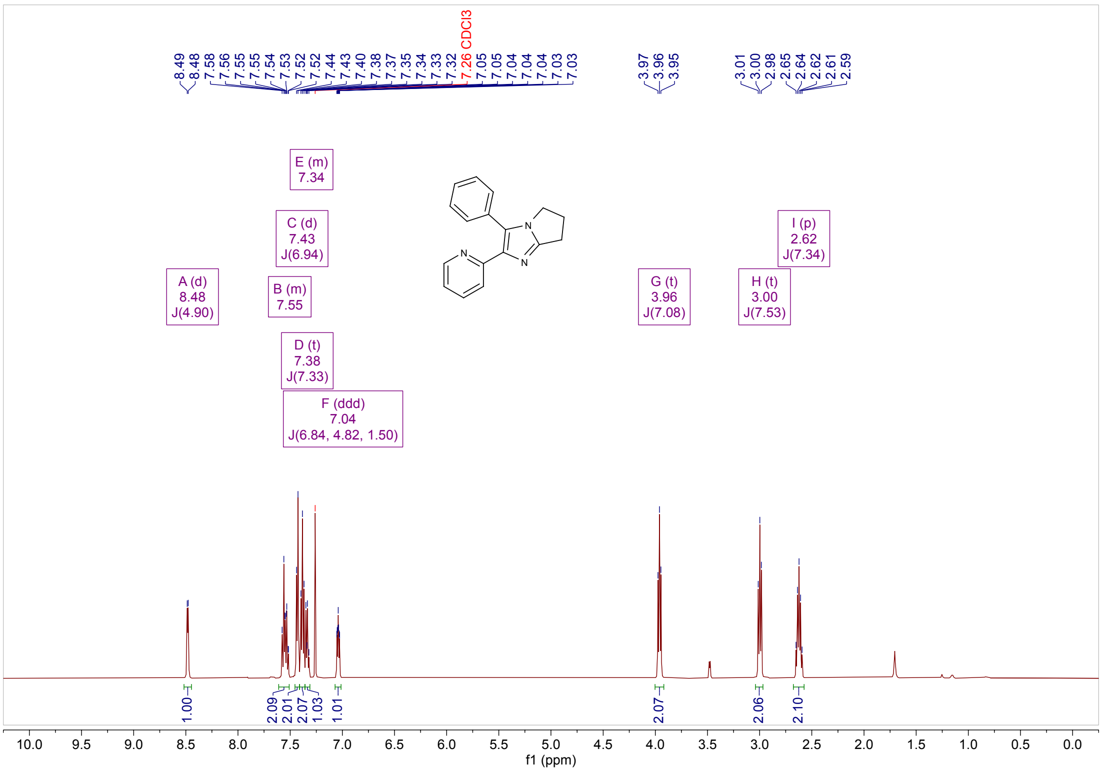

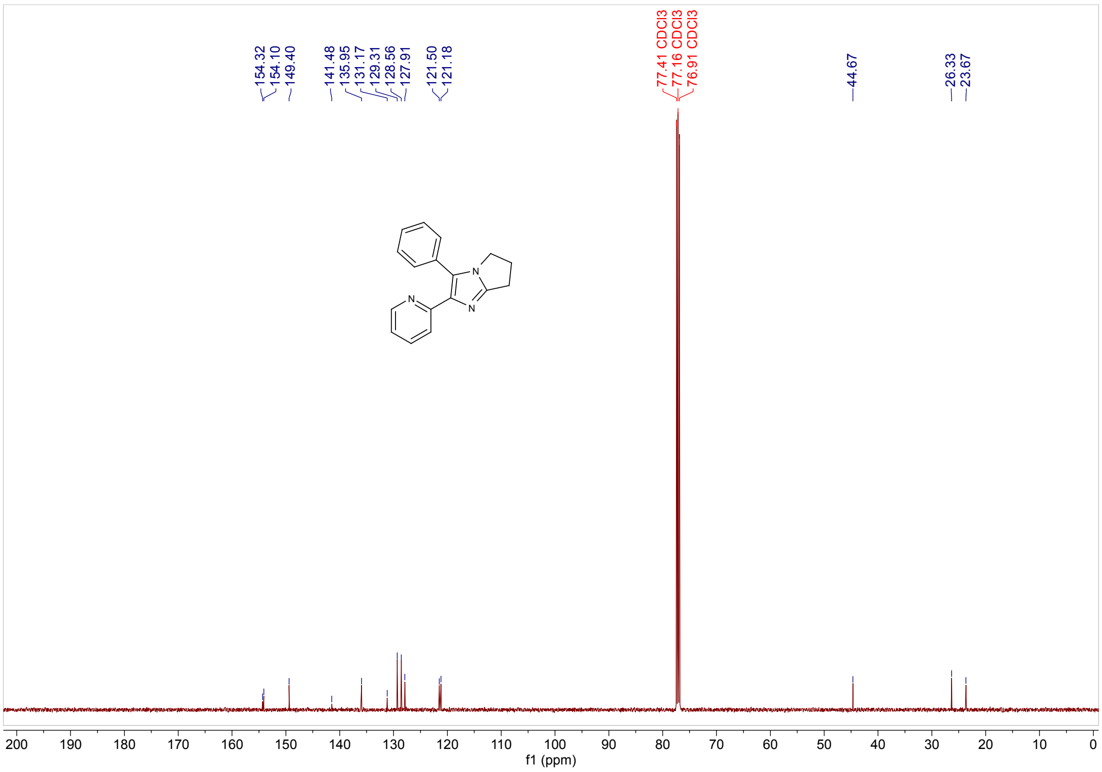

3-(4-(Methylsulfinyl)phenyl)-2-(pyridin-2-yl)-6,7-dihydro-5*H*-pyrrolo[1,2-*a*]imidazole (OSA_000838)

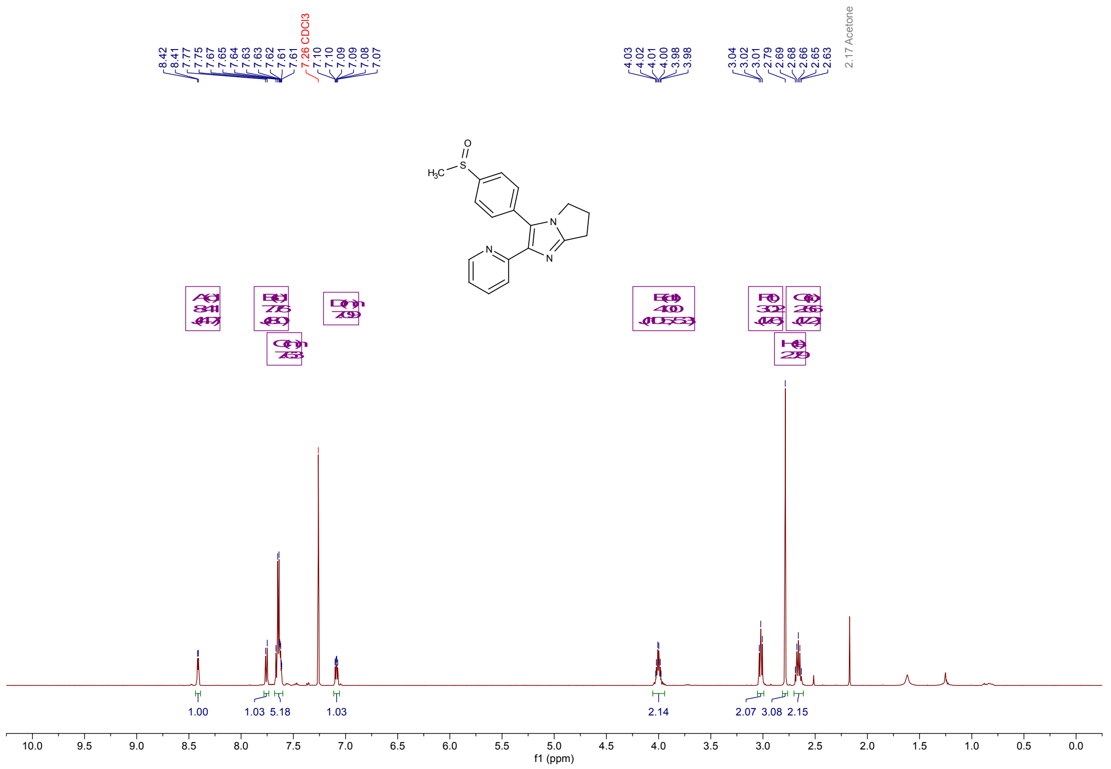

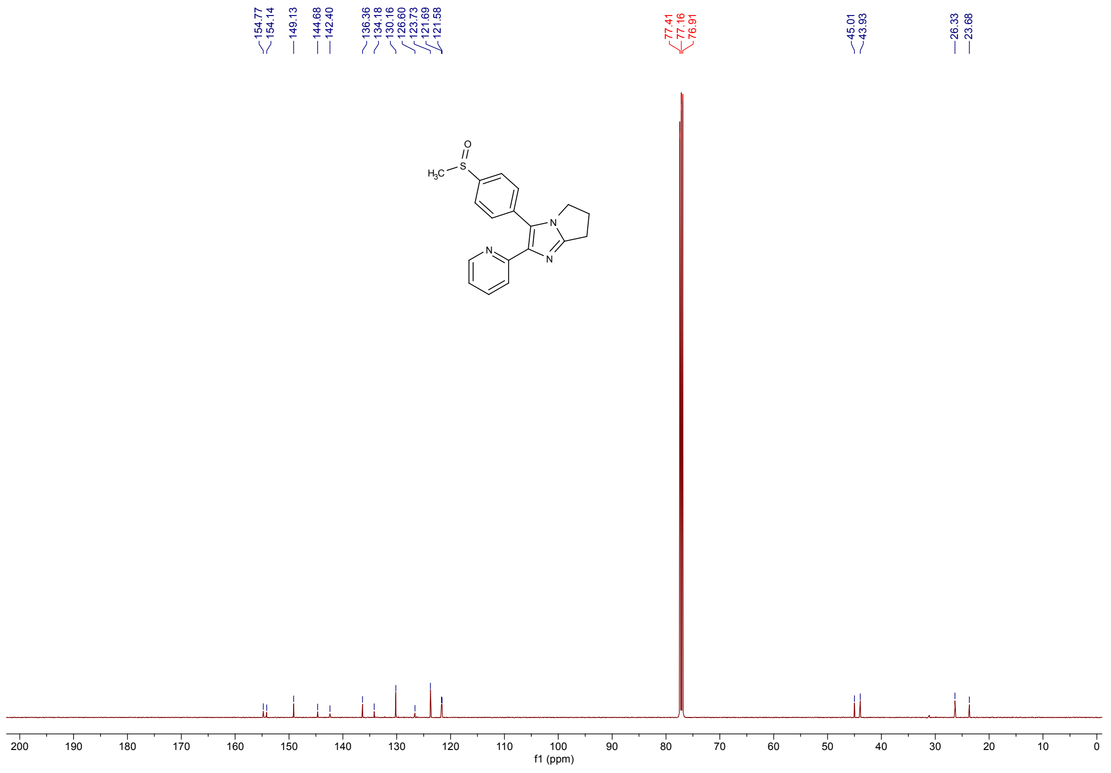

1-(4-(2-(Pyridin-2-yl)-6,7-dihydro-5*H*-pyrrolo[1,2-*a*]imidazol-3-yl)phenyl)ethan-1-one (OSA_000973)

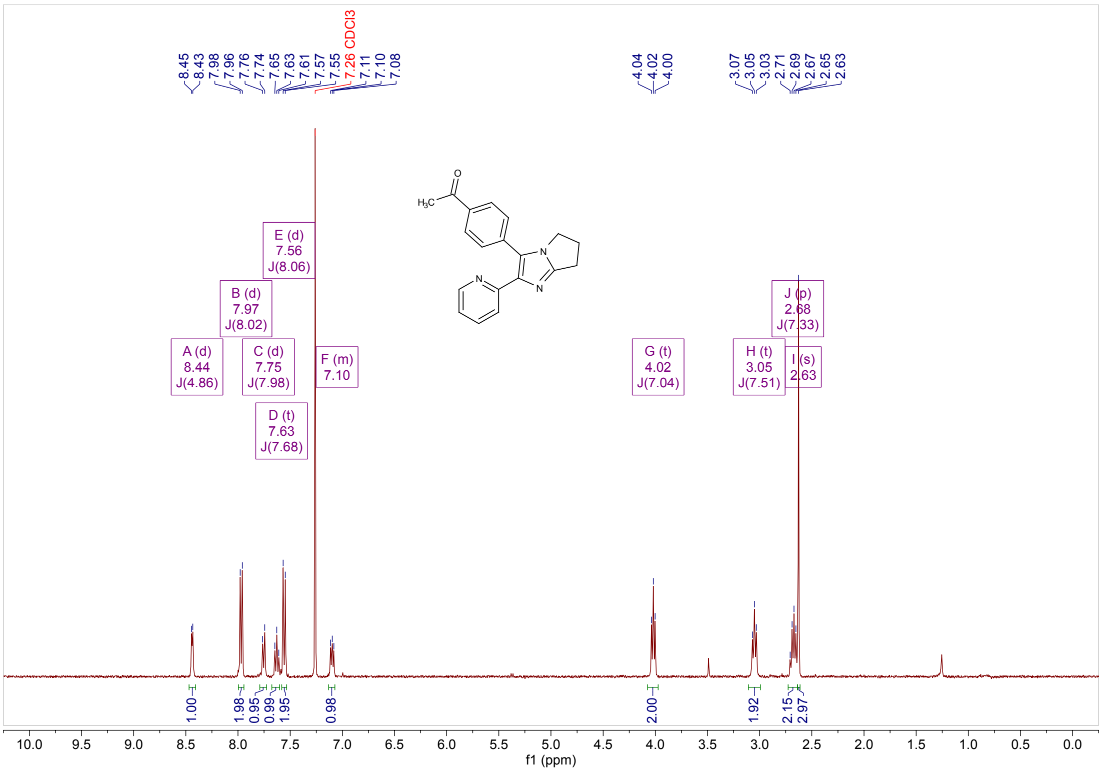

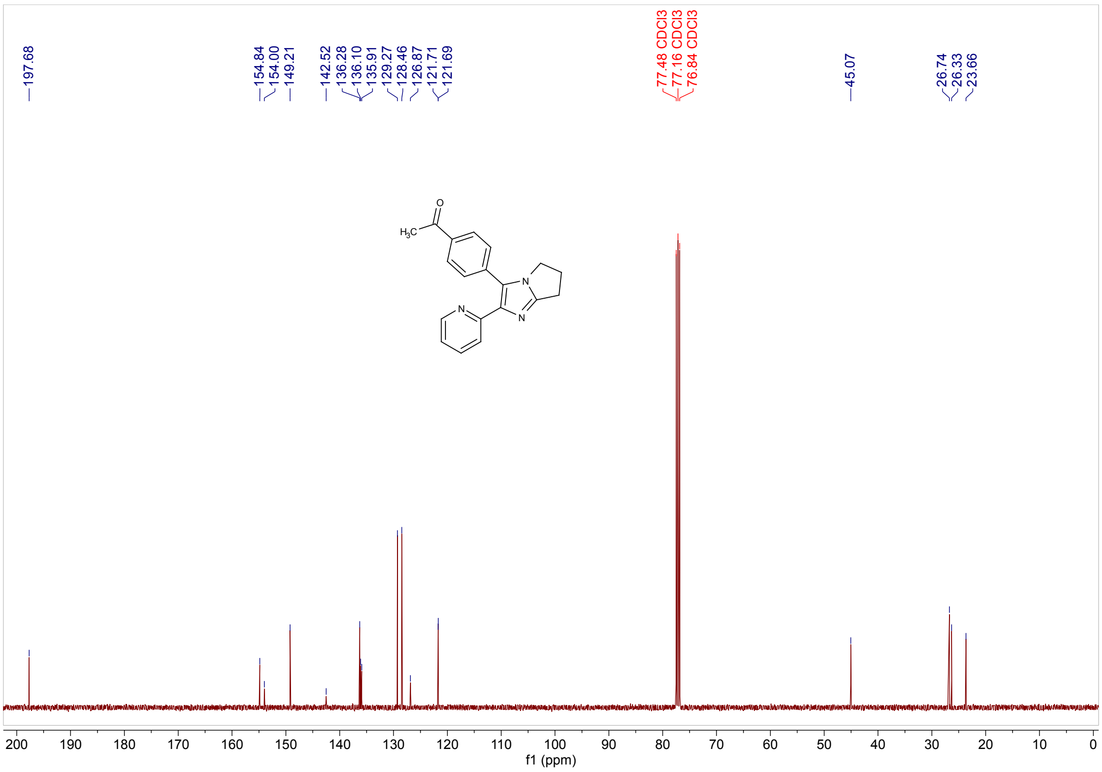

3-(4-(Methylsulfonyl)phenyl)-2-(pyridin-2-yl)-6,7-dihydro-5*H*-pyrrolo[1,2-*a*]imidazole (OSA_000837)

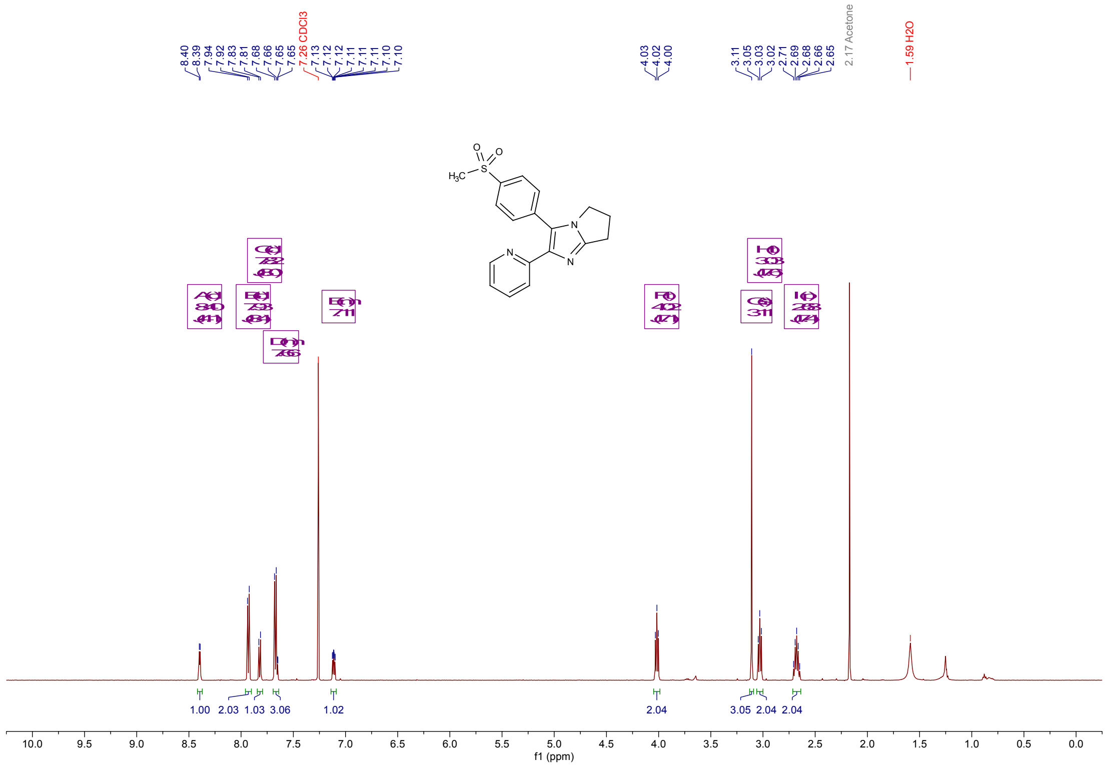

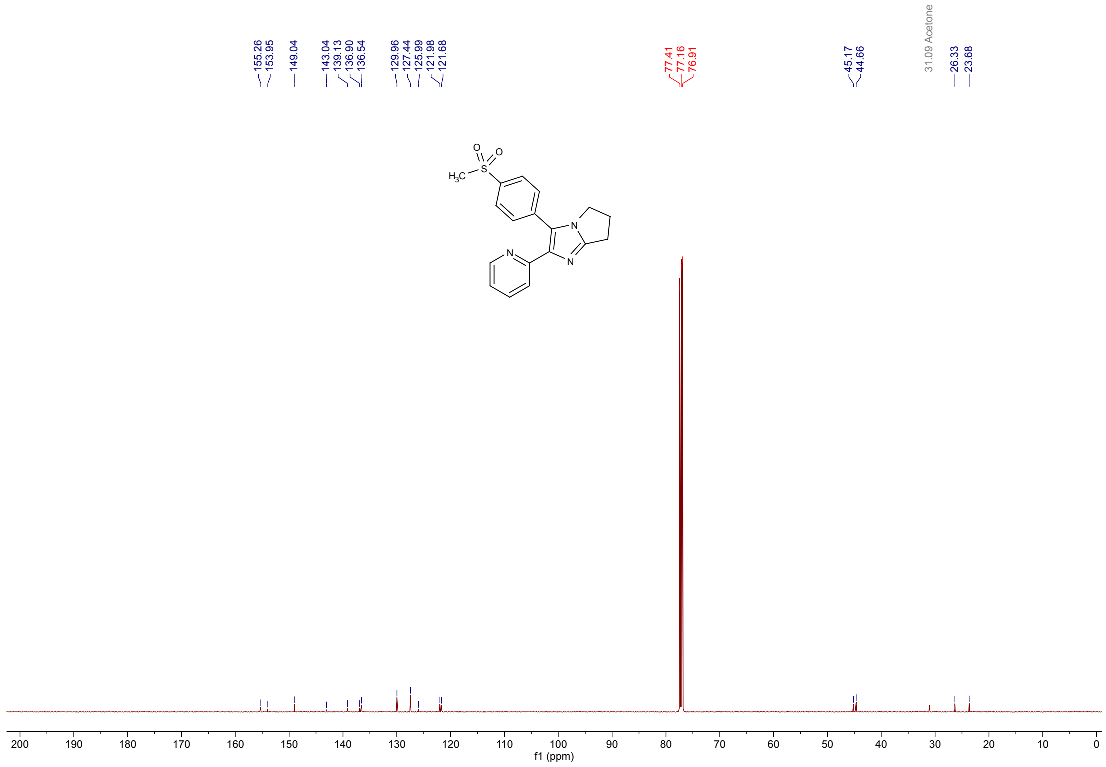

N,*N*-Dimethyl-4-(2-(pyridin-2-yl)-6,7-dihydro-5*H*-pyrrolo[1,2-*a*]imidazol-3-yl)benzamide (OSA_000974)

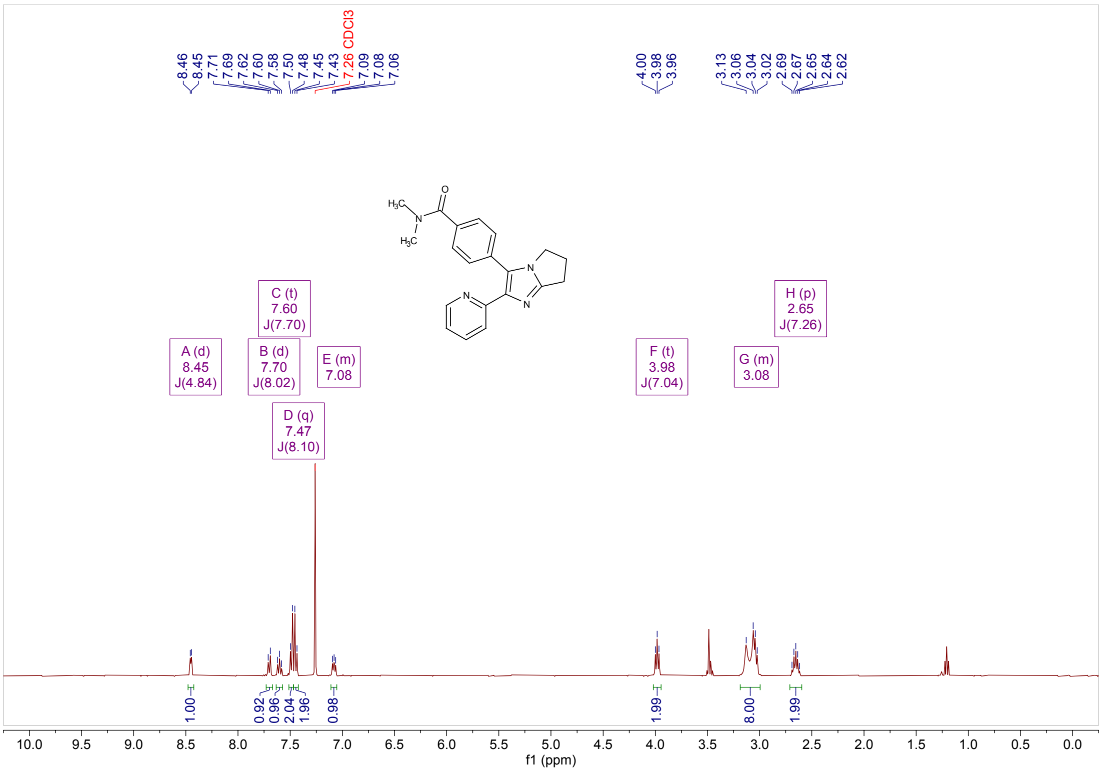

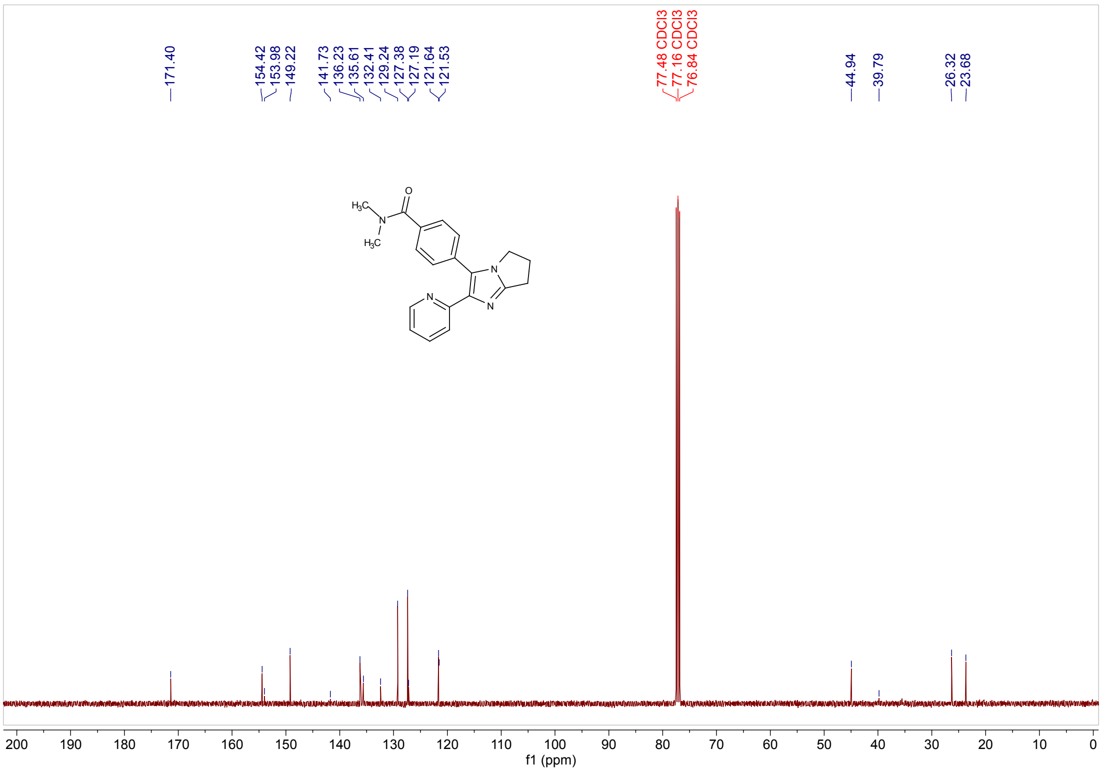

3-(2-(Pyridin-2-yl)-6,7-dihydro-5*H*-pyrrolo[1,2-*a*]imidazol-3-yl)benzonitrile (OSA_000864)

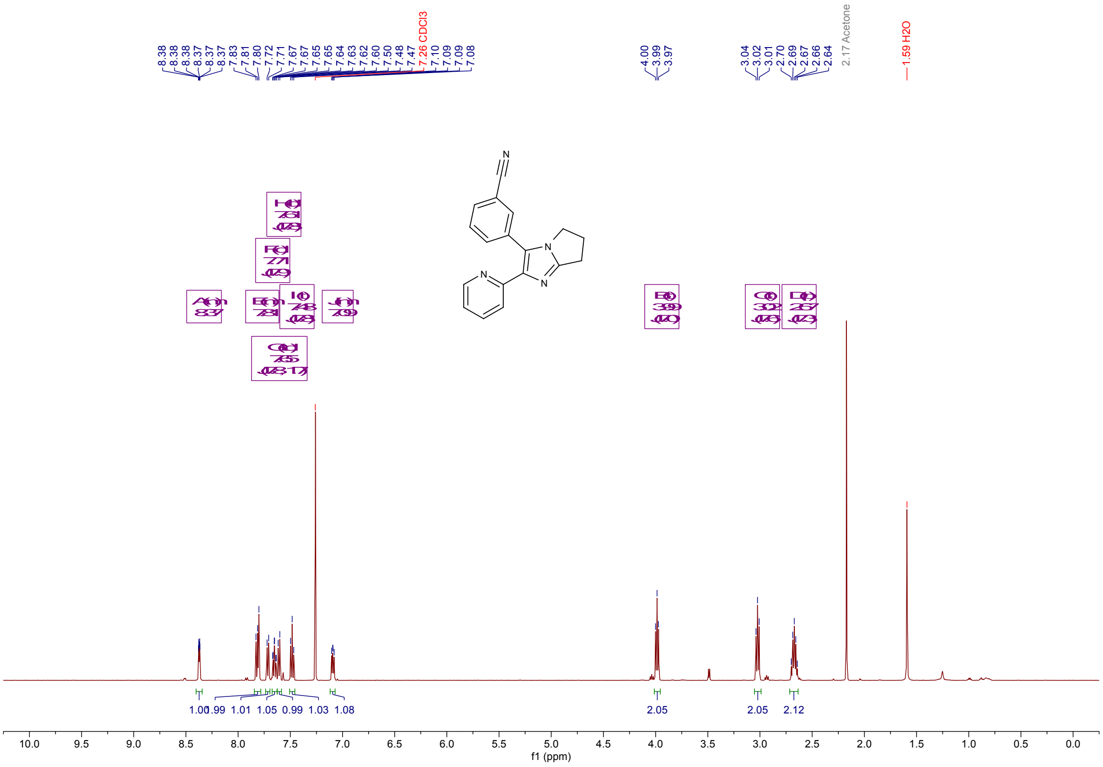

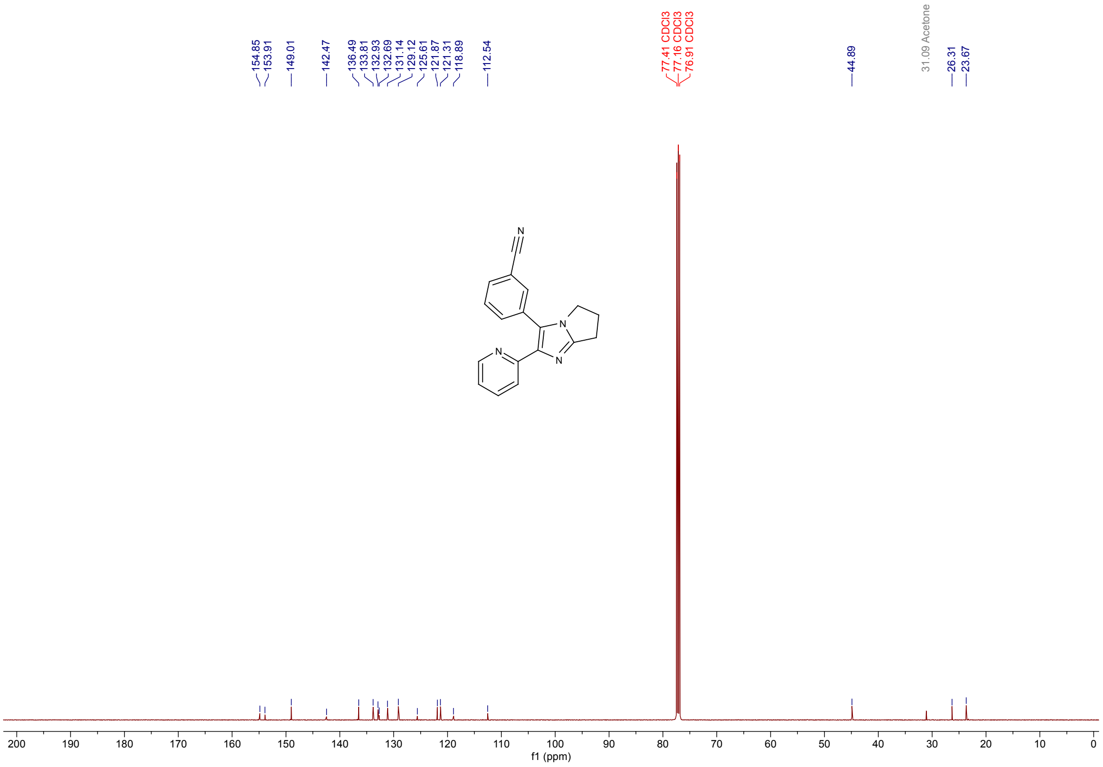

3-(3-Fluorophenyl)-2-(pyridin-2-yl)-6,7-dihydro-5*H*-pyrrolo[1,2-*a*]imidazole (OSA_000872)

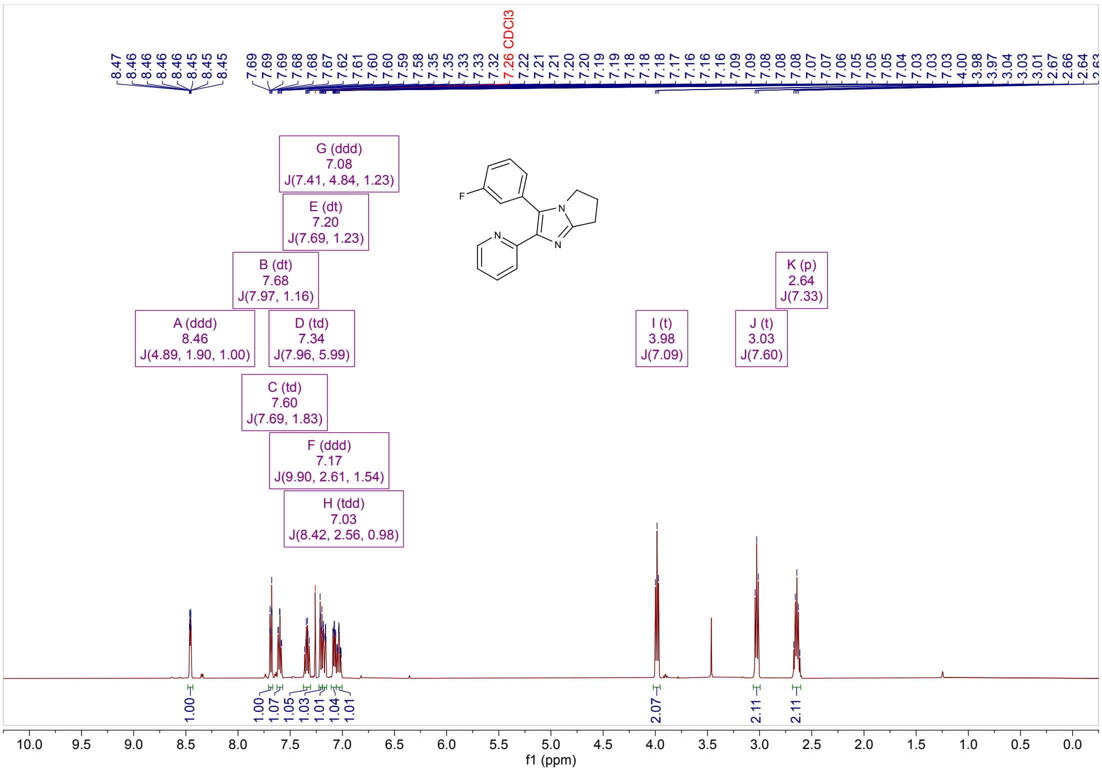

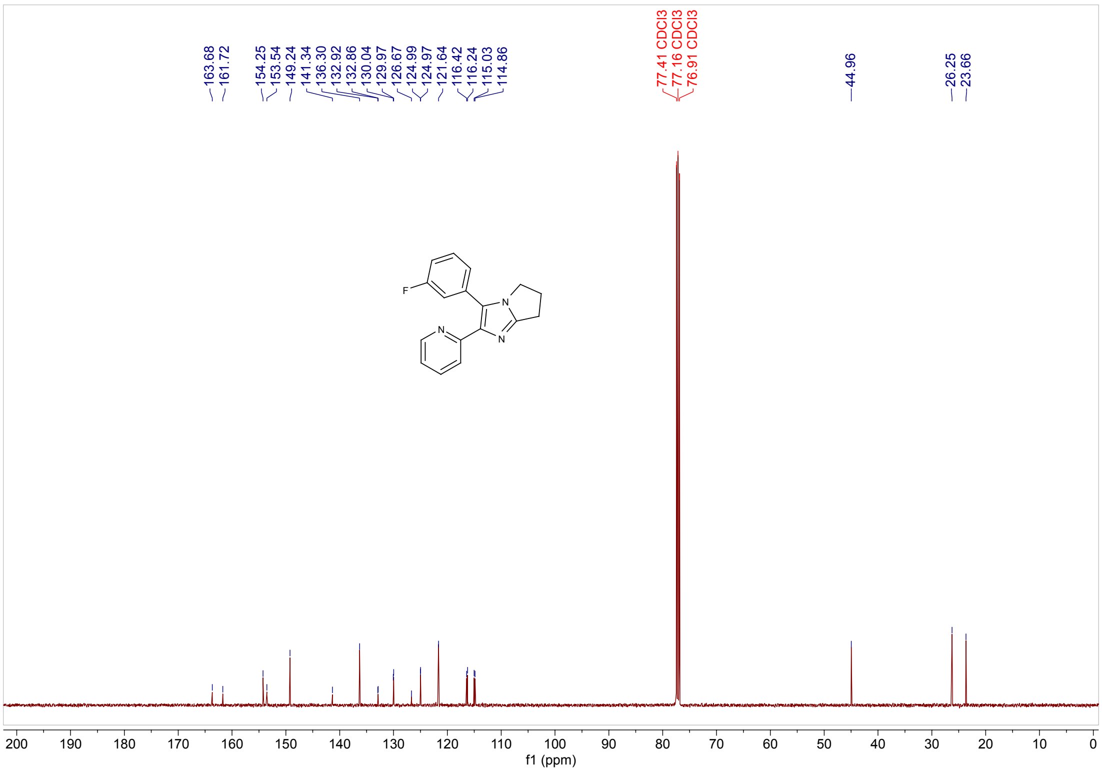

2-(Pyridin-2-yl)-3-(*o*-tolyl)-6,7-dihydro-5*H*-pyrrolo[1,2-*a*]imidazole (OSA_000977)

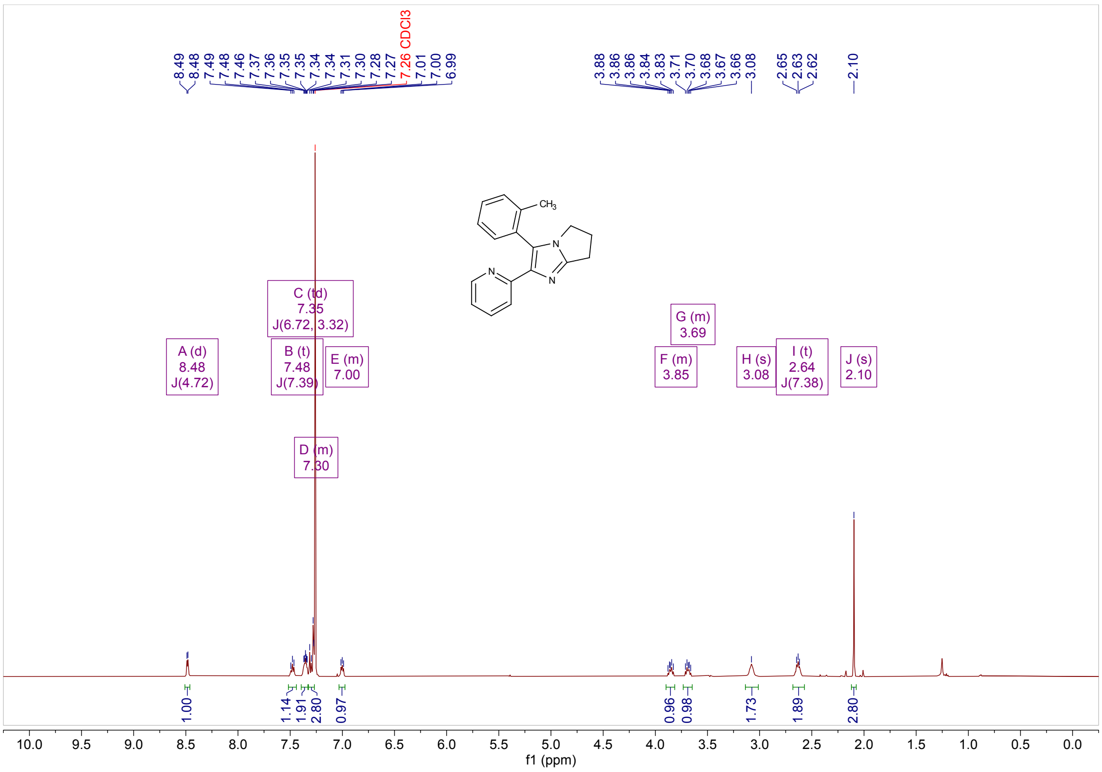

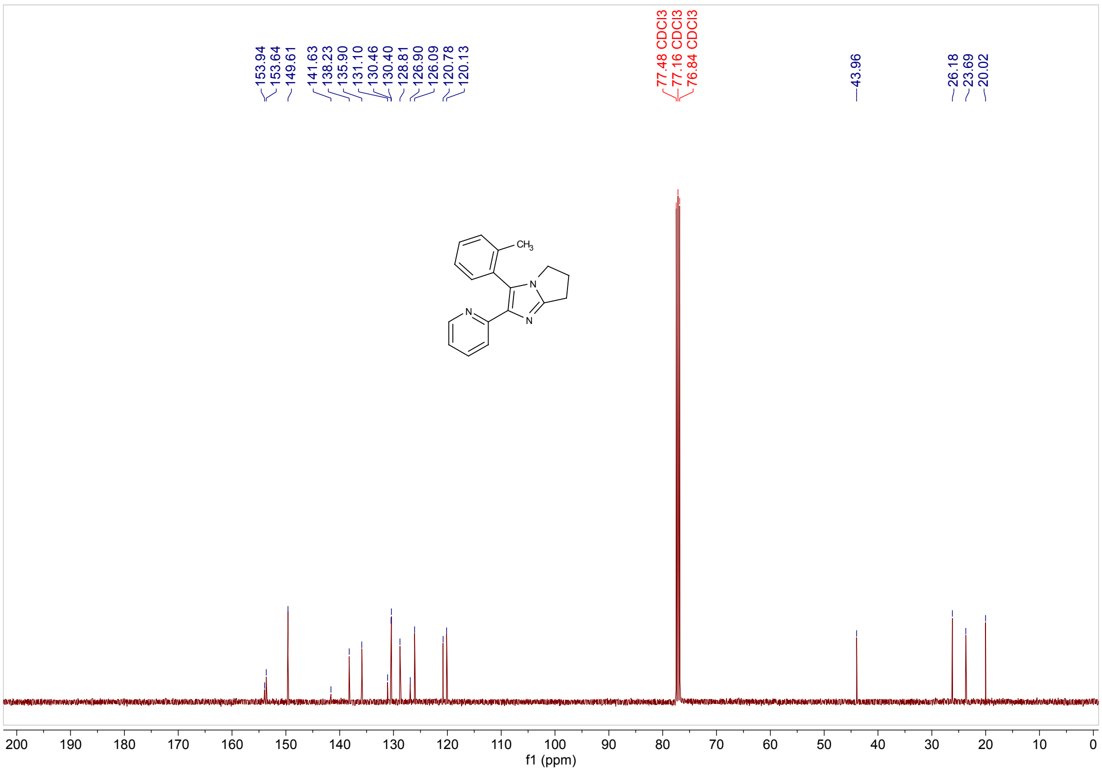

3-(3,4-Dimethoxyphenyl)-2-(pyridin-2-yl)-6,7-dihydro-5*H*-pyrrolo[1,2-*a*]imidazole (OSA_000820)

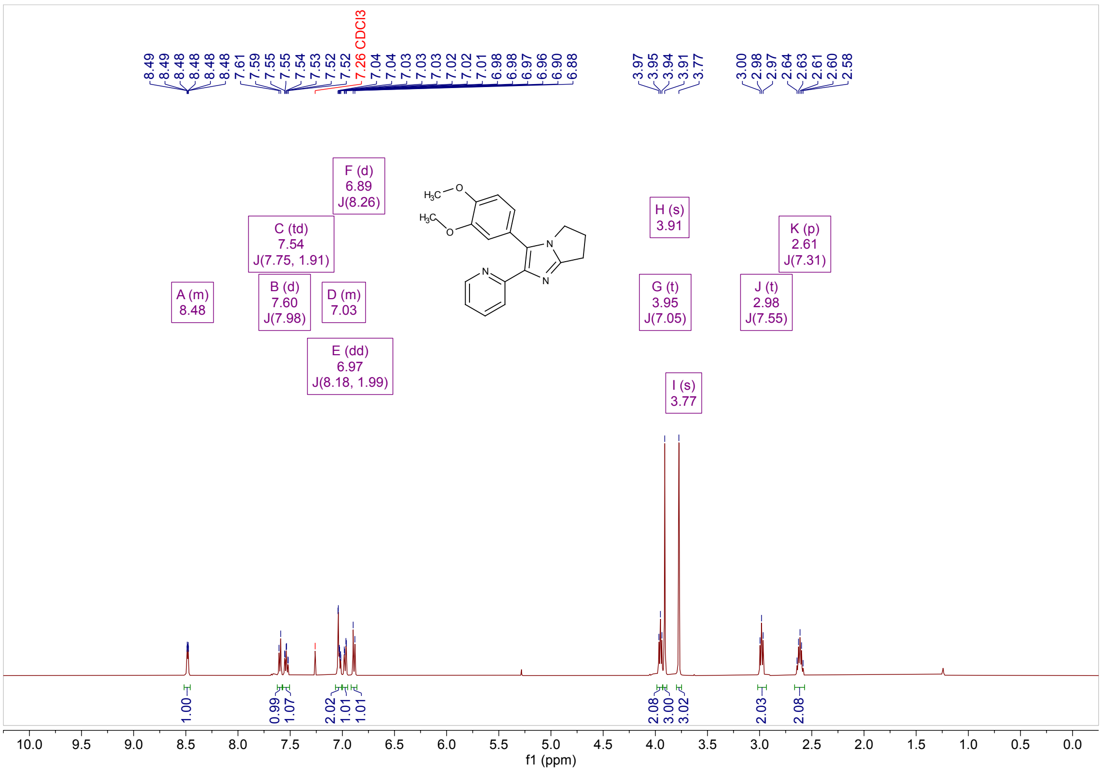

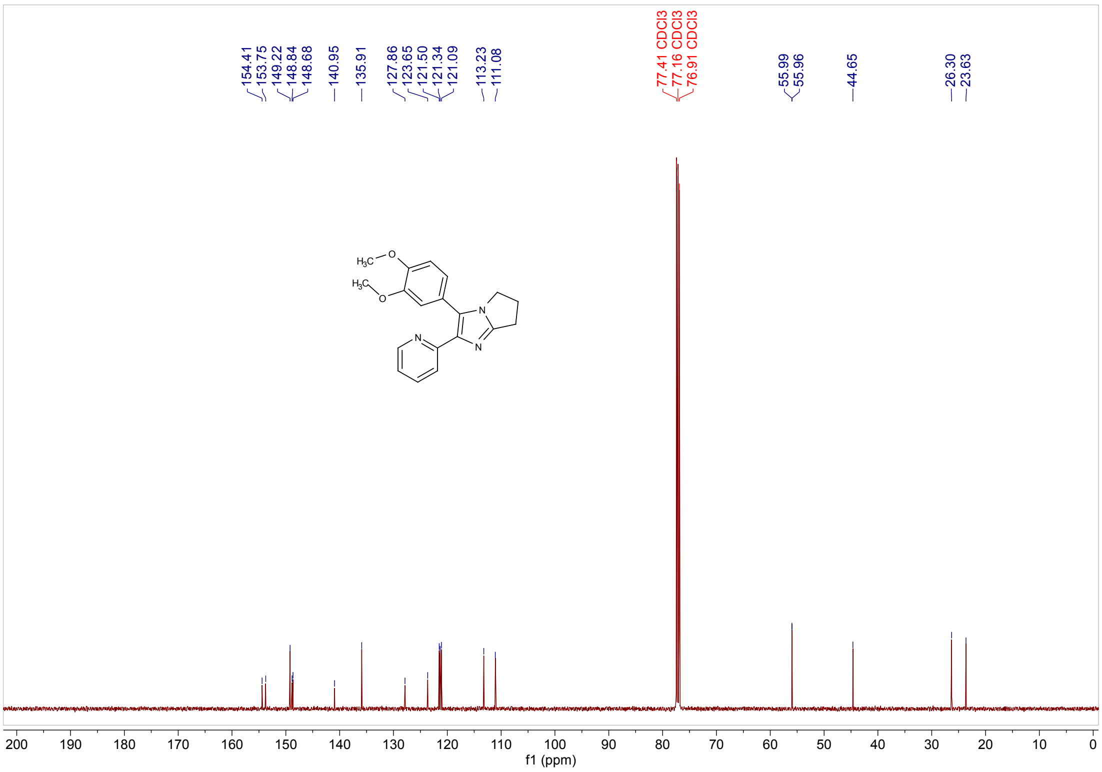

3-(Benzofuran-5-yl)-2-(pyrazin-2-yl)-6,7-dihydro-5*H*-pyrrolo[1,2-*a*]imidazole

(OSA_000873)

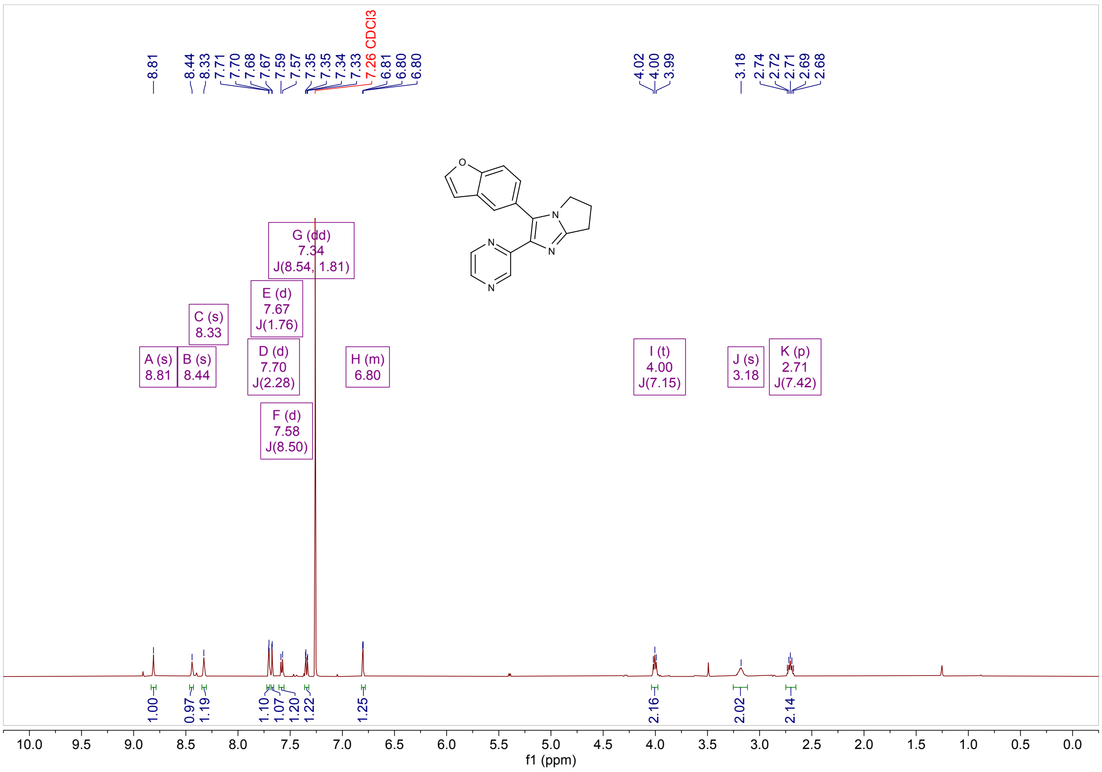

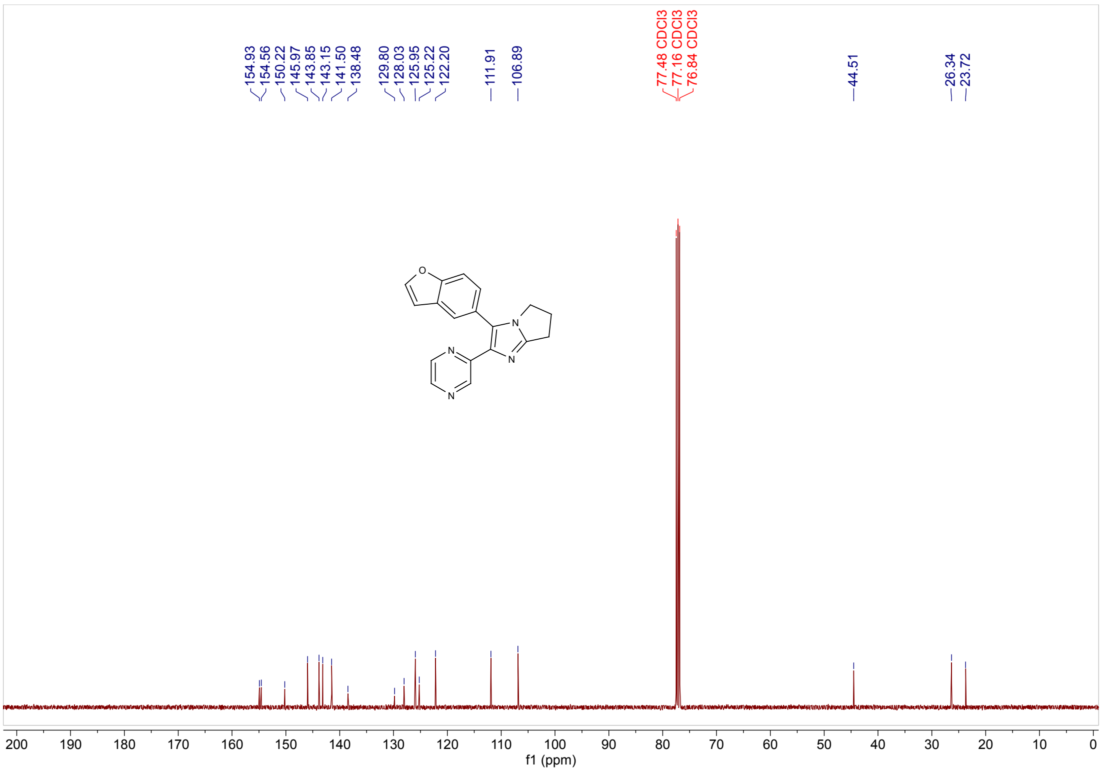

3-(4-Chlorophenyl)-2-(pyridin-2-yl)-6,7-dihydro-5*H*-pyrrolo[1,2-*a*]imidazole

(OSA_000874)

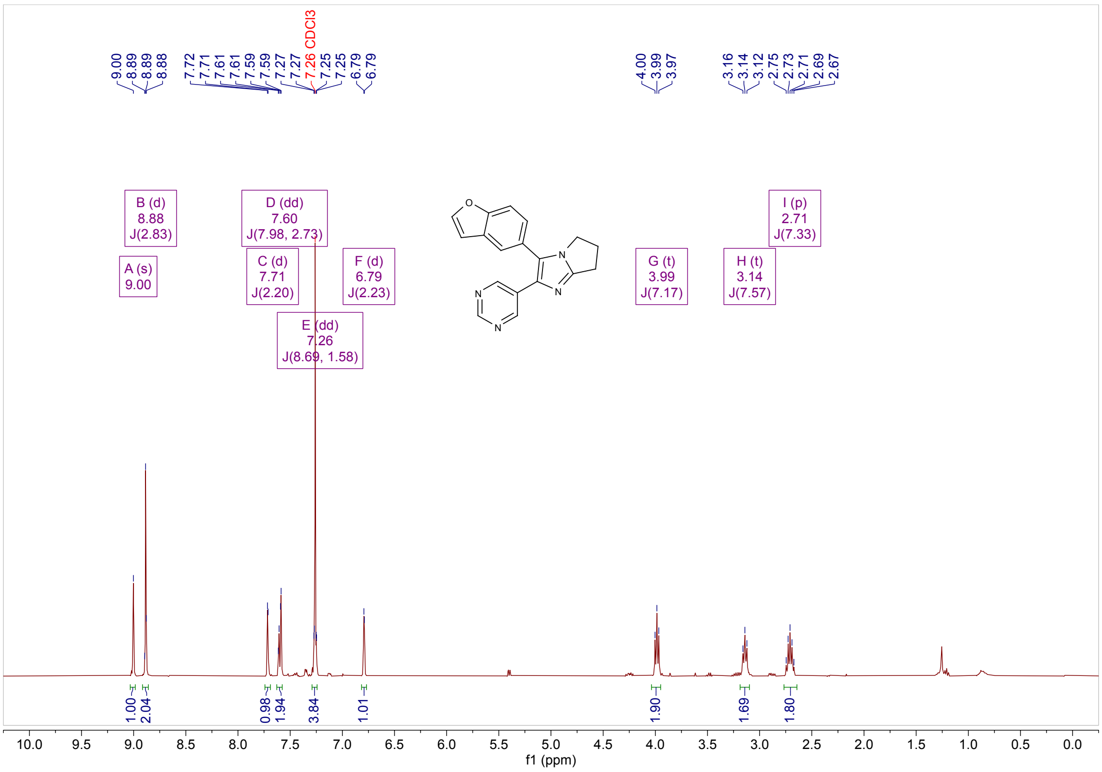

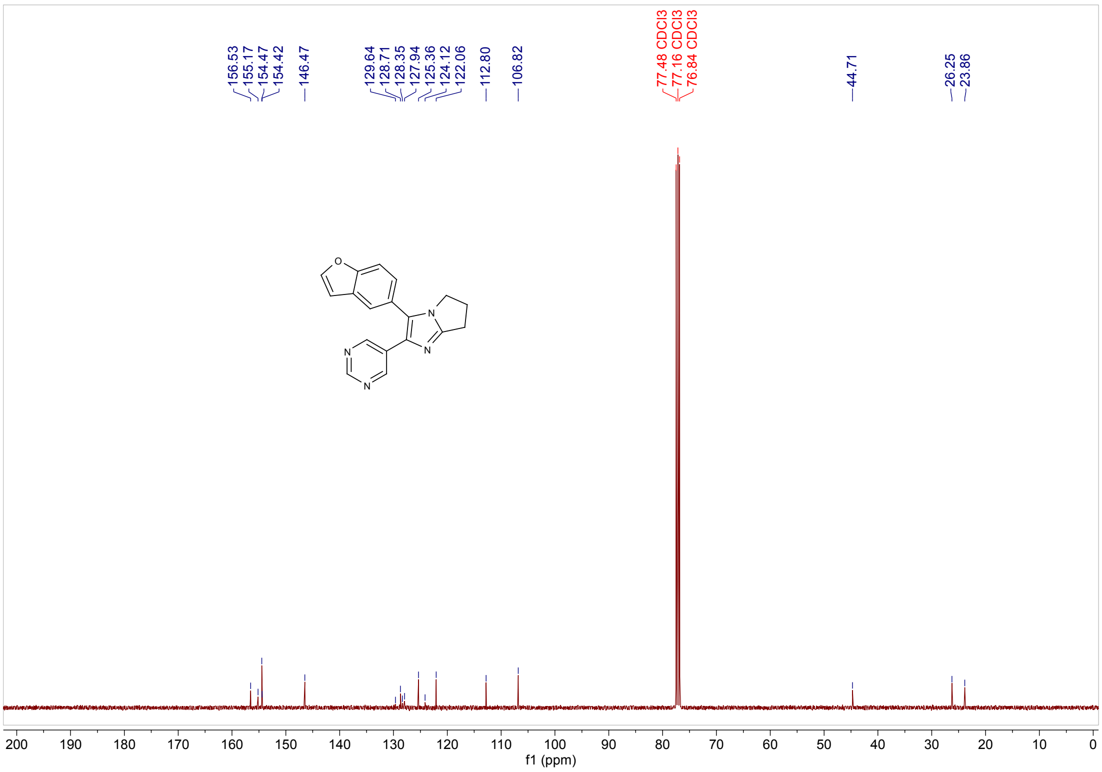

3-(Benzofuran-5-yl)-2-(pyridin-3-yl)-6,7-dihydro-5*H*-pyrrolo[1,2-*a*]imidazole

(OSA_000861)

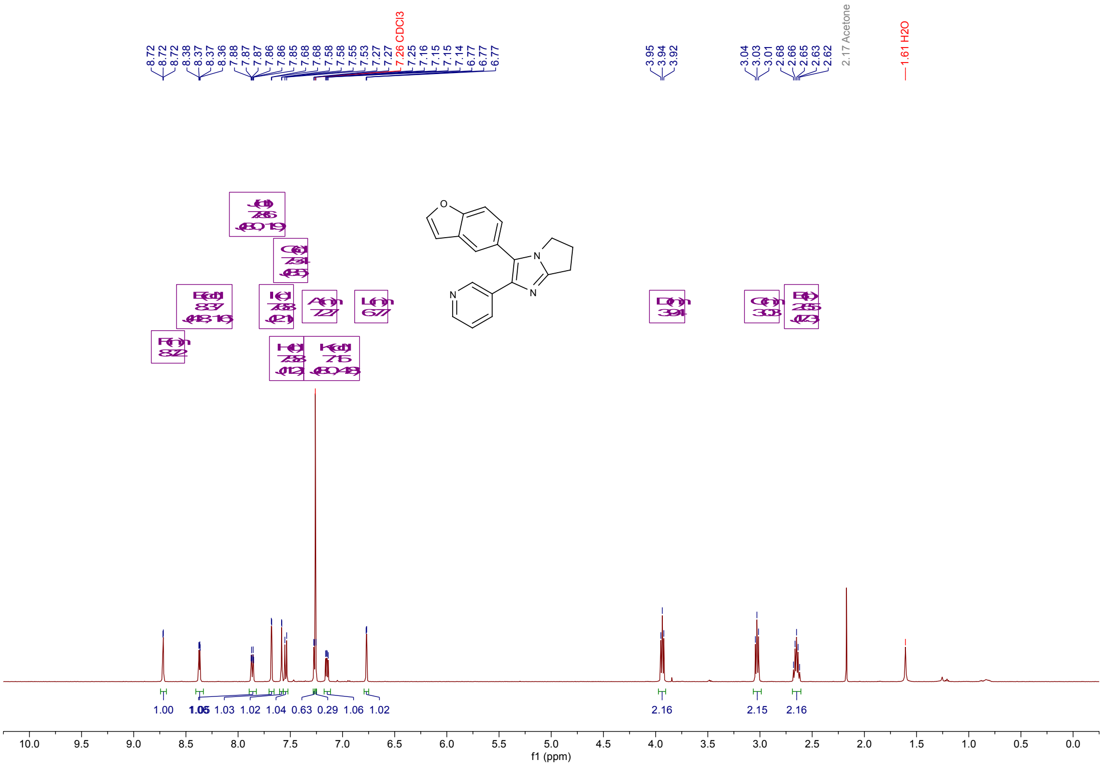

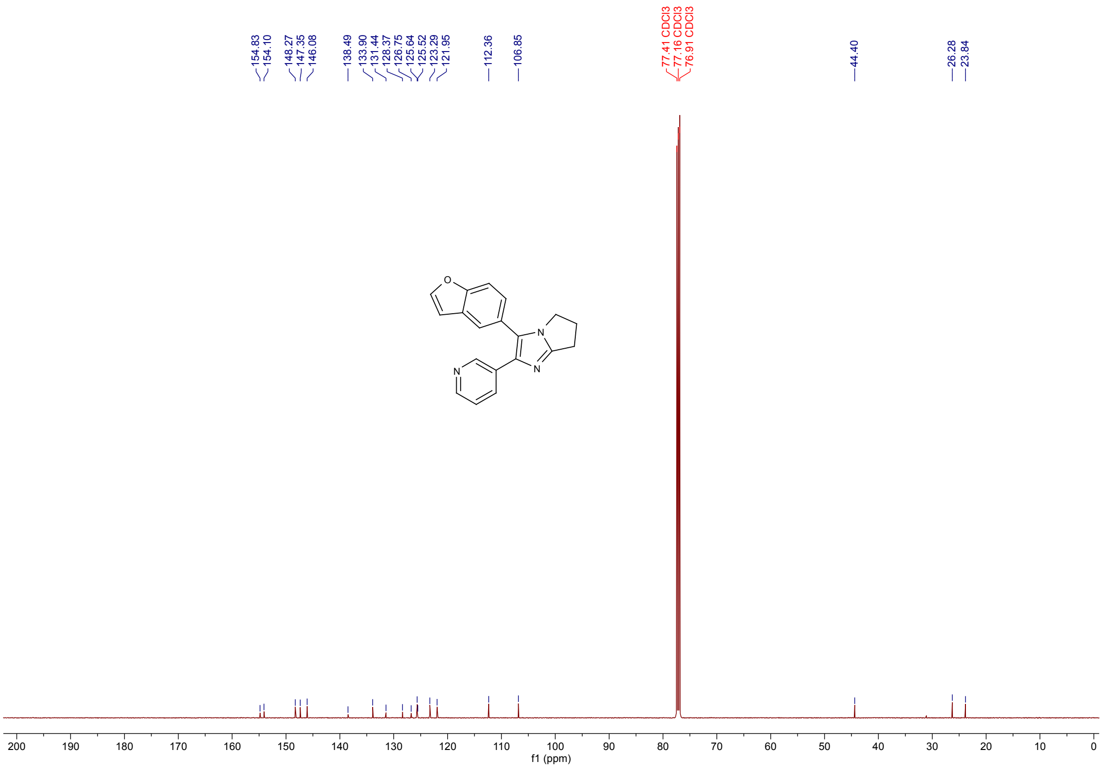

3-(Benzofuran-5-yl)-2-(pyridin-4-yl)-6,7-dihydro-5*H*-pyrrolo[1,2-*a*]imidazole

(OSA_000862)

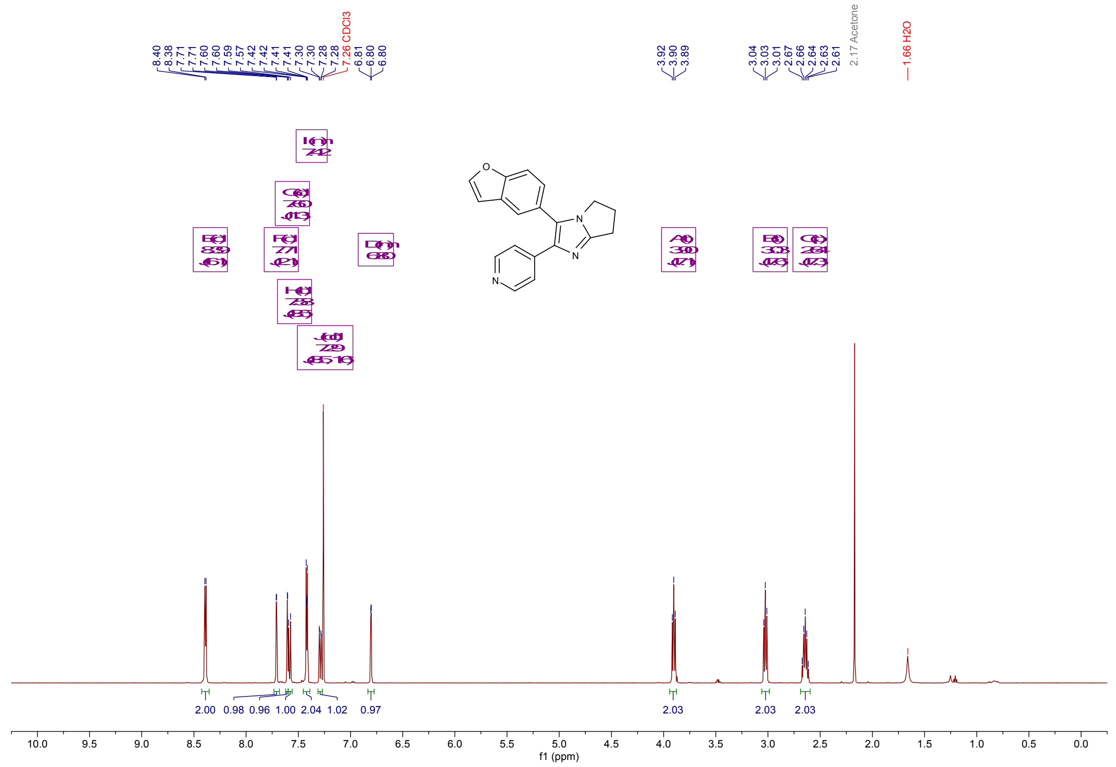

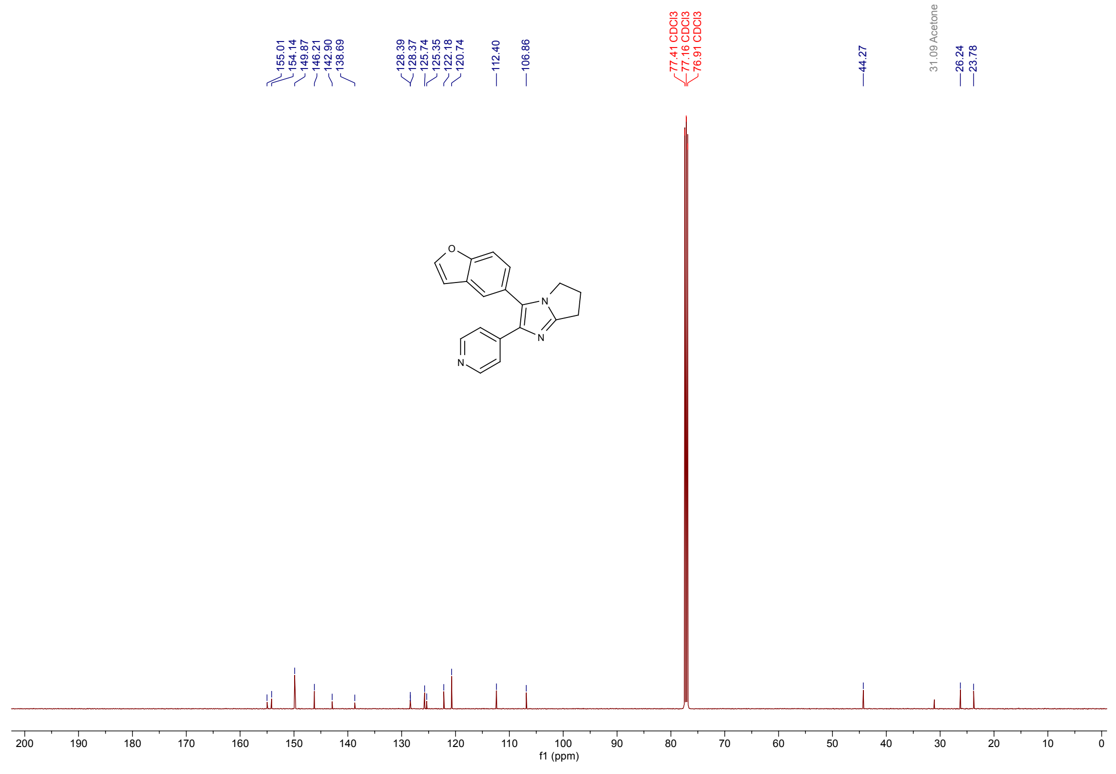

3-(Benzofuran-5-yl)-2-phenyl-6,7-dihydro-5*H*-pyrrolo[1,2-*a*]imidazole (OSA_000869)

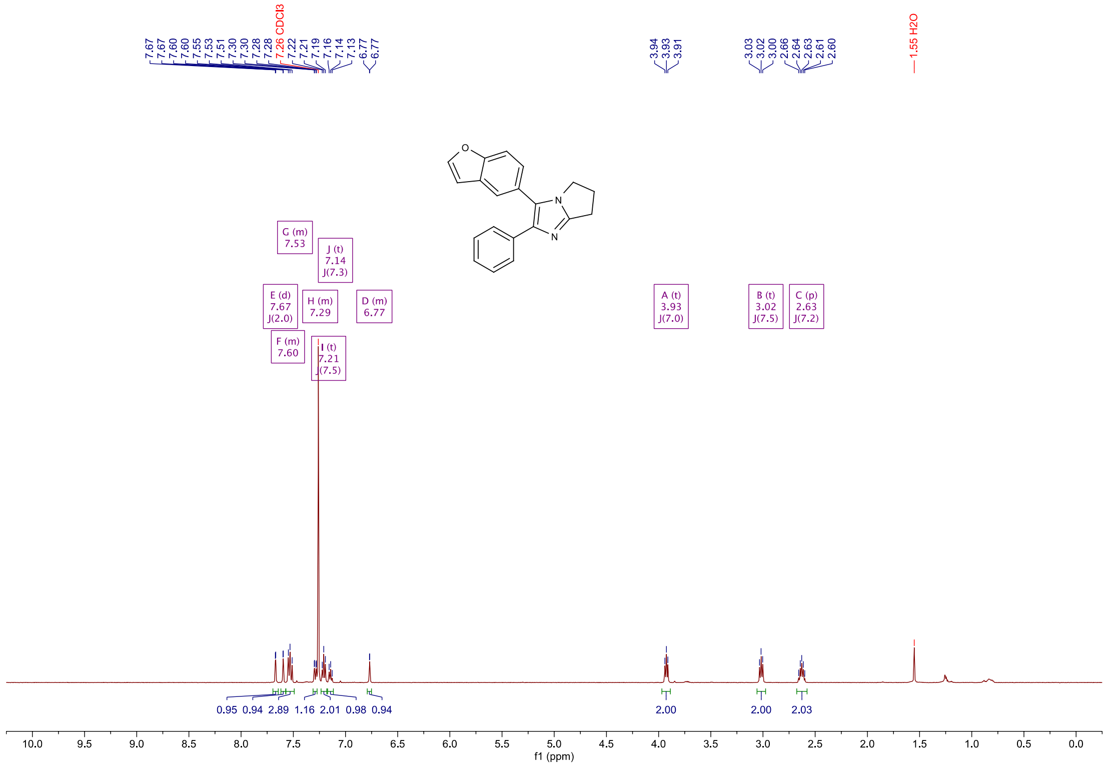

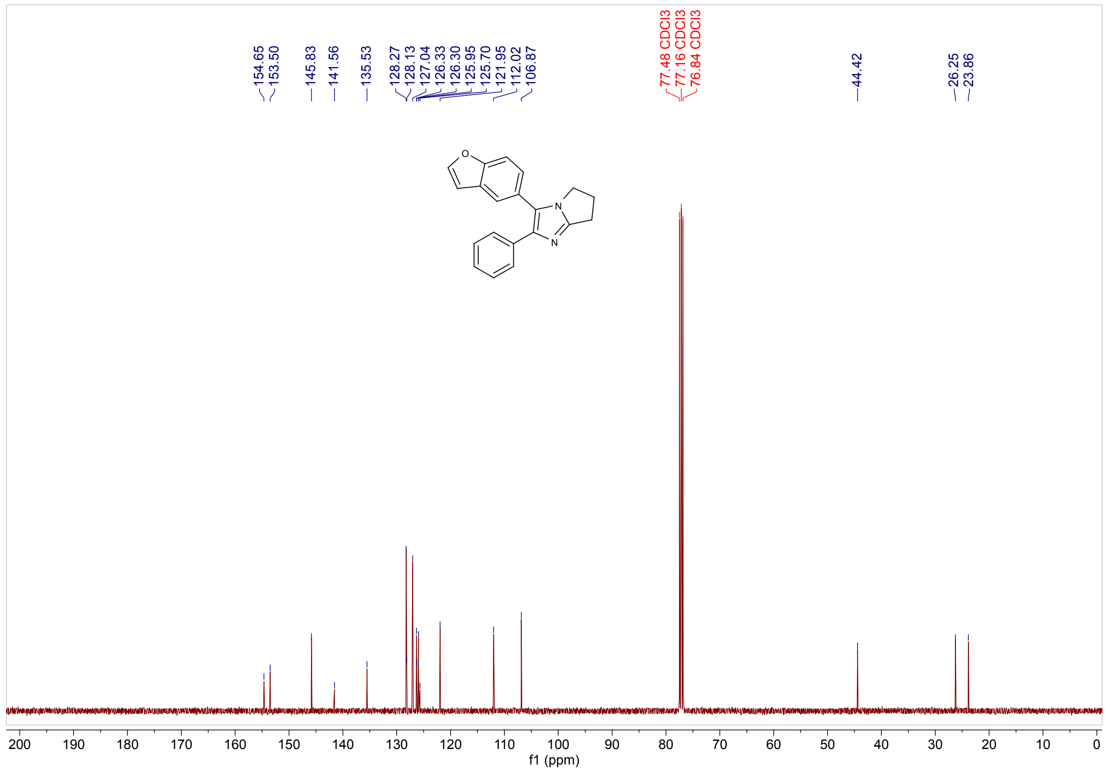

3-(Benzo[*b*]thiophen-5-yl)-2-(pyridin-2-yl)imidazo[1,2-*a*]pyridine (OSA_000871)

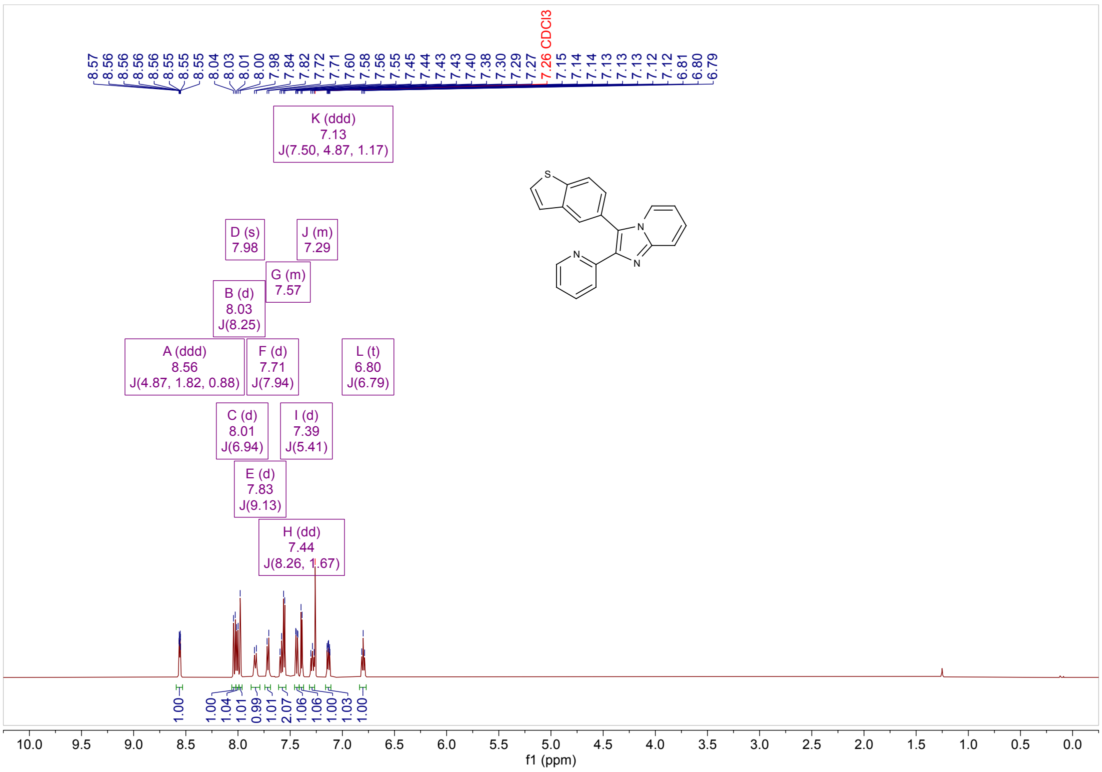

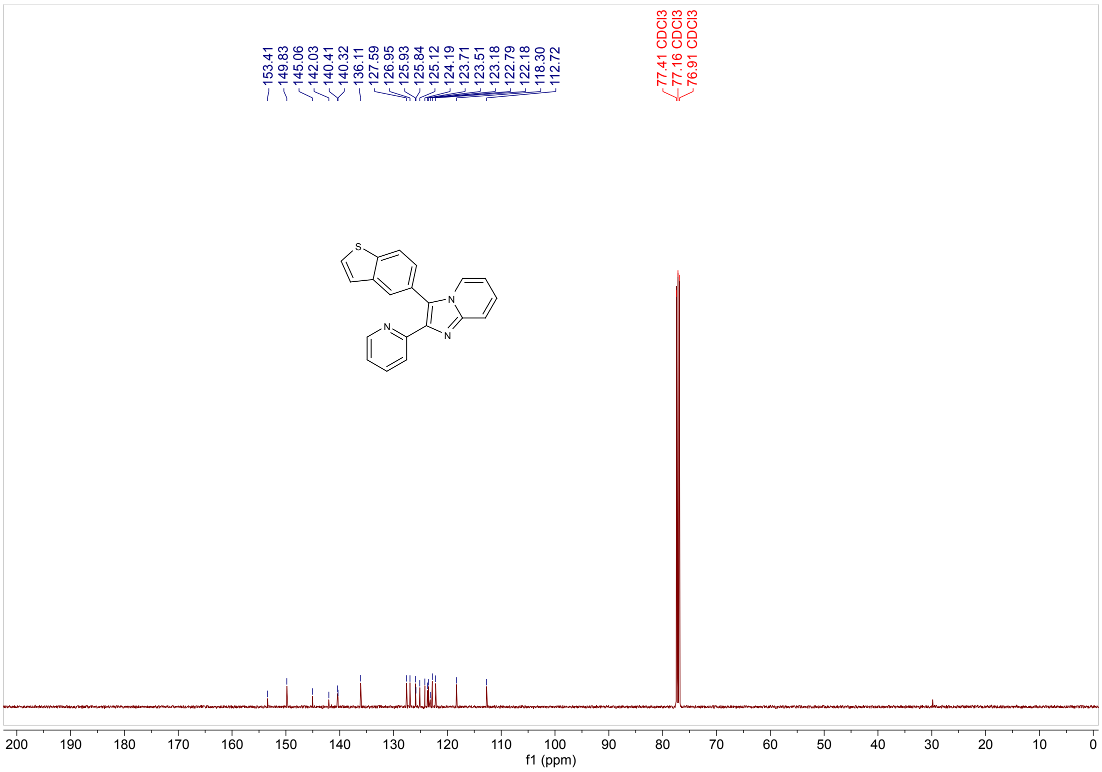

2-(Pyridin-2-yl)-3-(*p*-tolyl)imidazo[1,2-*a*]pyridine (OSA_001018)

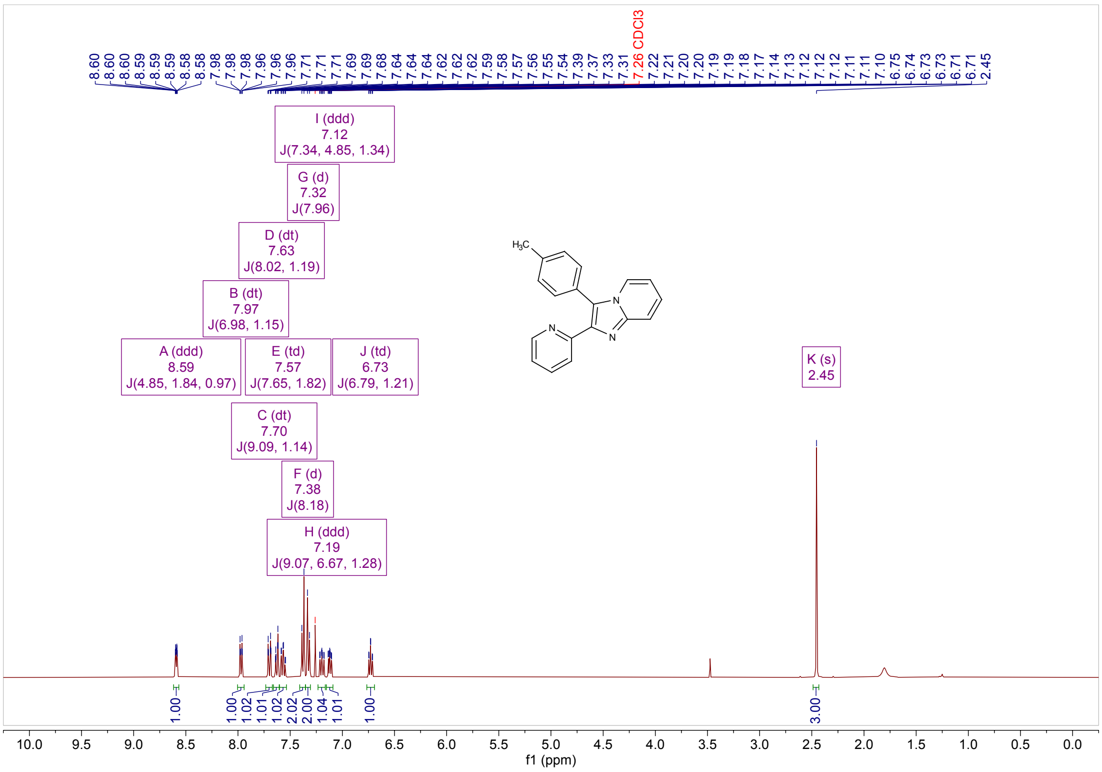

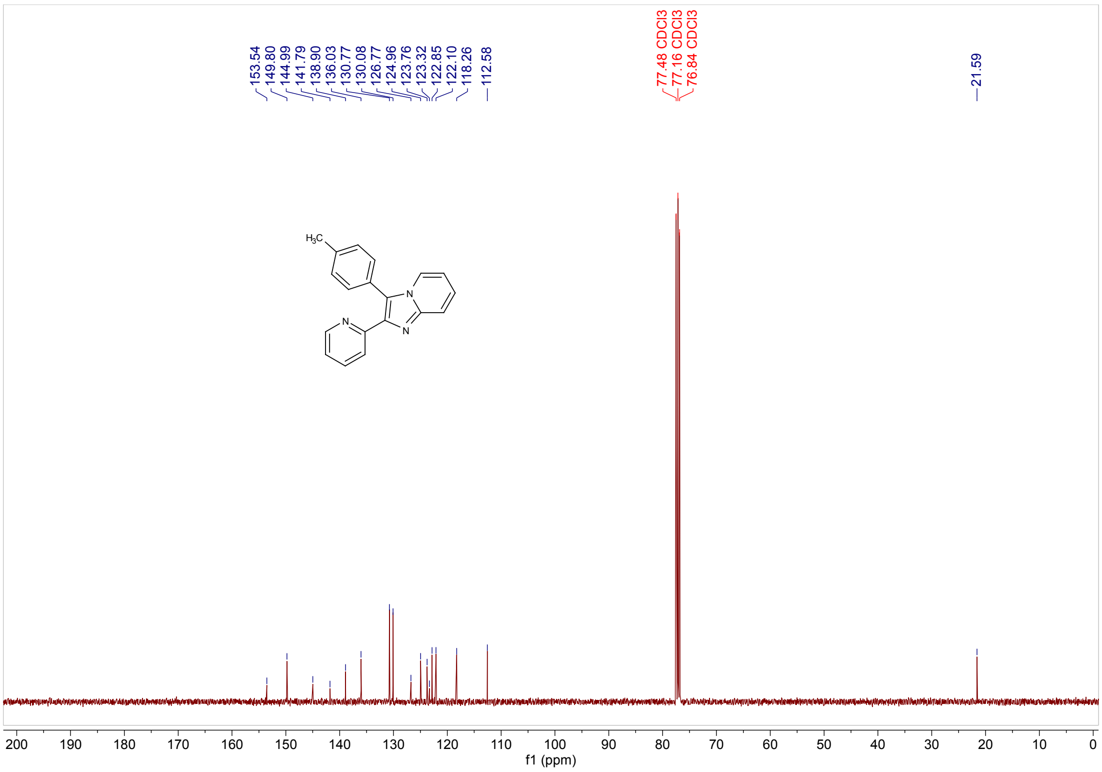

N,*N*-Diethyl-4-(2-(pyridin-2-yl)imidazo[1,2-*a*]pyridin-3-yl)aniline (OSA_001011)

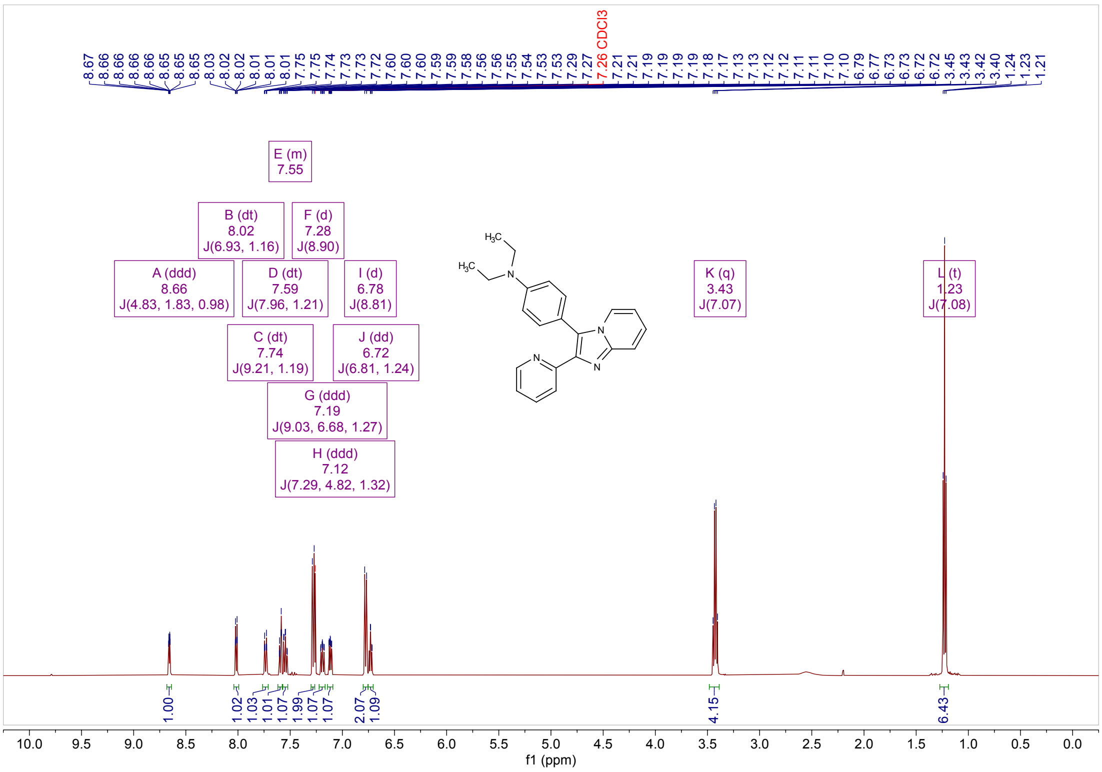

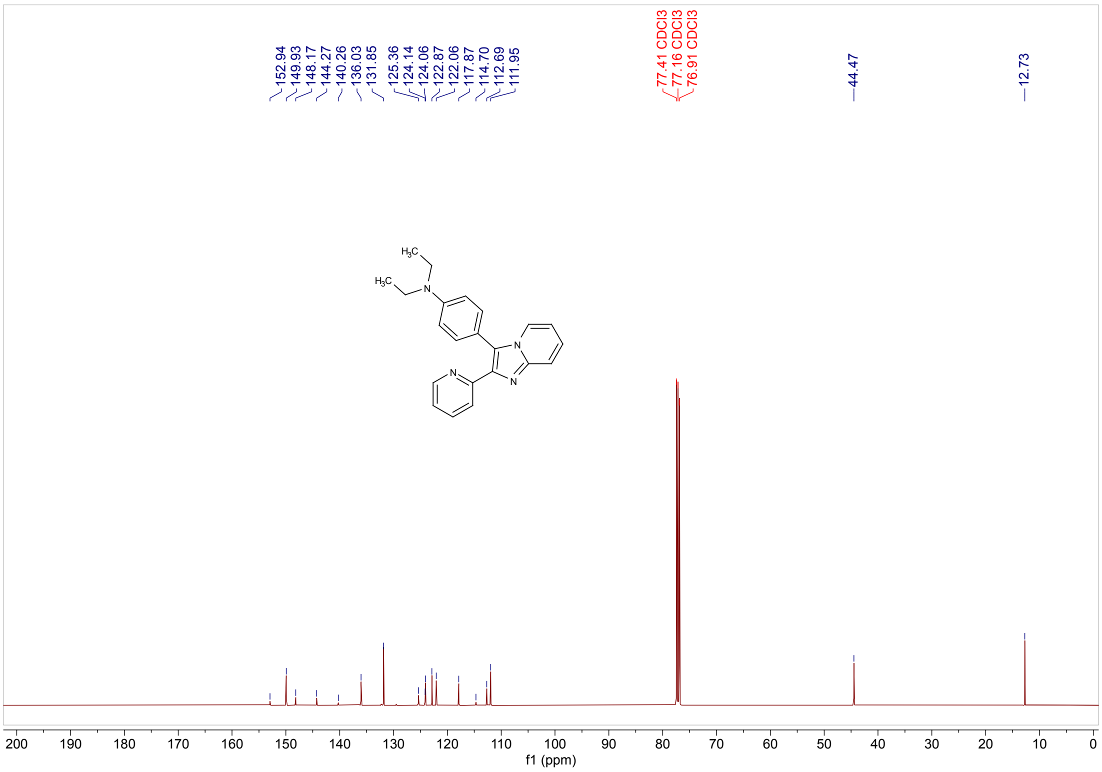

2-(Pyridin-2-yl)-*N*-(*p*-tolyl)imidazo[1,2-*a*]pyridin-3-amine (OSA_000988)

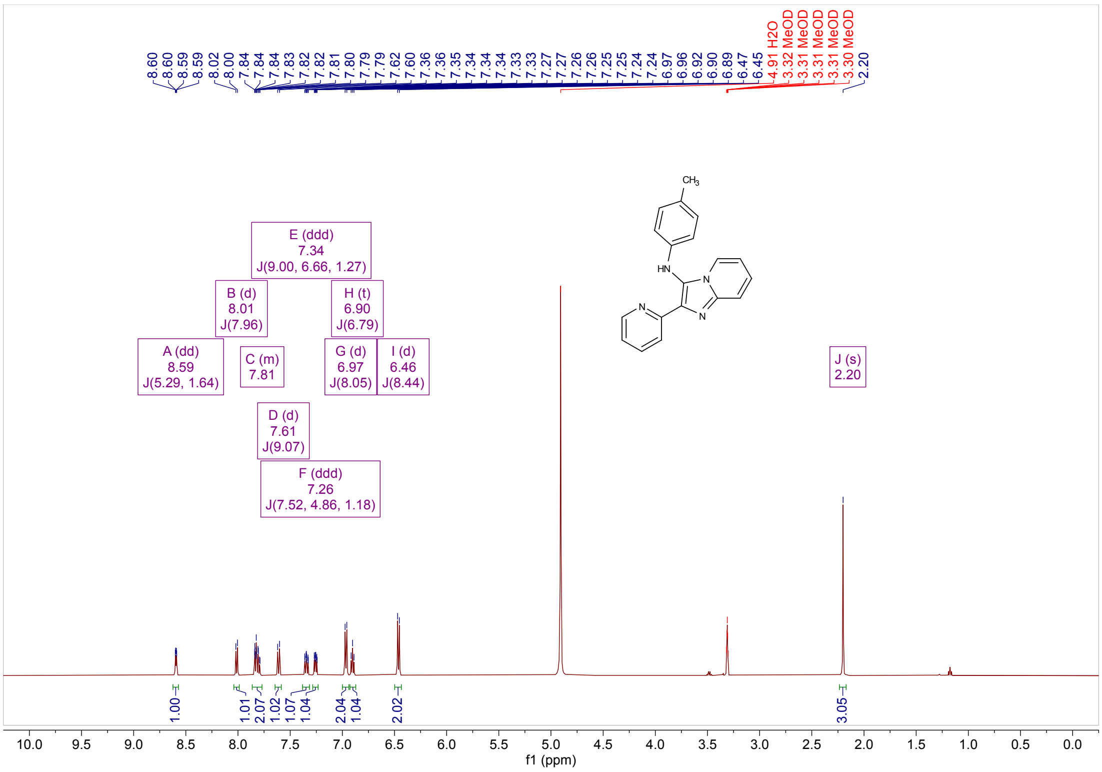

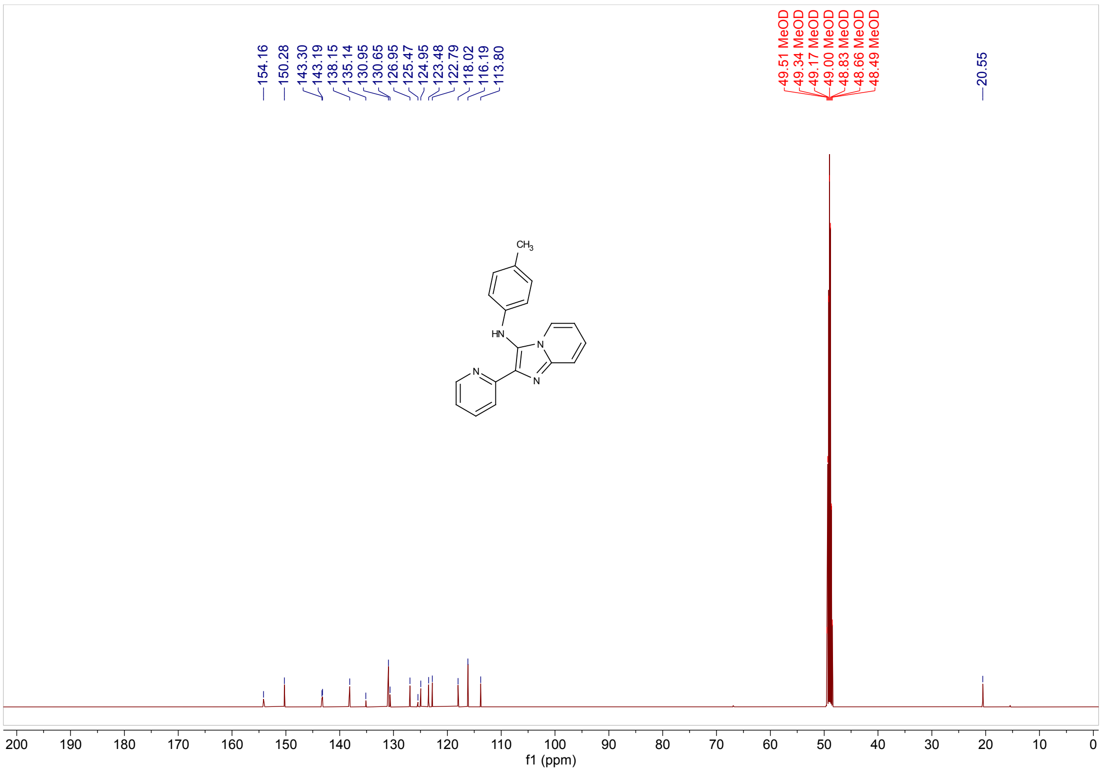

N-(4-Fluorophenyl)-2-(pyridin-2-yl)imidazo[1,2-a]pyridin-3-amine (OSA_000987)

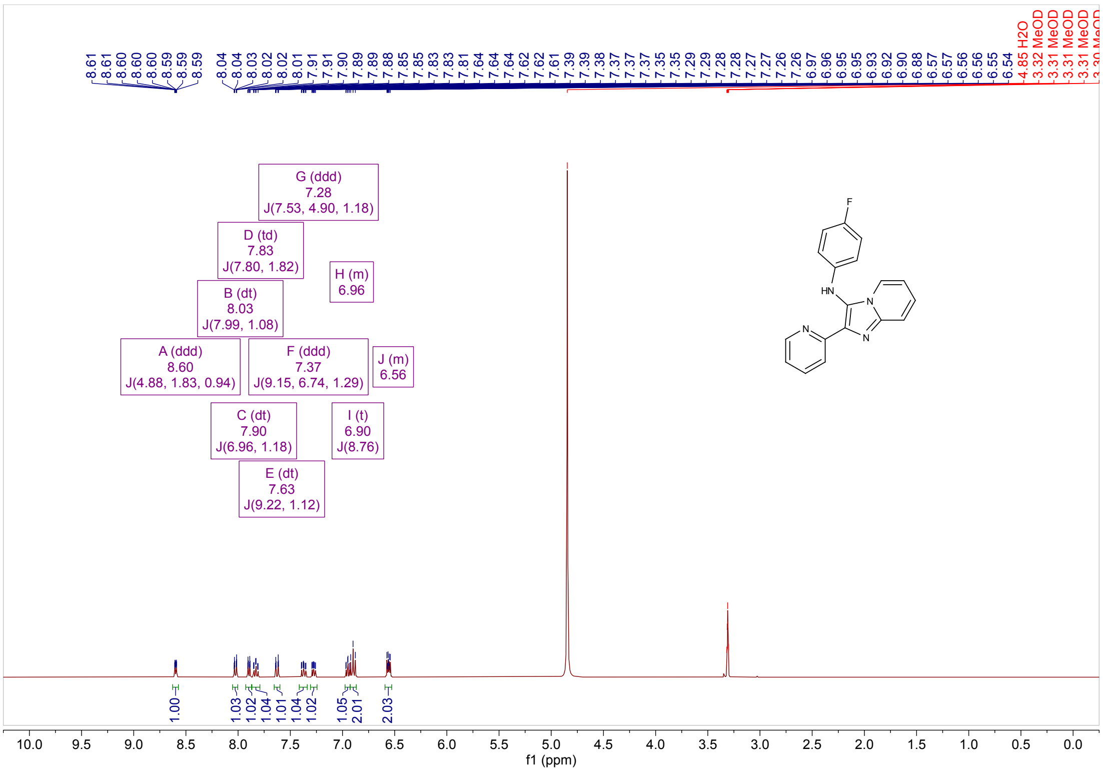

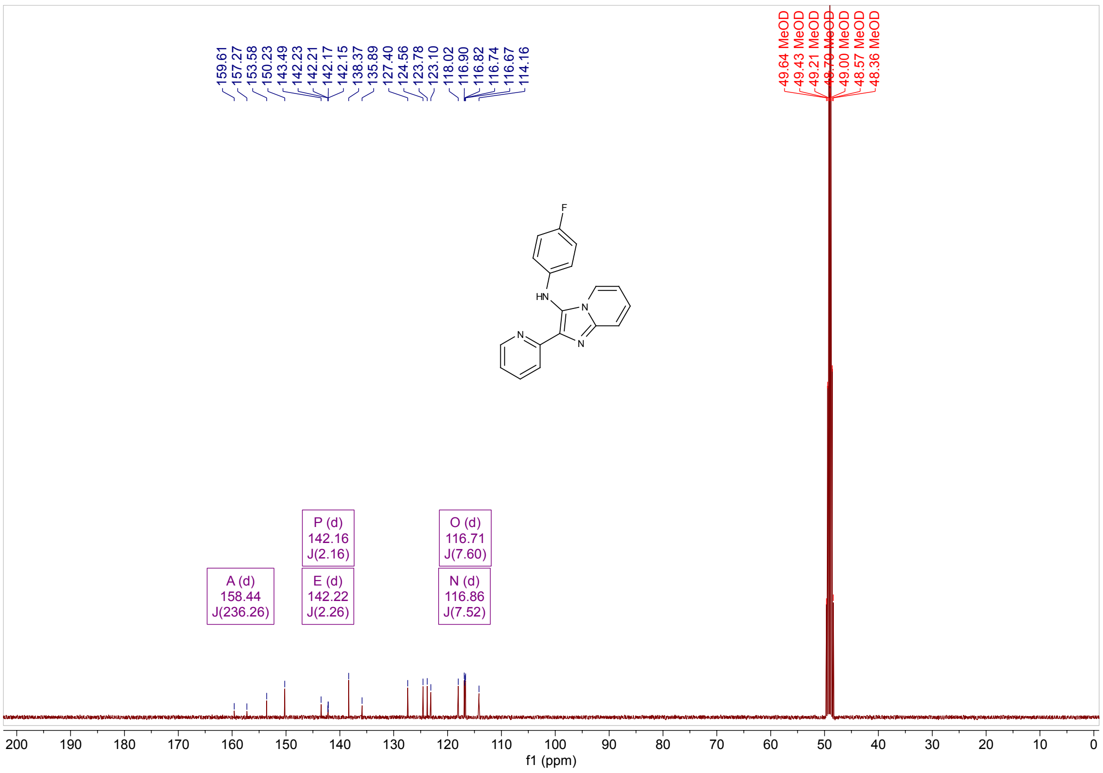

3-(Benzo[*d*][1,3]dioxol-5-yl)-2-(pyridin-2-yl)imidazo[1,2-*a*]pyridine (OSA_000814)

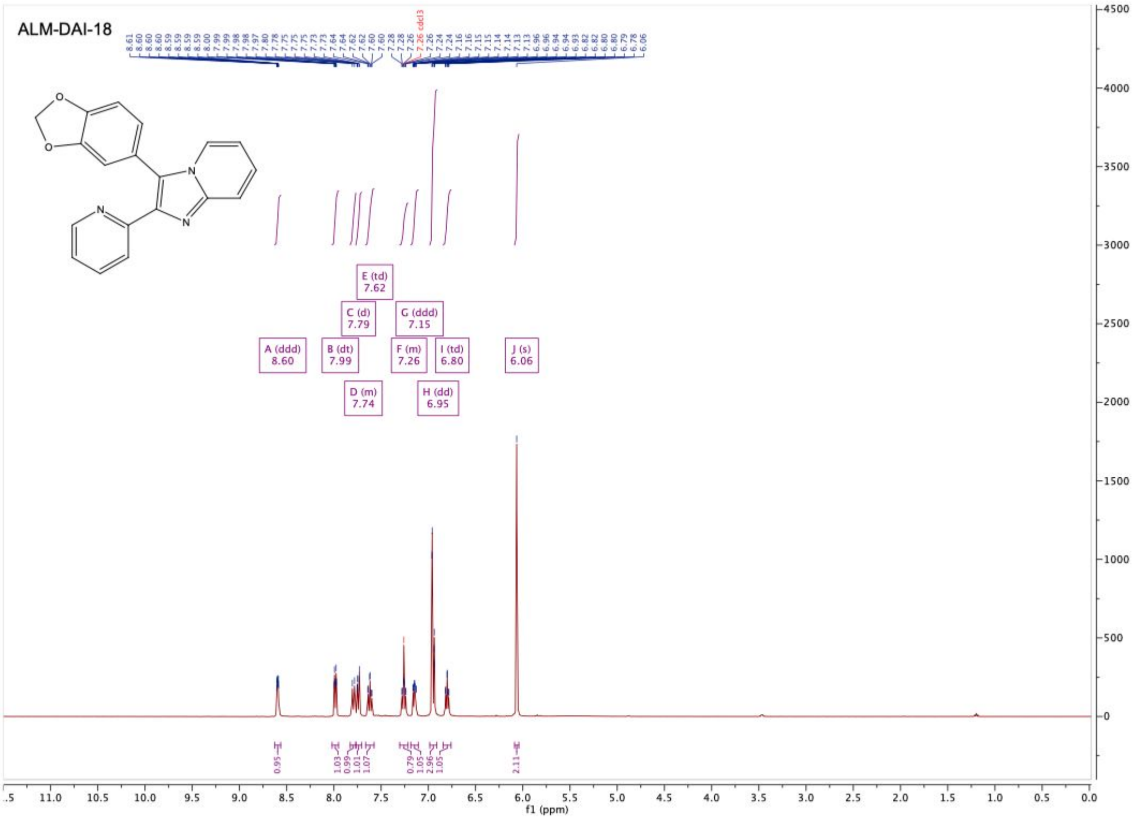

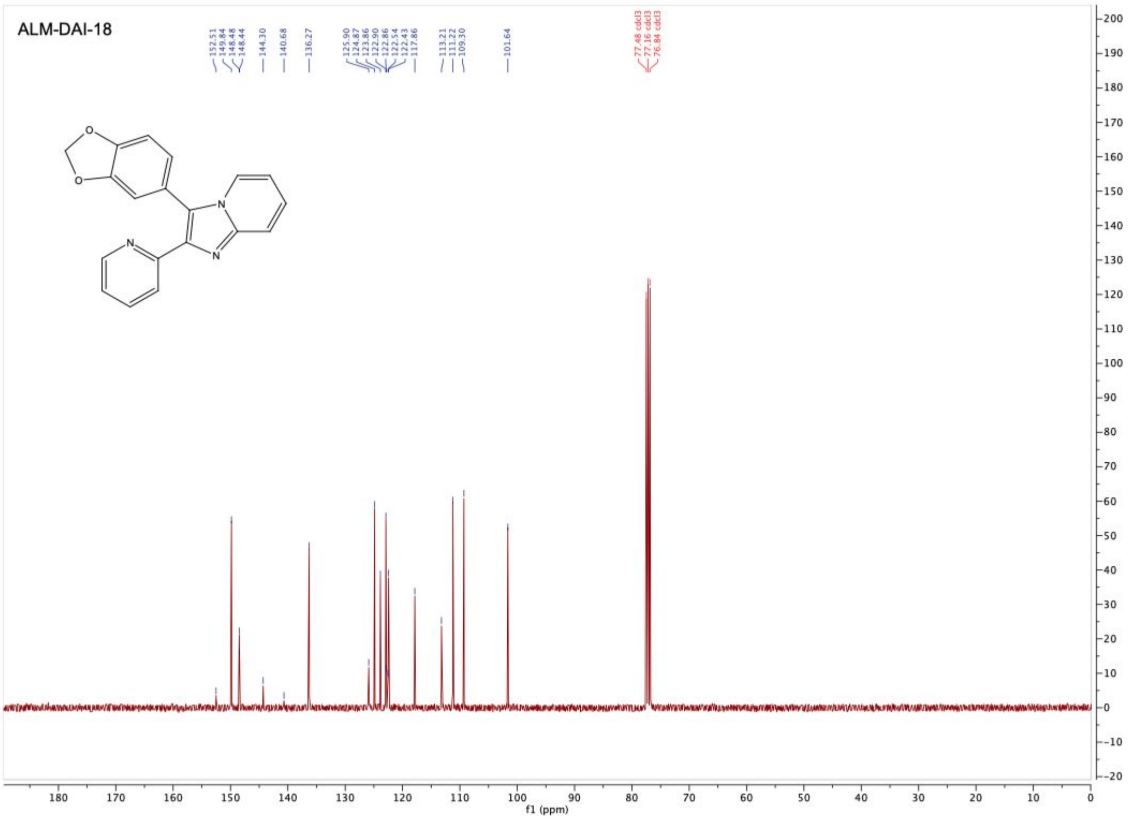

3-(2-Fluoropyridin-4-yl)-2-(pyridin-2-yl)imidazo[1,2-*a*]pyridine (OSA_000986)

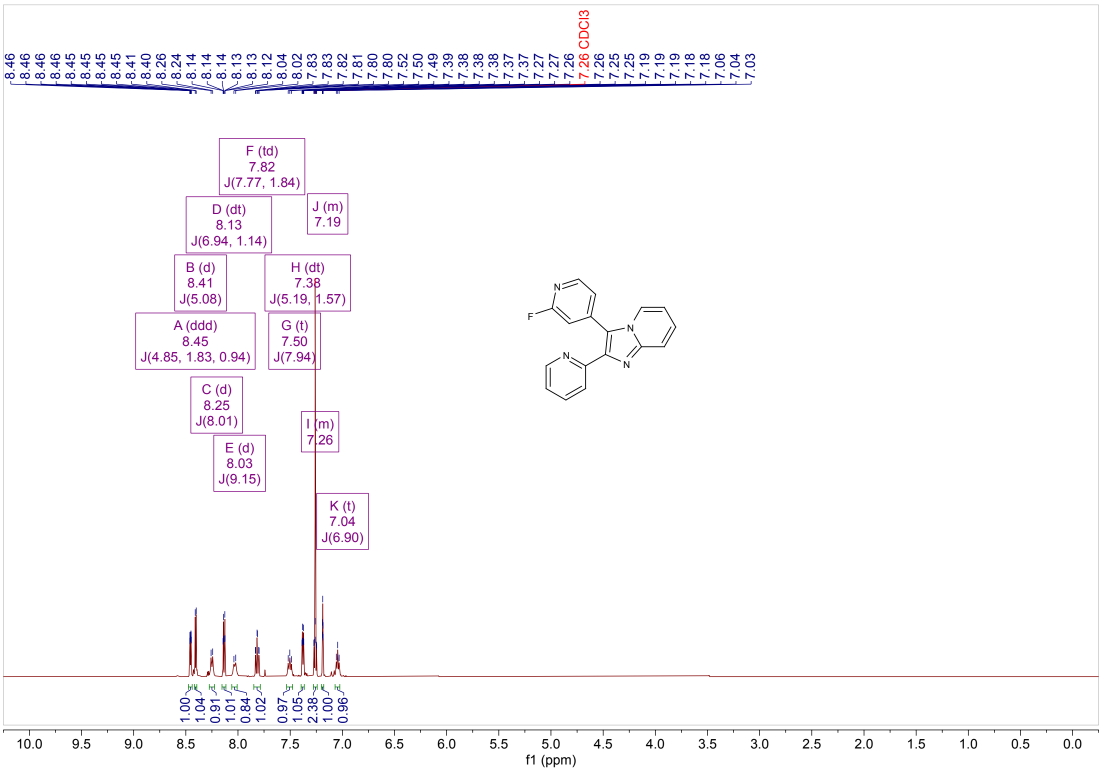

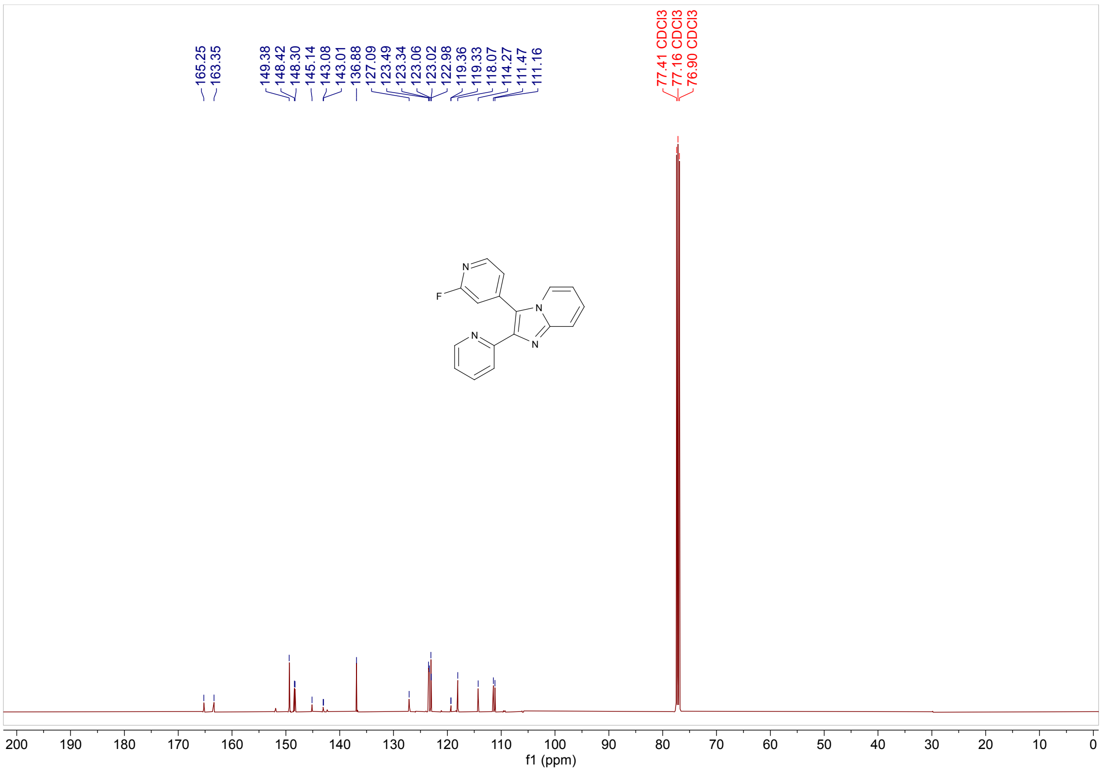

3-(4-(4-Methylpiperazin-1-yl)phenyl)-2-(pyridin-2-yl)imidazo[1,2-*a*]pyridine (OSA_001012)

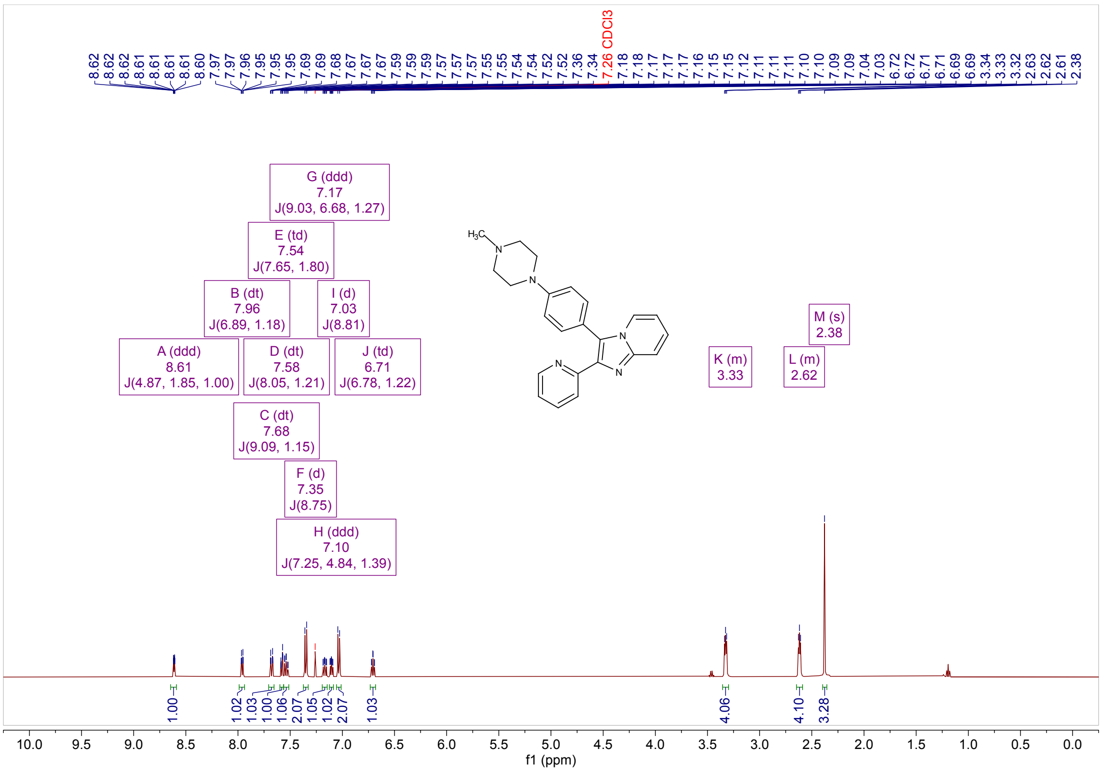

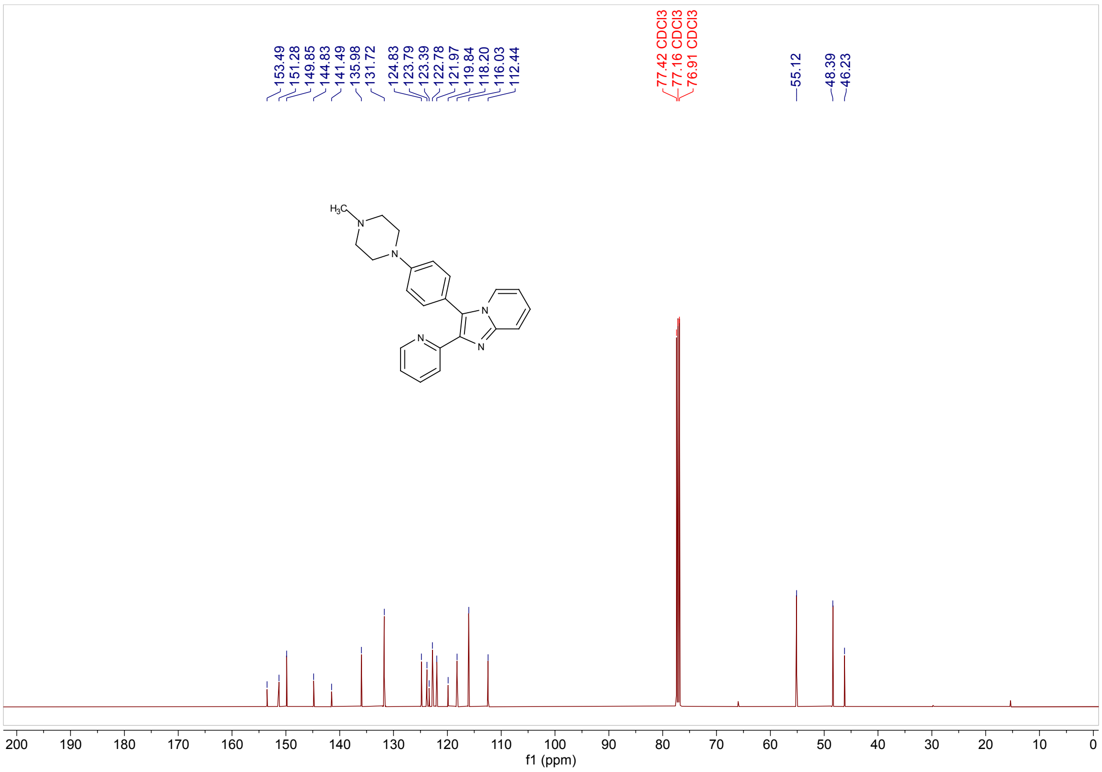

3-(4-Chlorophenyl)-2-(pyridin-2-yl)-6,7-dihydro-5*H*-pyrrolo[1,2-*a*]imidazole (OSA_000875)

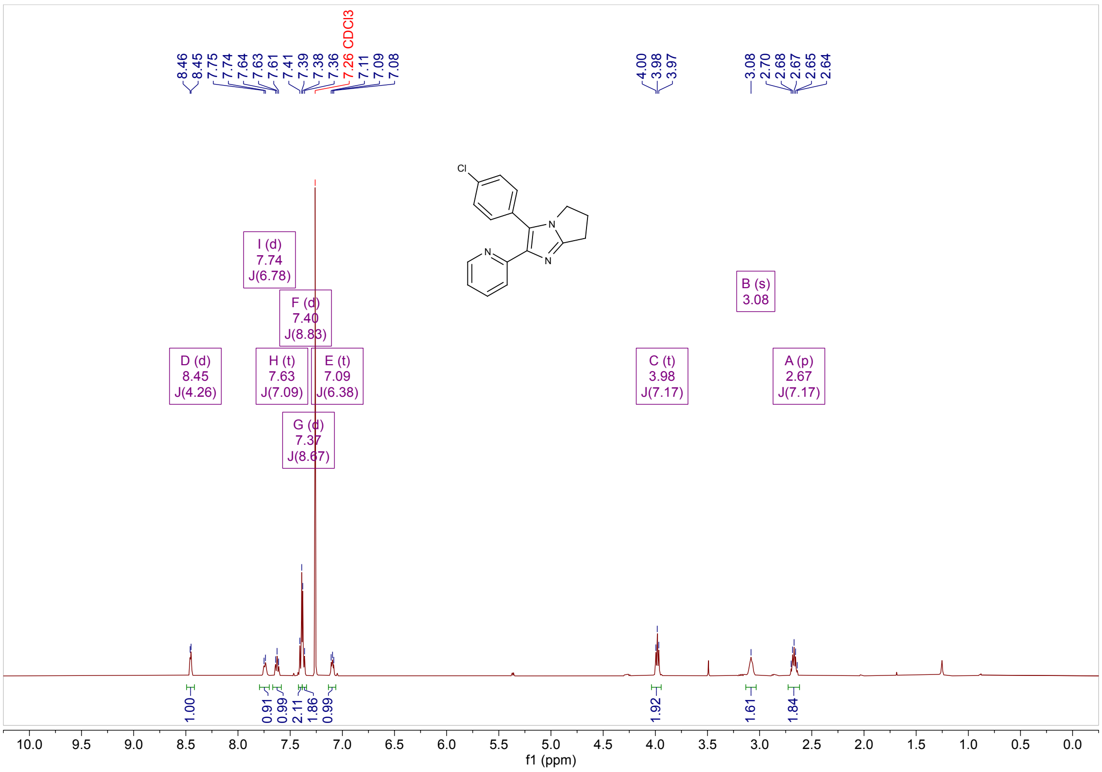

***N*-(4-Fluorophenyl)-2-(1*H*-pyrrol-2-yl)imidazo[1,2-*a*]pyridin-3-amine (OSA_001009)**

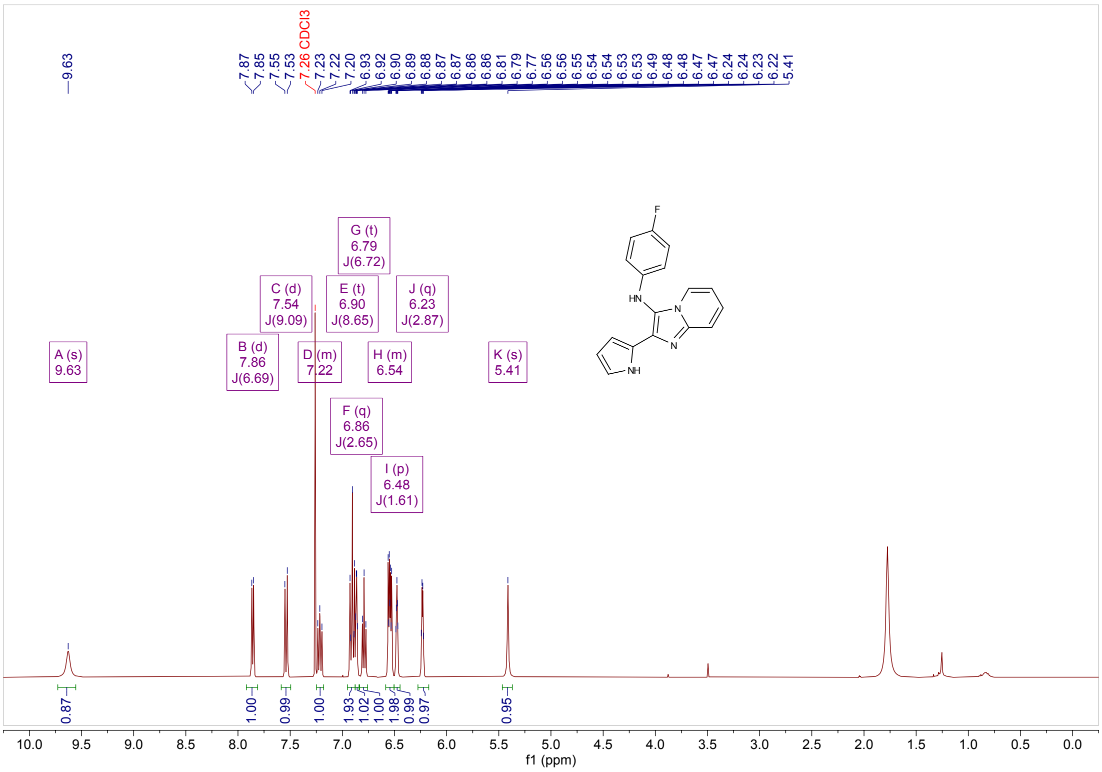

N-(4-Fluorophenyl)-2-(thiazol-2-yl)imidazo[1,2-*a*]pyridin-3-amine (OSA_001008)

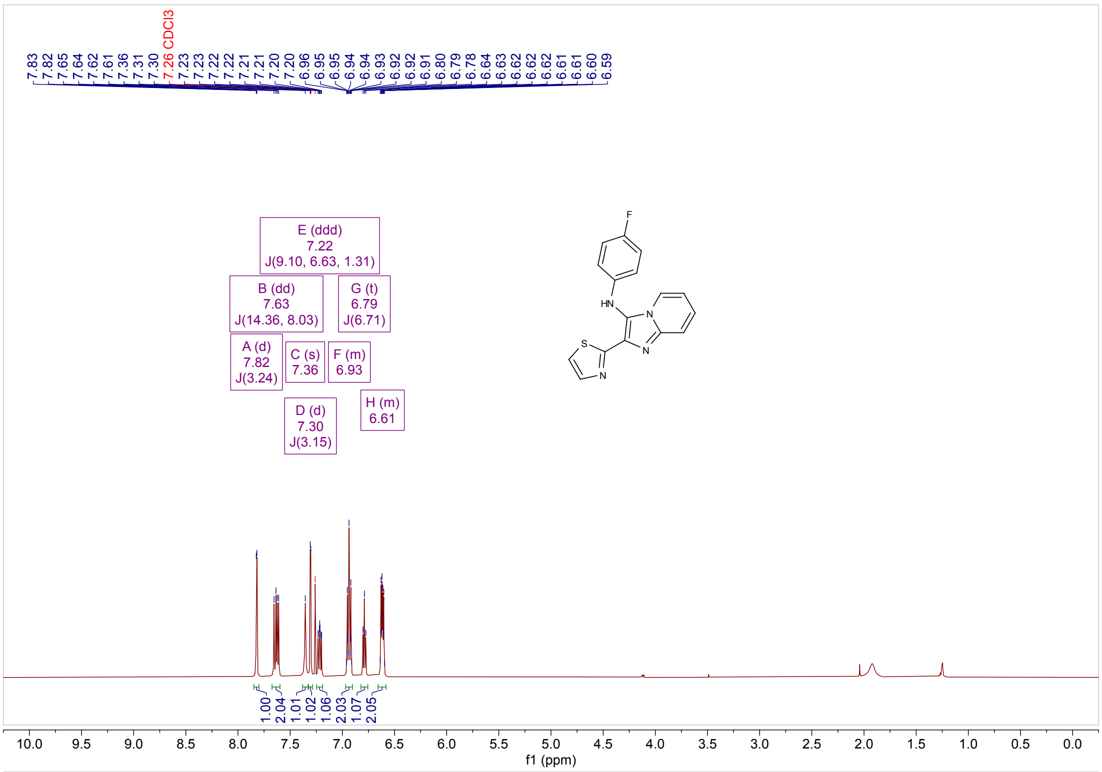

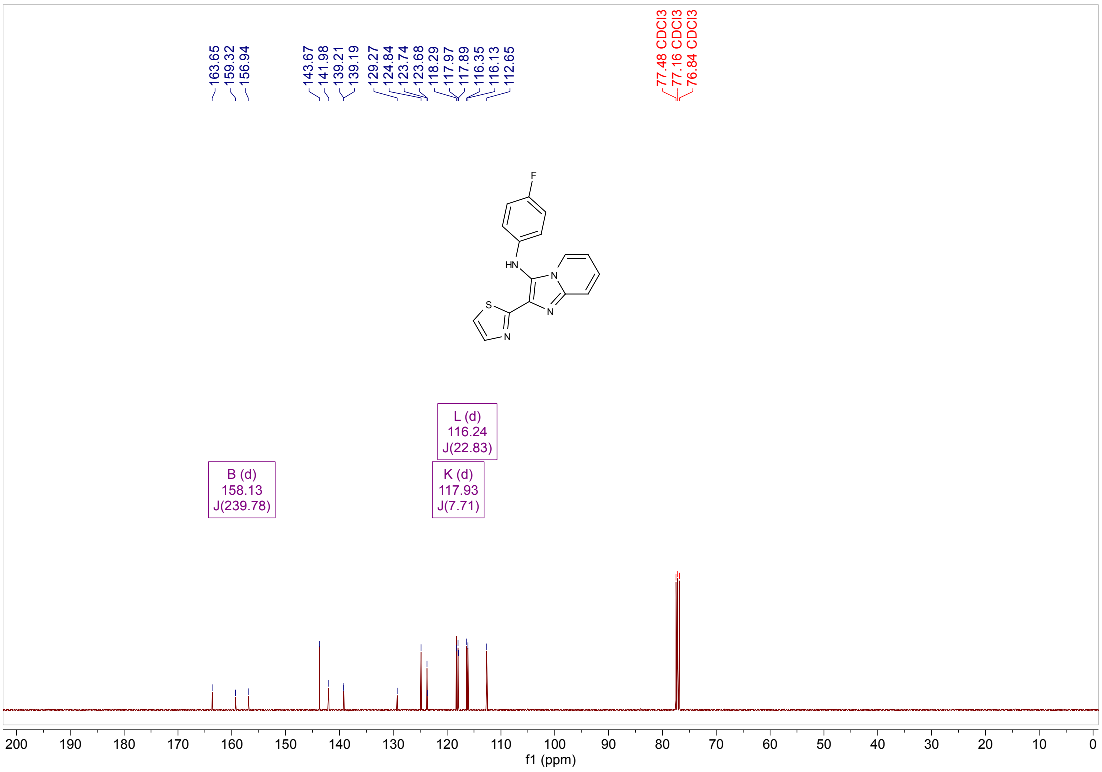

7-Chloro-2-(pyridin-2-yl)-3-(*p*-tolyl)imidazo[1,2-*a*]pyridine (OSA_000978)

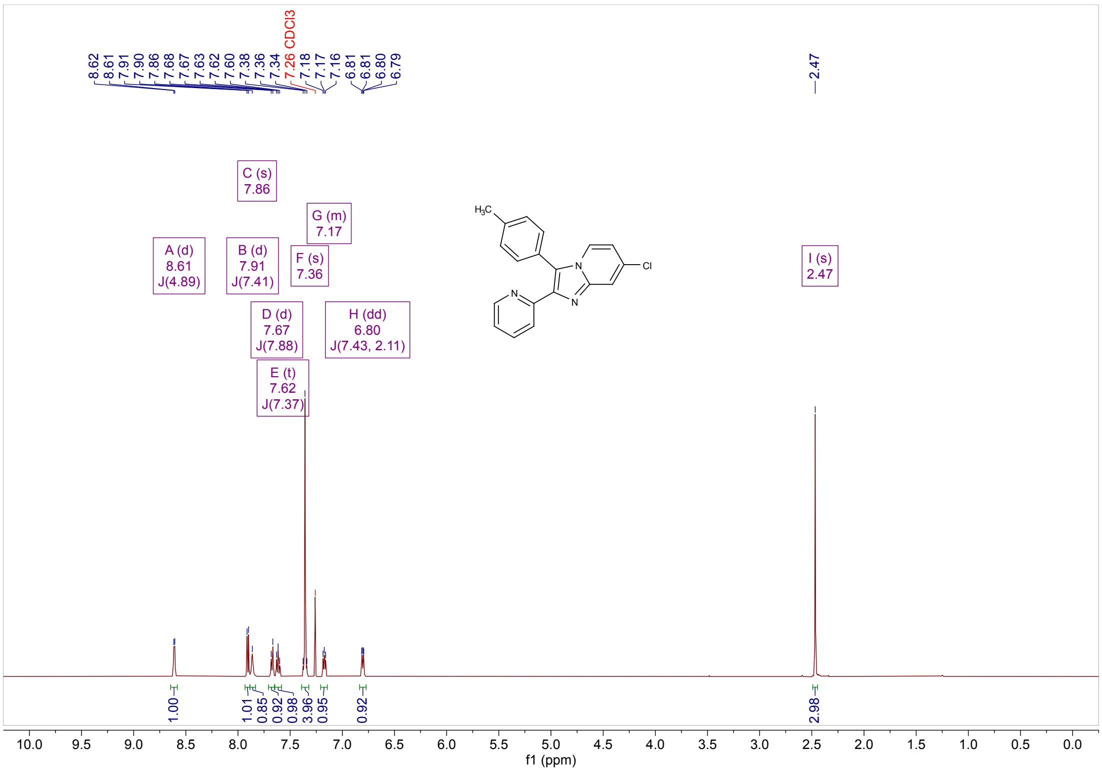

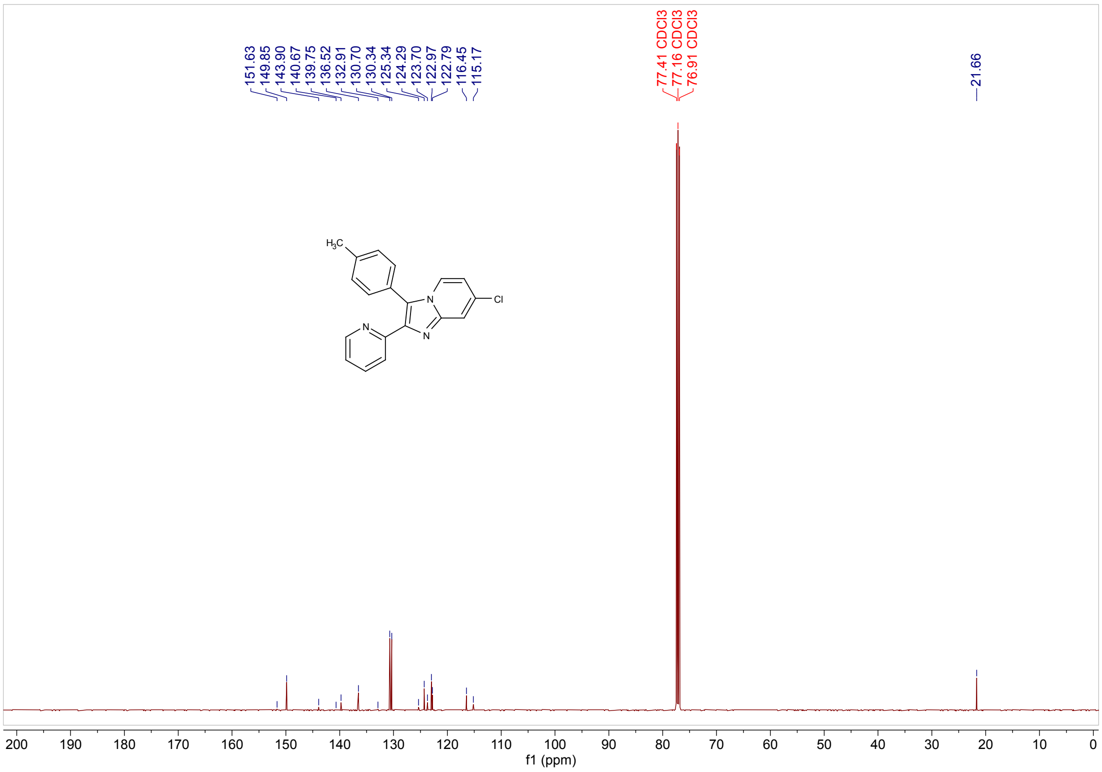

3-(Benzo[*b*]thiophen-5-yl)-2-phenyl-6,7-dihydro-5*H*-pyrrolo[1,2-*a*]imidazole

(OSA_000834)

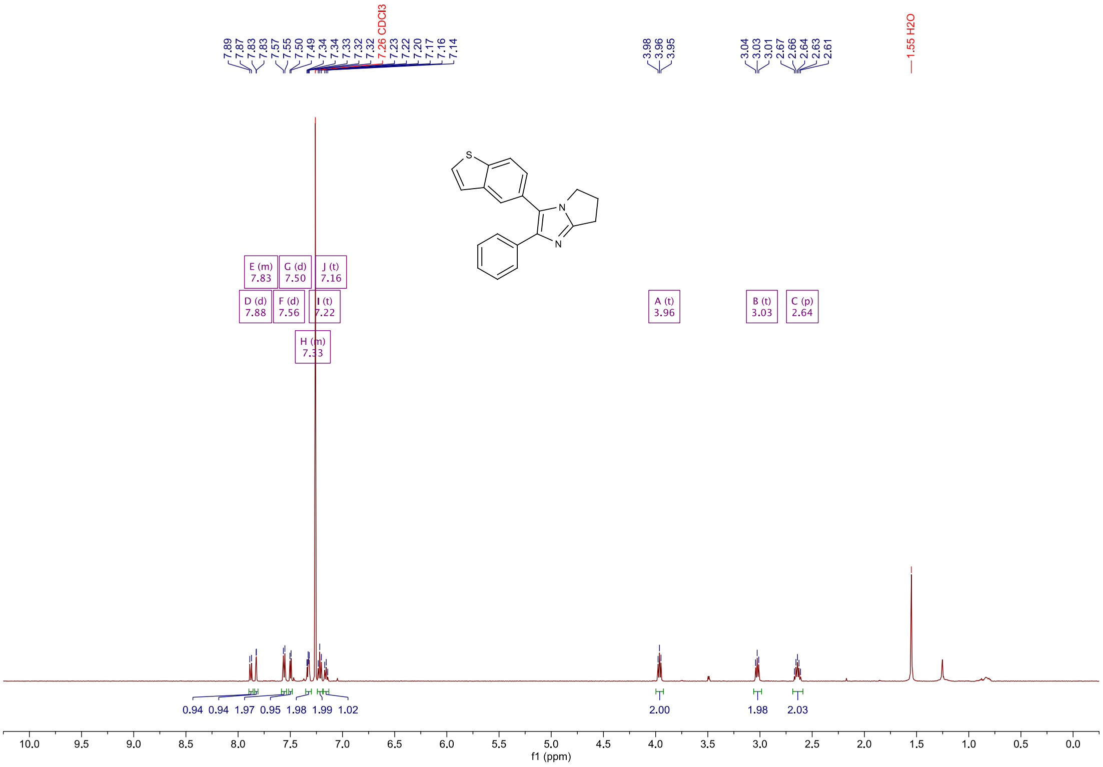

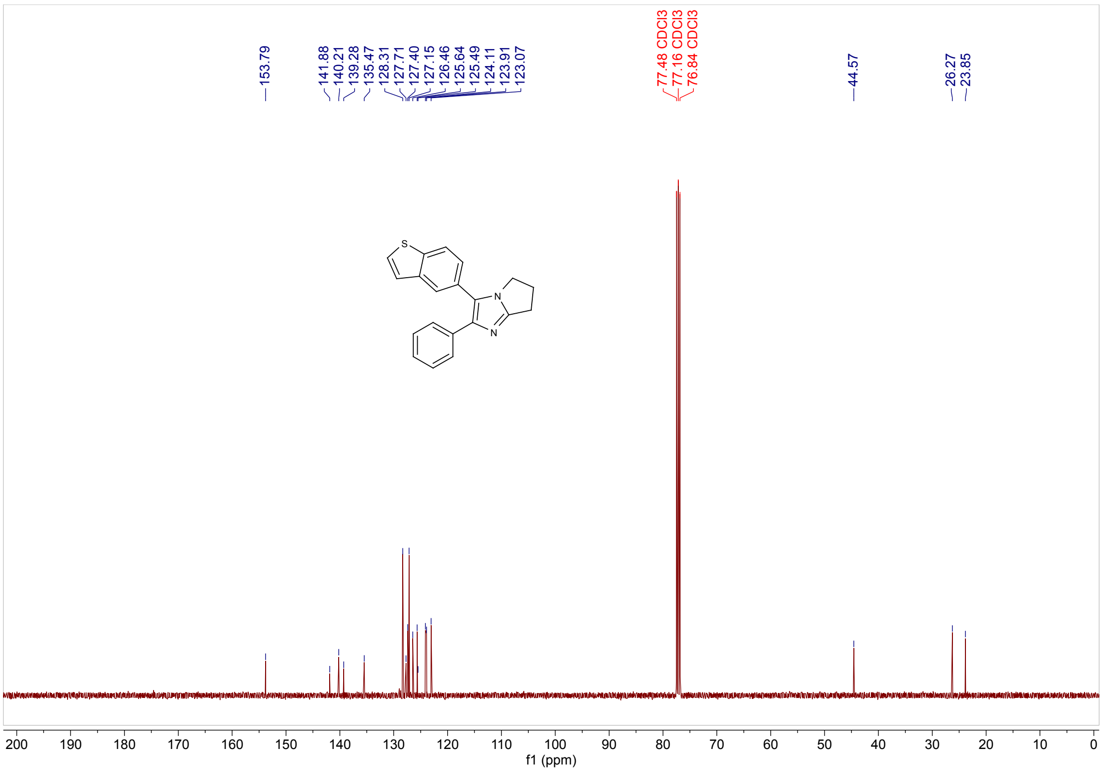

3-(4-Fluorophenyl)-2-(pyridin-2-yl)-6,7-dihydro-5*H*-pyrrolo[1,2-*a*]imidazole

(OSA_001053)

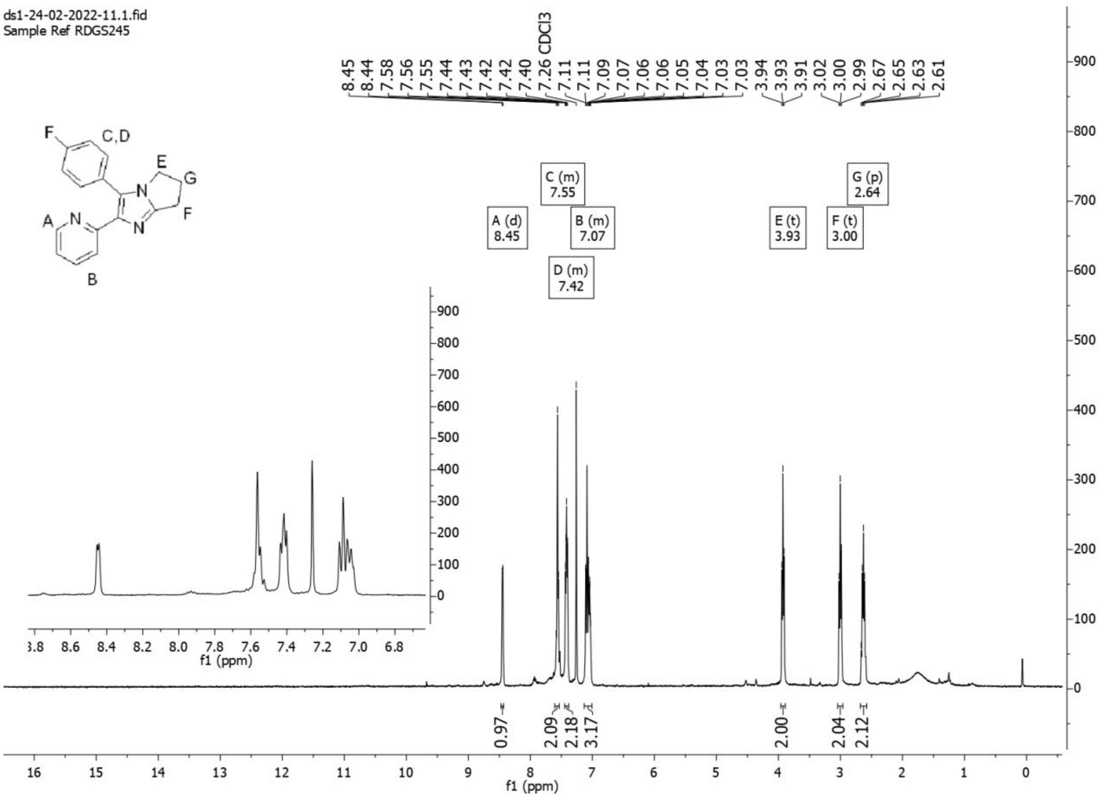

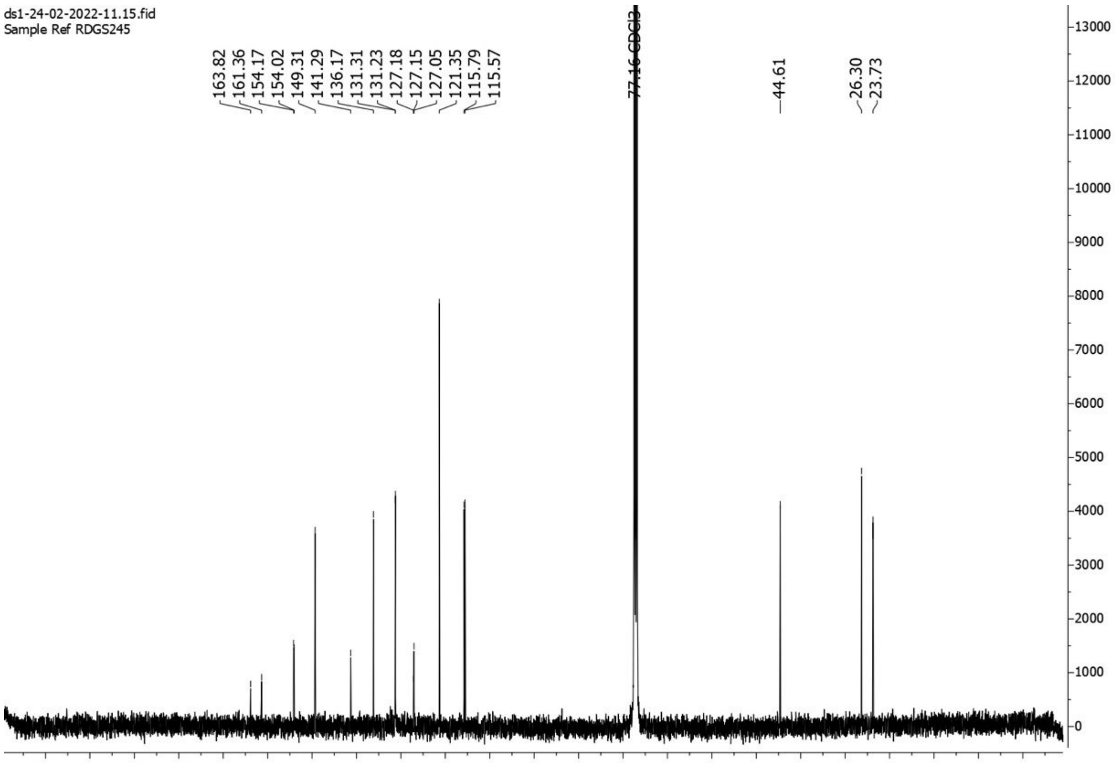

2-(Pyridin-2-yl)-3-(pyridin-4-yl)-6,7-dihydro-5*H*-pyrrolo[1,2-*a*]imidazole (OSA_001072)

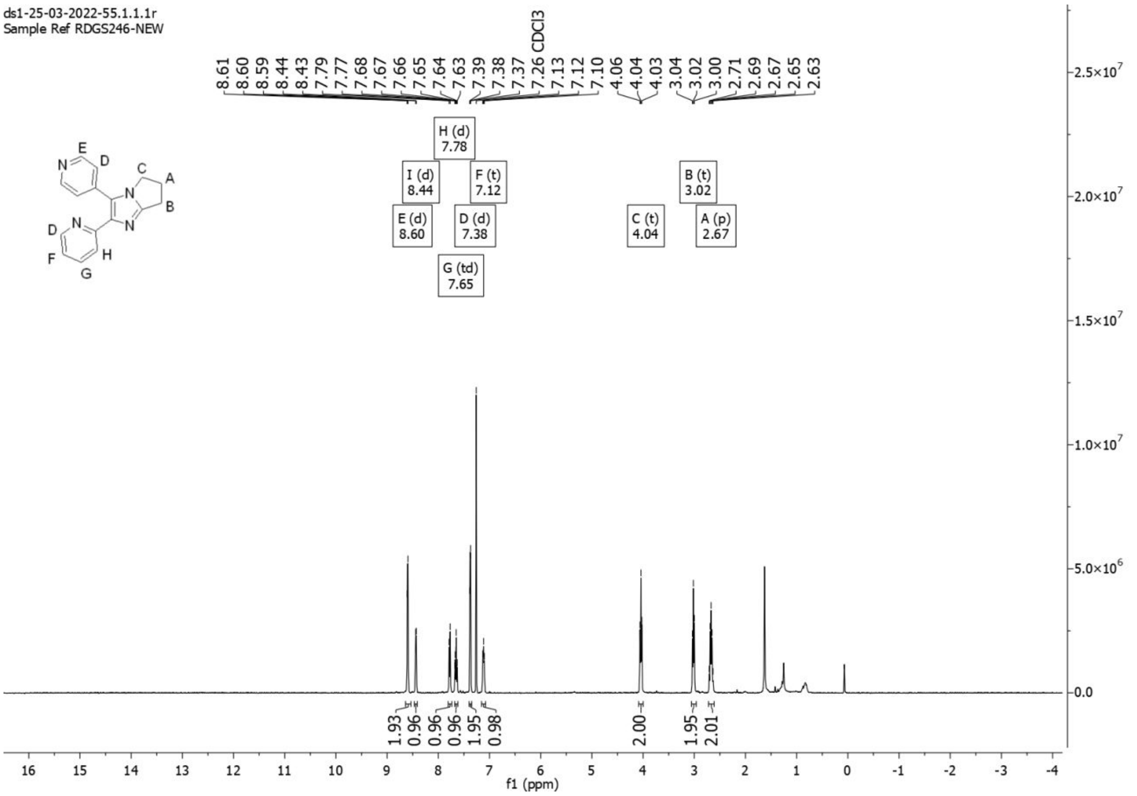

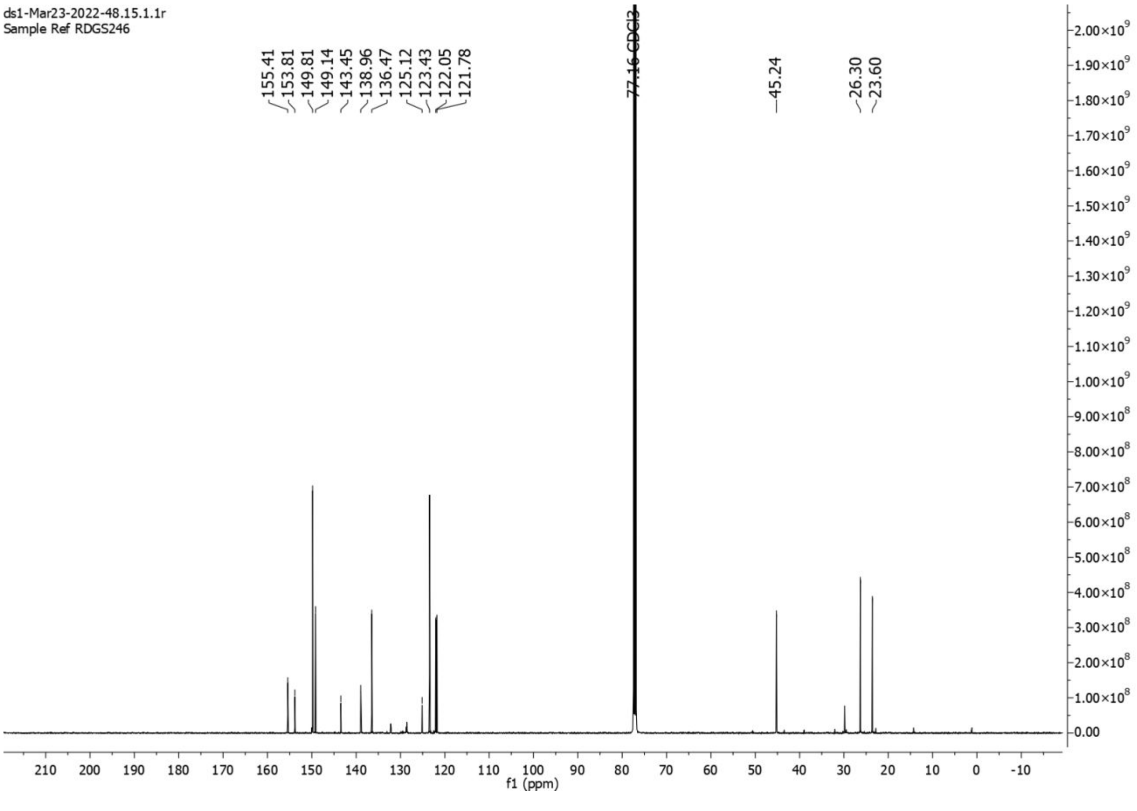

3-(2-Fluoropyridin-4-yl)-2-(pyridin-2-yl)-6,7-dihydro-5*H*-pyrrolo[1,2-*a*]imidazole

(OSA_001073)

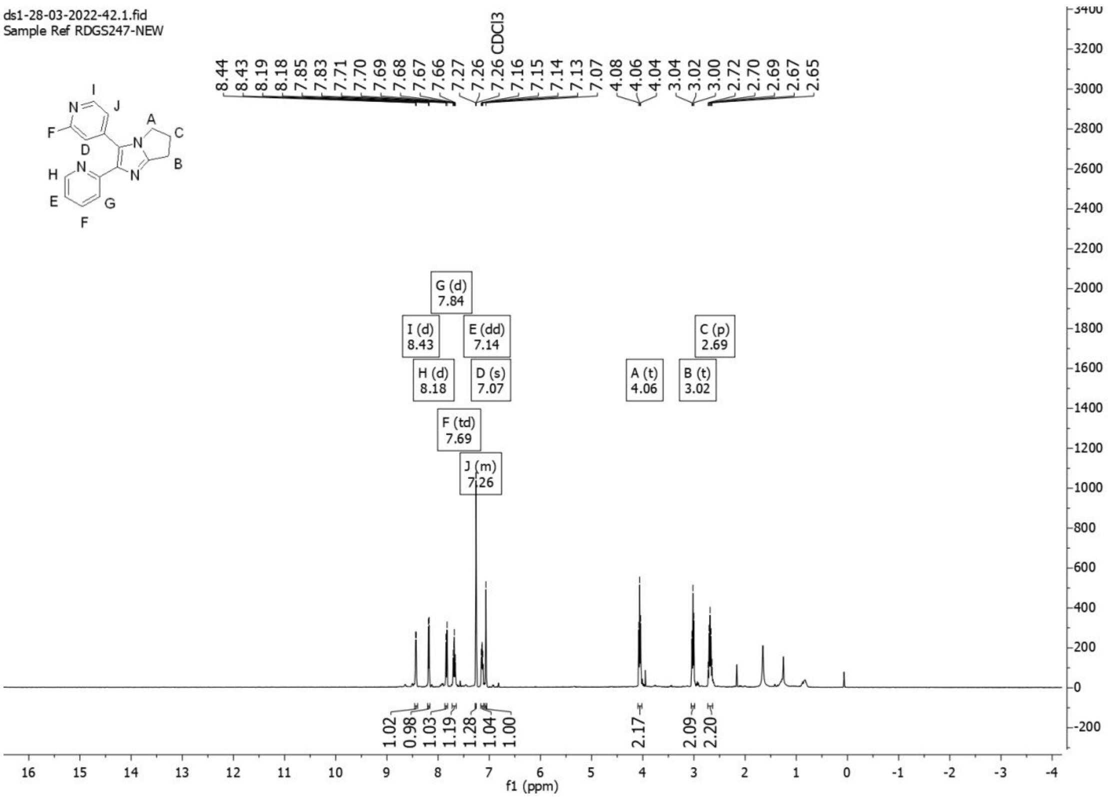

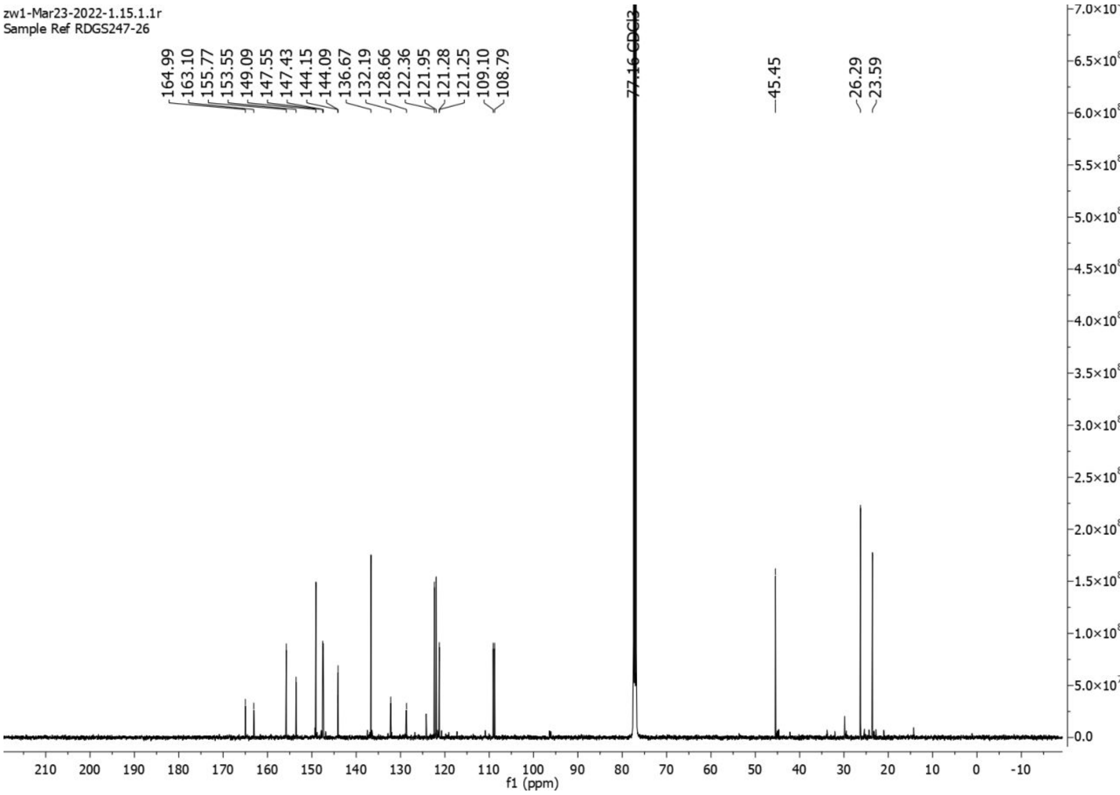

4-(2-(Pyridin-2-yl)-6,7-dihydro-5*H*-pyrrolo[1,2-*a*]imidazol-3-yl)aniline (OSA_001074)

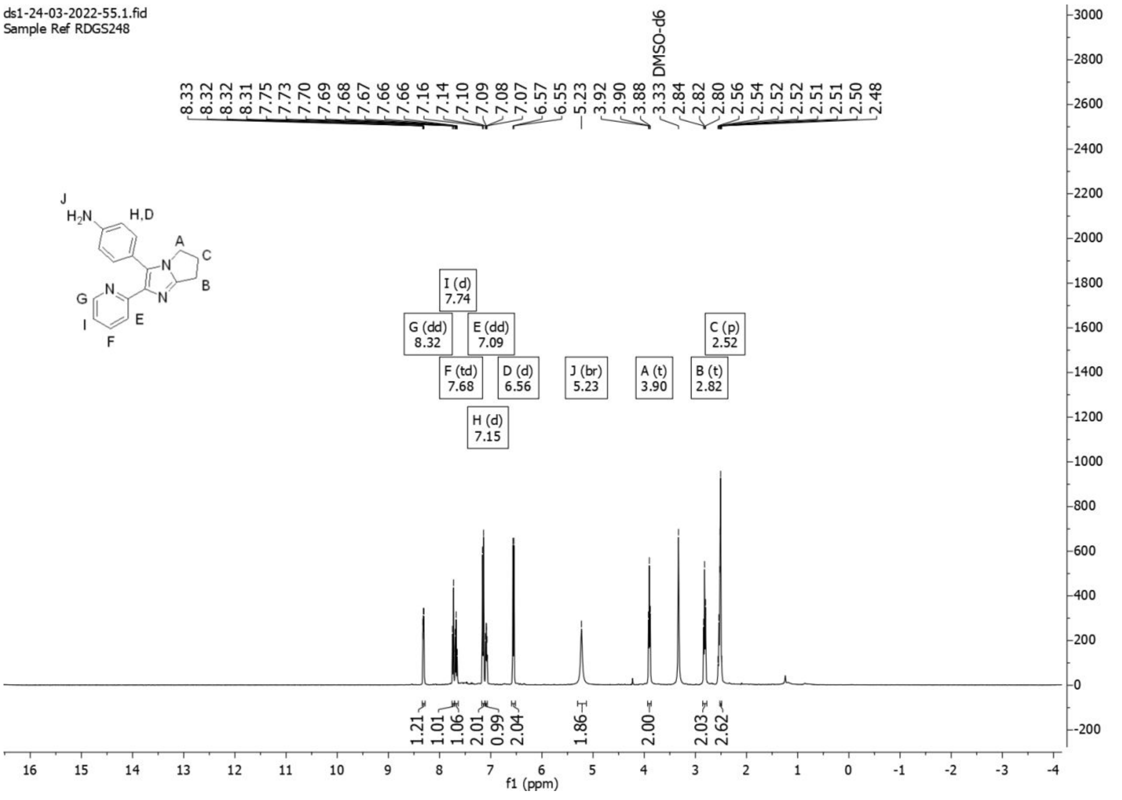

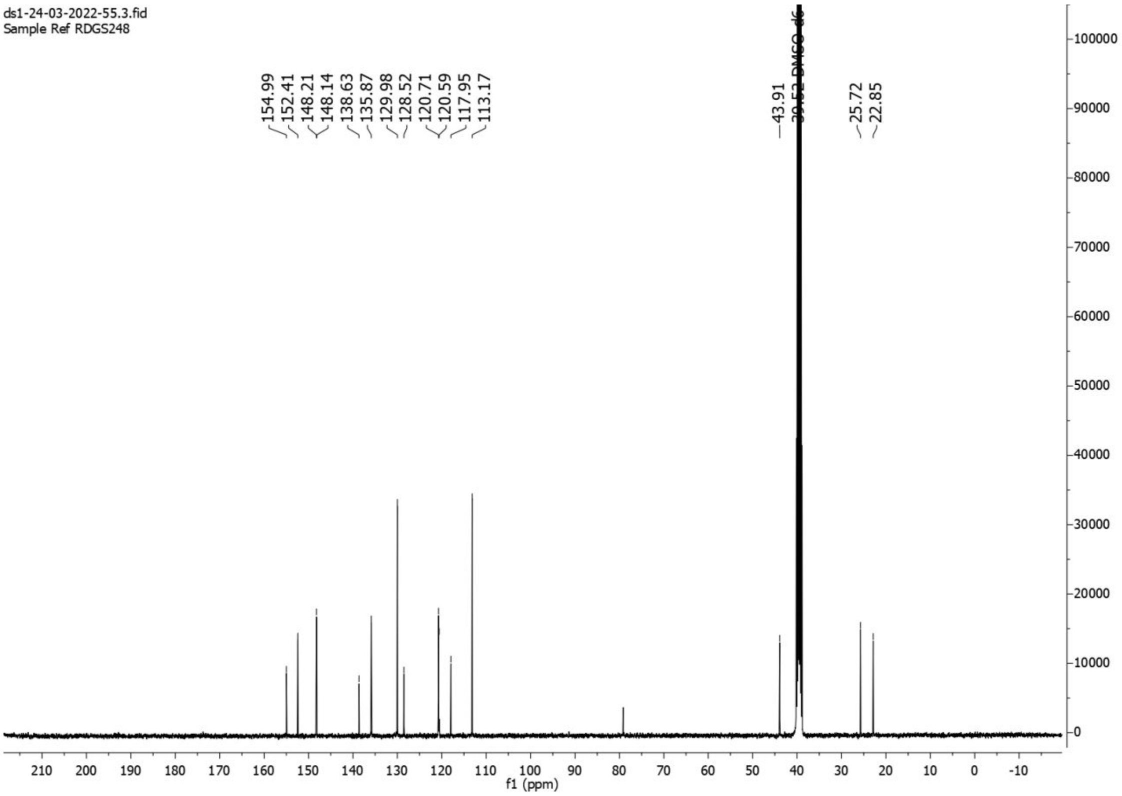

Mono(3-(6-cyanopyridin-1-ium-3-yl)-2-(pyridin-2-yl)-6,7-dihydro-5*H*-pyrrolo[1,2-*a*]imidazol-1-ium) monoamide (OSA_001075)

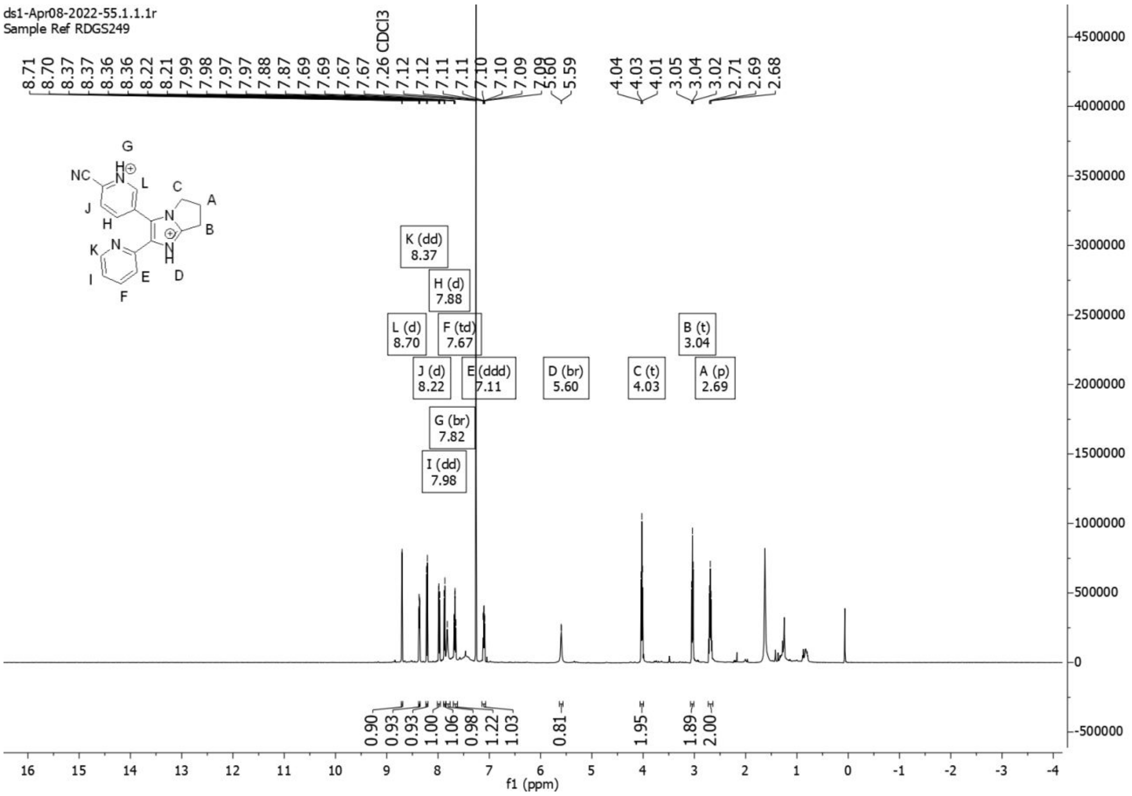

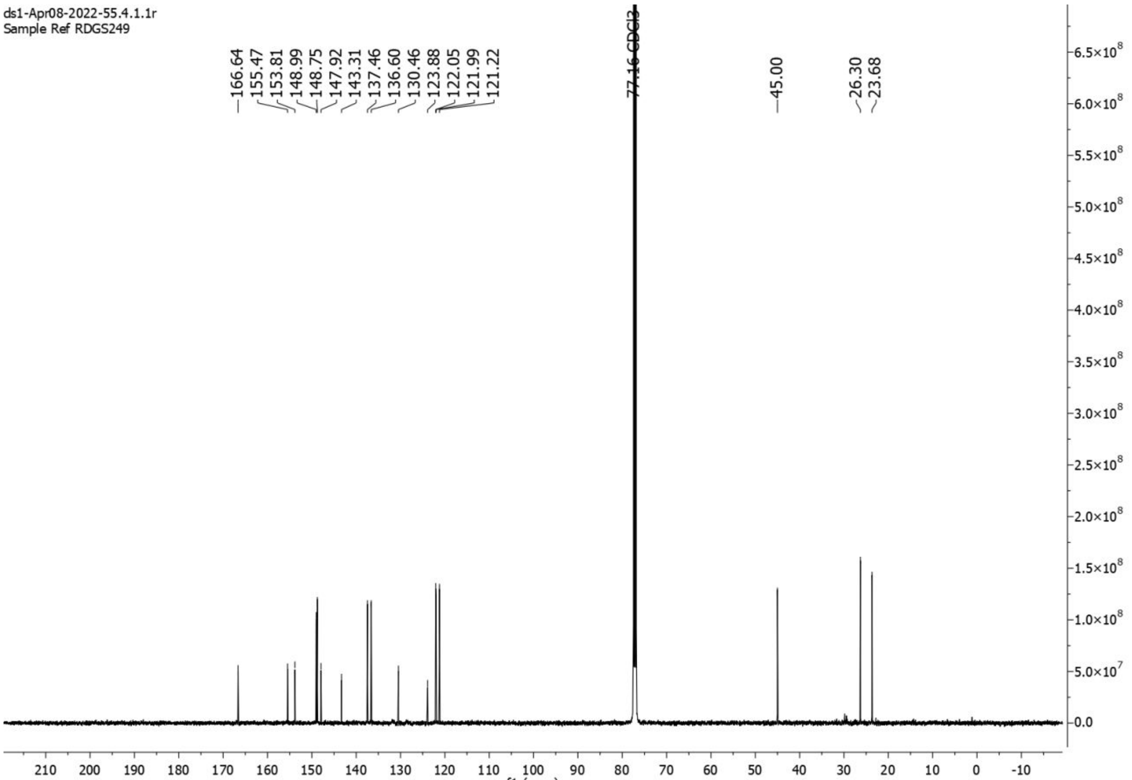

2-Methoxy-4-(2-(pyridin-2-yl)-6,7-dihydro-5*H*-pyrrolo[1,2-*a*]imidazol-3-yl)benzonitrile (OSA_001076)

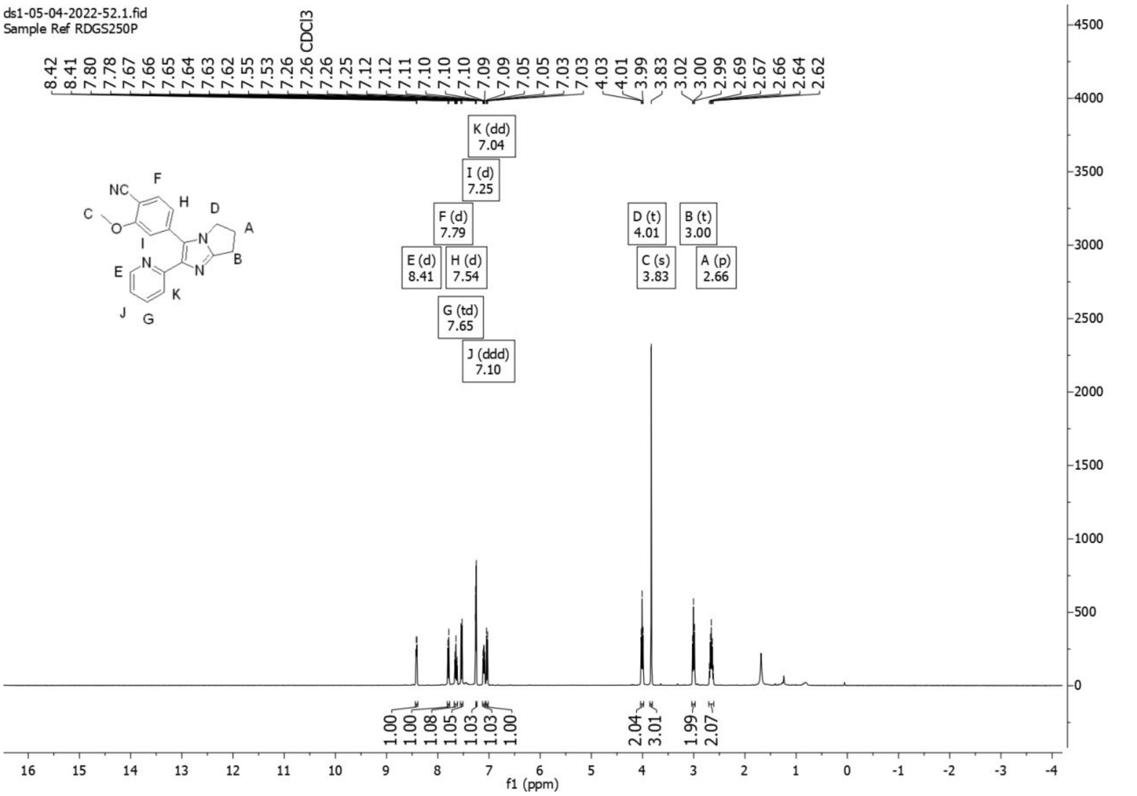

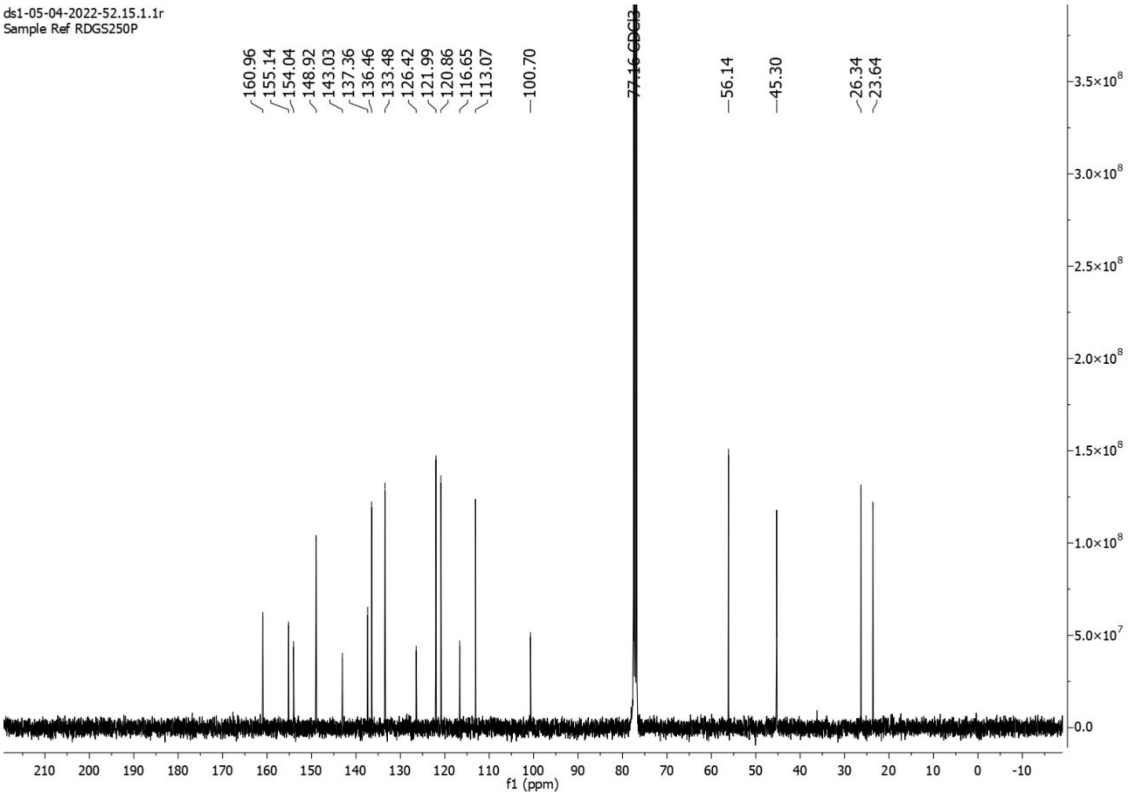

4-(2-(Pyridin-2-yl)-6,7-dihydro-5*H*-pyrrolo[1,2-*a*]imidazol-3-yl)-2-(trifluoromethyl)benzonitrile (OSA_001077)

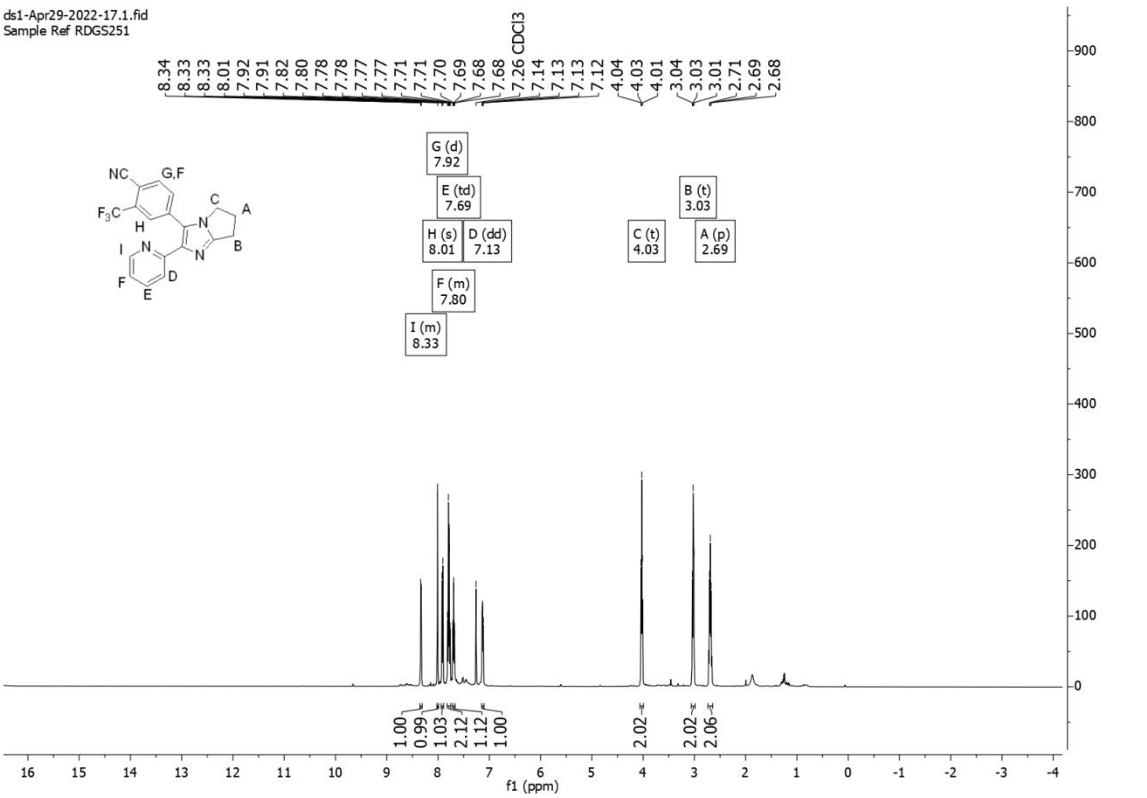

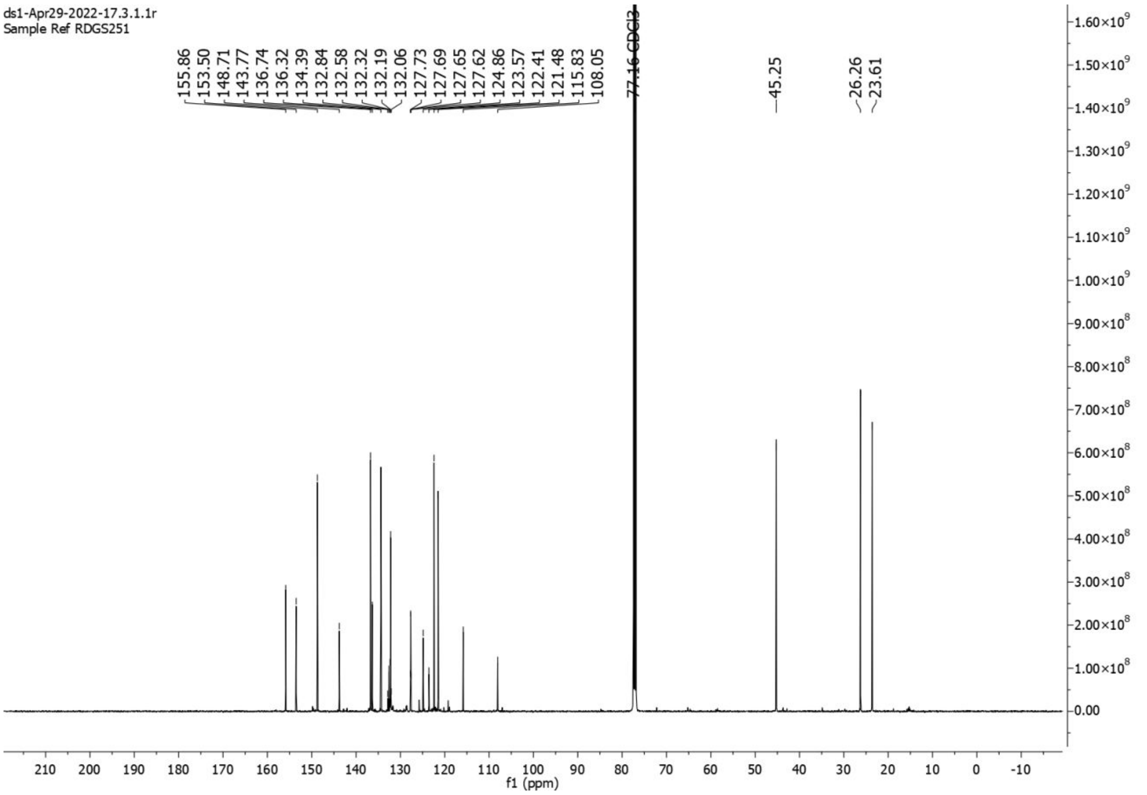

3-(Benzo[*d*][1,3]dioxol-5-yl)-2-(pyridin-3-yl)-6,7-dihydro-5*H*-pyrrolo[1,2-*a*]imidazole (OSA_000816)

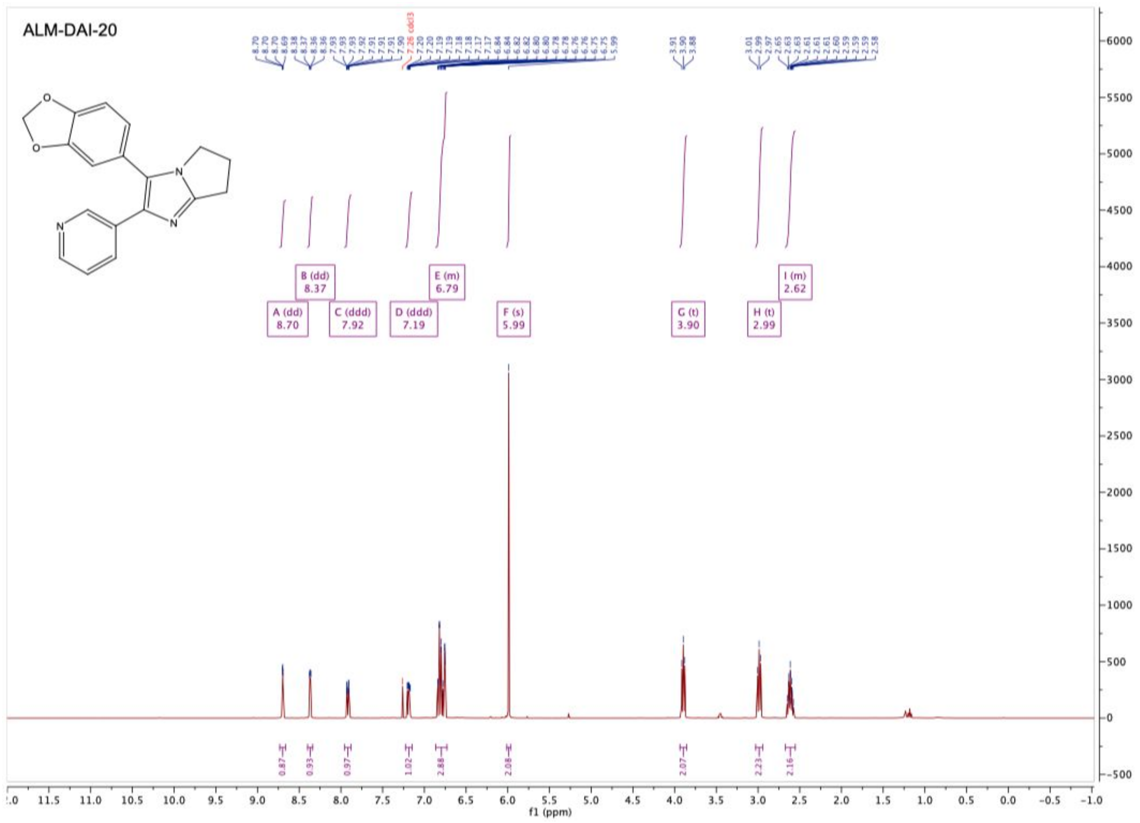

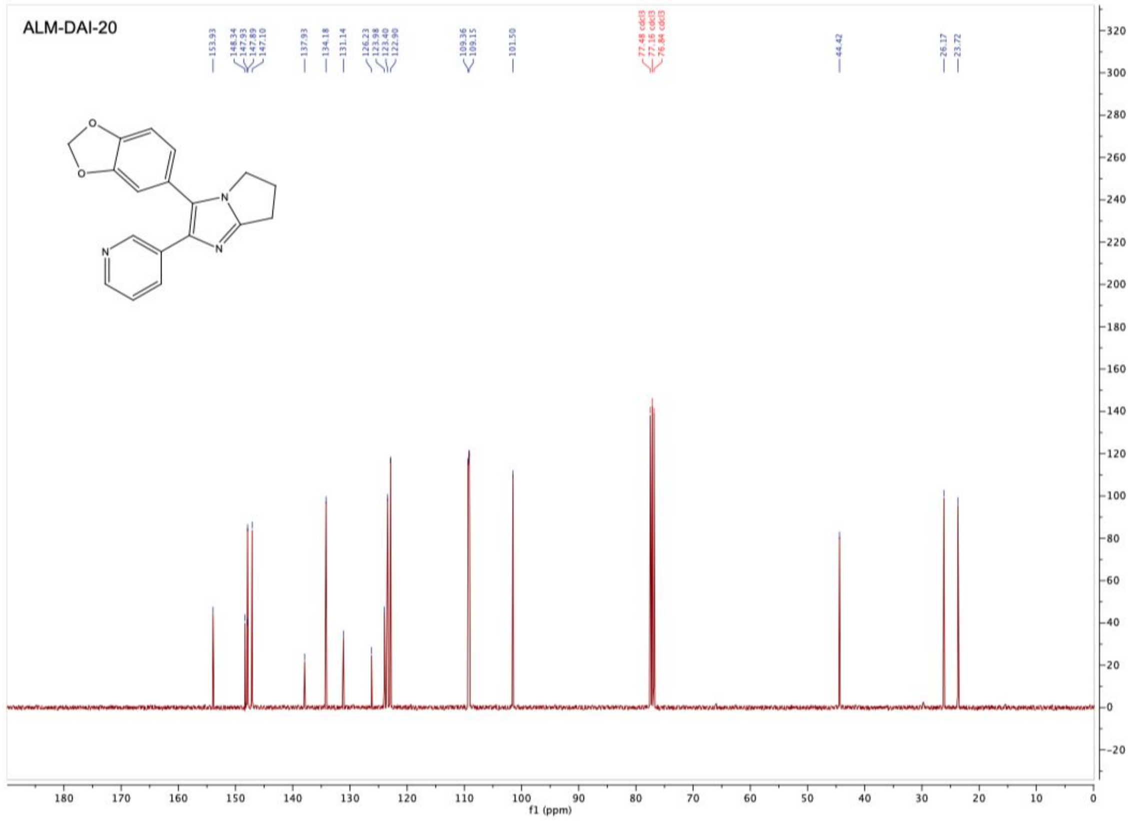

3-(Benzo[*d*][1,3]dioxol-5-yl)-2-(pyridin-4-yl)-6,7-dihydro-5*H*-pyrrolo[1,2-*a*]imidazole

(OSA_000817)

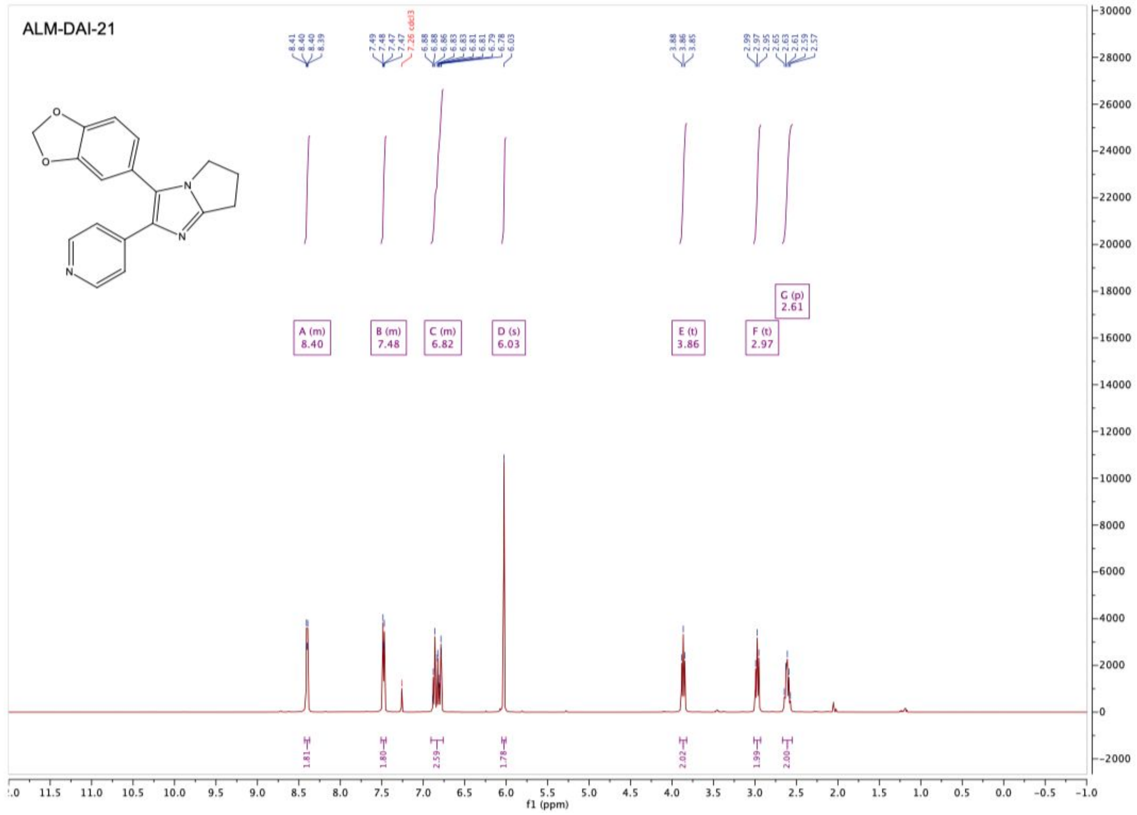

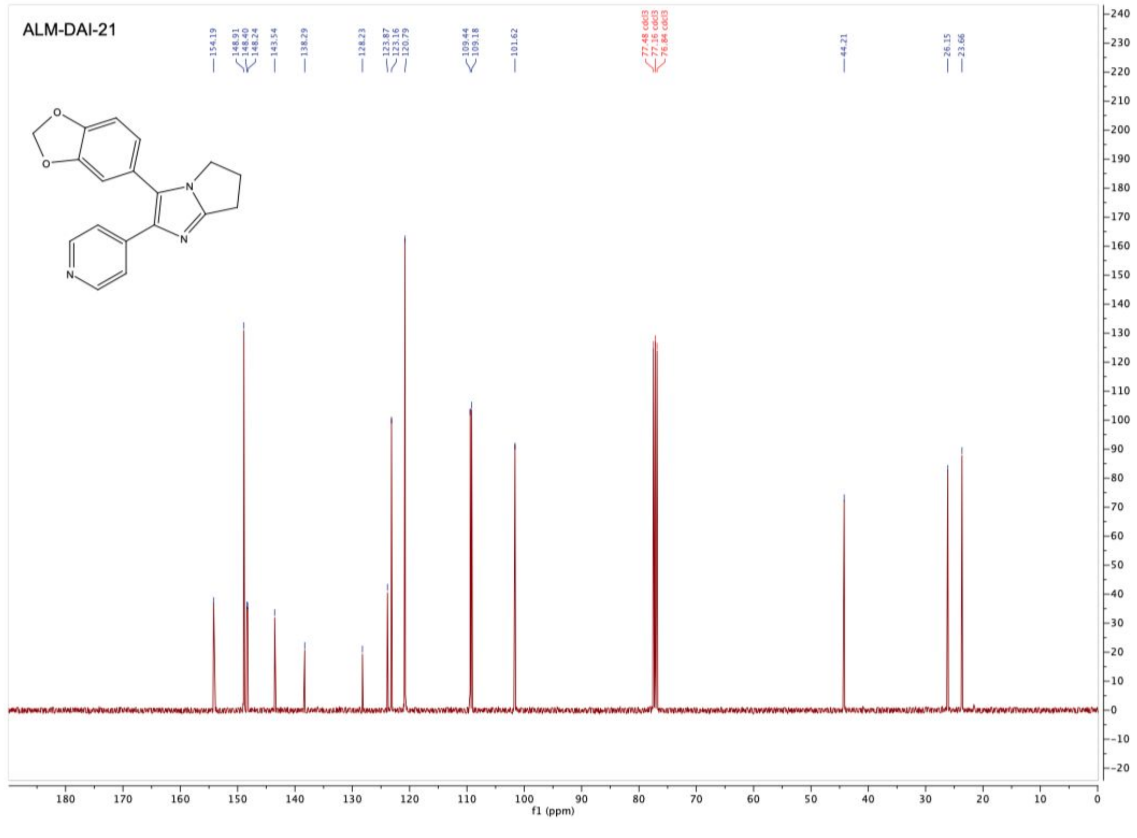

5-(Benzo[*d*][1,3]dioxol-5-yl)-6-(pyridin-2-yl)-2,3-dihydroimidazo[2,1-*b*]thiazole (OSA_000813)

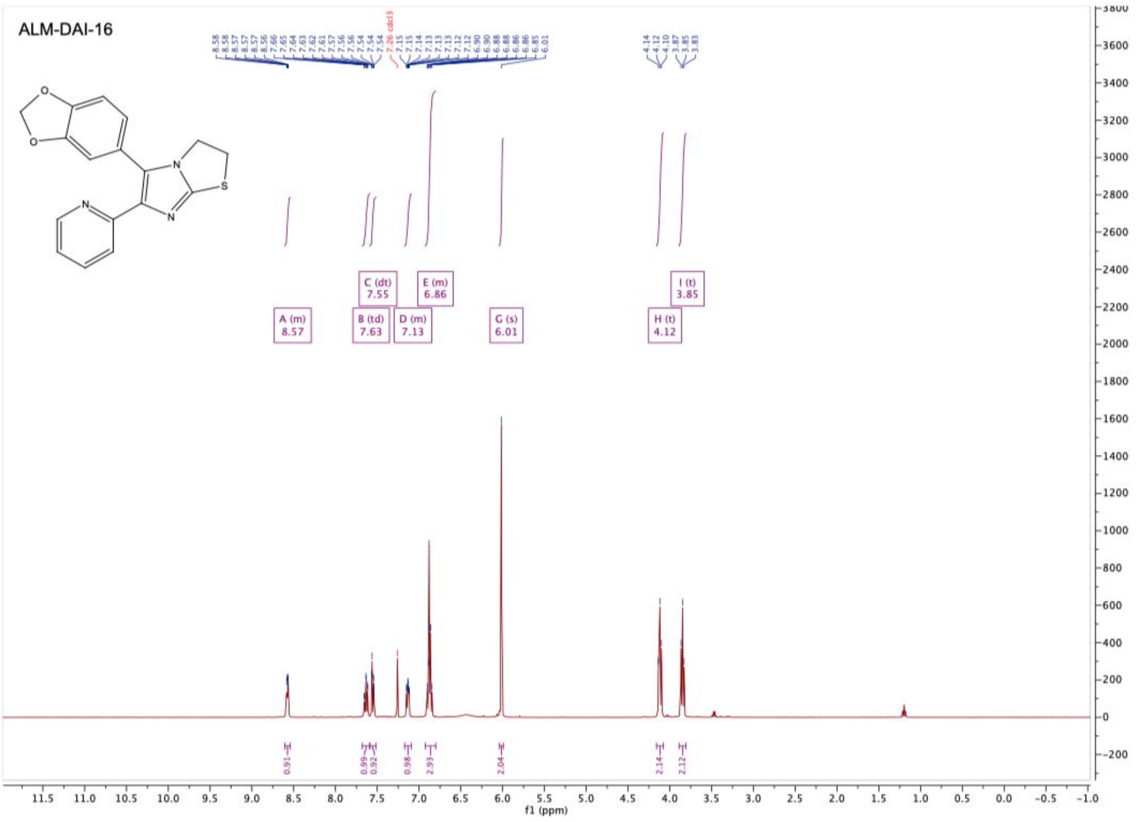

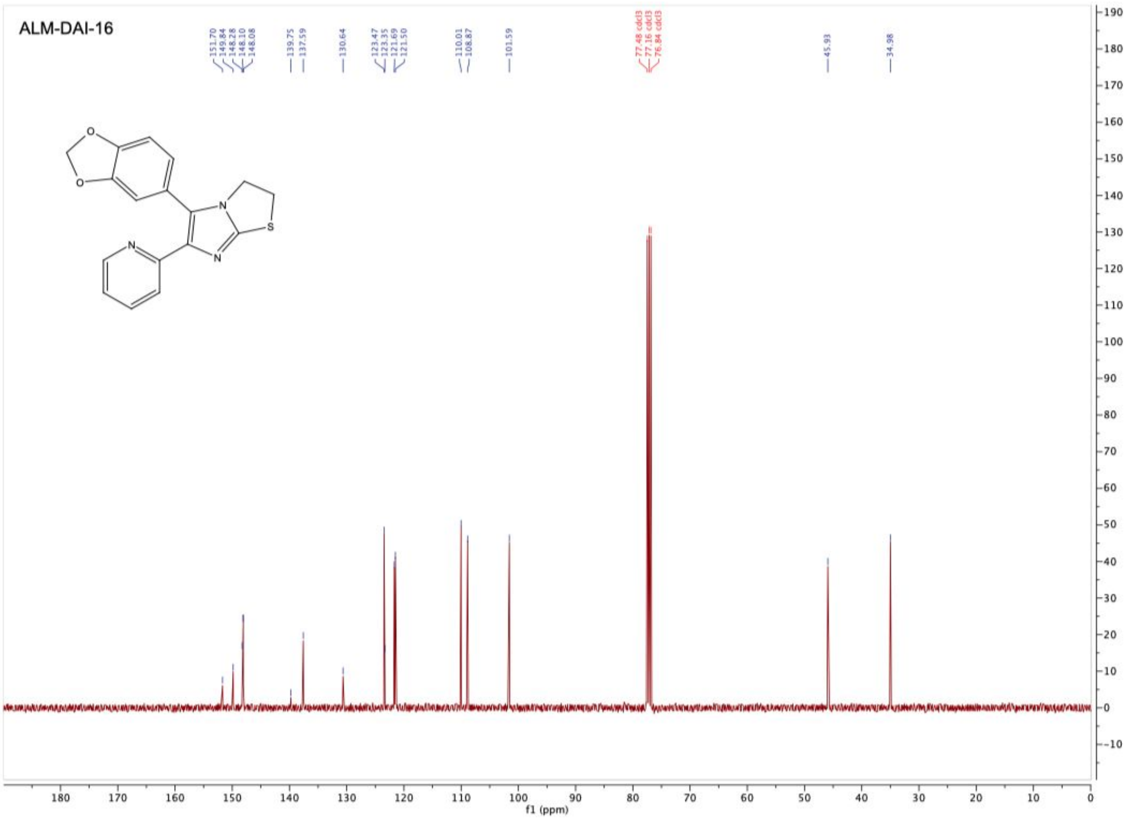

3-(Benzo[*d*][1,3]dioxol-5-yl)-2-(pyridin-2-yl)-5,6,7,8-tetrahydroimidazo[1,2-*a*]pyridine (OSA_000818)

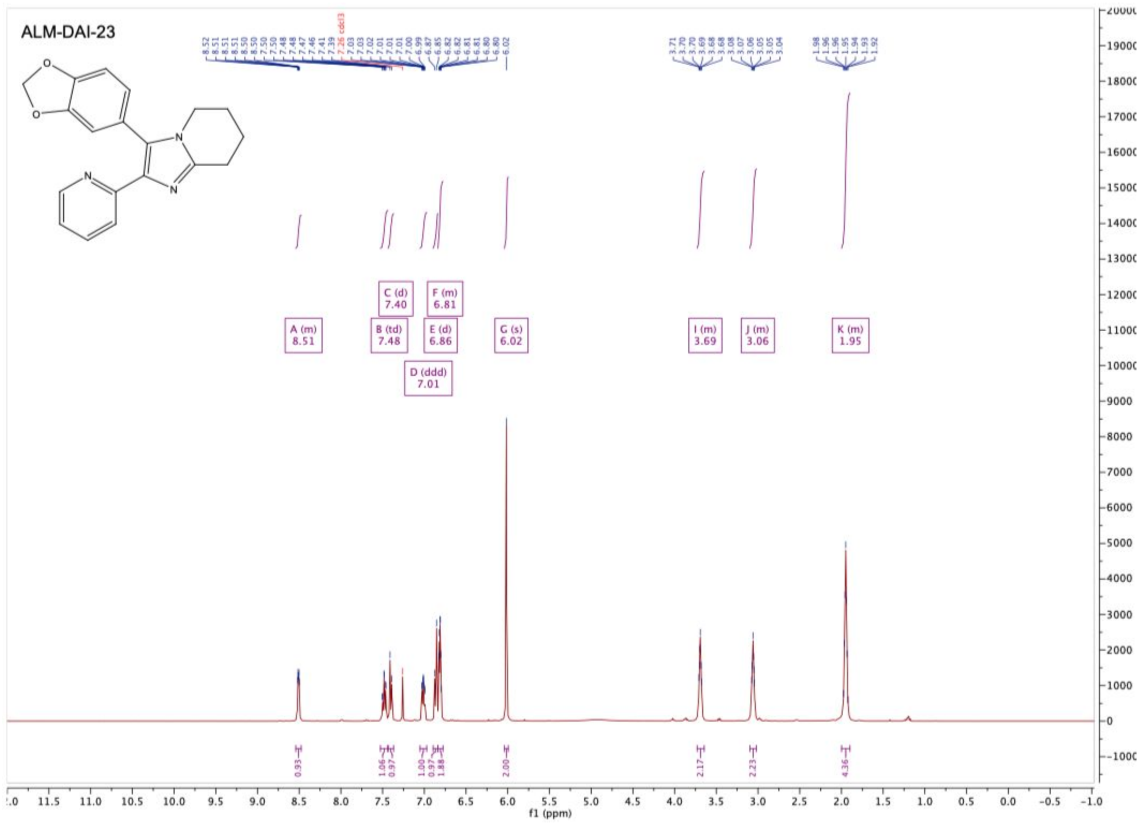

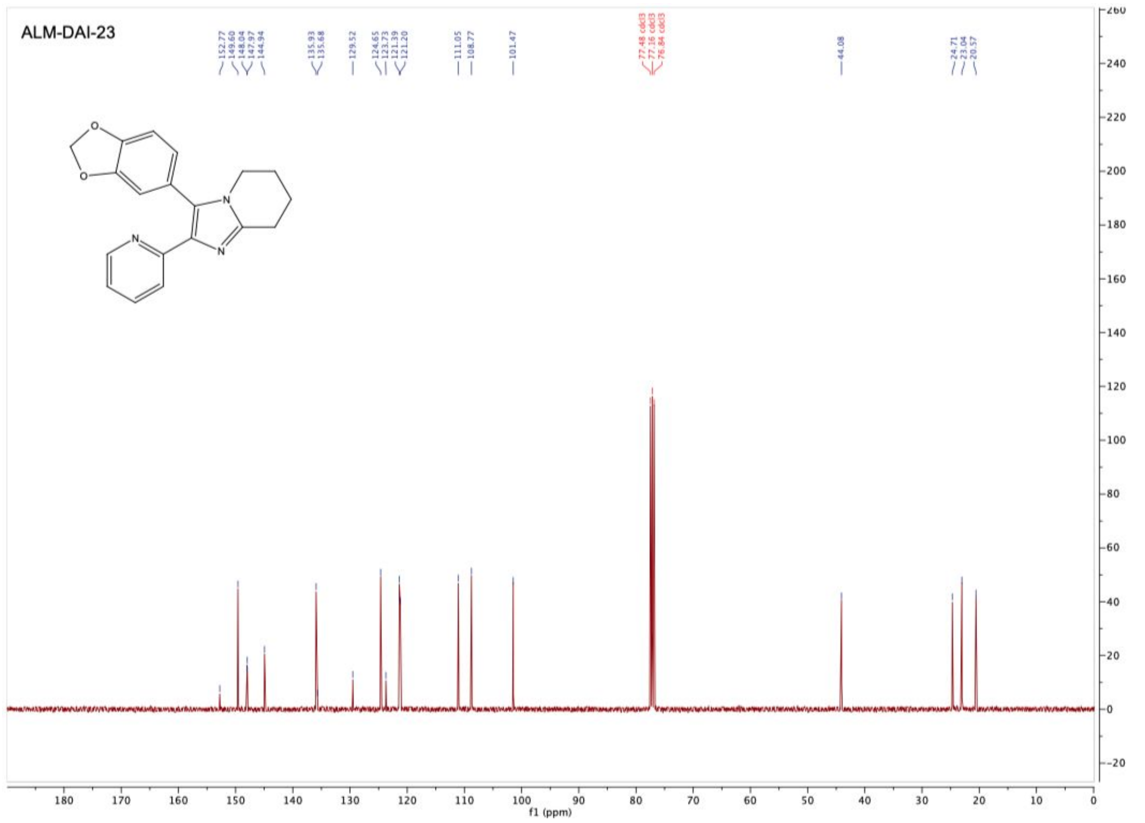

3-(Benzofuran-5-yl)-7-chloro-2-(pyridin-2-yl)imidazo[1,2-*a*]pyridine (OSA_000985)

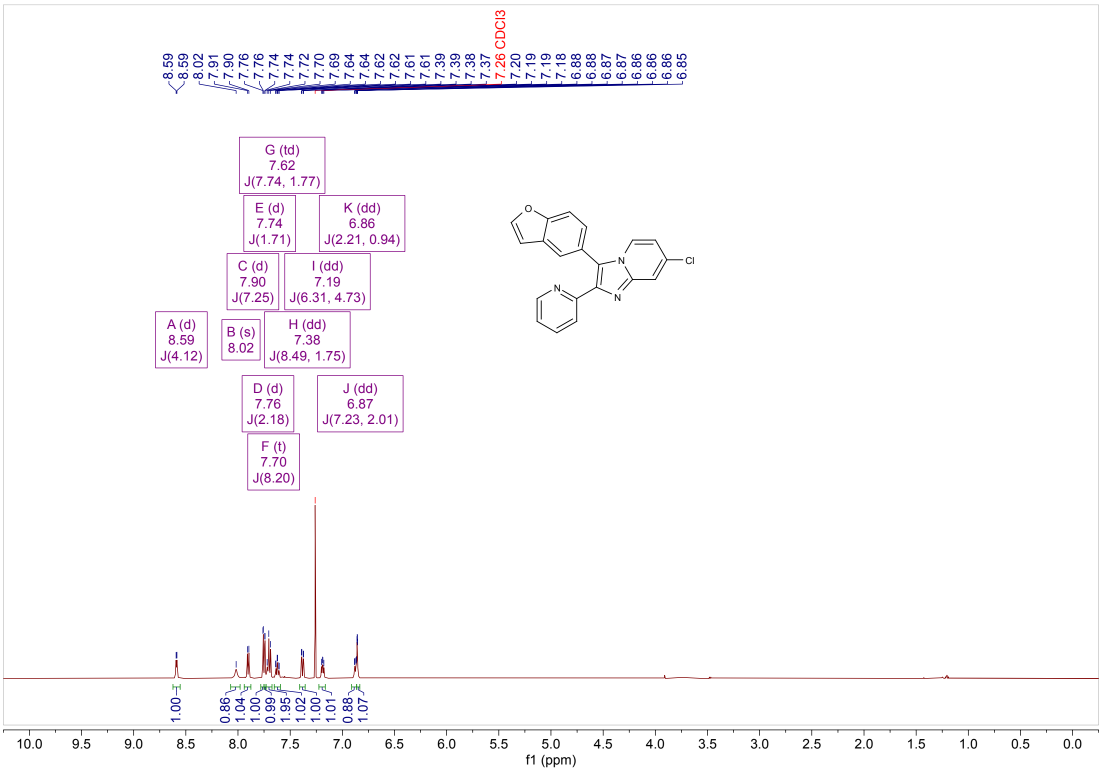

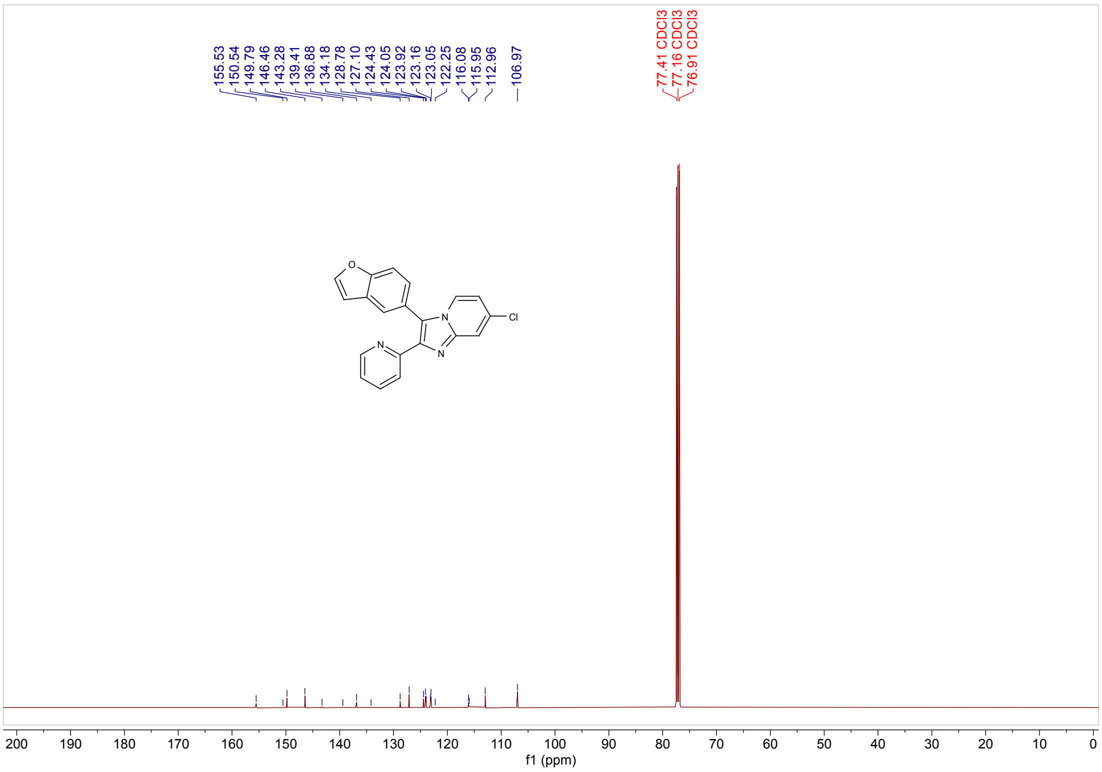

2-(Pyridin-2-yl)-3-(*p*-tolyl)imidazo[1,2-*a*]pyridine-7-carbonitrile (OSA_001013)

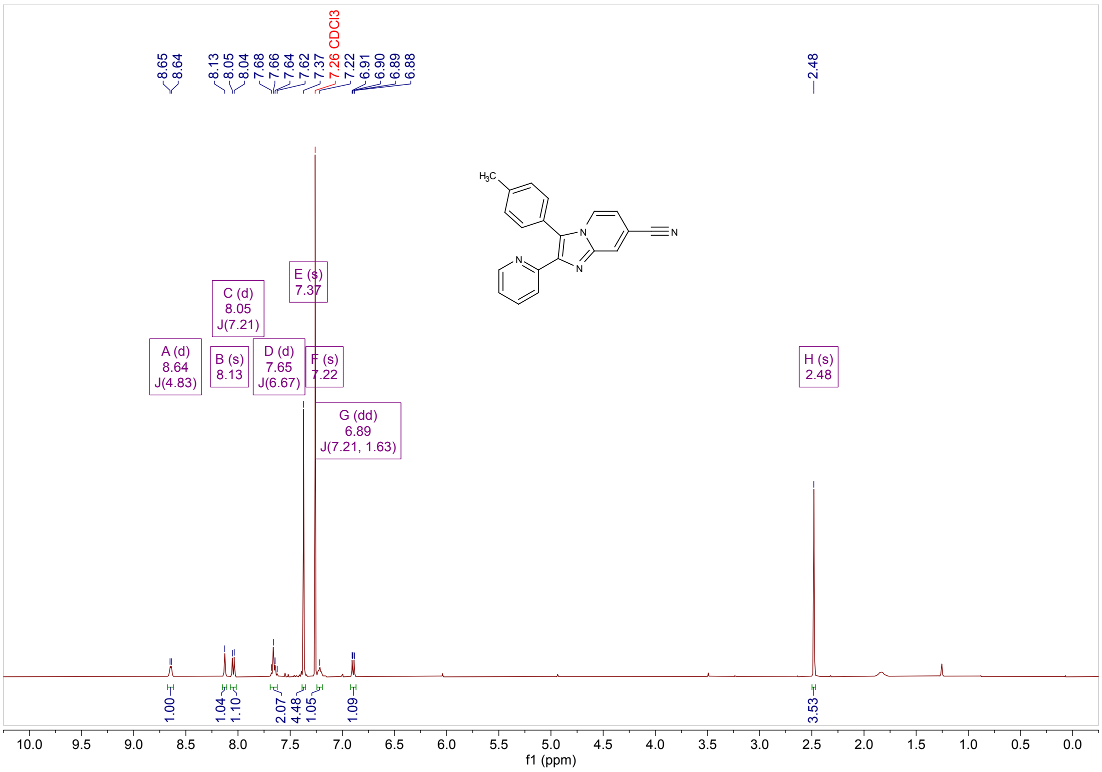

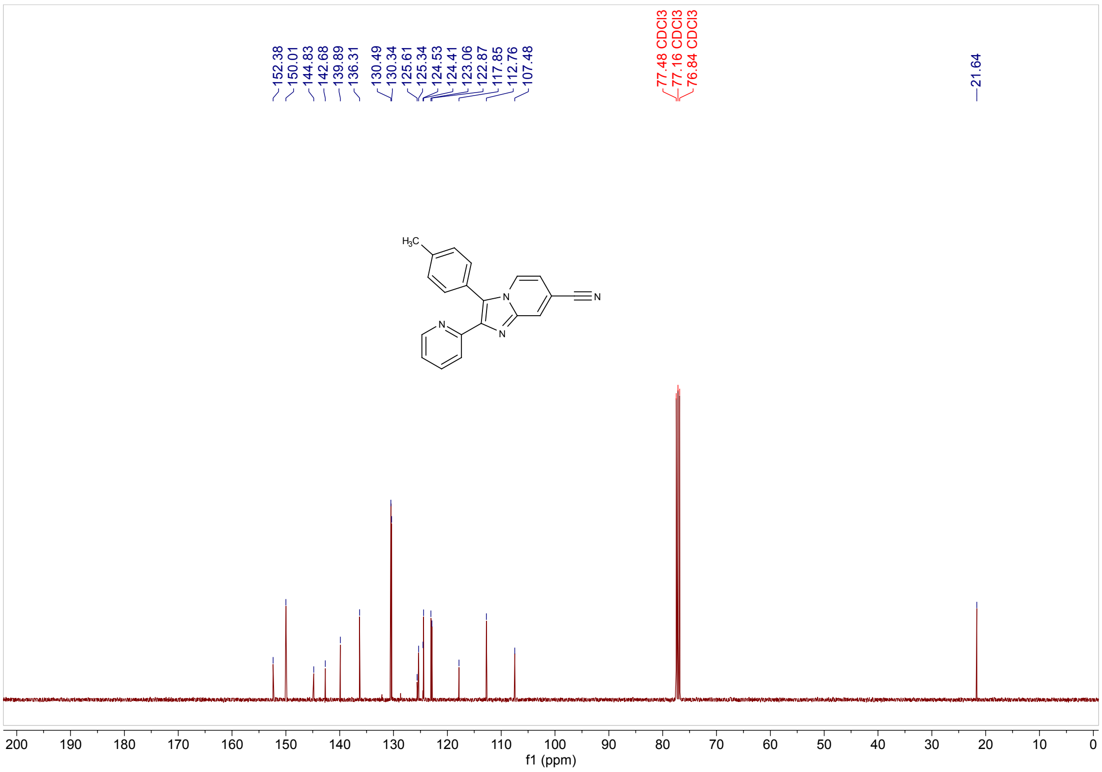

2-(Pyridin-2-yl)-3-(*p*-tolyl)imidazo[1,2-*a*]pyridine-7-carboxamide (OSA_001015)

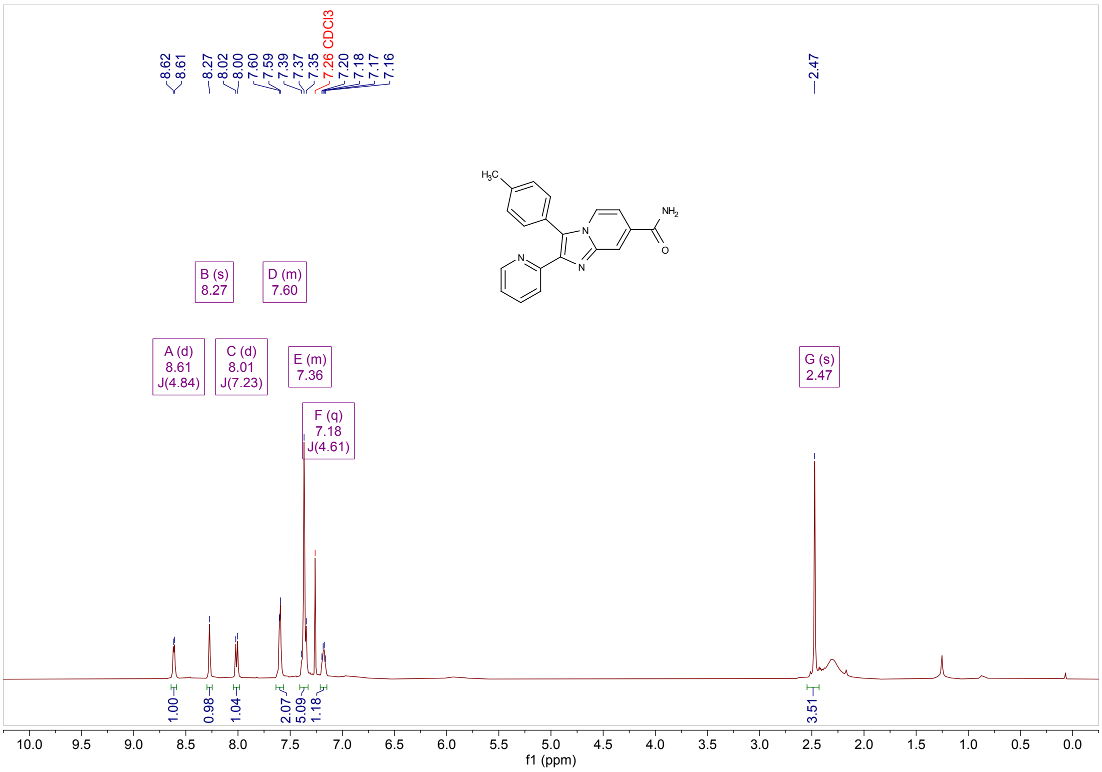

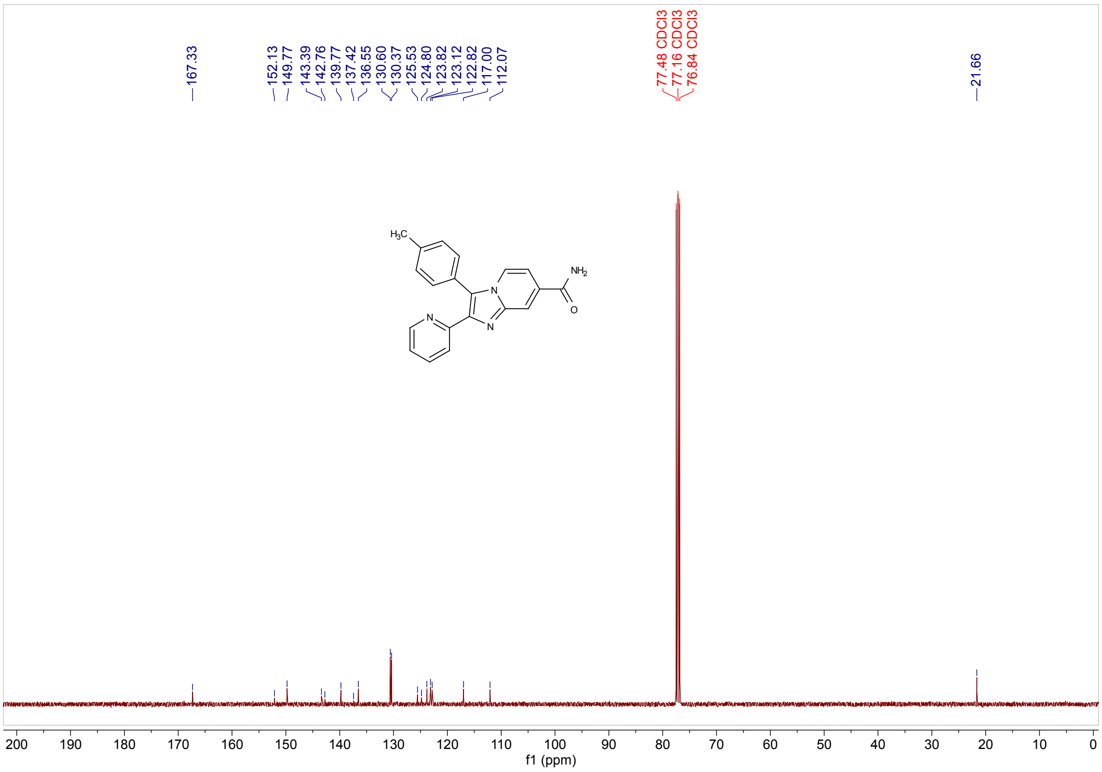

3-(Benzofuran-5-yl)-2-(pyridin-2-yl)imidazo[1,2-*a*]pyrimidine (OSA_000863)

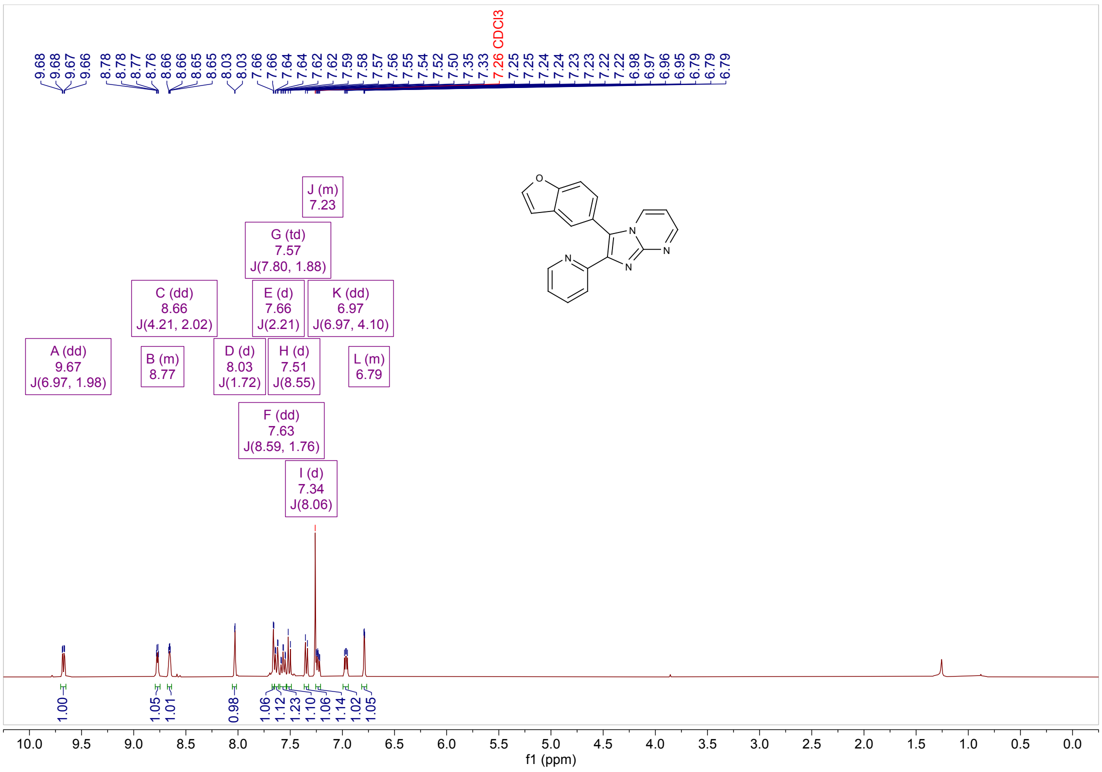

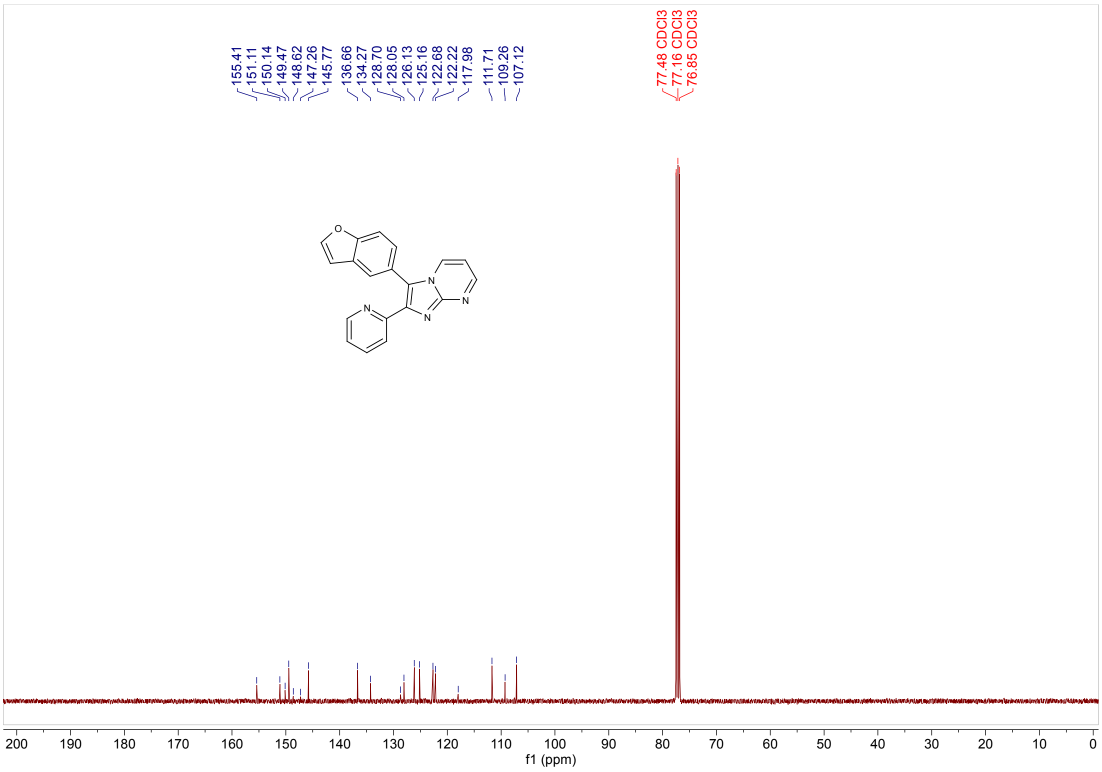

3-(Benzo[*d*][1,3]dioxol-5-yl)-2-(pyridin-2-yl)imidazo[1,2-*a*]pyrimidine (OSA_000815)

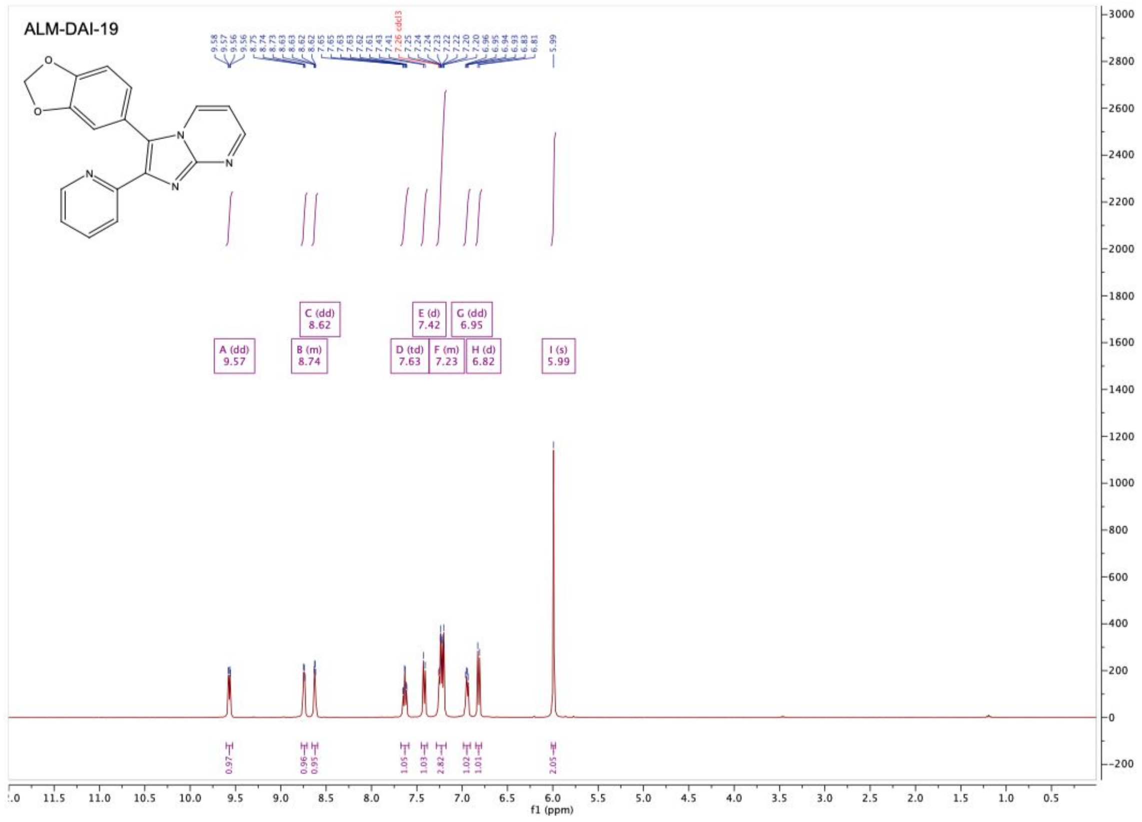

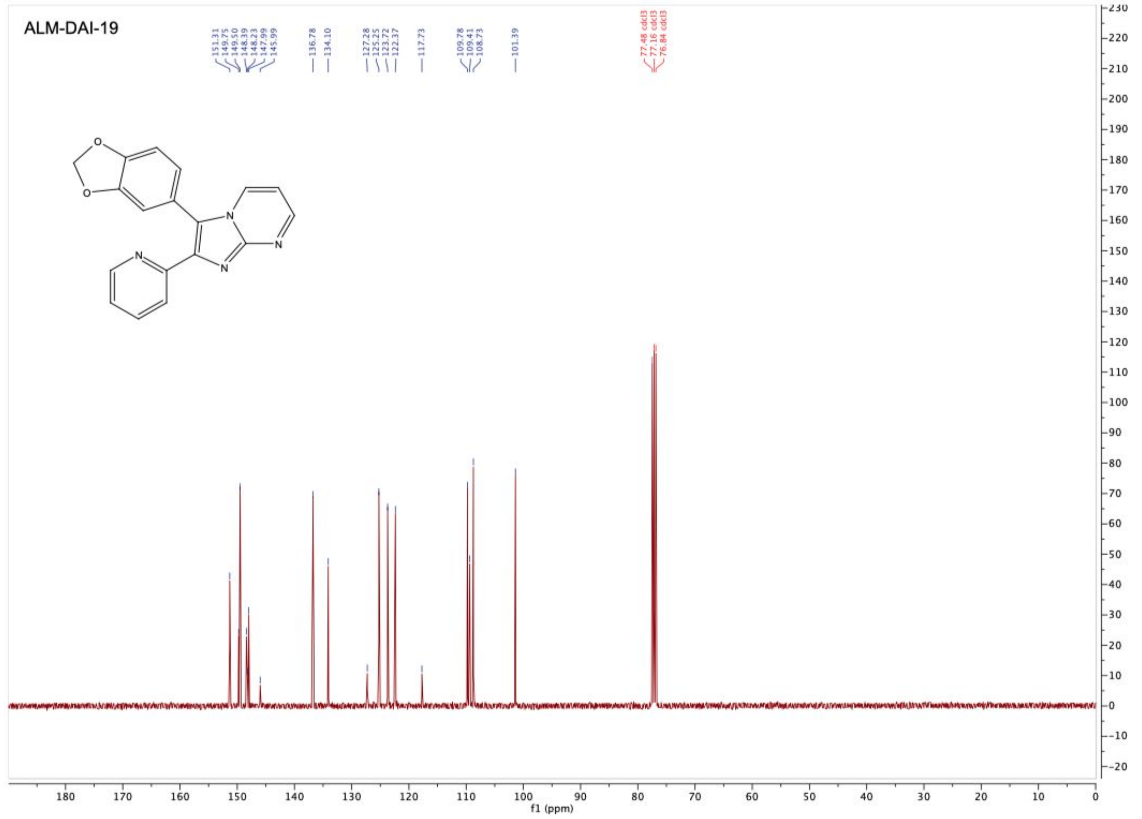

N-(4-Fluorophenyl)-*N*-isobutyl-2-(thiazol-2-yl)imidazo[1,2-*a*]pyridin-3-amine (OSA_001010)

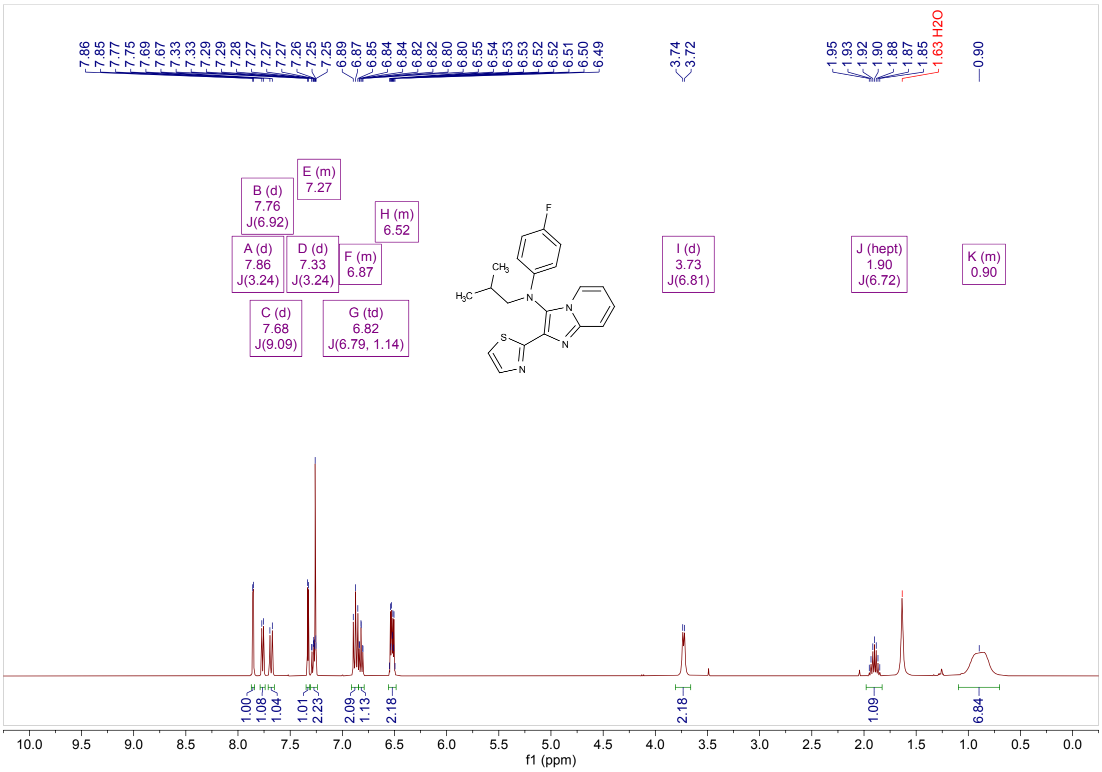

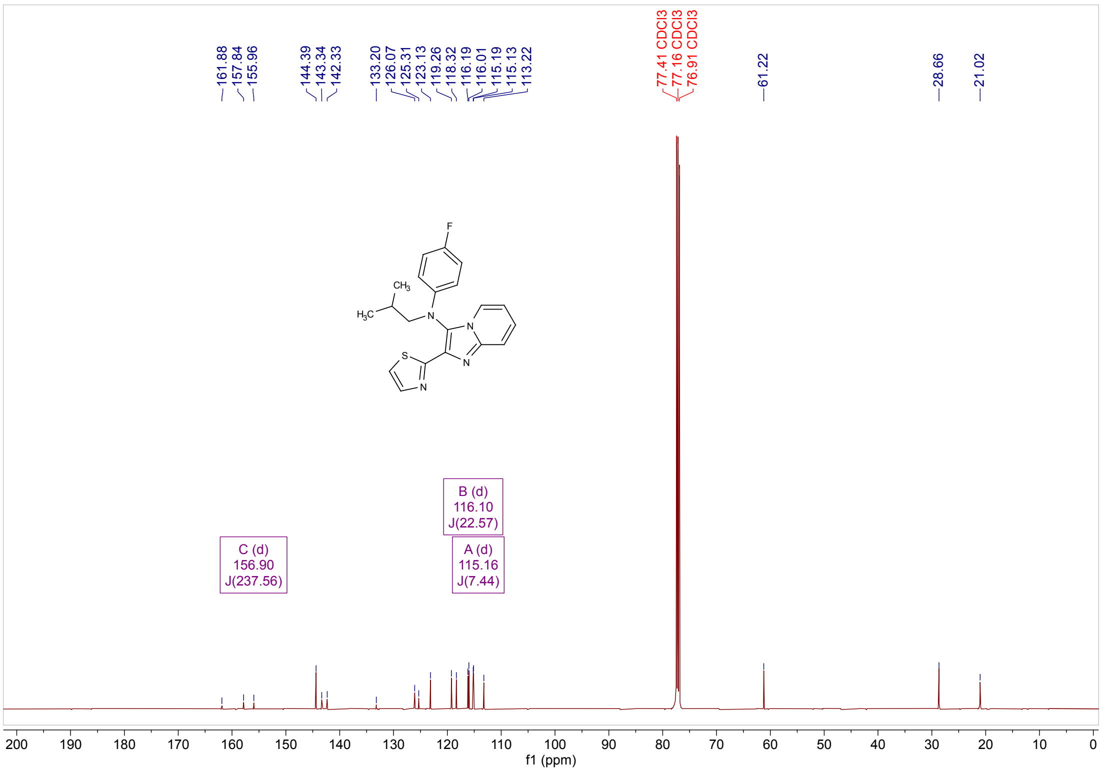

6-Methyl-2-phenylimidazo[1,2-*a*]pyridine (OSA_001026)

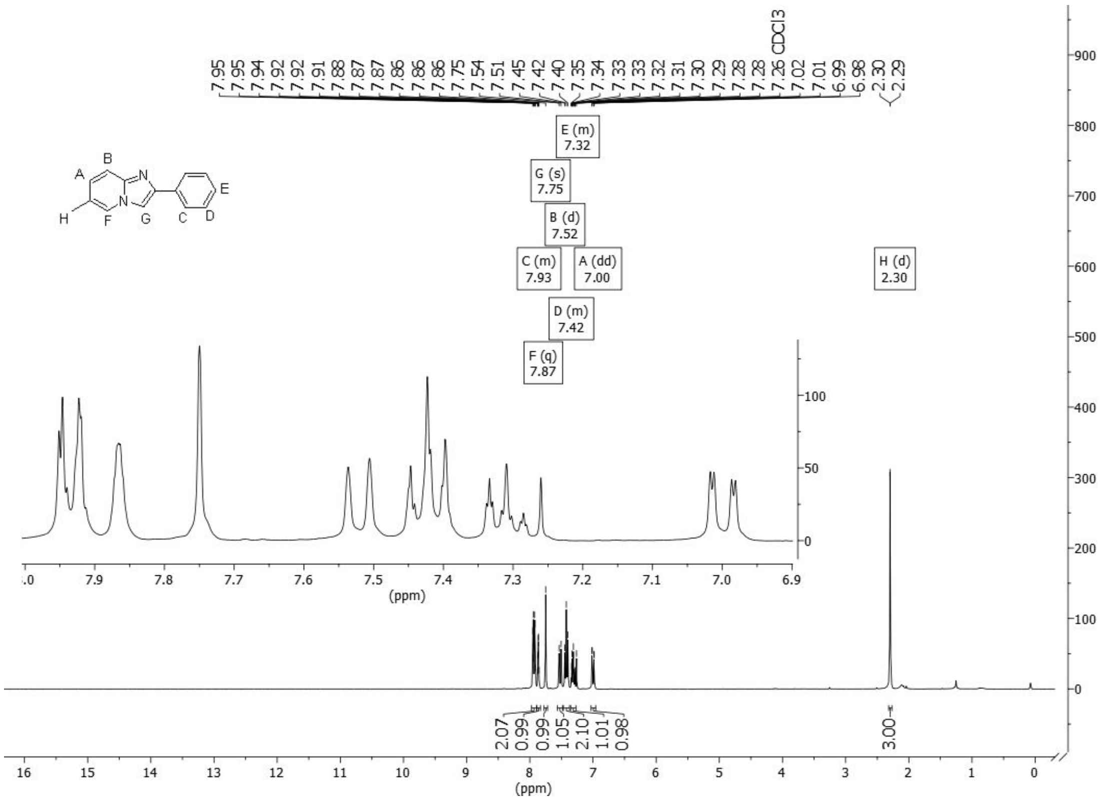

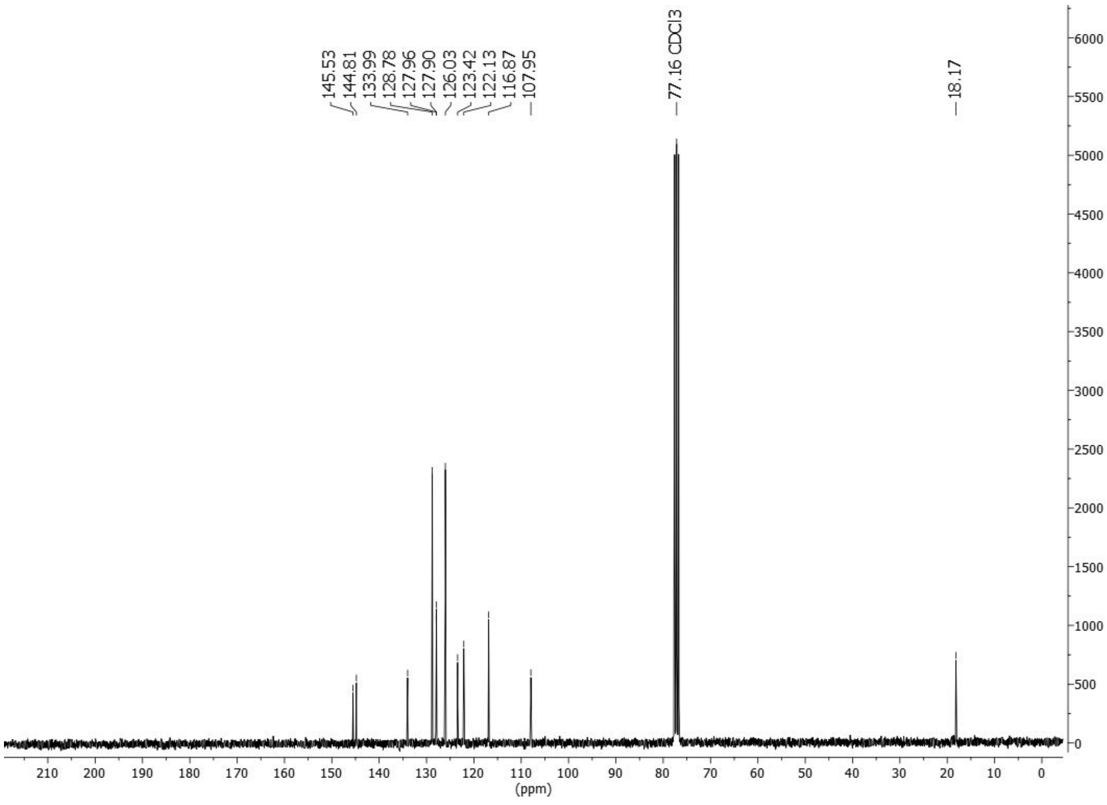

***N*-((6-Methyl-2-phenylimidazo[1,2-*a*]pyridin-3-yl)methyl)-3-(trifluoromethyl)aniline**

(OSA_001028)

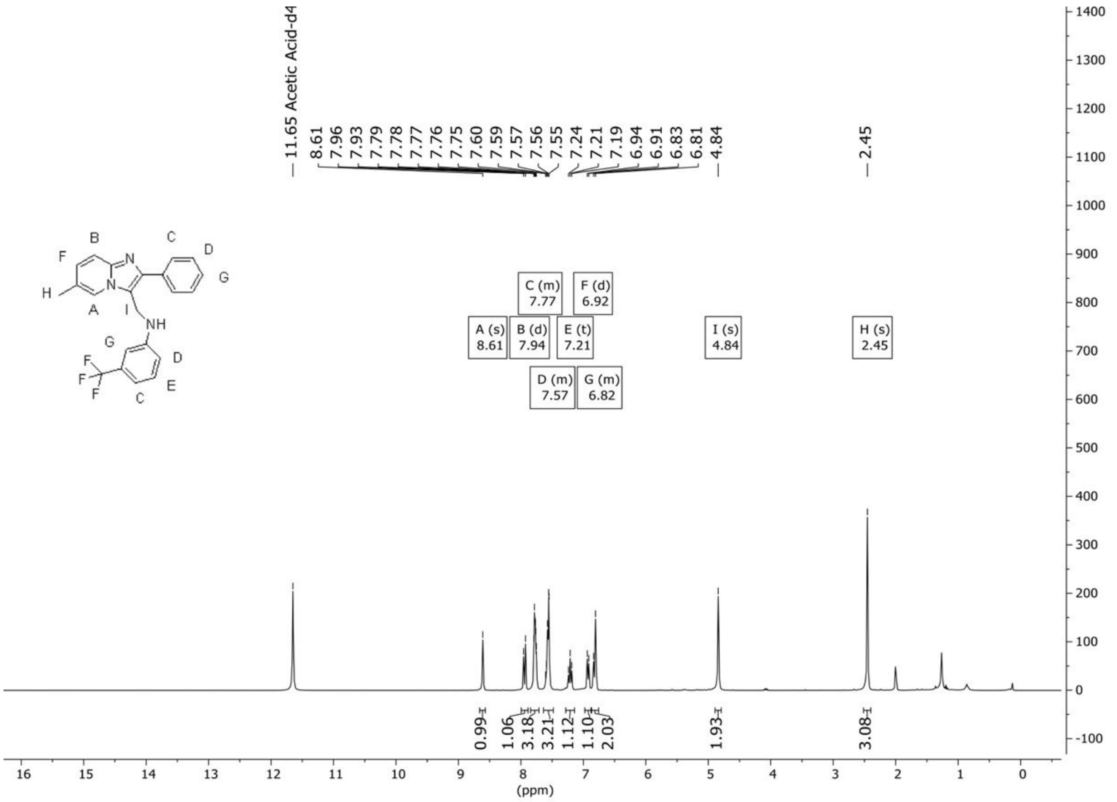

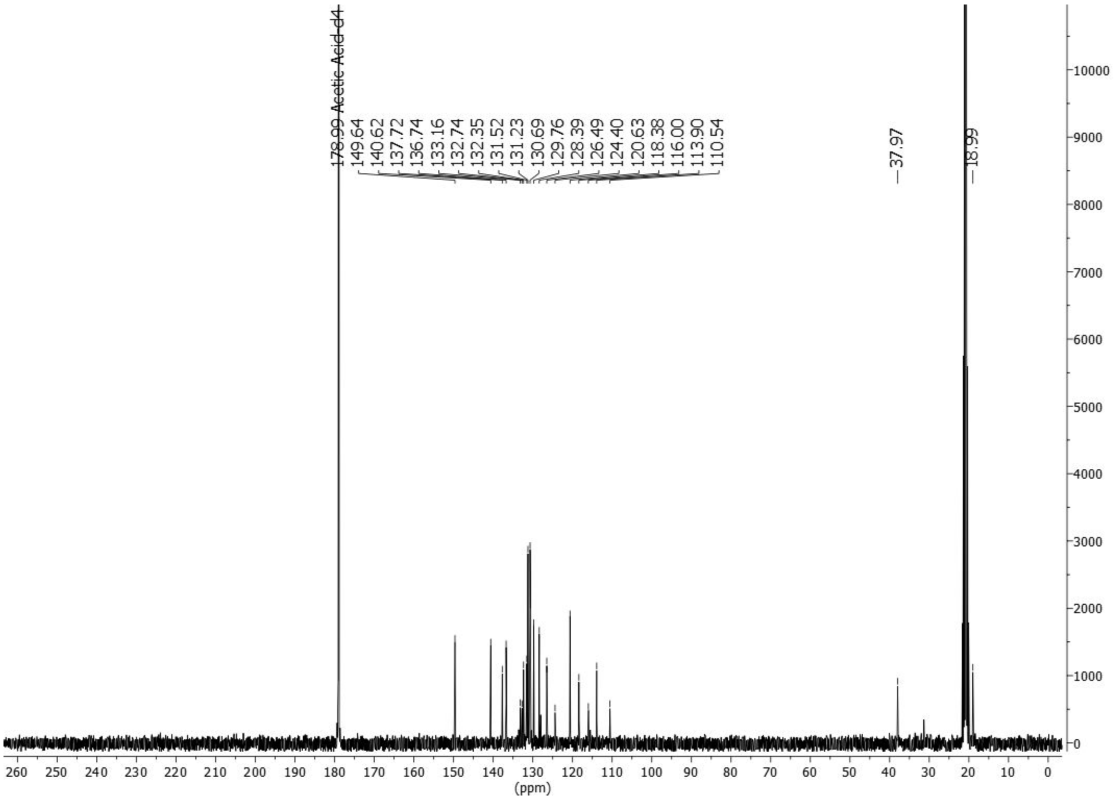

4-Fluoro-*N*-((6-methyl-2-phenylimidazo[1,2-*a*]pyridin-3-yl)methyl)aniline

(OSA_001027)

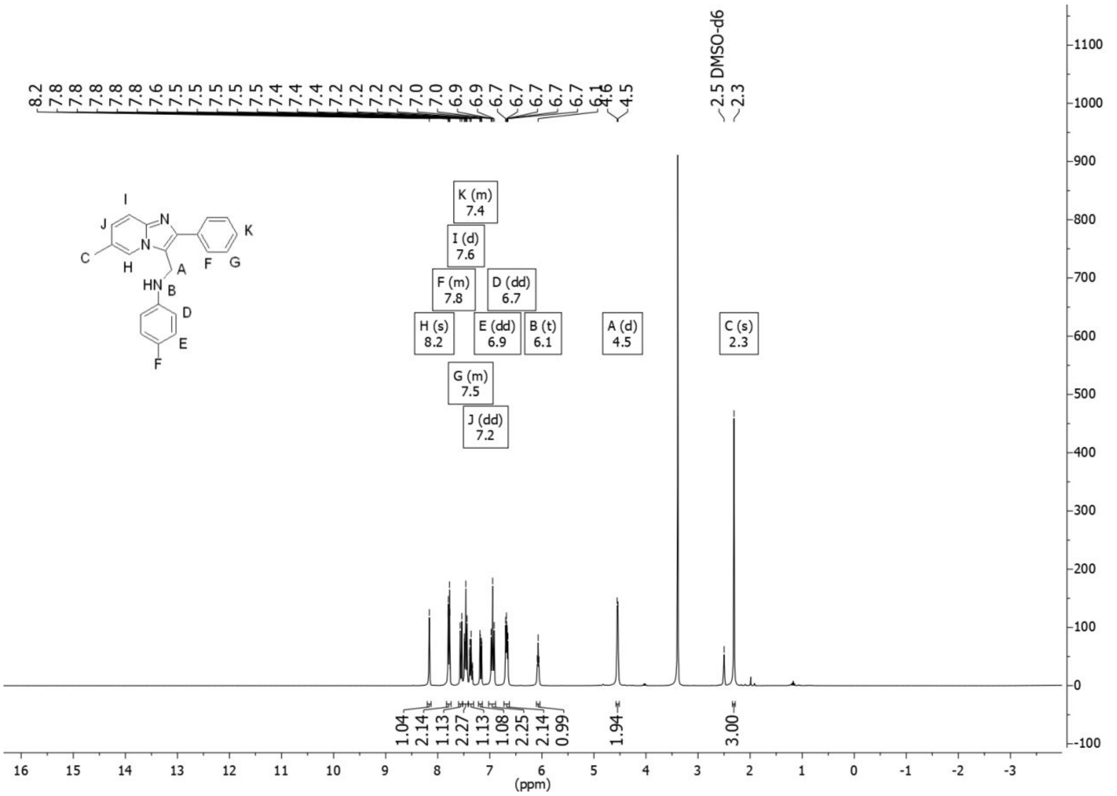

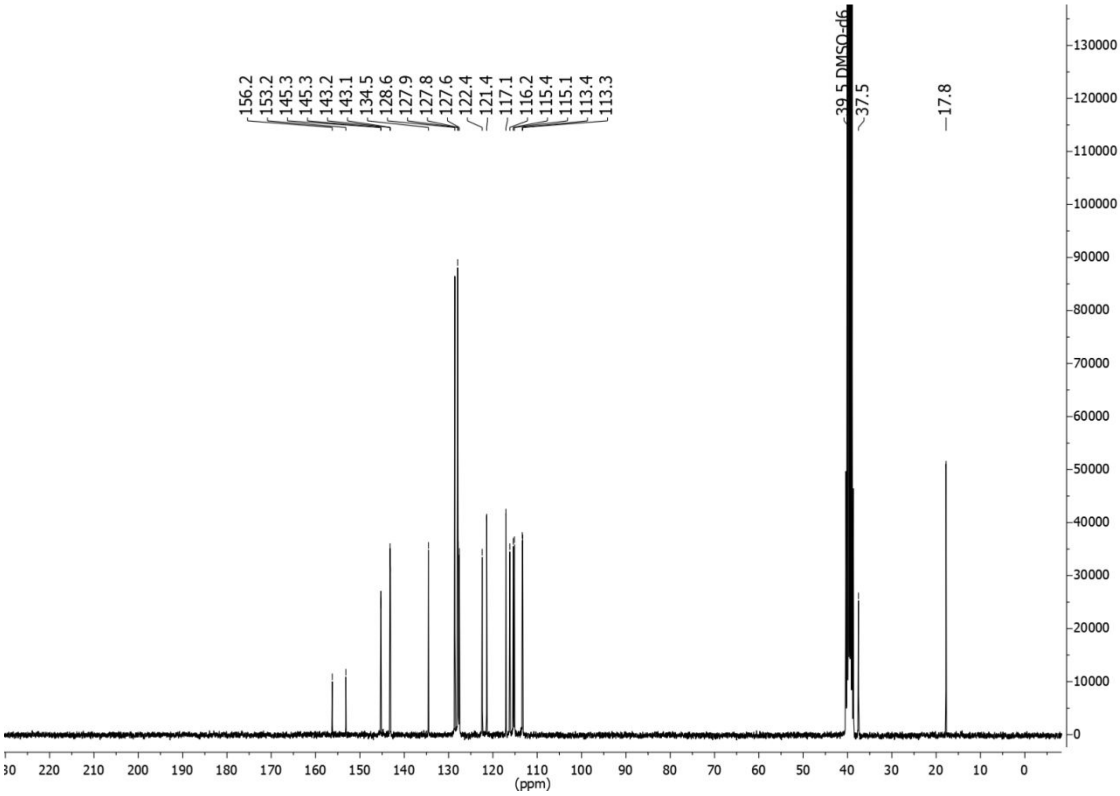

***N*-Butyl-*N*-((6-methyl-2-phenylimidazo[1,2-*a*]pyridin-3-yl)methyl)-3-(trifluoromethyl)aniline (OSA_001029)**

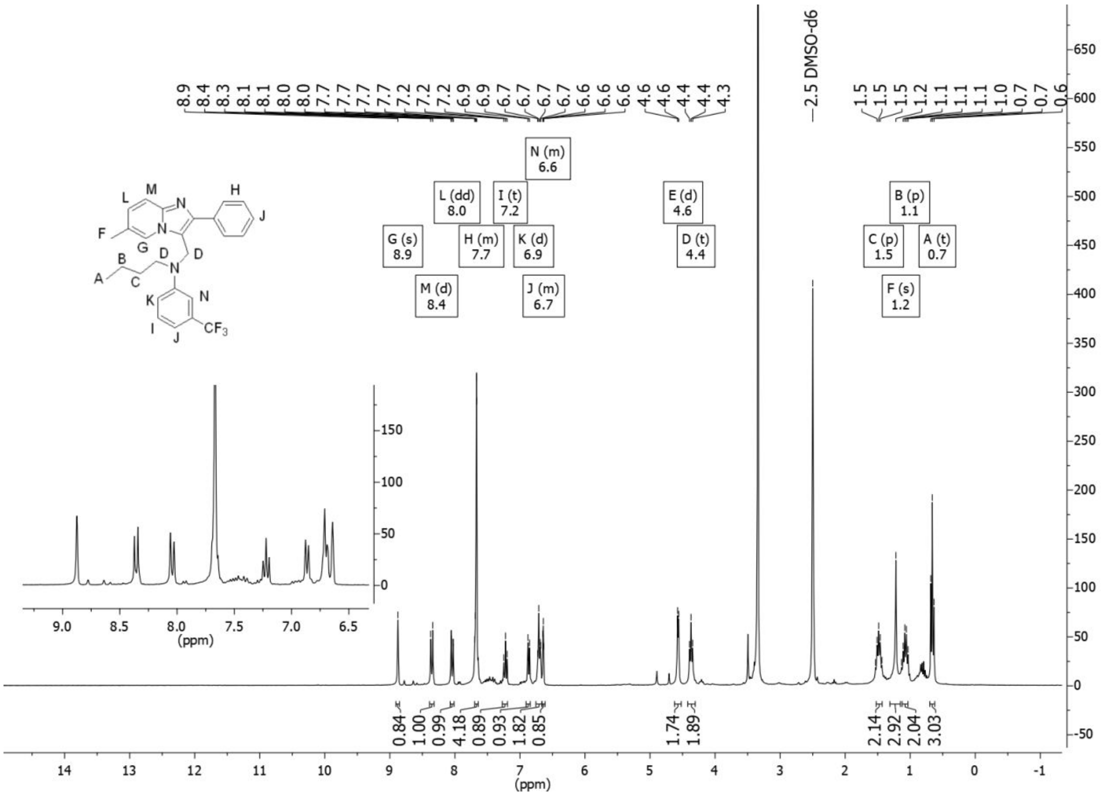

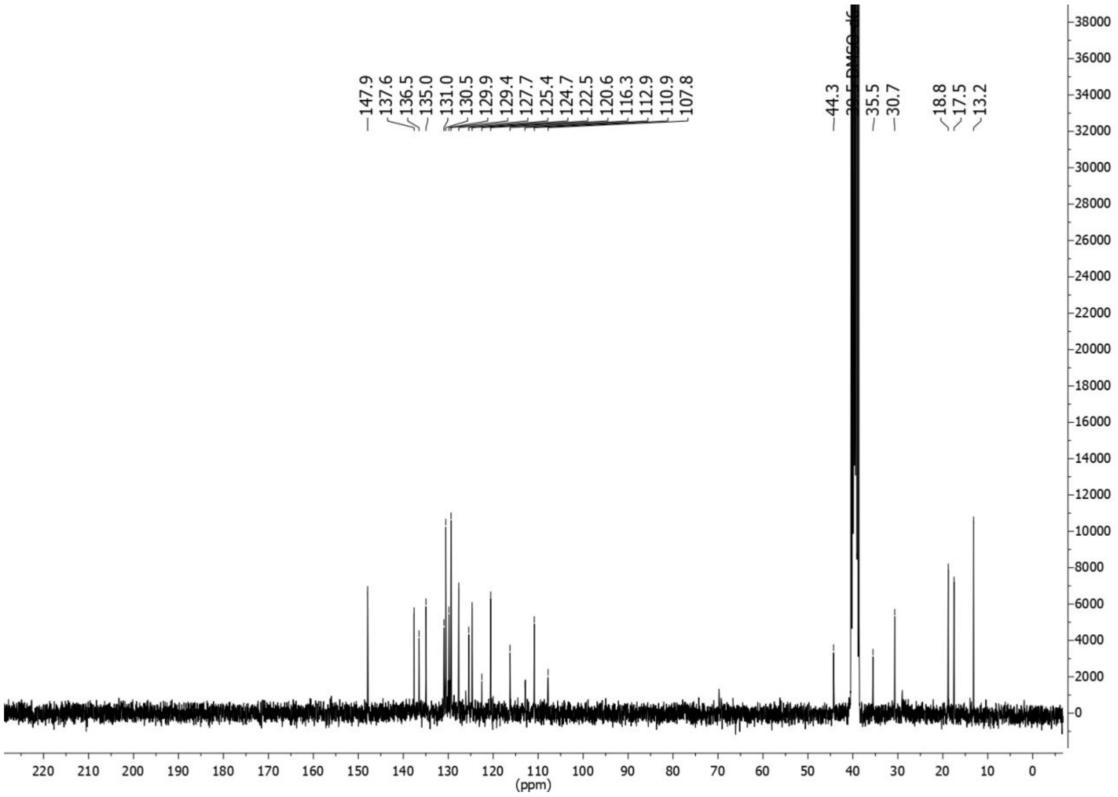

7-Methyl-2-(pyridin-2-yl)imidazo[1,2-*a*]pyridine (OSA_001030)

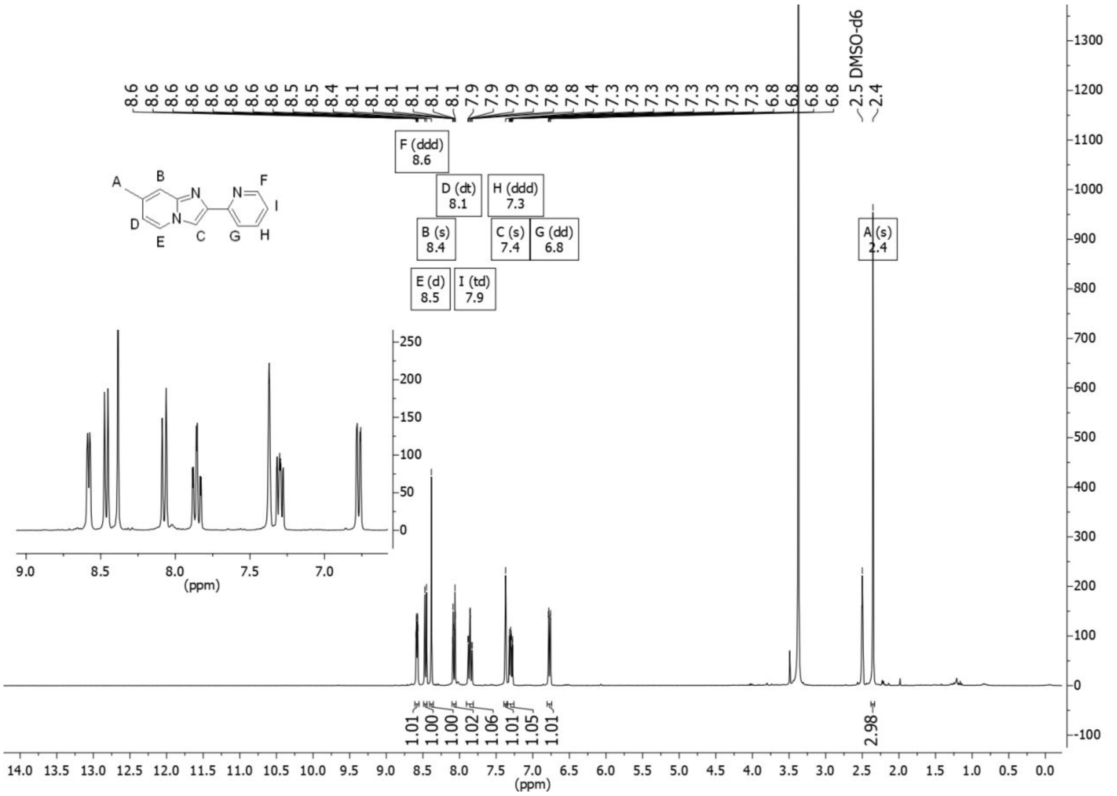

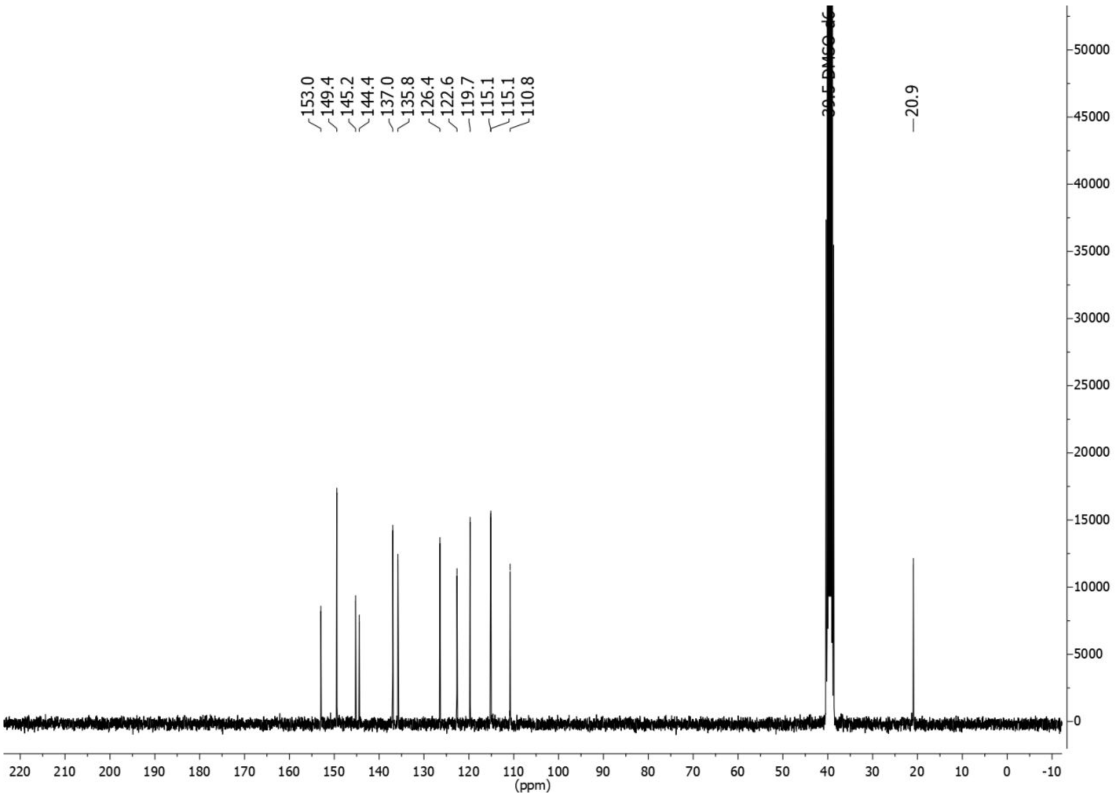

4-Fluoro-*N*-((7-methyl-2-(pyridin-2-yl)imidazo[1,2-*a*]pyridin-3-yl)methyl)aniline

(OSA_001031)

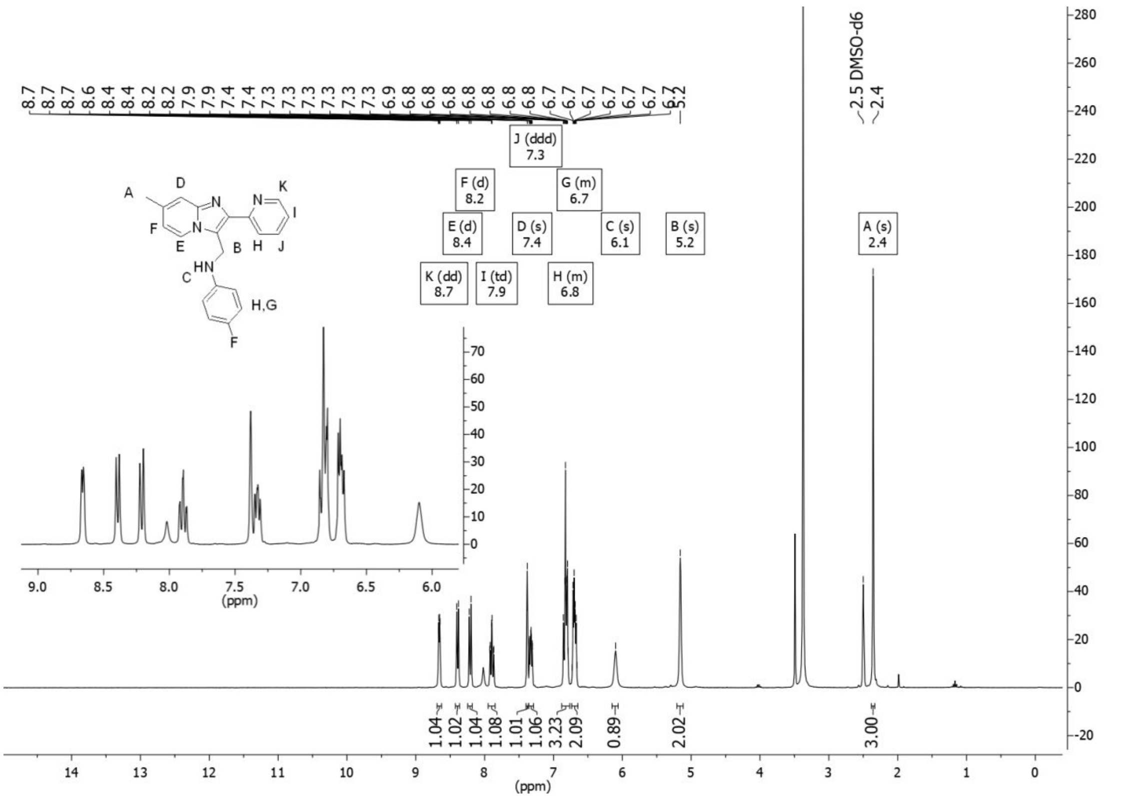

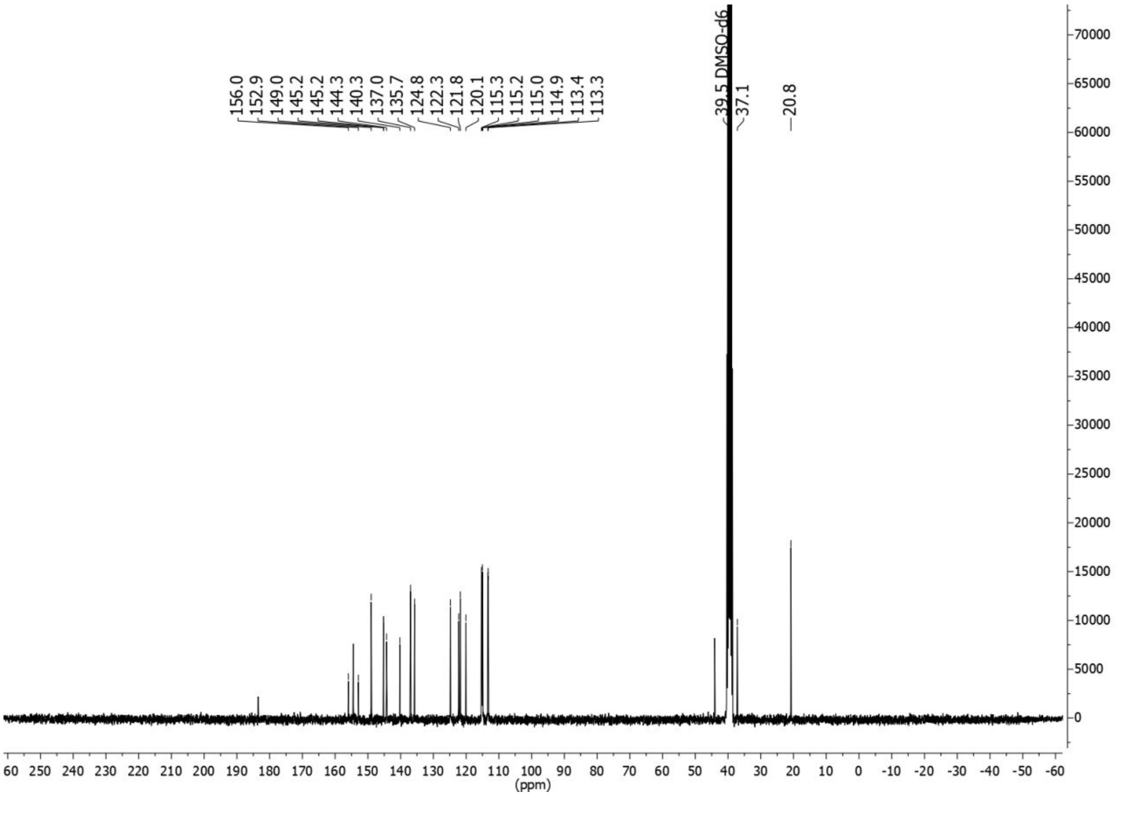

_N_-Butyl-4-fluoro-_N_-((7-methyl-2-(pyridin-2-yl)imidazo[1,2-_a_]pyridin-3-yl)methyl)aniline (OSA_001032)

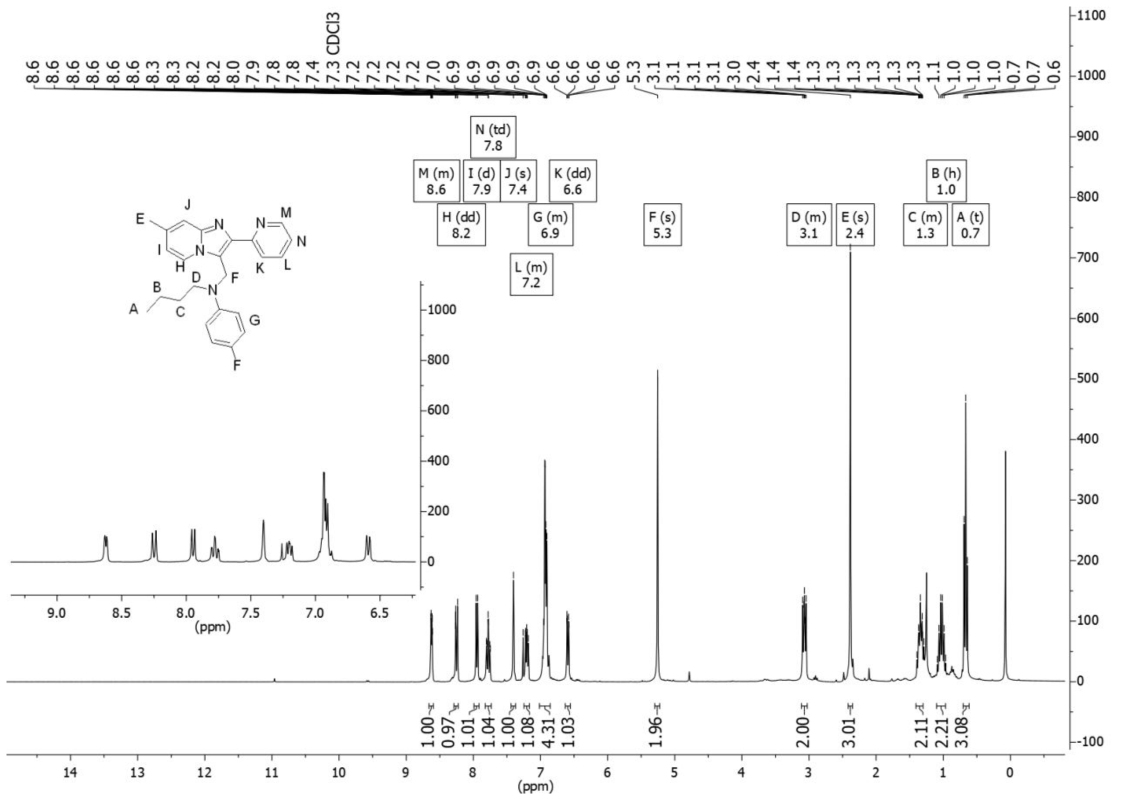

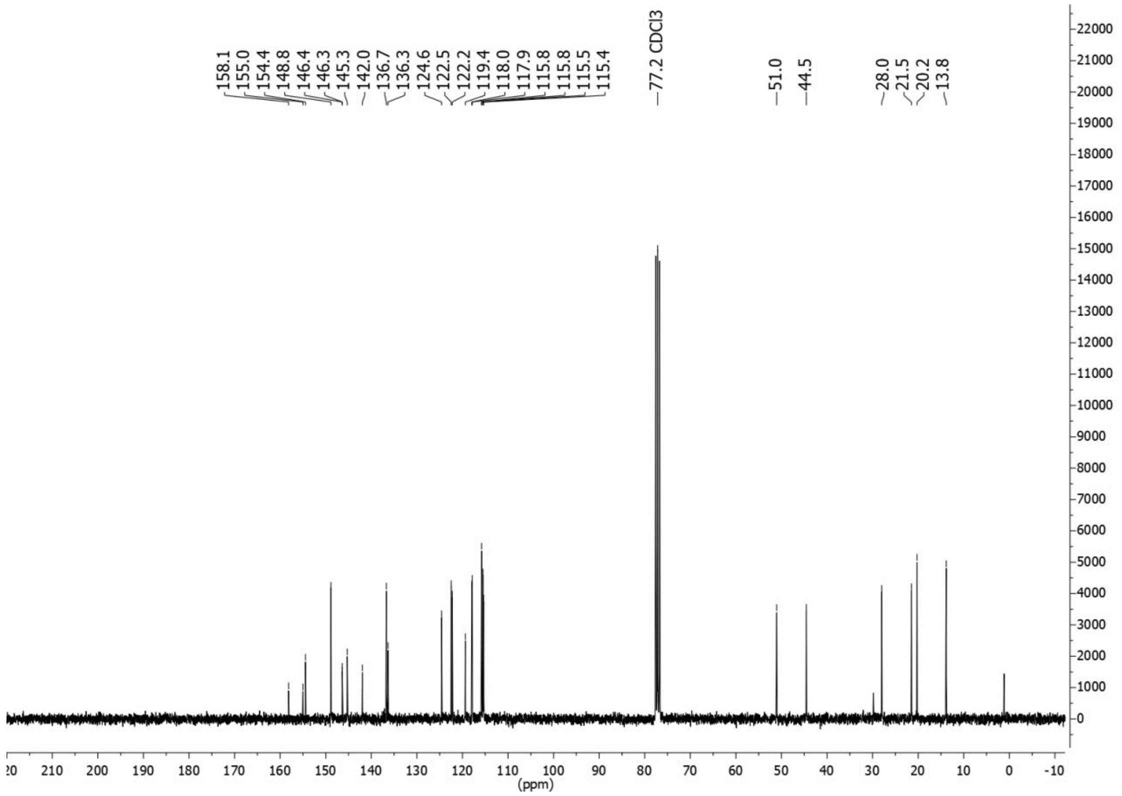

_N_-Isobutyl-_N_-((7-methyl-2-(pyridin-2-yl)imidazo[1,2-_a_]pyridin-3-yl)methyl)-3-(trifluoromethyl)aniline (OSA_001033)

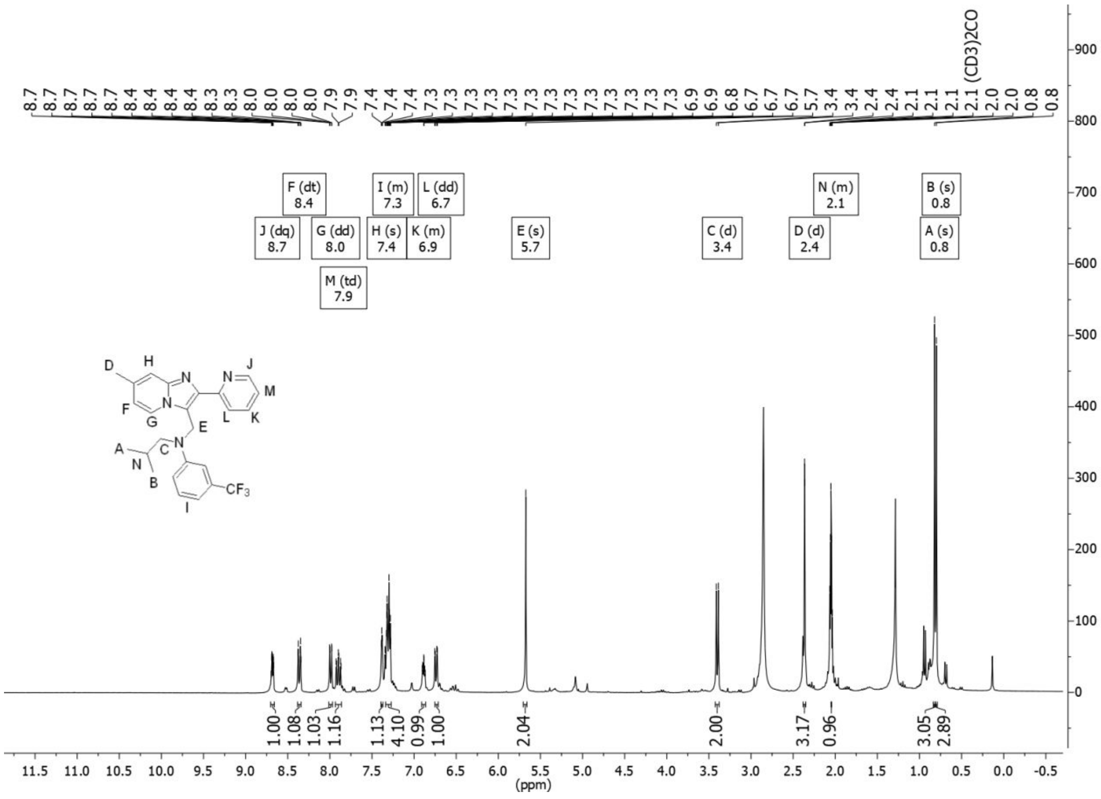

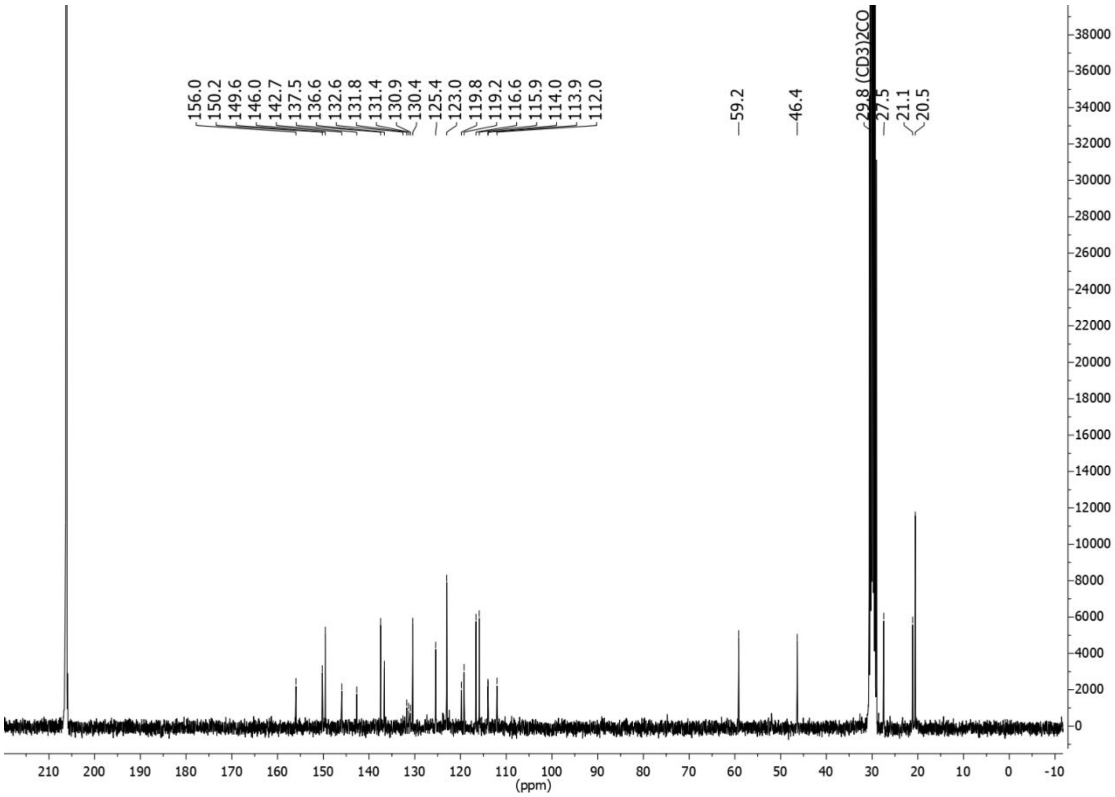

1-(Benzofuran-5-yl)-2-(pyridin-2-yl)-1*H*-benzo[*d*]imidazole (OSA_000991)

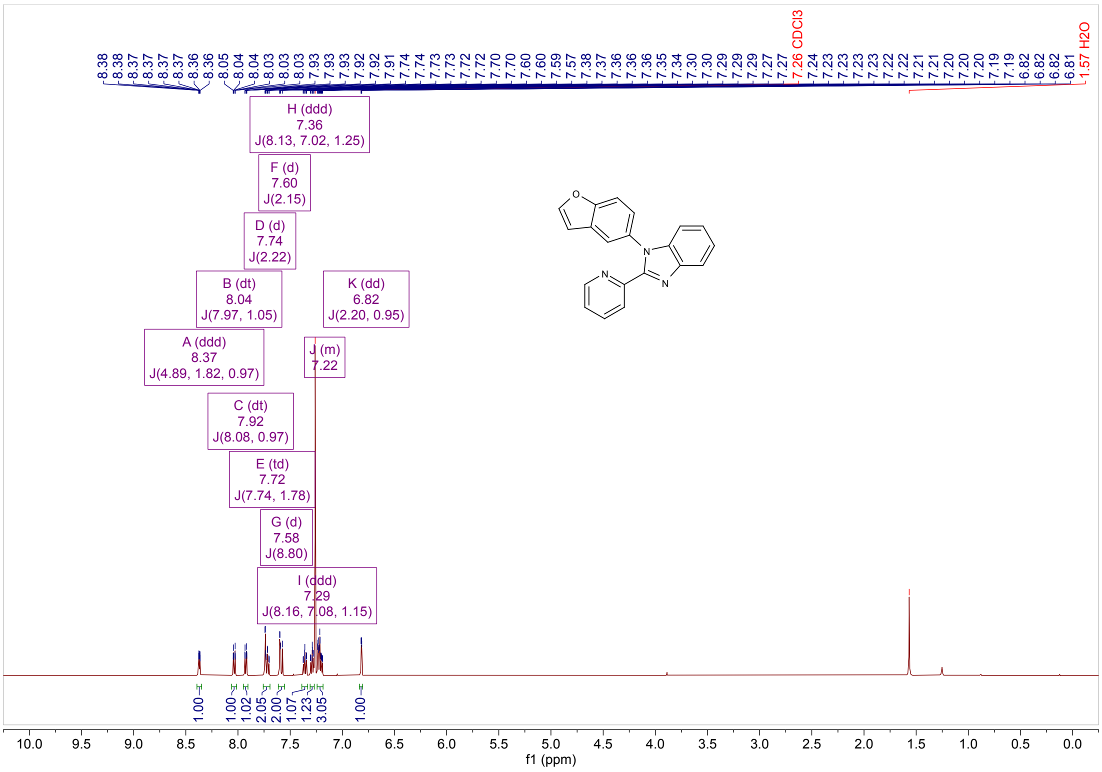

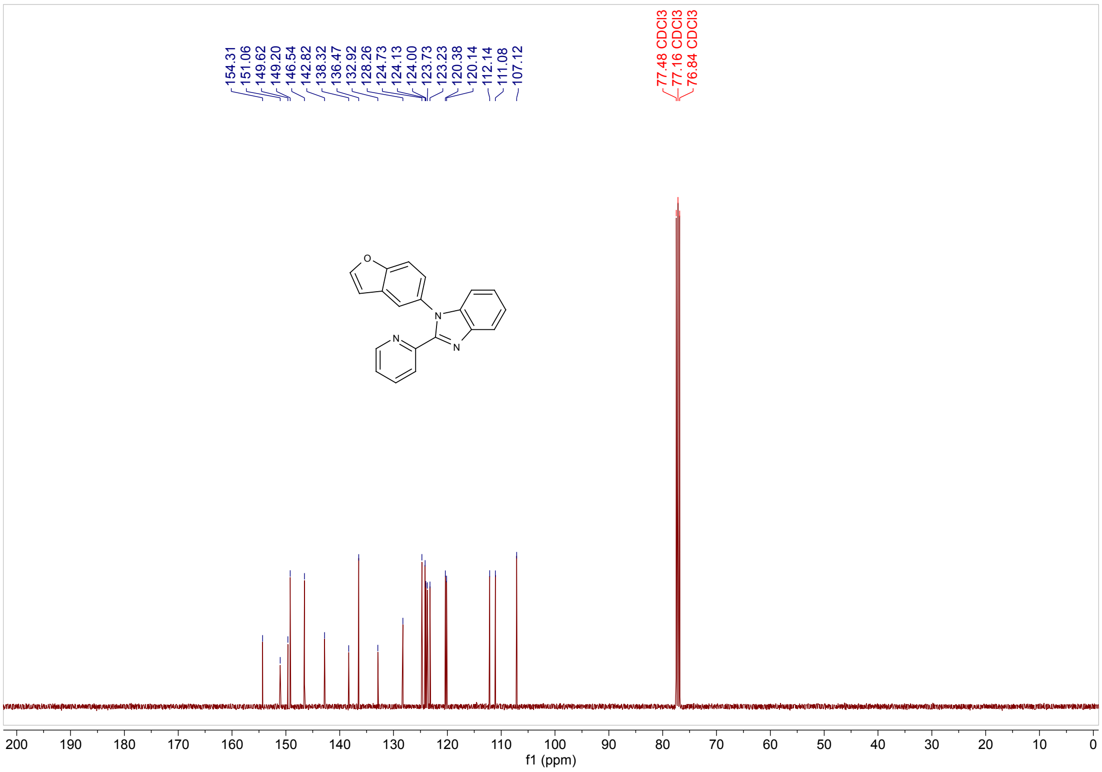

1-Phenyl-2-(pyridin-2-yl)-1*H*-benzo[*d*]imidazole (OSA_000992)

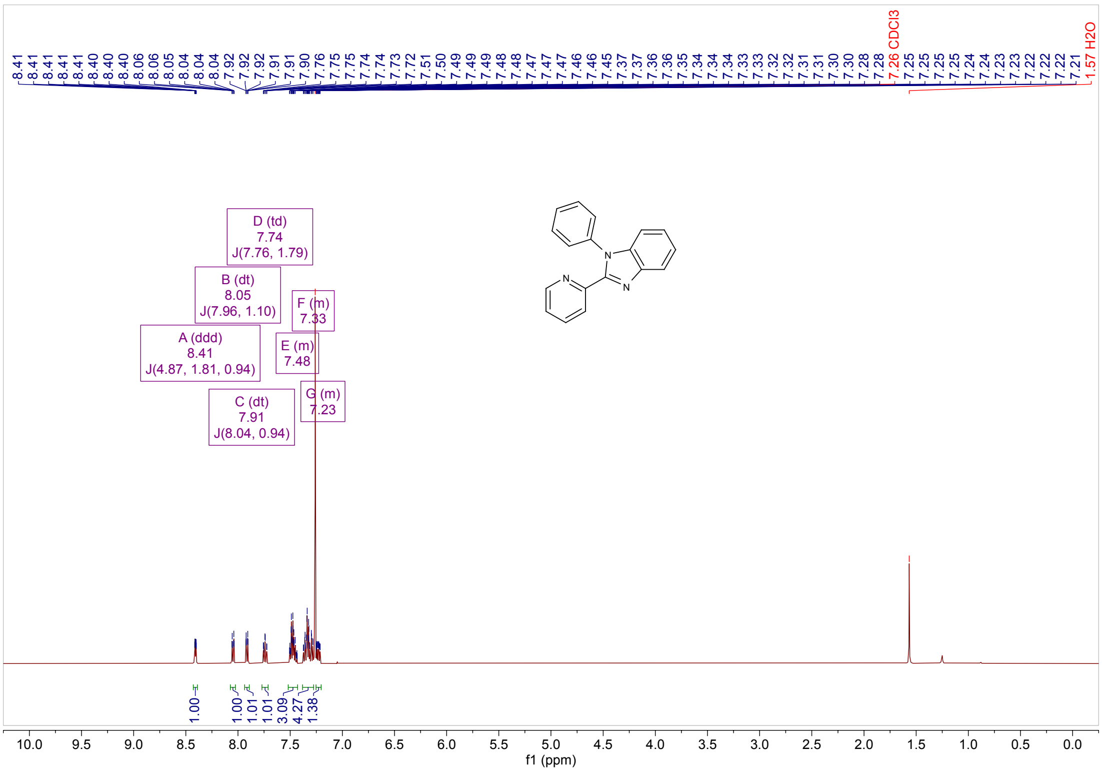

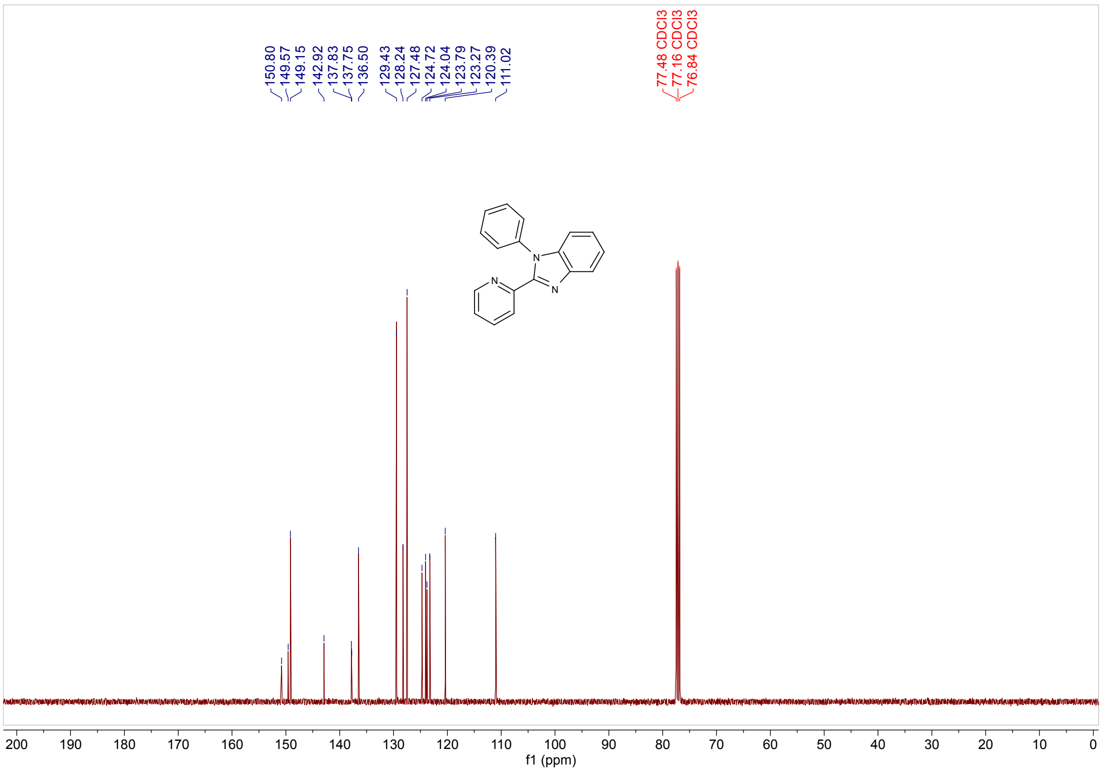

1-Benzyl-2-(pyridin-2-yl)-1*H*-benzo[*d*]imidazole (OSA_000993)

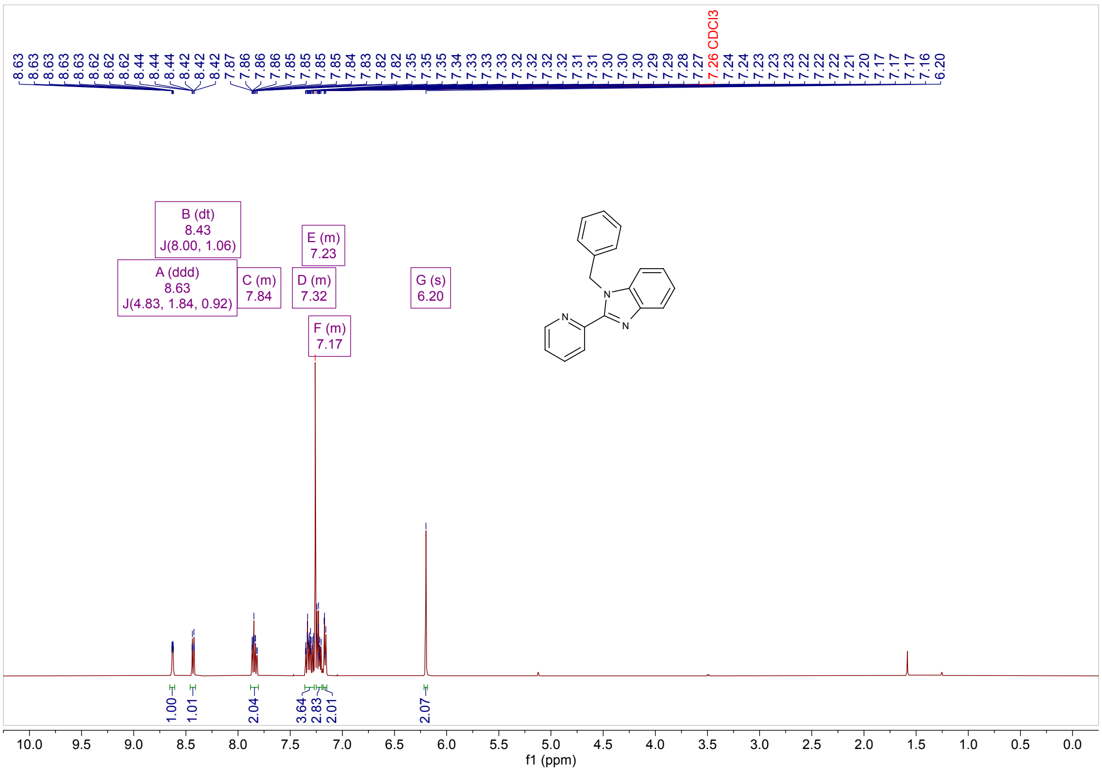

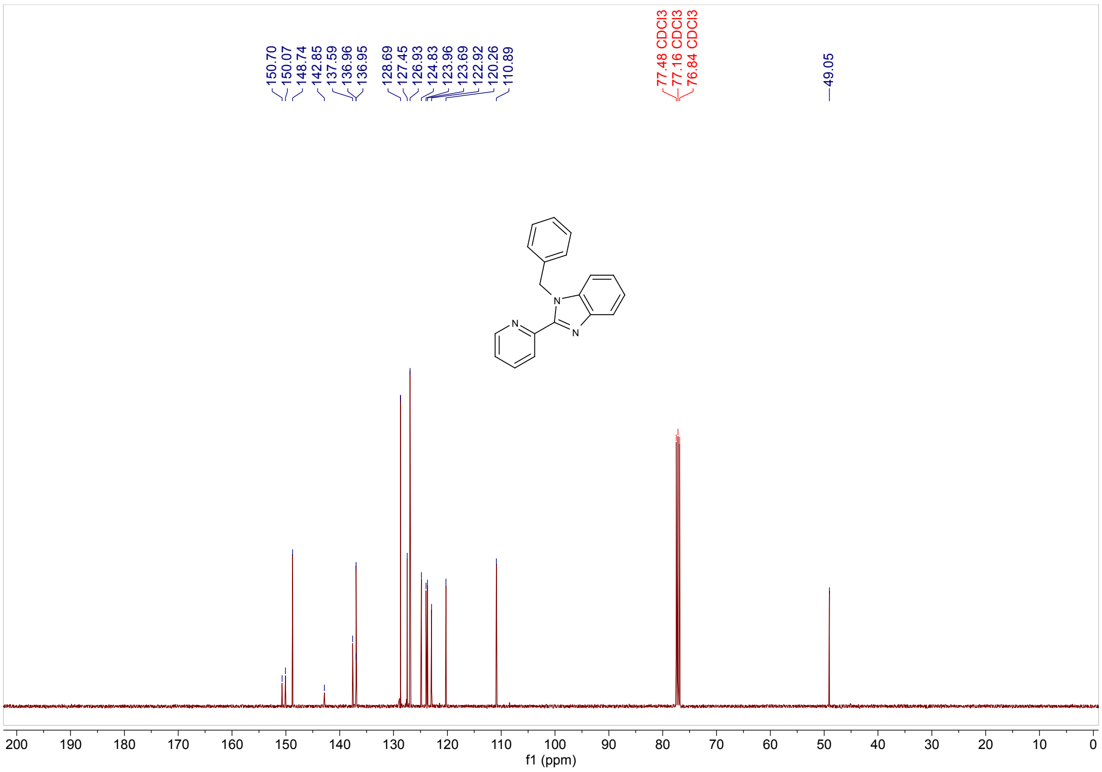

1-(4-(Piperidin-1-yl)phenyl)-2-(pyridin-2-yl)-1*H*-benzo[d]imidazole (OSA_000994)

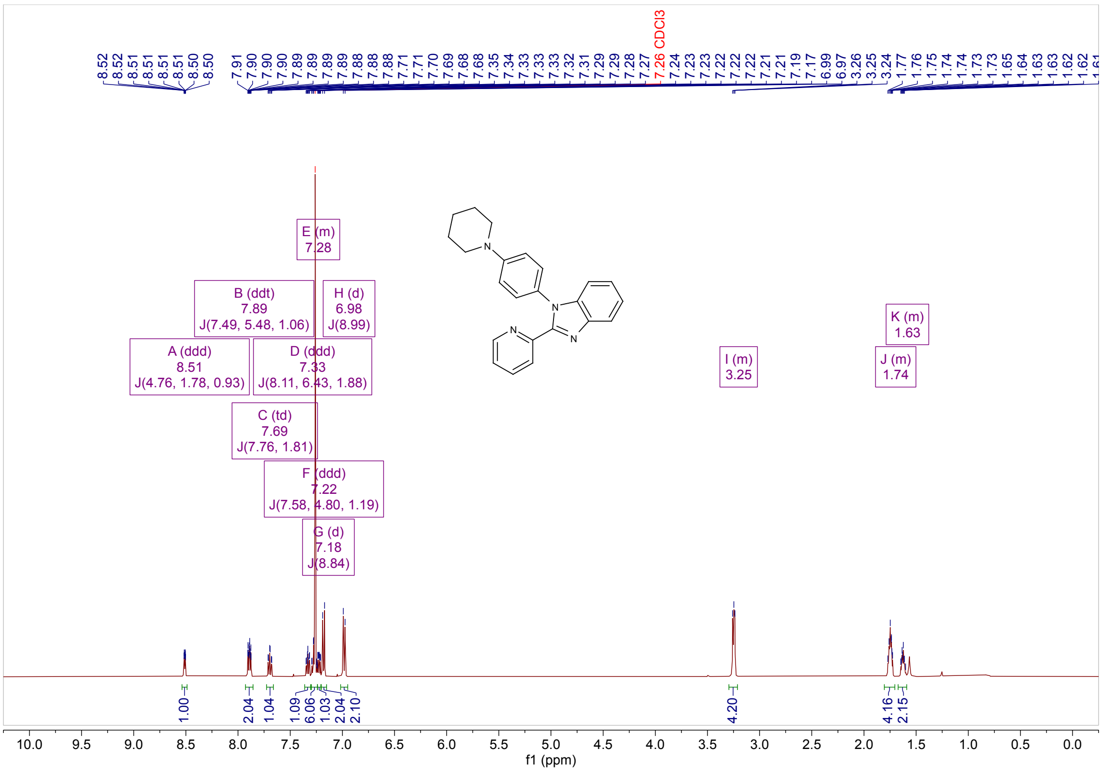

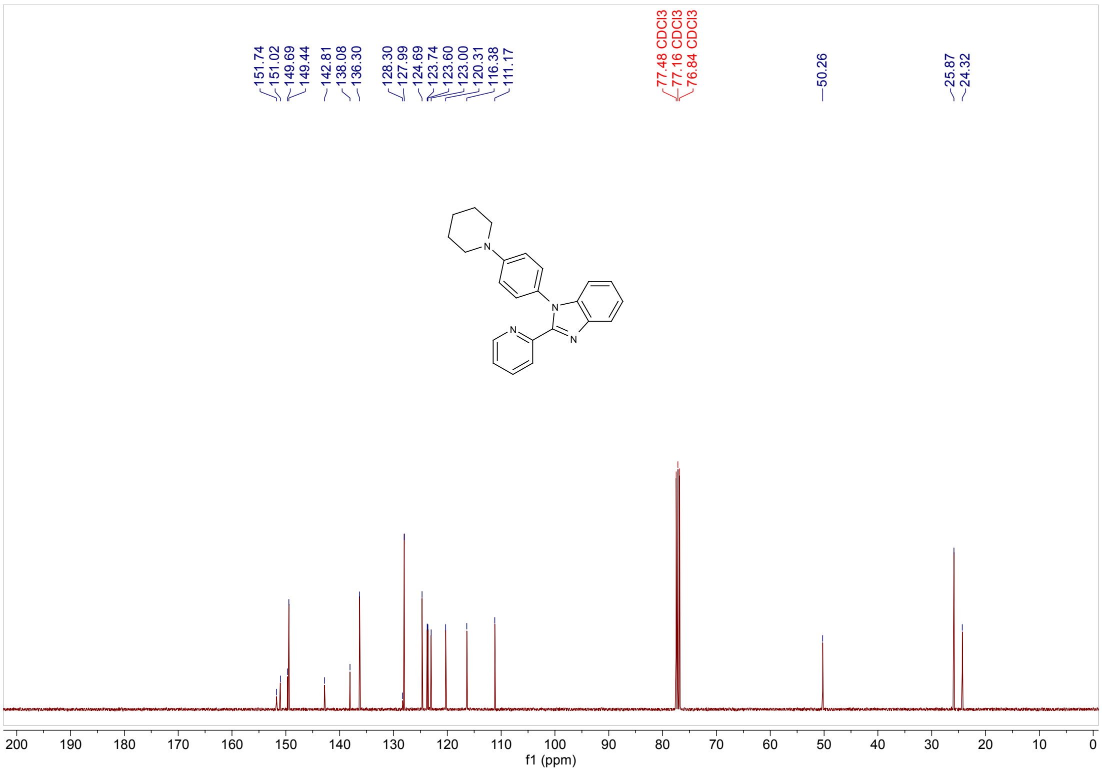

4-(4-(2-(Pyridin-2-yl)-1*H*-benzo[*d*]imidazol-1-yl)phenyl)morpholine (OSA_000995)

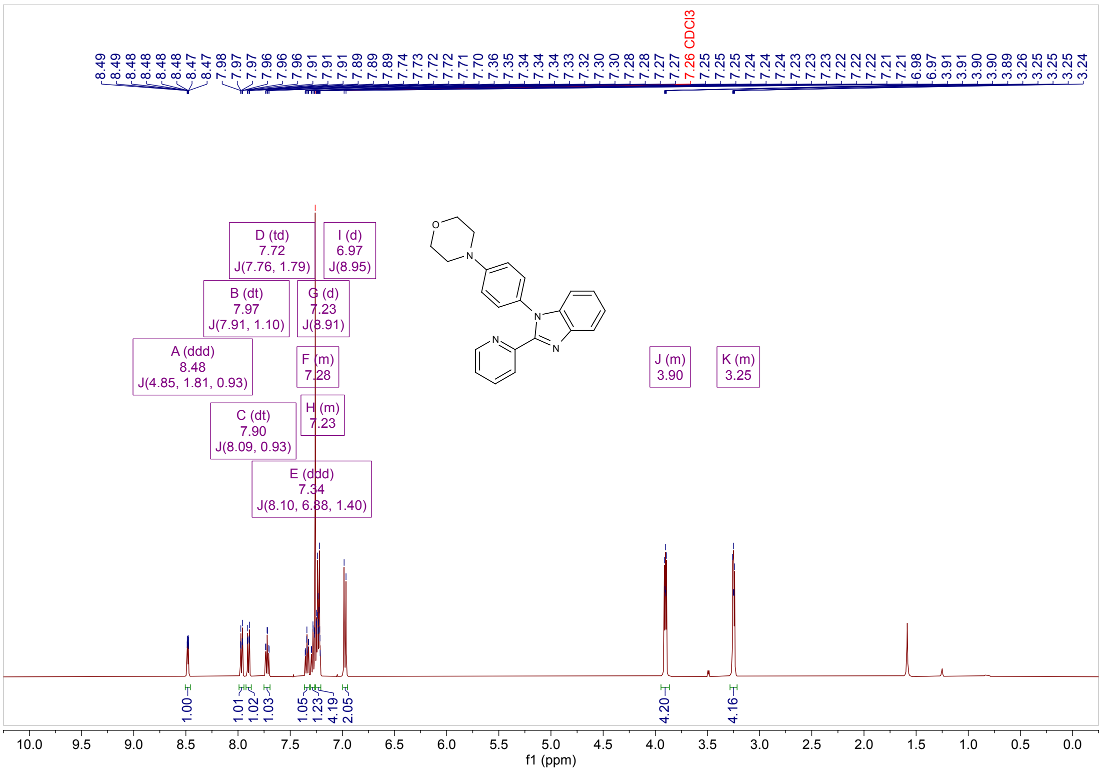

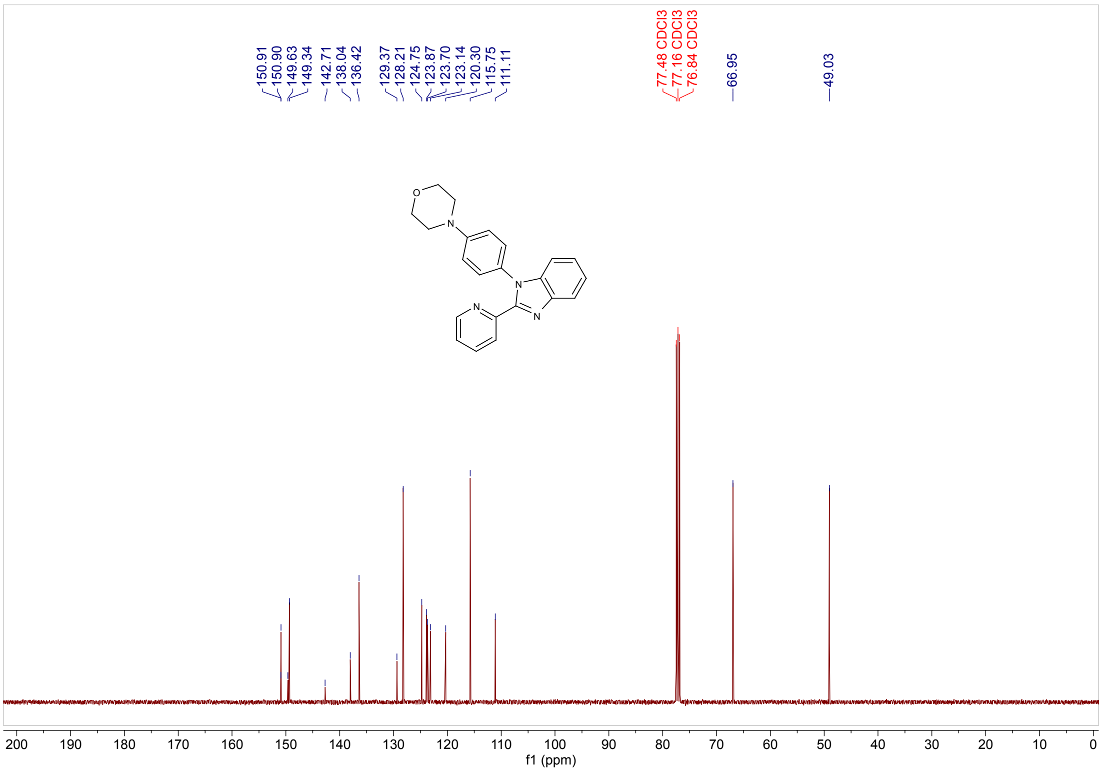

2-(4-(2-(Pyridin-2-yl)-1*H*-benzo[*d*]imidazol-1-yl)phenyl)acetonitrile (OSA_000996)

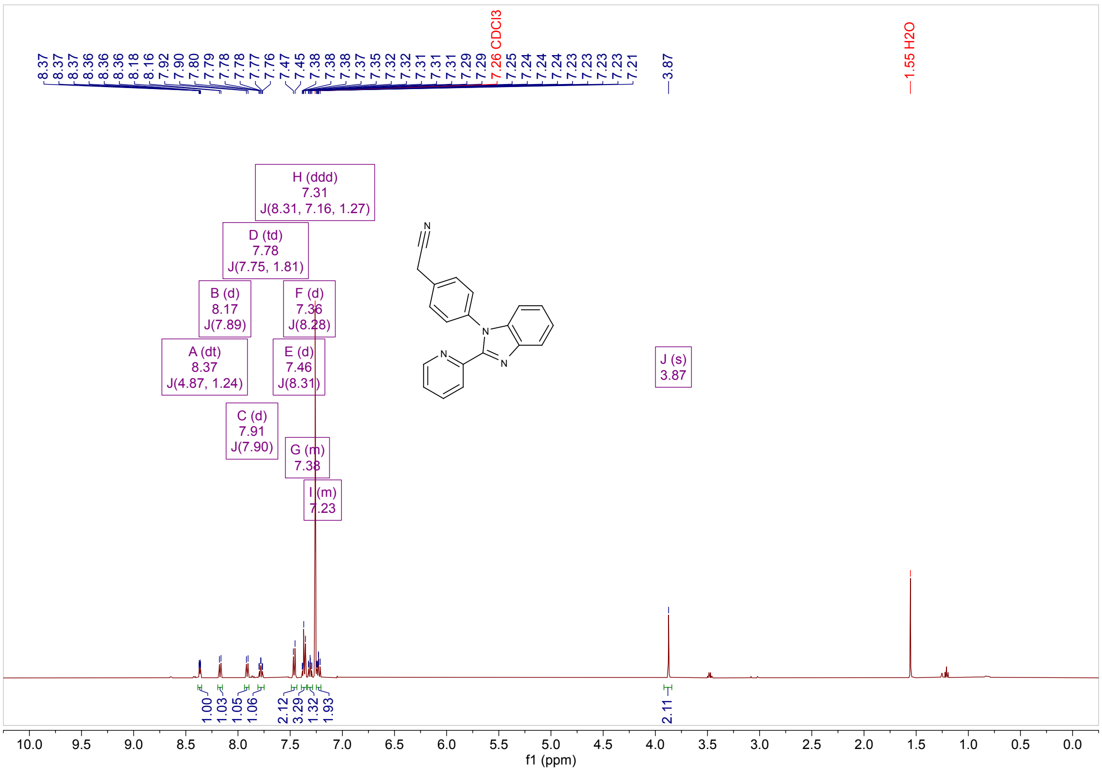

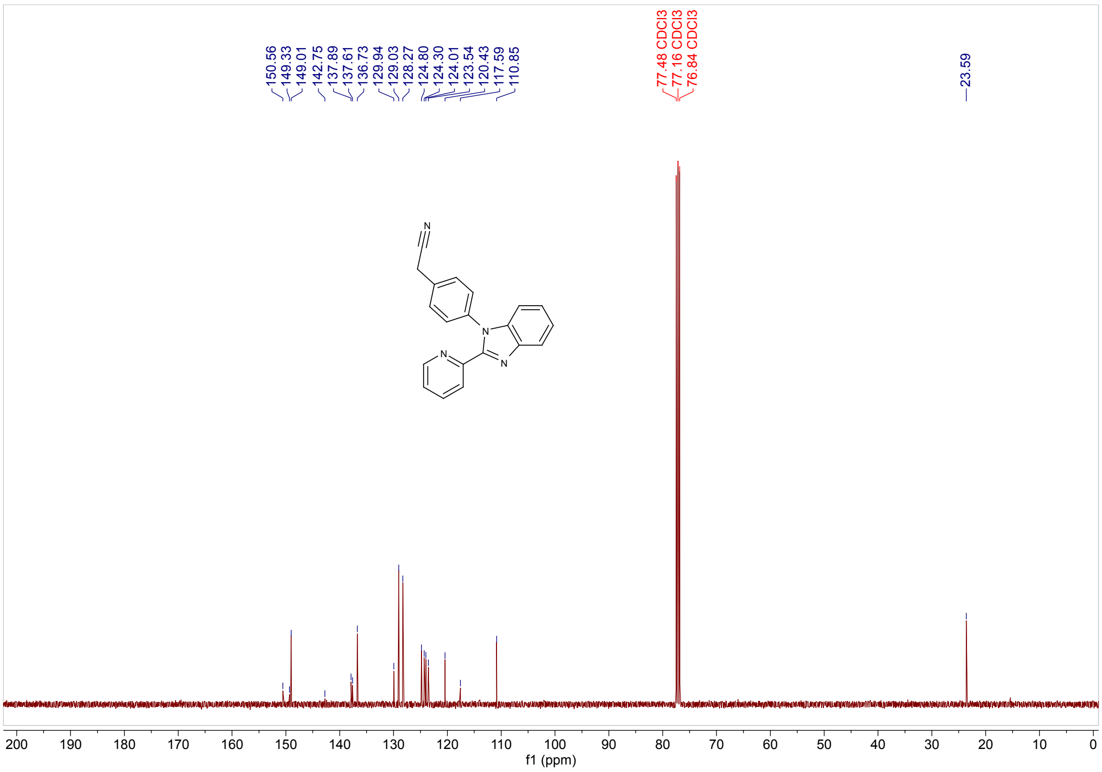

4-(2-(Pyridin-2-yl)-1*H*-benzo[*d*]imidazol-1-yl)benzonitrile (OSA_000990)

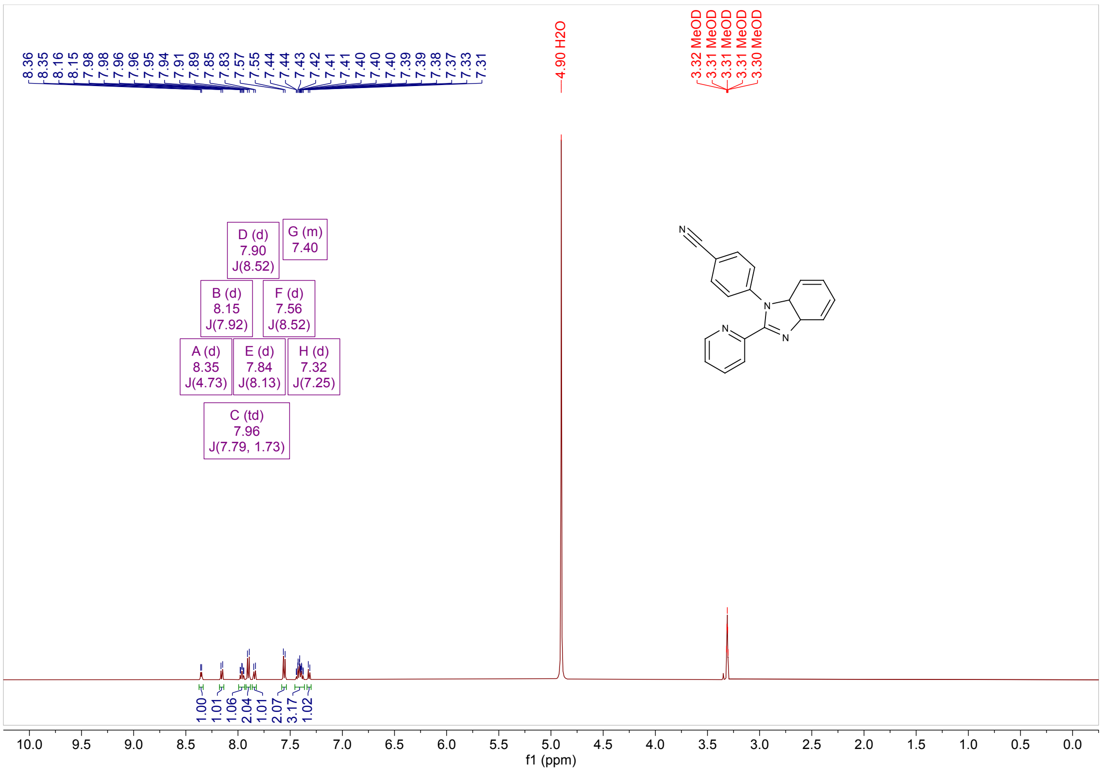

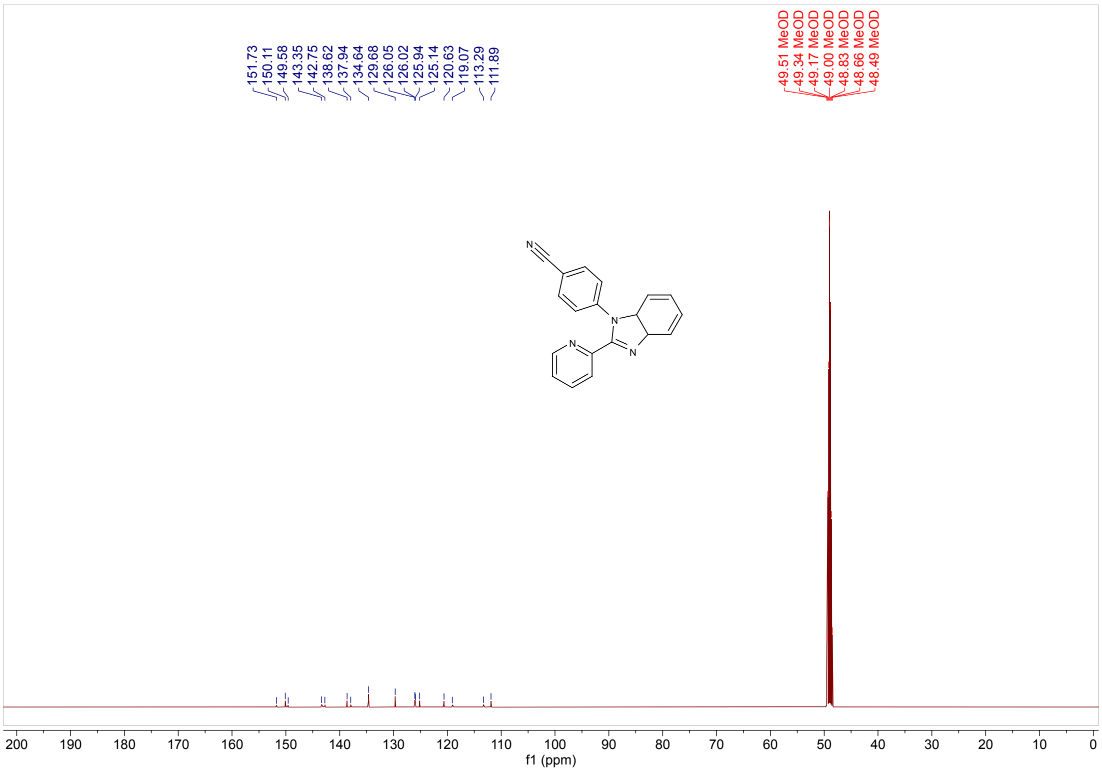

4-(2-(Pyridin-2-yl)-1*H*-benzo[*d*]imidazol-1-yl)benzamide (OSA_000989)

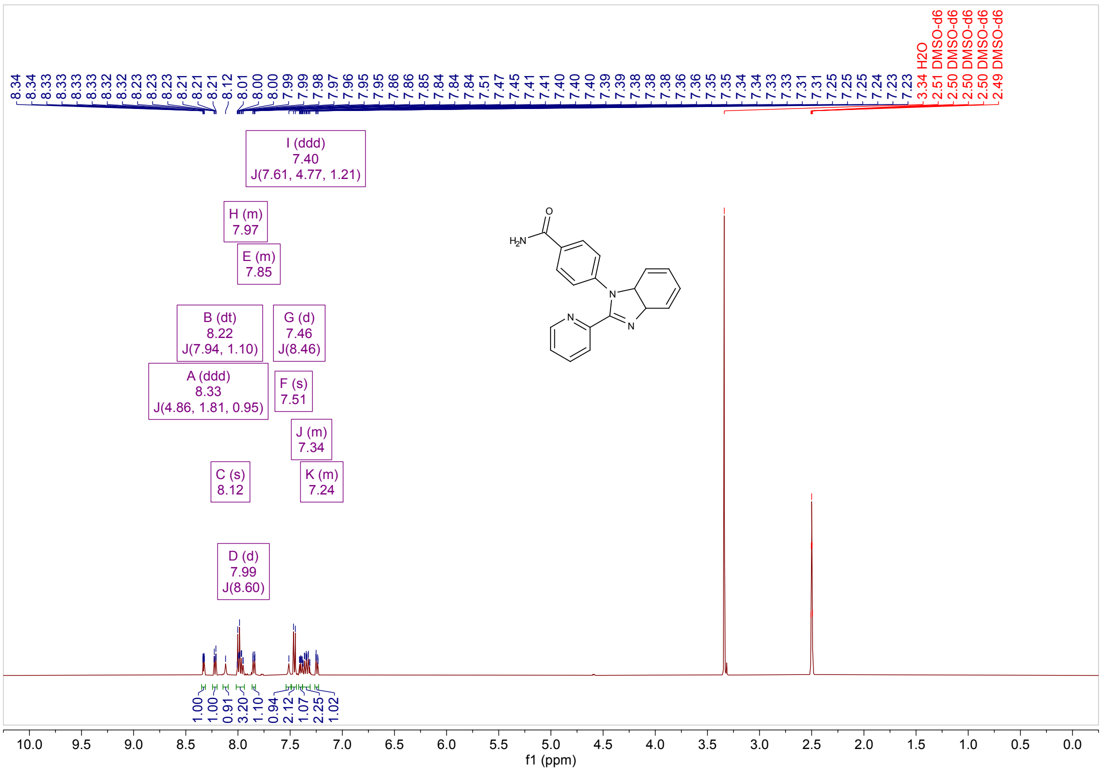

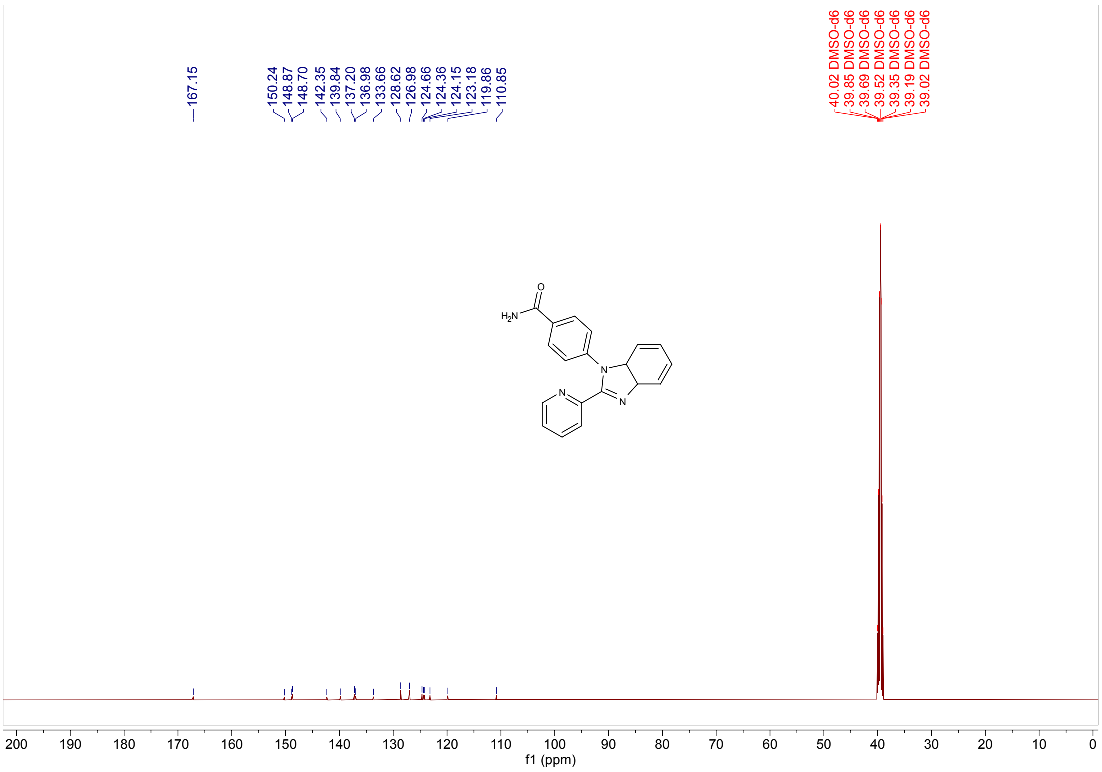

7-Methoxy-3-phenyl-2-(pyridin-2-yl)imidazo[1,2-*a*]pyridine (OSA_000860)

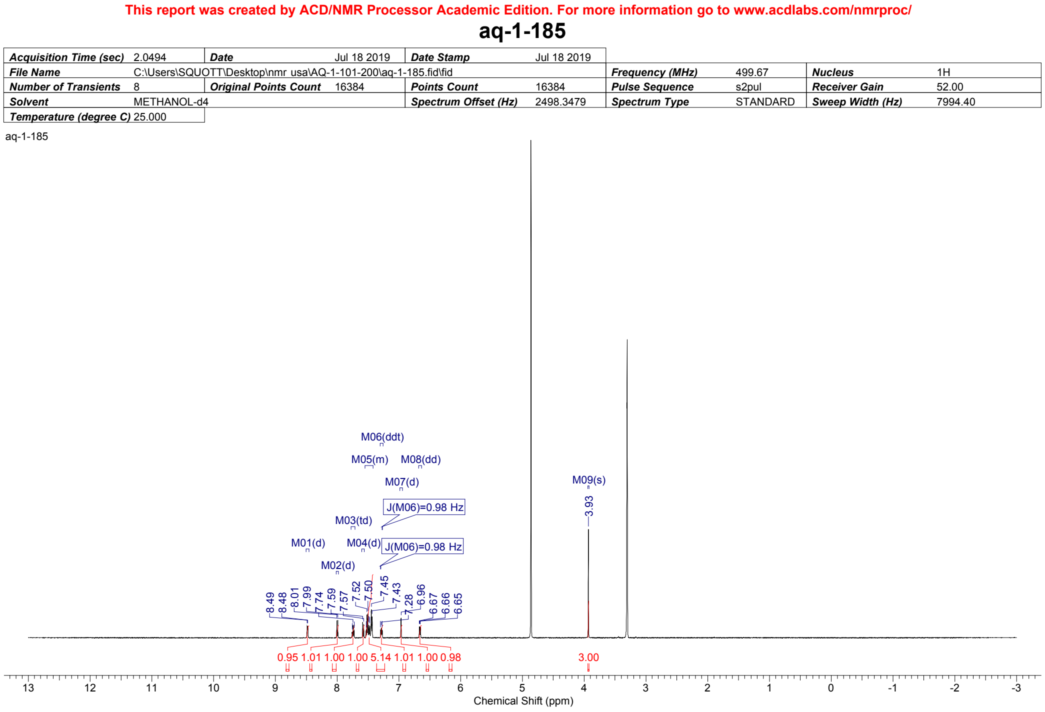

3-(4-Fluorophenyl)-7-methoxy-2-(pyridin-2-yl)imidazo[1,2-*a*]pyridine (OSA_000858)

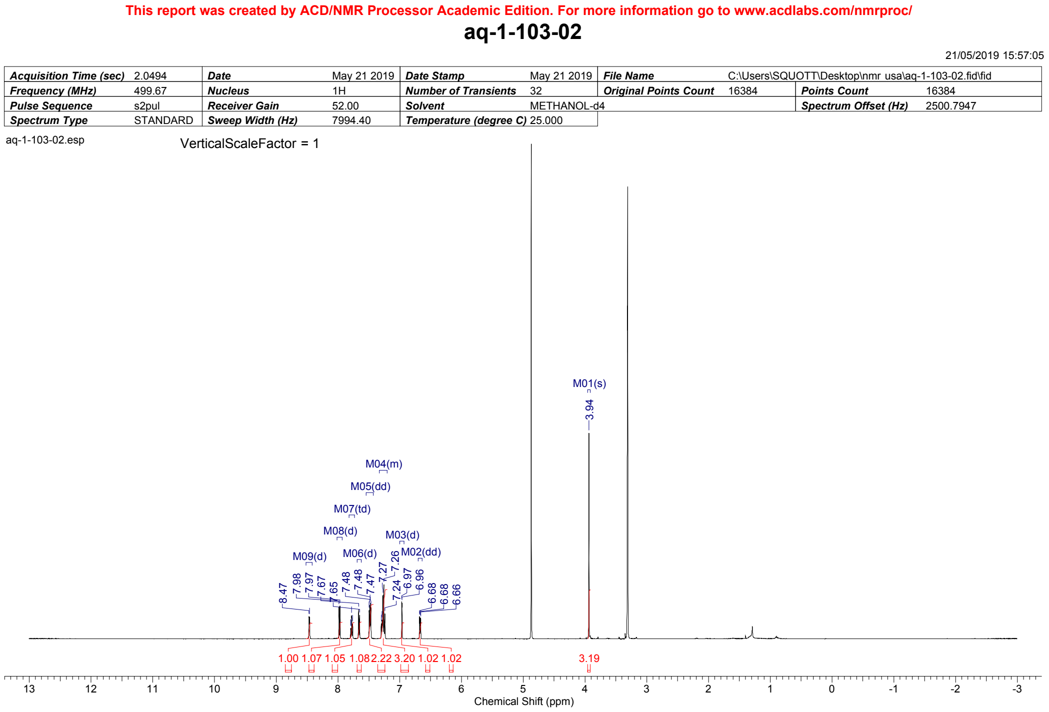

3-(6-Chloropyridin-2-yl)-7-methoxy-2-(pyridin-2-yl)imidazo[1,2-*a*]pyridine (OSA_000857)

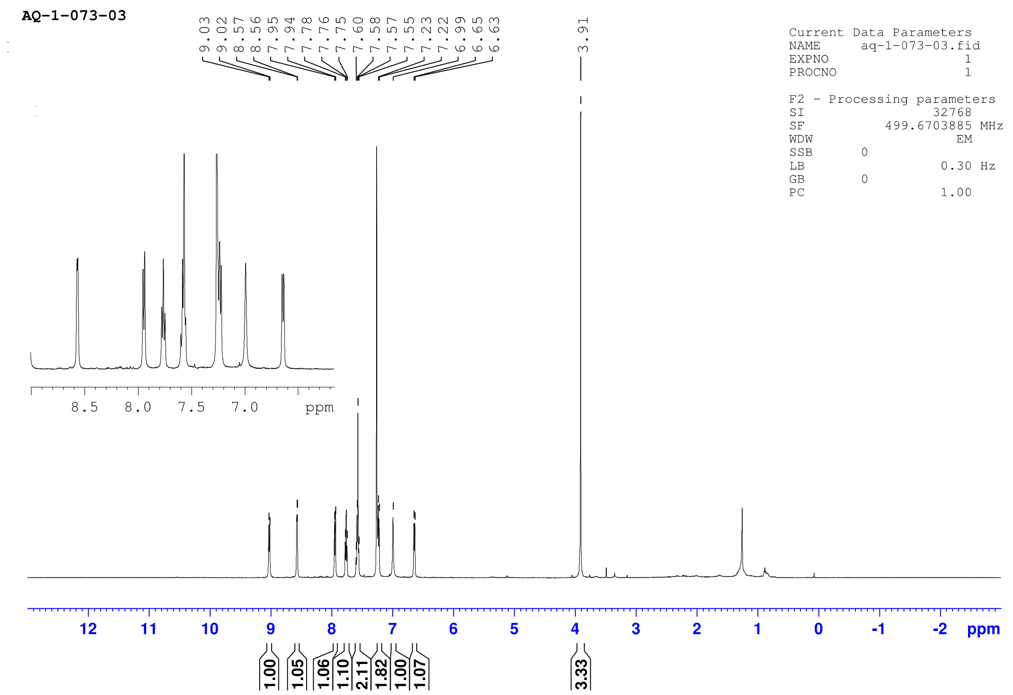

***N*-(2-Fluorophenyl)-7-methoxy-2-(1-methyl-1*H*-pyrazol-4-yl)imidazo[1,2-*a*]pyridin-3-amine (OSA_000855)**

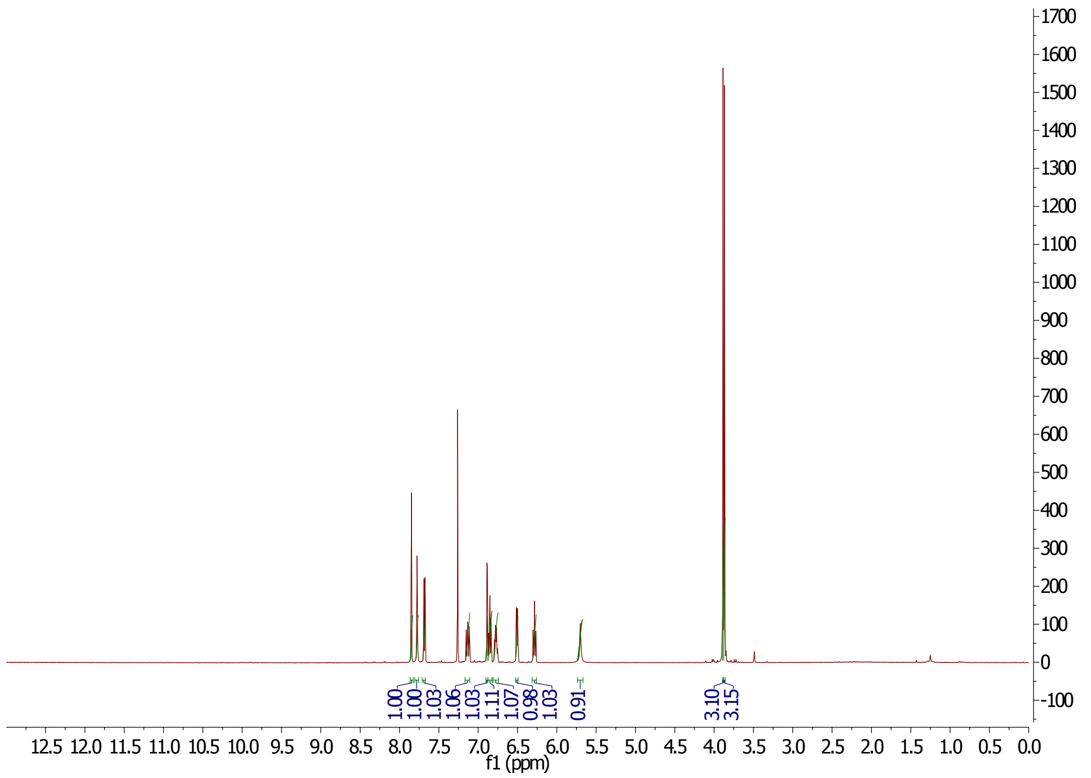

2-(4-(Dimethylamino)phenyl)-*N*-(4-fluorophenyl)-7-methylimidazo[1,2-*a*]pyridin-3-amine (OSA_000846)

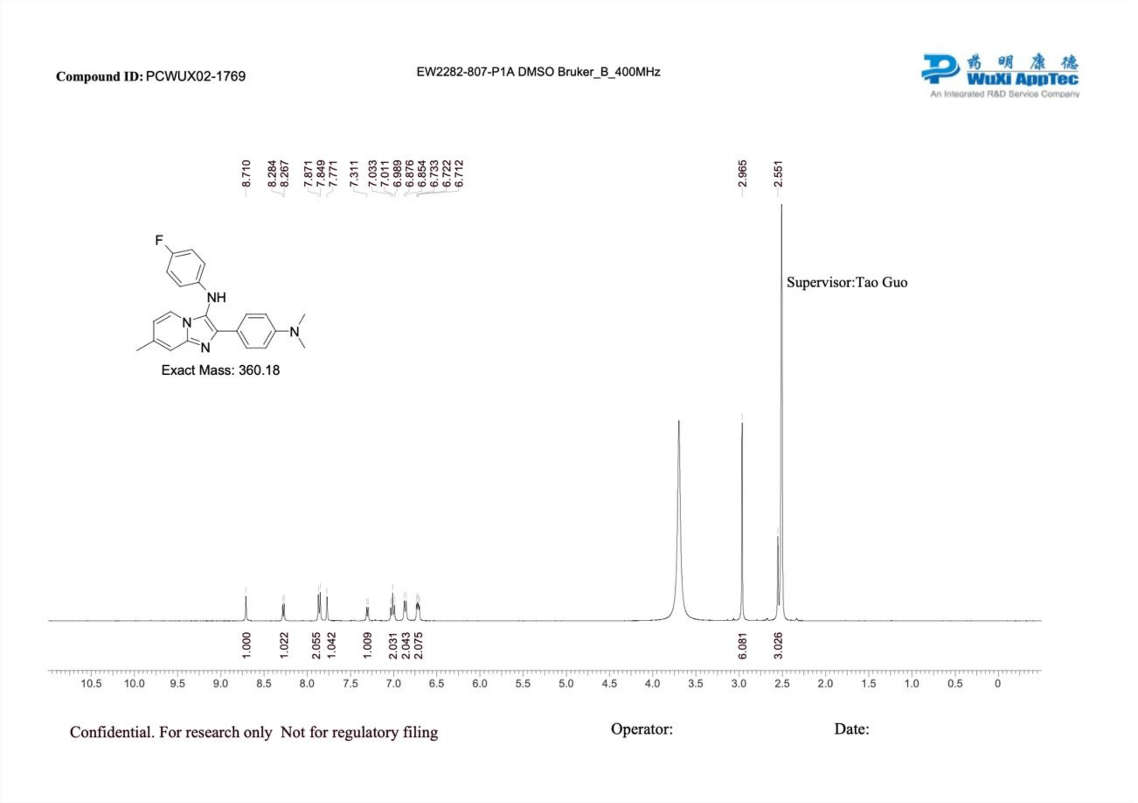

7-Methoxy-*N*-(2-methylpyridin-4-yl)-2-(pyridin-2-yl)imidazo[1,2-*a*]pyridin-3-amine (OSA_000854)

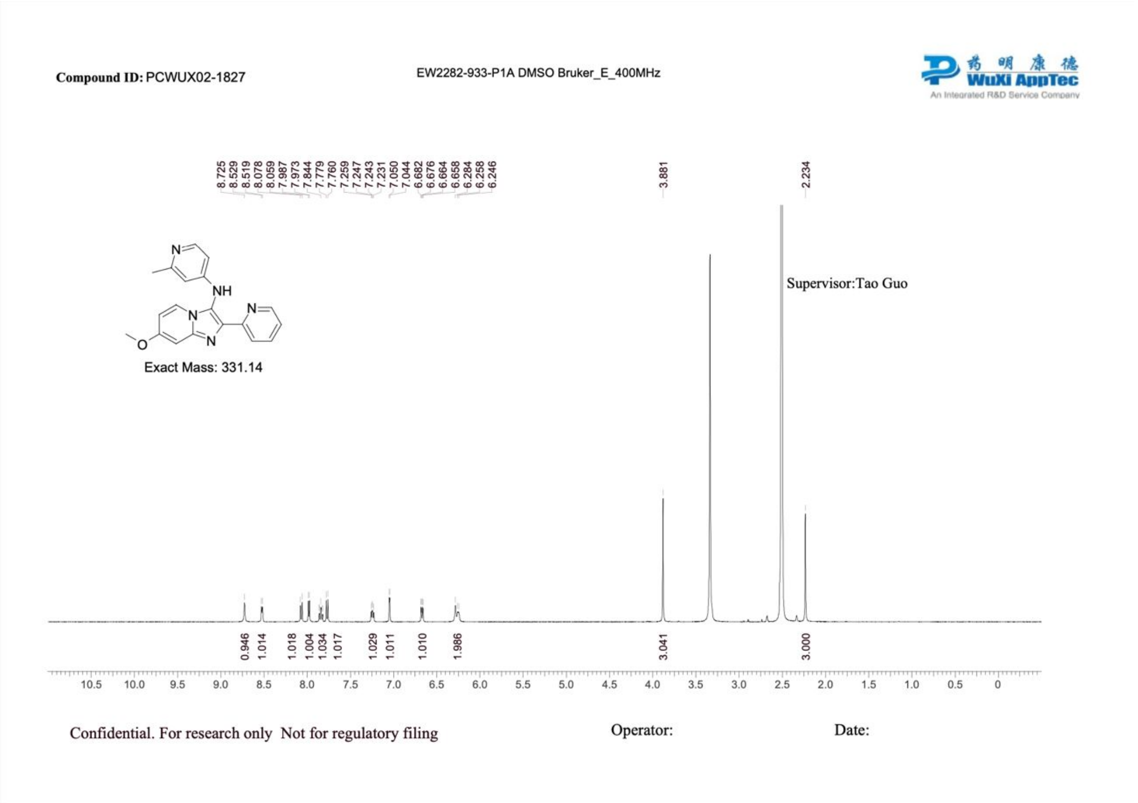

7-Methoxy-*N*-((1-methyl-1*H*-imidazol-4-yl)methyl)-2-(pyridin-2-yl)imidazo[1,2-*a*]pyridin-3-amine (OSA_000853)

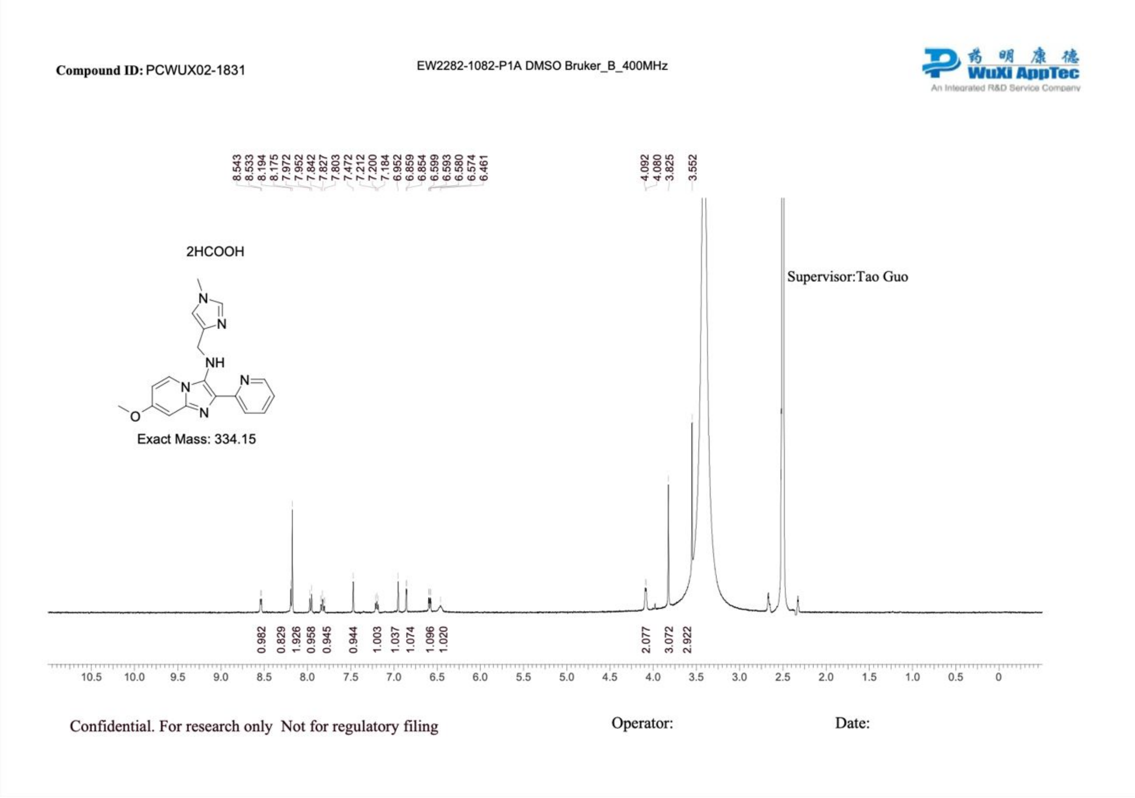

2-(3,4-Dimethoxyphenyl)-*N*-(furan-2-ylmethyl)-6-methylimidazo[1,2-*a*]pyridin-3-amine hydrochloride (OSA_000849)

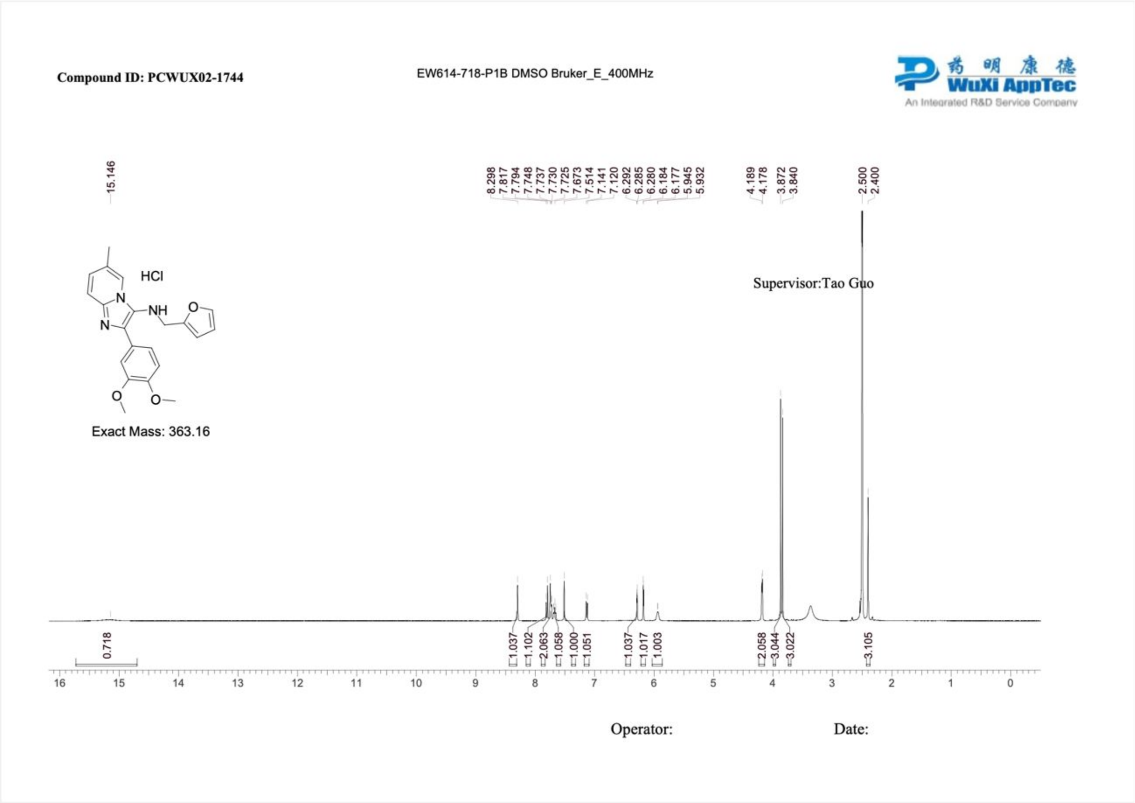

***N*-(2,3-Dihydrobenzo[*b*][1,4]dioxin-6-yl)-2-(1*H*-imidazol-4-yl)-7-methoxyimidazo[1,2-*a*]pyridin-3-amine (OSA_000866)**

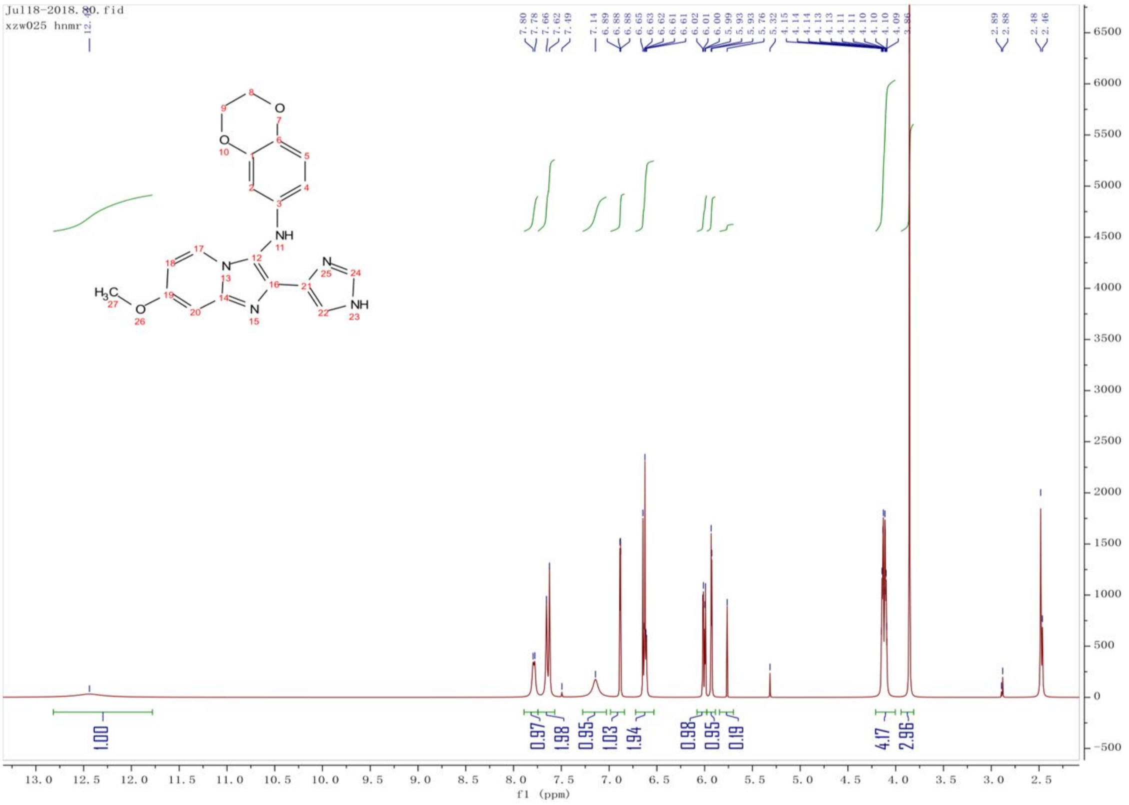

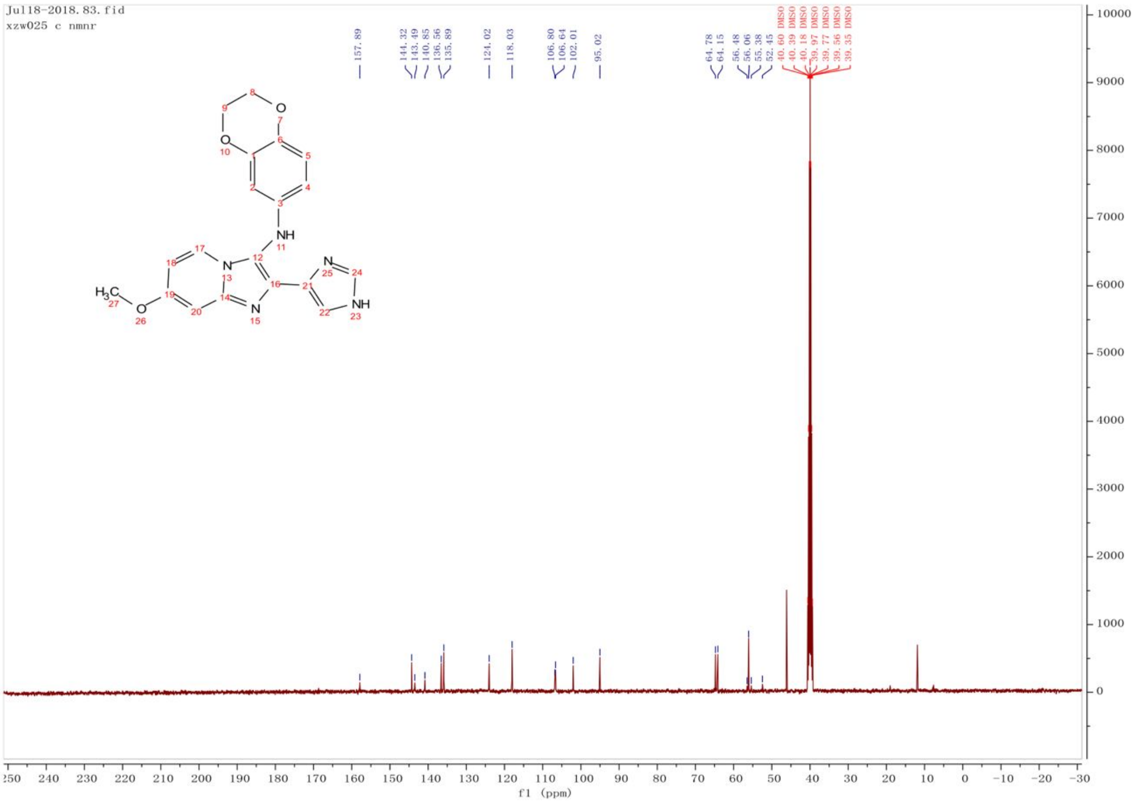

***N*-(2,3-dihydrobenzo[*b*][1,4]dioxin-6-yl)-2-(1*H*-imidazol-4-yl)-6-methoxyimidazo[1,2-*a*]pyridin-3-amine (OSA_000867)**

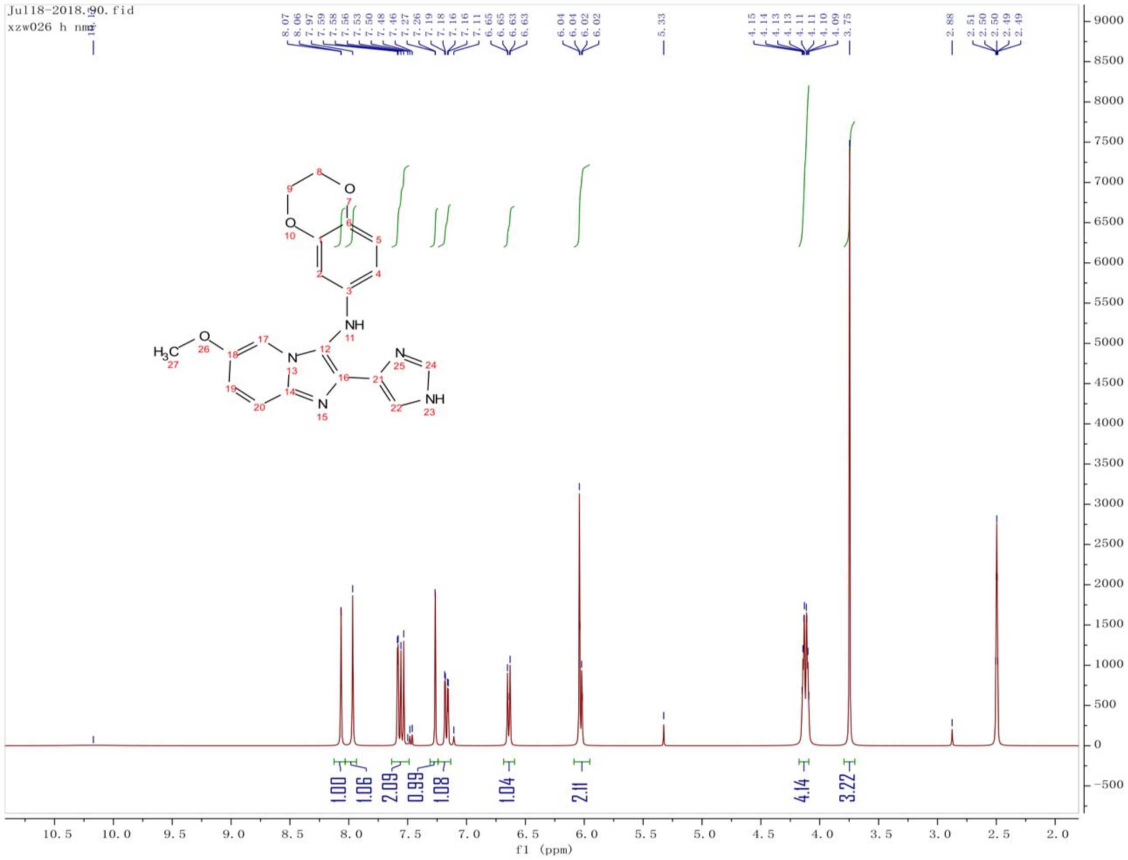

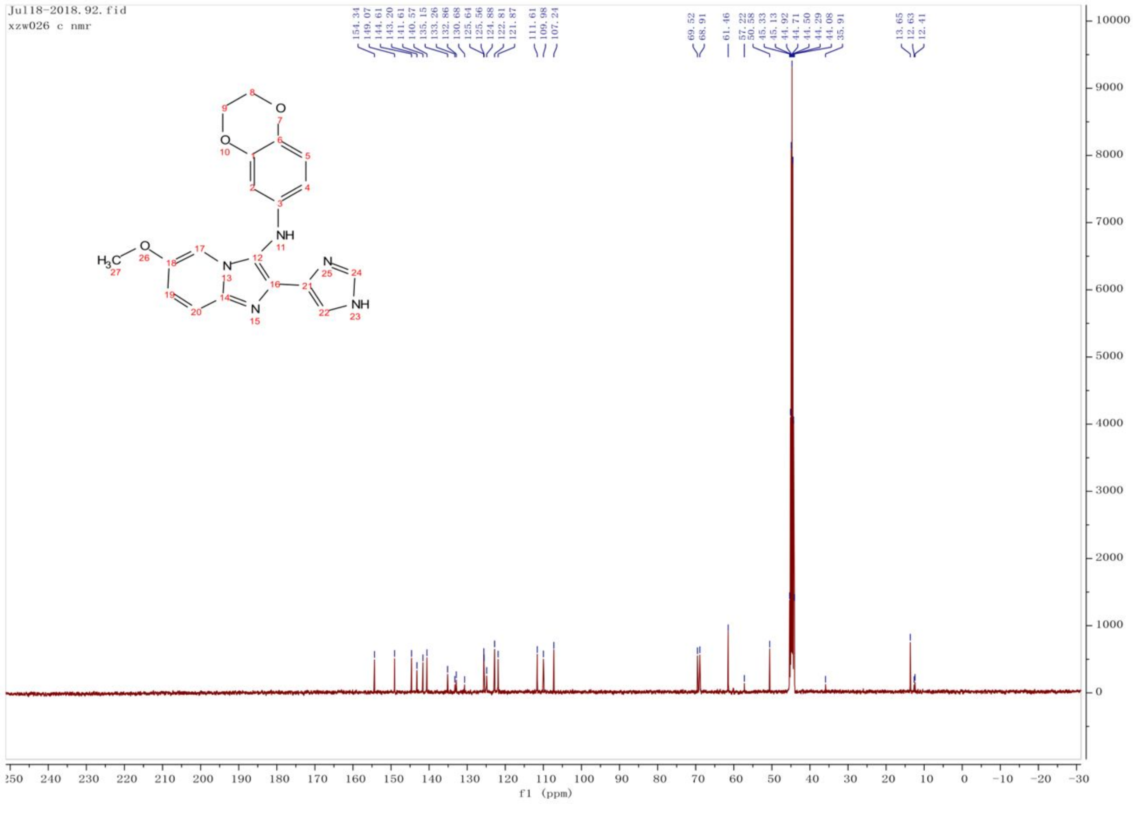

3-(Benzofuran-5-yl)-2-(pyridin-2-yl)-6,7-dihydro-5*H*-pyrrolo[1,2-*a*]imidazol-7-ol (OSA_000997)

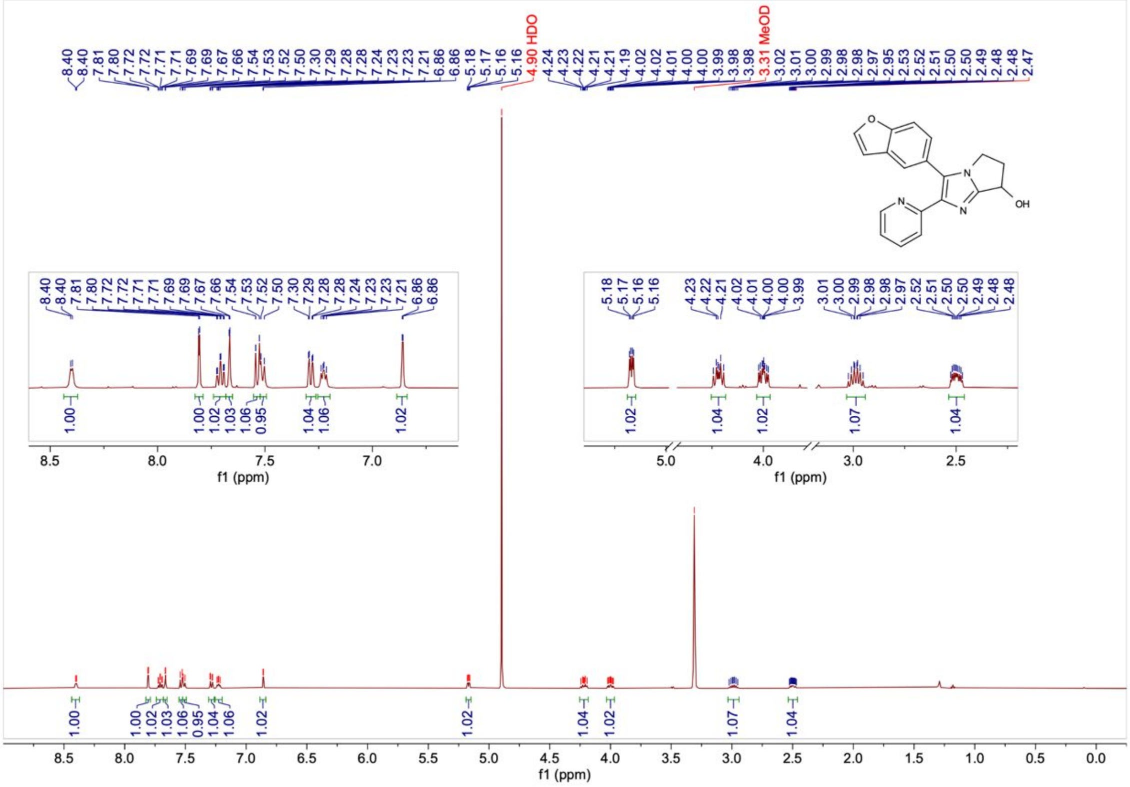

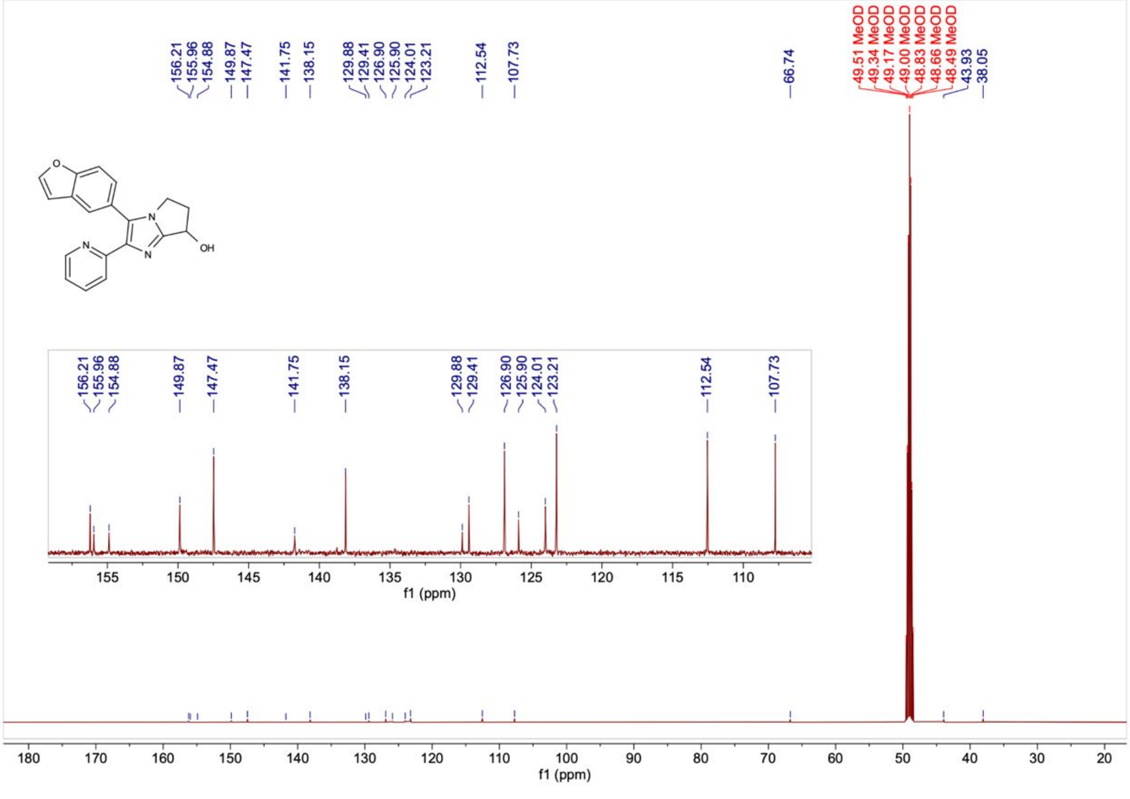

References

[1] Akao, Y.; Canan, S.; Cao, Y.; Condroski, K.; Engkvist, O.; Itono, S.; Kaki, R.; Kimura, C.; Kogej, T.; Nagaoka, K.; Naito, A.; Nakai, H.; Pairaudeau, G.; Radu, C.; Roberts, I.; Shimada, M.; Shum, D.; Watanabe, N.-a.; Xie, H.; Yonezawa, S.; Yoshida, O.; Yoshida, O.; Mowbray, C.; Perry, B. Collaborative Virtual Screening to Elaborate an Imidazo[1,2-*a*]pyridine Hit Series for Visceral Leishmaniasis. *RSC Med. Chem.* **2021**, *12*, 384-393. DOI: 10.1039/d0md00353k
[2] Wang, Y.; Frett, B.; Li, H.-y. Efficient Access to 2,3-Diarylimidazo[1,2-*a*]pyridines via a One-Pot, Ligand-Free, Palladium-Catalyzed Three-Component Reaction under Microwave Irradiation. *Org. Lett.* **2014**, *16*, 3016-3019. DOI: 10.1021/ol501136e
[3] Dichiara, M.; Simpson, Q. J.; Quotadamo, A.; Jalani, H. B.; Huang, A. X.; Millard, C. C.; Klug, D. M.; Tse, E. G.; Todd, M. H.; Gedder, D.; da Silva Emery, F.; Carlson, J. E.; Zheng, S.-L.; Vleminckx, M.; Matheeussen, A.; Caljon, G.; Pollastri, M. P.; Sjö, P.; Perry, B.; Ferrins, L., Structure-property Optimization of a Series of Imidazopyridines for Visceral Leishmaniasis, *ACS Infect. Dis.* **2023**, *in press* (id-2023-000406, accepted 01-Jun-2023).
[4] Wang, X.; Liu, W.-G.; Tung, C.-H.; Wu, L.-Z.; Cong, H. A Monophosphine Ligand Derived from Anthracene Photodimer: Synthetic Applications for Palladium-Catalyzed Coupling Reactions. *Org. Lett.* **2019**, *21*, 8158-8163. DOI: 10.1021/acs.orglett.9b02414
[5] Brown, M. F.; Che, Y.; Marfat, A.; Melnick, M. J.; Montgomery, J. I.; Reilly, U. *N*-linked Hydroxamic Acid Derivatives Useful as Antibacterial Agents. **2011**. WO2011073845A1
[6] Martin, F. M.; Mergott, D. J.; Owton, W. M. BACE Inhibitors. **2014**. WO2014066132A1
[7] Wang, F.; Jeon, K. O.; Salovich, J. M.; Macdonald, J. D.; Alvarado, J.; Gogliotti, R. D.; Phan, J.; Olejniczak, E. T.; Sun. Q.; Wang, S.; Camper, D.; Yuh, J. P.; Shaw, G.; Sai, J.; Rossanese, O. W.; Tansey, W. P.; Stauffer, S. R.; Fesik, S. W. Discovery of Potent 2-Aryl-6,7-dihydro-5*H*-pyrrolo[1,2-*a*]imidazoles as WDR5-WIN-Site Inhibitors Using Fragment-Based Methods and Structure-Based Design. *J. Med. Chem.* **2018**, *61*, 5623-5642. DOI: 10.1021/acs.jmedchem.8b00375
