# Supplementary material for: Open Source Antibiotics: Simple Diarylimidazoles Are Potent against Methicillin-Resistant Staphylococcus aureus
Source: ACS Infect Dis. 2023 Nov 22;9(12):2423–35. doi: 10.1021/acsinfecdis.3c00286 (PMC10714399; doi:10.1021/acsinfecdis.3c00286)
Supplement: Supplementary file 4 — id3c00286_si_003.pdf [file id3c00286_si_003.pdf]

MONASH University

CONFIDENTIAL

Physicochemical and metabolic evaluation of five compounds

UCL_Todd

Report #: CDCO_UCL_Todd_20_001

22 September 2020

Quality Statement:

This non-GLP study was conducted using established techniques in accordance with the relevant guidelines and standard operating procedures (SOPs) of the Centre for Drug Candidate Optimisation, Monash University. This report accurately reflects the raw data obtained during the performance of this study.

Director:	Susan Charman, PhD
Project Coordinator:	Karen White, PhD
Study Leader(s):	Michael Campbell, PhD<br>David Shackleford, PhD

Study contributor(s):	Caithlin White<br>Elly Crighton<br>Rahul Patil, PhD

Study number(s):	UCL_Todd_20_001

Centre for Drug Candidate Optimisation
Monash Institute of Pharmaceutical Sciences
Monash University (Parkville campus)
381 Royal Parade, Parkville, VIC 3052, Australia
ABN 12 377 614 012 | W: platforms.monash.edu/cdco/

cdco CENTRE FOR DRUG CANDIDATE OPTIMISATION

A. Experimental Methods

a) Calculated physicochemical parameters using ChemAxon JChem software

A range of physicochemical properties evaluating drug-likeness and likely oral absorption characteristics were calculated using the ChemAxon chemistry cartridge via JChem for Excel software (version 16.4.11). A brief description of each parameter is provided below, along with a suggested ideal range based on research reported in the ADME literature from key industry and academic sources.

MW (< 500): Molecular Weight.

$PSA_{pH\ 7.4}$ (< 140 Å^2): Polar surface area also inversely correlates with membrane permeability.

HBD (< 5) & HBA (< 10): Number of hydrogen bond donors and acceptors gives an indication of the hydrogen bonding capacity, which is inversely related to membrane permeability.

FRB (≤ 10): Number of freely rotating bonds represents the flexibility of a molecule's conformation.

Arom. Rings (< 4): Total number of aromatic and heteroaromatic rings is also related to molecular flexibility.

Fsp^3 (> 0.3): Fraction of sp^3 carbons to total carbons indicates the complexity of a molecule's 3D structure.

cpKa: Ionisation constants impact solubility and permeability. Only physiologically relevant predicted values are provided here (*i.e.* 0 < pKa < 12).

cLogP/$cLogD_{pH}$ (< 5): Partition coefficients reflect the lipophilic character of the neutral structure, while distribution coefficients reflect the partitioning properties of the ionised molecule at a specific pH.

b) Kinetic Solubility Estimation using Nephelometry (Sol_{pH})

Compound in DMSO was spiked into either pH 6.5 phosphate buffer or 0.01 M HCl (approx pH 2.0) with the final DMSO concentration being 1%. After 30 minutes had elapsed, samples were then analysed via Nephelometry to determine a solubility range. See Bevan and Lloyd (2000) Anal Chem, 72:1781-1787.

c) Distribution Coefficient Estimation using Chromatography ($gLogD_{pH}$)

Partition coefficient values (LogD) of the test compounds were estimated at pH 7.4 by correlation of their chromatographic retention properties against the characteristics of a series of standard compounds with known partition coefficient values. The method employed is a gradient HPLC based derivation of the method developed by Lombardo. See Lombardo *et al*. (2001) J Med Chem, 44:2490-2497.

d) *In Vitro* Metabolic Stability

Incubation:

The metabolic stability assay was performed by incubating each test compound with a suspension of liver microsomes (0.4 mg/mL, 37°C). The metabolic reaction was initiated by the addition of an NADPH-regenerating system and quenched at various time points over a 60 minute incubation by the addition of acetonitrile containing metolazone as internal standard. Control samples (containing no NADPH) were included (quenched at 2, 30 and 60 minutes) to monitor for potential degradation in the absence of cofactor.

The human liver microsomes used in this experiment were supplied by XenoTech, lot # 1710084.The rat liver microsomes used in this experiment were supplied by XenoTech, lot # 1610290. Microsomal incubations were performed at a substrate concentration of 1 µM.

Data analysis and interpretation:

Substrate depletion as a function of time was analysed as described by Obach (1999) Drug Metab. Dispos., 27:1350-1359. Intrinsic clearance (CL_{int}) values were classified at the limits of low and high clearance bands based on a rearrangement of the well stirred model of hepatic elimination. It should be noted that the potential impact of compound binding (either in microsomes or in blood) on the relationship between *in vitro* intrinsic clearance and hepatic clearance *in vivo* has not been accounted for in these studies.

Species	In Vitro Intrinsic Clearance (µL/min/mg protein)	
	**Low Clearance**	**High Clearance**
Human	< 11	> 59
Rat	< 17	> 92

B. Results

Experimental results are tabulated on the following page(s).

Table 1: Physicochemical and metabolic evaluation of five compounds

Compound (Batch)	Structure	MW	PSA $(Å^2)$	FRB	HBD	HBA	Arom. Rings	Fsp^3	Predicted pKa (0 - 12 only)	cLogP	cLogD at pH 7.4	gLogD at pH 7.4	$Sol_{2.0}$ (µg/mL)	$Sol_{6.5}$ (µg/mL)	Microsome Species	Cofactor	$T_{1/2}$ (min)	$CL_{int, in vitro}$ (µL/min/ mg protein)
UNC-ALM-DAI-27 (1)	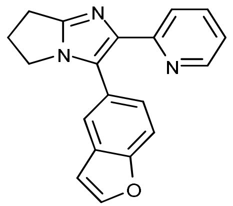	301.35	43.9	2	0	2	4	0.16	Basic: 3.1<br>Basic: 0.3<br>Acidic: none	3.4	3.4	1.9	> 100	25 - 50	Human	NADPH	34	51
															Rat	NADPH	24	72
UNC-ALM-DAI-28 (1)	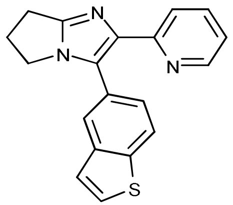	317.41	30.7	2	0	2	4	0.16	Basic: 3.1<br>Basic: 0.3<br>Acidic: none	4.1	4.1	2.3	50 - 100	12.5 - 25	Human	NADPH	24	72
															Rat	NADPH	3	503
UNC-ALM-DAI-16 (1)	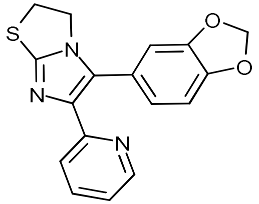	323.37	49.2	2	0	4	3	0.18	Basic: 1.4<br>Basic: 0.1<br>Acidic: none	3.4	3.4	1.6	25 - 50	12.5 - 25	Human	NADPH	47	37
															Rat	NADPH	23	74
UNC-ALM-DAI-18 (1)	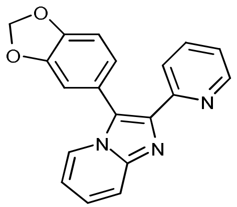	315.33	48.7	2	0	4	4	0.05	Basic: 2.6<br>Basic: 0.4<br>Acidic: none	3.2	3.2	1.8	> 100	25 - 50	Human	NADPH	37	47
															Rat	NADPH	18	99

**Compound (Batch)**	**Structure**	**MW**	**PSA** ($Å^2$)	**FRB**	**HBD**	**HBA**	**Arom. Rings**	**Fsp^3**	**Predicted pKa** (0 - 12 only)	**cLogP**	**cLogD** at pH 7.4	**gLogD** at pH 7.4	**Sol $_{2.0}$** (µg/mL)	**Sol $_{6.5}$** (µg/mL)	**Microsome Species**	**Cofactor**	**$T_{1/2}$** (min)	**$CL_{int, in vitro}$** (µL/min/ mg protein)
SB-400868 (1)	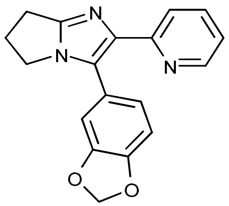	305.34	49.2	2	0	4	3	0.22	Basic: 3.1<br>Basic: 0.3<br>Acidic: none	2.8	2.8	1.5	> 100	50 - 100	Human	NADPH	62	28
															Rat	NADPH	29	59
