# Supplementary material for: Open Source Antibiotics: Simple Diarylimidazoles Are Potent against Methicillin-Resistant Staphylococcus aureus
Source: ACS Infect Dis. 2023 Nov 22;9(12):2423–35. doi: 10.1021/acsinfecdis.3c00286 (PMC10714399; doi:10.1021/acsinfecdis.3c00286)
Supplement: Supplementary file 5 — id3c00286_si_004.pdf [file id3c00286_si_004.pdf]

Community for Open Antimicrobial Drug Discovery

Hit-Confirmation

Antimicrobial screening, Cytotoxicity & Haemolysis

Procedure and Materials

Content

1.0 Summary 3
1.1 Study 3
1.2 Assay Parameters 3
1.3 Outcomes 3
1.4 Structural Novelty 4
1.5 Publishing CO-ADD Data 4
2.0 Methods 5
2.1 Sample Preparation 5
2.2 Antibacterial Assay 5
2.2.1 Procedure 5
2.2.2 Analysis 5
2.3 Antifungal Assay 6
2.3.1 Procedure 6
2.3.2 Analysis 6
2.4 Cytotoxicity Assay 7
2.4.1 Procedure 7
2.4.2 Analysis 7
2.5 Haemolysis Assay 7
2.5.1 Procedure 7
2.5.2 Analysis 7
2.6 Antibiotic, Cytotoxic and Haemolytic Standards Preparation and Quality Control 8
3.0 Materials 9
3.1 Assay Materials 9
3.2 Standards 9
3.3 Microbial strains and cell lines 9
4.0 Controls 10
4.1 Antimicrobial susceptibility of tested strains 10
4.1.1 Antibiotic standards 10
4.1.2 Antifungal standard 10
4.2 Susceptibility profile of cell lines 10
4.3 Susceptibility profile of human washed red cells 11
4.4 Outcome 11

1.0 Summary

1.1 Study

Hit Confirmation of active compounds by whole cell growth inhibition assays was conducted as an 8-point dose response to determine the Minimum Inhibitory Concentration (MIC), in duplicate (n=2). The inhibition of growth is measured against those microorganisms that showed susceptibility to the compounds tested in the Primary Screen.

Included in the Hit Confirmation were 5 bacteria: *Escherichia coli*, *Klebsiella pneumoniae, Acinetobacter baumannii, Pseudomonas aeruginosa* and *Staphylococcus aureus*, and 2 fungi *Candida albicans* and *Cryptococcus neoformans*.

In addition to determining MIC, active compounds were counter screened for cytotoxicity against a human embryonic kidney cell line, HEK293, by determining their CC_{50} value. The compounds were also screened for haemolysis of human red blood cells.

1.2 Assay Parameters

Assay Parameters	Bacteria	Fungi	HEK293	Haemolysis
Test concentration	32 - 0.25 µg/mL or 20 – 0.15 µM ≤0.5% DMSO	32 - 0.25 µg/mL or 20 – 0.15 µM ≤0.5% DMSO	32 - 0.25 µg/mL or 20 – 0.15 µM ≤0.5% DMSO	32 - 0.25 µg/mL or 20 – 0.15 µM ≤0.5% DMSO
QC	Duplicate (n=2) Control MIC: Pass	Duplicate (n=2) Control MIC: Pass	Duplicate (n=2) Control CC_{50}: Pass	Duplicate (n=2) Control HC_{10}: Pass
Plates	Non-Binding Surface (NBS), 384-well plate	Non-Binding Surface (NBS), 384-well plate	TC, 384-well black wall/clear bottom	Polypropylene 384-well and polystyrene 384-well plates
Media	Cation-adjusted Mueller Hinton broth	Yeast Nitrogen Base	DMEM supplemented with 10% FBS	0.9% NaCl
Read Out	OD_{600}	OD_{630} Resazurin $OD_{600-570}$	Resazurin $F_{560/590}$	OD_{405}

1.3 Outcomes

Hit Confirmation outcomes are detailed in individual Project reports, personalised for each Project Submission for each CO-ADD user.

Please see your data sheet with file extension **P0XXX_HC_data.xlsx,** for example CO-ADD Project **P0100, P0100_HC_data.xlsx**

1.4 Structural Novelty

As per the T&C's of CO-ADD, structures for all submitted compounds for antimicrobial screening should be disclosed to CO-ADD following Primary Screening. Without structures for all submitted compounds, Hit Confirmation assays will not be triggered.

If you have not already done so, please **provide CO-ADD with the chemical structure** of the full sample set in this study (both for compounds showing activity and those that do not), which will allow CO-ADD to filter out future samples with the same, or highly similar structure. In addition, please **notify CO-ADD** if you agree to publish the data (*i.e.* structures and activity) in the public bioactive database ChEMBL (www.ebi.ac.uk/chembl/). CO-ADD aims to increase the public knowledge of antimicrobial research, including data about non-active compounds.

All confirmed hits, without cytotoxicity or haemolytic activity, will be considered for further Hit-Validation, after a detailed analysis of structure-activity relationship and antimicrobial novelty, within CO-ADD samples, as well as, within public antimicrobial activity databases, like ChEMBL (www.ebi.ac.uk/chembl/).

1.5 Publishing CO-ADD Data

If you wish to publish data provided by CO-ADD, we kindly ask that you acknowledge CO-ADD appropriately with the following reference:

> Helping Chemists Discover New Antibiotics
> M.A. Blaskovich, J. Zuegg, A.G. Elliott, M.A. Cooper
> *ACS Infect. Dis.*, **2015**, 1(7), 285-287.
> DOI: 10.1021/acsinfecdis.5b00044; PMID: 27622818

as well as an acknowledgement for the funding of CO-ADD:

> *"The antimicrobial screening performed by CO-ADD (The Community for Antimicrobial Drug Discovery) was funded by the Wellcome Trust (UK) and The University of Queensland (Australia)."*

Please advise CO-ADD at your earliest convenience that you have used provided data for publication purposes. This information is extremely helpful in keeping track of the outputs from the CO-ADD initiative and supports the program in renewed funding possibilities to continue CO-ADD as a free screening service available to the academic community.

CO-ADD also asks, that where possible you publish your data in an Open Access journals.

2.0 Methods

2.1 Sample Preparation

Samples were provided by the collaborator and stored frozen at -20 °C. Samples were prepared in DMSO and water to a final testing concentration of 32 µg/mL or 20 µM (unless otherwise indicated in the data sheet) and serially diluted 1:2 fold for 8 times. Each sample concentration was prepared in 384-well plates, non-binding surface plate (**NBS;** Corning 3640) for each bacterial/fungal strain, tissue-culture treated (**TC-treated;** Corning 3712/3764) black for mammalian cell types and polypropylene 384-well (**PP**; Corning 3657) for haemolysis assays, all in duplicate (n=2), and keeping the final DMSO concentration to a maximum of 0.5%. All the sample preparation was done using liquid handling robots.

Compounds that showed notable solubility issues during stock solution preparation are detailed in the **Data sheet** for the individual Project.

2.2 Antibacterial Assay

2.2.1 Procedure

All bacteria were cultured in Cation-adjusted Mueller Hinton broth (**CAMHB**) at 37 °C overnight. A sample of each culture was then diluted 40-fold in fresh broth and incubated at 37 °C for 1.5-3 h. The resultant mid-log phase cultures were diluted (CFU/mL measured by OD_{600}), then added to each well of the compound containing plates, giving a cell density of 5×10^5 CFU/mL and a total volume of 50 µL. All the plates were covered and incubated at 37 °C for 18 h without shaking.

2.2.2 Analysis

Inhibition of bacterial growth was determined measuring absorbance at 600 nm (OD_{600}), using a Tecan M1000 Pro monochromator plate reader. The percentage of growth inhibition was calculated for each well, using the negative control (media only) and positive control (bacteria without inhibitors) on the same plate as references.

The percentage of growth inhibition was calculated for each well, using the negative control (media only) and positive control (bacteria without inhibitors) on the same plate. The MIC was determined as the lowest concentration at which the growth was fully inhibited, defined by an inhibition ≥ 80%. In addition, the maximal percentage of growth inhibition is reported as D_{Max}, indicating any compounds with partial activity.

Hits were classified by MIC ≤ 16 µg/mL or MIC ≤ 10 µM in either replicate (n=2 on different plates).

2.3 Antifungal Assay

2.3.1 Procedure

Fungi strains were cultured for 3 days on Yeast Extract-Peptone Dextrose (**YPD**) agar at 30 °C. A yeast suspension of 1 x 10^6 to 5 x 10^6 CFU/mL (as determined by OD_{530}) was prepared from five colonies. The suspension was subsequently diluted and added to each well of the compound-containing plates giving a final cell density of fungi suspension of 2.5 $\times 10^3$ CFU/mL and a total volume of 50 µL. All plates were covered and incubated at 35 °C for 36 h without shaking.

2.3.2 Analysis

Growth inhibition of *C. albicans* was determined measuring absorbance at 630 nm (OD_{630}), while the growth inhibition of *C. neoformans* was determined measuring the difference in absorbance between 600 and 570 nm ($OD_{600\text{-}570}$), after the addition of resazurin (0.001% final concentration) and incubation at 35 °C for 2 h. The absorbance was measured using a Biotek Multiflo Synergy HTX plate reader.

In both cases, the percentage of growth inhibition was calculated for each well, using the negative control (media only) and positive control (fungi without inhibitors) on the same plate. The MIC was determined as the lowest concentration at which the growth was fully inhibited, defined by an inhibition ≥ 80% for *C. albicans* and an inhibition ≥ 70% for *C. neoformans.* Due to a higher variance in growth and inhibition, a lower threshold was applied to the data for *C. neoformans*. In addition, the maximal percentage of growth inhibition is reported as D_{Max}, indicating any compounds with marginal activity.

Hits were classified by MIC ≤ 16 µg/mL or MIC ≤ 10 µM in either replicate (n=2 on different plates).

2.4 Cytotoxicity Assay

2.4.1 Procedure

HEK293 cells were counted manually in a Neubauer haemocytometer and then plated in the 384-well plates containing the compounds to give a density of 5000 cells/well in a final volume of 50 µL. **DMEM** supplemented with **10% FBS** was used as growth media and the cells were incubated together with the compounds for 20 h at 37 °C in 5% CO_2.

2.4.2 Analysis

Cytotoxicity (or cell viability) was measured by fluorescence, ex: 560/10 nm, em: 590/10 nm ($F_{560/590}$), after addition of 5 µL of 25 µg/mL resazurin (2.3 µg/mL final concentration) and after incubation for further 3 h at 37 °C in 5% CO_2. The fluorescence intensity was measured using a Tecan M1000 Pro monochromator plate reader, using automatic gain calculation.

CC_{50} (concentration at 50% cytotoxicity) were calculated by curve fitting the inhibition values *vs.* log(concentration) using a sigmoidal dose-response function, with variable fitting values for bottom, top and slope. In addition, the maximal percentage of cytotoxicity is reported as D_{Max}, indicating any compounds with partial cytotoxicity.

The curve fitting was implemented using Pipeline Pilot's dose-response component, resulting in similar values to curve fitting tools such as GraphPad's Prism and IDBS's XlFit. Any value with > indicate sample with no activity (low D_{Max} value) or samples with CC_{50} values above the maximum tested concentration (higher D_{Max} value).

Cytotoxic samples were classified by CC_{50} ≤ 32 µg/mL or CC_{50} ≤ 10 µM in either replicate (n=2 on different plates). In addition, samples were flagged as partial cytotoxic if D_{Max} ≥ 50%, even with CC_{50} > the maximum tested concentration.

2.5 Haemolysis Assay

2.5.1 Procedure

Human whole blood was washed three times with 3 volumes of 0.9% NaCl and then resuspended in same to a concentration of 0.5 x 10^8 cells/mL, as determined by manual cell count in a Neubauer haemocytometer. The washed cells were then added to the 384-well compound-containing plates for a final volume of 50 µL. After a 10 min shake on a plate shaker the plates were then incubated for 1 h at 37 °C. After incubation, the plates were centrifuged at 1000*g* for 10 min to pellet cells and debris, 25 µL of the supernatant was then transferred to a polystyrene 384-well assay plate.

2.5.2 Analysis

Haemolysis was determined by measuring the supernatant absorbance at 405 mm (OD_{405}). The absorbance was measured using a Tecan M1000 Pro monochromator plate reader.

HC_{10} and HC_{50} (concentration at 10% and 50% haemolysis, respectively) were calculated by curve fitting the inhibition values *vs*. log(concentration) using a sigmoidal dose-response function with variable fitting values for top, bottom and slope. In addition, the maximal

percentage of haemolysis is reported as D_{Max}, indicating any compounds with partial haemolysis.

The curve fitting was implemented using Pipeline Pilot's dose-response component, resulting in similar values to curve fitting tools such as GraphPad's Prism and IDBS's XlFit. Any value with > indicate sample with no activity (low D_{Max} value) or samples with HC_{10} values above the maximum tested concentration (higher D_{Max} value).

Haemolysis samples were classified by HC_{10} ≤ 32 µg/mL or HC_{10} ≤ 10 µM in either replicate (n=2 on different plates). In addition, samples were flagged as partial haemolytic if D_{Max} ≥ 50%, even with HC_{10} > the maximum tested concentration.

2.6 Antibiotic, Cytotoxic and Haemolytic Standards Preparation and Quality Control

Colistin and Vancomycin were used as positive bacterial inhibitor standards for Gram-negative and Gram-positive bacteria, respectively. Fluconazole was used as a positive fungal inhibitor standard for *C. albicans and C. neoformans.* Tamoxifen was used as a positive cytotoxicity standard. Melittin was used as a positive haemolytic standard.

Each antibiotic standard was provided in 4 concentrations, with 2 above and 2 below its MIC or CC_{50} value, and plated into the first 8 wells of column 23 of the 384-well NBS plates. Tamoxifen and melittin was used in 8 concentrations in 2 fold serial dilutions with 50 µg/mL highest concentration.

The quality control (QC) of the assays was determined by Z'-Factor, calculated from the Negative (media only) and Positive Controls (bacterial, fungal or cell culture without inhibitor), and the Standards. Plates with a Z'-Factor of ≥ 0.4 and Standards active at the highest and inactive at the lowest concentration, were accepted for further data analysis.

3.0 Materials

3.1 Assay Materials

Material	*Code*	*Brand/Supplier*	*Cat No.*
Compound preparation plate, Polypropylene	PP	Corning	3364
Assay Plates – Antimicrobial Non-binding surface	NBS 384w	Corning	3640
Assay Plates – Cytotoxicity Tissue culture treated	Black/Clear bottom 384w	Corning	3712
Assay Plates - Haemolysis	PP-Haem	Corning	3657
Reading Plates - Haemolysis	Clear 384w	Corning	3680
Growth media - bacteria	CAMHB	Bacto Laboratories	212322
Culture agar - fungi	YPD	Becton Dickinson	242720
Growth media - fungi	YNB	Becton Dickinson	233520
Resazurin		Sigma-Aldrich	R7017
Dulbecco's Modified Eagle Medium	DMEM	Life Technologies	11995-073
Foetal Bovine Serum	FBS	Bovogen	FFBS-500
0.9% NaCl	Saline	Baxter	AHF7124

3.2 Standards

Sample Name	*Sample ID*	*Full MW*	*Stock Conc. (mg/mL)*	*Solvent*	*Source*
Colistin - Sulfate	MCC_000094:02	1400.63	10.0	DMSO	Sigma; C4461
Vancomycin - HCL	MCC_000095:02	1485.71	10.0	DMSO	Sigma; 861987
Fluconazole	MCC_008383:01	306.27	2.56	DMSO	Sigma; F8929
Tamoxifen	MCC_000096:01	371.50	10	DMSO	Sigma; T5648
Melittin	MCC_008868:02	2846.46	10	Water	Sigma: M2272

3.3 Microbial strains and cell lines

ID	*Batch*	*Organism*	*Strain*	*Description*
GN_001	02	*Escherichia coli*	ATCC 25922	FDA control strain
GN_003	02	*Klebsiella pneumoniae*	ATCC 700603	MDR
GN_034	02	*Acinetobacter baumannii*	ATCC 19606	Type strain
GN_042	02	*Pseudomonas aeruginosa*	ATCC 27853	Quality control strain
GP_020	02	*Staphylococcus aureus*	ATCC 43300	MRSA
FG_001	01	*Candida albicans*	ATCC 90028	CLSI reference
FG_002	01	*Cryptococci neoformans*	ATCC 208821	H99, Type strain
MA_007	02	*Homo sapiens* embryonic kidney cells	ATCC CRL-1573	HEK 293
HA_150	-	*Homo sapiens*	ARCBS 5400 00150	Whole blood

4.0 Controls

4.1 Antimicrobial susceptibility of tested strains

Values are the average of ≥ 6 independent biological replicates. All values are within the expected range as per CLSI guidelines.

4.1.1 Antibiotic standards

MIC determined by BMD method, CA-MHB, Corning 3640 384 NBS plates		**GN_001:02**<br>***Escherichia coli***<br>FDA Control<br>ATCC 25922	**GN_003:02**<br>***Klebsiella pneumophila***<br>ESBL<br>ATCC 700603	**GN_034:02**<br>***Acinetobacter baumannii***<br>Type strain<br>ATCC 19606	**GN_042:02**<br>***Pseudomonas aeruginosa***<br>QC strain<br>ATCC 27853
Compound	Compound Type	MIC (µg/mL)			
Colistin - sulfate	Antibiotic	**0.125**	**0.25**	**0.25**	**0.25**

MIC determined by BMD method, CA-MHB, Corning 3640 384 NBS plates		**GP_020:02**<br>***Staphylococcus aureus***<br>MRSA<br>ATCC 43300
Compound	Compound Type	MIC (µg/mL)
Vancomycin - HCl	Antibiotic	**1**

4.1.2 Antifungal standard

MIC determined by BMD method, YNB, Corning 3640 384 NBS plates		**FG_001:02**<br>***Candida albicans***<br>CLSI reference<br>ATCC 90028	**FG_002:02**<br>***Cryptococcus neoformans H99***<br>Type strain<br>ATCC 208821
Compound	Compound Type	MIC (µg/mL)	
Fluconazole	Antifungal	**0.125**	**8**

4.2 Susceptibility profile of cell lines

Values are the average of > 6 independent biological replicates. CC_{50} is the concentration at 50% cytotoxicity.		**MA_007**<br>**HEK293**<br>**ATCC CRL-1573**	
		CC_{50} (µg/mL)	
Compound	Compound Type	Average	Stdev
Tamoxifen	PKC inhibitor	**9**	**2.2**

4.3 Susceptibility profile of human washed red cells

Values are the average of > 6 independent biological replicates. HC_{10} and HC_{50} are the concentrations at 10% and 50% haemolysis, respectively.		**HA_150 Human Whole blood ARCBS 00150**			
		HC_{10} (µg/mL)		HC_{50} (µg/mL)	
Compound	Compound Type	Average	Stdev	Average	Stdev
Melittin	Haemolytic peptide	**2.7**	**0.9**	**8.5**	**2.5**

4.4 Outcome

All standard compound controls displayed inhibitory values within the expected range for each assay type and each organism tested. For further information please contact the CO-ADD team at support@co-add.org.

Strain ID	***Species***	***Standard positive inhibitor control***	***Pass/Fail***
GN_001:02	*E. coli*	Colistin	Pass
GN_003:02	*K. pneumoniae* (MDR)	Colistin	Pass
GN_034:02	*A. baumannii*	Colistin	Pass
GN_042:02	*P. aeruginosa*	Colistin	Pass
GP_020:02	*S. aureus* (MRSA)	Vancomycin	Pass
FG_001:01	*C. albicans*	Fluconazole	Pass
FG_002:01	*C. neoformans* (H99)	Fluconazole	Pass
MA_007:02	*Homo sapiens* HEK293	Tamoxifen	Pass
HA_150	*Homo sapiens*	Melittin	Pass
